# Fast and cost-effective SARS-CoV-2 variant detection using Oxford Nanopore full-length spike gene sequencing

**DOI:** 10.1099/mgen.0.001013

**Published:** 2023-05-18

**Authors:** Cecilia Salazar, Ignacio Ferrés, Mercedes Paz, Alicia Costábile, Gonzalo Moratorio, Pilar Moreno, Gregorio Iraola

**Affiliations:** ^1^​ Laboratorio de Genómica Microbiana, Institut Pasteur de Montevideo, Montevideo, Uruguay; ^2^​ Centro de Innovación en Vigilancia Epidemiológica, Institut Pasteur de Montevideo, Montevideo, Uruguay; ^3^​ Laboratorio de Evolución Experimental de Virus, Institut Pasteur de Montevideo, Montevideo, Uruguay; ^4^​ Laboratorio de Virología Molecular, Facultad de Ciencias, Universidad de la República, Montevideo, Uruguay; ^5^​ Sección Bioquímica, Facultad de Ciencias, Universidad de la República, Montevideo, Uruguay; ^6^​ Wellcome Sanger Institute, Hinxton, UK; ^7^​ Centro de Biología Integrativa, Universidad Mayor, Santiago de Chile, Chile

**Keywords:** SARS-CoV-2, spike gene, Oxford Nanopore Technologies, surveillance

## Abstract

Most biologically relevant and diagnostic mutations in the severe acute respiratory syndrome coronavirus-2 (SARS-CoV-2) genome have been identified in the S gene through global genomic surveillance efforts. However, large-scale whole-genome sequencing (WGS) is still challenging in developing countries due to higher costs, reagent delays and limited infrastructure. Consequently, only a small fraction of SARS-CoV-2 samples are characterized through WGS in these regions. Here, we present a complete workflow consisting of a fast library preparation protocol based on tiled amplification of the S gene, followed by a PCR barcoding step and sequencing using Nanopore platforms. This protocol facilitates fast and cost-effective identification of main variants of concern and mutational surveillance of the S gene. By applying this protocol, report time and overall costs for SARS-CoV-2 variant detection could be reduced, contributing to improved genomic surveillance programmes, particularly in low-income regions.

## Data Summary

The authors confirm all supporting data, code and protocols have been provided within the article or through supplementary data files. Supplementary files are available with the online version of this article. Sequencing data generated in this study have been deposited in SRA under BioProjects PRJNA895020 and PRJNA895694. Protocols are provided through protocols.io links and code is available through https://github.com/microgenlab/spike.

**Impact Statement**

The development of an alternative cost-effective tool to assist in the epidemiological surveillance of SARS-CoV-2 is relevant to improve variant detection and characterization, especially in the context of limited resources. This could also be useful when high-throughput screening of samples is needed prior to whole genome sequencing. Furthermore, the procedures described here could be easily adaptable to detect and characterize different pathogens based on defined marker sequences and has the potential to transcend its immediate applicability in the ongoing COVID-19 pandemic.

## Introduction

The constant development and improvement of straightforward and cost-effective methods that allow tracking of infectious diseases are paramount for real-time pathogen surveillance, as has been clearly demonstrated during the ongoing coronavirus disease 2019 (COVID-19) pandemic. COVID-19 rapidly spread to different countries after its first identification in Wuhan (China), being declared a pandemic by the World Health Organization (WHO) on 11 March 2020. Severe acute respiratory syndrome coronavirus 2 (SARS-CoV-2), the causative agent of COVID-19, is an enveloped virus with a single-stranded positive sense RNA genome [1–3]. SARS-CoV-2 binds to the host angiotensin-converting enzyme 2 (ACE2) through the spike (S) protein, mediating cell entry [4]. Since the first complete genome sequences of SARS-CoV-2 were made publicly available in late December 2019, over 13 million sequences from all over the world have been submitted to the EpiCoV database at the Global Initiative on Sharing All Influenza Data (GISAID) (https://gisaid.org/) [5]. The fast and timely availability of all the genomic data facilitated the development of molecular diagnostic tests and also guided the investigation of viral transmission chains and genetic variants through active genomic surveillance [6, 7].

Different nomenclature systems are used to identify SARS-CoV-2 genetic variants [8, 9]. A widely used system is the dynamic PANGO nomenclature [10]. New PANGO lineages are designated only if a certain lineage contains at least five sequences with high genome coverage (≥95 %), while remaining sequences not included in the designation set can be assigned as an estimation of a lineage [10, 11]. Furthermore, a more recent designation for SARS-CoV-2 genetic variants, which defines viral transmissibility, disease severity, reinfection rates (i.e. escape from natural immunity) and vaccine effectiveness (i.e. escape from vaccine-induced immunity), was established. These are categorized in variants of interest (VOIs), variants of concern (VOCs) and variants of high consequence (VHCs). While no VHCs have been identified so far, five VOCs were determined based on specific mutations: B.1.1.7 (Alpha), B.1.351 (Beta), P.1 (Gamma), B.1.617.2 (Delta) and B.1.1.529 (Omicron) [12]. To date, the majority of circulating VOC are of Omicron and its descendant lineages. Most VOC-defining mutations have been identified in the S gene [13], which has accumulated mutations since SARS-CoV-2 emergence in 2019 and is expected to continue to diverge. For this reason, S gene-based lineage assignment is possible under the concept of ‘lineage set’, which considers that different lineages share identical spike sequences [14].

The standard approach to assess SARS-CoV-2 variants is through whole geneome sequencing (WGS). However, variant-specific quantitative reverse transcriptase (RT)-PCR assays for top-priority circulating lineages have been developed to reduce costs and significantly decrease reporting times [15]. Unfortunately, these approaches provide static information that does not allow identification of the most recent and dynamically emerging SARS-CoV-2 variants. To overcome this, a middle ground strategy has been implemented using high-throughput Illumina sequencing of the S gene, enhancing the deployment of real-time variant surveillance through cost reduction and scalability [16].

In this work, we present a complete workflow for fast and cost-effective surveillance of SARS-CoV-2 variants based on the complete S gene sequence. This strategy uses the Oxford Nanopore platforms and it is based on the tiled primer amplification approach, widely used for SARS-CoV-2 WGS [17, 18]. In this proof-of-concept, the workflow showed that the spike gene consensus sequences generated with Oxford Nanopore platforms are suitable to detect VOCs and VOIs, when reaching a reasonable S gene completeness and sequencing depth, allowing fast and scalable screening at a lower cost when compared to WGS.

## Methods

### SARS-CoV-2 clinical samples

A total of 44 residual de-identified extracted RNA samples from SARS-CoV-2 positive patients were referred to the Centro de Innovación en Vigilancia Epidemiológica (CIVE) sequencing facility at the Institut Pasteur de Montevideo (IPMon). IPMon was validated by the Ministry of Health of Uruguay as a diagnostic testing centre for COVID-19 and national SARS-CoV-2 genomic sequencing. Further sample details can be found in Table S1, available in the online version of this article.

### SARS-CoV-2 whole-genome amplification and sequencing

RNA samples were first reverse transcribed using the LunaScript RT SuperMix Kit (New England Biolabs) as described for the PCR tiling of SARS-CoV-2 virus – rapid barcoding (SQK-RBK110.96) ONT protocol. Genome amplification was performed using the Q5 Hot Start High-Fidelity 2× Master Mix (New England Biolabs) with a two-pool multiplex-PCR strategy based on the Primal Scheme approach [17]. The xGen SARS-CoV-2 Midnight Amplicon Panel (IDT) was used to amplify SARS-CoV-2 from reversed transcribed samples; barcoding and sequencing adapter attachment were performed as previously described [19].

### Base calling, demultiplexing and genome consensus sequence

High-accuracy base calling and demultiplexing was performed using Guppy 5.0.12 (https://nanoporetech.com/). A Nanopolish workflow was used to generate the consensus genome sequences within the poreCov pipeline [20]. SARS-CoV-2 PANGO lineage, Nextrain clade and completeness were assessed using pangolin v4.1.2 [11], Nextclade CLI v1.10.3 [21] and president v0.6.3 [22].

### Standard S gene sequencing using ONT PCR barcoding amplicon sequencing

RNA extracted from SARS-CoV-2-positive samples was reverse-transcribed using the LunaScript RT SuperMix Kit (New England Biolabs). First-round PCR was performed with the Q5 Hot Start High-Fidelity 2× Master Mix (New England Biolabs) and with 0.6 µM final concentration of ONT-tagged ARTIC V3 primers (PCR mix) spanning the spike gene sequence in a two-pool format A and B (Table S2) [17]. The primers were selected based on the PCR tiling of the SARS-CoV-2 spike protein gene with rapid barcoding and Spike Seq RT PCR Expansion (SQK-RBK110.96 and EXP-SRT001) ONT protocol. The PCR consisted of 10.5 µl of PCR master mix with 2 µl of reversed-transcribed product. The PCR thermal profile consisted of an initial denaturation of 30 s at 98 °C, followed by 20 cycles of 15 s at 98 °C and 3 min at 63 °C. PCR amplicons from pool A and B were combined and diluted 1 : 10. ONT barcodes (EXP-PBC096) were added using a PCR barcoding strategy. The PCR barcoding step consisted of 6 µl Q5 Hot Start High-Fidelity 2× Master Mix, 1 µl of PCR barcode primers and 5 µl of the 1 : 10 diluted amplicons. The thermal profile for the barcoding step consisted of an initial denaturation of 30 s at 98 °C, followed by 15 cycles of 7 s at 98 °C, 15 s at 62 °C and 30 s at 72 °C, and a final step of 2 min at 72 °C. Barcoded samples were pooled and cleaned using 0.5× volume of AMPure XP and 1 µg was end-prepped. After the end-prep reaction, the barcoded pool was cleaned using a 0.5× volume of AMPure XP beads and the AMX sequencing adapter was ligated using the NEBNext Quick T4 DNA Ligase (New England Biolabs). Approximately 800 ng was loaded into a FLO-MIN106D flow cell which was run for a maximum of 18 h. This method is hereafter referred to as Standard-S. A detailed description of the Standard-S library preparation protocol is provided online: https://www.protocols.io/view/standard-s-pcr-barcoding-of-sars-cov-2-s-gen-ampli-b2c4qayw.

### Fast S gene sequencing using ONT amplicon sequencing

Spike gene amplification and sample barcoding was performed in a two-round PCR strategy using a single tube per sample, as opposed to the Standard-S protocol in which the amplification and barcoding take place in two separate sets of PCR tubes and PCR master mixes. For the Fast-S protocol, PCR barcoding primers were immobilized in the tube cap based on a previously described method [23]. First-round amplification occurs at the bottom of the tube using the LongAmp Taq 2× Master Mix (New England Biolabs) and 0.6 µM ONT-tagged ARTIC V3 primers spanning the spike gene sequence in a two-pool format A and B as described above (Table S2). A second round of PCR was performed using the same PCR master mix dissolving the previously immobilized ONT PCR barcoding primers EXP-PBC096 (final concentration 0.8 µM) by tube inversion. The thermal profile for the second round of PCR consisted of an initial denaturation of 3 min at 95 °C, followed by 15 cycles of 15 s at 95 °C, 15 s at 62 °C and 50 s at 65 °C, and a final extension at 65 °C for 10 min. After samples were pooled and cleaned using AMPure XP beads 0.5× a repair and end-prep step was performed using the NEBNext Ultra II End Repair Mix and NEBNext FFPE master mix (New England Biolabs). Sequencing adapter (AMX) was ligated and ~300–800 ng of DNA library was loaded into a FLO-MIN106D flow cell. A sequencing run was stopped after approximately 12 h. This method is hereafter referred to as Fast-S and further protocol details can be found online: https://www.protocols.io/view/fast-s-single-tube-amplification-and-pcr-barcoding-81wgbypn3vpk/v1.

### Lineage assignment using the spike gene sequence

Spike gene consensus sequences were generated using Medaka [24] as implemented in the epi2me-lab/wf-artic Nextflow pipeline using the spike-seq, V1 scheme [17, 25]. The SARS-CoV-2 PANGO lineage set was assigned using hedgehog v1.0.19 (https://github.com/cov-lineages/hedgehog) [14], and president v0.6.3 [22] was used to determine gene completeness using the spike region of SARS-CoV-2 WIV04 (GISAID accession EPI_ISL_402124) as the reference sequence. Read sampling was perfomed with fastq-tools (https://github.com/dcjones/fastq-tools). Average sequencing depth was obtained using samtools v1.14 [26]. Significant differences among S gene sequencing depth and completeness were assessed using the Wilcoxon test as implemented in the ggsignif package [27]. Since Gamma, Delta and Omicron variants comprise a diverse set of sublinages, an abbreviated version was used for comparative purposes. Lineages were suffixed with an asterisk (‘.*”) to denote those with different sublineages (i.e. Omicron BA.1.1 is represented as BA.1.*).

### Assessment of Omicron sublineages using the S gene

To assess the feasibility of using the spike gene sequence as a genetic marker for a wider diversity of Omicron samples, 5000 Omicron VOC genomes were randomly retrieved from the EpiCoV/GISAID database [5] (EPI_SET_220929wd). Sequences were aligned using Nextalign CLI v1.4.5 [21] with the WIV04 genome as a reference sequence. The alignment positions from 21 076 to 26 315 containing the S gene sequence were manually extracted with AliView [28] and the PANGO lineage was inferred from the trimmed alignment using hedgehog v1.0.19, as described above. Genome-based and spike-based PANGO lineage assignments were compared and visualized using ggplot2 [29].

## Results

### The S gene sequence is useful to detect lineages of the main SARS-CoV-2 VOCs

First, we aimed to assess the suitability of classifying the main circulating VOIs and VOCs based on the consensus sequence of the S gene generated using a standard protocol (Standard-S) based on ONT amplicon sequencing (see Methods). Additionally, hedgehog v1.0.19 was used for lineage assignment of 44 SARS-Cov-2 samples from which their whole genome and S gene consensus sequences were generated. Lineage assignment was concordant between S-based and whole-genome-based approaches in ~70 % of samples (Table S3). The range of PANGO lineages corresponding to each lineage set is shown in Table S4.

**Fig. 1.**
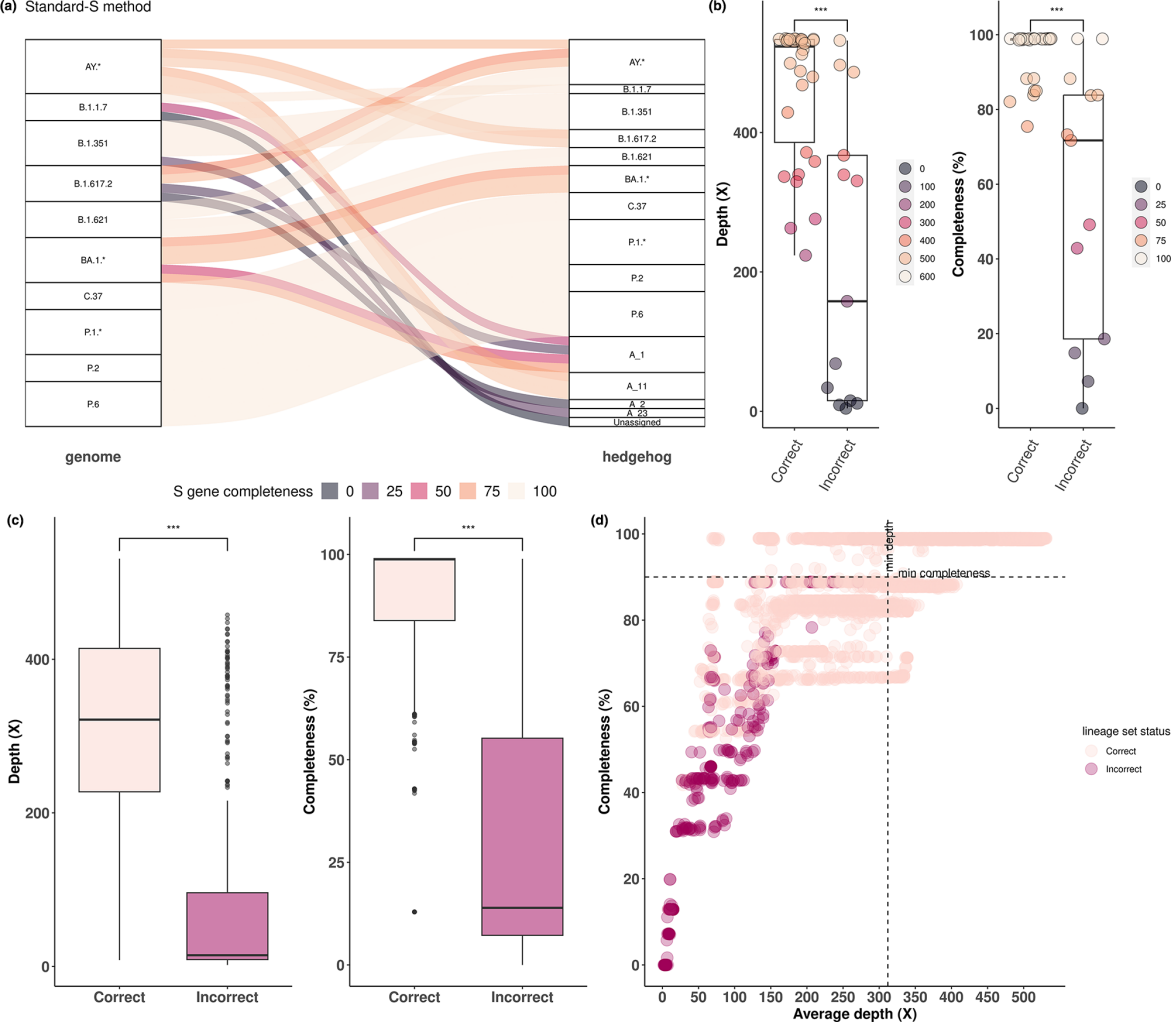
Spike-only SARS-CoV-2 lineage inference of the main VOCs, VOIs and non-VOC/VOIs (P.2 and P.6) obtained with the Standard-S protocol. (**a**) Comparison of the lineage assignment obtained between WGS- and S gene-based lineage classification. Connections between lineage assignments reflect the S gene completeness percentage of the gene. (**b**) Correct and incorrect lineage set assignment was assessed in terms of average sequencing depth (×) and S gene completeness (%). Significant differences were observed between correct and incorrect assignments (***P≤0.001). (**c**) Correct and incorrect lineage-set assignment from consensus spike-only sequences generated with read subsamples (500–10000) of each experimental sample. Significant differences were observed between correct and incorrect lineage-set assignments in terms of sequencing depth and S gene completeness. (**d**) Optimal sequencing depth and S gene completeness. Correct lineage assignment is maximized when both sequencing depth and S gene completeness are above the mean values for correct classification (top right quadrant).

Discrepancies with respect to genome-based classifications were observed for a set of PANGO lineages. Incorrect lineage set assignments were related to AY.* samples (*n*=2), B.1.1.7 (*n*=2), B.1.351 (*n*=1), B.1.617.2 (*n*=4), B.1.621 (*n*=2) and BA.1 (*n*=2). Overall, misassigned samples had a lower S gene completeness (48.5 ±33.7  %), with the exception of B.1.621 samples (~98.9 %) (Fig. 1a). Further details of the assignment results are given in Table S3. Significant differences (*P*≤0.001) between correct and incorrect lineage set assignments were observed in terms of sequencing depth and S gene completeness. Correct assignments resulted in an average sequencing depth of 463±99× and an S gene completeness of 95 ±7  %, whereas incorrect assignments resulted in an average sequencing depth of 219±210*×* and 56 ±36 % of S gene completeness (Fig. 1b).

To further understand the effect of the sequencing depth and gene completeness in the classification of VOCs, VOIs and non-VOC/VOI samples, S gene reads from each experimental sample were sampled (500 to 10000) and each resulting consensus sequence was reassigned (Figs S1 and S2). As with the experimental samples, a significant difference was observed in terms of sequencing depth and S gene completeness required for correct lineage assignment (Fig. 1c). A mean of 90±15 % gene sequence completeness and 312±127× average sequencing depth was required for correct PANGO lineage set assignment. In total, 97 % of sampling points above this cut-off threshold (*n*=1862) were correctly assigned to a PANGO lineage set. This suggests that samples that reach this cut-off criterion are assigned with a high level of confidence (Fig. 1d).

Together, our results on both real and simulated data show that the S gene sequences generated with the Standard-S protocol are suitable for SARS-CoV-2 lineage assignment with a spike-specific tool when achieving a reasonable completeness and sequencing depth of the S gene.

### VOCs and VOIs can be classified at lower costs using the Fast-S sequencing protocol

After implementing a standard protocol based on amplicon sequencing, we noted that there was still room for scaling up the screening capability in a single run, decreasing the costs and turnaround time. As a result, the Fast-S procedure was developed. Its main difference with respect to the Standard-S protocol is that the multiplex amplification and the indexing of samples occurs in a single tube using the same PCR master mix for both steps. The S gene amplification step takes place in the bottom of the tube and the indexing occurs once the amplification is finished using previously immobilized PCR barcodes in the tube caps, which are resuspended in the PCR mix by inversion (see Methods). Also, the PCR tubes are not open until the amplified material is barcoded, decreasing potential cross-contamination between samples.

**Fig. 2.**
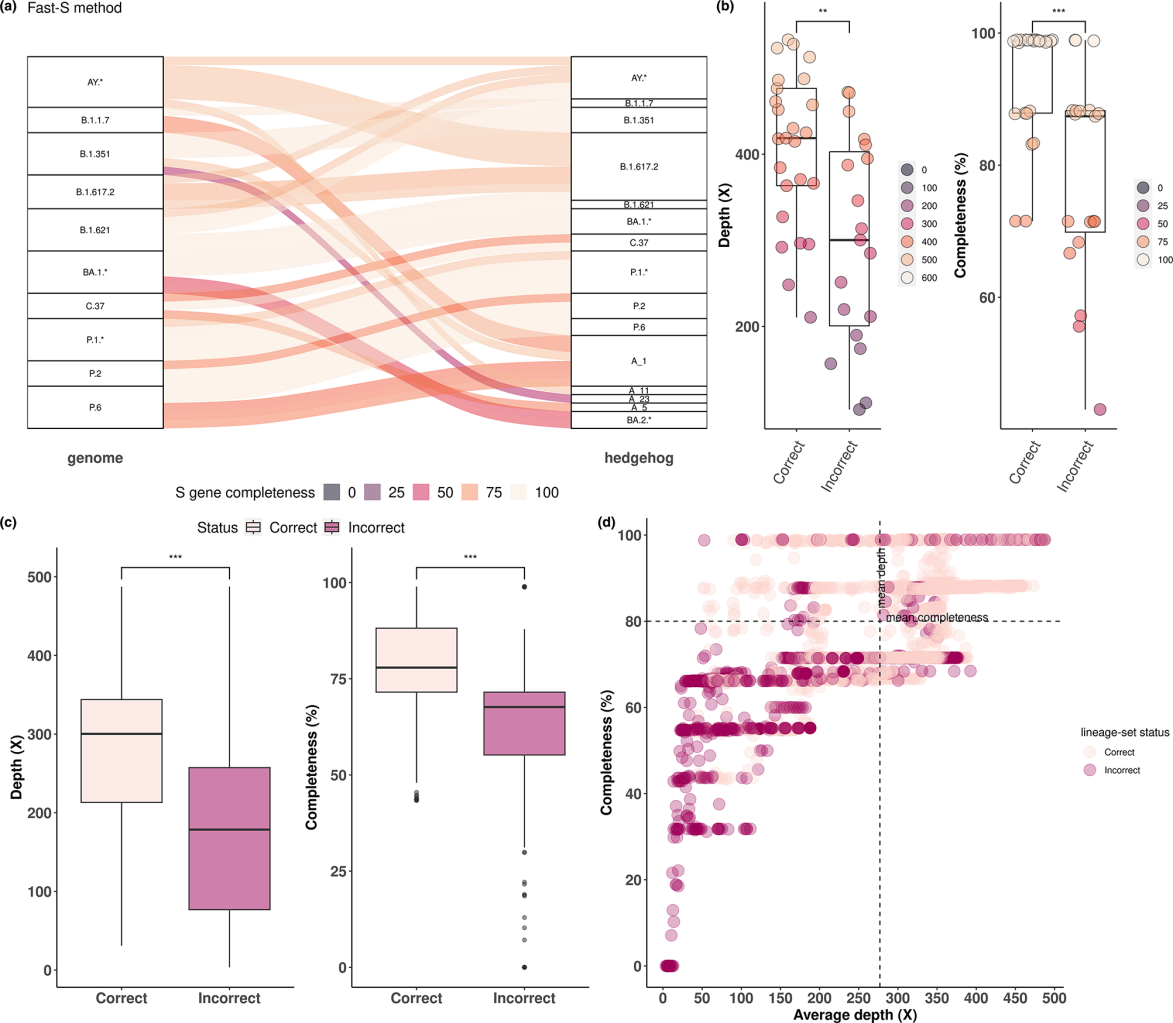
Spike-only SARS-CoV-2 lineage inference of the experimental dataset of VOCs, VOIs and non-VOCs/VOIs (P.2 and P.6) using the Fast-S method. (**a**) Comparison of the lineage assignment obtained with WGS- and S gene-based lineage classification. Connections between lineages reflect the S gene completeness percentage of the gene. (**b**) Correct and incorrect lineage assignment results were assessed in terms of sequencing depth and S gene completeness. Significant differences were observed between correct and incorrect assignments. (**c**) Correct and incorrect lineage-set assignment from consensus spike-only sequences generated with read subsamples (500–10000) of each experimental sample. Significant differences were observed between correct and incorrect lineage-set assignments in terms of sequencing depth and S gene completeness. (**d**) Optimal sequencing depth and S gene completeness. Correct lineage assignment is maximized when both sequencing depth and S gene completeness are above the mean values for correct classification (top right quadrant).

By applying the Fast-S method, incorrect lineage set assignments were related to AY.* (*n*=4), B.1.1.7 (*n*=2), B.1.351 (*n*=2), B.1.617.2 (*n*=1), B.1.621 (*n*=4), BA.1 (*n*=2), P.6 (*n*=3) and C.37 (*n*=1) samples. As with the Standard-S protocol, misassigned samples had a lower S gene completeness (73.6±14.3 %), with exception of B.1.621 samples (96.1±5.5 %) (Fig. 2a). Significant differences were observed in terms of average sequencing depth and S gene completeness between correct and incorrect classifications. The correctly classified samples had a mean gene completeness of 94 ±8% and a mean average sequencing depth of 407±90×. The incorrect classifications had a mean gene completeness of 78±15 % and a mean sequencing depth of 298±122× (Fig. 2b). After S gene read sampling and reassignment of each resulting consensus sequence (Figs S3 and S4), significant differences were observed in terms of average sequencing depth and S gene completeness (*P*≤0.001). Incorrect assignments were associated with average sequencing depth lower than 178× and a mean S gene completeness below 64 % (Fig. 2c). A combination of an average sequencing depth above 277±92× and a S gene completeness of 80±13 % was required to maximize correct lineage assignments (82 % of correct assignments) (Fig. 2d).

### S-based classification robustness is maintained within Omicron sublineages

**Fig. 3.**
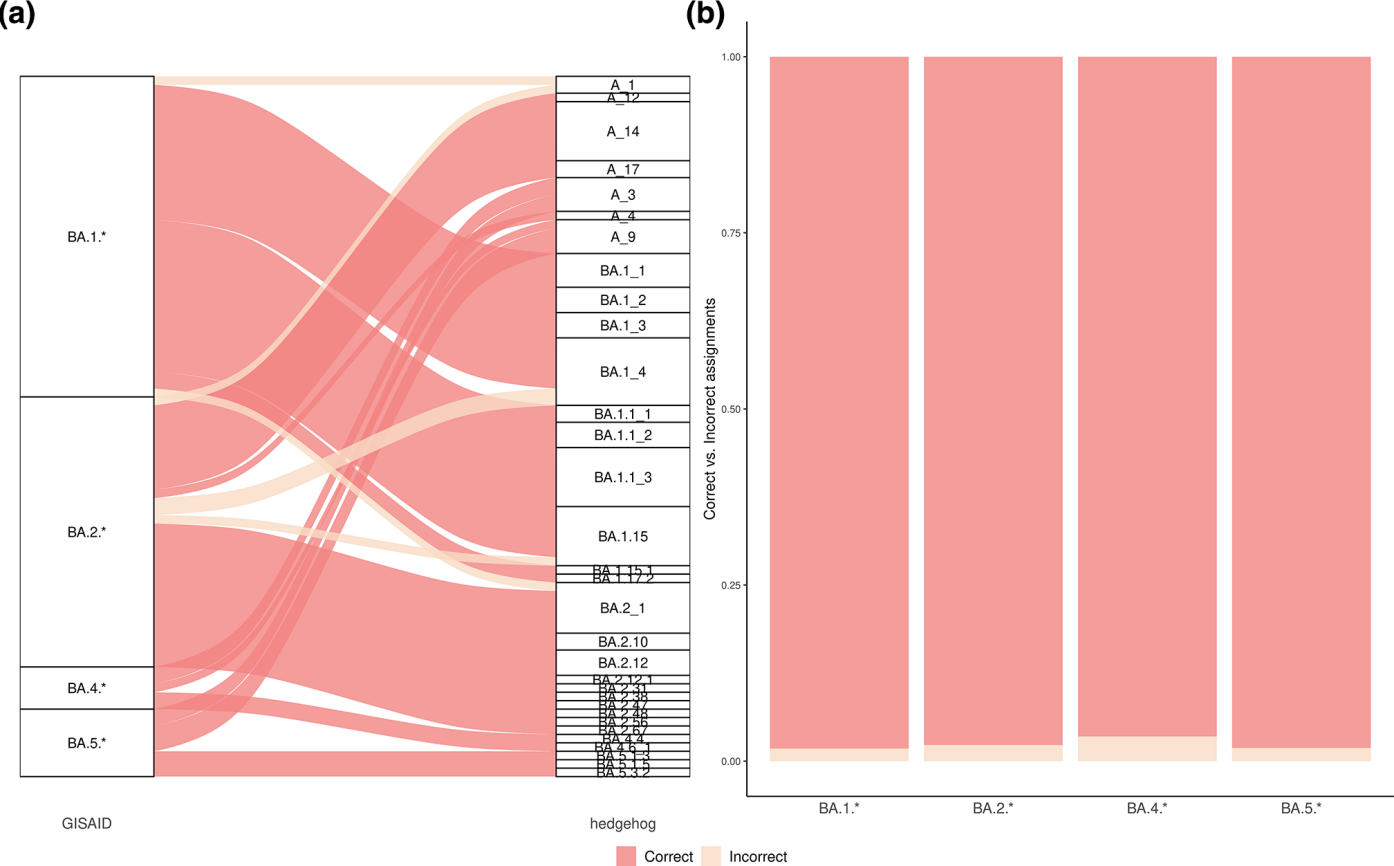
Genome-based lineage assignment compared to spike gene-based assignment of the Omicron dataset retrieved from the GISAID/EpiCoV database. (**a**) WGS- and S gene-based lineage assignment comparison of a subset of 300 sequences from the Omicron dataset. (**b**) Correct and incorrect lineage assignments from the Omicron dataset: correct assignments were obtained for 98.2 % of BA.1.* (*n*=2643), 97.7 % of BA.2.* (*n*=2096), 96.5 % of BA.4.* (*n*=57) and 98.1 % of BA.5.* (*n*=161) sequences.

To further evaluate the suitability of the S gene-based lineage assignment, we evaluated a dataset containing 5000 Omicron sublineages retrieved from EpiCov/GISAID database (Fig. S5). The hedgehog tool was used to assign the lineage set from the extracted S gene sequences from the dataset. Comparisons were made at Omicron sublineage level BA.1.*, BA.2.*, etc., for genome and S gene sequences, resulting in ~98 % of correct lineage set assignments (Fig. 3a). Overall, 2587 out of 2634 BA.1.* extracted S gene sequences from the Omicron dataset (98.2 %) were correctly assigned when compared to the genome-based classification (BA.1.*): 2048 out of 2096 (97.7 %) for BA.2.* lineages, 55 out of 57 (96.5 %) for BA.4.* lineages and 158 out 161 (98.1 %) for BA.5.* (Fig. 3b). A detailed description of the SARS-CoV-2 genomes and their spike-only sequence lineage assignments for each sublineage can be found in Fig. S6 and Table S5. Additionally, a significant difference between correct and incorrect assignments were observed in terms of gene completeness among the extracted S gene sequences from the Omicron dataset (Fig. S7). Correct assignments had an S gene completeness of 97.2±8.6% and incorrect assignments of 94.3±5.1 %. This suggests that high levels of S gene completeness are required to achieve a precise lineage set assignment within Omicron sublineages.

### Proposal of a new SARS-CoV-2 genomic surveillance workflow

Since the beginning of the global SARS-CoV-2 pandemic, genomic sequences have been shared mostly through the EpiCoV/GISAID database. These samples were collected from clinical specimens, but also from environmental and non-human hosts. Many countries such as the USA and the UK have been supporting genomic surveillance programmes in order to characterize the circulating SARS-CoV-2 variants [30, 31]. WHO has endorsed SARS-CoV-2 genomic surveillance as a priority [32]. However, its implementation has remained limited especially in low- and middle-income countries (LMICs) due to infrastructure requirements, higher reagent costs and stakeholder engagement. This aspect is reflected in the distribution of genome representation across different continents (Fig. S8).

**Fig. 4.**
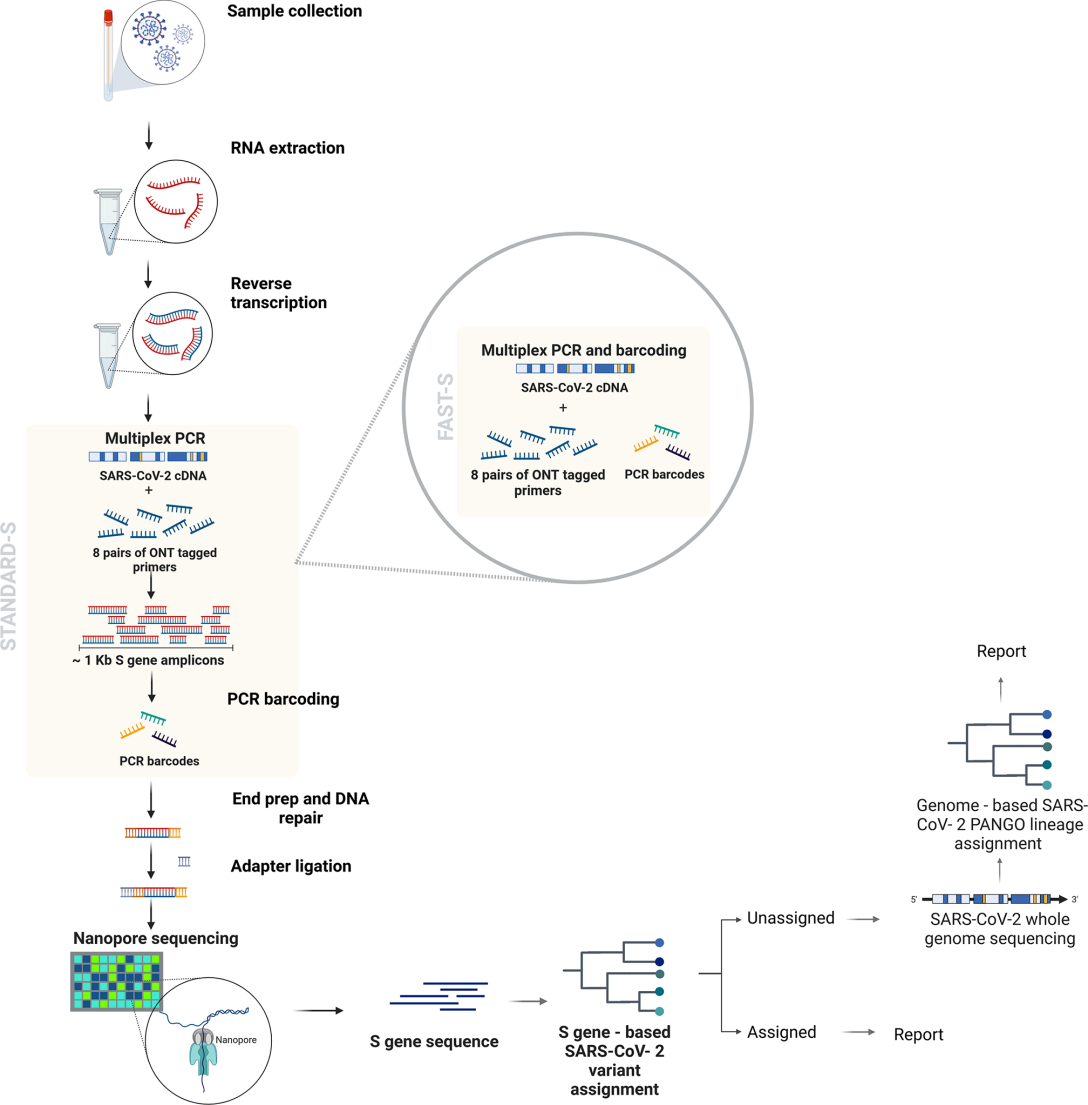
SARS-CoV-2 screening workflow proposal. RNA is extracted from SARS-CoV-2-positive samples and reverse transcribed. After single tube amplification and PCR barcoding, pooled samples are cleaned and end-prepped. The sequencing adapter is ligated and the final library is run in an ONT flow cell. S gene consensus sequences are generated using epi2me-lab/wf-artic using the V1 scheme. The PANGO lineage set is determined and reported. Samples unassigned and/or displaying unusual changes in the S gene sequence are submitted to WGS. Figure created with BioRender.com.

Here, we present a workflow that is intended to assist in decision-making in the context of SARS-CoV-2 genomic surveillance efforts, which represents a middle ground option between a qPCR assay and WGS. In this workflow, SARS-CoV-2 residual RNA from positive samples with pre-specified backgrounds (i.e. closed community outbreaks, breakthrough infections) are referred to the sequencing facility and subjected to reverse transcription. The samples are screened for variant detection where the S gene is amplified using the ARTIC Network primers that cover the full S gene sequence [33] and an ONT PCR barcoded library is prepared using the Standard-S or Fast-S protocol. After the sequencing adapter is ligated, the library is then loaded into an Oxford Nanopore flow cell and sequenced until a suitable S gene completeness is achieved. Consensus sequences for the S gene of the samples are generated and lineage is determined using hedgehog, a spike gene specific tool for lineage assignment. If lineage detection is achieved, results are reported. If a reasonable S gene completeness is reached and no lineage is assigned or relevant changes in the consensus sequence are observed, samples are submitted for WGS for further characterization (Fig. 4). These steps have the potential to optimize the variant surveillance workflow, delivering informative results of SARS-CoV-2 variant circulation at a lower cost. An estimation of the costs of the Fast-S protocol starts at USD 6, in contrast to USD 11 for WGS (Table 1). Estimation of the complete time of execution for both the Standard-S and Fast-S protocol is less than 20 h, whereas for WGS it is between 24 and 48 h (Table S6). A breakdown of reagent and material costs are included in Tables S7–S10.

**Table 1.** Costs and time for the S gene sequencing protocol and WGS of SARS-CoV-2

Strategy	Protocol	Cost per sample (USD)	Total time for library preparation (h)	Sequencing and analysis (h)
SARS-CoV-2 WGS	Midnight protocol	11–15	5	24–48
Target SARS-CoV-2 S gene	Standard-S	8–10	6	13–20
Target SARS-CoV-2 S gene	Fast-S	6–9	5	13–20

Variations in costs and reporting time are expected in different settings.

## Discussion

The S protein is critical for the function of SARS-CoV-2 and despite the development and deployment of vaccines, the persistence of the virus is driven by the emergence of hypermutated and increasingly transmissible variants. These variants are likely to evade current vaccines, mostly through mutations in the spike protein, more specifically in the receptor binding domain (RBD) [34 ]. As we move to an endemic phase in the COVID-19 pandemic, alternative strategies may be necessary for more rational and cost-effective variant surveillance. To aid the decisions around SARS-CoV-2 genomic sequencing, we propose a workflow that is based on the development of a fast variant screening protocol of the S gene (Fast-S), for the portable ONT sequencing platform.

Many strategies of S gene-based variant screening have emerged during the pandemic. The Illumina-based S gene tiling sequencing approach for the ectodomain of the SARS-CoV-2 S gene was used for 10–50 % of all COVID-19 cases in Austria from January to June 2021 [16]. Also, the HiSpike method was developed, using a three-step protocol with a turnaround time of ~30 h consisting of RT-PCR1 and PCR2, and a single cleanup step of the pooled MiSeq library [35]. Several other methods have been developed based on Sanger sequencing [36–42] and also for the Oxford Nanopore platform [43]. However, the steps in both the Standard-S and Fast-S workflow have the potential to enable S gene mutational surveillance with minimal requirements for a small group of samples per run (12–96) up to thousands of samples when in combination with dual amplicon indexing protocols, using a combination of ONT native barcodes [44] or costume barcodes [43]. Using the Fast-S protocol presented in this study, the amplification and barcoding steps can be performed in a single tube, minimizing the possibility of cross-contamination during amplicon handling between the amplification and barcoding steps. Since the protocol is based on a tiled amplification approach using a multiplex PCR, particular attention to the amplification performance of these primers is highly recommended in order to achieve a reasonable sequencing depth and S gene completeness to maximize PANGO lineage set assignments.

One of the key aspects of pathogen variant surveillance is the generation of reliable data and scalable procedures, while minimizing costs. The development of S gene-specific analysis tools that take into consideration the limitations of the lineage assignments from subgenomic sequences is of great importance. Hedgehog software considers information present only in the S gene to avoid conflicting or missing results, as opposed to pangolin [11], which is trained on a data set of genomes that have been designated to PANGO lineages using whole genome information. Moreover, hedgehog introduced the concept of a ‘lineage set’ to represent the range of PANGO lineages that are compatible with a given spike gene sequence [14]. The development of S gene sequencing protocols, as described in the present study, along with the development and refinement of dedicated bioinformatic tools have the potential to assist SARS-CoV-2 surveillance.

During the COVID-19 pandemic, timely public health responses were made mainly by tracking SARS-CoV-2 genetic diversity in real time. Consequently, a massive wave of genomic data were generated enabling global surveillance of the novel coronavirus. High-income regions such as Europe and North America led the upload of SARS-CoV-2 genomic data to EpiCoV/GISAID, accounting for 6.6 million and 4.4 million sequences as of August 2022, respectively. Other regions (South America, Asia, and Africa and Oceania) contributed collectively with 1.6 million sequences, highlighting the disparity of genomic sequencing efforts around the globe (Fig. S8). Although sequencing costs have decreased and portable platforms alleviate the need for dedicated infrastructure, genomic sequencing is still challenging in developing countries in terms of costs, logistics and skilled workforce [45]. Additionally, GDP, investment in research and development, and national coordinated sequencing efforts impact the ability of countries to perform real-time genomic surveillance [46]. In LMICs, most of the sequencing efforts are generated in academic institutions, which have performed little genomic surveillance with a slower turnaround time for data sharing through open-access databases. Even though the importance of data sharing is well recognized in these countries and the information is mostly shared with Public Health authorities, concerns have been raised that the efforts will benefit institutions with greater analysis capabilities, resulting in top-tier journal publications, grants and patents, from which LMICs do not benefit [47].

## Conclusions

Using the spike S gene sequencing protocols described in this proof-of-concept, a variant overview of up to 96 samples can be obtained at a starting cost of ~USD 6 per sample. The costs have the potential to be dramatically decreased when in combination with different multiplexing strategies. The turnaround time for generating a sample report is lower than for WGS, since the same number of samples require longer sequencing runs to achieve a suitable coverage for consensus generation. Samples that cannot be assigned using these methods or display unusual S gene sequence modifications are candidates for WGS for further characterization to confirm/discard the observed changes.

The S gene sequences obtained either with the Standard-S or Fast-S protocol are effective in discriminating samples at the variant level. Moreover, detection of the exact PANGO lineages at the sublineage level is still limited and regular updates in the primer scheme used for S gene amplification are likely to be required. However, samples with a reasonable sequencing depth and S gene completeness can be assigned to different lineages within known VOCs. Since the sequencing depth and gene completeness may not be good predictors of correct assignments in all cases, it is highly recommended to add positive controls in the sequencing run for validation of the results. Both the Standard-S and Fast-S protocols could be useful tools for high-throughput screening of SARS-CoV-2 samples. Additionally, one advantage of the Fast-S from other procedures is that it decreases the chances of cross-contamination between samples, as the tubes remain closed until the barcoding step is concluded. The aim of this work is to generate informative SARS-CoV-2 variant reports using cost-effective protocols, and the workflow presented here could also be useful to massive screening of other pathogens of interest.

## Supplementary Data

Supplementary material 1

Supplementary material 2
