# Supplementary material for: Fast and cost-effective SARS-CoV-2 variant detection using Oxford Nanopore full-length spike gene sequencing
Source: Microb Genom. 2023 May 18;9(5):mgen001013. doi: 10.1099/mgen.0.001013 (PMC10272875; doi:10.1099/mgen.0.001013)
Supplement: Supplementary material 1 [file mgen-9-1013-s001.pdf]

Supplementary material

Fast and cost-effective SARS-CoV-2 variant detection using Oxford Nanopore full-length spike gene sequencing

Cecilia Salazar[1,2,*], Ferrés Ignacio[1,2], Mercedes Paz[2,3,4], Alicia Costábile[2,,3,4,5], Gonzalo Moratorio[2,3,4], Pilar Moreno[2,3,4], Gregorio Iraola[1,2,6,7,*]

1 Laboratorio de Genómica Microbiana. Institut Pasteur de Montevideo. Uruguay

2 Centro de Innovación en Vigilancia Epidemiológica, Institut Pasteur de Montevideo. Uruguay

3 Laboratorio de Evolución Experimental de Virus, Institut Pasteur de Montevideo. Uruguay

4 Laboratorio de Virología Molecular, Facultad de Ciencias, Universidad de la República. Uruguay

5 Sección Bioquímica, Facultad de Ciencias, Universidad de la República. Uruguay

6 Wellcome Sanger Institute, Hinxton. United Kingdom

7 Centro de Biología Integrativa, Universidad Mayor. Chile

* Correspondence: Cecilia Salazar, Microbial Genomics Laboratory, Institut Pasteur de Montevideo, Montevideo 11400, Uruguay. Email: csalazar@pasteur.edu.uy; Gregorio Iraola, Microbial Genomics Laboratory, Institut Pasteur de Montevideo, Montevideo 11400, Uruguay. Email: giraola@pasteur.edu.uy.

Table S1: SARS-CoV-2 positive sample metadata available

Sample	Diagnostic Ct value	Location	Diagnostic date	Collection date
CUY1-000006	18.0	Artigas	2021-03-04	2021-03-04
CUY12-002713	15.3	Montevideo	2021-06-07	2021-06-06
CUY12-002716	16.8	Montevideo	2021-06-07	2021-06-06
CUY12-002721	19.9	Montevideo	2021-06-07	2021-06-05
CUY12-002725	15.9	Montevideo	2021-06-07	2021-06-05
CUY16-003496	16.3	Montevideo	2021-02-18	MD
CUY16-003498	15.8	Montevideo	2021-02-18	MD
CUY16-003501	16.5	Montevideo	2021-02-17	MD
CUY16-003536	19.6	Montevideo	2021-01-29	MD
CUY16-003538	17.7	Montevideo	2021-01-28	MD
CUY16-003792	15.3	Montevideo	2021-07-07	2021-07-05
CUY16-003800	16.7	Montevideo	2021-07-07	2021-07-06
CUY16-003801	17.7	Montevideo	2021-07-07	2021-07-06
CUY16-003802	13.5	Montevideo	2021-07-07	2021-07-06
CUY16-003803	15.2	Montevideo	2021-07-07	2021-07-06
CUY16-003804	14.5	Montevideo	2021-07-07	2021-07-06
CUY17-003849	21.0	Canelones	2021-07-07	2021-07-07
CUY17-003866	25.8	MD	2021-07-12	MD
CUY17-003867	27.9	MD	2021-07-12	MD
CUY17-003892	15.7	Montevideo	2021-07-14	MD
CUY17-003893	15.7	Montevideo	2021-07-14	MD
CUY17-003901	10.0	Montevideo	2021-07-09	2021-07-09
CUY17-003909	18.4	Montevideo	2021-07-14	2021-07-12
CUY17-003910	18.6	Montevideo	2021-07-14	2021-07-14
CUY17-003939	14.8	Montevideo	2021-07-14	2021-07-13
CUY18-004019	22.2	MD	2021-07-19	MD
CUY18-004022	20.2	MD	2021-07-19	MD
CUY18-004023	20.8	MD	2021-07-19	MD
CUY18-004026	22.3	MD	2021-07-16	MD
CUY19-004183	20.2	MD	2021-07-18	MD
CUY21-004441	15.8	MD	2021-08-01	MD
CUY23-004816	19.0	Montevideo	2021-08-22	2021-08-22
CUY24-004876	15.0	Montevideo	2021-08-23	MD
CUY24-004954	15.3	Montevideo	2021-08-30	MD
CUY26-005235	17.9	Montevideo	MD	2021-09-06
CUY4-000486	1.0	Montevideo	MD	2021-04-03
CUY42-007286	19.0	Canelones	2022-01-04	2022-01-04
CUY42-007288	19.0	Canelones	2022-01-04	2022-01-04
CUY42-007289	20.0	Montevideo	2022-01-04	2022-01-04
CUY42-007291	22.0	San José	2022-01-04	2022-01-04
CUY42-007294	18.0	San José	2022-01-04	2022-01-04
CUY5-000547	17.0	Montevideo	MD	2021-04-10
CUY6-001233	23.2	Canelones	2021-04-19	2021-04-18
CUY8-001646	19.4	Canelones	2021-04-17	2021-04-17

MD: missing data

Table S2: Pool A (Odd) and pool B (pair) ARTIC Network primers for S gene amplification

Primer	ONT TAG - primers (5’ -> 3’)
ONT_Sseq_1_LEFT	**TTTCTGTTGGTGCTGATATTGC** ACAAAAGAAAATGACTCTAAAGAGGGTTT
ONT_Sseq_1_RIGHT	**ACTTGCCTGTCGCTCTATCTTC** ACTCTGAACTCACTTTCCATCCAAC
ONT_Sseq_3_LEFT	**TTTCTGTTGGTGCTGATATTGC** AGAGTCCAACCAACAGAATCTATTGT
ONT_Sseq_3_RIGHT	**ACTTGCCTGTCGCTCTATCTTC** ACCTGTGCCTGTTAAACCATTGA
ONT_Sseq_5_LEFT	**TTTCTGTTGGTGCTGATATTGC** CAACTTACTCCTACTTGGCGTGT
ONT_Sseq_5_RIGHT	**ACTTGCCTGTCGCTCTATCTTC** TGGAGCTAAGTTGTTTAACAAGCG
ONT_Sseq_7_LEFT	**TTTCTGTTGGTGCTGATATTGC** GGGCTATCATCTTATGTCCTTCCCT
ONT_Sseq_7_RIGHT	**ACTTGCCTGTCGCTCTATCTTC** AGGTGTGAGTAAACTGTTACAAACAAC
ONT_Sseq_2_LEFT	**TTTCTGTTGGTGCTGATATTGC** ACACGTGGTGTTTATTACCCTGAC
ONT_Sseq_2_RIGHT	**ACTTGCCTGTCGCTCTATCTTC** GCAACACAGTTGCTGATTCTCTTC
ONT_Sseq_4_LEFT	**TTTCTGTTGGTGCTGATATTGC** CCAGCAACTGTTTGTGGACCTA
ONT_Sseq_4_RIGHT	**ACTTGCCTGTCGCTCTATCTTC** TGTGTACAAAAACTGCCATATTGCA
ONT_Sseq_6_LEFT	**TTTCTGTTGGTGCTGATATTGC** TTGCCTTGGTGATATTGCTGCT
ONT_Sseq_6_RIGHT	**ACTTGCCTGTCGCTCTATCTTC** TGCCAGAGATGTCACCTAAATCAA
ONT_Sseq_8_LEFT	**TTTCTGTTGGTGCTGATATTGC** TGCTGTAGTTGTCTCAAGGGCT
ONT_Sseq_8_RIGHT	**ACTTGCCTGTCGCTCTATCTTC** ACGAAAGCAAGAAAAAGAAGTACGC

Table S3: Results of S gene sequencing using the Standard-S and Fast-S amplicon sequencing compared to WGS

		Genome			Standard-S						Fast-S					
**Sample**	**biosample**	**WGS reads**	**Nextclade**	**PANGO lineage**	**Barcode**	**Std-S reads**	**Depth**	**Completeness**	**hedgehog**	**set_description**	**Barcode**	**Fast-S reads**	**Depth**	**Completeness**	**hedgehog**	**set_description**
**CUY1-000006**	SAMN31488805	79.526	20B	P.2	barcode83	318.857	534,8	98,97	P.2	P.2	barcode81	30.004	384,3	98,97	P.2	P.2
**CUY12-002713**	SAMN31488806	55.916	20J (Gamma, V3)	P.1	barcode45	96.014	534,5	98,97	P.1	P.1_1	barcode43	92.921	487,6	98,95	P.1	P.1_1
**CUY12-002716**	SAMN31488807	42.759	20J (Gamma, V3)	P.1	barcode57	86.224	533,9	98,97	P.1	P.1_1	barcode55	101.068	485,9	98,95	P.1	P.1_1
**CUY12-002721**	SAMN31488808	51.674	20J (Gamma, V3)	P.1	barcode69	5.405	428,8	98,93	P.1	P.1_1	barcode67	6.631	292,0	87,88	P.1	P.1_1
**CUY12-002725**	SAMN31488809	50.346	20J (Gamma, V3)	P.1	barcode81	77.297	533,8	98,95	P.1	P.1_1	barcode79	142.683	532,8	98,95	P.1	P.1_1
**CUY16-003496**	SAMN31488810	10.523	20B	P.2	barcode71	62.622	531,0	98,97	P.2	P.2	barcode69	7.533	327,0	71,53	P.2	P.2
**CUY16-003498**	SAMN31488811	14.866	20B	P.6	barcode36	24.253	499,3	98,97	P.6	P.6	barcode34	2.132	156,6	66,68	A	A_1
**CUY16-003501**	SAMN31488812	17.526	20B	P.6	barcode48	48.057	532,1	98,97	P.6	P.6	barcode46	33.906	366,2	98,97	P.6	P.6
**CUY16-003536**	SAMN31488813	17.579	20B	P.6	barcode60	25.024	530,5	98,97	P.6	P.6	barcode58	3.970	219,8	68,32	A	A_1
**CUY16-003538**	SAMN31488814	23.725	20B	P.6	barcode72	5.522	336,8	98,97	P.6	P.6	barcode70	6.314	211,4	71,41	A	A_1
**CUY16-003792**	SAMN31488815	12.612	21A (Delta)	B.1.617.2	barcode71	88.338	339,4	71,72	AY.30	AY.30	barcode62	5.074	457,3	88,19	B.1.617.2	B.1.617.2_7
**CUY16-003800**	SAMN31488816	14.559	20H (Beta, V2)	B.1.351	barcode68	146	15,3	18,59	A	A_23	barcode66	170.148	512,7	98,80	B.1.351	B.1.351
**CUY16-003801**	SAMN31488817	13.649	20H (Beta, V2)	B.1.351	barcode92	37.334	519,0	98,80	B.1.351	B.1.351	barcode90	33.130	284,9	87,71	A	A_1
**CUY16-003802**	SAMN31488818	22.218	20H (Beta, V2)	B.1.351	barcode80	348.485	533,7	98,80	B.1.351	B.1.351	barcode78	185.166	527,8	98,78	B.1.351	B.1.351
**CUY16-003803**	SAMN31488819	12.872	20H (Beta, V2)	B.1.351	barcode21	132.790	533,5	98,80	B.1.351	B.1.351	barcode19	50.019	430,0	98,80	B.1.351	B.1.351
**CUY16-003804**	SAMN31488820	15.395	20H (Beta, V2)	B.1.351	barcode09	4.258	262,8	98,80	B.1.351	B.1.351	barcode07	1.734	111,1	43,02	A	A_23
**CUY17-003849**	SAMN31488821	37.374	20I (Alpha, V1)	B.1.1.7	barcode08	147.783	532,5	98,78	B.1.1.7	B.1.1.7_1	barcode06	14.087	461,2	98,78	B.1.1.7	B.1.1.7_1
**CUY17-003866**	SAMN31488822	13.967	20I (Alpha, V1)	B.1.1.7	barcode20	676	33,8	42,84	A	A_1	barcode18	31.301	174,2	71,49	A	A_1
**CUY17-003867**	SAMN31488823	11.691	20I (Alpha, V1)	B.1.1.7	barcode32	151	4,2	0,00	Unassigned	Unassigned	barcode30	48.735	313,5	71,49	A	A_1
**CUY17-003892**	SAMN31488824	38.353	21I (Delta)	AY.26	barcode95	116.610	367,3	83,72	B.1.617.2	B.1.617.2_7	barcode08	145.978	449,5	88,30	B.1.617.2	B.1.617.2_7
**CUY17-003893**	SAMN31488825	34.808	21A (Delta)	B.1.617.2	barcode59	7.707	330,7	83,85	AY.48	AY.48	barcode20	278.482	471,9	88,28	AY.48	AY.48
**CUY17-003901**	SAMN31488826	13.986	21J (Delta)	AY.122	barcode22	30.000	486,6	88,26	B.1.617.2	B.1.617.2_2	barcode03	16.000	471,0	88,21	B.1.617.2	B.1.617.2_2
**CUY17-003909**	SAMN31488827	12.572	21A (Delta)	B.1.617.2	barcode34	3.000	11,4	14,83	A	A_1	barcode15	7.500	210,4	83,15	B.1.617.2	B.1.617.2_2
**CUY17-003910**	SAMN31488828	14.056	21J (Delta)	AY.122	barcode58	7.500	339,7	82,08	A	A_11	barcode39	10.000	346,1	87,40	B.1.617.2	B.1.617.2_2
**CUY17-003939**	SAMN31488829	50.819	20J (Gamma, V3)	P.1	barcode33	7.707	488,2	98,93	P.1	P.1_1	barcode31	29.566	418,0	98,91	P.1	P.1_1
**CUY18-004019**	SAMN31488830	10.835	21H (Mu)	B.1.621	barcode58	37.398	512,4	98,95	B.1.621	B.1.621	barcode56	33.026	387,4	98,93	AY.33.2	AY.33.2
**CUY18-004022**	SAMN31488831	13.124	21H (Mu)	B.1.621	barcode70	77.392	532,1	98,93	AY.33.2	AY.33.2	barcode68	28.136	251,3	87,82	AY.33.2	AY.33.2
**CUY18-004023**	SAMN31488832	16.371	21H (Mu)	B.1.621	barcode82	25.329	496,9	98,91	AY.33.2	AY.33.2	barcode80	69.169	417,5	98,89	B.1.617.2	B.1.617.2_2
**CUY18-004026**	SAMN31488833	4.898	21H (Mu)	B.1.621.1	barcode93	6.056	479,9	98,89	B.1.621.1	B.1.621.1	barcode54	133.724	410,7	98,82	AY.30	AY.30
**CUY19-004183**	SAMN31488834	73.438	21G (Lambda)	C.37	barcode35	16.120	527,9	98,57	C.37	C.37	barcode33	4.968	295,6	71,53	C.37	C.37
**CUY21-004441**	SAMN31488835	29.349	21H (Mu)	B.1.621	[redacted]						barcode51	17.000	523,3	98,95	B.1.621	B.1.621
**CUY23-004816**	SAMN31488836	22.640	21J (Delta)	AY.20	barcode47	76.909	358,5	83,85	AY.20	AY.20	barcode32	56.577	418,7	87,82	AY.20	AY.20
**CUY24-004876**	SAMN31488837	31.373	21J (Delta)	AY.25.1	barcode46	21.000	371,5	88,24	A	A_11	barcode27	7.000	395,0	88,17	B.1.617.2	B.1.617.2_4
**CUY24-004954**	SAMN31488838	23.594	21J (Delta)	AY.99.2	barcode46	194.573	468,2	88,28	A	A_11	barcode44	74.859	414,8	87,79	A	A_11
**CUY26-005235**	SAMN31488839	17.810	21I (Delta)	B.1.617.2	barcode82	500	9,2	7,21	A	A_2	barcode63	15.000	363,5	83,32	B.1.617.2	B.1.617.2_3
**CUY4-000486**	SAMN31488840	22.255	20B	P.2	barcode12	106.885	534,3	98,97	P.2	P.2	barcode10	79.571	451,7	98,97	P.2	P.2
**CUY42-007286**	SAMN31488841	73.308	21K (Omicron)	BA.1	barcode36	6.500	157,9	73,26	A	A_1	barcode01	3.500	103,6	55,61	BA.2.12	BA.2.12
**CUY42-007288**	SAMN31488842	55.059	21K (Omicron)	BA.1	barcode48	8.000	223,7	75,44	BA.1.15	BA.1.15	barcode13	6.500	296,7	98,59	BA.1.15	BA.1.15
**CUY42-007289**	SAMN31488843	67.186	21K (Omicron)	BA.1	barcode60	15.500	329,6	84,96	BA.1.15	BA.1.15	barcode25	5.000	248,4	98,74	BA.1	BA.1_2
**CUY42-007291**	SAMN31488844	31.682	21K (Omicron)	BA.1	barcode72	3.500	68,5	49,16	A	A_1	barcode37	4.500	189,9	57,21	BA.2.12	BA.2.12
**CUY42-007294**	SAMN31488845	90.587	21K (Omicron)	BA.1.1	barcode84	9.500	276,1	84,96	BA.1	BA.1_4	barcode49	8.000	370,7	98,80	BA.1.1	BA.1.1_2
**CUY5-000547**	SAMN31488846	65.494	20B	P.6	barcode24	59.987	533,9	98,97	P.6	P.6	barcode22	95.108	476,4	98,97	P.6	P.6
**CUY6-001233**	SAMN31488847	19.595	21G (Lambda)	C.37.1	barcode47	36.472	530,8	98,57	C.37.1	C.37.1	barcode45	5.607	300,2	71,49	A	A_5
**CUY8-001646**	SAMN31488848	31.632	21G (Lambda)	C.37.1	barcode59	326.563	533,7	98,65	C.37.1	C.37.1	barcode57	46.296	424,9	98,55	C.37.1	C.37.1

Table S4: PANGO lineage range lineages obtained with Hedgehog

Sample	Method	hedgehog_lineage	set_description	**lineages** (obtained from https://github.com/cov-lineages/hedgehog/blob/main/hedgehog/data/)
CUY1-000006	Standard-S	P.2	P.2	P.2
CUY4-000486	Standard-S	P.2	P.2	P.2
CUY5-000547	Standard-S	P.6	P.6	P.6
CUY6-001233	Standard-S	C.37.1	C.37.1	C.37.1
CUY8-001646	Standard-S	C.37.1	C.37.1	C.37.1
CUY12-002713	Standard-S	P.1	P.1_1	P.1|P.1.1|P.1.10|P.1.10.1|P.1.12|P.1.13|P.1.15|P.1.16|P.1.2
CUY12-002716	Standard-S	P.1	P.1_1	P.1|P.1.1|P.1.10|P.1.10.1|P.1.12|P.1.13|P.1.15|P.1.16|P.1.2
CUY12-002721	Standard-S	P.1	P.1_1	P.1|P.1.1|P.1.10|P.1.10.1|P.1.12|P.1.13|P.1.15|P.1.16|P.1.2
CUY12-002725	Standard-S	P.1	P.1_1	P.1|P.1.1|P.1.10|P.1.10.1|P.1.12|P.1.13|P.1.15|P.1.16|P.1.2
CUY16-003496	Standard-S	P.2	P.2	P.2
CUY16-003498	Standard-S	P.6	P.6	P.6
CUY16-003501	Standard-S	P.6	P.6	P.6
CUY16-003536	Standard-S	P.6	P.6	P.6
CUY16-003538	Standard-S	P.6	P.6	P.6
CUY16-003792	Standard-S	AY.30	AY.30	AY.30
CUY16-003800	Standard-S	A	A_23	B.1.1.345|B.1.1.393|B.1.400.1|N.9
CUY16-003801	Standard-S	B.1.351	B.1.351	B.1.351
CUY16-003802	Standard-S	B.1.351	B.1.351	B.1.351
CUY16-003803	Standard-S	B.1.351	B.1.351	B.1.351
CUY16-003804	Standard-S	B.1.351	B.1.351	B.1.351
CUY17-003849	Standard-S	B.1.1.7	B.1.1.7_1	B.1.1.7|Q.1|Q.3|Q.7
CUY17-003866	Standard-S	A	A_1	A.18|AE.2|AE.4|AE.7|AE.8|AF.1|AG.1|AN.1|AP.1|AQ.1|B.1|B.1.1|B.1.1.10|B.1.1.101|B.1.1.110|B.1.1.111|B.1.1.113|B.1.1.12|B.1.1.121|B.1.1.127|B.1.1.128|B.1.1.129|B.1.1.132|B.1.1.133|B.1.1.134|B.1.1.135|B.1.1.139|B.1.1.148|B.1.1.152|B.1.1.157|B.1.1.158|B.1.1.159|B.1.1.162|B.1.1.163|B.1.1.164|B.1.1.166|B.1.1.169|B.1.1.171|B.1.1.172|B.1.1.174|B.1.1.176|B.1.1.177|B.1.1.181|B.1.1.182|B.1.1.184|B.1.1.185|B.1.1.187|B.1.1.189|B.1.1.192|B.1.1.198|B.1.1.200|B.1.1.201|B.1.1.204|B.1.1.205|B.1.1.209|B.1.1.210|B.1.1.214|B.1.1.216|B.1.1.217|B.1.1.218|B.1.1.219|B.1.1.225|B.1.1.226|B.1.1.229|B.1.1.230|B.1.1.231|B.1.1.232|B.1.1.236|B.1.1.243|B.1.1.244|B.1.1.25|B.1.1.253|B.1.1.254|B.1.1.257|B.1.1.258|B.1.1.26|B.1.1.261|B.1.1.265|B.1.1.266|B.1.1.267|B.1.1.268|B.1.1.27|B.1.1.271|B.1.1.274|B.1.1.277|B.1.1.282|B.1.1.283|B.1.1.284|B.1.1.285|B.1.1.286|B.1.1.288|B.1.1.290|B.1.1.291|B.1.1.297|B.1.1.301|B.1.1.304|B.1.1.305|B.1.1.306|B.1.1.307|B.1.1.308|B.1.1.31|B.1.1.315|B.1.1.317|B.1.1.319|B.1.1.323|B.1.1.324|B.1.1.325|B.1.1.326|B.1.1.328|B.1.1.329|B.1.1.33|B.1.1.331|B.1.1.334|B.1.1.335|B.1.1.34|B.1.1.342|B.1.1.344|B.1.1.346|B.1.1.347|B.1.1.348|B.1.1.350|B.1.1.352|B.1.1.354|B.1.1.355|B.1.1.356|B.1.1.357|B.1.1.359|B.1.1.366|B.1.1.367|B.1.1.368|B.1.1.370|B.1.1.373|B.1.1.376|B.1.1.378|B.1.1.381|B.1.1.382|B.1.1.383|B.1.1.386|B.1.1.387|B.1.1.388|B.1.1.39|B.1.1.391|B.1.1.392|B.1.1.398|B.1.1.40|B.1.1.401|B.1.1.402|B.1.1.404|B.1.1.405|B.1.1.406|B.1.1.407|B.1.1.409|B.1.1.410|B.1.1.411|B.1.1.414|B.1.1.416|B.1.1.419|B.1.1.422|B.1.1.424|B.1.1.426|B.1.1.427|B.1.1.430|B.1.1.431|B.1.1.432|B.1.1.434|B.1.1.435|B.1.1.436|B.1.1.437|B.1.1.438|B.1.1.44|B.1.1.440|B.1.1.441|B.1.1.442|B.1.1.447|B.1.1.448|B.1.1.450|B.1.1.452|B.1.1.453|B.1.1.456|B.1.1.458|B.1.1.459|B.1.1.46|B.1.1.465|B.1.1.466|B.1.1.47|B.1.1.48|B.1.1.481|B.1.1.482|B.1.1.484|B.1.1.485|B.1.1.487|B.1.1.5|B.1.1.50|B.1.1.506|B.1.1.507|B.1.1.513|B.1.1.514|B.1.1.515|B.1.1.516|B.1.1.518|B.1.1.52|B.1.1.53|B.1.1.54|B.1.1.57|B.1.1.61|B.1.1.62|B.1.1.67|B.1.1.70|B.1.1.71|B.1.1.77|B.1.1.8|B.1.1.87|B.1.1.88|B.1.1.89|B.1.1.98|B.1.1.99|B.1.103|B.1.104|B.1.106|B.1.108|B.1.110|B.1.110.1|B.1.110.3|B.1.111|B.1.112|B.1.113|B.1.115|B.1.116|B.1.118|B.1.119|B.1.12|B.1.124|B.1.13|B.1.131|B.1.134|B.1.137|B.1.139|B.1.142|B.1.143|B.1.145|B.1.146|B.1.147|B.1.149|B.1.151|B.1.153|B.1.157|B.1.160.32|B.1.161|B.1.162|B.1.163|B.1.164|B.1.166|B.1.167|B.1.169|B.1.170|B.1.177.31|B.1.178|B.1.179|B.1.180|B.1.181|B.1.182|B.1.184|B.1.187|B.1.188|B.1.189|B.1.190|B.1.192|B.1.194|B.1.195|B.1.199|B.1.2|B.1.201|B.1.203|B.1.206|B.1.208|B.1.210|B.1.211|B.1.212|B.1.213|B.1.214|B.1.215|B.1.218|B.1.22|B.1.22.1|B.1.220|B.1.223|B.1.224|B.1.225|B.1.227|B.1.229|B.1.23|B.1.232|B.1.233|B.1.234|B.1.236|B.1.237|B.1.239|B.1.241|B.1.245|B.1.247|B.1.250|B.1.252|B.1.263|B.1.264|B.1.264.1|B.1.265|B.1.267|B.1.268|B.1.270|B.1.273|B.1.274|B.1.276|B.1.277|B.1.279|B.1.280|B.1.281|B.1.284|B.1.285|B.1.291|B.1.293|B.1.294|B.1.298|B.1.301|B.1.302|B.1.304|B.1.306|B.1.308|B.1.309|B.1.310|B.1.311|B.1.314|B.1.319|B.1.320|B.1.321|B.1.323|B.1.324|B.1.328|B.1.329|B.1.330|B.1.332|B.1.333|B.1.334|B.1.335|B.1.336|B.1.337|B.1.338|B.1.340|B.1.341|B.1.342|B.1.343|B.1.344|B.1.346|B.1.348|B.1.349|B.1.354|B.1.355|B.1.356|B.1.357|B.1.358|B.1.359|B.1.36.19|B.1.36.22|B.1.360|B.1.361|B.1.362|B.1.362.2|B.1.363|B.1.369|B.1.369.1|B.1.37|B.1.371|B.1.377|B.1.378|B.1.379|B.1.38|B.1.380|B.1.381|B.1.382|B.1.383|B.1.384|B.1.385|B.1.387|B.1.39|B.1.390|B.1.391|B.1.395|B.1.397|B.1.399|B.1.400|B.1.401|B.1.403|B.1.408|B.1.409|B.1.411|B.1.413|B.1.415|B.1.416|B.1.416.1|B.1.417|B.1.422|B.1.423|B.1.424|B.1.425|B.1.428|B.1.432|B.1.433|B.1.434|B.1.435|B.1.436|B.1.437|B.1.438|B.1.439|B.1.441|B.1.442|B.1.443|B.1.444|B.1.445|B.1.446|B.1.450|B.1.451|B.1.452|B.1.453|B.1.456|B.1.459|B.1.466|B.1.467|B.1.469|B.1.470|B.1.475|B.1.478|B.1.479|B.1.482|B.1.483|B.1.485|B.1.486|B.1.487|B.1.492|B.1.493|B.1.494|B.1.495|B.1.496|B.1.497|B.1.498|B.1.499|B.1.500|B.1.501|B.1.502|B.1.503|B.1.504|B.1.505|B.1.506|B.1.507|B.1.508|B.1.509|B.1.510|B.1.511|B.1.513|B.1.516|B.1.518|B.1.520|B.1.521|B.1.523|B.1.527|B.1.528|B.1.530|B.1.535|B.1.537|B.1.538|B.1.539|B.1.540|B.1.541|B.1.544|B.1.545|B.1.548|B.1.555|B.1.556|B.1.558|B.1.560|B.1.564|B.1.565|B.1.566|B.1.567|B.1.568|B.1.569|B.1.571|B.1.573|B.1.574|B.1.576|B.1.577|B.1.578|B.1.580|B.1.581|B.1.582|B.1.585|B.1.586|B.1.589|B.1.590|B.1.592|B.1.593|B.1.595|B.1.595.3|B.1.595.4|B.1.596|B.1.596.1|B.1.597|B.1.598|B.1.6|B.1.600|B.1.601|B.1.604|B.1.609|B.1.611|B.1.612|B.1.613|B.1.615|B.1.67|B.1.76|B.1.78|B.1.83|B.1.84|B.1.9|B.1.9.3|B.1.9.4|B.1.9.5|B.1.93|B.1.94|B.1.96|B.4.8|D.4|D.5|K.1|K.2|L.2|N.1|N.3|N.4|N.6|N.8|S.1
CUY17-003867	Standard-S	Unassigned	Unassigned	NA
CUY17-003892	Standard-S	B.1.617.2	B.1.617.2_7	AY.26|AY.65
CUY17-003893	Standard-S	AY.48	AY.48	AY.48
CUY17-003901	Standard-S	B.1.617.2	B.1.617.2_2	AY.109|AY.122|AY.122.4|AY.13|AY.14|AY.15|AY.16|AY.16.1|AY.25|AY.3|AY.3.2|AY.43|AY.43.8|AY.43.9|AY.44|AY.46|AY.46.1|AY.46.6|AY.51|AY.54|AY.6|AY.7|AY.7.2|AY.76|AY.77|AY.79|AY.88|AY.91|AY.91.1|AY.98
CUY17-003909	Standard-S	A	A_1	A.18|AE.2|AE.4|AE.7|AE.8|AF.1|AG.1|AN.1|AP.1|AQ.1|B.1|B.1.1|B.1.1.10|B.1.1.101|B.1.1.110|B.1.1.111|B.1.1.113|B.1.1.12|B.1.1.121|B.1.1.127|B.1.1.128|B.1.1.129|B.1.1.132|B.1.1.133|B.1.1.134|B.1.1.135|B.1.1.139|B.1.1.148|B.1.1.152|B.1.1.157|B.1.1.158|B.1.1.159|B.1.1.162|B.1.1.163|B.1.1.164|B.1.1.166|B.1.1.169|B.1.1.171|B.1.1.172|B.1.1.174|B.1.1.176|B.1.1.177|B.1.1.181|B.1.1.182|B.1.1.184|B.1.1.185|B.1.1.187|B.1.1.189|B.1.1.192|B.1.1.198|B.1.1.200|B.1.1.201|B.1.1.204|B.1.1.205|B.1.1.209|B.1.1.210|B.1.1.214|B.1.1.216|B.1.1.217|B.1.1.218|B.1.1.219|B.1.1.225|B.1.1.226|B.1.1.229|B.1.1.230|B.1.1.231|B.1.1.232|B.1.1.236|B.1.1.243|B.1.1.244|B.1.1.25|B.1.1.253|B.1.1.254|B.1.1.257|B.1.1.258|B.1.1.26|B.1.1.261|B.1.1.265|B.1.1.266|B.1.1.267|B.1.1.268|B.1.1.27|B.1.1.271|B.1.1.274|B.1.1.277|B.1.1.282|B.1.1.283|B.1.1.284|B.1.1.285|B.1.1.286|B.1.1.288|B.1.1.290|B.1.1.291|B.1.1.297|B.1.1.301|B.1.1.304|B.1.1.305|B.1.1.306|B.1.1.307|B.1.1.308|B.1.1.31|B.1.1.315|B.1.1.317|B.1.1.319|B.1.1.323|B.1.1.324|B.1.1.325|B.1.1.326|B.1.1.328|B.1.1.329|B.1.1.33|B.1.1.331|B.1.1.334|B.1.1.335|B.1.1.34|B.1.1.342|B.1.1.344|B.1.1.346|B.1.1.347|B.1.1.348|B.1.1.350|B.1.1.352|B.1.1.354|B.1.1.355|B.1.1.356|B.1.1.357|B.1.1.359|B.1.1.366|B.1.1.367|B.1.1.368|B.1.1.370|B.1.1.373|B.1.1.376|B.1.1.378|B.1.1.381|B.1.1.382|B.1.1.383|B.1.1.386|B.1.1.387|B.1.1.388|B.1.1.39|B.1.1.391|B.1.1.392|B.1.1.398|B.1.1.40|B.1.1.401|B.1.1.402|B.1.1.404|B.1.1.405|B.1.1.406|B.1.1.407|B.1.1.409|B.1.1.410|B.1.1.411|B.1.1.414|B.1.1.416|B.1.1.419|B.1.1.422|B.1.1.424|B.1.1.426|B.1.1.427|B.1.1.430|B.1.1.431|B.1.1.432|B.1.1.434|B.1.1.435|B.1.1.436|B.1.1.437|B.1.1.438|B.1.1.44|B.1.1.440|B.1.1.441|B.1.1.442|B.1.1.447|B.1.1.448|B.1.1.450|B.1.1.452|B.1.1.453|B.1.1.456|B.1.1.458|B.1.1.459|B.1.1.46|B.1.1.465|B.1.1.466|B.1.1.47|B.1.1.48|B.1.1.481|B.1.1.482|B.1.1.484|B.1.1.485|B.1.1.487|B.1.1.5|B.1.1.50|B.1.1.506|B.1.1.507|B.1.1.513|B.1.1.514|B.1.1.515|B.1.1.516|B.1.1.518|B.1.1.52|B.1.1.53|B.1.1.54|B.1.1.57|B.1.1.61|B.1.1.62|B.1.1.67|B.1.1.70|B.1.1.71|B.1.1.77|B.1.1.8|B.1.1.87|B.1.1.88|B.1.1.89|B.1.1.98|B.1.1.99|B.1.103|B.1.104|B.1.106|B.1.108|B.1.110|B.1.110.1|B.1.110.3|B.1.111|B.1.112|B.1.113|B.1.115|B.1.116|B.1.118|B.1.119|B.1.12|B.1.124|B.1.13|B.1.131|B.1.134|B.1.137|B.1.139|B.1.142|B.1.143|B.1.145|B.1.146|B.1.147|B.1.149|B.1.151|B.1.153|B.1.157|B.1.160.32|B.1.161|B.1.162|B.1.163|B.1.164|B.1.166|B.1.167|B.1.169|B.1.170|B.1.177.31|B.1.178|B.1.179|B.1.180|B.1.181|B.1.182|B.1.184|B.1.187|B.1.188|B.1.189|B.1.190|B.1.192|B.1.194|B.1.195|B.1.199|B.1.2|B.1.201|B.1.203|B.1.206|B.1.208|B.1.210|B.1.211|B.1.212|B.1.213|B.1.214|B.1.215|B.1.218|B.1.22|B.1.22.1|B.1.220|B.1.223|B.1.224|B.1.225|B.1.227|B.1.229|B.1.23|B.1.232|B.1.233|B.1.234|B.1.236|B.1.237|B.1.239|B.1.241|B.1.245|B.1.247|B.1.250|B.1.252|B.1.263|B.1.264|B.1.264.1|B.1.265|B.1.267|B.1.268|B.1.270|B.1.273|B.1.274|B.1.276|B.1.277|B.1.279|B.1.280|B.1.281|B.1.284|B.1.285|B.1.291|B.1.293|B.1.294|B.1.298|B.1.301|B.1.302|B.1.304|B.1.306|B.1.308|B.1.309|B.1.310|B.1.311|B.1.314|B.1.319|B.1.320|B.1.321|B.1.323|B.1.324|B.1.328|B.1.329|B.1.330|B.1.332|B.1.333|B.1.334|B.1.335|B.1.336|B.1.337|B.1.338|B.1.340|B.1.341|B.1.342|B.1.343|B.1.344|B.1.346|B.1.348|B.1.349|B.1.354|B.1.355|B.1.356|B.1.357|B.1.358|B.1.359|B.1.36.19|B.1.36.22|B.1.360|B.1.361|B.1.362|B.1.362.2|B.1.363|B.1.369|B.1.369.1|B.1.37|B.1.371|B.1.377|B.1.378|B.1.379|B.1.38|B.1.380|B.1.381|B.1.382|B.1.383|B.1.384|B.1.385|B.1.387|B.1.39|B.1.390|B.1.391|B.1.395|B.1.397|B.1.399|B.1.400|B.1.401|B.1.403|B.1.408|B.1.409|B.1.411|B.1.413|B.1.415|B.1.416|B.1.416.1|B.1.417|B.1.422|B.1.423|B.1.424|B.1.425|B.1.428|B.1.432|B.1.433|B.1.434|B.1.435|B.1.436|B.1.437|B.1.438|B.1.439|B.1.441|B.1.442|B.1.443|B.1.444|B.1.445|B.1.446|B.1.450|B.1.451|B.1.452|B.1.453|B.1.456|B.1.459|B.1.466|B.1.467|B.1.469|B.1.470|B.1.475|B.1.478|B.1.479|B.1.482|B.1.483|B.1.485|B.1.486|B.1.487|B.1.492|B.1.493|B.1.494|B.1.495|B.1.496|B.1.497|B.1.498|B.1.499|B.1.500|B.1.501|B.1.502|B.1.503|B.1.504|B.1.505|B.1.506|B.1.507|B.1.508|B.1.509|B.1.510|B.1.511|B.1.513|B.1.516|B.1.518|B.1.520|B.1.521|B.1.523|B.1.527|B.1.528|B.1.530|B.1.535|B.1.537|B.1.538|B.1.539|B.1.540|B.1.541|B.1.544|B.1.545|B.1.548|B.1.555|B.1.556|B.1.558|B.1.560|B.1.564|B.1.565|B.1.566|B.1.567|B.1.568|B.1.569|B.1.571|B.1.573|B.1.574|B.1.576|B.1.577|B.1.578|B.1.580|B.1.581|B.1.582|B.1.585|B.1.586|B.1.589|B.1.590|B.1.592|B.1.593|B.1.595|B.1.595.3|B.1.595.4|B.1.596|B.1.596.1|B.1.597|B.1.598|B.1.6|B.1.600|B.1.601|B.1.604|B.1.609|B.1.611|B.1.612|B.1.613|B.1.615|B.1.67|B.1.76|B.1.78|B.1.83|B.1.84|B.1.9|B.1.9.3|B.1.9.4|B.1.9.5|B.1.93|B.1.94|B.1.96|B.4.8|D.4|D.5|K.1|K.2|L.2|N.1|N.3|N.4|N.6|N.8|S.1
CUY17-003910	Standard-S	A	A_11	AY.17|AY.25.1|AY.25.1.2|AY.46.5|AY.7.1|AY.80|AY.83|AY.93|B.1.617.2
CUY17-003939	Standard-S	P.1	P.1_1	P.1|P.1.1|P.1.10|P.1.10.1|P.1.12|P.1.13|P.1.15|P.1.16|P.1.2
CUY18-004019	Standard-S	B.1.621	B.1.621	B.1.621

CUY18-004022	Standard-S	AY.33.2	AY.33.2	AY.33.2
CUY18-004023	Standard-S	AY.33.2	AY.33.2	AY.33.2
CUY18-004026	Standard-S	B.1.621.1	B.1.621.1	B.1.621.1
CUY19-004183	Standard-S	C.37	C.37	C.37
CUY23-004816	Standard-S	AY.20	AY.20	AY.20
CUY24-004876	Standard-S	A	A_11	AY.17|AY.25.1|AY.25.1.2|AY.46.5|AY.7.1|AY.80|AY.83|AY.93|B.1.617.2
CUY24-004954	Standard-S	A	A_11	AY.17|AY.25.1|AY.25.1.2|AY.46.5|AY.7.1|AY.80|AY.83|AY.93|B.1.617.2
CUY26-005235	Standard-S	A	A_2	A|A.1|A.15|A.16|A.17|A.2|A.2.2|A.2.4|A.21|A.22|A.24|A.25|A.4|A.5|A.7|B|B.1.1.161|B.1.1.220|B.1.1.340|B.1.1.371|B.1.14|B.1.214.1|B.1.260|B.1.393|B.1.462|B.1.473|B.1.533|B.10|B.11|B.12|B.13|B.18|B.19|B.20|B.23|B.26|B.27|B.28|B.3|B.3.1|B.30|B.31|B.32|B.35|B.36|B.37|B.38|B.39|B.4|B.4.1|B.4.2|B.4.4|B.4.5|B.4.6|B.4.7|B.40|B.41|B.42|B.44|B.49|B.5|B.50|B.51|B.52|B.55|B.56|B.57|B.58|B.6|B.61
CUY42-007286	Standard-S	A	A_1	A.18|AE.2|AE.4|AE.7|AE.8|AF.1|AG.1|AN.1|AP.1|AQ.1|B.1|B.1.1|B.1.1.10|B.1.1.101|B.1.1.110|B.1.1.111|B.1.1.113|B.1.1.12|B.1.1.121|B.1.1.127|B.1.1.128|B.1.1.129|B.1.1.132|B.1.1.133|B.1.1.134|B.1.1.135|B.1.1.139|B.1.1.148|B.1.1.152|B.1.1.157|B.1.1.158|B.1.1.159|B.1.1.162|B.1.1.163|B.1.1.164|B.1.1.166|B.1.1.169|B.1.1.171|B.1.1.172|B.1.1.174|B.1.1.176|B.1.1.177|B.1.1.181|B.1.1.182|B.1.1.184|B.1.1.185|B.1.1.187|B.1.1.189|B.1.1.192|B.1.1.198|B.1.1.200|B.1.1.201|B.1.1.204|B.1.1.205|B.1.1.209|B.1.1.210|B.1.1.214|B.1.1.216|B.1.1.217|B.1.1.218|B.1.1.219|B.1.1.225|B.1.1.226|B.1.1.229|B.1.1.230|B.1.1.231|B.1.1.232|B.1.1.236|B.1.1.243|B.1.1.244|B.1.1.25|B.1.1.253|B.1.1.254|B.1.1.257|B.1.1.258|B.1.1.26|B.1.1.261|B.1.1.265|B.1.1.266|B.1.1.267|B.1.1.268|B.1.1.27|B.1.1.271|B.1.1.274|B.1.1.277|B.1.1.282|B.1.1.283|B.1.1.284|B.1.1.285|B.1.1.286|B.1.1.288|B.1.1.290|B.1.1.291|B.1.1.297|B.1.1.301|B.1.1.304|B.1.1.305|B.1.1.306|B.1.1.307|B.1.1.308|B.1.1.31|B.1.1.315|B.1.1.317|B.1.1.319|B.1.1.323|B.1.1.324|B.1.1.325|B.1.1.326|B.1.1.328|B.1.1.329|B.1.1.33|B.1.1.331|B.1.1.334|B.1.1.335|B.1.1.34|B.1.1.342|B.1.1.344|B.1.1.346|B.1.1.347|B.1.1.348|B.1.1.350|B.1.1.352|B.1.1.354|B.1.1.355|B.1.1.356|B.1.1.357|B.1.1.359|B.1.1.366|B.1.1.367|B.1.1.368|B.1.1.370|B.1.1.373|B.1.1.376|B.1.1.378|B.1.1.381|B.1.1.382|B.1.1.383|B.1.1.386|B.1.1.387|B.1.1.388|B.1.1.39|B.1.1.391|B.1.1.392|B.1.1.398|B.1.1.40|B.1.1.401|B.1.1.402|B.1.1.404|B.1.1.405|B.1.1.406|B.1.1.407|B.1.1.409|B.1.1.410|B.1.1.411|B.1.1.414|B.1.1.416|B.1.1.419|B.1.1.422|B.1.1.424|B.1.1.426|B.1.1.427|B.1.1.430|B.1.1.431|B.1.1.432|B.1.1.434|B.1.1.435|B.1.1.436|B.1.1.437|B.1.1.438|B.1.1.44|B.1.1.440|B.1.1.441|B.1.1.442|B.1.1.447|B.1.1.448|B.1.1.450|B.1.1.452|B.1.1.453|B.1.1.456|B.1.1.458|B.1.1.459|B.1.1.46|B.1.1.465|B.1.1.466|B.1.1.47|B.1.1.48|B.1.1.481|B.1.1.482|B.1.1.484|B.1.1.485|B.1.1.487|B.1.1.5|B.1.1.50|B.1.1.506|B.1.1.507|B.1.1.513|B.1.1.514|B.1.1.515|B.1.1.516|B.1.1.518|B.1.1.52|B.1.1.53|B.1.1.54|B.1.1.57|B.1.1.61|B.1.1.62|B.1.1.67|B.1.1.70|B.1.1.71|B.1.1.77|B.1.1.8|B.1.1.87|B.1.1.88|B.1.1.89|B.1.1.98|B.1.1.99|B.1.103|B.1.104|B.1.106|B.1.108|B.1.110|B.1.110.1|B.1.110.3|B.1.111|B.1.112|B.1.113|B.1.115|B.1.116|B.1.118|B.1.119|B.1.12|B.1.124|B.1.13|B.1.131|B.1.134|B.1.137|B.1.139|B.1.142|B.1.143|B.1.145|B.1.146|B.1.147|B.1.149|B.1.151|B.1.153|B.1.157|B.1.160.32|B.1.161|B.1.162|B.1.163|B.1.164|B.1.166|B.1.167|B.1.169|B.1.170|B.1.177.31|B.1.178|B.1.179|B.1.180|B.1.181|B.1.182|B.1.184|B.1.187|B.1.188|B.1.189|B.1.190|B.1.192|B.1.194|B.1.195|B.1.199|B.1.2|B.1.201|B.1.203|B.1.206|B.1.208|B.1.210|B.1.211|B.1.212|B.1.213|B.1.214|B.1.215|B.1.218|B.1.22|B.1.22.1|B.1.220|B.1.223|B.1.224|B.1.225|B.1.227|B.1.229|B.1.23|B.1.232|B.1.233|B.1.234|B.1.236|B.1.237|B.1.239|B.1.241|B.1.245|B.1.247|B.1.250|B.1.252|B.1.263|B.1.264|B.1.264.1|B.1.265|B.1.267|B.1.268|B.1.270|B.1.273|B.1.274|B.1.276|B.1.277|B.1.279|B.1.280|B.1.281|B.1.284|B.1.285|B.1.291|B.1.293|B.1.294|B.1.298|B.1.301|B.1.302|B.1.304|B.1.306|B.1.308|B.1.309|B.1.310|B.1.311|B.1.314|B.1.319|B.1.320|B.1.321|B.1.323|B.1.324|B.1.328|B.1.329|B.1.330|B.1.332|B.1.333|B.1.334|B.1.335|B.1.336|B.1.337|B.1.338|B.1.340|B.1.341|B.1.342|B.1.343|B.1.344|B.1.346|B.1.348|B.1.349|B.1.354|B.1.355|B.1.356|B.1.357|B.1.358|B.1.359|B.1.36.19|B.1.36.22|B.1.360|B.1.361|B.1.362|B.1.362.2|B.1.363|B.1.369|B.1.369.1|B.1.37|B.1.371|B.1.377|B.1.378|B.1.379|B.1.38|B.1.380|B.1.381|B.1.382|B.1.383|B.1.384|B.1.385|B.1.387|B.1.39|B.1.390|B.1.391|B.1.395|B.1.397|B.1.399|B.1.400|B.1.401|B.1.403|B.1.408|B.1.409|B.1.411|B.1.413|B.1.415|B.1.416|B.1.416.1|B.1.417|B.1.422|B.1.423|B.1.424|B.1.425|B.1.428|B.1.432|B.1.433|B.1.434|B.1.435|B.1.436|B.1.437|B.1.438|B.1.439|B.1.441|B.1.442|B.1.443|B.1.444|B.1.445|B.1.446|B.1.450|B.1.451|B.1.452|B.1.453|B.1.456|B.1.459|B.1.466|B.1.467|B.1.469|B.1.470|B.1.475|B.1.478|B.1.479|B.1.482|B.1.483|B.1.485|B.1.486|B.1.487|B.1.492|B.1.493|B.1.494|B.1.495|B.1.496|B.1.497|B.1.498|B.1.499|B.1.500|B.1.501|B.1.502|B.1.503|B.1.504|B.1.505|B.1.506|B.1.507|B.1.508|B.1.509|B.1.510|B.1.511|B.1.513|B.1.516|B.1.518|B.1.520|B.1.521|B.1.523|B.1.527|B.1.528|B.1.530|B.1.535|B.1.537|B.1.538|B.1.539|B.1.540|B.1.541|B.1.544|B.1.545|B.1.548|B.1.555|B.1.556|B.1.558|B.1.560|B.1.564|B.1.565|B.1.566|B.1.567|B.1.568|B.1.569|B.1.571|B.1.573|B.1.574|B.1.576|B.1.577|B.1.578|B.1.580|B.1.581|B.1.582|B.1.585|B.1.586|B.1.589|B.1.590|B.1.592|B.1.593|B.1.595|B.1.595.3|B.1.595.4|B.1.596|B.1.596.1|B.1.597|B.1.598|B.1.6|B.1.600|B.1.601|B.1.604|B.1.609|B.1.611|B.1.612|B.1.613|B.1.615|B.1.67|B.1.76|B.1.78|B.1.83|B.1.84|B.1.9|B.1.9.3|B.1.9.4|B.1.9.5|B.1.93|B.1.94|B.1.96|B.4.8|D.4|D.5|K.1|K.2|L.2|N.1|N.3|N.4|N.6|N.8|S.1
CUY42-007288	Standard-S	BA.1.15	BA.1.15	BA.1.15
CUY42-007289	Standard-S	BA.1.15	BA.1.15	BA.1.15
CUY42-007291	Standard-S	A	A_1	A.18|AE.2|AE.4|AE.7|AE.8|AF.1|AG.1|AN.1|AP.1|AQ.1|B.1|B.1.1|B.1.1.10|B.1.1.101|B.1.1.110|B.1.1.111|B.1.1.113|B.1.1.12|B.1.1.121|B.1.1.127|B.1.1.128|B.1.1.129|B.1.1.132|B.1.1.133|B.1.1.134|B.1.1.135|B.1.1.139|B.1.1.148|B.1.1.152|B.1.1.157|B.1.1.158|B.1.1.159|B.1.1.162|B.1.1.163|B.1.1.164|B.1.1.166|B.1.1.169|B.1.1.171|B.1.1.172|B.1.1.174|B.1.1.176|B.1.1.177|B.1.1.181|B.1.1.182|B.1.1.184|B.1.1.185|B.1.1.187|B.1.1.189|B.1.1.192|B.1.1.198|B.1.1.200|B.1.1.201|B.1.1.204|B.1.1.205|B.1.1.209|B.1.1.210|B.1.1.214|B.1.1.216|B.1.1.217|B.1.1.218|B.1.1.219|B.1.1.225|B.1.1.226|B.1.1.229|B.1.1.230|B.1.1.231|B.1.1.232|B.1.1.236|B.1.1.243|B.1.1.244|B.1.1.25|B.1.1.253|B.1.1.254|B.1.1.257|B.1.1.258|B.1.1.26|B.1.1.261|B.1.1.265|B.1.1.266|B.1.1.267|B.1.1.268|B.1.1.27|B.1.1.271|B.1.1.274|B.1.1.277|B.1.1.282|B.1.1.283|B.1.1.284|B.1.1.285|B.1.1.286|B.1.1.288|B.1.1.290|B.1.1.291|B.1.1.297|B.1.1.301|B.1.1.304|B.1.1.305|B.1.1.306|B.1.1.307|B.1.1.308|B.1.1.31|B.1.1.315|B.1.1.317|B.1.1.319|B.1.1.323|B.1.1.324|B.1.1.325|B.1.1.326|B.1.1.328|B.1.1.329|B.1.1.33|B.1.1.331|B.1.1.334|B.1.1.335|B.1.1.34|B.1.1.342|B.1.1.344|B.1.1.346|B.1.1.347|B.1.1.348|B.1.1.350|B.1.1.352|B.1.1.354|B.1.1.355|B.1.1.356|B.1.1.357|B.1.1.359|B.1.1.366|B.1.1.367|B.1.1.368|B.1.1.370|B.1.1.373|B.1.1.376|B.1.1.378|B.1.1.381|B.1.1.382|B.1.1.383|B.1.1.386|B.1.1.387|B.1.1.388|B.1.1.39|B.1.1.391|B.1.1.392|B.1.1.398|B.1.1.40|B.1.1.401|B.1.1.402|B.1.1.404|B.1.1.405|B.1.1.406|B.1.1.407|B.1.1.409|B.1.1.410|B.1.1.411|B.1.1.414|B.1.1.416|B.1.1.419|B.1.1.422|B.1.1.424|B.1.1.426|B.1.1.427|B.1.1.430|B.1.1.431|B.1.1.432|B.1.1.434|B.1.1.435|B.1.1.436|B.1.1.437|B.1.1.438|B.1.1.44|B.1.1.440|B.1.1.441|B.1.1.442|B.1.1.447|B.1.1.448|B.1.1.450|B.1.1.452|B.1.1.453|B.1.1.456|B.1.1.458|B.1.1.459|B.1.1.46|B.1.1.465|B.1.1.466|B.1.1.47|B.1.1.48|B.1.1.481|B.1.1.482|B.1.1.484|B.1.1.485|B.1.1.487|B.1.1.5|B.1.1.50|B.1.1.506|B.1.1.507|B.1.1.513|B.1.1.514|B.1.1.515|B.1.1.516|B.1.1.518|B.1.1.52|B.1.1.53|B.1.1.54|B.1.1.57|B.1.1.61|B.1.1.62|B.1.1.67|B.1.1.70|B.1.1.71|B.1.1.77|B.1.1.8|B.1.1.87|B.1.1.88|B.1.1.89|B.1.1.98|B.1.1.99|B.1.103|B.1.104|B.1.106|B.1.108|B.1.110|B.1.110.1|B.1.110.3|B.1.111|B.1.112|B.1.113|B.1.115|B.1.116|B.1.118|B.1.119|B.1.12|B.1.124|B.1.13|B.1.131|B.1.134|B.1.137|B.1.139|B.1.142|B.1.143|B.1.145|B.1.146|B.1.147|B.1.149|B.1.151|B.1.153|B.1.157|B.1.160.32|B.1.161|B.1.162|B.1.163|B.1.164|B.1.166|B.1.167|B.1.169|B.1.170|B.1.177.31|B.1.178|B.1.179|B.1.180|B.1.181|B.1.182|B.1.184|B.1.187|B.1.188|B.1.189|B.1.190|B.1.192|B.1.194|B.1.195|B.1.199|B.1.2|B.1.201|B.1.203|B.1.206|B.1.208|B.1.210|B.1.211|B.1.212|B.1.213|B.1.214|B.1.215|B.1.218|B.1.22|B.1.22.1|B.1.220|B.1.223|B.1.224|B.1.225|B.1.227|B.1.229|B.1.23|B.1.232|B.1.233|B.1.234|B.1.236|B.1.237|B.1.239|B.1.241|B.1.245|B.1.247|B.1.250|B.1.252|B.1.263|B.1.264|B.1.264.1|B.1.265|B.1.267|B.1.268|B.1.270|B.1.273|B.1.274|B.1.276|B.1.277|B.1.279|B.1.280|B.1.281|B.1.284|B.1.285|B.1.291|B.1.293|B.1.294|B.1.298|B.1.301|B.1.302|B.1.304|B.1.306|B.1.308|B.1.309|B.1.310|B.1.311|B.1.314|B.1.319|B.1.320|B.1.321|B.1.323|B.1.324|B.1.328|B.1.329|B.1.330|B.1.332|B.1.333|B.1.334|B.1.335|B.1.336|B.1.337|B.1.338|B.1.340|B.1.341|B.1.342|B.1.343|B.1.344|B.1.346|B.1.348|B.1.349|B.1.354|B.1.355|B.1.356|B.1.357|B.1.358|B.1.359|B.1.36.19|B.1.36.22|B.1.360|B.1.361|B.1.362|B.1.362.2|B.1.363|B.1.369|B.1.369.1|B.1.37|B.1.371|B.1.377|B.1.378|B.1.379|B.1.38|B.1.380|B.1.381|B.1.382|B.1.383|B.1.384|B.1.385|B.1.387|B.1.39|B.1.390|B.1.391|B.1.395|B.1.397|B.1.399|B.1.400|B.1.401|B.1.403|B.1.408|B.1.409|B.1.411|B.1.413|B.1.415|B.1.416|B.1.416.1|B.1.417|B.1.422|B.1.423|B.1.424|B.1.425|B.1.428|B.1.432|B.1.433|B.1.434|B.1.435|B.1.436|B.1.437|B.1.438|B.1.439|B.1.441|B.1.442|B.1.443|B.1.444|B.1.445|B.1.446|B.1.450|B.1.451|B.1.452|B.1.453|B.1.456|B.1.459|B.1.466|B.1.467|B.1.469|B.1.470|B.1.475|B.1.478|B.1.479|B.1.482|B.1.483|B.1.485|B.1.486|B.1.487|B.1.492|B.1.493|B.1.494|B.1.495|B.1.496|B.1.497|B.1.498|B.1.499|B.1.500|B.1.501|B.1.502|B.1.503|B.1.504|B.1.505|B.1.506|B.1.507|B.1.508|B.1.509|B.1.510|B.1.511|B.1.513|B.1.516|B.1.518|B.1.520|B.1.521|B.1.523|B.1.527|B.1.528|B.1.530|B.1.535|B.1.537|B.1.538|B.1.539|B.1.540|B.1.541|B.1.544|B.1.545|B.1.548|B.1.555|B.1.556|B.1.558|B.1.560|B.1.564|B.1.565|B.1.566|B.1.567|B.1.568|B.1.569|B.1.571|B.1.573|B.1.574|B.1.576|B.1.577|B.1.578|B.1.580|B.1.581|B.1.582|B.1.585|B.1.586|B.1.589|B.1.590|B.1.592|B.1.593|B.1.595|B.1.595.3|B.1.595.4|B.1.596|B.1.596.1|B.1.597|B.1.598|B.1.6|B.1.600|B.1.601|B.1.604|B.1.609|B.1.611|B.1.612|B.1.613|B.1.615|B.1.67|B.1.76|B.1.78|B.1.83|B.1.84|B.1.9|B.1.9.3|B.1.9.4|B.1.9.5|B.1.93|B.1.94|B.1.96|B.4.8|D.4|D.5|K.1|K.2|L.2|N.1|N.3|N.4|N.6|N.8|S.1
CUY42-007294	Standard-S	BA.1	BA.1_4	BA.1|BA.1.20
CUY1-000006	Fast-S	P.2	P.2	P.2
CUY4-000486	Fast-S	P.2	P.2	P.2
CUY5-000547	Fast-S	P.6	P.6	P.6
CUY6-001233	Fast-S	A	A_5	B.1.1.1|B.1.1.369|B.1.1.372|C.1|C.10|C.11|C.12|C.13|C.14|C.18|C.2|C.20|C.21|C.22|C.23|C.26|C.28|C.29|C.32|C.33|C.34|C.35|C.36|C.5|C.6|C.8|C.9
CUY8-001646	Fast-S	C.37.1	C.37.1	C.37.1
CUY12-002713	Fast-S	P.1	P.1_1	P.1|P.1.1|P.1.10|P.1.10.1|P.1.12|P.1.13|P.1.15|P.1.16|P.1.2
CUY12-002716	Fast-S	P.1	P.1_1	P.1|P.1.1|P.1.10|P.1.10.1|P.1.12|P.1.13|P.1.15|P.1.16|P.1.2
CUY12-002721	Fast-S	P.1	P.1_1	P.1|P.1.1|P.1.10|P.1.10.1|P.1.12|P.1.13|P.1.15|P.1.16|P.1.2
CUY12-002725	Fast-S	P.1	P.1_1	P.1|P.1.1|P.1.10|P.1.10.1|P.1.12|P.1.13|P.1.15|P.1.16|P.1.2
CUY16-003496	Fast-S	P.2	P.2	P.2

CUY16-003498	Fast-S	A	A_1	A.18|AE.2|AE.4|AE.7|AE.8|AF.1|AG.1|AN.1|AP.1|AQ.1|B.1|B.1.1|B.1.1.10|B.1.1.101|B.1.1.110|B.1.1.111|B.1.1.113|B.1.1.12|B.1.1.121|B.1.1.127|B.1.1.128|B.1.1.129|B.1.1.132|B.1.1.133|B.1.1.134|B.1.1.135|B.1.1.139|B.1.1.148|B.1.1.152|B.1.1.157|B.1.1.158|B.1.1.159|B.1.1.162|B.1.1.163|B.1.1.164|B.1.1.166|B.1.1.169|B.1.1.171|B.1.1.172|B.1.1.174|B.1.1.176|B.1.1.177|B.1.1.181|B.1.1.182|B.1.1.184|B.1.1.185|B.1.1.187|B.1.1.189|B.1.1.192|B.1.1.198|B.1.1.200|B.1.1.201|B.1.1.204|B.1.1.205|B.1.1.209|B.1.1.210|B.1.1.214|B.1.1.216|B.1.1.217|B.1.1.218|B.1.1.219|B.1.1.225|B.1.1.226|B.1.1.229|B.1.1.230|B.1.1.231|B.1.1.232|B.1.1.236|B.1.1.243|B.1.1.244|B.1.1.25|B.1.1.253|B.1.1.254|B.1.1.257|B.1.1.258|B.1.1.26|B.1.1.261|B.1.1.265|B.1.1.266|B.1.1.267|B.1.1.268|B.1.1.27|B.1.1.271|B.1.1.274|B.1.1.277|B.1.1.282|B.1.1.283|B.1.1.284|B.1.1.285|B.1.1.286|B.1.1.288|B.1.1.290|B.1.1.291|B.1.1.297|B.1.1.301|B.1.1.304|B.1.1.305|B.1.1.306|B.1.1.307|B.1.1.308|B.1.1.31|B.1.1.315|B.1.1.317|B.1.1.319|B.1.1.323|B.1.1.324|B.1.1.325|B.1.1.326|B.1.1.328|B.1.1.329|B.1.1.33|B.1.1.331|B.1.1.334|B.1.1.335|B.1.1.34|B.1.1.342|B.1.1.344|B.1.1.346|B.1.1.347|B.1.1.348|B.1.1.350|B.1.1.352|B.1.1.354|B.1.1.355|B.1.1.356|B.1.1.357|B.1.1.359|B.1.1.366|B.1.1.367|B.1.1.368|B.1.1.370|B.1.1.373|B.1.1.376|B.1.1.378|B.1.1.381|B.1.1.382|B.1.1.383|B.1.1.386|B.1.1.387|B.1.1.388|B.1.1.39|B.1.1.391|B.1.1.392|B.1.1.398|B.1.1.40|B.1.1.401|B.1.1.402|B.1.1.404|B.1.1.405|B.1.1.406|B.1.1.407|B.1.1.409|B.1.1.410|B.1.1.411|B.1.1.414|B.1.1.416|B.1.1.419|B.1.1.422|B.1.1.424|B.1.1.426|B.1.1.427|B.1.1.430|B.1.1.431|B.1.1.432|B.1.1.434|B.1.1.435|B.1.1.436|B.1.1.437|B.1.1.438|B.1.1.44|B.1.1.440|B.1.1.441|B.1.1.442|B.1.1.447|B.1.1.448|B.1.1.450|B.1.1.452|B.1.1.453|B.1.1.456|B.1.1.458|B.1.1.459|B.1.1.46|B.1.1.465|B.1.1.466|B.1.1.47|B.1.1.48|B.1.1.481|B.1.1.482|B.1.1.484|B.1.1.485|B.1.1.487|B.1.1.5|B.1.1.50|B.1.1.506|B.1.1.507|B.1.1.513|B.1.1.514|B.1.1.515|B.1.1.516|B.1.1.518|B.1.1.52|B.1.1.53|B.1.1.54|B.1.1.57|B.1.1.61|B.1.1.62|B.1.1.67|B.1.1.70|B.1.1.71|B.1.1.77|B.1.1.8|B.1.1.87|B.1.1.88|B.1.1.89|B.1.1.98|B.1.1.99|B.1.103|B.1.104|B.1.106|B.1.108|B.1.110|B.1.110.1|B.1.110.3|B.1.111|B.1.112|B.1.113|B.1.115|B.1.116|B.1.118|B.1.119|B.1.12|B.1.124|B.1.13|B.1.131|B.1.134|B.1.137|B.1.139|B.1.142|B.1.143|B.1.145|B.1.146|B.1.147|B.1.149|B.1.151|B.1.153|B.1.157|B.1.160.32|B.1.161|B.1.162|B.1.163|B.1.164|B.1.166|B.1.167|B.1.169|B.1.170|B.1.177.31|B.1.178|B.1.179|B.1.180|B.1.181|B.1.182|B.1.184|B.1.187|B.1.188|B.1.189|B.1.190|B.1.192|B.1.194|B.1.195|B.1.199|B.1.2|B.1.201|B.1.203|B.1.206|B.1.208|B.1.210|B.1.211|B.1.212|B.1.213|B.1.214|B.1.215|B.1.218|B.1.22|B.1.22.1|B.1.220|B.1.223|B.1.224|B.1.225|B.1.227|B.1.229|B.1.23|B.1.232|B.1.233|B.1.234|B.1.236|B.1.237|B.1.239|B.1.241|B.1.245|B.1.247|B.1.250|B.1.252|B.1.263|B.1.264|B.1.264.1|B.1.265|B.1.267|B.1.268|B.1.270|B.1.273|B.1.274|B.1.276|B.1.277|B.1.279|B.1.280|B.1.281|B.1.284|B.1.285|B.1.291|B.1.293|B.1.294|B.1.298|B.1.301|B.1.302|B.1.304|B.1.306|B.1.308|B.1.309|B.1.310|B.1.311|B.1.314|B.1.319|B.1.320|B.1.321|B.1.323|B.1.324|B.1.328|B.1.329|B.1.330|B.1.332|B.1.333|B.1.334|B.1.335|B.1.336|B.1.337|B.1.338|B.1.340|B.1.341|B.1.342|B.1.343|B.1.344|B.1.346|B.1.348|B.1.349|B.1.354|B.1.355|B.1.356|B.1.357|B.1.358|B.1.359|B.1.36.19|B.1.36.22|B.1.360|B.1.361|B.1.362|B.1.362.2|B.1.363|B.1.369|B.1.369.1|B.1.37|B.1.371|B.1.377|B.1.378|B.1.379|B.1.38|B.1.380|B.1.381|B.1.382|B.1.383|B.1.384|B.1.385|B.1.387|B.1.39|B.1.390|B.1.391|B.1.395|B.1.397|B.1.399|B.1.400|B.1.401|B.1.403|B.1.408|B.1.409|B.1.411|B.1.413|B.1.415|B.1.416|B.1.416.1|B.1.417|B.1.422|B.1.423|B.1.424|B.1.425|B.1.428|B.1.432|B.1.433|B.1.434|B.1.435|B.1.436|B.1.437|B.1.438|B.1.439|B.1.441|B.1.442|B.1.443|B.1.444|B.1.445|B.1.446|B.1.450|B.1.451|B.1.452|B.1.453|B.1.456|B.1.459|B.1.466|B.1.467|B.1.469|B.1.470|B.1.475|B.1.478|B.1.479|B.1.482|B.1.483|B.1.485|B.1.486|B.1.487|B.1.492|B.1.493|B.1.494|B.1.495|B.1.496|B.1.497|B.1.498|B.1.499|B.1.500|B.1.501|B.1.502|B.1.503|B.1.504|B.1.505|B.1.506|B.1.507|B.1.508|B.1.509|B.1.510|B.1.511|B.1.513|B.1.516|B.1.518|B.1.520|B.1.521|B.1.523|B.1.527|B.1.528|B.1.530|B.1.535|B.1.537|B.1.538|B.1.539|B.1.540|B.1.541|B.1.544|B.1.545|B.1.548|B.1.555|B.1.556|B.1.558|B.1.560|B.1.564|B.1.565|B.1.566|B.1.567|B.1.568|B.1.569|B.1.571|B.1.573|B.1.574|B.1.576|B.1.577|B.1.578|B.1.580|B.1.581|B.1.582|B.1.585|B.1.586|B.1.589|B.1.590|B.1.592|B.1.593|B.1.595|B.1.595.3|B.1.595.4|B.1.596|B.1.596.1|B.1.597|B.1.598|B.1.6|B.1.600|B.1.601|B.1.604|B.1.609|B.1.611|B.1.612|B.1.613|B.1.615|B.1.67|B.1.76|B.1.78|B.1.83|B.1.84|B.1.9|B.1.9.3|B.1.9.4|B.1.9.5|B.1.93|B.1.94|B.1.96|B.4.8|D.4|D.5|K.1|K.2|L.2|N.1|N.3|N.4|N.6|N.8|S.1
CUY16-003501	Fast-S	P.6	P.6	P.6
CUY16-003536	Fast-S	A	A_1	A.18|AE.2|AE.4|AE.7|AE.8|AF.1|AG.1|AN.1|AP.1|AQ.1|B.1|B.1.1|B.1.1.10|B.1.1.101|B.1.1.110|B.1.1.111|B.1.1.113|B.1.1.12|B.1.1.121|B.1.1.127|B.1.1.128|B.1.1.129|B.1.1.132|B.1.1.133|B.1.1.134|B.1.1.135|B.1.1.139|B.1.1.148|B.1.1.152|B.1.1.157|B.1.1.158|B.1.1.159|B.1.1.162|B.1.1.163|B.1.1.164|B.1.1.166|B.1.1.169|B.1.1.171|B.1.1.172|B.1.1.174|B.1.1.176|B.1.1.177|B.1.1.181|B.1.1.182|B.1.1.184|B.1.1.185|B.1.1.187|B.1.1.189|B.1.1.192|B.1.1.198|B.1.1.200|B.1.1.201|B.1.1.204|B.1.1.205|B.1.1.209|B.1.1.210|B.1.1.214|B.1.1.216|B.1.1.217|B.1.1.218|B.1.1.219|B.1.1.225|B.1.1.226|B.1.1.229|B.1.1.230|B.1.1.231|B.1.1.232|B.1.1.236|B.1.1.243|B.1.1.244|B.1.1.25|B.1.1.253|B.1.1.254|B.1.1.257|B.1.1.258|B.1.1.26|B.1.1.261|B.1.1.265|B.1.1.266|B.1.1.267|B.1.1.268|B.1.1.27|B.1.1.271|B.1.1.274|B.1.1.277|B.1.1.282|B.1.1.283|B.1.1.284|B.1.1.285|B.1.1.286|B.1.1.288|B.1.1.290|B.1.1.291|B.1.1.297|B.1.1.301|B.1.1.304|B.1.1.305|B.1.1.306|B.1.1.307|B.1.1.308|B.1.1.31|B.1.1.315|B.1.1.317|B.1.1.319|B.1.1.323|B.1.1.324|B.1.1.325|B.1.1.326|B.1.1.328|B.1.1.329|B.1.1.33|B.1.1.331|B.1.1.334|B.1.1.335|B.1.1.34|B.1.1.342|B.1.1.344|B.1.1.346|B.1.1.347|B.1.1.348|B.1.1.350|B.1.1.352|B.1.1.354|B.1.1.355|B.1.1.356|B.1.1.357|B.1.1.359|B.1.1.366|B.1.1.367|B.1.1.368|B.1.1.370|B.1.1.373|B.1.1.376|B.1.1.378|B.1.1.381|B.1.1.382|B.1.1.383|B.1.1.386|B.1.1.387|B.1.1.388|B.1.1.39|B.1.1.391|B.1.1.392|B.1.1.398|B.1.1.40|B.1.1.401|B.1.1.402|B.1.1.404|B.1.1.405|B.1.1.406|B.1.1.407|B.1.1.409|B.1.1.410|B.1.1.411|B.1.1.414|B.1.1.416|B.1.1.419|B.1.1.422|B.1.1.424|B.1.1.426|B.1.1.427|B.1.1.430|B.1.1.431|B.1.1.432|B.1.1.434|B.1.1.435|B.1.1.436|B.1.1.437|B.1.1.438|B.1.1.44|B.1.1.440|B.1.1.441|B.1.1.442|B.1.1.447|B.1.1.448|B.1.1.450|B.1.1.452|B.1.1.453|B.1.1.456|B.1.1.458|B.1.1.459|B.1.1.46|B.1.1.465|B.1.1.466|B.1.1.47|B.1.1.48|B.1.1.481|B.1.1.482|B.1.1.484|B.1.1.485|B.1.1.487|B.1.1.5|B.1.1.50|B.1.1.506|B.1.1.507|B.1.1.513|B.1.1.514|B.1.1.515|B.1.1.516|B.1.1.518|B.1.1.52|B.1.1.53|B.1.1.54|B.1.1.57|B.1.1.61|B.1.1.62|B.1.1.67|B.1.1.70|B.1.1.71|B.1.1.77|B.1.1.8|B.1.1.87|B.1.1.88|B.1.1.89|B.1.1.98|B.1.1.99|B.1.103|B.1.104|B.1.106|B.1.108|B.1.110|B.1.110.1|B.1.110.3|B.1.111|B.1.112|B.1.113|B.1.115|B.1.116|B.1.118|B.1.119|B.1.12|B.1.124|B.1.13|B.1.131|B.1.134|B.1.137|B.1.139|B.1.142|B.1.143|B.1.145|B.1.146|B.1.147|B.1.149|B.1.151|B.1.153|B.1.157|B.1.160.32|B.1.161|B.1.162|B.1.163|B.1.164|B.1.166|B.1.167|B.1.169|B.1.170|B.1.177.31|B.1.178|B.1.179|B.1.180|B.1.181|B.1.182|B.1.184|B.1.187|B.1.188|B.1.189|B.1.190|B.1.192|B.1.194|B.1.195|B.1.199|B.1.2|B.1.201|B.1.203|B.1.206|B.1.208|B.1.210|B.1.211|B.1.212|B.1.213|B.1.214|B.1.215|B.1.218|B.1.22|B.1.22.1|B.1.220|B.1.223|B.1.224|B.1.225|B.1.227|B.1.229|B.1.23|B.1.232|B.1.233|B.1.234|B.1.236|B.1.237|B.1.239|B.1.241|B.1.245|B.1.247|B.1.250|B.1.252|B.1.263|B.1.264|B.1.264.1|B.1.265|B.1.267|B.1.268|B.1.270|B.1.273|B.1.274|B.1.276|B.1.277|B.1.279|B.1.280|B.1.281|B.1.284|B.1.285|B.1.291|B.1.293|B.1.294|B.1.298|B.1.301|B.1.302|B.1.304|B.1.306|B.1.308|B.1.309|B.1.310|B.1.311|B.1.314|B.1.319|B.1.320|B.1.321|B.1.323|B.1.324|B.1.328|B.1.329|B.1.330|B.1.332|B.1.333|B.1.334|B.1.335|B.1.336|B.1.337|B.1.338|B.1.340|B.1.341|B.1.342|B.1.343|B.1.344|B.1.346|B.1.348|B.1.349|B.1.354|B.1.355|B.1.356|B.1.357|B.1.358|B.1.359|B.1.36.19|B.1.36.22|B.1.360|B.1.361|B.1.362|B.1.362.2|B.1.363|B.1.369|B.1.369.1|B.1.37|B.1.371|B.1.377|B.1.378|B.1.379|B.1.38|B.1.380|B.1.381|B.1.382|B.1.383|B.1.384|B.1.385|B.1.387|B.1.39|B.1.390|B.1.391|B.1.395|B.1.397|B.1.399|B.1.400|B.1.401|B.1.403|B.1.408|B.1.409|B.1.411|B.1.413|B.1.415|B.1.416|B.1.416.1|B.1.417|B.1.422|B.1.423|B.1.424|B.1.425|B.1.428|B.1.432|B.1.433|B.1.434|B.1.435|B.1.436|B.1.437|B.1.438|B.1.439|B.1.441|B.1.442|B.1.443|B.1.444|B.1.445|B.1.446|B.1.450|B.1.451|B.1.452|B.1.453|B.1.456|B.1.459|B.1.466|B.1.467|B.1.469|B.1.470|B.1.475|B.1.478|B.1.479|B.1.482|B.1.483|B.1.485|B.1.486|B.1.487|B.1.492|B.1.493|B.1.494|B.1.495|B.1.496|B.1.497|B.1.498|B.1.499|B.1.500|B.1.501|B.1.502|B.1.503|B.1.504|B.1.505|B.1.506|B.1.507|B.1.508|B.1.509|B.1.510|B.1.511|B.1.513|B.1.516|B.1.518|B.1.520|B.1.521|B.1.523|B.1.527|B.1.528|B.1.530|B.1.535|B.1.537|B.1.538|B.1.539|B.1.540|B.1.541|B.1.544|B.1.545|B.1.548|B.1.555|B.1.556|B.1.558|B.1.560|B.1.564|B.1.565|B.1.566|B.1.567|B.1.568|B.1.569|B.1.571|B.1.573|B.1.574|B.1.576|B.1.577|B.1.578|B.1.580|B.1.581|B.1.582|B.1.585|B.1.586|B.1.589|B.1.590|B.1.592|B.1.593|B.1.595|B.1.595.3|B.1.595.4|B.1.596|B.1.596.1|B.1.597|B.1.598|B.1.6|B.1.600|B.1.601|B.1.604|B.1.609|B.1.611|B.1.612|B.1.613|B.1.615|B.1.67|B.1.76|B.1.78|B.1.83|B.1.84|B.1.9|B.1.9.3|B.1.9.4|B.1.9.5|B.1.93|B.1.94|B.1.96|B.4.8|D.4|D.5|K.1|K.2|L.2|N.1|N.3|N.4|N.6|N.8|S.1
CUY16-003538	Fast-S	A	A_1	A.18|AE.2|AE.4|AE.7|AE.8|AF.1|AG.1|AN.1|AP.1|AQ.1|B.1|B.1.1|B.1.1.10|B.1.1.101|B.1.1.110|B.1.1.111|B.1.1.113|B.1.1.12|B.1.1.121|B.1.1.127|B.1.1.128|B.1.1.129|B.1.1.132|B.1.1.133|B.1.1.134|B.1.1.135|B.1.1.139|B.1.1.148|B.1.1.152|B.1.1.157|B.1.1.158|B.1.1.159|B.1.1.162|B.1.1.163|B.1.1.164|B.1.1.166|B.1.1.169|B.1.1.171|B.1.1.172|B.1.1.174|B.1.1.176|B.1.1.177|B.1.1.181|B.1.1.182|B.1.1.184|B.1.1.185|B.1.1.187|B.1.1.189|B.1.1.192|B.1.1.198|B.1.1.200|B.1.1.201|B.1.1.204|B.1.1.205|B.1.1.209|B.1.1.210|B.1.1.214|B.1.1.216|B.1.1.217|B.1.1.218|B.1.1.219|B.1.1.225|B.1.1.226|B.1.1.229|B.1.1.230|B.1.1.231|B.1.1.232|B.1.1.236|B.1.1.243|B.1.1.244|B.1.1.25|B.1.1.253|B.1.1.254|B.1.1.257|B.1.1.258|B.1.1.26|B.1.1.261|B.1.1.265|B.1.1.266|B.1.1.267|B.1.1.268|B.1.1.27|B.1.1.271|B.1.1.274|B.1.1.277|B.1.1.282|B.1.1.283|B.1.1.284|B.1.1.285|B.1.1.286|B.1.1.288|B.1.1.290|B.1.1.291|B.1.1.297|B.1.1.301|B.1.1.304|B.1.1.305|B.1.1.306|B.1.1.307|B.1.1.308|B.1.1.31|B.1.1.315|B.1.1.317|B.1.1.319|B.1.1.323|B.1.1.324|B.1.1.325|B.1.1.326|B.1.1.328|B.1.1.329|B.1.1.33|B.1.1.331|B.1.1.334|B.1.1.335|B.1.1.34|B.1.1.342|B.1.1.344|B.1.1.346|B.1.1.347|B.1.1.348|B.1.1.350|B.1.1.352|B.1.1.354|B.1.1.355|B.1.1.356|B.1.1.357|B.1.1.359|B.1.1.366|B.1.1.367|B.1.1.368|B.1.1.370|B.1.1.373|B.1.1.376|B.1.1.378|B.1.1.381|B.1.1.382|B.1.1.383|B.1.1.386|B.1.1.387|B.1.1.388|B.1.1.39|B.1.1.391|B.1.1.392|B.1.1.398|B.1.1.40|B.1.1.401|B.1.1.402|B.1.1.404|B.1.1.405|B.1.1.406|B.1.1.407|B.1.1.409|B.1.1.410|B.1.1.411|B.1.1.414|B.1.1.416|B.1.1.419|B.1.1.422|B.1.1.424|B.1.1.426|B.1.1.427|B.1.1.430|B.1.1.431|B.1.1.432|B.1.1.434|B.1.1.435|B.1.1.436|B.1.1.437|B.1.1.438|B.1.1.44|B.1.1.440|B.1.1.441|B.1.1.442|B.1.1.447|B.1.1.448|B.1.1.450|B.1.1.452|B.1.1.453|B.1.1.456|B.1.1.458|B.1.1.459|B.1.1.46|B.1.1.465|B.1.1.466|B.1.1.47|B.1.1.48|B.1.1.481|B.1.1.482|B.1.1.484|B.1.1.485|B.1.1.487|B.1.1.5|B.1.1.50|B.1.1.506|B.1.1.507|B.1.1.513|B.1.1.514|B.1.1.515|B.1.1.516|B.1.1.518|B.1.1.52|B.1.1.53|B.1.1.54|B.1.1.57|B.1.1.61|B.1.1.62|B.1.1.67|B.1.1.70|B.1.1.71|B.1.1.77|B.1.1.8|B.1.1.87|B.1.1.88|B.1.1.89|B.1.1.98|B.1.1.99|B.1.103|B.1.104|B.1.106|B.1.108|B.1.110|B.1.110.1|B.1.110.3|B.1.111|B.1.112|B.1.113|B.1.115|B.1.116|B.1.118|B.1.119|B.1.12|B.1.124|B.1.13|B.1.131|B.1.134|B.1.137|B.1.139|B.1.142|B.1.143|B.1.145|B.1.146|B.1.147|B.1.149|B.1.151|B.1.153|B.1.157|B.1.160.32|B.1.161|B.1.162|B.1.163|B.1.164|B.1.166|B.1.167|B.1.169|B.1.170|B.1.177.31|B.1.178|B.1.179|B.1.180|B.1.181|B.1.182|B.1.184|B.1.187|B.1.188|B.1.189|B.1.190|B.1.192|B.1.194|B.1.195|B.1.199|B.1.2|B.1.201|B.1.203|B.1.206|B.1.208|B.1.210|B.1.211|B.1.212|B.1.213|B.1.214|B.1.215|B.1.218|B.1.22|B.1.22.1|B.1.220|B.1.223|B.1.224|B.1.225|B.1.227|B.1.229|B.1.23|B.1.232|B.1.233|B.1.234|B.1.236|B.1.237|B.1.239|B.1.241|B.1.245|B.1.247|B.1.250|B.1.252|B.1.263|B.1.264|B.1.264.1|B.1.265|B.1.267|B.1.268|B.1.270|B.1.273|B.1.274|B.1.276|B.1.277|B.1.279|B.1.280|B.1.281|B.1.284|B.1.285|B.1.291|B.1.293|B.1.294|B.1.298|B.1.301|B.1.302|B.1.304|B.1.306|B.1.308|B.1.309|B.1.310|B.1.311|B.1.314|B.1.319|B.1.320|B.1.321|B.1.323|B.1.324|B.1.328|B.1.329|B.1.330|B.1.332|B.1.333|B.1.334|B.1.335|B.1.336|B.1.337|B.1.338|B.1.340|B.1.341|B.1.342|B.1.343|B.1.344|B.1.346|B.1.348|B.1.349|B.1.354|B.1.355|B.1.356|B.1.357|B.1.358|B.1.359|B.1.36.19|B.1.36.22|B.1.360|B.1.361|B.1.362|B.1.362.2|B.1.363|B.1.369|B.1.369.1|B.1.37|B.1.371|B.1.377|B.1.378|B.1.379|B.1.38|B.1.380|B.1.381|B.1.382|B.1.383|B.1.384|B.1.385|B.1.387|B.1.39|B.1.390|B.1.391|B.1.395|B.1.397|B.1.399|B.1.400|B.1.401|B.1.403|B.1.408|B.1.409|B.1.411|B.1.413|B.1.415|B.1.416|B.1.416.1|B.1.417|B.1.422|B.1.423|B.1.424|B.1.425|B.1.428|B.1.432|B.1.433|B.1.434|B.1.435|B.1.436|B.1.437|B.1.438|B.1.439|B.1.441|B.1.442|B.1.443|B.1.444|B.1.445|B.1.446|B.1.450|B.1.451|B.1.452|B.1.453|B.1.456|B.1.459|B.1.466|B.1.467|B.1.469|B.1.470|B.1.475|B.1.478|B.1.479|B.1.482|B.1.483|B.1.485|B.1.486|B.1.487|B.1.492|B.1.493|B.1.494|B.1.495|B.1.496|B.1.497|B.1.498|B.1.499|B.1.500|B.1.501|B.1.502|B.1.503|B.1.504|B.1.505|B.1.506|B.1.507|B.1.508|B.1.509|B.1.510|B.1.511|B.1.513|B.1.516|B.1.518|B.1.520|B.1.521|B.1.523|B.1.527|B.1.528|B.1.530|B.1.535|B.1.537|B.1.538|B.1.539|B.1.540|B.1.541|B.1.544|B.1.545|B.1.548|B.1.555|B.1.556|B.1.558|B.1.560|B.1.564|B.1.565|B.1.566|B.1.567|B.1.568|B.1.569|B.1.571|B.1.573|B.1.574|B.1.576|B.1.577|B.1.578|B.1.580|B.1.581|B.1.582|B.1.585|B.1.586|B.1.589|B.1.590|B.1.592|B.1.593|B.1.595|B.1.595.3|B.1.595.4|B.1.596|B.1.596.1|B.1.597|B.1.598|B.1.6|B.1.600|B.1.601|B.1.604|B.1.609|B.1.611|B.1.612|B.1.613|B.1.615|B.1.67|B.1.76|B.1.78|B.1.83|B.1.84|B.1.9|B.1.9.3|B.1.9.4|B.1.9.5|B.1.93|B.1.94|B.1.96|B.4.8|D.4|D.5|K.1|K.2|L.2|N.1|N.3|N.4|N.6|N.8|S.1
CUY16-003792	Fast-S	B.1.617.2	B.1.617.2_7	AY.26|AY.65
CUY16-003800	Fast-S	B.1.351	B.1.351	B.1.351

<table>
<tr><td>CUY16-003801</td><td>Fast-S</td><td>A</td><td>A_1</td><td>A.18|AE.2|AE.4|AE.7|AE.8|AF.1|AG.1|AN.1|AP.1|AQ.1|B.1|B.1.1|B.1.1.10|B.1.1.101|B.1.1.110|B.1.1.111|B.1.1.113|B.1.1.12|B.1.1.121|B.1.1.127|B.1.1.128|B.1.1.129|B.1.1.132|B.1.1.133|B.1.1.134|B.1.1.135|B.1.1.139|B.1.1.148|B.1.1.152|B.1.1.157|B.1.1.158|B.1.1.159|B.1.1.162|B.1.1.163|B.1.1.164|B.1.1.166|B.1.1.169|B.1.1.171|B.1.1.172|B.1.1.174|B.1.1.176|B.1.1.177|B.1.1.181|B.1.1.182|B.1.1.184|B.1.1.185|B.1.1.187|B.1.1.189|B.1.1.192|B.1.1.198|B.1.1.200|B.1.1.201|B.1.1.204|B.1.1.205|B.1.1.209|B.1.1.210|B.1.1.214|B.1.1.216|B.1.1.217|B.1.1.218|B.1.1.219|B.1.1.225|B.1.1.226|B.1.1.229|B.1.1.230|B.1.1.231|B.1.1.232|B.1.1.236|B.1.1.243|B.1.1.244|B.1.1.25|B.1.1.253|B.1.1.254|B.1.1.257|B.1.1.258|B.1.1.26|B.1.1.261|B.1.1.265|B.1.1.266|B.1.1.267|B.1.1.268|B.1.1.27|B.1.1.271|B.1.1.274|B.1.1.277|B.1.1.282|B.1.1.283|B.1.1.284|B.1.1.285|B.1.1.286|B.1.1.288|B.1.1.290|B.1.1.291|B.1.1.297|B.1.1.301|B.1.1.304|B.1.1.305|B.1.1.306|B.1.1.307|B.1.1.308|B.1.1.31|B.1.1.315|B.1.1.317|B.1.1.319|B.1.1.323|B.1.1.324|B.1.1.325|B.1.1.326|B.1.1.328|B.1.1.329|B.1.1.33|B.1.1.331|B.1.1.334|B.1.1.335|B.1.1.34|B.1.1.342|B.1.1.344|B.1.1.346|B.1.1.347|B.1.1.348|B.1.1.350|B.1.1.352|B.1.1.354|B.1.1.355|B.1.1.356|B.1.1.357|B.1.1.359|B.1.1.366|B.1.1.367|B.1.1.368|B.1.1.370|B.1.1.373|B.1.1.376|B.1.1.378|B.1.1.381|B.1.1.382|B.1.1.383|B.1.1.386|B.1.1.387|B.1.1.388|B.1.1.39|B.1.1.391|B.1.1.392|B.1.1.398|B.1.1.40|B.1.1.401|B.1.1.402|B.1.1.404|B.1.1.405|B.1.1.406|B.1.1.407|B.1.1.409|B.1.1.410|B.1.1.411|B.1.1.414|B.1.1.416|B.1.1.419|B.1.1.422|B.1.1.424|B.1.1.426|B.1.1.427|B.1.1.430|B.1.1.431|B.1.1.432|B.1.1.434|B.1.1.435|B.1.1.436|B.1.1.437|B.1.1.438|B.1.1.44|B.1.1.440|B.1.1.441|B.1.1.442|B.1.1.447|B.1.1.448|B.1.1.450|B.1.1.452|B.1.1.453|B.1.1.456|B.1.1.458|B.1.1.459|B.1.1.46|B.1.1.465|B.1.1.466|B.1.1.47|B.1.1.48|B.1.1.481|B.1.1.482|B.1.1.484|B.1.1.485|B.1.1.487|B.1.1.5|B.1.1.50|B.1.1.506|B.1.1.507|B.1.1.513|B.1.1.514|B.1.1.515|B.1.1.516|B.1.1.518|B.1.1.52|B.1.1.53|B.1.1.54|B.1.1.57|B.1.1.61|B.1.1.62|B.1.1.67|B.1.1.70|B.1.1.71|B.1.1.77|B.1.1.8|B.1.1.87|B.1.1.88|B.1.1.89|B.1.1.98|B.1.1.99|B.1.103|B.1.104|B.1.106|B.1.108|B.1.110|B.1.110.1|B.1.110.3|B.1.111|B.1.112|B.1.113|B.1.115|B.1.116|B.1.118|B.1.119|B.1.12|B.1.124|B.1.13|B.1.131|B.1.134|B.1.137|B.1.139|B.1.142|B.1.143|B.1.145|B.1.146|B.1.147|B.1.149|B.1.151|B.1.153|B.1.157|B.1.160.32|B.1.161|B.1.162|B.1.163|B.1.164|B.1.166|B.1.167|B.1.169|B.1.170|B.1.177.31|B.1.178|B.1.179|B.1.180|B.1.181|B.1.182|B.1.184|B.1.187|B.1.188|B.1.189|B.1.190|B.1.192|B.1.194|B.1.195|B.1.199|B.1.2|B.1.201|B.1.203|B.1.206|B.1.208|B.1.210|B.1.211|B.1.212|B.1.213|B.1.214|B.1.215|B.1.218|B.1.22|B.1.22.1|B.1.220|B.1.223|B.1.224|B.1.225|B.1.227|B.1.229|B.1.23|B.1.232|B.1.233|B.1.234|B.1.236|B.1.237|B.1.239|B.1.241|B.1.245|B.1.247|B.1.250|B.1.252|B.1.263|B.1.264|B.1.264.1|B.1.265|B.1.267|B.1.268|B.1.270|B.1.273|B.1.274|B.1.276|B.1.277|B.1.279|B.1.280|B.1.281|B.1.284|B.1.285|B.1.291|B.1.293|B.1.294|B.1.298|B.1.301|B.1.302|B.1.304|B.1.306|B.1.308|B.1.309|B.1.310|B.1.311|B.1.314|B.1.319|B.1.320|B.1.321|B.1.323|B.1.324|B.1.328|B.1.329|B.1.330|B.1.332|B.1.333|B.1.334|B.1.335|B.1.336|B.1.337|B.1.338|B.1.340|B.1.341|B.1.342|B.1.343|B.1.344|B.1.346|B.1.348|B.1.349|B.1.354|B.1.355|B.1.356|B.1.357|B.1.358|B.1.359|B.1.36.19|B.1.36.22|B.1.360|B.1.361|B.1.362|B.1.362.2|B.1.363|B.1.369|B.1.369.1|B.1.37|B.1.371|B.1.377|B.1.378|B.1.379|B.1.38|B.1.380|B.1.381|B.1.382|B.1.383|B.1.384|B.1.385|B.1.387|B.1.39|B.1.390|B.1.391|B.1.395|B.1.397|B.1.399|B.1.400|B.1.401|B.1.403|B.1.408|B.1.409|B.1.411|B.1.413|B.1.415|B.1.416|B.1.416.1|B.1.417|B.1.422|B.1.423|B.1.424|B.1.425|B.1.428|B.1.432|B.1.433|B.1.434|B.1.435|B.1.436|B.1.437|B.1.438|B.1.439|B.1.441|B.1.442|B.1.443|B.1.444|B.1.445|B.1.446|B.1.450|B.1.451|B.1.452|B.1.453|B.1.456|B.1.459|B.1.466|B.1.467|B.1.469|B.1.470|B.1.475|B.1.478|B.1.479|B.1.482|B.1.483|B.1.485|B.1.486|B.1.487|B.1.492|B.1.493|B.1.494|B.1.495|B.1.496|B.1.497|B.1.498|B.1.499|B.1.500|B.1.501|B.1.502|B.1.503|B.1.504|B.1.505|B.1.506|B.1.507|B.1.508|B.1.509|B.1.510|B.1.511|B.1.513|B.1.516|B.1.518|B.1.520|B.1.521|B.1.523|B.1.527|B.1.528|B.1.530|B.1.535|B.1.537|B.1.538|B.1.539|B.1.540|B.1.541|B.1.544|B.1.545|B.1.548|B.1.555|B.1.556|B.1.558|B.1.560|B.1.564|B.1.565|B.1.566|B.1.567|B.1.568|B.1.569|B.1.571|B.1.573|B.1.574|B.1.576|B.1.577|B.1.578|B.1.580|B.1.581|B.1.582|B.1.585|B.1.586|B.1.589|B.1.590|B.1.592|B.1.593|B.1.595|B.1.595.3|B.1.595.4|B.1.596|B.1.596.1|B.1.597|B.1.598|B.1.6|B.1.600|B.1.601|B.1.604|B.1.609|B.1.611|B.1.612|B.1.613|B.1.615|B.1.67|B.1.76|B.1.78|B.1.83|B.1.84|B.1.9|B.1.9.3|B.1.9.4|B.1.9.5|B.1.93|B.1.94|B.1.96|B.4.8|D.4|D.5|K.1|K.2|L.2|N.1|N.3|N.4|N.6|N.8|S.1</td></tr>
<tr><td>CUY16-003802</td><td>Fast-S</td><td>B.1.351</td><td>B.1.351</td><td>B.1.351</td></tr>
<tr><td>CUY16-003803</td><td>Fast-S</td><td>B.1.351</td><td>B.1.351</td><td>B.1.351</td></tr>
<tr><td>CUY16-003804</td><td>Fast-S</td><td>A</td><td>A_23</td><td>B.1.1.345|B.1.1.393|B.1.400.1|N.9</td></tr>
<tr><td>CUY17-003849</td><td>Fast-S</td><td>B.1.1.7</td><td>B.1.1.7_1</td><td>B.1.1.7|Q.1|Q.3|Q.7</td></tr>
<tr><td>CUY17-003866</td><td>Fast-S</td><td>A</td><td>A_1</td><td>A.18|AE.2|AE.4|AE.7|AE.8|AF.1|AG.1|AN.1|AP.1|AQ.1|B.1|B.1.1|B.1.1.10|B.1.1.101|B.1.1.110|B.1.1.111|B.1.1.113|B.1.1.12|B.1.1.121|B.1.1.127|B.1.1.128|B.1.1.129|B.1.1.132|B.1.1.133|B.1.1.134|B.1.1.135|B.1.1.139|B.1.1.148|B.1.1.152|B.1.1.157|B.1.1.158|B.1.1.159|B.1.1.162|B.1.1.163|B.1.1.164|B.1.1.166|B.1.1.169|B.1.1.171|B.1.1.172|B.1.1.174|B.1.1.176|B.1.1.177|B.1.1.181|B.1.1.182|B.1.1.184|B.1.1.185|B.1.1.187|B.1.1.189|B.1.1.192|B.1.1.198|B.1.1.200|B.1.1.201|B.1.1.204|B.1.1.205|B.1.1.209|B.1.1.210|B.1.1.214|B.1.1.216|B.1.1.217|B.1.1.218|B.1.1.219|B.1.1.225|B.1.1.226|B.1.1.229|B.1.1.230|B.1.1.231|B.1.1.232|B.1.1.236|B.1.1.243|B.1.1.244|B.1.1.25|B.1.1.253|B.1.1.254|B.1.1.257|B.1.1.258|B.1.1.26|B.1.1.261|B.1.1.265|B.1.1.266|B.1.1.267|B.1.1.268|B.1.1.27|B.1.1.271|B.1.1.274|B.1.1.277|B.1.1.282|B.1.1.283|B.1.1.284|B.1.1.285|B.1.1.286|B.1.1.288|B.1.1.290|B.1.1.291|B.1.1.297|B.1.1.301|B.1.1.304|B.1.1.305|B.1.1.306|B.1.1.307|B.1.1.308|B.1.1.31|B.1.1.315|B.1.1.317|B.1.1.319|B.1.1.323|B.1.1.324|B.1.1.325|B.1.1.326|B.1.1.328|B.1.1.329|B.1.1.33|B.1.1.331|B.1.1.334|B.1.1.335|B.1.1.34|B.1.1.342|B.1.1.344|B.1.1.346|B.1.1.347|B.1.1.348|B.1.1.350|B.1.1.352|B.1.1.354|B.1.1.355|B.1.1.356|B.1.1.357|B.1.1.359|B.1.1.366|B.1.1.367|B.1.1.368|B.1.1.370|B.1.1.373|B.1.1.376|B.1.1.378|B.1.1.381|B.1.1.382|B.1.1.383|B.1.1.386|B.1.1.387|B.1.1.388|B.1.1.39|B.1.1.391|B.1.1.392|B.1.1.398|B.1.1.40|B.1.1.401|B.1.1.402|B.1.1.404|B.1.1.405|B.1.1.406|B.1.1.407|B.1.1.409|B.1.1.410|B.1.1.411|B.1.1.414|B.1.1.416|B.1.1.419|B.1.1.422|B.1.1.424|B.1.1.426|B.1.1.427|B.1.1.430|B.1.1.431|B.1.1.432|B.1.1.434|B.1.1.435|B.1.1.436|B.1.1.437|B.1.1.438|B.1.1.44|B.1.1.440|B.1.1.441|B.1.1.442|B.1.1.447|B.1.1.448|B.1.1.450|B.1.1.452|B.1.1.453|B.1.1.456|B.1.1.458|B.1.1.459|B.1.1.46|B.1.1.465|B.1.1.466|B.1.1.47|B.1.1.48|B.1.1.481|B.1.1.482|B.1.1.484|B.1.1.485|B.1.1.487|B.1.1.5|B.1.1.50|B.1.1.506|B.1.1.507|B.1.1.513|B.1.1.514|B.1.1.515|B.1.1.516|B.1.1.518|B.1.1.52|B.1.1.53|B.1.1.54|B.1.1.57|B.1.1.61|B.1.1.62|B.1.1.67|B.1.1.70|B.1.1.71|B.1.1.77|B.1.1.8|B.1.1.87|B.1.1.88|B.1.1.89|B.1.1.98|B.1.1.99|B.1.103|B.1.104|B.1.106|B.1.108|B.1.110|B.1.110.1|B.1.110.3|B.1.111|B.1.112|B.1.113|B.1.115|B.1.116|B.1.118|B.1.119|B.1.12|B.1.124|B.1.13|B.1.131|B.1.134|B.1.137|B.1.139|B.1.142|B.1.143|B.1.145|B.1.146|B.1.147|B.1.149|B.1.151|B.1.153|B.1.157|B.1.160.32|B.1.161|B.1.162|B.1.163|B.1.164|B.1.166|B.1.167|B.1.169|B.1.170|B.1.177.31|B.1.178|B.1.179|B.1.180|B.1.181|B.1.182|B.1.184|B.1.187|B.1.188|B.1.189|B.1.190|B.1.192|B.1.194|B.1.195|B.1.199|B.1.2|B.1.201|B.1.203|B.1.206|B.1.208|B.1.210|B.1.211|B.1.212|B.1.213|B.1.214|B.1.215|B.1.218|B.1.22|B.1.22.1|B.1.220|B.1.223|B.1.224|B.1.225|B.1.227|B.1.229|B.1.23|B.1.232|B.1.233|B.1.234|B.1.236|B.1.237|B.1.239|B.1.241|B.1.245|B.1.247|B.1.250|B.1.252|B.1.263|B.1.264|B.1.264.1|B.1.265|B.1.267|B.1.268|B.1.270|B.1.273|B.1.274|B.1.276|B.1.277|B.1.279|B.1.280|B.1.281|B.1.284|B.1.285|B.1.291|B.1.293|B.1.294|B.1.298|B.1.301|B.1.302|B.1.304|B.1.306|B.1.308|B.1.309|B.1.310|B.1.311|B.1.314|B.1.319|B.1.320|B.1.321|B.1.323|B.1.324|B.1.328|B.1.329|B.1.330|B.1.332|B.1.333|B.1.334|B.1.335|B.1.336|B.1.337|B.1.338|B.1.340|B.1.341|B.1.342|B.1.343|B.1.344|B.1.346|B.1.348|B.1.349|B.1.354|B.1.355|B.1.356|B.1.357|B.1.358|B.1.359|B.1.36.19|B.1.36.22|B.1.360|B.1.361|B.1.362|B.1.362.2|B.1.363|B.1.369|B.1.369.1|B.1.37|B.1.371|B.1.377|B.1.378|B.1.379|B.1.38|B.1.380|B.1.381|B.1.382|B.1.383|B.1.384|B.1.385|B.1.387|B.1.39|B.1.390|B.1.391|B.1.395|B.1.397|B.1.399|B.1.400|B.1.401|B.1.403|B.1.408|B.1.409|B.1.411|B.1.413|B.1.415|B.1.416|B.1.416.1|B.1.417|B.1.422|B.1.423|B.1.424|B.1.425|B.1.428|B.1.432|B.1.433|B.1.434|B.1.435|B.1.436|B.1.437|B.1.438|B.1.439|B.1.441|B.1.442|B.1.443|B.1.444|B.1.445|B.1.446|B.1.450|B.1.451|B.1.452|B.1.453|B.1.456|B.1.459|B.1.466|B.1.467|B.1.469|B.1.470|B.1.475|B.1.478|B.1.479|B.1.482|B.1.483|B.1.485|B.1.486|B.1.487|B.1.492|B.1.493|B.1.494|B.1.495|B.1.496|B.1.497|B.1.498|B.1.499|B.1.500|B.1.501|B.1.502|B.1.503|B.1.504|B.1.505|B.1.506|B.1.507|B.1.508|B.1.509|B.1.510|B.1.511|B.1.513|B.1.516|B.1.518|B.1.520|B.1.521|B.1.523|B.1.527|B.1.528|B.1.530|B.1.535|B.1.537|B.1.538|B.1.539|B.1.540|B.1.541|B.1.544|B.1.545|B.1.548|B.1.555|B.1.556|B.1.558|B.1.560|B.1.564|B.1.565|B.1.566|B.1.567|B.1.568|B.1.569|B.1.571|B.1.573|B.1.574|B.1.576|B.1.577|B.1.578|B.1.580|B.1.581|B.1.582|B.1.585|B.1.586|B.1.589|B.1.590|B.1.592|B.1.593|B.1.595|B.1.595.3|B.1.595.4|B.1.596|B.1.596.1|B.1.597|B.1.598|B.1.6|B.1.600|B.1.601|B.1.604|B.1.609|B.1.611|B.1.612|B.1.613|B.1.615|B.1.67|B.1.76|B.1.78|B.1.83|B.1.84|B.1.9|B.1.9.3|B.1.9.4|B.1.9.5|B.1.93|B.1.94|B.1.96|B.4.8|D.4|D.5|K.1|K.2|L.2|N.1|N.3|N.4|N.6|N.8|S.1</td></tr>
<tr><td>CUY17-003867</td><td>Fast-S</td><td>A</td><td>A_1</td><td>A.18|AE.2|AE.4|AE.7|AE.8|AF.1|AG.1|AN.1|AP.1|AQ.1|B.1|B.1.1|B.1.1.10|B.1.1.101|B.1.1.110|B.1.1.111|B.1.1.113|B.1.1.12|B.1.1.121|B.1.1.127|B.1.1.128|B.1.1.129|B.1.1.132|B.1.1.133|B.1.1.134|B.1.1.135|B.1.1.139|B.1.1.148|B.1.1.152|B.1.1.157|B.1.1.158|B.1.1.159|B.1.1.162|B.1.1.163|B.1.1.164|B.1.1.166|B.1.1.169|B.1.1.171|B.1.1.172|B.1.1.174|B.1.1.176|B.1.1.177|B.1.1.181|B.1.1.182|B.1.1.184|B.1.1.185|B.1.1.187|B.1.1.189|B.1.1.192|B.1.1.198|B.1.1.200|B.1.1.201|B.1.1.204|B.1.1.205|B.1.1.209|B.1.1.210|B.1.1.214|B.1.1.216|B.1.1.217|B.1.1.218|B.1.1.219|B.1.1.225|B.1.1.226|B.1.1.229|B.1.1.230|B.1.1.231|B.1.1.232|B.1.1.236|B.1.1.243|B.1.1.244|B.1.1.25|B.1.1.253|B.1.1.254|B.1.1.257|B.1.1.258|B.1.1.26|B.1.1.261|B.1.1.265|B.1.1.266|B.1.1.267|B.1.1.268|B.1.1.27|B.1.1.271|B.1.1.274|B.1.1.277|B.1.1.282|B.1.1.283|B.1.1.284|B.1.1.285|B.1.1.286|B.1.1.288|B.1.1.290|B.1.1.291|B.1.1.297|B.1.1.301|B.1.1.304|B.1.1.305|B.1.1.306|B.1.1.307|B.1.1.308|B.1.1.31|B.1.1.315|B.1.1.317|B.1.1.319|B.1.1.323|B.1.1.324|B.1.1.325|B.1.1.326|B.1.1.328|B.1.1.329|B.1.1.33|B.1.1.331|B.1.1.334|B.1.1.335|B.1.1.34|B.1.1.342|B.1.1.344|B.1.1.346|B.1.1.347|B.1.1.348|B.1.1.350|B.1.1.352|B.1.1.354|B.1.1.355|B.1.1.356|B.1.1.357|B.1.1.359|B.1.1.366|B.1.1.367|B.1.1.368|B.1.1.370|B.1.1.373|B.1.1.376|B.1.1.378|B.1.1.381|B.1.1.382|B.1.1.383|B.1.1.386|B.1.1.387|B.1.1.388|B.1.1.39|B.1.1.391|B.1.1.392|B.1.1.398|B.1.1.40|B.1.1.401|B.1.1.402|B.1.1.404|B.1.1.405|B.1.1.406|B.1.1.407|B.1.1.409|B.1.1.410|B.1.1.411|B.1.1.414|B.1.1.416|B.1.1.419|B.1.1.422|B.1.1.424|B.1.1.426|B.1.1.427|B.1.1.430|B.1.1.431|B.1.1.432|B.1.1.434|B.1.1.435|B.1.1.436|B.1.1.437|B.1.1.438|B.1.1.44|B.1.1.440|B.1.1.441|B.1.1.442|B.1.1.447|B.1.1.448|B.1.1.450|B.1.1.452|B.1.1.453|B.1.1.456|B.1.1.458|B.1.1.459|B.1.1.46|B.1.1.465|B.1.1.466|B.1.1.47|B.1.1.48|B.1.1.481|B.1.1.482|B.1.1.484|B.1.1.485|B.1.1.487|B.1.1.5|B.1.1.50|B.1.1.506|B.1.1.507|B.1.1.513|B.1.1.514|B.1.1.515|B.1.1.516|B.1.1.518|B.1.1.52|B.1.1.53|B.1.1.54|B.1.1.57|B.1.1.61|B.1.1.62|B.1.1.67|B.1.1.70|B.1.1.71|B.1.1.77|B.1.1.8|B.1.1.87|B.1.1.88|B.1.1.89|B.1.1.98|B.1.1.99|B.1.103|B.1.104|B.1.106|B.1.108|B.1.110|B.1.110.1|B.1.110.3|B.1.111|B.1.112|B.1.113|B.1.115|B.1.116|B.1.118|B.1.119|B.1.12|B.1.124|B.1.13|B.1.131|B.1.134|B.1.137|B.1.139|B.1.142|B.1.143|B.1.145|B.1.146|B.1.147|B.1.149|B.1.151|B.1.153|B.1.157|B.1.160.32|B.1.161|B.1.162|B.1.163|B.1.164|B.1.166|B.1.167|B.1.169|B.1.170|B.1.177.31|B.1.178|B.1.179|B.1.180|B.1.181|B.1.182|B.1.184|B.1.187|B.1.188|B.1.189|B.1.190|B.1.192|B.1.194|B.1.195|B.1.199|B.1.2|B.1.201|B.1.203|B.1.206|B.1.208|B.1.210|B.1.211|B.1.212|B.1.213|B.1.214|B.1.215|B.1.218|B.1.22|B.1.22.1|B.1.220|B.1.223|B.1.224|B.1.225|B.1.227|B.1.229|B.1.23|B.1.232|B.1.233|B.1.234|B.1.236|B.1.237|B.1.239|B.1.241|B.1.245|B.1.247|B.1.250|B.1.252|B.1.263|B.1.264|B.1.264.1|B.1.265|B.1.267|B.1.268|B.1.270|B.1.273|B.1.274|B.1.276|B.1.277|B.1.279|B.1.280|B.1.281|B.1.284|B.1.285|B.1.291|B.1.293|B.1.294|B.1.298|B.1.301|B.1.302|B.1.304|B.1.306|B.1.308|B.1.309|B.1.310|B.1.311|B.1.314|B.1.319|B.1.320|B.1.321|B.1.323|B.1.324|B.1.328|B.1.329|B.1.330|B.1.332|B.1.333|B.1.334|B.1.335|B.1.336|B.1.337|B.1.338|B.1.340|B.1.341|B.1.342|B.1.343|B.1.344|B.1.346|B.1.348|B.1.349|B.1.354|B.1.355|B.1.356|B.1.357|B.1.358|B.1.359|B.1.36.19|B.1.36.22|B.1.360|B.1.361|B.1.362|B.1.362.2|B.1.363|B.1.369|B.1.369.1|B.1.37|B.1.371|B.1.377|B.1.378|B.1.379|B.1.38|B.1.380|B.1.381|B.1.382|B.1.383|B.1.384|B.1.385|B.1.387|B.1.39|B.1.390|B.1.391|B.1.395|B.1.397|B.1.399|B.1.400|B.1.401|B.1.403|B.1.408|B.1.409|B.1.411|B.1.413|B.1.415|B.1.416|B.1.416.1|B.1.417|B.1.422|B.1.423|B.1.424|B.1.425|B.1.428|B.1.432|B.1.433|B.1.434|B.1.435|B.1.436|B.1.437|B.1.438|B.1.439|B.1.441|B.1.442|B.1.443|B.1.444|B.1.445|B.1.446|B.1.450|B.1.451|B.1.452|B.1.453|B.1.456|B.1.459|B.1.466|B.1.467|B.1.469|B.1.470|B.1.475|B.1.478|B.1.479|B.1.482|B.1.483|B.1.485|B.1.486|B.1.487|B.1.492|B.1.493|B.1.494|B.1.495|B.1.496|B.1.497|B.1.498|B.1.499|B.1.500|B.1.501|B.1.502|B.1.503|B.1.504|B.1.505|B.1.506|B.1.507|B.1.508|B.1.509|B.1.510|B.1.511|B.1.513|B.1.516|B.1.518|B.1.520|B.1.521|B.1.523|B.1.527|B.1.528|B.1.530|B.1.535|B.1.537|B.1.538|B.1.539|B.1.540|B.1.541|B.1.544|B.1.545|B.1.548|B.1.555|B.1.556|B.1.558|B.1.560|B.1.564|B.1.565|B.1.566|B.1.567|B.1.568|B.1.569|B.1.571|B.1.573|B.1.574|B.1.576|B.1.577|B.1.578|B.1.580|B.1.581|B.1.582|B.1.585|B.1.586|B.1.589|B.1.590|B.1.592|B.1.593|B.1.595|B.1.595.3|B.1.595.4|B.1.596|B.1.596.1|B.1.597|B.1.598|B.1.6|B.1.600|B.1.601|B.1.604|B.1.609|B.1.611|B.1.612|B.1.613|B.1.615|B.1.67|B.1.76|B.1.78|B.1.83|B.1.84|B.1.9|B.1.9.3|B.1.9.4|B.1.9.5|B.1.93|B.1.94|B.1.96|B.4.8|D.4|D.5|K.1|K.2|L.2|N.1|N.3|N.4|N.6|N.8|S.1</td></tr>
<tr><td>CUY17-003892</td><td>Fast-S</td><td>B.1.617.2</td><td>B.1.617.2_7</td><td>AY.26|AY.65</td></tr>
<tr><td>CUY17-003893</td><td>Fast-S</td><td>AY.48</td><td>AY.48</td><td>AY.48</td></tr>
<tr><td>CUY17-003901</td><td>Fast-S</td><td>B.1.617.2</td><td>B.1.617.2_2</td><td>AY.109|AY.122|AY.122.4|AY.13|AY.14|AY.15|AY.16|AY.16.1|AY.25|AY.3|AY.3.2|AY.43|AY.43.8|AY.43.9|AY.44|AY.46|AY.46.1|AY.46.6|AY.51|AY.54|AY.6|AY.7|AY.7.2|AY.76|AY.77|AY.79|AY.88|AY.91|AY.91.1|AY.98</td></tr>
<tr><td>CUY17-003909</td><td>Fast-S</td><td>B.1.617.2</td><td>B.1.617.2_2</td><td>AY.109|AY.122|AY.122.4|AY.13|AY.14|AY.15|AY.16|AY.16.1|AY.25|AY.3|AY.3.2|AY.43|AY.43.8|AY.43.9|AY.44|AY.46|AY.46.1|AY.46.6|AY.51|AY.54|AY.6|AY.7|AY.7.2|AY.76|AY.77|AY.79|AY.88|AY.91|AY.91.1|AY.98</td></tr>
<tr><td>CUY17-003910</td><td>Fast-S</td><td>B.1.617.2</td><td>B.1.617.2_2</td><td>AY.109|AY.122|AY.122.4|AY.13|AY.14|AY.15|AY.16|AY.16.1|AY.25|AY.3|AY.3.2|AY.43|AY.43.8|AY.43.9|AY.44|AY.46|AY.46.1|AY.46.6|AY.51|AY.54|AY.6|AY.7|AY.7.2|AY.76|AY.77|AY.79|AY.88|AY.91|AY.91.1|AY.98</td></tr>
<tr><td>CUY17-003939</td><td>Fast-S</td><td>P.1</td><td>P.1_1</td><td>P.1|P.1.1|P.1.10|P.1.10.1|P.1.12|P.1.13|P.1.15|P.1.16|P.1.2</td></tr>
<tr><td>CUY18-004019</td><td>Fast-S</td><td>AY.33.2</td><td>AY.33.2</td><td>AY.33.2</td></tr>
<tr><td>CUY18-004022</td><td>Fast-S</td><td>AY.33.2</td><td>AY.33.2</td><td>AY.33.2</td></tr>
<tr><td>CUY18-004023</td><td>Fast-S</td><td>B.1.617.2</td><td>B.1.617.2_2</td><td>AY.109|AY.122|AY.122.4|AY.13|AY.14|AY.15|AY.16|AY.16.1|AY.25|AY.3|AY.3.2|AY.43|AY.43.8|AY.43.9|AY.44|AY.46|AY.46.1|AY.46.6|AY.51|AY.54|AY.6|AY.7|AY.7.2|AY.76|AY.77|AY.79|AY.88|AY.91|AY.91.1|AY.98</td></tr>
<tr><td>CUY18-004026</td><td>Fast-S</td><td>AY.30</td><td>AY.30</td><td>AY.30</td></tr>
</table>

CUY19-004183	Fast-S	C.37	C.37	C.37
CUY21-004441	Fast-S	B.1.621	B.1.621	B.1.621
CUY23-004816	Fast-S	AY.20	AY.20	AY.20
CUY24-004876	Fast-S	B.1.617.2	B.1.617.2_4	AY.122.5|AY.45|AY.46.3|AY.84|AY.99|AY.99.1|AY.99.2
CUY24-004954	Fast-S	A	A_11	AY.17|AY.25.1|AY.25.1.2|AY.46.5|AY.7.1|AY.80|AY.83|AY.93|B.1.617.2
CUY26-005235	Fast-S	B.1.617.2	B.1.617.2_3	AY.37|AY.55|AY.57|AY.58|AY.59|AY.60|AY.61|AY.62|AY.66|AY.67|AY.72|AY.73
CUY42-007286	Fast-S	BA.2.12	BA.2.12	BA.2.12
CUY42-007288	Fast-S	BA.1.15	BA.1.15	BA.1.15
CUY42-007289	Fast-S	BA.1	BA.1_2	BA.1.1.15|BA.1.10|BA.1.13|BA.1.14|BA.1.17|BA.1.6
CUY42-007291	Fast-S	BA.2.12	BA.2.12	BA.2.12
CUY42-007294	Fast-S	BA.1.1	BA.1.1_2	BA.1.1|BA.1.1.18

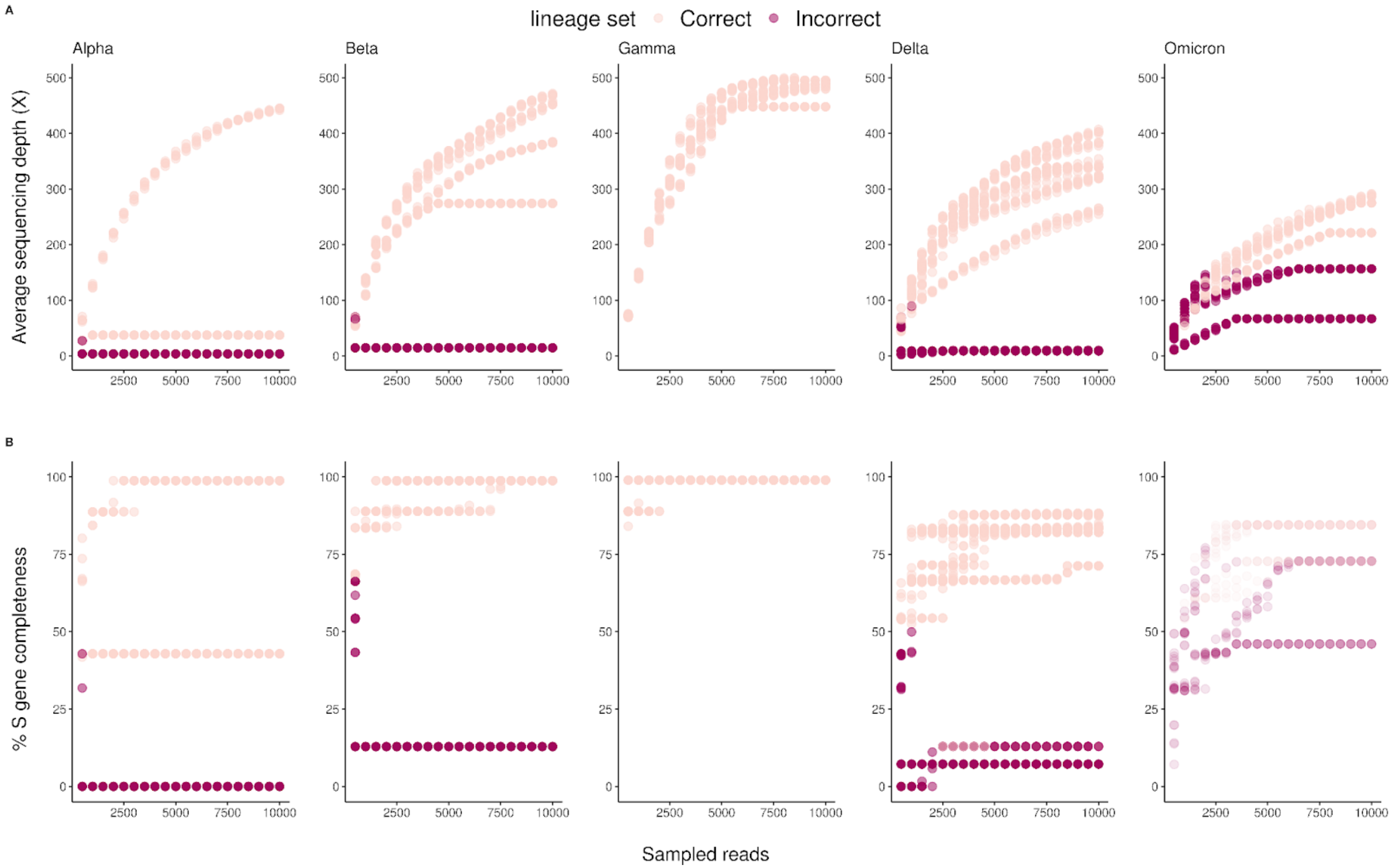

Figure S1: STANDARD-S classification status of consensus S genes generated using a set of random subsampling of reads for each VOC sample. A) Average sequencing depth (X) vs sampled reads. B) % of S gene completeness. Incorrect classifications were detected at lower sequencing depth and gene completeness.

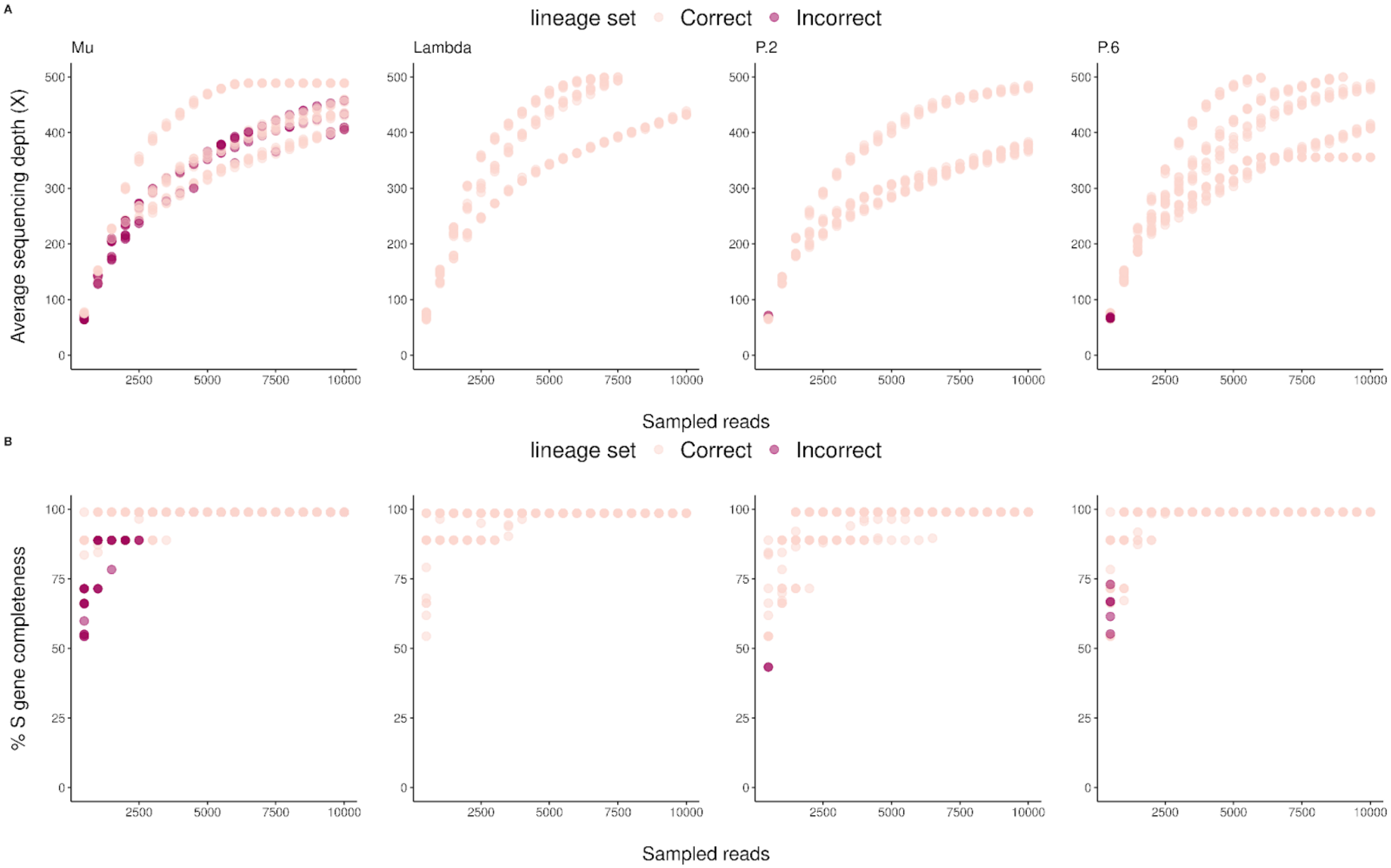

Figure S2: STANDARD-S classification status of consensus S genes generated using a set of random subsampling of reads for each VOI and non VOC/VOI samples (P.2 and P.6). **A)** Average sequencing depth (X) vs sampled reads. **B)** % of S gene completeness. Correct classifications were obtained in a wide range of sequencing depth and S gene completeness for most of the samples.

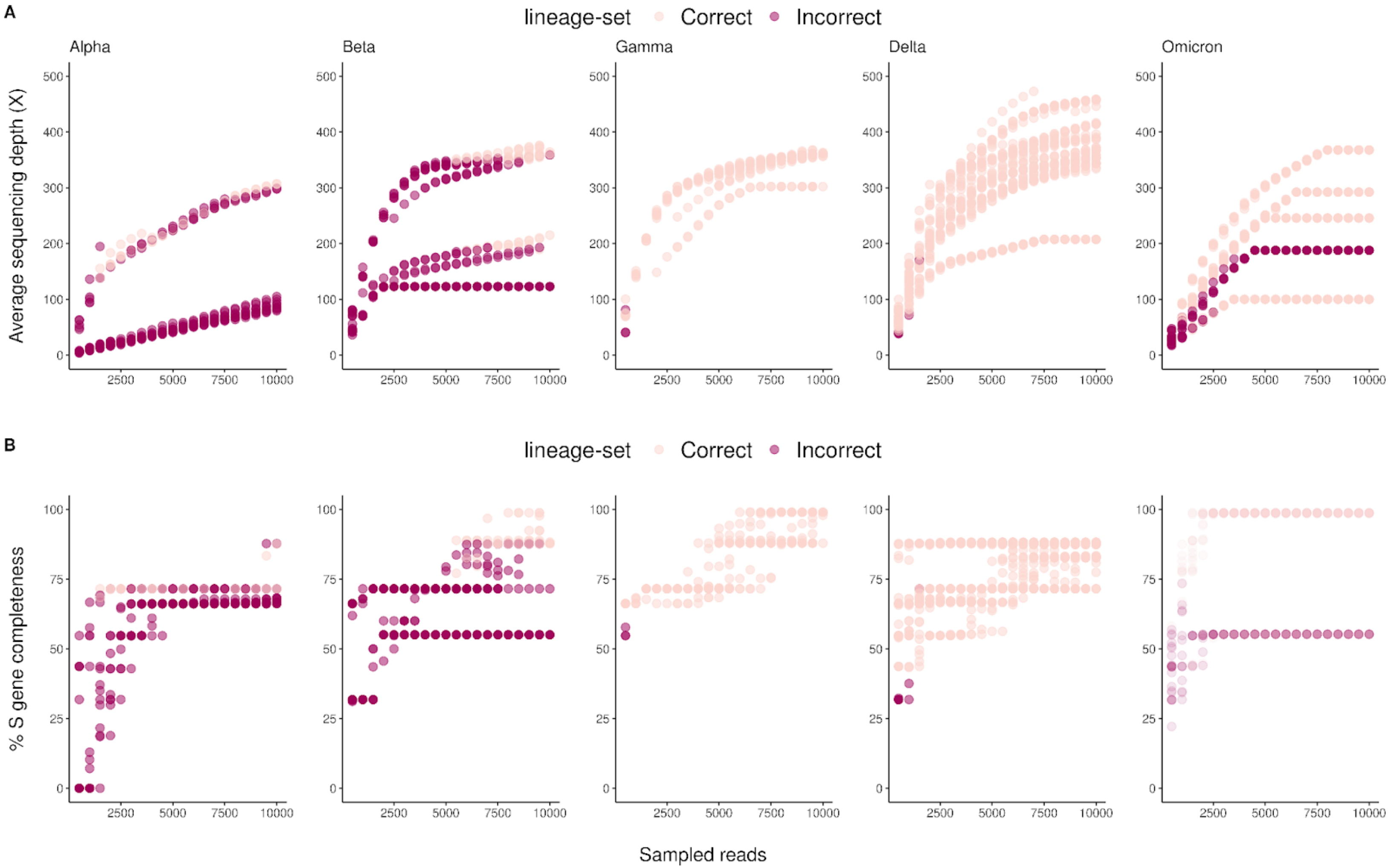

Figure S3: FAST-S classification status of consensus S genes generated using a set of random subsampling of reads for each VOC sample. A) Average sequencing depth (X) vs sampled reads. B) % of S gene completeness.

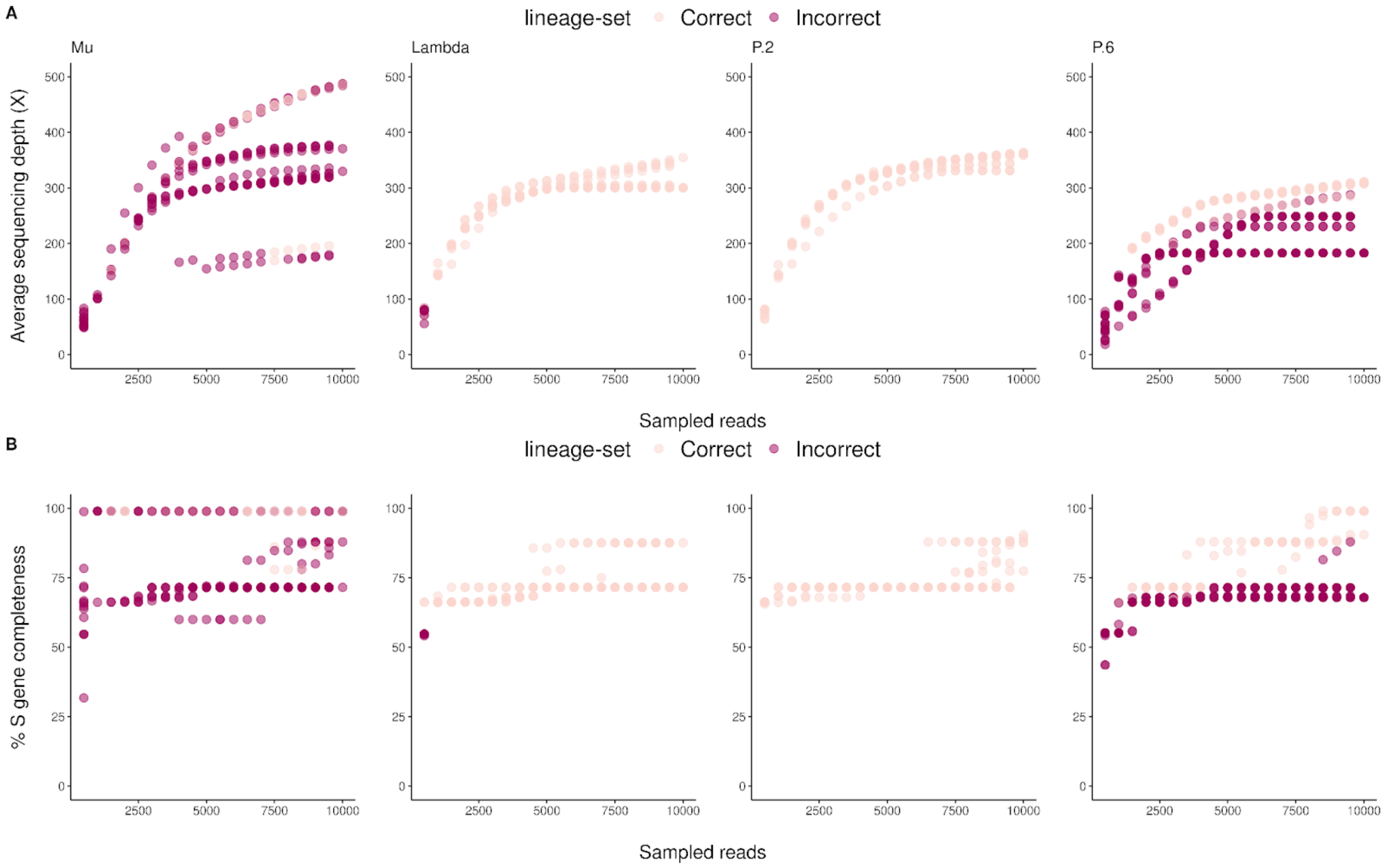

Figure S4: FAST-S classification status of consensus S genes generated using a set of random subsampling of reads for each VOI and non VOC/VOI samples (P.2 and P.6). A) Average sequencing depth (X) vs sampled reads. B) % of S gene completeness.

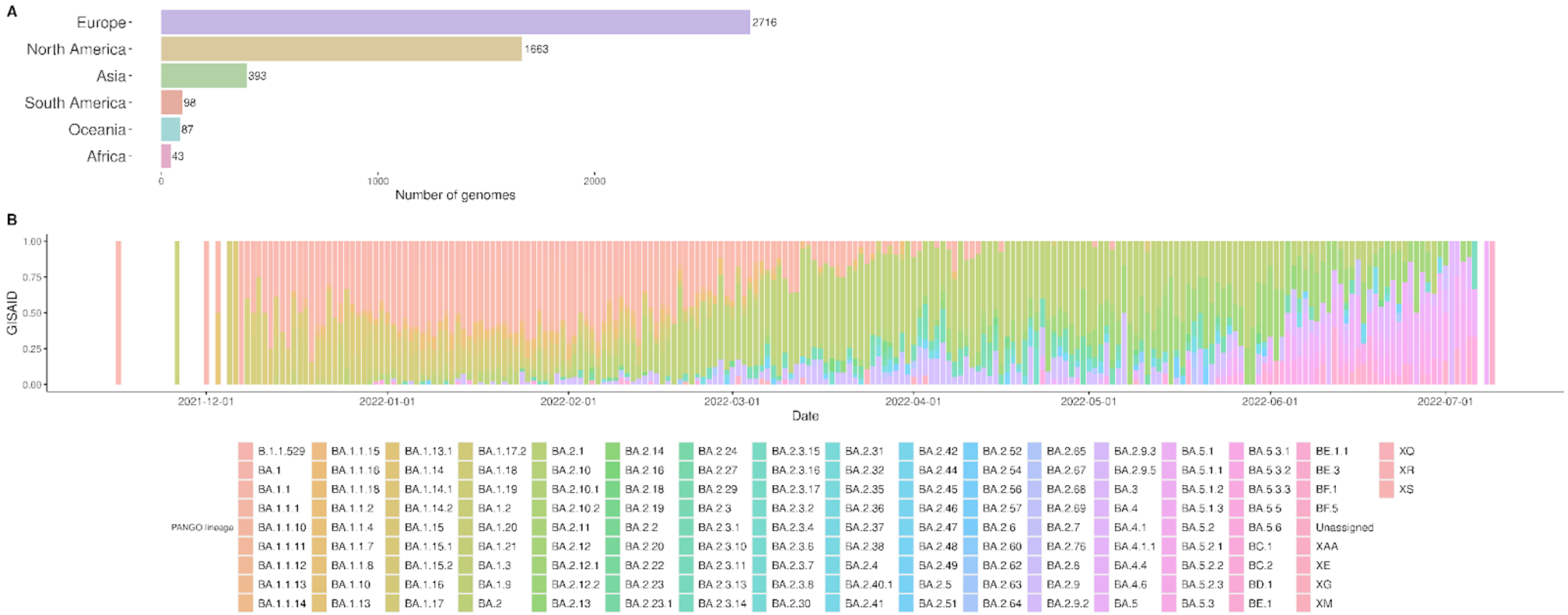

Figure S5: Omicron VOC subsampled genomes (n = 5000) from EpiCoV/GISAID (EPI_SET_220929wd). **A)** Subsample representation across continents. **B)** Relative abundance of the subsampled Omicron VOC genomes over time.

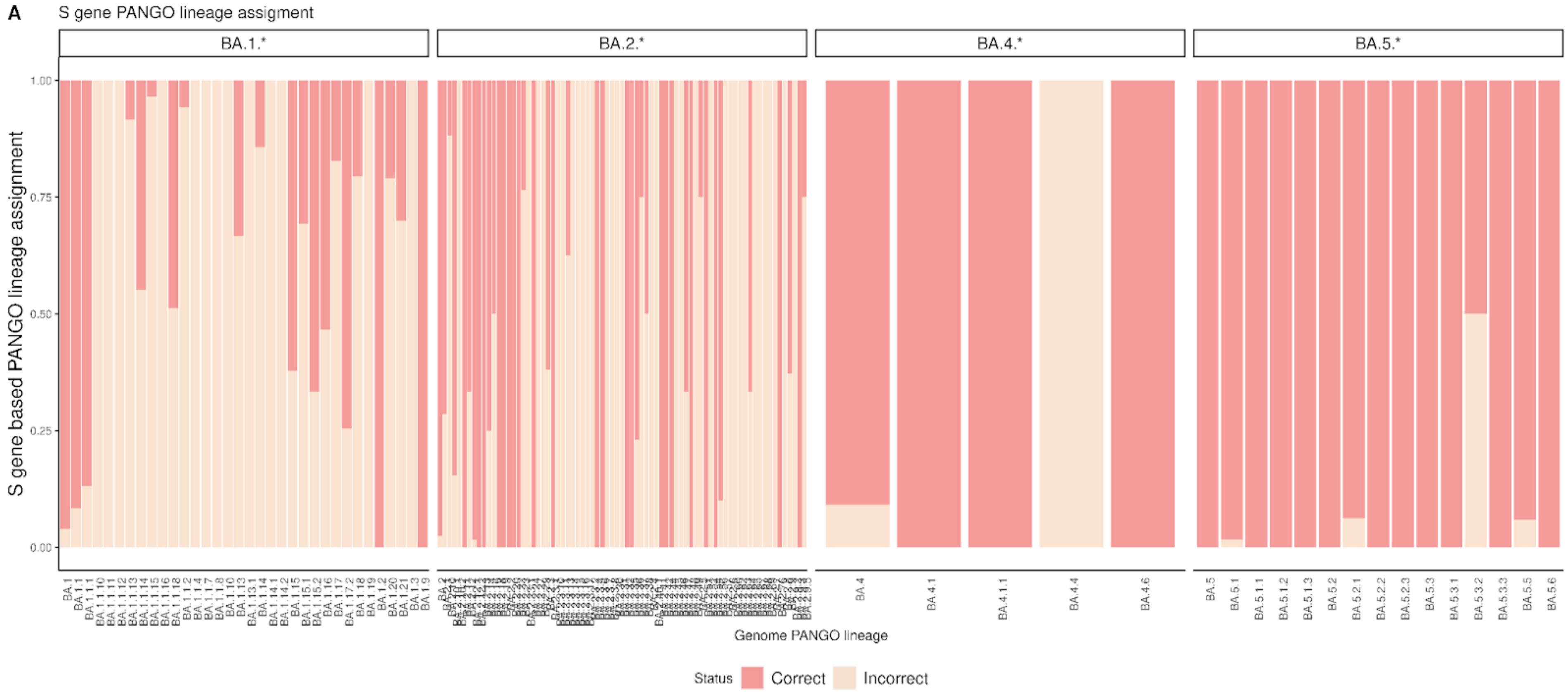

Figure S6: Genome lineage and S gene lineage assignment matches of the Omicron dataset (EPI_SET_220929wd). Correct and incorrect match between genome PANGO lineage and S gene lineage set of BA.1.*, BA.2.*, BA.4.* and BA.5.* samples. Correct assignments were considered when the genome lineages were found in the range of PANGO lineages of a specific lineage set. If no matches were found in the range of PANGO lineages, the assignment was considered incorrect. Correct assignments have a mean S gene completeness of 97.2% ± 5.1 and incorrect assignments 94.2% ± 8.6%. In addition, 75% of BA.1.*, 85% of BA.2.* S gene sequences, 95% of BA.4.* and 97% of BA.5.* samples matched correctly the genome PANGO lineage.

Table S5: Omicron daataset lineage set

GISAID_accession	GISAID_Pango.lineage	hedgehog	hedgehog_set_description	sequence_id	S gene completeness (%)
EPI_ISL_10001512	BA.2	BA.2	BA.2_1	QEUH-3632394|EPI_ISL_10001512|United Kingdom|BA.2|2022-02-08	99,81
EPI_ISL_10002547	BA.1.17.2	BA.1.17.2	BA.1.17.2	SG-CLM-02074563|EPI_ISL_10002547|Switzerland|BA.1.17.2|2022-02-07	99,64
EPI_ISL_10002861	BA.2	BA.2	BA.2_1	QEUH-36232D5|EPI_ISL_10002861|United Kingdom|BA.2|2022-02-09	99,81
EPI_ISL_10003971	BA.1.1.15	BA.1.1	BA.1.1_3	QEUH-3641ECE|EPI_ISL_10003971|United Kingdom|BA.1.1.15|2022-02-09	99,64
EPI_ISL_10004833	BA.1.18	BA.1	BA.1_3	PAC-CERBAHC-01834581|EPI_ISL_10004833|France|BA.1.18|2022-01-24	96,62
EPI_ISL_10005917	BA.1	BA.1.1	BA.1.1_3	RP-RKI-I-517514|EPI_ISL_10005917|Germany|BA.1|2022-02-01	89,87
EPI_ISL_10006551	BA.1.1	BA.1.1	BA.1.1_3	NW-RKI-I-518084|EPI_ISL_10006551|Germany|BA.1.1|2022-01-25	99,58
EPI_ISL_10006646	BA.1.1	BA.1.15	BA.1.15	NW-RKI-I-518188|EPI_ISL_10006646|Germany|BA.1.1|2022-01-25	87,92
EPI_ISL_10007413	BA.1.17	BA.1	BA.1_4	BB-RKI-I-520276|EPI_ISL_10007413|Germany|BA.1.17|2022-01-31	99,64
EPI_ISL_10008696	BA.1.18	BA.1.15	BA.1.15	BW-RKI-I-521048|EPI_ISL_10008696|Germany|BA.1.18|2022-01-28	84,20
EPI_ISL_10013280	XG	BA.2	BA.2_1	DCGC-369198|EPI_ISL_10013280|Denmark|XG|2022-02-05	99,81
EPI_ISL_10014423	BA.2.9	BA.2	BA.2_1	DCGC-369547|EPI_ISL_10014423|Denmark|BA.2.9|2022-02-09	99,79
EPI_ISL_10015893	BA.1.1	BA.1	BA.1_4	Jessa_11-2207-001449|EPI_ISL_10015893|Belgium|BA.1.1|2022-02-14	97,31
EPI_ISL_10018470	BA.2	BA.2	BA.2_1	NW-RKI-I-526185|EPI_ISL_10018470|Germany|BA.2|2022-02-03	99,81
EPI_ISL_10018834	BA.2	A	A_14	HE-RKI-I-526345|EPI_ISL_10018834|Germany|BA.2|2022-01-27	98,00
EPI_ISL_10020550	BA.1.1.1	BA.1.1	BA.1.1_3	BY-RKI-I-527509|EPI_ISL_10020550|Germany|BA.1.1.1|2022-01-21	99,64
EPI_ISL_10025085	BA.1.1.1	BA.1.1	BA.1.1_3	SN-RKI-I-533293|EPI_ISL_10025085|Germany|BA.1.1.1|2022-02-02	99,60
EPI_ISL_10025768	BA.1	BA.1.15	BA.1.15	NW-RKI-I-534817|EPI_ISL_10025768|Germany|BA.1|2022-02-01	81,63
EPI_ISL_10026169	BA.1.1	BA.1.1	BA.1.1_2	HE-RKI-I-536523|EPI_ISL_10026169|Germany|BA.1.1|2022-02-03	99,62
EPI_ISL_10026425	BA.1	BA.1.1	BA.1.1_3	SH-RKI-I-536785|EPI_ISL_10026425|Germany|BA.1|2022-01-31	95,54
EPI_ISL_10028525	BA.1.1	BA.1.1	BA.1.1_3	NW-RKI-I-539949|EPI_ISL_10028525|Germany|BA.1.1|2022-02-08	89,62
EPI_ISL_10029915	BA.1.1	BA.1.1	BA.1.1_2	AZ-ASU44146|EPI_ISL_10029915|USA|BA.1.1|2022-02-11	95,94
EPI_ISL_10030258	BA.1	BA.1	BA.1_3	TH-RKI-I-541383|EPI_ISL_10030258|Germany|BA.1|2022-01-18	99,54
EPI_ISL_10030403	BA.1.1	BA.1	BA.1_4	CO-CDC-MMB13653960|EPI_ISL_10030403|USA|BA.1.1|2022-01-19	83,13
EPI_ISL_10030783	BA.1.1.18	BA.1	BA.1_4	CO-CDC-MMB13808400|EPI_ISL_10030783|USA|BA.1.1.18|2022-01-23	97,10
EPI_ISL_10032058	BA.1.1	BA.1.1	BA.1.1_2	NW-RKI-I-542545|EPI_ISL_10032058|Germany|BA.1.1|2022-02-03	85,92
EPI_ISL_10032829	BA.1.1	BA.1.1	BA.1.1_2	CA-CDC-FG-266963|EPI_ISL_10032829|USA|BA.1.1|2022-02-06	95,40
EPI_ISL_10038087	BA.1	BA.1.1.16	BA.1.1.16	IMBA_IMP-1503_A01|EPI_ISL_10038087|Austria|BA.1|2022-01-14	49,80
EPI_ISL_10040049	BA.1.1.18	BA.1.1	BA.1.1_2	CA-CDC-FG-266985|EPI_ISL_10040049|USA|BA.1.1.18|2022-02-07	99,52
EPI_ISL_10040120	BA.1	BA.1.15	BA.1.15	CA-CDC-FG-266914|EPI_ISL_10040120|USA|BA.1|2022-02-07	97,86
EPI_ISL_10041119	BA.1.15	BA.1.15	BA.1.15	UT-CDC-LC0527208|EPI_ISL_10041119|USA|BA.1.15|2022-01-27	99,64
EPI_ISL_10041526	BA.1.1	BA.1.1	BA.1.1_2	NM-CDC-LC0527826|EPI_ISL_10041526|USA|BA.1.1|2022-02-01	99,64
EPI_ISL_10043912	BA.1.1	BA.1.1	BA.1.1_2	NV-CDC-ASC210693062|EPI_ISL_10043912|USA|BA.1.1|2022-02-04	96,78
EPI_ISL_10046066	BA.1.1	BA.1.1	BA.1.1_2	KDCA29012|EPI_ISL_10046066|South Korea|BA.1.1|2022-01-25	99,52
EPI_ISL_10046954	BA.1.1.1	BA.1.1	BA.1.1_2	PAC-IHU-63101_Nova1|EPI_ISL_10046954|France|BA.1.1.1|2022-01	96,01
EPI_ISL_10048115	BA.1	BA.1	BA.1_4	ALDP-36927F0|EPI_ISL_10048115|United Kingdom|BA.1|2022-02-11	99,64
EPI_ISL_10049971	BA.1	BA.1	BA.1_4	MILK-3696C57|EPI_ISL_10049971|United Kingdom|BA.1|2022-02-12	99,64
EPI_ISL_10050895	BA.1.15	BA.1	BA.1_1	VT-CDCBI-CRSP_UP3Y7MB3AP4PC64O|EPI_ISL_10050895|USA|BA.1.15|2022-02-08	83,15
EPI_ISL_10052424	BA.1	BA.1	BA.1_4	MILK-369C653|EPI_ISL_10052424|United Kingdom|BA.1|2022-02-12	99,64
EPI_ISL_10052449	BA.2.3	A	A_14	ALDP-369AFF0|EPI_ISL_10052449|United Kingdom|BA.2.3|2022-02-11	99,81
EPI_ISL_10052489	BA.2.1	A	A_14	LSPA-369B672|EPI_ISL_10052489|United Kingdom|BA.2.1|2022-02-11	99,81
EPI_ISL_10053384	BA.1.15.1	BA.1.15.1	BA.1.15.1	MILK-369CE6A|EPI_ISL_10053384|United Kingdom|BA.1.15.1|2022-02-12	99,64
EPI_ISL_10053577	BA.2	A	A_14	ALDP-369F33B|EPI_ISL_10053577|United Kingdom|BA.2|2022-02-11	99,81
EPI_ISL_10053933	BC.1	BA.1.1	BA.1.1_2	RIMD05064|EPI_ISL_10053933|Japan|BC.1|2022-01-15	99,64
EPI_ISL_10056138	BA.1.1	BA.1	BA.1_4	PHEC-5R04EZ2D|EPI_ISL_10056138|United Kingdom|BA.1.1|2022	83,95
EPI_ISL_10057207	BA.1.15	BA.1.15	BA.1.15	LSPA-3677DE0|EPI_ISL_10057207|United Kingdom|BA.1.15|2022-02-10	99,64
EPI_ISL_10059257	BA.1.1.15	BA.1.1	BA.1.1_3	LSPA-368572D|EPI_ISL_10059257|United Kingdom|BA.1.1.15|2022-02-09	99,64
EPI_ISL_10059473	BA.1	BA.1.15	BA.1.15	PHEC-YYNQEY4|EPI_ISL_10059473|United Kingdom|BA.1|2022-01-24	85,75
EPI_ISL_10059974	BA.1	BA.1	BA.1_4	LSPA-3669525|EPI_ISL_10059974|United Kingdom|BA.1|2022-02-08	99,64
EPI_ISL_10060989	BD.1	BD.1	BD.1	LSPA-366880F|EPI_ISL_10060989|United Kingdom|BD.1|2022-02-10	99,64
EPI_ISL_10061571	BA.2	A	A_17	DCGC-371155|EPI_ISL_10061571|Denmark|BA.2|2022-02-12	99,81
EPI_ISL_10062635	BA.2	A	A_17	DCGC-371774|EPI_ISL_10062635|Denmark|BA.2|2022-02-10	99,79
EPI_ISL_10063815	BA.2.9	A	A_17	DCGC-372211|EPI_ISL_10063815|Denmark|BA.2.9|2022-02-12	99,79
EPI_ISL_10064040	BA.2	A	A_17	DCGC-372392|EPI_ISL_10064040|Denmark|BA.2|2022-02-12	99,81
EPI_ISL_10064453	BA.2.9	BA.2	BA.2_1	DCGC-372804|EPI_ISL_10064453|Denmark|BA.2.9|2022-02-10	89,56
EPI_ISL_10066792	BA.1.1	BA.1	BA.1_4	OR-OSPHL04990|EPI_ISL_10066792|USA|BA.1.1|2022-02-07	84,05
EPI_ISL_10069830	BA.1.15	BA.1	BA.1_3	AL-FIOCRUZ-IAM7519|EPI_ISL_10069830|Brazil|BA.1.15|2022-01-06	90,99
EPI_ISL_10071149	BA.1	BA.1	BA.1_3	CA-TCPHL-020122-142|EPI_ISL_10071149|USA|BA.1|2022-01-23	96,03
EPI_ISL_10071404	BA.1.1.15	BA.1.1	BA.1.1_3	BRE-IPP05942|EPI_ISL_10071404|France|BA.1.1.15|2022-01-24	99,64
EPI_ISL_10071458	BA.1.1.1	BA.1.1	BA.1.1_3	BRE-IPP08272|EPI_ISL_10071458|France|BA.1.1.1|2022-01-31	99,64
EPI_ISL_10072222	BA.1	BA.1.1	BA.1.1_2	ZH-EMC-5054|EPI_ISL_10072222|Netherlands|BA.1|2022-02-06	95,95
EPI_ISL_10074349	BA.1.1	BA.1.1	BA.1.1_2	GA-CDC-LC0529442|EPI_ISL_10074349|USA|BA.1.1|2022-02-03	99,64
EPI_ISL_10075020	BA.1.1	BA.1.1	BA.1.1_2	MN-CDC-IBX537250476919|EPI_ISL_10075020|USA|BA.1.1|2022-02-06	96,68
EPI_ISL_10075902	BA.1.1.18	BA.1.1	BA.1.1_3	TN-ASC-210543829|EPI_ISL_10075902|USA|BA.1.1.18|2022-01-24	97,06
EPI_ISL_10075903	BA.1.15	BA.1.15	BA.1.15	TN-ASC-210543831|EPI_ISL_10075903|USA|BA.1.15|2022-01-24	99,64
EPI_ISL_10077987	BA.1	A	A_2	TN-ASC-210578358|EPI_ISL_10077987|USA|BA.1|2021-12-31	97,00
EPI_ISL_10079295	BA.1.1	BA.1.1	BA.1.1_2	MA-21965|EPI_ISL_10079295|Chile|BA.1.1|2022-01-31	99,47
EPI_ISL_10079340	BA.1.1	BA.1.1	BA.1.1_2	TA-22231|EPI_ISL_10079340|Chile|BA.1.1|2022-02-02	99,52
EPI_ISL_10079778	BA.2	A	A_17	09_SE100_22CS101838|EPI_ISL_10079778|Sweden|BA.2|2022-02-08	99,81
EPI_ISL_10080141	BA.1.20	BA.1.15	BA.1.15	DC-DFS-PHL-05556|EPI_ISL_10080141|USA|BA.1.20|2022-01-10	75,27
EPI_ISL_10081715	BA.1.1	BA.1.1	BA.1.1_2	TN-ASC-210577619|EPI_ISL_10081715|USA|BA.1.1|2021-12-29	97,48
EPI_ISL_10082275	BA.1.15	BA.1.15	BA.1.15	WI-ASC-210586840|EPI_ISL_10082275|USA|BA.1.15|2022-01-07	99,64
EPI_ISL_10082686	BA.1.1	BA.1.1	BA.1.1_2	ID-IBL-791116|EPI_ISL_10082686|USA|BA.1.1|2022-01-28	99,60
EPI_ISL_10082962	BA.1.15	BA.1.15	BA.1.15	TN-ASC-210503968|EPI_ISL_10082962|USA|BA.1.15|2022-01-03	96,91
EPI_ISL_10083202	BA.1	BA.1	BA.1_4	TN-ASC-210591399|EPI_ISL_10083202|USA|BA.1|2022-01-08	98,45
EPI_ISL_10085619	BA.1.1	BA.1.1	BA.1.1_2	MA-CDCBI-CRSP_FALSACPQJ62TZ3GR|EPI_ISL_10085619|USA|BA.1.1|2022-02-09	94,54
EPI_ISL_10085978	BA.1.1	BA.1	BA.1_1	NY-CDCBI-CRSP_JEOQM4ZB62MFX5PU|EPI_ISL_10085978|USA|BA.1.1|2022-02-08	94,52
EPI_ISL_10086107	BA.1.1	BA.1.1	BA.1.1_3	CA-HLX-STM-9K5P8E4UH|EPI_ISL_10086107|USA|BA.1.1|2022-01-26	78,55
EPI_ISL_10086324	BA.1.1	BA.1.1	BA.1.1_2	CA-HLX-STM-P9U8CRV2U|EPI_ISL_10086324|USA|BA.1.1|2022-01-28	99,64
EPI_ISL_10087736	BA.1.1.18	BA.1.1	BA.1.1_3	NM-UNM-ED01314|EPI_ISL_10087736|USA|BA.1.1.18|2022-01-10	99,64
EPI_ISL_10088241	BA.1	BA.1	BA.1_2	PZH-UMB-15698|EPI_ISL_10088241|Poland|BA.1|2022-02-04	99,64
EPI_ISL_10088877	BA.1.15	BA.1.15	BA.1.15	CO-CDPHE-2102748567|EPI_ISL_10088877|USA|BA.1.15|2022-01-15	99,64
EPI_ISL_10089378	BA.1.1	BA.1.1	BA.1.1_2	CO-CDPHE-2102773243|EPI_ISL_10089378|USA|BA.1.1|2022-01-13	99,64
EPI_ISL_10089947	BA.1.1.18	BA.1.1	BA.1.1_2	CO-CDPHE-2102781686|EPI_ISL_10089947|USA|BA.1.1.18|2022-01-25	90,94
EPI_ISL_10090454	BA.1.1	BA.1.1	BA.1.1_2	CO-CDPHE-2102773103|EPI_ISL_10090454|USA|BA.1.1|2022-01-13	99,64
EPI_ISL_10090476	BA.1.15	BA.1.15	BA.1.15	CO-CDPHE-2102773008|EPI_ISL_10090476|USA|BA.1.15|2022-01-13	99,64
EPI_ISL_10092612	BA.1.1	BA.1.1	BA.1.1_2	UT-UPHL-220216273164|EPI_ISL_10092612|USA|BA.1.1|2022-01-03	99,64
EPI_ISL_10094456	BA.2	BA.2	BA.2_1	DCGC-375443|EPI_ISL_10094456|Denmark|BA.2|2022-02-13	99,81
EPI_ISL_10094787	BA.2.9	A	A_17	DCGC-375776|EPI_ISL_10094787|Denmark|BA.2.9|2022-02-11	99,98
EPI_ISL_10095863	BA.2.9	A	A_17	DCGC-376458|EPI_ISL_10095863|Denmark|BA.2.9|2022-02-13	99,81
EPI_ISL_10096006	BA.1.17	BA.1.17.2	BA.1.17.2	DMSc-07556|EPI_ISL_10096006|Thailand|BA.1.17|2022-02-03	99,64
EPI_ISL_10100575	BA.1.1.1	BA.1.1	BA.1.1_3	OCC-HCL722000608501|EPI_ISL_10100575|France|BA.1.1.1|2022-02-07	99,64
EPI_ISL_10103966	BA.1.20	BA.1	BA.1_3	BCN_InDRE_FB6015_E02313832845_S11326|EPI_ISL_10103966|Mexico|BA.1.20|2022-01-11	99,64
EPI_ISL_10104089	BA.1.1	BA.1.1	BA.1.1_2	CMX_InDRE_FB7768_E_S11621|EPI_ISL_10104089|Mexico|BA.1.1|2022-01-21	99,64
EPI_ISL_10105582	BA.1.1	BA.1.1	BA.1.1_2	ZAC_LANGEBIO_IMSS_5519|EPI_ISL_10105582|Mexico|BA.1.1|2022-01-12	99,64
EPI_ISL_10105733	BA.2	BA.2	BA.2_1	LSPA-36E2C6B|EPI_ISL_10105733|United Kingdom|BA.2|2022-02-12	94,07
EPI_ISL_10106960	BA.1.1	BA.1.1	BA.1.1_2	MILK-36A19DA|EPI_ISL_10106960|United Kingdom|BA.1.1|2022-02-12	99,64
EPI_ISL_10107484	BA.2	A	A_14	ALDP-36AF5BA|EPI_ISL_10107484|United Kingdom|BA.2|2022-02-11	99,81
EPI_ISL_10108137	BA.1	BA.1.15	BA.1.15	ALDP-36D2132|EPI_ISL_10108137|United Kingdom|BA.1|2022-02-12	99,64

EPI_ISL_10108920	BA.1.1	BA.1.1	BA.1.1_2	ALDP-36C4230|EPI_ISL_10108920|United Kingdom|BA.1.1|2022-02-12	99,64
EPI_ISL_10112961	BA.1.17.2	BA.1.17.2	BA.1.17.2	QEUH-3684F80|EPI_ISL_10112961|United Kingdom|BA.1.17.2|2022-02-11	99,64
EPI_ISL_10113381	BA.1.1	BA.1.1	BA.1.1_3	QEUH-368A904|EPI_ISL_10113381|United Kingdom|BA.1.1|2022-02-11	99,64
EPI_ISL_10113531	BA.1.1	BA.1.1	BA.1.1_3	QEUH-36810CC|EPI_ISL_10113531|United Kingdom|BA.1.1|2022-02-11	99,64
EPI_ISL_10113657	BA.2	A	A_14	QEUH-3681B8F|EPI_ISL_10113657|United Kingdom|BA.2|2022-02-11	99,81
EPI_ISL_10114066	BA.1.1	BA.1.1	BA.1.1_2	FL-CDC-STM-DCZUVT9KV|EPI_ISL_10114066|USA|BA.1.1|2022-02-06	99,64
EPI_ISL_10114251	BA.1.1	BA.1.1	BA.1.1_3	FL-CDC-STM-25V8WBK58|EPI_ISL_10114251|USA|BA.1.1|2022-02-06	99,64
EPI_ISL_10115174	BA.1.17.2	BA.1.17.2	BA.1.17.2	PZH-GUM-11821|EPI_ISL_10115174|Poland|BA.1.17.2|2022	99,64
EPI_ISL_10115323	BA.1.1.1	BA.1.1	BA.1.1_2	PZH-GUM-12263|EPI_ISL_10115323|Poland|BA.1.1.1|2022-01-28	99,60
EPI_ISL_10115737	BA.1	BA.1	BA.1_4	WSSEGorzow-22S1255|EPI_ISL_10115737|Poland|BA.1|2022-02-07	97,20
EPI_ISL_10115945	BA.2.2	BA.2.2	BA.2.2	CH22011047R|EPI_ISL_10115945|Hong Kong|BA.2.2|2022-01-24	94,09
EPI_ISL_10118558	BA.2	BA.2	BA.2_1	ALDP-3711D9C|EPI_ISL_10118558|United Kingdom|BA.2|2022-02-14	99,81
EPI_ISL_10119348	BA.1.1	BA.1.1	BA.1.1_3	TKYkbm2288|EPI_ISL_10119348|Japan|BA.1.1|2022-01-13	99,64
EPI_ISL_10120830	BA.1.1	BA.1.1	BA.1.1_2	ALDP-36EF299|EPI_ISL_10120830|United Kingdom|BA.1.1|2022-02-13	99,64
EPI_ISL_10124640	BA.2	A	A_14	LOND-YYBRCS6|EPI_ISL_10124640|United Kingdom|BA.2|2022-02-02	99,81
EPI_ISL_10126239	BA.1.14	BA.1	BA.1_3	SP-IB_SEQ46648|EPI_ISL_10126239|Brazil|BA.1.14|2022-02-08	99,64
EPI_ISL_10126737	BA.1.1	BA.1.15	BA.1.15	ZNA_25148911|EPI_ISL_10126737|Belgium|BA.1.1|2022-01-31	91,76
EPI_ISL_10127074	BA.1.17.2	BA.1.15	BA.1.15	FNHK-Ps-003405|EPI_ISL_10127074|Czech Republic|BA.1.17.2|2022-02-08	94,09
EPI_ISL_10127423	BA.1.1.1	BA.1.1	BA.1.1_2	CT-HCB-1866|EPI_ISL_10127423|Spain|BA.1.1.1|2022-02-09	94,68
EPI_ISL_10129298	BA.1	BA.1.15	BA.1.15	328138|EPI_ISL_10129298|Greece|BA.1|2022-01-23	99,64
EPI_ISL_10129397	BA.1.1.1	BA.1	BA.1_2	327891|EPI_ISL_10129397|Greece|BA.1.1.1|2022-01-22	98,45
EPI_ISL_10132642	BA.1.1.1	BA.1	BA.1_1	NAT-22-15592|EPI_ISL_10132642|Czech Republic|BA.1.1.1|2022-02-07	95,02
EPI_ISL_10136738	BA.1	BA.1.15	BA.1.15	NIC_BKK_SEQ4139|EPI_ISL_10136738|Thailand|BA.1|2022-01	98,26
EPI_ISL_10137753	BA.1.1	BA.1.1	BA.1.1_2	TN-ASC-210686298|EPI_ISL_10137753|USA|BA.1.1|2022-01-26	99,64
EPI_ISL_10138812	BA.1.1.18	BA.1.1	BA.1.1_2	TN-ASC-210548987|EPI_ISL_10138812|USA|BA.1.1.18|2022-02-08	96,85
EPI_ISL_10146179	BA.1.1	BA.1.1	BA.1.1_2	CA-CDC-FG-270049|EPI_ISL_10146179|USA|BA.1.1|2022-02-07	96,57
EPI_ISL_10146571	BA.1.1.2	BA.1.1	BA.1.1_2	CA-CDC-FG-269370|EPI_ISL_10146571|USA|BA.1.1.2|2022-02-08	95,63
EPI_ISL_10152381	BA.1.18	BA.1.15	BA.1.15	01_SE100_22CS501946|EPI_ISL_10152381|Sweden|BA.1.18|2022-02-10	94,37
EPI_ISL_10157619	BA.1.1	BA.1.1	BA.1.1_2	TN-ASC-210543939|EPI_ISL_10157619|USA|BA.1.1|2022-01-24	99,64
EPI_ISL_10174523	BA.1.15	BA.1	BA.1_3	NIRE-017ff5|EPI_ISL_10174523|United Kingdom|BA.1.15|2022-01-03	96,43
EPI_ISL_10176344	BA.1.1.1	BA.1	BA.1_4	WSSEGorzow-22S1308|EPI_ISL_10176344|Poland|BA.1.1.1|2022-02-08	98,30
EPI_ISL_10177378	BA.1	BA.1	BA.1_3	NIRE-018df5|EPI_ISL_10177378|United Kingdom|BA.1|2022-01-17	96,03
EPI_ISL_10181255	BA.1.1.15	BA.1.1	BA.1.1_3	NORT-YNBZJ5C|EPI_ISL_10181255|United Kingdom|BA.1.1.15|2022	99,64
EPI_ISL_10181432	BA.2	A	A_17	NORT-YNBZRXK|EPI_ISL_10181432|United Kingdom|BA.2|2022	99,81
EPI_ISL_10181916	BA.1.17.2	BA.1.15	BA.1.15	PHEC-5R0A9Z62|EPI_ISL_10181916|United Kingdom|BA.1.17.2|2022	94,83
EPI_ISL_10182107	BA.1.1.15	BA.1.1	BA.1.1_3	PHEC-5R0B5Z7C|EPI_ISL_10182107|United Kingdom|BA.1.1.15|2022	99,64
EPI_ISL_10182878	BA.1.17.2	BA.1.17.2	BA.1.17.2	PHEC-5S079Z29|EPI_ISL_10182878|United Kingdom|BA.1.17.2|2022	99,96
EPI_ISL_10183657	BA.1.15	BA.1.15	BA.1.15	PHEC-5T039Z78|EPI_ISL_10183657|United Kingdom|BA.1.15|2022	80,73
EPI_ISL_10185299	BA.1.1.14	BA.1.1	BA.1.1_3	PHEP-YYRGDGB|EPI_ISL_10185299|United Kingdom|BA.1.1.14|2022-01-12	84,49
EPI_ISL_10186360	BA.1.1	BA.1.1	BA.1.1_2	PHWC-PEN6MG|EPI_ISL_10186360|United Kingdom|BA.1.1|2022-01-28	99,64
EPI_ISL_10186513	BA.1.1	BA.1.1	BA.1.1_2	PHWC-PENCA7|EPI_ISL_10186513|United Kingdom|BA.1.1|2022-01-30	99,52
EPI_ISL_10192381	BA.1	BA.1	BA.1_4	MILK-37096C9|EPI_ISL_10192381|United Kingdom|BA.1|2022-02-15	99,64
EPI_ISL_10193218	BA.2	A	A_14	QEUH-3725612|EPI_ISL_10193218|United Kingdom|BA.2|2022-02-15	99,81
EPI_ISL_10193357	BA.2	A	A_14	NORT-YNB341J|EPI_ISL_10193357|United Kingdom|BA.2|2022	99,81
EPI_ISL_10195716	BA.1.17.2	BA.1.17.2	BA.1.17.2	ALDP-3718982|EPI_ISL_10195716|United Kingdom|BA.1.17.2|2022-02-14	99,58
EPI_ISL_10196955	BA.1	BA.1.15	BA.1.15	NORT-YNBZBKZ|EPI_ISL_10196955|United Kingdom|BA.1|2022	99,64
EPI_ISL_10197636	BA.1	BA.1	BA.1_4	9297|EPI_ISL_10197636|Poland|BA.1|2022-02-22	97,20
EPI_ISL_10201808	BA.1	BA.1	BA.1_1	22SNR1561_wsserze|EPI_ISL_10201808|Poland|BA.1|2022-02-05	88,84
EPI_ISL_10202381	BA.2	A	A_17	DCGC-377570|EPI_ISL_10202381|Denmark|BA.2|2022-02-12	99,81
EPI_ISL_10203860	BA.2.9	A	A_17	DCGC-378112|EPI_ISL_10203860|Denmark|BA.2.9|2022-02-15	99,81
EPI_ISL_10204883	BA.2	BA.2	BA.2_1	DCGC-378775|EPI_ISL_10204883|Denmark|BA.2|2022-02-14	99,96
EPI_ISL_10205742	BA.2	BA.2	BA.2_1	DCGC-379129|EPI_ISL_10205742|Denmark|BA.2|2022-02-14	99,81
EPI_ISL_10207863	BA.2.9	BA.2	BA.2_1	DCGC-380107|EPI_ISL_10207863|Denmark|BA.2.9|2022-02-15	99,81
EPI_ISL_10210650	BA.1.1	BA.1.1	BA.1.1_3	NICD-N28332|EPI_ISL_10210650|South Africa|BA.1.1|2022-01-05	99,62
EPI_ISL_10210651	BA.1.17.2	BA.1	BA.1_2	NICD-N28324|EPI_ISL_10210651|South Africa|BA.1.17.2|2022-01-06	99,62
EPI_ISL_10213495	BA.1.1	BA.1.1	BA.1.1_2	TN-ASC-210684784|EPI_ISL_10213495|USA|BA.1.1|2022-01-25	99,64
EPI_ISL_10214270	BA.1.1	BA.1.1	BA.1.1_2	TN-ASC-210646074|EPI_ISL_10214270|USA|BA.1.1|2022-01-27	96,87
EPI_ISL_10214965	BA.1.1.18	BA.1.1	BA.1.1_3	TN-ASC-210646535|EPI_ISL_10214965|USA|BA.1.1.18|2022-01-29	87,56
EPI_ISL_10216669	BA.2.9	A	A_17	DCGC-382449|EPI_ISL_10216669|Denmark|BA.2.9|2022-02-16	99,81
EPI_ISL_10216783	BA.2	BA.2	BA.2_1	DCGC-382564|EPI_ISL_10216783|Denmark|BA.2|2022-02-11	99,81
EPI_ISL_10217925	BA.2	A	A_17	DCGC-383300|EPI_ISL_10217925|Denmark|BA.2|2022-02-15	99,81
EPI_ISL_10217953	BA.2	BA.2	BA.2_1	DCGC-383328|EPI_ISL_10217953|Denmark|BA.2|2022-01-22	96,01
EPI_ISL_10220373	BA.1.1	BA.1.1	BA.1.1_3	BFC-HMN-22022080543|EPI_ISL_10220373|France|BA.1.1|2022-01-24	98,23
EPI_ISL_10220754	BA.1.1	BA.1	BA.1_4	GES-HMN-22022030744|EPI_ISL_10220754|France|BA.1.1|2022-01-11	92,65
EPI_ISL_10221274	BA.1.1.18	BA.1.1	BA.1.1_2	TN-ASC-210537625|EPI_ISL_10221274|USA|BA.1.1.18|2022-01-18	99,50
EPI_ISL_10221320	BA.1.15	BA.1	BA.1_4	TN-ASC-210537680|EPI_ISL_10221320|USA|BA.1.15|2022-01-18	99,64
EPI_ISL_10221940	BA.1.1	BA.1.1	BA.1.1_2	NC-CDC-ASC210691402|EPI_ISL_10221940|USA|BA.1.1|2022-02-02	99,64
EPI_ISL_10222119	BA.1.1	BA.1.1	BA.1.1_2	NY-CDC-ASC210692735|EPI_ISL_10222119|USA|BA.1.1|2022-02-02	96,85
EPI_ISL_10223273	BA.1.1	BA.1.1	BA.1.1_2	SK-RRPL-344961|EPI_ISL_10223273|Canada|BA.1.1|2022-01-28	99,64
EPI_ISL_10224184	BA.1.1	BA.1.1	BA.1.1_2	FL-CDC-STM-6HS3KAV6S|EPI_ISL_10224184|USA|BA.1.1|2022-02-02	99,64
EPI_ISL_10225415	BA.1.1	BA.1.1	BA.1.1_2	NV-NSPHL-22-00039096|EPI_ISL_10225415|USA|BA.1.1|2022-02-14	99,62
EPI_ISL_10226781	BA.1	BA.1	BA.1_4	RI-CDC-2-5590029|EPI_ISL_10226781|USA|BA.1|2022-01-28	99,64
EPI_ISL_10227949	BA.1.1	BA.1.1	BA.1.1_2	CA-CDC-ASC210547358|EPI_ISL_10227949|USA|BA.1.1|2022-02-05	99,64
EPI_ISL_10231435	BA.1.1.8	BA.1.1	BA.1.1_3	NC-CDC-MMB13995073|EPI_ISL_10231435|USA|BA.1.1.8|2022-01-28	98,19
EPI_ISL_10231943	BA.1	BA.1	BA.1_4	WSSEGorzow-22S1345|EPI_ISL_10231943|Poland|BA.1|2022-02-06	93,34
EPI_ISL_10232698	BA.1.20	BA.1.15	BA.1.15	CA-CDC-FG-272324|EPI_ISL_10232698|USA|BA.1.20|2022-02-10	93,68
EPI_ISL_10233367	BA.1.1	BA.1.1	BA.1.1_2	CA-CDC-FG-272017|EPI_ISL_10233367|USA|BA.1.1|2022-02-12	91,26
EPI_ISL_10234534	BA.1.20	BA.1.15	BA.1.15	MN-CDC-QDX33539930|EPI_ISL_10234534|USA|BA.1.20|2022-01-29	99,64
EPI_ISL_10235875	BA.1	BA.1.1	BA.1.1_2	BC-BCCDC-347150|EPI_ISL_10235875|Canada|BA.1|2022-01-23	99,29
EPI_ISL_10235979	BA.1.1	BA.1.1	BA.1.1_2	MI-CDC-QDX33539972|EPI_ISL_10235979|USA|BA.1.1|2022-01-31	99,64
EPI_ISL_10237184	BA.1.1	BA.1.1	BA.1.1_2	NJ-CDC-QDX33591743|EPI_ISL_10237184|USA|BA.1.1|2022-02-05	99,64
EPI_ISL_10237788	BA.1.1	BA.1.1	BA.1.1_2	PA-CDC-MMB13947428|EPI_ISL_10237788|USA|BA.1.1|2022-01-27	97,20
EPI_ISL_10237891	BA.1.18	BA.1	BA.1_4	GA-CDC-MMB13957268|EPI_ISL_10237891|USA|BA.1.18|2022-01-27	97,20
EPI_ISL_10239413	BA.1.1	BA.1.1	BA.1.1_2	MA-MASPHL-09806|EPI_ISL_10239413|USA|BA.1.1|2022-01-07	99,05
EPI_ISL_10240293	BA.2.2	BA.2.2	BA.2.2	VM22015702|EPI_ISL_10240293|Hong Kong|BA.2.2|2022-02-10	99,81
EPI_ISL_10240795	BA.1.1	BA.1.1	BA.1.1_2	UT-UPHL-220218192812|EPI_ISL_10240795|USA|BA.1.1|2022-01-31	99,64
EPI_ISL_10243438	BA.1.1.15	BA.1.1	BA.1.1_3	MILK-3733A60|EPI_ISL_10243438|United Kingdom|BA.1.1.15|2022-02-16	99,64
EPI_ISL_10244616	BA.2	BA.2	BA.2_1	MILK-372C174|EPI_ISL_10244616|United Kingdom|BA.2|2022-02-16	99,81
EPI_ISL_10245425	BA.2	A	A_14	MILK-3728CFF|EPI_ISL_10245425|United Kingdom|BA.2|2022-02-16	99,81
EPI_ISL_10246220	BA.1	BA.1	BA.1_4	MILK-36FE604|EPI_ISL_10246220|United Kingdom|BA.1|2022-02-14	99,64
EPI_ISL_10246584	BA.2.10	A	A_14	QEUH-36D1072|EPI_ISL_10246584|United Kingdom|BA.2.10|2022-02-12	99,81
EPI_ISL_10247787	BA.1.1	BA.1.1	BA.1.1_3	PHEC-5T0A1Z97|EPI_ISL_10247787|United Kingdom|BA.1.1|2022	91,81
EPI_ISL_10248074	BA.1.18	BA.1.15	BA.1.15	BFC-HMN-22022140104|EPI_ISL_10248074|France|BA.1.18|2022-02-07	92,98
EPI_ISL_10248188	BA.2	BA.2	BA.2_1	GES-HMN-22022160669|EPI_ISL_10248188|France|BA.2|2022-02-14	99,81
EPI_ISL_10250086	BA.1	BA.1.15	BA.1.15	CO-CDPHE-2102782787|EPI_ISL_10250086|USA|BA.1|2022-01-26	69,53
EPI_ISL_10251467	BA.1.1	A	A_1	VS-UV-355|EPI_ISL_10251467|Chile|BA.1.1|2022-01-09	58,73
EPI_ISL_10253640	BA.1.1	BA.1.1	BA.1.1_2	CO-CDPHE-2102901543|EPI_ISL_10253640|USA|BA.1.1|2022-01-17	88,38
EPI_ISL_10254205	BA.1.1	BA.1.1	BA.1.1_2	PZH-UMB-16035|EPI_ISL_10254205|Poland|BA.1.1|2022-02-10	99,64
EPI_ISL_10258899	BA.1.18	BA.1	BA.1_3	DCGC-384547|EPI_ISL_10258899|Denmark|BA.1.18|2022-02-17	99,62
EPI_ISL_10261714	BA.1.17	BA.1	BA.1_3	QLD38326|EPI_ISL_10261714|Australia|BA.1.17|2022-01-13	99,60
EPI_ISL_10263785	BA.1.17	BA.1	BA.1_2	QLD37925|EPI_ISL_10263785|Australia|BA.1.17|2022-01-08	99,50

EPI_ISL_10266684	BA.1	A	A_1	B30b42_SN-IR2-0051281|EPI_ISL_10266684|Senegal|BA.1|2022-02-05	45,79
EPI_ISL_10266708	BA.1.1	BA.1.1	BA.1.1_2	CHUNamur14049532|EPI_ISL_10266708|Belgium|BA.1.1|2022-02-18	99,64
EPI_ISL_10268638	BA.1	BA.1.1	BA.1.1_2	APU-13710|EPI_ISL_10268638|Peru|BA.1|2022-01-21	96,57
EPI_ISL_10269850	BA.1.1	BA.1.1	BA.1.1_2	MA-CDC-LC0533122|EPI_ISL_10269850|USA|BA.1.1|2022-02-07	99,64
EPI_ISL_10270619	BA.1	BA.1	BA.1_3	NY-WMC2021-1194|EPI_ISL_10270619|USA|BA.1|2021-12-25	93,88
EPI_ISL_10272633	BA.1.1	BA.1.1	BA.1.1_2	NY-GBW-JFK000001|EPI_ISL_10272633|USA|BA.1.1|2021-12-29	96,58
EPI_ISL_10273161	BA.1.1	BA.1.1	BA.1.1_1	MT-CDC-LC0538122|EPI_ISL_10273161|USA|BA.1.1|2022-02-10	99,64
EPI_ISL_10273203	BA.1.1	BA.1.1	BA.1.1_2	AZ-CDC-LC0537902|EPI_ISL_10273203|USA|BA.1.1|2022-02-10	99,64
EPI_ISL_10274333	BA.1.15	BA.1.15	BA.1.15	OR-CDC-ASC210697863|EPI_ISL_10274333|USA|BA.1.15|2022-02-08	99,64
EPI_ISL_10274368	BA.1.1	BA.1.1	BA.1.1_2	MI-CDC-ASC210698079|EPI_ISL_10274368|USA|BA.1.1|2022-02-08	99,64
EPI_ISL_10274767	BA.1.1	BA.1.1	BA.1.1_2	BL-ETHZ-36308409|EPI_ISL_10274767|Switzerland|BA.1.1|2022-02-10	89,24
EPI_ISL_10277350	BA.1.1.18	BA.1.1	BA.1.1_2	GA-CDC-MMB14217562|EPI_ISL_10277350|USA|BA.1.1.18|2022-02-07	97,20
EPI_ISL_10278257	BA.1.1.18	BA.1.1	BA.1.1_2	CA-CDC-FG-273760|EPI_ISL_10278257|USA|BA.1.1.18|2022-02-08	95,61
EPI_ISL_10278314	BA.1.1	BA.1.1	BA.1.1_2	CA-CDC-FG-275128|EPI_ISL_10278314|USA|BA.1.1|2022-02-09	97,73
EPI_ISL_10279318	BA.1.15	BA.1	BA.1_3	MN-CDC-IBX142171603784|EPI_ISL_10279318|USA|BA.1.15|2022-02-10	96,26
EPI_ISL_10280752	BA.1.17	BA.1	BA.1_4	CM-HGUCR-821470|EPI_ISL_10280752|Spain|BA.1.17|2022-02-03	99,64
EPI_ISL_10282027	BA.1.1	BA.1.1	BA.1.1_2	TN-ASC-210541012|EPI_ISL_10282027|USA|BA.1.1|2022-01-23	99,64
EPI_ISL_10283800	BA.2	A	A_14	SH-RKI-I-545068|EPI_ISL_10283800|Germany|BA.2|2022-02-07	99,81
EPI_ISL_10284931	BA.2	A	A_17	MILK-3772582|EPI_ISL_10284931|United Kingdom|BA.2|2022-02-19	99,81
EPI_ISL_10287489	BA.1.1	BA.1.1	BA.1.1_2	MILK-375FE44|EPI_ISL_10287489|United Kingdom|BA.1.1|2022-02-17	96,03
EPI_ISL_10287760	BA.2.16	A	A_14	LSPA-3762BB0|EPI_ISL_10287760|United Kingdom|BA.2.16|2022-02-14	99,81
EPI_ISL_10288359	BA.1.1	BA.1.1	BA.1.1_2	LSPA-374B92C|EPI_ISL_10288359|United Kingdom|BA.1.1|2022-02-16	99,64
EPI_ISL_10288538	BA.1.14	BA.1	BA.1_3	NW-RKI-I-547846|EPI_ISL_10288538|Germany|BA.1.14|2022-02-10	99,64
EPI_ISL_10288778	BA.1	BA.1	BA.1_4	NW-RKI-I-547909|EPI_ISL_10288778|Germany|BA.1|2022-02-10	99,64
EPI_ISL_10290935	BA.2	A	A_17	NW-RKI-I-550154|EPI_ISL_10290935|Germany|BA.2|2022-02-10	99,81
EPI_ISL_10293775	BA.1	BA.1.15	BA.1.15	RP-RKI-I-554017|EPI_ISL_10293775|Germany|BA.1|2022-02-11	99,64
EPI_ISL_10293905	BA.1.1	BA.1.1	BA.1.1_2	NW-RKI-I-554148|EPI_ISL_10293905|Germany|BA.1.1|2022-02-12	99,64
EPI_ISL_10293926	BA.1.1	BA.1.1	BA.1.1_2	NW-RKI-I-554169|EPI_ISL_10293926|Germany|BA.1.1|2022-02-12	99,64
EPI_ISL_10295849	BA.2	A	A_17	SH-RKI-I-558047|EPI_ISL_10295849|Germany|BA.2|2022-02-09	99,81
EPI_ISL_10296606	BA.1.1	BA.1.1	BA.1.1_2	HE-RKI-I-558846|EPI_ISL_10296606|Germany|BA.1.1|2022-02-09	99,62
EPI_ISL_10296792	BA.1.1	BA.1.1	BA.1.1_2	Bangkok-CONI-1965|EPI_ISL_10296792|Thailand|BA.1.1|2022-01-29	82,48
EPI_ISL_10296999	BA.1	BA.1.15	BA.1.15	HH-RKI-I-559222|EPI_ISL_10296999|Germany|BA.1|2022-02-09	99,64
EPI_ISL_10297490	BA.1.20	BA.1.15	BA.1.15	DC-DFS-PHL-05682|EPI_ISL_10297490|USA|BA.1.20|2021-12-30	74,30
EPI_ISL_10300050	BA.1.1	BA.1.1	BA.1.1_2	CO-CDPHE-2102816556|EPI_ISL_10300050|USA|BA.1.1|2022-01-27	99,64
EPI_ISL_10303837	BA.2	BA.2.12.1	BA.2.12.1	SN-RKI-I-564850|EPI_ISL_10303837|Germany|BA.2|2022-02-17	88,76
EPI_ISL_10305815	BA.2.9	A	A_17	BE-RKI-I-565667|EPI_ISL_10305815|Germany|BA.2.9|2022-02-14	99,79
EPI_ISL_10306891	BA.1.14	BA.1.15	BA.1.15	SH-RKI-I-566399|EPI_ISL_10306891|Germany|BA.1.14|2022-02-11	99,64
EPI_ISL_10309063	BA.1.1	BA.1.15	BA.1.15	SP-IB_151345|EPI_ISL_10309063|Brazil|BA.1.1|2022-02-04	84,93
EPI_ISL_10309236	BA.1.14.1	BA.1.15	BA.1.15	SP-IB_151160|EPI_ISL_10309236|Brazil|BA.1.14.1|2022-01-31	88,51
EPI_ISL_10309930	BA.1.1	BA.1.1	BA.1.1_2	SP-IB_151748|EPI_ISL_10309930|Brazil|BA.1.1|2022-01-26	97,96
EPI_ISL_10310988	BA.1.1	BA.1.1	BA.1.1_2	TH-RKI-I-567344|EPI_ISL_10310988|Germany|BA.1.1|2022-01-18	99,56
EPI_ISL_10311135	BA.2	BA.2	BA.2_1	RI-CDCBI-CRSP_D22QMBBIXZ23SPSP|EPI_ISL_10311135|USA|BA.2|2022-02-08	95,21
EPI_ISL_10311612	BA.1.1	BA.1.1	BA.1.1_2	TN-ASC-210600283|EPI_ISL_10311612|USA|BA.1.1|2022-01-17	99,64
EPI_ISL_10316296	BA.1	BA.1.15	BA.1.15	LTU000_NVSPL_MB10460|EPI_ISL_10316296|Lithuania|BA.1|2022-02-01	96,87
EPI_ISL_10317069	BA.1.1	BA.1.1	BA.1.1_3	ST-MD5777|EPI_ISL_10317069|Germany|BA.1.1|2022-01-19	84,53
EPI_ISL_10317224	BA.1.17.2	BA.1.17.2	BA.1.17.2	ST-MD6097|EPI_ISL_10317224|Germany|BA.1.17.2|2022-01-29	99,64
EPI_ISL_10317365	BA.2	A	A_17	ST-MD6467|EPI_ISL_10317365|Germany|BA.2|2022-02-07	99,81
EPI_ISL_10318907	BA.2	BA.2	BA.2_1	DCGC-385981|EPI_ISL_10318907|Denmark|BA.2|2022-02-15	89,45
EPI_ISL_10319003	BA.2	BA.2	BA.2_1	DCGC-386081|EPI_ISL_10319003|Denmark|BA.2|2022-02-19	91,22
EPI_ISL_10319434	BA.1.1	BA.1.1	BA.1.1_2	CONI-2082|EPI_ISL_10319434|Thailand|BA.1.1|2022-02-02	99,64
EPI_ISL_10321155	BA.1.1	BA.1	BA.1_4	CO-CDC-MMB14143049|EPI_ISL_10321155|USA|BA.1.1|2022-02-03	97,20
EPI_ISL_10322698	BA.1.15	BA.1	BA.1_4	GA-CDC-MMB14319509|EPI_ISL_10322698|USA|BA.1.15|2022-02-11	97,20
EPI_ISL_10322772	BA.1	BA.1	BA.1_3	CA-CDC-FG-274933|EPI_ISL_10322772|USA|BA.1|2022-02-09	93,80
EPI_ISL_10331003	BA.1.1	BA.1.15	BA.1.15	DC-DFS-PHL-05732|EPI_ISL_10331003|USA|BA.1.1|2022-01-05	64,24
EPI_ISL_10331828	BA.1	BA.1.15	BA.1.15	CA-CDC-ASC210547296|EPI_ISL_10331828|USA|BA.1|2022-02-04	99,64
EPI_ISL_10332161	BA.1.15	BA.1	BA.1_3	TX-CDC-ASC210548639|EPI_ISL_10332161|USA|BA.1.15|2022-02-07	97,69
EPI_ISL_10333977	BA.1	BA.1.15	BA.1.15	KS-CDC-ASC210700689|EPI_ISL_10333977|USA|BA.1|2022-02-09	99,64
EPI_ISL_10334440	BA.1.1	BA.1.1	BA.1.1_3	WACCBIP-GS2359|EPI_ISL_10334440|Ghana|BA.1.1|2021-12-21	81,21
EPI_ISL_10335150	BA.1	BA.1.15	BA.1.15	ULB-IBC_CV8439029201|EPI_ISL_10335150|Belgium|BA.1|2022-02-07	99,64
EPI_ISL_10335838	BA.2	A	A_14	MILK-37940D4|EPI_ISL_10335838|United Kingdom|BA.2|2022-02-19	99,81
EPI_ISL_10336430	BA.1.1.1	BA.1.1	BA.1.1_2	QEUH-378AEE6|EPI_ISL_10336430|United Kingdom|BA.1.1.1|2022-02-18	99,64
EPI_ISL_10337431	BA.1.1.13	BA.1.1	BA.1.1_3	QEUH-377A07A|EPI_ISL_10337431|United Kingdom|BA.1.1.13|2022-02-17	99,64
EPI_ISL_10338044	BA.2	A	A_14	QEUH-3734917|EPI_ISL_10338044|United Kingdom|BA.2|2022-02-15	99,81
EPI_ISL_10338064	BA.1.1	BA.1.1	BA.1.1_3	ALDP-37359E9|EPI_ISL_10338064|United Kingdom|BA.1.1|2022-02-15	99,64
EPI_ISL_10340980	BA.1.1.2	BA.1.1	BA.1.1_3	TKYkbm4830|EPI_ISL_10340980|Japan|BA.1.1.2|2022-01-17	99,64
EPI_ISL_10347292	BA.1.17	BA.1	BA.1_4	MC-HCUVA-89071165|EPI_ISL_10347292|Spain|BA.1.17|2022-01-11	98,15
EPI_ISL_10348110	BA.2.10	A	A_14	KDCA29868|EPI_ISL_10348110|South Korea|BA.2.10|2022-01-21	99,75
EPI_ISL_10350354	BA.1.1	BA.1.1	BA.1.1_2	DHSC-CYB7H1Z|EPI_ISL_10350354|United Kingdom|BA.1.1|2022-02-02	93,34
EPI_ISL_10353900	BA.1.17.2	BA.1.17.2	BA.1.17.2	DHSC-CYBDGY8|EPI_ISL_10353900|United Kingdom|BA.1.17.2|2022-01-31	97,20
EPI_ISL_10354654	BA.1.17.2	BA.1	BA.1_4	DHSC-CYBFOTS|EPI_ISL_10354654|United Kingdom|BA.1.17.2|2022-01-04	97,20
EPI_ISL_10356083	BA.1.1	BA.1.1	BA.1.1_2	DHSC-CYBMTTJ|EPI_ISL_10356083|United Kingdom|BA.1.1|2022-02-08	96,39
EPI_ISL_10356893	BA.1	BA.1	BA.1_4	DHSC-CYBQ9SM|EPI_ISL_10356893|United Kingdom|BA.1|2022-01-02	97,25
EPI_ISL_10357154	BA.1.17.2	BA.1.17.2	BA.1.17.2	DHSC-CYBRBC1|EPI_ISL_10357154|United Kingdom|BA.1.17.2|2022-01-31	86,17
EPI_ISL_10357481	BA.1	BA.1	BA.1_4	7152|EPI_ISL_10357481|Norway|BA.1|2022-01-31	99,64
EPI_ISL_10358346	BA.1.1	BA.1.1	BA.1.1_2	DHSC-CYBWN6M|EPI_ISL_10358346|United Kingdom|BA.1.1|2022-01-20	93,34
EPI_ISL_10358827	BA.1	BA.1.1.16	BA.1.1.16	IMBA_IMP-1558_H04|EPI_ISL_10358827|Austria|BA.1|2022-02-13	49,80
EPI_ISL_10361421	BA.1	BA.1	BA.1_1	PUE_InDRE_FB9546_E21314775893_S11969|EPI_ISL_10361421|Mexico|BA.1|2022-01-25	97,61
EPI_ISL_10365360	BA.1.1	BA.1.1	BA.1.1_2	DHSC-CYDH9GP|EPI_ISL_10365360|United Kingdom|BA.1.1|2021-12-21	97,25
EPI_ISL_10366345	BA.1	BA.1.1	BA.1.1_3	OCC-HCL722000641401|EPI_ISL_10366345|France|BA.1|2022-02-06	99,64
EPI_ISL_10368939	BA.1	BA.1	BA.1_4	CA-CDC-ASC210551978|EPI_ISL_10368939|USA|BA.1|2022-02-09	96,95
EPI_ISL_10369447	BA.2	BA.2	BA.2_1	MH-INSACOGIP10301|EPI_ISL_10369447|India|BA.2|2022-02-05	99,81
EPI_ISL_10369718	BA.1.1	BA.1.1	BA.1.1_2	CO-CDC-ASC210551088|EPI_ISL_10369718|USA|BA.1.1|2022-02-09	99,64
EPI_ISL_10373043	BA.1.18	BA.1.15	BA.1.15	CT-CDC-ASC210553045|EPI_ISL_10373043|USA|BA.1.18|2022-02-11	99,64
EPI_ISL_10376342	BA.1.1	BA.1.1	BA.1.1_2	DHSC-CYN3SCT|EPI_ISL_10376342|United Kingdom|BA.1.1|2022-02-05	97,20
EPI_ISL_10379018	BA.1	BA.1	BA.1_4	DHSC-CYNAN6N|EPI_ISL_10379018|United Kingdom|BA.1|2022-01-31	94,47
EPI_ISL_10380028	BA.1.1.15	BA.1.1	BA.1.1_2	DHSC-CYND8PM|EPI_ISL_10380028|United Kingdom|BA.1.1.15|2021-12-20	97,20
EPI_ISL_10380298	BA.1.17	BA.1	BA.1_4	DHSC-CYNE1ZB|EPI_ISL_10380298|United Kingdom|BA.1.17|2022-01-10	93,34
EPI_ISL_10380622	BA.1	BA.1	BA.1_4	DHSC-CYNETM5|EPI_ISL_10380622|United Kingdom|BA.1|2022-02-04	97,20
EPI_ISL_10383693	BA.2.10	A	A_14	NJ-CDC-QDX33831835|EPI_ISL_10383693|USA|BA.2.10|2022-02-12	99,81
EPI_ISL_10383919	BA.1.15	BA.1	BA.1_4	OR-OSPHL05155|EPI_ISL_10383919|USA|BA.1.15|2022-02-08	97,20
EPI_ISL_10384521	BA.1.15.1	BA.1.15.1	BA.1.15.1	DHSC-CYNT9YO|EPI_ISL_10384521|United Kingdom|BA.1.15.1|2021-12-18	97,96
EPI_ISL_10386588	BA.1.1	BA.1.1	BA.1.1_2	MT-CDC-ASC210696999|EPI_ISL_10386588|USA|BA.1.1|2022-02-11	99,64
EPI_ISL_10387815	BA.1.1.18	BA.1.1	BA.1.1_2	TN-CDC-ASC210698744|EPI_ISL_10387815|USA|BA.1.1.18|2022-02-11	96,93
EPI_ISL_10387904	BA.1.1	BA.1.1	BA.1.1_2	TX-CDC-ASC210553678|EPI_ISL_10387904|USA|BA.1.1|2022-02-11	96,83
EPI_ISL_10389200	BA.1.1	BA.1.1	BA.1.1_2	DHSC-CYYAQ4Y|EPI_ISL_10389200|United Kingdom|BA.1.1|2022-02-02	93,55
EPI_ISL_10389344	BA.1	BA.1	BA.1_4	DHSC-CYYB477|EPI_ISL_10389344|United Kingdom|BA.1|2022-02-04	97,48
EPI_ISL_10389575	BA.1.1	BA.1.1	BA.1.1_3	WV-CDC-ASC210553910|EPI_ISL_10389575|USA|BA.1.1|2022-02-12	99,64
EPI_ISL_10389867	BA.1.1	BA.1.1	BA.1.1_2	NJ-CDC-ASC210554135|EPI_ISL_10389867|USA|BA.1.1|2022-02-12	99,64
EPI_ISL_10390211	BA.2	A	A_14	NH-CDC-ASC210554823|EPI_ISL_10390211|USA|BA.2|2022-02-12	99,81
EPI_ISL_10393323	BA.1	BA.1	BA.1_4	DHSC-CYYT9OT|EPI_ISL_10393323|United Kingdom|BA.1|2022-02-05	97,20
EPI_ISL_10395292	BA.2.9	A	A_17	PHWC-PEJO69|EPI_ISL_10395292|United Kingdom|BA.2.9|2022-02-02	99,81

EPI_ISL_10396320	BA.1.1	BA.1.1	BA.1.1_2	IL-IDPH-PER-SV-002565|EPI_ISL_10396320|USA|BA.1.1|2021-12-29	99,64
EPI_ISL_10396851	BA.1.1	BA.1.1	BA.1.1_2	ND-NDDH-11952|EPI_ISL_10396851|USA|BA.1.1|2022-02-17	91,26
EPI_ISL_10397730	BA.1.1	BA.1.1	BA.1.1_2	WA-PHL-014707|EPI_ISL_10397730|USA|BA.1.1|2022-02-15	96,03
EPI_ISL_10397944	BA.1.1	BA.1.1	BA.1.1_2	WA-PHL-014926|EPI_ISL_10397944|USA|BA.1.1|2022-02-14	96,03
EPI_ISL_10399968	BA.2	BA.2	BA.2_1	DCGC-390148|EPI_ISL_10399968|Denmark|BA.2|2022-02-15	99,79
EPI_ISL_10400755	BA.2	A	A_17	DCGC-390940|EPI_ISL_10400755|Denmark|BA.2|2022-02-15	99,81
EPI_ISL_10401013	BA.2.9	A	A_17	DCGC-391199|EPI_ISL_10401013|Denmark|BA.2.9|2022-02-15	99,81
EPI_ISL_10401376	BA.2	BA.2	BA.2_1	DCGC-391564|EPI_ISL_10401376|Denmark|BA.2|2022-02-15	99,77
EPI_ISL_10402851	BA.2	BA.2	BA.2_1	Kce-2466|EPI_ISL_10402851|Poland|BA.2|2022-02-21	94,75
EPI_ISL_10403715	BA.1	BA.1	BA.1_1	2202-1096_wsselodz|EPI_ISL_10403715|Poland|BA.1|2022-02-06	88,84
EPI_ISL_10403914	BA.1.1	BA.1.15	BA.1.15	CA-CDPH-500045148|EPI_ISL_10403914|USA|BA.1.1|2022-01-06	80,12
EPI_ISL_10404677	BA.1.1	BA.1.1	BA.1.1_2	CA-CDPH-3000302347|EPI_ISL_10404677|USA|BA.1.1|2022-01-14	95,73
EPI_ISL_10405054	BA.1	BA.2	BA.2_1	CA-CDPH-3000306855|EPI_ISL_10405054|USA|BA.1|2022-02-01	92,23
EPI_ISL_10406489	BA.1.1	BA.1	BA.1_3	Kumamoto-K3275|EPI_ISL_10406489|Japan|BA.1.1|2022-01	97,58
EPI_ISL_10407931	BA.1	BA.1.15	BA.1.15	CA-CDPH-3000316071|EPI_ISL_10407931|USA|BA.1|2022-02-15	69,05
EPI_ISL_10408599	BA.1.15	BA.1.15	BA.1.15	CO-CDPHE-2102853245|EPI_ISL_10408599|USA|BA.1.15|2022-01-06	99,64
EPI_ISL_10408884	BA.1.1.18	BA.1.1	BA.1.1_3	CO-CDPHE-2102872582|EPI_ISL_10408884|USA|BA.1.1.18|2022-01-30	92,44
EPI_ISL_10410562	BA.1.1	BA.1.1	BA.1.1_2	CA-CDPH-3000306290|EPI_ISL_10410562|USA|BA.1.1|2022-02-01	99,64
EPI_ISL_10410617	BA.1.1	BA.1.1	BA.1.1_2	CA-CDPH-3000310279|EPI_ISL_10410617|USA|BA.1.1|2022-02-06	89,16
EPI_ISL_10412484	BA.1.1	BA.1.1	BA.1.1_2	LSPA-37E5BD3|EPI_ISL_10412484|United Kingdom|BA.1.1|2022-02-21	95,99
EPI_ISL_10413071	BA.1.1.12	BA.1.1	BA.1.1_3	MILK-37DE1BD|EPI_ISL_10413071|United Kingdom|BA.1.1.12|2022-02-19	99,64
EPI_ISL_10413364	BA.1	BA.1.15	BA.1.15	ALDP-37D8687|EPI_ISL_10413364|United Kingdom|BA.1|2022-02-19	99,64
EPI_ISL_10414246	BA.1.17	BA.1.15	BA.1.15	MILK-37DF741|EPI_ISL_10414246|United Kingdom|BA.1.17|2022-02-21	99,64
EPI_ISL_10415265	BA.1.17.2	BA.1.17.2	BA.1.17.2	MILK-37B82F2|EPI_ISL_10415265|United Kingdom|BA.1.17.2|2022-02-20	99,64
EPI_ISL_10415508	BA.2	A	A_17	MILK-37B8AEB|EPI_ISL_10415508|United Kingdom|BA.2|2022-02-20	99,81
EPI_ISL_10415585	BA.1.1	BA.1.1	BA.1.1_3	LSPA-37BF02E|EPI_ISL_10415585|United Kingdom|BA.1.1|2022-02-19	99,64
EPI_ISL_10416452	BA.1.1	BA.1.1	BA.1.1_3	LSPA-37B7150|EPI_ISL_10416452|United Kingdom|BA.1.1|2022-02-19	99,64
EPI_ISL_10417032	BA.1.1	BA.1.1	BA.1.1_3	ALDP-37B3C35|EPI_ISL_10417032|United Kingdom|BA.1.1|2022-02-18	99,64
EPI_ISL_10417278	BA.2	A	A_17	MILK-37CBEDA|EPI_ISL_10417278|United Kingdom|BA.2|2022-02-20	99,81
EPI_ISL_10417972	BA.1	BA.1.15	BA.1.15	QEUH-37D1624|EPI_ISL_10417972|United Kingdom|BA.1|2022-02-19	99,64
EPI_ISL_10419252	BA.2	A	A_14	ALDP-37BBB03|EPI_ISL_10419252|United Kingdom|BA.2|2022-02-20	99,81
EPI_ISL_10421569	BA.2	BA.2	BA.2_1	MILK-37998AA|EPI_ISL_10421569|United Kingdom|BA.2|2022-02-19	99,81
EPI_ISL_10423231	BA.1.15.1	BA.1.15.1	BA.1.15.1	MILK-36DEFC3|EPI_ISL_10423231|United Kingdom|BA.1.15.1|2022-02-14	99,64
EPI_ISL_10423344	BA.1.1.14	BA.1.1	BA.1.1_3	QEUH-36DF5C0|EPI_ISL_10423344|United Kingdom|BA.1.1.14|2022-02-13	99,64
EPI_ISL_10424800	BA.1.1	BA.1.1	BA.1.1_3	ALDP-36D7EDC|EPI_ISL_10424800|United Kingdom|BA.1.1|2022-02-13	99,64
EPI_ISL_10425287	BA.1.1.15	BA.1.1	BA.1.1_3	QEUH-36CCA9A|EPI_ISL_10425287|United Kingdom|BA.1.1.15|2022-02-12	99,64
EPI_ISL_10426652	BA.1.1	BA.1.1	BA.1.1_2	QEUH-36CE078|EPI_ISL_10426652|United Kingdom|BA.1.1|2022-02-12	99,64
EPI_ISL_10426686	BA.1	BA.1	BA.1_4	CO-CDC-MMB14022410|EPI_ISL_10426686|USA|BA.1|2022-01-30	84,56
EPI_ISL_10427416	BA.1.1	BA.1.1	BA.1.1_3	MILK-36BDA50|EPI_ISL_10427416|United Kingdom|BA.1.1|2022-02-13	99,64
EPI_ISL_10428452	BA.1	BA.1.15	BA.1.15	MILK-36C817B|EPI_ISL_10428452|United Kingdom|BA.1|2022-02-13	99,58
EPI_ISL_10428571	BA.2	A	A_14	QEUH-36C22BA|EPI_ISL_10428571|United Kingdom|BA.2|2022-02-12	99,81
EPI_ISL_10429043	BA.1.1	BA.1.1	BA.1.1_2	MILK-36C483E|EPI_ISL_10429043|United Kingdom|BA.1.1|2022-02-13	99,64
EPI_ISL_10430200	BA.1.15	BA.1	BA.1_3	NCDC-NR-GL-002403|EPI_ISL_10430200|Nigeria|BA.1.15|2021-12-16	70,52
EPI_ISL_10430564	BA.1.1	BA.1.1	BA.1.1_3	MN-CDC-QDX33874022|EPI_ISL_10430564|USA|BA.1.1|2022-02-09	99,64
EPI_ISL_10430576	BA.1.15	BA.1.15	BA.1.15	TX-CDC-QDX33831901|EPI_ISL_10430576|USA|BA.1.15|2022-02-10	99,64
EPI_ISL_10432146	BA.1.1.2	BA.1.1	BA.1.1_3	SZ-NIG-Y213426|EPI_ISL_10432146|Japan|BA.1.1.2|2022-02-07	99,64
EPI_ISL_10433117	BA.2	A	A_14	NSW-ICPMR-21006|EPI_ISL_10433117|Australia|BA.2|2022-02-16	78,76
EPI_ISL_10435266	BA.1.17.2	BA.1.17.2	BA.1.17.2	PT28345|EPI_ISL_10435266|Portugal|BA.1.17.2|2022-02-15	99,64
EPI_ISL_10436445	BA.1.1.1	BA.1.1	BA.1.1_3	run220223_RUVZ_TN-22-0496|EPI_ISL_10436445|Slovakia|BA.1.1.1|2022-02-23	99,64
EPI_ISL_10437621	BD.1	BD.1	BD.1	DHSC-CYB4JSE|EPI_ISL_10437621|United Kingdom|BD.1|2022-02-10	97,20
EPI_ISL_10437691	BA.1.17.2	BA.1.17.2	BA.1.17.2	DHSC-CYB51YK|EPI_ISL_10437691|United Kingdom|BA.1.17.2|2021-12-20	97,20
EPI_ISL_10440745	BA.1.1	BA.1	BA.1_4	DHSC-CYBIPFT|EPI_ISL_10440745|United Kingdom|BA.1.1|2022-01-12	90,96
EPI_ISL_10441201	BA.2	A	A_14	DHSC-CYBKM9I|EPI_ISL_10441201|United Kingdom|BA.2|2022-02-14	99,03
EPI_ISL_10443188	BA.1.15	BA.1.15	BA.1.15	MS-UMMC-M477F6-532399|EPI_ISL_10443188|USA|BA.1.15|2022-02-09	94,71
EPI_ISL_10443618	BA.2	BA.2	BA.2_1	HSGM-G3226|EPI_ISL_10443618|Turkey|BA.2|2022-02-18	99,81
EPI_ISL_10444055	BA.1.1.15	BA.1.1	BA.1.1_2	22BG-EU_014921_Pl140|EPI_ISL_10444055|Bulgaria|BA.1.1.15|2022-01-08	99,64
EPI_ISL_10444310	BA.1	BA.1	BA.1_4	22BG-EU_015188_Pl143|EPI_ISL_10444310|Bulgaria|BA.1|2022-01-10	99,64
EPI_ISL_10445206	BA.1.1	BA.1.1	BA.1.1_2	DHSC-CYBYWFI|EPI_ISL_10445206|United Kingdom|BA.1.1|2022-02-09	93,34
EPI_ISL_10446008	BA.2	A	A_14	DHSC-CYD4B66|EPI_ISL_10446008|United Kingdom|BA.2|2022-02-12	99,03
EPI_ISL_10446996	BA.1.1.14	BA.1.1	BA.1.1_2	DHSC-CYD8DMK|EPI_ISL_10446996|United Kingdom|BA.1.1.14|2022-02-11	88,57
EPI_ISL_10450985	BA.1	BA.1	BA.1_4	VD-CHUV-GEN10562|EPI_ISL_10450985|Switzerland|BA.1|2022-02-16	96,34
EPI_ISL_10451692	BA.1.1	BA.1.1	BA.1.1_2	TI-EOC-26698396|EPI_ISL_10451692|Switzerland|BA.1.1|2022-02-22	99,54
EPI_ISL_10452487	BA.1.1	BA.1	BA.1_4	DHSC-CYN18DY|EPI_ISL_10452487|United Kingdom|BA.1.1|2022-02-12	92,52
EPI_ISL_10454375	BA.2	A	A_14	MILK-380288D|EPI_ISL_10454375|United Kingdom|BA.2|2022-02-20	99,81
EPI_ISL_10458896	BA.1	BA.1	BA.1_4	DHSC-CYNYIQB|EPI_ISL_10458896|United Kingdom|BA.1|2021-12-21	97,20
EPI_ISL_10460519	BA.1.15.1	BA.1.15.1	BA.1.15.1	DHSC-CYY9IMK|EPI_ISL_10460519|United Kingdom|BA.1.15.1|2021-12-21	92,54
EPI_ISL_10461404	BA.2.3	A	A_14	MILK-37F5648|EPI_ISL_10461404|United Kingdom|BA.2.3|2022-02-21	99,81
EPI_ISL_10461998	BA.1.1	BA.1.1	BA.1.1_3	LSPA-37F91DA|EPI_ISL_10461998|United Kingdom|BA.1.1|2022-02-21	99,64
EPI_ISL_10463075	BA.2	A	A_14	MILK-37FDEC0|EPI_ISL_10463075|United Kingdom|BA.2|2022-02-21	99,81
EPI_ISL_10463555	BA.2	BA.2	BA.2_1	LSPA-37E836F|EPI_ISL_10463555|United Kingdom|BA.2|2022-02-20	99,81
EPI_ISL_10464416	BA.2	A	A_14	MILK-37F4CDE|EPI_ISL_10464416|United Kingdom|BA.2|2022-02-21	99,81
EPI_ISL_10464480	BA.1.15	BA.1	BA.1_4	NM-CDC-ASC210856487|EPI_ISL_10464480|USA|BA.1.15|2022-02-13	99,64
EPI_ISL_10466235	BA.1	BA.1	BA.1_3	IL-CDC-ASC210556303|EPI_ISL_10466235|USA|BA.1|2022-02-14	96,93
EPI_ISL_10466507	BA.1.1	BA.1.1	BA.1.1_2	NE-CDC-ASC210858079|EPI_ISL_10466507|USA|BA.1.1|2022-02-14	96,76
EPI_ISL_10468197	BA.2.1	A	A_14	LSPA-37536FD|EPI_ISL_10468197|United Kingdom|BA.2.1|2022-02-17	99,81
EPI_ISL_10469804	BA.1.17	BA.1.17.2	BA.1.17.2	LSPA-3752B0E|EPI_ISL_10469804|United Kingdom|BA.1.17|2022-02-16	99,64
EPI_ISL_10469813	BA.1	BA.1	BA.1_4	DHSC-CYYSEIW|EPI_ISL_10469813|United Kingdom|BA.1|2022-02-12	93,34
EPI_ISL_10469921	BA.1.1.18	BA.1.1	BA.1.1_2	FL-CDC-ASC210857669|EPI_ISL_10469921|USA|BA.1.1.18|2022-02-14	99,64
EPI_ISL_10471130	BA.1.17	BA.1	BA.1_4	CM-HGUCR-822572|EPI_ISL_10471130|Spain|BA.1.17|2022-02-04	99,64
EPI_ISL_10471247	BA.1.20	BA.1.15	BA.1.15	KY-CDC-ASC210816267|EPI_ISL_10471247|USA|BA.1.20|2022-02-15	93,05
EPI_ISL_10473837	BA.2	A	A_14	MILK-370D470|EPI_ISL_10473837|United Kingdom|BA.2|2022-02-16	99,81
EPI_ISL_10475724	BA.2	A	A_14	NIRE-019e1f|EPI_ISL_10475724|United Kingdom|BA.2|2022-02-08	99,81
EPI_ISL_10476772	BA.1.15	BA.1.15	BA.1.15	CA-CDC-FG-277819|EPI_ISL_10476772|USA|BA.1.15|2022-02-11	93,68
EPI_ISL_10479361	BA.1	BA.1	BA.1_2	CA-CDC-FG-277123|EPI_ISL_10479361|USA|BA.1|2022-02-11	98,09
EPI_ISL_10480476	BA.1.1	BA.1.1	BA.1.1_3	NORT-YNBHQYW|EPI_ISL_10480476|United Kingdom|BA.1.1|2022	99,64
EPI_ISL_10480681	BA.2	A	A_14	01_SE100_22CS102395|EPI_ISL_10480681|Sweden|BA.2|2022-02-18	94,91
EPI_ISL_10482130	BA.1.15	BA.1	BA.1_3	NORW-3189273|EPI_ISL_10482130|United Kingdom|BA.1.15|2022-01-13	96,03
EPI_ISL_10482705	BA.1	BA.1.15	BA.1.15	NORW-318E91E|EPI_ISL_10482705|United Kingdom|BA.1|2022-01-02	91,66
EPI_ISL_10482921	BA.1.1	BA.1.1	BA.1.1_2	QEUH-36FCADD|EPI_ISL_10482921|United Kingdom|BA.1.1|2022-02-14	99,64
EPI_ISL_10485730	BA.2	BA.2	BA.2_1	SG-Risch-2222205402|EPI_ISL_10485730|Switzerland|BA.2|2022-02-14	90,52
EPI_ISL_10486622	BA.2.10	A	A_14	QEUH-36FAC33|EPI_ISL_10486622|United Kingdom|BA.2.10|2022-02-14	99,81
EPI_ISL_10486759	BA.1.15	BA.1.15	BA.1.15	QEUH-36FB467|EPI_ISL_10486759|United Kingdom|BA.1.15|2022-02-13	99,64
EPI_ISL_10488616	BA.1.1	BA.1.1	BA.1.1_3	ALDP-36F6D70|EPI_ISL_10488616|United Kingdom|BA.1.1|2022-02-13	99,64
EPI_ISL_10488731	BA.1.1.14	BA.1.1	BA.1.1_2	LSPA-36F5A4D|EPI_ISL_10488731|United Kingdom|BA.1.1.14|2022-02-12	99,64
EPI_ISL_10490160	XS	BA.1.1	BA.1.1_2	ND-NDDH-12071|EPI_ISL_10490160|USA|XS|2022-02-21	96,18
EPI_ISL_10491248	BA.1.1	BA.1.1	BA.1.1_2	OH-CDC-ASC210859400|EPI_ISL_10491248|USA|BA.1.1|2022-02-15	96,93
EPI_ISL_10491930	BA.1.1	BA.1.1	BA.1.1_2	CA-CDC-ASC210818898|EPI_ISL_10491930|USA|BA.1.1|2022-02-16	96,37
EPI_ISL_10492220	BA.1.1	BA.1.1	BA.1.1_2	TN-CDC-ASC210861170|EPI_ISL_10492220|USA|BA.1.1|2022-02-18	98,07
EPI_ISL_10492238	BA.1.20	BA.1	BA.1_3	CT-CDC-ASC210860568|EPI_ISL_10492238|USA|BA.1.20|2022-02-12	96,76
EPI_ISL_10494799	BA.1.1	BA.1.1	BA.1.1_2	IMR_WC80612|EPI_ISL_10494799|Malaysia|BA.1.1|2022-01-31	82,48
EPI_ISL_10495946	BA.1	BA.1	BA.1_3	IC-5207|EPI_ISL_10495946|Japan|BA.1|2022-02-01	99,64

EPI_ISL_10497522	BA.1	BA.1	BA.1_1	2141WSN2022_wsseol|EPI_ISL_10497522|Poland|BA.1|2022-02-09	93,99
EPI_ISL_10498378	BA.1.1	BA.1.1	BA.1.1_2	DHSC-CYBIDYF|EPI_ISL_10498378|United Kingdom|BA.1.1|2022-02-15	97,31
EPI_ISL_10498479	BA.1.18	BA.1	BA.1_4	OR_UO_MW000589_S321_L001|EPI_ISL_10498479|USA|BA.1.18|2022-01-19	99,68
EPI_ISL_10498830	BA.1	BA.2.12	BA.2.12	OR_UO_MAP003088_S12_L002|EPI_ISL_10498830|USA|BA.1|2022-01-25	84,16
EPI_ISL_10500705	BA.1.1	BA.1.1	BA.1.1_2	DHSC-CYNJYB6|EPI_ISL_10500705|United Kingdom|BA.1.1|2022-02-14	97,16
EPI_ISL_10500921	BA.1.1	BA.1.1	BA.1.1_2	DHSC-CYNR4W8|EPI_ISL_10500921|United Kingdom|BA.1.1|2022-02-13	97,20
EPI_ISL_10501654	BA.1.1	BA.1.1	BA.1.1_3	NORT-YNB6914|EPI_ISL_10501654|United Kingdom|BA.1.1|2022	99,64
EPI_ISL_10502362	BA.2	BA.2	BA.2_1	UZA-UA-CV8521203961|EPI_ISL_10502362|Belgium|BA.2|2022-02-22	99,81
EPI_ISL_10502450	BA.2	A	A_14	NORT-YNB74QK|EPI_ISL_10502450|United Kingdom|BA.2|2022-02-10	99,81
EPI_ISL_10502485	BA.2	A	A_14	NORT-YNB76BY|EPI_ISL_10502485|United Kingdom|BA.2|2022	99,56
EPI_ISL_10503364	BA.1.1	BA.1	BA.1_4	Jessa_11-2208-005070ZOL|EPI_ISL_10503364|Belgium|BA.1.1|2022-02-24	97,31
EPI_ISL_10503413	BA.1	BA.1.15	BA.1.15	PHEC-YYNW8NK|EPI_ISL_10503413|United Kingdom|BA.1|2022-02-03	92,33
EPI_ISL_10505451	BA.1	BA.1	BA.1_4	PZH-GUM-13631|EPI_ISL_10505451|Poland|BA.1|2022-02-03	99,64
EPI_ISL_10505760	BA.1.1	BA.1.1	BA.1.1_3	PZH-GUM-14211|EPI_ISL_10505760|Poland|BA.1.1|2022-02-12	99,64
EPI_ISL_10506277	BA.2	A	A_14	PHEP-YYREWRO|EPI_ISL_10506277|United Kingdom|BA.2|2022-02-09	99,77
EPI_ISL_10506612	BA.2	A	A_17	DCGC-392132|EPI_ISL_10506612|Denmark|BA.2|2022-02-22	99,81
EPI_ISL_10509075	BA.1.17.2	BA.1.17.2	BA.1.17.2	PHWC-PEMJAQ|EPI_ISL_10509075|United Kingdom|BA.1.17.2|2022-02-06	97,52
EPI_ISL_10510934	BA.2	A	A_17	DCGC-393765|EPI_ISL_10510934|Denmark|BA.2|2022-02-21	99,98
EPI_ISL_10511678	BA.1.1.14	BA.1.1	BA.1.1_2	DCGC-394436|EPI_ISL_10511678|Denmark|BA.1.1.14|2022-02-18	99,68
EPI_ISL_10511974	BA.1	BA.1.15	BA.1.15	NIC_BKK_SEQ00212|EPI_ISL_10511974|Thailand|BA.1|2021-12-31	86,82
EPI_ISL_10512478	BA.2	A	A_14	UNIMAS-GHML1172|EPI_ISL_10512478|Malaysia|BA.2|2022-02-10	99,81
EPI_ISL_10513538	BA.1.17.2	BA.1.17.2	BA.1.17.2	THL-202206336|EPI_ISL_10513538|Finland|BA.1.17.2|2022-02-04	99,71
EPI_ISL_10513726	BA.1.18	BA.1	BA.1_4	THL-202206581|EPI_ISL_10513726|Finland|BA.1.18|2022-02-09	99,64
EPI_ISL_10515820	BA.2	BA.2	BA.2_1	MILK-3812E5B|EPI_ISL_10515820|United Kingdom|BA.2|2022-02-22	99,81
EPI_ISL_10517065	BA.2	BA.2	BA.2_1	NEWC-382C88D|EPI_ISL_10517065|United Kingdom|BA.2|2022-02-23	99,81
EPI_ISL_10518267	BA.1.17.2	BA.1.17.2	BA.1.17.2	LSPA-384E560|EPI_ISL_10518267|United Kingdom|BA.1.17.2|2022-02-23	99,64
EPI_ISL_10520549	BA.1.1	BA.1.1	BA.1.1_2	ALDP-38202B8|EPI_ISL_10520549|United Kingdom|BA.1.1|2022-02-21	99,64
EPI_ISL_10521324	BA.2	BA.2	BA.2_1	MILK-3845B3A|EPI_ISL_10521324|United Kingdom|BA.2|2022-02-23	99,81
EPI_ISL_10521474	BA.1.15	BA.1.15	BA.1.15	MILK-3845EB9|EPI_ISL_10521474|United Kingdom|BA.1.15|2022-02-23	99,64
EPI_ISL_10521797	BA.1.14.1	BA.1.15	BA.1.15	RS-FIOCRUZ-4504|EPI_ISL_10521797|Brazil|BA.1.14.1|2022-01-26	99,64
EPI_ISL_10522221	BA.1.1.15	BA.1.1	BA.1.1_2	MILK-3835E34|EPI_ISL_10522221|United Kingdom|BA.1.1.15|2022-02-23	99,64
EPI_ISL_10525136	BA.1.20	BA.1.15	BA.1.15	MN-CDC-IBX658483800708|EPI_ISL_10525136|USA|BA.1.20|2022-02-15	96,64
EPI_ISL_10525581	BA.2	BA.2	BA.2_1	QEUH-3810558|EPI_ISL_10525581|United Kingdom|BA.2|2022-02-22	99,81
EPI_ISL_10527476	BA.2	BA.2	BA.2_1	QEUH-3801095|EPI_ISL_10527476|United Kingdom|BA.2|2022-02-21	99,81
EPI_ISL_10528750	BA.1.1	BA.1.1	BA.1.1_2	ALDP-380A0F6|EPI_ISL_10528750|United Kingdom|BA.1.1|2022-02-21	99,64
EPI_ISL_10530264	BA.1.1	BA.1.1	BA.1.1_2	LSPA-3818D1C|EPI_ISL_10530264|United Kingdom|BA.1.1|2022-02-22	99,64
EPI_ISL_10531082	BA.2	A	A_14	QEUH-37E5DCE|EPI_ISL_10531082|United Kingdom|BA.2|2022-02-21	99,81
EPI_ISL_10531229	BA.1.15	BA.1.15	BA.1.15	LA-EVTL12609|EPI_ISL_10531229|USA|BA.1.15|2022-01-27	99,64
EPI_ISL_10531468	BA.2	BA.2	BA.2_1	QEUH-37ECF85|EPI_ISL_10531468|United Kingdom|BA.2|2022-02-21	99,81
EPI_ISL_10534248	BA.2	BA.2	BA.2_1	QEUH-37C0E6C|EPI_ISL_10534248|United Kingdom|BA.2|2022-02-20	99,81
EPI_ISL_10535970	BA.2	A	A_14	MILK-37952EC|EPI_ISL_10535970|United Kingdom|BA.2|2022-02-20	99,81
EPI_ISL_10536129	BA.1.1.13	BA.1.1	BA.1.1_4	MILK-37956A4|EPI_ISL_10536129|United Kingdom|BA.1.1.13|2022-02-05	99,64
EPI_ISL_10536737	BA.1	BA.1.15	BA.1.15	MILK-379B398|EPI_ISL_10536737|United Kingdom|BA.1|2022-02-20	96,57
EPI_ISL_10537927	BA.2	A	A_14	MILK-37A9B9F|EPI_ISL_10537927|United Kingdom|BA.2|2022-02-19	99,81
EPI_ISL_10539300	BA.1.1	BA.1.1	BA.1.1_3	QEUH-378FE0F|EPI_ISL_10539300|United Kingdom|BA.1.1|2022-02-18	99,64
EPI_ISL_10542246	BA.2	A	A_14	MILK-377CE05|EPI_ISL_10542246|United Kingdom|BA.2|2022-02-14	99,81
EPI_ISL_10542249	BA.1.1.1	BA.1.1	BA.1.1_2	QEUH-377BFE5|EPI_ISL_10542249|United Kingdom|BA.1.1.1|2022-02-18	96,03
EPI_ISL_10542300	BA.2	A	A_14	QEUH-377A942|EPI_ISL_10542300|United Kingdom|BA.2|2022-02-18	99,81
EPI_ISL_10542570	BA.1.1	BA.1.1	BA.1.1_3	QEUH-377BDEB|EPI_ISL_10542570|United Kingdom|BA.1.1|2022-02-18	99,64
EPI_ISL_10543156	BA.1	BA.1.15	BA.1.15	MILK-373648D|EPI_ISL_10543156|United Kingdom|BA.1|2022-02-16	99,64
EPI_ISL_10547643	BA.1.15	BA.1.15	BA.1.15	TN-ASC-210583927|EPI_ISL_10547643|USA|BA.1.15|2022-01-13	99,64
EPI_ISL_10548251	BA.1.1	BA.1.1	BA.1.1_2	TN-ASC-210588138|EPI_ISL_10548251|USA|BA.1.1|2022-01-10	96,55
EPI_ISL_10548288	BA.1.1	BA.1.1	BA.1.1_2	TN-ASC-210582011|EPI_ISL_10548288|USA|BA.1.1|2022-01-11	99,64
EPI_ISL_10548965	BA.2	BA.2	BA.2_1	QEUH-37817D5|EPI_ISL_10548965|United Kingdom|BA.2|2022-02-17	99,81
EPI_ISL_10549556	BA.1.1	BA.1.1	BA.1.1_2	WI-UW-9555|EPI_ISL_10549556|USA|BA.1.1|2022-02-14	97,46
EPI_ISL_10552575	BA.1.1	BA.1	BA.1_1	CeMM24345|EPI_ISL_10552575|Austria|BA.1.1|2022-02-09	91,03
EPI_ISL_10552721	BA.1.1	BA.1	BA.1_1	CeMM24509|EPI_ISL_10552721|Austria|BA.1.1|2022-02-11	96,22
EPI_ISL_10553142	BA.1	BA.1	BA.1_3	ON-PHL-22-08788|EPI_ISL_10553142|Canada|BA.1|2022-02-12	96,03
EPI_ISL_10556181	BA.1.1	B.1.160.7	B.1.160.7	FL-MSL-3963|EPI_ISL_10556181|USA|BA.1.1|2022-01	96,93
EPI_ISL_10557695	BA.1.1	BA.1.1	BA.1.1_2	CA-HLX-STM-4BZ3KDTWH|EPI_ISL_10557695|USA|BA.1.1|2022-02-06	99,64
EPI_ISL_10558895	BA.1.1	BA.1.1	BA.1.1_2	CA-HLX-STM-78DHZKYXY|EPI_ISL_10558895|USA|BA.1.1|2022-02-15	99,14
EPI_ISL_10560875	BA.1.1	BA.1.1	BA.1.1_2	RI-CDC-LC0542848|EPI_ISL_10560875|USA|BA.1.1|2022-02-14	99,64
EPI_ISL_10561620	BA.1.15	BA.1.15	BA.1.15	AZ-ASU46912|EPI_ISL_10561620|USA|BA.1.15|2022-01-10	94,70
EPI_ISL_10561626	BA.1	BA.1.15	BA.1.15	AZ-ASU46788|EPI_ISL_10561626|USA|BA.1|2022-01-07	81,53
EPI_ISL_10563383	BA.1.20	BA.1	BA.1_4	GA-CDC-LC0544784|EPI_ISL_10563383|USA|BA.1.20|2022-02-17	99,64
EPI_ISL_10563553	BA.1.1	BA.1.1	BA.1.1_2	PA-CDC-LC0546374|EPI_ISL_10563553|USA|BA.1.1|2022-02-18	99,64
EPI_ISL_10563670	BA.1	BA.1.15	BA.1.15	VIC41585|EPI_ISL_10563670|Australia|BA.1|2022-02-09	99,64
EPI_ISL_10563678	BA.1	BA.1.15	BA.1.15	VIC41590|EPI_ISL_10563678|Australia|BA.1|2022-02-10	99,56
EPI_ISL_10563835	BA.1	BA.1.15	BA.1.15	VIC41435|EPI_ISL_10563835|Australia|BA.1|2022-02-08	99,56
EPI_ISL_10567107	BA.1.1	BA.1.1	BA.1.1_2	CA-CDC-FG-280827|EPI_ISL_10567107|USA|BA.1.1|2022-02-14	99,64
EPI_ISL_10569392	BA.1.15	BA.1.15	BA.1.15	CA-CDC-FG-282925|EPI_ISL_10569392|USA|BA.1.15|2022-02-18	99,64
EPI_ISL_10569723	BA.1.20	BA.1	BA.1_4	CA-CDC-FG-283064|EPI_ISL_10569723|USA|BA.1.20|2022-02-18	99,26
EPI_ISL_10570430	BA.1.1	BA.1.1	BA.1.1_2	CA-CDC-FG-283832|EPI_ISL_10570430|USA|BA.1.1|2022-02-20	99,64
EPI_ISL_10570776	BA.1.1.18	BA.1.1	BA.1.1_1	CO-CDPHE-2102653335|EPI_ISL_10570776|USA|BA.1.1.18|2022-01-10	93,68
EPI_ISL_10570905	BA.1	BA.1.15	BA.1.15	CO-CDPHE-2102652900|EPI_ISL_10570905|USA|BA.1|2022-01-12	84,85
EPI_ISL_10572027	BA.1.1	BA.1.1	BA.1.1_3	QEUH-3858888|EPI_ISL_10572027|United Kingdom|BA.1.1|2022-02-23	99,64
EPI_ISL_10572443	BA.1.1	BA.1.1	BA.1.1_3	QEUH-3832C1F|EPI_ISL_10572443|United Kingdom|BA.1.1|2022-02-23	99,64
EPI_ISL_10573109	BA.1.1	BA.1.1	BA.1.1_3	QEUH-384EA16|EPI_ISL_10573109|United Kingdom|BA.1.1|2022-02-23	99,64
EPI_ISL_10573256	BA.2	BA.2	BA.2_1	QEUH-383ADC9|EPI_ISL_10573256|United Kingdom|BA.2|2022-02-23	99,81
EPI_ISL_10574133	BA.2	BA.2	BA.2_1	QEUH-3845FD4|EPI_ISL_10574133|United Kingdom|BA.2|2022-02-23	99,81
EPI_ISL_10574526	BA.2	A	A_14	QEUH-38423EA|EPI_ISL_10574526|United Kingdom|BA.2|2022-02-22	99,75
EPI_ISL_10575521	BA.1.1.18	BA.1.1	BA.1.1_2	SC-CDC-MMB13987525|EPI_ISL_10575521|USA|BA.1.1.18|2022-01-31	93,34
EPI_ISL_10575735	BA.1.1	BA.1	BA.1_4	GA-CDC-MMB14077163|EPI_ISL_10575735|USA|BA.1.1|2022-02-01	91,22
EPI_ISL_10577732	BA.2.9	A	A_17	DHSC-CYNCAE1|EPI_ISL_10577732|United Kingdom|BA.2.9|2022-02-16	99,05
EPI_ISL_10579148	BA.1.10	BA.1.15	BA.1.15	PHEC-YYNI6T1|EPI_ISL_10579148|United Kingdom|BA.1.10|2022-02-06	92,27
EPI_ISL_10580010	BA.1.15.1	BA.1.15.1	BA.1.15.1	PHEC-YYNZIY4|EPI_ISL_10580010|United Kingdom|BA.1.15.1|2022-01-09	99,60
EPI_ISL_10580057	BA.1	BA.1	BA.1_4	PHEC-YYNZM19|EPI_ISL_10580057|United Kingdom|BA.1|2022-01-22	96,76
EPI_ISL_10580957	BA.2	BA.2	BA.2_1	LB-R00062-S155|EPI_ISL_10580957|Austria|BA.2|2022-02-17	97,00
EPI_ISL_10584793	BA.1.17.2	BA.1.17.2	BA.1.17.2	PHWC-PEPKCN|EPI_ISL_10584793|United Kingdom|BA.1.17.2|2022-02-08	99,64
EPI_ISL_10584838	BA.1.1.13	BA.1.1	BA.1.1_2	PHWC-PEPM5N|EPI_ISL_10584838|United Kingdom|BA.1.1.13|2022-02-08	99,64
EPI_ISL_10584883	BA.2	A	A_17	DCGC-395877|EPI_ISL_10584883|Denmark|BA.2|2022-01-20	99,81
EPI_ISL_10585714	BA.1.1	BA.1.1	BA.1.1_3	DC-Curative-234304|EPI_ISL_10585714|USA|BA.1.1|2022-02-18	83,29
EPI_ISL_10586030	BA.1.15	A	A_1	TX-Curative-267104|EPI_ISL_10586030|USA|BA.1.15|2022-02-18	69,89
EPI_ISL_10587284	BA.2	A	A_17	DCGC-396517|EPI_ISL_10587284|Denmark|BA.2|2022-02-23	99,81
EPI_ISL_10588498	BA.2	A	A_14	PHWC-PEX37C|EPI_ISL_10588498|United Kingdom|BA.2|2022-02-09	99,81
EPI_ISL_10588614	BA.1.17.2	BA.1.17.2	BA.1.17.2	PHWC-PEXBIW|EPI_ISL_10588614|United Kingdom|BA.1.17.2|2022-02-09	99,64
EPI_ISL_10588905	BA.1.17.2	BA.1	BA.1_2	30273|EPI_ISL_10588905|Croatia|BA.1.17.2|2022-02-10	99,64
EPI_ISL_10589185	BA.1.1	BA.1.1	BA.1.1_3	PHWC-PEXR8Q|EPI_ISL_10589185|United Kingdom|BA.1.1|2022-02-09	99,64
EPI_ISL_10589745	BA.1.1	BA.1.1	BA.1.1_2	30663|EPI_ISL_10589745|Croatia|BA.1.1|2022-02-08	99,64
EPI_ISL_10590485	BA.2.36	BA.2	BA.2_2	ULB-IBC_CV8434230832|EPI_ISL_10590485|Belgium|BA.2.36|2022-02-24	99,81
EPI_ISL_10590523	BA.1	BA.1	BA.1_4	NRL_s633|EPI_ISL_10590523|Czech Republic|BA.1|2022-01-28	99,39

EPI_ISL_10591304	BA.1.15	BA.1.15	BA.1.15	AZ-ASU48456|EPI_ISL_10591304|USA|BA.1.15|2022-01-07	81,64
EPI_ISL_10591843	BA.1.17.2	BA.1	BA.1_4	WSSEGorzow-22S1670|EPI_ISL_10591843|Poland|BA.1.17.2|2022-02-13	94,79
EPI_ISL_10593312	BA.2	BA.1	BA.1_4	21789151P|EPI_ISL_10593312|Sweden|BA.2|2022-02-14	95,94
EPI_ISL_10594170	BA.1.18	BA.1.15	BA.1.15	NOR-HMN-22022160314|EPI_ISL_10594170|France|BA.1.18|2022-02-07	90,15
EPI_ISL_10597761	BA.2	BA.1	BA.1_4	21776836P|EPI_ISL_10597761|Sweden|BA.2|2022-02-07	95,94
EPI_ISL_10597782	BA.1	BA.1	BA.1_4	DA10562727|EPI_ISL_10597782|Sweden|BA.1|2022-02-07	96,64
EPI_ISL_10597961	BA.2	BA.1	BA.1_4	21769118P|EPI_ISL_10597961|Sweden|BA.2|2022-02-07	95,94
EPI_ISL_10598024	BA.1.1.1	BA.1	BA.1_4	261754461512|EPI_ISL_10598024|Sweden|BA.1.1.1|2022-02-05	95,73
EPI_ISL_10598521	BA.2.9	BA.1	BA.1_4	22M005863|EPI_ISL_10598521|Sweden|BA.2.9|2022-01-24	95,94
EPI_ISL_10598988	BA.2	BA.1	BA.1_4	10390342|EPI_ISL_10598988|Sweden|BA.2|2022-02-04	95,94
EPI_ISL_10599476	BA.1.1	BA.1.1	BA.1.1_2	WI-CDC-ASC210861688|EPI_ISL_10599476|USA|BA.1.1|2022-02-18	96,79
EPI_ISL_10599570	BA.1.1	BA.1.1	BA.1.1_2	KY-CDC-ASC210749423|EPI_ISL_10599570|USA|BA.1.1|2022-02-18	96,76
EPI_ISL_10600304	BA.1.1	BA.1	BA.1_4	22-517950|EPI_ISL_10600304|Sweden|BA.1.1|2022-01-25	95,73
EPI_ISL_10601806	BA.1.1.18	BA.1.1	BA.1.1_2	CO-CDPHE-2102653150|EPI_ISL_10601806|USA|BA.1.1.18|2022-01-12	96,79
EPI_ISL_10602068	BA.1.1	BA.1.1	BA.1.1_2	AZ-TG1247127|EPI_ISL_10602068|USA|BA.1.1|2022-02-07	99,62
EPI_ISL_10602157	BA.2.9	BA.1	BA.1_4	101785058|EPI_ISL_10602157|Sweden|BA.2.9|2022-01-31	95,02
EPI_ISL_10603958	BA.1.1	BA.1.1	BA.1.1_2	FL-CDC-ASC210748886|EPI_ISL_10603958|USA|BA.1.1|2022-02-19	99,64
EPI_ISL_10605329	BA.1.1	BA.1.1	BA.1.1_2	CA-CDC-ASC210749011|EPI_ISL_10605329|USA|BA.1.1|2022-02-19	97,27
EPI_ISL_10606079	BA.1.1	BA.1	BA.1_4	EP000609461|EPI_ISL_10606079|Sweden|BA.1.1|2022-01-18	95,73
EPI_ISL_10606727	BA.1.1	BA.1.1	BA.1.1_3	TX-HMH-MCoV-76182|EPI_ISL_10606727|USA|BA.1.1|2022-01-06	99,64
EPI_ISL_10608928	BA.1.1	BA.1.1	BA.1.1_3	TX-HMH-MCoV-77402|EPI_ISL_10608928|USA|BA.1.1|2022-01-07	99,64
EPI_ISL_10609670	BA.1.1.1	BA.1	BA.1_4	2265362745|EPI_ISL_10609670|Sweden|BA.1.1.1|2022-01-14	96,66
EPI_ISL_10610655	BA.2	BA.1	BA.1_4	10326149|EPI_ISL_10610655|Sweden|BA.2|2022-01-10	95,94
EPI_ISL_10611409	BA.1	BA.1.15	BA.1.15	PEP123446|EPI_ISL_10611409|Sweden|BA.1|2021-12-30	97,69
EPI_ISL_10615716	BA.1	BA.1.15	BA.1.15	4062503464|EPI_ISL_10615716|Sweden|BA.1|2021-12-27	95,71
EPI_ISL_10616057	BA.1.17	BA.1.15	BA.1.15	10278971|EPI_ISL_10616057|Sweden|BA.1.17|2021-12-26	96,66
EPI_ISL_10616567	BA.1.17.2	BA.1	BA.1_4	4021058843VN|EPI_ISL_10616567|Sweden|BA.1.17.2|2021-12-22	95,73
EPI_ISL_10616576	BA.1.15	BA.1.15	BA.1.15	4021058832VN|EPI_ISL_10616576|Sweden|BA.1.15|2021-12-22	82,29
EPI_ISL_10620363	BA.1.20	BA.1	BA.1_3	FL-Curative-296883|EPI_ISL_10620363|USA|BA.1.20|2022-02-01	83,15
EPI_ISL_10620712	BA.1.15	BA.1.15	BA.1.15	2927332764|EPI_ISL_10620712|Sweden|BA.1.15|2021-12-12	95,73
EPI_ISL_10624664	BA.1.1	BA.1.1	BA.1.1_2	VA-CDC-QDX33873507|EPI_ISL_10624664|USA|BA.1.1|2022-02-12	99,64
EPI_ISL_10625024	BA.1.1	BA.1.1	BA.1.1_2	AZ-CDC-QDX33994980|EPI_ISL_10625024|USA|BA.1.1|2022-02-15	99,64
EPI_ISL_10626561	BA.2	BA.2	BA.2_1	22MV0977|EPI_ISL_10626561|New Zealand|BA.2|2022-02-14	90,52
EPI_ISL_10628394	BA.1.18	BA.1.15	BA.1.15	DHSC-CYNAIA5|EPI_ISL_10628394|United Kingdom|BA.1.18|2022-02-17	94,39
EPI_ISL_10629470	BA.1.1.1	BA.1.1	BA.1.1_3	DHSC-CYYWR3W|EPI_ISL_10629470|United Kingdom|BA.1.1.1|2022-02-17	99,60
EPI_ISL_10630652	BA.1.1	BA.1.1	BA.1.1_3	PHEC-5V077ZF2|EPI_ISL_10630652|United Kingdom|BA.1.1|2022	84,05
EPI_ISL_10630824	BA.2.10	BA.2	BA.2_1	KA-CBR-1602DK076|EPI_ISL_10630824|India|BA.2.10|2022-02-02	99,81
EPI_ISL_10632947	BA.1.17.2	BA.1.17.2	BA.1.17.2	PHEC-YYNAE5Q|EPI_ISL_10632947|United Kingdom|BA.1.17.2|2022-01-06	97,42
EPI_ISL_10633030	BA.1	BA.1	BA.1_3	PHEC-YYNAHYT|EPI_ISL_10633030|United Kingdom|BA.1|2022-02-07	99,60
EPI_ISL_10635245	BA.2	A	A_17	DCGC-398033|EPI_ISL_10635245|Denmark|BA.2|2022-02-24	99,81
EPI_ISL_10635726	BA.2.10	BA.2.10	BA.2.10	g0221223|EPI_ISL_10635726|Cambodia|BA.2.10|2022-02-22	99,81
EPI_ISL_10637453	BA.2	BA.2	BA.2_1	DCGC-399020|EPI_ISL_10637453|Denmark|BA.2|2022-02-25	99,81
EPI_ISL_10637674	BA.2.9	A	A_17	DCGC-399165|EPI_ISL_10637674|Denmark|BA.2.9|2022-02-23	99,81
EPI_ISL_10639597	BA.1.1	BA.1.1	BA.1.1_2	NOR-20575043|EPI_ISL_10639597|France|BA.1.1|2022-02-05	90,98
EPI_ISL_10641105	BA.2	A	A_14	QEUH-385831E|EPI_ISL_10641105|United Kingdom|BA.2|2022-02-23	99,81
EPI_ISL_10644144	BA.2	BA.2	BA.2_1	MILK-3866E76|EPI_ISL_10644144|United Kingdom|BA.2|2022-02-24	93,95
EPI_ISL_10645795	BA.1.15	BA.1.15	BA.1.15	SK-RRPL-365024|EPI_ISL_10645795|Canada|BA.1.15|2021-12-15	99,64
EPI_ISL_10646065	BA.2	BA.2	BA.2_1	BL-ETHZ-36337266|EPI_ISL_10646065|Switzerland|BA.2|2022-02-13	99,81
EPI_ISL_10646415	BA.1	BA.1.19	BA.1.19	NICD-N34390|EPI_ISL_10646415|South Africa|BA.1|2021-12-30	97,06
EPI_ISL_10647927	BA.1.17.2	BA.1	BA.1_3	PAC-IHU-66408_Nova1|EPI_ISL_10647927|France|BA.1.17.2|2022-02	68,92
EPI_ISL_10648335	BA.1.1.1	BA.1.1	BA.1.1_3	PAC-IHU-66707_Nova1|EPI_ISL_10648335|France|BA.1.1.1|2022-02	90,19
EPI_ISL_10648996	BA.1.1.1	BA.1.1	BA.1.1_3	PAC-IHU-66912_Nova1|EPI_ISL_10648996|France|BA.1.1.1|2022-02	85,94
EPI_ISL_10650263	BA.1.1.1	BA.1.1	BA.1.1_2	PAC-IHU-64502_Nova1RZ|EPI_ISL_10650263|France|BA.1.1.1|2022-02	95,57
EPI_ISL_10650354	BA.2	BA.2.74	BA.2.74	PAC-IHU-64601_Nova1RZ|EPI_ISL_10650354|France|BA.2|2022-02	94,56
EPI_ISL_10651105	BA.1.1	BA.1.1	BA.1.1_2	NY-NYULH5949|EPI_ISL_10651105|USA|BA.1.1|2022-02-13	94,79
EPI_ISL_10651806	BA.1	BA.1.15	BA.1.15	AB-ABPHL-54035|EPI_ISL_10651806|Canada|BA.1|2022-02-01	99,64
EPI_ISL_10652754	BA.1.1	BA.1.1	BA.1.1_2	VA-CDC-ASC210822517|EPI_ISL_10652754|USA|BA.1.1|2022-02-21	96,93
EPI_ISL_10655209	BA.1.1	BA.1.1	BA.1.1_2	ME-HETL-J11952|EPI_ISL_10655209|USA|BA.1.1|2022-01-17	99,64
EPI_ISL_10655532	BA.1.1.18	BA.1.1	BA.1.1_3	GA-CDC-MMB14077927|EPI_ISL_10655532|USA|BA.1.1.18|2022-02-01	99,64
EPI_ISL_10660175	BA.1.1.2	BA.1.1	BA.1.1_3	PG-191597|EPI_ISL_10660175|Japan|BA.1.1.2|2022-01-15	99,64
EPI_ISL_10660523	BA.1.1.2	BA.1.1	BA.1.1_2	PG-194511|EPI_ISL_10660523|Japan|BA.1.1.2|2022-01-14	99,64
EPI_ISL_10661132	BA.2.10	A	A_14	PY-ILBS-WGS3846|EPI_ISL_10661132|India|BA.2.10|2022-01-15	97,65
EPI_ISL_10662077	BA.1.1.2	BA.1.1	BA.1.1_3	PG-199500|EPI_ISL_10662077|Japan|BA.1.1.2|2022-01-18	99,64
EPI_ISL_10662691	BA.1.1.2	BA.1.1	BA.1.1_2	PG-185613|EPI_ISL_10662691|Japan|BA.1.1.2|2022-01-17	99,64
EPI_ISL_10662718	BA.1.1	BA.1.1	BA.1.1_2	PG-185640|EPI_ISL_10662718|Japan|BA.1.1|2022-01-13	99,64
EPI_ISL_10664639	BA.1.1.2	BA.1.1	BA.1.1_3	PG-194767|EPI_ISL_10664639|Japan|BA.1.1.2|2022-01-16	99,64
EPI_ISL_10665465	BA.1.1.2	BA.1.1	BA.1.1_3	PG-189693|EPI_ISL_10665465|Japan|BA.1.1.2|2022-01-19	99,64
EPI_ISL_10666051	BC.1	BA.1.1	BA.1.1_2	PG-188809|EPI_ISL_10666051|Japan|BC.1|2022-01-26	99,64
EPI_ISL_10668693	BA.1.20	BA.1.15	BA.1.15	PG-187673|EPI_ISL_10668693|Japan|BA.1.20|2022-01-24	99,64
EPI_ISL_10669712	BA.1.1.2	BA.1.1	BA.1.1_3	PG-189009|EPI_ISL_10669712|Japan|BA.1.1.2|2022-01-23	99,64
EPI_ISL_10670199	BA.2	A	A_14	DHSC-CYDU9WN|EPI_ISL_10670199|United Kingdom|BA.2|2022-02-18	99,81
EPI_ISL_10672455	BA.1.1	BA.1.1	BA.1.1_2	PHEC-YYN4FI3|EPI_ISL_10672455|United Kingdom|BA.1.1|2022-02-13	96,70
EPI_ISL_10672626	BA.1.1	BA.1.1	BA.1.1_3	PHEC-YYN4KD3|EPI_ISL_10672626|United Kingdom|BA.1.1|2022-02-16	96,58
EPI_ISL_10672801	BA.1.17	AY.4.10	AY.4.10	NSW-ICPMR-21361|EPI_ISL_10672801|Australia|BA.1.17|2021-12-22	85,38
EPI_ISL_10673016	BC.1	BA.1.1	BA.1.1_2	PG-182487|EPI_ISL_10673016|Japan|BC.1|2022-01-13	99,64
EPI_ISL_10675345	BA.2	A	A_17	PHWC-PEOTXQ|EPI_ISL_10675345|United Kingdom|BA.2|2022-02-14	99,81
EPI_ISL_10677601	BA.1.1.2	BA.1.1	BA.1.1_3	PG-192603|EPI_ISL_10677601|Japan|BA.1.1.2|2022-01-26	99,64
EPI_ISL_10677763	BA.2.9	A	A_17	DCGC-400490|EPI_ISL_10677763|Denmark|BA.2.9|2022-02-26	99,96
EPI_ISL_10679408	BA.1.1.2	BA.1.1	BA.1.1_3	PG-182188|EPI_ISL_10679408|Japan|BA.1.1.2|2022-01-06	99,64
EPI_ISL_10679784	BA.2	A	A_17	DCGC-401776|EPI_ISL_10679784|Denmark|BA.2|2022-02-27	99,81
EPI_ISL_10681204	BA.1.1.1	BA.1.1	BA.1.1_1	MBLG-CTMAPF22479280|EPI_ISL_10681204|Belgium|BA.1.1.1|2022-02-25	99,37
EPI_ISL_10682075	BA.1.1.2	BA.1.1	BA.1.1_3	PG-189864|EPI_ISL_10682075|Japan|BA.1.1.2|2022-01-12	99,62
EPI_ISL_10683609	BA.1	AY.43	AY.43_1	IMBA_IMP-1590_G08|EPI_ISL_10683609|Austria|BA.1|2022-02-21	46,82
EPI_ISL_10685051	BA.1.1.2	BA.1.1	BA.1.1_1	PG-190179|EPI_ISL_10685051|Japan|BA.1.1.2|2022-01-17	99,64
EPI_ISL_10686732	BA.2	A	A_14	MILK-386A249|EPI_ISL_10686732|United Kingdom|BA.2|2022-02-25	99,81
EPI_ISL_10686968	BA.2	BA.2	BA.2_1	QEUH-38692FF|EPI_ISL_10686968|United Kingdom|BA.2|2022-02-25	99,81
EPI_ISL_10687144	BA.1.17	BA.1.15	BA.1.15	MILK-3869ECE|EPI_ISL_10687144|United Kingdom|BA.1.17|2022-02-25	99,64
EPI_ISL_10687779	BA.2	A	A_14	HSLL-3883435|EPI_ISL_10687779|United Kingdom|BA.2|2022-02-26	99,81
EPI_ISL_10689262	BA.1.1.2	BA.1.1	BA.1.1_1	PG-177780|EPI_ISL_10689262|Japan|BA.1.1.2|2022-01-10	99,64
EPI_ISL_10689458	BA.1.1.2	BA.1.1	BA.1.1_3	PG-181823|EPI_ISL_10689458|Japan|BA.1.1.2|2022-01-14	99,64
EPI_ISL_10689755	BA.1.1.2	BA.1.1	BA.1.1_3	PG-186907|EPI_ISL_10689755|Japan|BA.1.1.2|2022-01-13	99,64
EPI_ISL_10694613	BA.2	BA.2	BA.2_2	GE-RUMC-000534|EPI_ISL_10694613|Netherlands|BA.2|2022-02-21	93,17
EPI_ISL_10695226	BA.1.15	BA.1.15	BA.1.15	AZ-ASU48385|EPI_ISL_10695226|USA|BA.1.15|2022-01-08	90,98
EPI_ISL_10695272	BA.1.1	BA.1.1	BA.1.1_3	PG-185177|EPI_ISL_10695272|Japan|BA.1.1|2022-01-20	99,64
EPI_ISL_10695431	BA.1.1	BA.1.1	BA.1.1_2	NY-UB-ECMC-00213|EPI_ISL_10695431|USA|BA.1.1|2021-12-28	90,92
EPI_ISL_10697698	BA.1.1.18	BA.1.1	BA.1.1_2	KS-KSU-1322|EPI_ISL_10697698|USA|BA.1.1.18|2022-01-21	99,69
EPI_ISL_10699247	BA.1.1	BA.1.1	BA.1.1_2	CA-CDC-STM-8K2AXSXSJ|EPI_ISL_10699247|USA|BA.1.1|2022-02-17	99,29
EPI_ISL_10699338	BA.1.15	BA.1.15	BA.1.15	TX-CDC-STM-8WVWQZ88G|EPI_ISL_10699338|USA|BA.1.15|2022-02-17	99,64
EPI_ISL_10699656	BA.1	BA.1.15	BA.1.15	BA_22_00004898|EPI_ISL_10699656|Slovakia|BA.1|2022-01-12	92,67
EPI_ISL_10701886	BA.1.1	BA.1.1	BA.1.1_2	CA-CDC-STM-DYHAD5NBK|EPI_ISL_10701886|USA|BA.1.1|2022-02-22	99,64
EPI_ISL_10701889	BA.1.1	BA.1.1	BA.1.1_2	CA-CDC-STM-KHVNH54FS|EPI_ISL_10701889|USA|BA.1.1|2022-02-22	99,64

EPI_ISL_10701932	BA.1.1.1	BA.1.1	BA.1.1_3	BA_22_00006773|EPI_ISL_10701932|Slovakia|BA.1.1.1|2022-01-30	91,59
EPI_ISL_10703158	BA.1.1	BA.1.1	BA.1.1_2	TLA_InDRE_FB10486_E29315293755_S12207|EPI_ISL_10703158|Mexico|BA.1.1|2022-02-03	99,64
EPI_ISL_10709629	BA.2	BA.2	BA.2_1	BRBR-3897A96|EPI_ISL_10709629|United Kingdom|BA.2|2022-02-25	99,81
EPI_ISL_10711406	BA.2	A	A_14	BRBR-387D155|EPI_ISL_10711406|United Kingdom|BA.2|2022-02-26	99,81
EPI_ISL_10711549	BA.2	A	A_14	BRBR-387CF3E|EPI_ISL_10711549|United Kingdom|BA.2|2022-02-26	99,77
EPI_ISL_10712608	BA.1.15	BA.1	BA.1_4	MA-CDCBI-CRSP_46YJMDKNSZJ6DITR|EPI_ISL_10712608|USA|BA.1.15|2022-02-22	99,64
EPI_ISL_10713409	BA.1.1	BA.1.1	BA.1.1_2	MA-CDCBI-CRSP_LCQO3QYZCNANAI6D|EPI_ISL_10713409|USA|BA.1.1|2022-02-16	88,78
EPI_ISL_10715068	BA.1.15	BA.1	BA.1_4	UT-UPHL-220226623346|EPI_ISL_10715068|USA|BA.1.15|2022-01-31	99,64
EPI_ISL_10715885	BA.1.1	BA.1.1	BA.1.1_2	CO-CDPHE-2102934140|EPI_ISL_10715885|USA|BA.1.1|2022-02-14	99,52
EPI_ISL_10718150	BA.2	BA.2	BA.2_1	OCC-HCL722000770401|EPI_ISL_10718150|France|BA.2|2022-02-14	99,81
EPI_ISL_10718767	BA.2	A	A_17	NAQ-HCL722000872001|EPI_ISL_10718767|France|BA.2|2022-02-21	99,81
EPI_ISL_10718937	BA.1.1.1	BA.1.1	BA.1.1_3	ARA-HCL722000893101|EPI_ISL_10718937|France|BA.1.1.1|2022-02-21	99,64
EPI_ISL_10719647	BA.1.1	BA.1.1	BA.1.1_2	IL-CDC-LC0547737|EPI_ISL_10719647|USA|BA.1.1|2022-02-16	99,64
EPI_ISL_10722070	BA.1.1	BA.1.1	BA.1.1_2	UT-UPHL-220228714333|EPI_ISL_10722070|USA|BA.1.1|2022-02-04	99,64
EPI_ISL_10723728	BA.1.1	BA.1	BA.1_4	FL-UCF_NP_728|EPI_ISL_10723728|USA|BA.1.1|2022-01-27	80,31
EPI_ISL_10723906	BA.1	BA.1	BA.1_1	KY-GD_02110|EPI_ISL_10723906|USA|BA.1|2021-12-19	99,69
EPI_ISL_10724138	BA.1.1	BA.1.1	BA.1.1_2	SN-NIHRD-WGS.22.03216|EPI_ISL_10724138|Indonesia|BA.1.1|2022-02-08	99,64
EPI_ISL_10724400	BA.1.1	BA.1.1	BA.1.1_2	GE-DIA-161347798301|EPI_ISL_10724400|Switzerland|BA.1.1|2022-02-21	99,54
EPI_ISL_10727675	BA.2	A	A_14	MILK-388B9B6|EPI_ISL_10727675|United Kingdom|BA.2|2022-02-27	99,18
EPI_ISL_10728039	BA.2.1	A	A_14	BRBR-389EA26|EPI_ISL_10728039|United Kingdom|BA.2.1|2022-02-25	99,81
EPI_ISL_10728808	BA.1.1	BA.1.1	BA.1.1_2	SC-CDC-QDX34073188|EPI_ISL_10728808|USA|BA.1.1|2022-02-19	99,64
EPI_ISL_10728933	BA.1	BA.1	BA.1_4	PZH-UMB-17674|EPI_ISL_10728933|Poland|BA.1|2022-02-22	99,64
EPI_ISL_10729325	BA.1.15	BA.1	BA.1_4	TCH-017|EPI_ISL_10729325|Chad|BA.1.15|2021-12-30	89,20
EPI_ISL_10729665	BA.2.10	BA.2	BA.2_1	PY-SEQ_13074_S341_R1_001|EPI_ISL_10729665|India|BA.2.10|2022-02-05	99,81
EPI_ISL_10729973	BA.1	BA.1	BA.1_3	HSGM-G3951|EPI_ISL_10729973|Turkey|BA.1|2022-02-23	99,64
EPI_ISL_10730189	BA.1	BA.1	BA.1_4	HSGM-G3806|EPI_ISL_10730189|Turkey|BA.1|2022-02-23	99,64
EPI_ISL_10730842	BA.2	A	A_17	DCGC-402169|EPI_ISL_10730842|Denmark|BA.2|2022-02-27	99,81
EPI_ISL_10731197	BA.2	A	A_17	DCGC-402529|EPI_ISL_10731197|Denmark|BA.2|2022-02-24	99,98
EPI_ISL_10731234	BA.2	BA.2	BA.2_1	DCGC-402567|EPI_ISL_10731234|Denmark|BA.2|2022-02-27	99,79
EPI_ISL_10731260	BA.2	BA.2	BA.2_1	DCGC-402593|EPI_ISL_10731260|Denmark|BA.2|2022-02-24	81,97
EPI_ISL_10731567	BA.2.9	A	A_17	DCGC-402902|EPI_ISL_10731567|Denmark|BA.2.9|2022-02-27	99,77
EPI_ISL_10733310	BA.2.9	A	A_17	DCGC-404506|EPI_ISL_10733310|Denmark|BA.2.9|2022-02-27	99,81
EPI_ISL_10734074	BA.2	A	A_14	MILK-388CFB3|EPI_ISL_10734074|United Kingdom|BA.2|2022-02-26	99,81
EPI_ISL_10736360	BA.2	A	A_14	MILK-38B444A|EPI_ISL_10736360|United Kingdom|BA.2|2022-02-28	99,81
EPI_ISL_10738273	BA.1.1	BA.1	BA.1_2	NW-RKI-I-570728|EPI_ISL_10738273|Germany|BA.1.1|2022-02-15	99,33
EPI_ISL_10739459	BA.1	BA.1	BA.1_3	BW-RKI-I-571518|EPI_ISL_10739459|Germany|BA.1|2022-02-08	99,60
EPI_ISL_10739884	BA.1.1	BA.1.1	BA.1.1_3	WD-NVRL-S22IRL00087284|EPI_ISL_10739884|Ireland|BA.1.1|2022-01-25	93,74
EPI_ISL_10739955	BA.1.15	BA.1	BA.1_3	SN-RKI-I-571606|EPI_ISL_10739955|Germany|BA.1.15|2022-02-11	99,58
EPI_ISL_10740176	BA.1.17.2	BA.1.17.2	BA.1.17.2	RP-RKI-I-571750|EPI_ISL_10740176|Germany|BA.1.17.2|2022-02-11	93,11
EPI_ISL_10740233	BA.2.4	A	A_14	2261|EPI_ISL_10740233|Singapore|BA.2.4|2022-02-28	99,81
EPI_ISL_10740290	BA.2	A	A_14	2316|EPI_ISL_10740290|Singapore|BA.2|2022-02-24	99,79
EPI_ISL_10742447	BA.1.1	BA.1.1	BA.1.1_2	DHSC-CYBFS6S|EPI_ISL_10742447|United Kingdom|BA.1.1|2022-02-20	99,64
EPI_ISL_10742505	BA.2	A	A_14	DHSC-CYBGZ45|EPI_ISL_10742505|United Kingdom|BA.2|2022-02-20	99,81
EPI_ISL_10742937	BA.2	A	A_14	DHSC-CYBOO8W|EPI_ISL_10742937|United Kingdom|BA.2|2022-02-19	99,81
EPI_ISL_10744273	BA.2	BA.2	BA.2_1	NW-RKI-I-575090|EPI_ISL_10744273|Germany|BA.2|2022-02-19	99,81
EPI_ISL_10745039	BA.2	BA.2	BA.2_1	DHSC-CYDQXU9|EPI_ISL_10745039|United Kingdom|BA.2|2022-02-20	99,81
EPI_ISL_10745337	BA.1.1	BA.1.1	BA.1.1_2	LTU000_VULSK_220207120116|EPI_ISL_10745337|Lithuania|BA.1.1|2022-02-07	99,60
EPI_ISL_10745537	BA.1.1	BA.1.1	BA.1.1_2	LTU000_NVSPL_MB12240|EPI_ISL_10745537|Lithuania|BA.1.1|2022-02-07	93,36
EPI_ISL_10746314	BA.2	A	A_14	KA-RFNB-5882|EPI_ISL_10746314|India|BA.2|2022-02	99,81
EPI_ISL_10746337	BA.2.10	A	A_14	KA-RFNB-5936|EPI_ISL_10746337|India|BA.2.10|2022-02	99,81
EPI_ISL_10746358	BA.2.10	BA.2.10	BA.2.10	KA-RFNB-5988|EPI_ISL_10746358|India|BA.2.10|2022-02	99,81
EPI_ISL_10750434	BA.1.1.12	BA.1.1	BA.1.1_3	DHSC-CYY7JUJ|EPI_ISL_10750434|United Kingdom|BA.1.1.12|2022-02-20	99,64
EPI_ISL_10751382	BA.2	A	A_14	DHSC-CYYMBI1|EPI_ISL_10751382|United Kingdom|BA.2|2022-02-20	99,81
EPI_ISL_10754304	BA.1.17	BA.1	BA.1_4	BY-RKI-I-578857|EPI_ISL_10754304|Germany|BA.1.17|2022-02-21	99,62
EPI_ISL_10755989	BA.1.1	BA.1.1	BA.1.1_3	BW-RKI-I-579911|EPI_ISL_10755989|Germany|BA.1.1|2022-02-03	99,64
EPI_ISL_10757005	BA.2	A	A_14	BW-RKI-I-580552|EPI_ISL_10757005|Germany|BA.2|2022-02-03	98,51
EPI_ISL_10758022	BA.1.18	BA.1	BA.1_4	NOR-HMN-22022230337|EPI_ISL_10758022|France|BA.1.18|2022-02-14	99,22
EPI_ISL_10758332	BA.1.1	BA.1.1	BA.1.1_3	PHEC-5V0A4Z61|EPI_ISL_10758332|United Kingdom|BA.1.1|2022	99,62
EPI_ISL_10758346	BA.1.1	BA.1.1	BA.1.1_2	BW-RKI-I-581083|EPI_ISL_10758346|Germany|BA.1.1|2022-02-03	99,64
EPI_ISL_10758906	BA.1.1	BA.1.1	BA.1.1_2	SP-IB_151964|EPI_ISL_10758906|Brazil|BA.1.1|2022-02-07	99,64
EPI_ISL_10761334	BA.1.1	BA.1.1	BA.1.1_2	NY-PRL-2022_0227_00F19|EPI_ISL_10761334|USA|BA.1.1|2022-02-26	99,52
EPI_ISL_10762071	BA.1.1	BA.1.1	BA.1.1_2	SL-RKI-I-581964|EPI_ISL_10762071|Germany|BA.1.1|2022-01-19	99,60
EPI_ISL_10763370	BA.1.1	BA.1.1	BA.1.1_2	S22B403|EPI_ISL_10763370|Lithuania|BA.1.1|2022-02-14	99,64
EPI_ISL_10763493	BA.1.1.1	BA.1.1	BA.1.1_3	SN-RKI-I-582335|EPI_ISL_10763493|Germany|BA.1.1.1|2022-02-11	95,44
EPI_ISL_10766571	BA.1	BA.1	BA.1_4	PHEC-YYN787B|EPI_ISL_10766571|United Kingdom|BA.1|2022-01-27	99,62
EPI_ISL_10767031	BA.1.1	BA.1.1	BA.1.1_3	PHEC-YYN7HR9|EPI_ISL_10767031|United Kingdom|BA.1.1|2022-02-21	92,23
EPI_ISL_10770250	BA.1.15	BA.1.15	BA.1.15	PHWC-PE1XOJ|EPI_ISL_10770250|United Kingdom|BA.1.15|2022-02-12	97,65
EPI_ISL_10770959	BA.1.1	BA.1.1	BA.1.1_2	BA_22_00008700|EPI_ISL_10770959|Slovakia|BA.1.1|2022-02-01	96,60
EPI_ISL_10771715	BA.1.1.15	BA.1.1	BA.1.1_3	PHWC-PETMKH|EPI_ISL_10771715|United Kingdom|BA.1.1.15|2022-02-01	99,33
EPI_ISL_10772444	BA.2	A	A_14	PHWC-PEUGOO|EPI_ISL_10772444|United Kingdom|BA.2|2022-02-12	99,81
EPI_ISL_10773912	BA.1.18	BA.1	BA.1_2	SL-SU-10500034|EPI_ISL_10773912|Germany|BA.1.18|2022-02-18	95,95
EPI_ISL_10773914	BA.2	A	A_4	SL-SU-10500045|EPI_ISL_10773914|Germany|BA.2|2022-02-18	99,79
EPI_ISL_10775216	BA.1.1	BA.1.1	BA.1.1_3	UT-CDC-2-5632287|EPI_ISL_10775216|USA|BA.1.1|2021-12-14	99,64
EPI_ISL_10779677	BA.1.1	BA.1.1	BA.1.1_2	CA-CDC-FG-286215|EPI_ISL_10779677|USA|BA.1.1|2022-02-23	99,64
EPI_ISL_10780864	BA.1	BA.1	BA.1_3	CA-LACPHL-AF07217|EPI_ISL_10780864|USA|BA.1|2022-01-23	99,64
EPI_ISL_10783519	BA.1	BA.1	BA.1_4	BC-BCCDC-353785|EPI_ISL_10783519|Canada|BA.1|2022-01-29	99,64
EPI_ISL_10783545	BA.1.1	BA.1.1	BA.1.1_2	BC-BCCDC-353816|EPI_ISL_10783545|Canada|BA.1.1|2022-01-29	99,64
EPI_ISL_10784196	BA.1	BA.1.15	BA.1.15	BC-BCCDC-355088|EPI_ISL_10784196|Canada|BA.1|2022-01-30	99,64
EPI_ISL_10784245	BA.1.17.2	BA.1.17.2	BA.1.17.2	BC-BCCDC-355203|EPI_ISL_10784245|Canada|BA.1.17.2|2022-01-25	99,64
EPI_ISL_10784435	BA.1.1	BA.1.1	BA.1.1_2	BC-BCCDC-355659|EPI_ISL_10784435|Canada|BA.1.1|2022-01-31	99,64
EPI_ISL_10784532	BA.1.1	BA.1.1	BA.1.1_2	IN-GD-022422-57|EPI_ISL_10784532|USA|BA.1.1|2022-02-19	99,64
EPI_ISL_10786844	BA.2	A	A_17	01_SE100_22CS502619|EPI_ISL_10786844|Sweden|BA.2|2022-02-22	99,81
EPI_ISL_10787461	BA.1.1	BA.1.1	BA.1.1_3	BC-BCCDC-359153|EPI_ISL_10787461|Canada|BA.1.1|2022-02-05	99,64
EPI_ISL_10789560	BA.1.1	BA.1.1	BA.1.1_3	RI-RISHL-E03804|EPI_ISL_10789560|USA|BA.1.1|2022-02-16	83,19
EPI_ISL_10791465	BA.1.1	BA.1.1	BA.1.1_2	PA-CDC-QDX34074069|EPI_ISL_10791465|USA|BA.1.1|2022-02-12	99,64
EPI_ISL_10791479	BA.1.1	BA.1.1	BA.1.1_2	PA-CDC-QDX34074150|EPI_ISL_10791479|USA|BA.1.1|2022-02-16	99,64
EPI_ISL_10792875	BA.1.1.18	BA.1.1	BA.1.1_3	NE-NPHL22-4725|EPI_ISL_10792875|USA|BA.1.1.18|2022-01-14	99,64
EPI_ISL_10793923	BA.1.1.18	BA.1.1	BA.1.1_2	AZ-ASU50237|EPI_ISL_10793923|USA|BA.1.1.18|2022-01-19	93,49
EPI_ISL_10795293	BA.2.9	A	A_17	MILK-38D0378|EPI_ISL_10795293|United Kingdom|BA.2.9|2022-03-01	99,81
EPI_ISL_10799599	BA.2	BA.2	BA.2_1	PLYM-38C036C|EPI_ISL_10799599|United Kingdom|BA.2|2022-02-28	99,81
EPI_ISL_10800088	BA.1.1	BA.1.1	BA.1.1_3	DHSC-CYBC64E|EPI_ISL_10800088|United Kingdom|BA.1.1|2022-02-22	99,64
EPI_ISL_10800198	BA.2	A	A_14	BRBR-38C2300|EPI_ISL_10800198|United Kingdom|BA.2|2022-03-01	99,33
EPI_ISL_10800334	BA.2	A	A_14	DHSC-CYBGR4Q|EPI_ISL_10800334|United Kingdom|BA.2|2022-02-22	99,81
EPI_ISL_10800480	BA.2.10	BA.2	BA.2_1	DHSC-CYBKCFJ|EPI_ISL_10800480|United Kingdom|BA.2.10|2022-02-22	99,81
EPI_ISL_10801330	BA.2	BA.2	BA.2_1	QEUH-38A0A8B|EPI_ISL_10801330|United Kingdom|BA.2|2022-02-27	99,81
EPI_ISL_10802287	BA.2	A	A_14	QEUH-388E5A0|EPI_ISL_10802287|United Kingdom|BA.2|2022-02-27	99,81
EPI_ISL_10802454	BA.2.8	BA.2	BA.2_1	QEUH-388E5DD|EPI_ISL_10802454|United Kingdom|BA.2.8|2022-02-27	99,81
EPI_ISL_10802682	BA.1.1	BA.1.1	BA.1.1_2	QEUH-387B7BF|EPI_ISL_10802682|United Kingdom|BA.1.1|2022-02-26	99,64
EPI_ISL_10803371	BA.2	BA.2	BA.2_1	QEUH-38807F3|EPI_ISL_10803371|United Kingdom|BA.2|2022-02-26	99,81
EPI_ISL_10804730	BA.2	A	A_17	NORT-YNNDKXC|EPI_ISL_10804730|United Kingdom|BA.2|2022	99,81
EPI_ISL_10805427	BA.1.1	BA.1.1	BA.1.1_3	31247|EPI_ISL_10805427|Croatia|BA.1.1|2022-02-16	99,64

EPI_ISL_10805460	BA.2	BA.2	BA.2_1	31283|EPI_ISL_10805460|Croatia|BA.2|2022-02-21	93,67
EPI_ISL_10806753	BA.2	A	A_17	DCGC-405549|EPI_ISL_10806753|Denmark|BA.2|2022-02-22	99,77
EPI_ISL_10807072	BA.1.1	BA.1	BA.1_4	RM-19757|EPI_ISL_10807072|Chile|BA.1.1|2022-02-01	90,35
EPI_ISL_10810663	BA.2	A	A_17	DCGC-408236|EPI_ISL_10810663|Denmark|BA.2|2022-02-26	99,81
EPI_ISL_10812839	BA.2	A	A_17	DCGC-409745|EPI_ISL_10812839|Denmark|BA.2|2022-02-26	99,98
EPI_ISL_10815635	BA.1.1	BA.1.1	BA.1.1_3	BY-MVP-000011258|EPI_ISL_10815635|Germany|BA.1.1|2022-01-24	99,64
EPI_ISL_10816148	BA.1.1	BA.1.1	BA.1.1_3	BY-MVP-000012303|EPI_ISL_10816148|Germany|BA.1.1|2022-02-11	99,64
EPI_ISL_10817145	BA.1.15	BA.1.15	BA.1.15	TX-HMH-MCoV-77802|EPI_ISL_10817145|USA|BA.1.15|2022-01-10	99,64
EPI_ISL_10817347	BA.1	BA.1.15	BA.1.15	TX-HMH-MCoV-78028|EPI_ISL_10817347|USA|BA.1|2022-01-10	99,64
EPI_ISL_10819035	BA.1	BA.1.15	BA.1.15	TX-HMH-MCoV-78591|EPI_ISL_10819035|USA|BA.1|2022-01-11	99,48
EPI_ISL_10819342	BA.1.15	BA.1.15	BA.1.15	TX-HMH-MCoV-78224|EPI_ISL_10819342|USA|BA.1.15|2022-01-10	99,64
EPI_ISL_10819846	BA.1.1	BA.1	BA.1_4	EMR-IZSLER-2022-065054-019-01|EPI_ISL_10819846|Italy|BA.1.1|2022-02-25	95,02
EPI_ISL_10819858	BA.1.15.1	BA.1.15	BA.1.15	EMR-IZSLER-2022-065054-005-01|EPI_ISL_10819858|Italy|BA.1.15.1|2022-02-25	93,59
EPI_ISL_10820632	BA.1.15	BA.1.15	BA.1.15	TX-HMH-MCoV-79228|EPI_ISL_10820632|USA|BA.1.15|2022-01-12	99,64
EPI_ISL_10822502	BA.1.1	BA.1.1	BA.1.1_2	17-032980-KR|EPI_ISL_10822502|Slovenia|BA.1.1|2022-02-15	93,88
EPI_ISL_10823573	BA.1.1	BA.1.1	BA.1.1_2	TX-HMH-MCoV-80486|EPI_ISL_10823573|USA|BA.1.1|2022-01-10	99,64
EPI_ISL_10824182	BA.1.1	BA.1.1	BA.1.1_2	TI-EOC-26709594|EPI_ISL_10824182|Switzerland|BA.1.1|2022-02-25	99,60
EPI_ISL_10825405	BA.1.1	BA.1.1	BA.1.1_3	17-007741-CE|EPI_ISL_10825405|Slovenia|BA.1.1|2022-01-10	99,64
EPI_ISL_10829199	BA.1.15	BA.1.15	BA.1.15	TX-HMH-MCoV-86383|EPI_ISL_10829199|USA|BA.1.15|2022-01-26	99,64
EPI_ISL_10829325	BA.1.1	BA.1.1	BA.1.1_2	TX-HMH-MCoV-84282|EPI_ISL_10829325|USA|BA.1.1|2022-01-22	99,64
EPI_ISL_10830860	BA.1.15	BA.1	BA.1_4	TX-HMH-MCoV-88055|EPI_ISL_10830860|USA|BA.1.15|2022-01-28	99,64
EPI_ISL_10831033	BA.1.1	BA.1.1	BA.1.1_2	TX-HMH-MCoV-89426|EPI_ISL_10831033|USA|BA.1.1|2022-02-04	99,64
EPI_ISL_10831052	BA.1.1	BA.1.1	BA.1.1_3	TX-HMH-MCoV-89402|EPI_ISL_10831052|USA|BA.1.1|2022-02-03	99,64
EPI_ISL_10832239	BA.1.15	BA.1.15	BA.1.15	TX-HMH-MCoV-90771|EPI_ISL_10832239|USA|BA.1.15|2021-12-28	99,64
EPI_ISL_10833127	BA.1.15	BA.1	BA.1_4	TX-HMH-MCoV-83487|EPI_ISL_10833127|USA|BA.1.15|2022-01-18	99,58
EPI_ISL_10833309	BA.1.15	BA.1.15	BA.1.15	TX-HMH-MCoV-83052|EPI_ISL_10833309|USA|BA.1.15|2022-01-19	99,64
EPI_ISL_10833419	BA.1.1	BA.1.1	BA.1.1_2	TX-HMH-MCoV-83161|EPI_ISL_10833419|USA|BA.1.1|2022-01-19	99,62
EPI_ISL_10833562	BA.1.1	BA.1.1	BA.1.1_2	TX-HMH-MCoV-90974|EPI_ISL_10833562|USA|BA.1.1|2022-02-10	99,58
EPI_ISL_10834701	BA.1.15	BA.1.15	BA.1.15	TX-HMH-MCoV-82401|EPI_ISL_10834701|USA|BA.1.15|2022-01-16	99,62
EPI_ISL_10835896	BA.1.15	BA.1.15	BA.1.15	TX-HMH-MCoV-90485|EPI_ISL_10835896|USA|BA.1.15|2021-12-27	99,64
EPI_ISL_10837464	BA.1	BA.1.15	BA.1.15	TX-HMH-MCoV-93100|EPI_ISL_10837464|USA|BA.1|2021-12-30	99,62
EPI_ISL_10838166	BA.1	BA.1.15	BA.1.15	TX-HMH-MCoV-93613|EPI_ISL_10838166|USA|BA.1|2022-02-22	99,43
EPI_ISL_10838837	BA.1.1	BA.1.1	BA.1.1_2	MA-CDC-STM-WXT4UGNPQ|EPI_ISL_10838837|USA|BA.1.1|2022-02-22	99,64
EPI_ISL_10839204	BA.1.1.18	BA.1.1	BA.1.1_3	TN-CDC-LC0548229|EPI_ISL_10839204|USA|BA.1.1.18|2022-02-09	99,64
EPI_ISL_10839275	BA.1.1	BA.1.1	BA.1.1_2	TN-CDC-LC0550173|EPI_ISL_10839275|USA|BA.1.1|2022-02-11	99,64
EPI_ISL_10840527	BA.1.1.18	BA.1.1	BA.1.1_3	VA-CDC-LC0549504|EPI_ISL_10840527|USA|BA.1.1.18|2022-02-20	99,64
EPI_ISL_10842496	BA.1.1	BA.1.1	BA.1.1_3	CA-OC-2449|EPI_ISL_10842496|USA|BA.1.1|2022-02-17	99,64
EPI_ISL_10843677	BA.1.1.2	BA.1.1	BA.1.1_3	TKYkbm6196|EPI_ISL_10843677|Japan|BA.1.1.2|2022-01-18	99,64
EPI_ISL_10843783	BA.1.1.2	BA.1.1	BA.1.1_3	TKYkbm6802|EPI_ISL_10843783|Japan|BA.1.1.2|2022-01-22	99,64
EPI_ISL_10844458	BA.1.18	BA.1	BA.1_4	CT-HUGTiPM083AR3A3|EPI_ISL_10844458|Spain|BA.1.18|2022-02-23	99,64
EPI_ISL_10844651	BA.1.1	BA.1.1	BA.1.1_2	VEN-OR01P18839|EPI_ISL_10844651|Italy|BA.1.1|2022-01-21	99,64
EPI_ISL_10844838	BA.1.17.2	BA.1.17.2	BA.1.17.2	MILK-38F7A3A|EPI_ISL_10844838|United Kingdom|BA.1.17.2|2022-03-02	99,64
EPI_ISL_10845386	BA.2	BA.2	BA.2_1	QEUH-38E3A9B|EPI_ISL_10845386|United Kingdom|BA.2|2022-03-01	99,81
EPI_ISL_10846323	XE	A	A_14	BRBR-38EF473|EPI_ISL_10846323|United Kingdom|XE|2022-03-02	99,81
EPI_ISL_10847003	BA.1	BA.1.15	BA.1.15	Kce-2919|EPI_ISL_10847003|Poland|BA.1|2022-03-01	88,17
EPI_ISL_10848404	BA.1	BA.1	BA.1_4	QEUH-38D8BD2|EPI_ISL_10848404|United Kingdom|BA.1|2022-03-01	99,64
EPI_ISL_10850980	BA.1	BA.1	BA.1_4	QEUH-38D3C98|EPI_ISL_10850980|United Kingdom|BA.1|2022-03-01	99,64
EPI_ISL_10852055	BA.1.1.1	BA.1.1	BA.1.1_3	QEUH-38E402F|EPI_ISL_10852055|United Kingdom|BA.1.1.1|2022-02-28	99,64
EPI_ISL_10852058	BA.1.15.1	BA.1.15.1	BA.1.15.1	DHSC-CYNRCO7|EPI_ISL_10852058|United Kingdom|BA.1.15.1|2022-02-23	99,64
EPI_ISL_10852801	BA.2	BA.2	BA.2_1	QEUH-38E5D36|EPI_ISL_10852801|United Kingdom|BA.2|2022-03-01	99,81
EPI_ISL_10856224	BA.1.17	BA.1	BA.1_3	CL-COV25711|EPI_ISL_10856224|Spain|BA.1.17|2022-02-12	96,03
EPI_ISL_10856711	BA.1.18	BA.1	BA.1_3	CL-COV25951|EPI_ISL_10856711|Spain|BA.1.18|2022-02-18	98,30
EPI_ISL_10858867	BA.2.9	BA.2	BA.2_1	PLYM-38EBC15|EPI_ISL_10858867|United Kingdom|BA.2.9|2022-03-02	96,26
EPI_ISL_10858969	BA.2.9	A	A_17	DCGC-410853|EPI_ISL_10858969|Denmark|BA.2.9|2022-03-03	99,75
EPI_ISL_10860746	BA.2	BA.2	BA.2_1	PHEC-5W03FZ3A|EPI_ISL_10860746|United Kingdom|BA.2|2022	99,81
EPI_ISL_10861275	BA.1.1	BA.1.1	BA.1.1_3	22BG-NC_015783_R27|EPI_ISL_10861275|Bulgaria|BA.1.1|2022-01-18	99,64
EPI_ISL_10861698	BA.1.1	BA.1.1	BA.1.1_2	PHEC-YYNHTQA|EPI_ISL_10861698|United Kingdom|BA.1.1|2022-02-12	98,21
EPI_ISL_10861747	BA.2.9	A	A_17	DCGC-412027|EPI_ISL_10861747|Denmark|BA.2.9|2022-03-03	99,81
EPI_ISL_10863861	BA.2.10	A	A_14	PHWC-PEWID4|EPI_ISL_10863861|United Kingdom|BA.2.10|2022-02-16	99,81
EPI_ISL_10864062	BA.2	A	A_17	PHWC-PEWWSW|EPI_ISL_10864062|United Kingdom|BA.2|2022-02-20	99,81
EPI_ISL_10864817	BA.2	BA.2	BA.2_1	AZDelta-2208-10577|EPI_ISL_10864817|Belgium|BA.2|2022-02-28	98,76
EPI_ISL_10865519	BA.1.15	BA.1	BA.1_1	TX-HHD-2202246456|EPI_ISL_10865519|USA|BA.1.15|2022-02-18	95,13
EPI_ISL_10869291	BA.1.1	BA.1.1	BA.1.1_2	UT-UPHL-220308563096|EPI_ISL_10869291|USA|BA.1.1|2022-01-31	99,64
EPI_ISL_10872626	BA.1.18	BA.1	BA.1_3	TX-CDC-ASC210752953|EPI_ISL_10872626|USA|BA.1.18|2022-02-25	99,64
EPI_ISL_10875848	BA.1.1	BA.1.1	BA.1.1_2	MA-CDCBI-CRSP_2AEGQEW3PBCUQEUU|EPI_ISL_10875848|USA|BA.1.1|2022-02-28	99,64
EPI_ISL_10878313	BA.1.15	BA.1	BA.1_4	NE-NPHL22-4133|EPI_ISL_10878313|USA|BA.1.15|2022-01-13	99,64
EPI_ISL_10878568	BA.1	BA.1	BA.1_3	NICD-N34936|EPI_ISL_10878568|South Africa|BA.1|2022-01-03	96,66
EPI_ISL_10879094	BA.1.1	BA.1.1	BA.1.1_2	NE-COPH-S806780|EPI_ISL_10879094|USA|BA.1.1|2022-01-31	96,72
EPI_ISL_10879345	BA.1.1.16	BA.1.1	BA.1.1_2	MD-HP27586-PIDVNPZRKZ|EPI_ISL_10879345|USA|BA.1.1.16|2021-12-31	99,64
EPI_ISL_10879620	BA.1.1.18	BA.1.1	BA.1.1_3	TKYkbm7152|EPI_ISL_10879620|Japan|BA.1.1.18|2022-01-25	99,64
EPI_ISL_10879624	BA.1.1.2	BA.1.1	BA.1.1_3	TKYkbm7156|EPI_ISL_10879624|Japan|BA.1.1.2|2022-01-25	99,64
EPI_ISL_10879640	BA.1.1.2	BA.1.1	BA.1.1_3	TKYkbm7172|EPI_ISL_10879640|Japan|BA.1.1.2|2022-01-25	99,64
EPI_ISL_10880889	BA.2	A	A_14	QEUH-3903751|EPI_ISL_10880889|United Kingdom|BA.2|2022-03-02	99,81
EPI_ISL_10882893	BA.1.1	BA.1.1	BA.1.1_2	BRBR-38F8565|EPI_ISL_10882893|United Kingdom|BA.1.1|2022-03-03	99,64
EPI_ISL_10883010	BA.2	A	A_17	MILK-3905A56|EPI_ISL_10883010|United Kingdom|BA.2|2022-03-04	99,81
EPI_ISL_10884252	BA.2	A	A_14	PHEC-YYN9QTQ|EPI_ISL_10884252|United Kingdom|BA.2|2022-02-22	92,44
EPI_ISL_10886198	BA.1.1	BA.1.1	BA.1.1_3	PHWC-PEI4MD|EPI_ISL_10886198|United Kingdom|BA.1.1|2022-02-19	99,64
EPI_ISL_10887780	BA.1	BA.1.1	BA.1.1_2	PHWC-PESB9T|EPI_ISL_10887780|United Kingdom|BA.1|2022-02-16	99,64
EPI_ISL_10888862	BA.1.1	BA.1.1	BA.1.1_3	PHWC-PESQQ8|EPI_ISL_10888862|United Kingdom|BA.1.1|2022-02-17	99,64
EPI_ISL_10889369	BA.1.17	BA.1	BA.1_3	ICH-741121187|EPI_ISL_10889369|Israel|BA.1.17|2022-02-12	96,28
EPI_ISL_10890780	BA.1.15	BA.1	BA.1_1	TX-HHD-2202098259|EPI_ISL_10890780|USA|BA.1.15|2022-02-03	95,13
EPI_ISL_10890960	BA.1	BA.1.15	BA.1.15	NIC_BKK_FRS559|EPI_ISL_10890960|Thailand|BA.1|2022-01-28	99,64
EPI_ISL_10891660	BA.2	A	A_17	DCGC-413141|EPI_ISL_10891660|Denmark|BA.2|2022-03-04	99,81
EPI_ISL_10891665	BA.2	A	A_17	DCGC-413146|EPI_ISL_10891665|Denmark|BA.2|2022-03-02	99,79
EPI_ISL_10891760	BA.2	A	A_17	DCGC-413241|EPI_ISL_10891760|Denmark|BA.2|2022-03-02	99,79
EPI_ISL_10896204	BA.1.1	BA.1.1	BA.1.1_3	TN-CDC-LC0553730|EPI_ISL_10896204|USA|BA.1.1|2022-02-13	99,64
EPI_ISL_10896437	BA.2	BA.2.2	BA.2.2	TX-CDC-LC0554737|EPI_ISL_10896437|USA|BA.2|2022-02-18	99,81
EPI_ISL_10896565	BA.1.1	BA.1.1	BA.1.1_2	FL-BPHL-3041|EPI_ISL_10896565|USA|BA.1.1|2022-01-14	82,48
EPI_ISL_10897179	BA.1	BA.1	BA.1_2	LB-R00064-S370|EPI_ISL_10897179|Austria|BA.1|2022-03-03	89,83
EPI_ISL_10897416	BA.1.1.18	BA.1.1	BA.1.1_3	PA-CDC-LC0555388|EPI_ISL_10897416|USA|BA.1.1.18|2022-02-23	99,64
EPI_ISL_10897709	BA.1.1	BA.1.1	BA.1.1_3	WV-CDC-LC0556071|EPI_ISL_10897709|USA|BA.1.1|2022-02-24	99,64
EPI_ISL_10898412	BA.1.13	BA.1	BA.1_2	CVL-HMN-22022240257|EPI_ISL_10898412|France|BA.1.13|2022-01-31	99,07
EPI_ISL_10899430	BA.1.1	BA.1.1	BA.1.1_2	LI-RIVM-90554|EPI_ISL_10899430|Netherlands|BA.1.1|2022-02-22	99,64
EPI_ISL_10900103	BA.1.17.2	BA.1.17.2	BA.1.17.2	SK-NML-367940|EPI_ISL_10900103|Canada|BA.1.17.2|2022-02-21	99,60
EPI_ISL_10900181	BA.1	BA.1.1	BA.1.1_3	SK-RRPL-368575|EPI_ISL_10900181|Canada|BA.1|2022-02-14	99,64
EPI_ISL_10901180	BA.1.15	BA.1	BA.1_4	WI-CDC-QDX34231195|EPI_ISL_10901180|USA|BA.1.15|2022-02-22	99,64
EPI_ISL_10901957	BA.1.1	BA.1.1	BA.1.1_2	NV-CDC-QDX34246669|EPI_ISL_10901957|USA|BA.1.1|2022-02-26	99,64
EPI_ISL_10902599	BA.2	A	A_17	NW-RKI-I-584467|EPI_ISL_10902599|Germany|BA.2|2022-02-17	96,97
EPI_ISL_10904367	BA.1.1	BA.1.1	BA.1.1_2	BW-RKI-I-586783|EPI_ISL_10904367|Germany|BA.1.1|2022-02-21	99,64
EPI_ISL_10904398	BA.2	BA.2	BA.2_1	BW-RKI-I-586909|EPI_ISL_10904398|Germany|BA.2|2022-02-18	99,81
EPI_ISL_10905901	BA.1.1	BA.1.1	BA.1.1_2	AZ-ASU51144|EPI_ISL_10905901|USA|BA.1.1|2022-01-20	90,98

EPI_ISL_10906741	BA.1.1	BA.1	BA.1_1	NI-RKI-I-589923|EPI_ISL_10906741|Germany|BA.1.1|2022-02-13	90,99
EPI_ISL_10906833	BA.2	BA.2	BA.2_1	RP-RKI-I-590018|EPI_ISL_10906833|Germany|BA.2|2022-02-15	97,56
EPI_ISL_10906967	BA.1.1	BA.1	BA.1_2	NI-RKI-I-590155|EPI_ISL_10906967|Germany|BA.1.1|2022-02-19	99,33
EPI_ISL_10910123	BA.1.15	BA.1	BA.1_4	AL-UAB-GX2187|EPI_ISL_10910123|USA|BA.1.15|2022-02-07	97,20
EPI_ISL_10911136	BA.1.1	BA.1.1	BA.1.1_2	BY-RKI-I-593639|EPI_ISL_10911136|Germany|BA.1.1|2022-02-23	99,64
EPI_ISL_10912175	BA.1.1.2	BA.1.1	BA.1.1_3	TKYkbm9097|EPI_ISL_10912175|Japan|BA.1.1.2|2022-02-08	99,64
EPI_ISL_10912420	BA.2	BA.2	BA.2_1	BW-RKI-I-594778|EPI_ISL_10912420|Germany|BA.2|2022-02-24	99,81
EPI_ISL_10914252	BA.1.1	BA.1.1	BA.1.1_3	NW-RKI-I-598372|EPI_ISL_10914252|Germany|BA.1.1|2022-02-21	99,56
EPI_ISL_10914294	BA.2	BA.2	BA.2_1	NW-RKI-I-598414|EPI_ISL_10914294|Germany|BA.2|2022-02-23	99,81
EPI_ISL_10916664	BA.1.1	BA.1.1	BA.1.1_2	NI-RKI-I-601474|EPI_ISL_10916664|Germany|BA.1.1|2022-02-04	99,64
EPI_ISL_10916923	BA.2.9	BA.2	BA.2_1	BY-RKI-I-601739|EPI_ISL_10916923|Germany|BA.2.9|2022-03-01	99,81
EPI_ISL_10917878	BA.2	A	A_17	SN-RKI-I-602360|EPI_ISL_10917878|Germany|BA.2|2022-03-02	99,07
EPI_ISL_10919962	BA.2	BA.2	BA.2_1	NW-RKI-I-605537|EPI_ISL_10919962|Germany|BA.2|2022-02-23	95,40
EPI_ISL_10920179	BA.2	A	A_17	NW-RKI-I-605776|EPI_ISL_10920179|Germany|BA.2|2022-03-04	99,81
EPI_ISL_10921332	BA.1.18	BA.1	BA.1_3	HH-RKI-I-606967|EPI_ISL_10921332|Germany|BA.1.18|2022-02-15	99,58
EPI_ISL_10922498	BA.1	BA.1.15	BA.1.15	BY-RKI-I-608448|EPI_ISL_10922498|Germany|BA.1|2022-01-25	90,82
EPI_ISL_10922554	BA.1	BA.1.15	BA.1.15	BY-RKI-I-608509|EPI_ISL_10922554|Germany|BA.1|2022-01-25	92,62
EPI_ISL_10923852	BA.2	A	A_4	PLYM-39365E2|EPI_ISL_10923852|United Kingdom|BA.2|2022-03-03	99,81
EPI_ISL_10924919	BA.2	BA.2	BA.2_1	QEUH-393FA80|EPI_ISL_10924919|United Kingdom|BA.2|2022-03-04	99,81
EPI_ISL_10925013	BA.2	A	A_14	MILK-393D7A9|EPI_ISL_10925013|United Kingdom|BA.2|2022-03-06	99,81
EPI_ISL_10925558	BA.2.3	BA.2	BA.2_1	LSPA-393BB90|EPI_ISL_10925558|United Kingdom|BA.2.3|2022-03-04	99,81
EPI_ISL_10927803	BA.2.9	A	A_17	ARA-CFD700000294946|EPI_ISL_10927803|France|BA.2.9|2022-03-06	99,81
EPI_ISL_10928861	BA.2	A	A_14	LSPA-3913FB0|EPI_ISL_10928861|United Kingdom|BA.2|2022-03-03	99,81
EPI_ISL_10929751	BA.2	BA.2	BA.2_1	4075673064|EPI_ISL_10929751|Sweden|BA.2|2022-02-25	99,81
EPI_ISL_10934246	BA.1	BA.1.15	BA.1.15	QEUH-3903EB6|EPI_ISL_10934246|United Kingdom|BA.1|2022-03-03	99,64
EPI_ISL_10934734	BA.2	BA.2	BA.2_1	MILK-392C549|EPI_ISL_10934734|United Kingdom|BA.2|2022-03-04	99,81
EPI_ISL_10935068	BA.2	BA.2	BA.2_1	DCGC-415820|EPI_ISL_10935068|Denmark|BA.2|2022-02-20	99,81
EPI_ISL_10938617	BA.2.9	A	A_17	DCGC-416346|EPI_ISL_10938617|Denmark|BA.2.9|2022-03-03	99,77
EPI_ISL_10940056	BA.2.9	A	A_17	DCGC-417022|EPI_ISL_10940056|Denmark|BA.2.9|2022-03-05	99,81
EPI_ISL_10943321	BA.2	BA.2	BA.2_1	D-NVRL-G22IRL07167|EPI_ISL_10943321|Ireland|BA.2|2022-02-02	90,35
EPI_ISL_10943545	BA.1.17.2	A	A_1	PAC-ChTo-T220470460|EPI_ISL_10943545|France|BA.1.17.2|2022-01-30	69,55
EPI_ISL_10944032	BA.2	BA.2.10	BA.2.10	RJ-SMS-ICMR-INSACOG-TS-8067|EPI_ISL_10944032|India|BA.2|2022-02-03	97,48
EPI_ISL_10945191	BA.1.15	BA.1.15	BA.1.15	TX-HMH-MCoV-94883|EPI_ISL_10945191|USA|BA.1.15|2021-12-28	99,64
EPI_ISL_10947472	BA.1.1.18	BA.1.1	BA.1.1_2	GA-CDC-MMB14273044|EPI_ISL_10947472|USA|BA.1.1.18|2022-02-09	92,23
EPI_ISL_10947963	BA.1.1	BA.1.1	BA.1.1_2	MA-CDCBI-CRSP_TT35FXEH73XIUG4X|EPI_ISL_10947963|USA|BA.1.1|2022-03-03	99,64
EPI_ISL_10950868	BA.1.1	BA.1.1	BA.1.1_2	ME-CDC-ASC210730168|EPI_ISL_10950868|USA|BA.1.1|2022-03-01	90,94
EPI_ISL_10955485	BA.1.1	BA.1.1	BA.1.1_2	CO-CDPHE-2102529881|EPI_ISL_10955485|USA|BA.1.1|2021-12-28	90,98
EPI_ISL_10955532	BA.1.1.18	BA.1.1	BA.1.1_2	CO-CDPHE-2102537366|EPI_ISL_10955532|USA|BA.1.1.18|2021-12-28	90,98
EPI_ISL_10956199	BA.1.1.18	BA.1.1	BA.1.1_3	CO-CDPHE-2102987079|EPI_ISL_10956199|USA|BA.1.1.18|2022-02-24	89,32
EPI_ISL_10956338	BA.1.1	BA.1	BA.1_4	CO-CDPHE-2102968150|EPI_ISL_10956338|USA|BA.1.1|2022-01-01	83,72
EPI_ISL_10956931	BA.1.1	BA.1	BA.1_4	CO-CDPHE-2103012117|EPI_ISL_10956931|USA|BA.1.1|2022-01-20	93,11
EPI_ISL_10957252	BA.1.1	BA.1	BA.1_4	CO-CDPHE-2103006102|EPI_ISL_10957252|USA|BA.1.1|2022-01-21	79,41
EPI_ISL_10958065	BA.1.1	BA.1.1	BA.1.1_2	PA-CDC-LC0556950|EPI_ISL_10958065|USA|BA.1.1|2022-02-17	99,64
EPI_ISL_10958444	BA.1.1	BA.1.15	BA.1.15	NV-CDC-LC0557254|EPI_ISL_10958444|USA|BA.1.1|2022-02-28	86,32
EPI_ISL_10960353	BA.2	BA.2	BA.2_1	MILK-3962F94|EPI_ISL_10960353|United Kingdom|BA.2|2022-03-06	99,81
EPI_ISL_10960391	BA.2	A	A_14	MILK-3961A58|EPI_ISL_10960391|United Kingdom|BA.2|2022-03-05	99,81
EPI_ISL_10961186	BA.2	A	A_14	LSPA-390F166|EPI_ISL_10961186|United Kingdom|BA.2|2022-03-03	99,81
EPI_ISL_10961691	BA.2	BA.2	BA.2_1	LSPA-3930A43|EPI_ISL_10961691|United Kingdom|BA.2|2022-03-04	99,81
EPI_ISL_10963110	BA.2	A	A_14	LSPA-394BFA0|EPI_ISL_10963110|United Kingdom|BA.2|2022-03-04	99,81
EPI_ISL_10964334	BA.1.1.13	BA.1.1	BA.1.1_3	MILK-3946752|EPI_ISL_10964334|United Kingdom|BA.1.1.13|2022-03-05	99,64
EPI_ISL_10964455	BA.1.1.1	BA.1.1	BA.1.1_1	ALDP-3933D83|EPI_ISL_10964455|United Kingdom|BA.1.1.1|2022-03-04	99,64
EPI_ISL_10966687	BA.2	A	A_17	QEUH-3956F48|EPI_ISL_10966687|United Kingdom|BA.2|2022-03-05	99,81
EPI_ISL_10967925	BA.2	BA.2.10	BA.2.10	ARA-IPP12399|EPI_ISL_10967925|France|BA.2|2022-02-14	99,81
EPI_ISL_10969239	BA.1	BA.1.15	BA.1.15	BA_22_00011132|EPI_ISL_10969239|Slovakia|BA.1|2022-02-18	92,10
EPI_ISL_10971412	BA.2.10	A	A_14	BRBR-397A3F7|EPI_ISL_10971412|United Kingdom|BA.2.10|2022-03-06	99,81
EPI_ISL_10971458	BA.1.1.15	BA.1.1	BA.1.1_2	BRBR-395DA40|EPI_ISL_10971458|United Kingdom|BA.1.1.15|2022-03-05	94,85
EPI_ISL_10972352	BA.2	BA.2	BA.2_1	BRBR-39552E6|EPI_ISL_10972352|United Kingdom|BA.2|2022-03-05	99,81
EPI_ISL_10974207	BA.1.17	BA.1.15	BA.1.15	QLD0x00CF22|EPI_ISL_10974207|Australia|BA.1.17|2022-02-01	99,64
EPI_ISL_10974751	BA.1.17	BA.1	BA.1_2	QLD0x00C988|EPI_ISL_10974751|Australia|BA.1.17|2022-02-05	99,64
EPI_ISL_10975272	BA.1.17	BA.1	BA.1_1	QLD0x00CB4C|EPI_ISL_10975272|Australia|BA.1.17|2022-02-11	99,50
EPI_ISL_10975370	BA.1.17	BA.1	BA.1_4	QLD0x00C8E5|EPI_ISL_10975370|Australia|BA.1.17|2022-01-27	99,64
EPI_ISL_10975386	BA.1.17	BA.1	BA.1_4	QLD0x00CA32|EPI_ISL_10975386|Australia|BA.1.17|2022-02-04	99,60
EPI_ISL_10975968	BA.2	BA.2	BA.2_1	LSPA-3943CA1|EPI_ISL_10975968|United Kingdom|BA.2|2022-03-04	99,81
EPI_ISL_10976245	BA.1.1	BA.1.1	BA.1.1_3	LSPA-39441B0|EPI_ISL_10976245|United Kingdom|BA.1.1|2022-03-04	99,64
EPI_ISL_10976587	BA.2.10	BA.2.10	BA.2.10	LSPA-39633C4|EPI_ISL_10976587|United Kingdom|BA.2.10|2022-03-06	99,81
EPI_ISL_10976764	BA.2	BA.2	BA.2_1	LSPA-39630DC|EPI_ISL_10976764|United Kingdom|BA.2|2022-03-06	99,81
EPI_ISL_10976774	BA.2	A	A_14	MILK-39699CC|EPI_ISL_10976774|United Kingdom|BA.2|2022-03-07	99,81
EPI_ISL_10977043	BA.1.17.2	BA.1.17.2	BA.1.17.2	BRBR-395C908|EPI_ISL_10977043|United Kingdom|BA.1.17.2|2022-03-07	99,64
EPI_ISL_10978119	BA.2.32	BA.2.32	BA.2.32	YO-GS-10056|EPI_ISL_10978119|Indonesia|BA.2.32|2022-02-07	99,81
EPI_ISL_10978455	BA.2	BA.2	BA.2_1	BRBR-397185B|EPI_ISL_10978455|United Kingdom|BA.2|2022-03-06	99,81
EPI_ISL_10978470	BA.1.1.15	BA.1.1	BA.1.1_2	QEUH-3973543|EPI_ISL_10978470|United Kingdom|BA.1.1.15|2022-03-05	99,64
EPI_ISL_10979320	BA.2	A	A_17	QEUH-3957D5C|EPI_ISL_10979320|United Kingdom|BA.2|2022-03-04	99,81
EPI_ISL_10981712	BA.2.12	A	A_4	SA141942|EPI_ISL_10981712|Australia|BA.2.12|2022-03-06	97,96
EPI_ISL_10982199	BA.2	BA.2	BA.2_1	DCGC-418098|EPI_ISL_10982199|Denmark|BA.2|2022-02-14	99,81
EPI_ISL_10986311	BA.2.10	A	A_14	PT29559|EPI_ISL_10986311|Portugal|BA.2.10|2022-02-27	99,81
EPI_ISL_10986587	BA.2	A	A_14	BRBR-39906A7|EPI_ISL_10986587|United Kingdom|BA.2|2022-03-07	99,79
EPI_ISL_10988090	BA.2.10	A	A_14	LSPA-39AF13B|EPI_ISL_10988090|United Kingdom|BA.2.10|2022-03-08	99,81
EPI_ISL_10988873	BA.2	A	A_14	BRBR-39A80BD|EPI_ISL_10988873|United Kingdom|BA.2|2022-03-07	99,81
EPI_ISL_10989322	BA.2	A	A_14	QEUH-39AA983|EPI_ISL_10989322|United Kingdom|BA.2|2022-03-06	99,81
EPI_ISL_10990410	BA.2	A	A_14	LSPA-39ADA7E|EPI_ISL_10990410|United Kingdom|BA.2|2022-03-08	99,81
EPI_ISL_10990682	BA.2	BA.2	BA.2_1	LSPA-39AD021|EPI_ISL_10990682|United Kingdom|BA.2|2022-03-08	99,81
EPI_ISL_10991688	BA.2	A	A_14	LSPA-399E8A3|EPI_ISL_10991688|United Kingdom|BA.2|2022-03-07	99,81
EPI_ISL_10993070	BA.2	A	A_17	LSPA-39A1E9C|EPI_ISL_10993070|United Kingdom|BA.2|2022-03-04	99,81
EPI_ISL_10993452	BA.1.1	BA.1.1	BA.1.1_2	ARCH-002819D2|EPI_ISL_10993452|United Kingdom|BA.1.1|2022	94,58
EPI_ISL_10994843	BA.2.10	BA.2	BA.2_1	MAV172341|EPI_ISL_10994843|Maldives|BA.2.10|2022-02-20	91,95
EPI_ISL_10996148	BA.2.10	A	A_4	8314|EPI_ISL_10996148|Mauritius|BA.2.10|2022-02-07	90,52
EPI_ISL_10996246	BA.2	BA.2	BA.2_1	DHSC-CYYIABX|EPI_ISL_10996246|United Kingdom|BA.2|2022-02-22	99,81
EPI_ISL_10996341	BA.1.1	BA.1.1	BA.1.1_3	DHSC-CYYQ9ZK|EPI_ISL_10996341|United Kingdom|BA.1.1|2022-01-28	99,64
EPI_ISL_10998196	BA.1.1	BA.1.1	BA.1.1_2	NORT-YNNF4XA|EPI_ISL_10998196|United Kingdom|BA.1.1|2022	99,64
EPI_ISL_10999240	BA.2	A	A_14	NORT-YNNG89E|EPI_ISL_10999240|United Kingdom|BA.2|2022	90,48
EPI_ISL_11000561	BA.2.3	A	A_14	NORT-YNNRZ1S|EPI_ISL_11000561|United Kingdom|BA.2.3|2022	99,81
EPI_ISL_11001526	BA.2.10	BA.2	BA.2_1	AP-CCMB-CIC4829|EPI_ISL_11001526|India|BA.2.10|2022-01-20	99,81
EPI_ISL_11001664	BA.2.10	BA.2	BA.2_1	AP-CCMB-CIC4996|EPI_ISL_11001664|India|BA.2.10|2022-01-27	97,67
EPI_ISL_11002140	BA.1.1.15	BA.1.1	BA.1.1_2	PHEC-YYDBEAN|EPI_ISL_11002140|United Kingdom|BA.1.1.15|2022-01-27	96,70
EPI_ISL_11002202	BA.2.9	A	A_17	FL-Risch-2230401409|EPI_ISL_11002202|Liechtenstein|BA.2.9|2022-02-03	90,50
EPI_ISL_11002874	BA.1.15	BA.1	BA.1_4	SP-IB_153455|EPI_ISL_11002874|Brazil|BA.1.15|2022-02-15	99,64
EPI_ISL_11004114	BA.2	BA.2	BA.2_1	PHEC-YYDNJYO|EPI_ISL_11004114|United Kingdom|BA.2|2022-03-01	99,79
EPI_ISL_11005353	BA.1.17.2	BA.1.17.2	BA.1.17.2	PHWC-PE45BC|EPI_ISL_11005353|United Kingdom|BA.1.17.2|2022-02-25	99,62
EPI_ISL_11005884	BA.2	BA.2	BA.2_1	PHWC-PE4OAE|EPI_ISL_11005884|United Kingdom|BA.2|2022-02-24	99,81
EPI_ISL_11006588	BA.1.1	BA.1.1	BA.1.1_2	PHWC-PE5QMD|EPI_ISL_11006588|United Kingdom|BA.1.1|2022-02-24	93,68
EPI_ISL_11007291	BA.2	A	A_14	PHWC-PEAOCF|EPI_ISL_11007291|United Kingdom|BA.2|2022-02-21	99,81

EPI_ISL_11007652	BA.1.15	BA.1	BA.1_4	TX-HMH-MCoV-95350|EPI_ISL_11007652|USA|BA.1.15|2021-12-28	99,60
EPI_ISL_11010762	BA.1.1	BA.1.1	BA.1.1_2	NM-UNM-ED_02563|EPI_ISL_11010762|USA|BA.1.1|2022-02-23	96,53
EPI_ISL_11011370	BA.1.1	BA.1.1	BA.1.1_2	NY-NYGC-1101-VTM1-YRPW669|EPI_ISL_11011370|USA|BA.1.1|2022-01-24	99,64
EPI_ISL_11012350	BA.1.15	A	A_4	QUI-UTP-VG-742|EPI_ISL_11012350|Colombia|BA.1.15|2021-12	74,18
EPI_ISL_11013977	BA.1.1	BA.1.1	BA.1.1_2	CA-IVY-A93J26M7|EPI_ISL_11013977|USA|BA.1.1|2022-02-11	99,64
EPI_ISL_11015148	BA.1.1	BA.1.1	BA.1.1_2	UT-UPHL-220312052596|EPI_ISL_11015148|USA|BA.1.1|2022-02-25	99,81
EPI_ISL_11016628	BA.1.1	BA.1.1	BA.1.1_2	TX-DSHS-16306|EPI_ISL_11016628|USA|BA.1.1|2022-01-28	62,47
EPI_ISL_11017537	BA.1	BA.1	BA.1_4	CERI-KRISP-K037001|EPI_ISL_11017537|Angola|BA.1|2022-01-04	86,93
EPI_ISL_11017605	BA.2.10	A	A_14	22CV2641|EPI_ISL_11017605|New Zealand|BA.2.10|2022-02-24	90,52
EPI_ISL_11018621	BA.1	BA.1	BA.1_4	CA-CDPH-500044507|EPI_ISL_11018621|USA|BA.1|2022-01-03	95,90
EPI_ISL_11018678	BA.1.1	BA.1.1	BA.1.1_2	CA-CDPH-500044578|EPI_ISL_11018678|USA|BA.1.1|2021-12-31	90,84
EPI_ISL_11019127	BA.1.1	BA.1.1	BA.1.1_2	CA-CDPH-500047955|EPI_ISL_11019127|USA|BA.1.1|2022-01-18	95,92
EPI_ISL_11019159	BA.1.15	BA.1.15	BA.1.15	CA-CDPH-500048017|EPI_ISL_11019159|USA|BA.1.15|2022-01-21	90,86
EPI_ISL_11019396	BA.2.3	A	A_4	CA-CDPH-500049532|EPI_ISL_11019396|USA|BA.2.3|2022-01-27	99,47
EPI_ISL_11019646	BA.1.18	BA.1.15	BA.1.15	NAQ-IPP12766|EPI_ISL_11019646|France|BA.1.18|2022-02-14	99,64
EPI_ISL_11020704	BA.1.1.1	BA.1.1	BA.1.1_3	GES-IPP11502|EPI_ISL_11020704|France|BA.1.1.1|2022-02-07	99,64
EPI_ISL_11020742	BA.1.1.1	BA.1.1	BA.1.1_3	CVL-IPP11570|EPI_ISL_11020742|France|BA.1.1.1|2022-02-13	99,64
EPI_ISL_11022223	BA.2	A	A_4	PHEC-YYDRN8F|EPI_ISL_11022223|United Kingdom|BA.2|2022-02-27	99,73
EPI_ISL_11023523	BA.1.1.15	BA.1.1	BA.1.1_2	D-Enfer-COV230222020_F5|EPI_ISL_11023523|Ireland|BA.1.1.15|2022-02-23	96,72
EPI_ISL_11024770	BA.1.1	BA.1.1	BA.1.1_3	AGES-637922|EPI_ISL_11024770|Austria|BA.1.1|2022-02-01	99,64
EPI_ISL_11026089	BA.1.1	BA.1.1	BA.1.1_2	BA-FIOCRUZ-PVM91810|EPI_ISL_11026089|Brazil|BA.1.1|2022-01-20	96,57
EPI_ISL_11026835	BA.2	A	A_17	DCGC-420923|EPI_ISL_11026835|Denmark|BA.2|2022-01-19	99,81
EPI_ISL_11027347	BA.1	BA.1.15	BA.1.15	SP-IB_153877|EPI_ISL_11027347|Brazil|BA.1|2022-02-23	99,64
EPI_ISL_11029018	BA.2	A	A_17	DCGC-422290|EPI_ISL_11029018|Denmark|BA.2|2022-03-08	99,81
EPI_ISL_11031024	BA.1.1.1	BA.1.1	BA.1.1_2	CT-HUGTiPM084OA8C2|EPI_ISL_11031024|Spain|BA.1.1.1|2022-02-28	99,64
EPI_ISL_11031356	BA.2	A	A_14	MILK-39C6F52|EPI_ISL_11031356|United Kingdom|BA.2|2022-03-08	99,81
EPI_ISL_11032861	BA.1.1	BA.1.1	BA.1.1_2	NY-CDC-LC0558500|EPI_ISL_11032861|USA|BA.1.1|2022-02-21	99,64
EPI_ISL_11032976	BA.1.1	BA.1.1	BA.1.1_2	NH-CDC-LC0558234|EPI_ISL_11032976|USA|BA.1.1|2022-02-22	99,64
EPI_ISL_11034002	BA.2	A	A_14	BRBR-39DB78F|EPI_ISL_11034002|United Kingdom|BA.2|2022-03-09	99,81
EPI_ISL_11034463	BA.1.1	BA.1	BA.1_4	NY-CDC-LC0558579|EPI_ISL_11034463|USA|BA.1.1|2022-02-27	89,62
EPI_ISL_11034614	BA.2	A	A_14	ALDP-39C5603|EPI_ISL_11034614|United Kingdom|BA.2|2022-03-07	99,81
EPI_ISL_11037901	BA.1.1	BA.1.1	BA.1.1_2	KS-KHEL-10153|EPI_ISL_11037901|USA|BA.1.1|2022-01-25	99,64
EPI_ISL_11040142	BA.2	A	A_14	HSLL-39C93AE|EPI_ISL_11040142|United Kingdom|BA.2|2022-03-08	99,81
EPI_ISL_11040337	BA.2	A	A_14	HSLL-39C9229|EPI_ISL_11040337|United Kingdom|BA.2|2022-03-08	99,81
EPI_ISL_11041233	BA.1.1	BA.1.1	BA.1.1_2	OCC-CHU-TLS-2204612822|EPI_ISL_11041233|France|BA.1.1|2022-02-15	82,48
EPI_ISL_11041821	BA.2.3	A	A_14	QEUH-39A2DE9|EPI_ISL_11041821|United Kingdom|BA.2.3|2022-03-07	99,81
EPI_ISL_11042123	BA.2.12	BA.2.12	BA.2.12	QEUH-3999750|EPI_ISL_11042123|United Kingdom|BA.2.12|2022-03-07	99,81
EPI_ISL_11045512	BA.1.1	BA.1.1	BA.1.1_2	QEUH-39821B6|EPI_ISL_11045512|United Kingdom|BA.1.1|2022-03-06	99,64
EPI_ISL_11046284	BA.2	BA.2	BA.2_1	QEUH-397ACDE|EPI_ISL_11046284|United Kingdom|BA.2|2022-03-06	99,81
EPI_ISL_11047043	BA.2.23	A	A_14	QEUH-395A278|EPI_ISL_11047043|United Kingdom|BA.2.23|2022-03-05	99,81
EPI_ISL_11055853	BA.1.1	BA.1.1	BA.1.1_2	LHUB-ULB_TAG012|EPI_ISL_11055853|Belgium|BA.1.1|2022-01-26	99,64
EPI_ISL_11061542	BA.2	A	A_17	PHWC-PEH6JI|EPI_ISL_11061542|United Kingdom|BA.2|2022-02-27	99,79
EPI_ISL_11065284	BA.2	A	A_17	DCGC-424176|EPI_ISL_11065284|Denmark|BA.2|2022-01-27	99,60
EPI_ISL_11065666	BA.2.9	A	A_17	DCGC-424451|EPI_ISL_11065666|Denmark|BA.2.9|2022-03-07	99,81
EPI_ISL_11065701	XG	BA.2	BA.2_1	DCGC-424487|EPI_ISL_11065701|Denmark|XG|2022-03-07	99,77
EPI_ISL_11066282	BA.2	A	A_14	TN-NCDC-8309360|EPI_ISL_11066282|India|BA.2|2022-02-14	99,81
EPI_ISL_11072312	BA.2.63	A	A_14	334106|EPI_ISL_11072312|Greece|BA.2.63|2022-02-20	99,81
EPI_ISL_11072364	BA.2	A	A_17	334507|EPI_ISL_11072364|Greece|BA.2|2022-02-22	99,81
EPI_ISL_11072407	BA.2	A	A_14	334096|EPI_ISL_11072407|Greece|BA.2|2022-02-20	99,81
EPI_ISL_11072993	BA.2	BA.2.12	BA.2.12	2956WSN2022_wsseol|EPI_ISL_11072993|Poland|BA.2|2022-03-02	94,75
EPI_ISL_11074945	BA.2	BA.2	BA.2_1	UP-NCDC-8109334|EPI_ISL_11074945|India|BA.2|2022-02-07	91,99
EPI_ISL_11075724	BA.2.5	BA.2.10	BA.2.10	NAT-22-20299|EPI_ISL_11075724|Czech Republic|BA.2.5|2022-02-28	98,42
EPI_ISL_11076227	BA.2	A	A_14	MILK-3A07DA5|EPI_ISL_11076227|United Kingdom|BA.2|2022-03-09	99,81
EPI_ISL_11076541	BA.2	BA.2	BA.2_1	MILK-3A03EF1|EPI_ISL_11076541|United Kingdom|BA.2|2022-03-10	94,09
EPI_ISL_11076766	BA.2	BA.2	BA.2_1	BRBR-39F991C|EPI_ISL_11076766|United Kingdom|BA.2|2022-03-10	99,81
EPI_ISL_11077934	BA.2	BA.2	BA.2_1	BRBR-39FEE27|EPI_ISL_11077934|United Kingdom|BA.2|2022-03-10	94,09
EPI_ISL_11078205	BA.2	A	A_14	PLYM-39FE179|EPI_ISL_11078205|United Kingdom|BA.2|2022-03-09	99,81
EPI_ISL_11078396	BA.1.1	BA.1.1	BA.1.1_3	MILK-3A03556|EPI_ISL_11078396|United Kingdom|BA.1.1|2022-03-10	99,64
EPI_ISL_11078398	BA.2	A	A_14	MILK-3A02399|EPI_ISL_11078398|United Kingdom|BA.2|2022-03-09	99,81
EPI_ISL_11078815	BA.2.3	A	A_14	PLYM-3A01233|EPI_ISL_11078815|United Kingdom|BA.2.3|2022-03-08	99,81
EPI_ISL_11078923	BA.2	A	A_14	ALDP-3A01FA5|EPI_ISL_11078923|United Kingdom|BA.2|2022-03-09	99,81
EPI_ISL_11080892	BA.2	BA.2	BA.2_1	BRBR-39F693D|EPI_ISL_11080892|United Kingdom|BA.2|2022-03-09	94,09
EPI_ISL_11081786	BA.1.1.14	BA.1.1	BA.1.1_3	PLYM-39D00CB|EPI_ISL_11081786|United Kingdom|BA.1.1.14|2022-03-08	99,62
EPI_ISL_11082298	BA.1.1	BA.1.1	BA.1.1_2	NC-CDC-QDX34246435|EPI_ISL_11082298|USA|BA.1.1|2022-02-24	99,64
EPI_ISL_11082699	BA.2	A	A_4	QEUH-39DF071|EPI_ISL_11082699|United Kingdom|BA.2|2022-03-09	99,81
EPI_ISL_11084422	BA.2	A	A_14	QEUH-39E067B|EPI_ISL_11084422|United Kingdom|BA.2|2022-03-09	99,81
EPI_ISL_11084715	BA.2.1	BA.2	BA.2_1	BRBR-39ECFCF|EPI_ISL_11084715|United Kingdom|BA.2.1|2022-03-09	99,81
EPI_ISL_11084747	BA.2	BA.2	BA.2_1	LSPA-39EC202|EPI_ISL_11084747|United Kingdom|BA.2|2022-03-09	93,49
EPI_ISL_11085276	BA.2	BA.2	BA.2_1	LSPA-39EA84E|EPI_ISL_11085276|United Kingdom|BA.2|2022-03-09	94,43
EPI_ISL_11087939	BA.2	A	A_14	LSPA-39D81DF|EPI_ISL_11087939|United Kingdom|BA.2|2022-03-09	99,81
EPI_ISL_11090339	BA.1.1	BA.1.1	BA.1.1_2	IL-CDC-ASC210732227|EPI_ISL_11090339|USA|BA.1.1|2022-03-05	99,64
EPI_ISL_11090964	BA.2.23	A	A_14	QEUH-39E4AE4|EPI_ISL_11090964|United Kingdom|BA.2.23|2022-03-09	99,81
EPI_ISL_11091279	BA.1.1	BA.1.1	BA.1.1_2	IL-CDC-ASC210757084|EPI_ISL_11091279|USA|BA.1.1|2022-03-07	99,64
EPI_ISL_11091811	BA.2	BA.1	BA.1_4	DA10369739|EPI_ISL_11091811|Sweden|BA.2|2022-03-02	95,94
EPI_ISL_11093369	BA.2	BA.1	BA.1_4	21805864P|EPI_ISL_11093369|Sweden|BA.2|2022-03-01	95,94
EPI_ISL_11093392	BA.1.1	BA.1.1	BA.1.1_2	AQ01135|EPI_ISL_11093392|Iran|BA.1.1|2022-02-15	84,05
EPI_ISL_11095760	BA.1.1	BA.1.1	BA.1.1_3	BFC-HMN-22032010383|EPI_ISL_11095760|France|BA.1.1|2022-02-21	91,95
EPI_ISL_11096815	BA.2	BA.1	BA.1_4	DA10355092|EPI_ISL_11096815|Sweden|BA.2|2022-02-21	95,94
EPI_ISL_11099555	BA.2.3	BA.2	BA.2_1	QEUH-3997F4B|EPI_ISL_11099555|United Kingdom|BA.2.3|2022-03-07	99,81
EPI_ISL_11099658	BA.2	BA.2	BA.2_1	QEUH-39931D0|EPI_ISL_11099658|United Kingdom|BA.2|2022-03-07	99,81
EPI_ISL_11101289	BA.1	BA.1	BA.1_4	VA-CDC-2-5699259|EPI_ISL_11101289|USA|BA.1|2022-02-06	99,64
EPI_ISL_11105046	BA.1	BA.1.15	BA.1.15	NICD-N34624|EPI_ISL_11105046|South Africa|BA.1|2021-12-21	96,16
EPI_ISL_11107798	BA.1.1	B.1.1.318	B.1.1.318_1	OCC-ChuMtp-99226409938|EPI_ISL_11107798|France|BA.1.1|2022-01-25	86,82
EPI_ISL_11108498	BA.1.1	BA.1.1	BA.1.1_2	LAZ-AMC-2202211258-DS|EPI_ISL_11108498|Italy|BA.1.1|2022-02-21	99,12
EPI_ISL_11109370	BA.2	BA.2.12	BA.2.12	UP-NCDC-8109232|EPI_ISL_11109370|India|BA.2|2022-02-04	75,10
EPI_ISL_11110694	BA.1.1	BA.1.1	BA.1.1_2	SIN_LANGEBIO_IMSS_5957|EPI_ISL_11110694|Mexico|BA.1.1|2022-02-11	99,64
EPI_ISL_11110701	BA.1.1	BA.1.1	BA.1.1_2	MIC_LANGEBIO_IMSS_5964|EPI_ISL_11110701|Mexico|BA.1.1|2022-02-10	99,54
EPI_ISL_11110870	BA.1	BA.1.1	BA.1.1_2	CMX_LANGEBIO_IMSS_6123|EPI_ISL_11110870|Mexico|BA.1|2022-02-21	98,11
EPI_ISL_11113034	BA.2	BA.2	BA.2_1	DCGC-424804|EPI_ISL_11113034|Denmark|BA.2|2022-03-10	94,87
EPI_ISL_11113292	BA.2	BA.2	BA.2_1	DCGC-425064|EPI_ISL_11113292|Denmark|BA.2|2022-03-11	99,81
EPI_ISL_11113625	BA.1	BA.1	BA.1_1	DCGC-425253|EPI_ISL_11113625|Denmark|BA.1|2022-03-07	99,12
EPI_ISL_11114973	BA.2	A	A_17	DCGC-426120|EPI_ISL_11114973|Denmark|BA.2|2022-03-11	99,98
EPI_ISL_11115106	BA.2	A	A_17	DCGC-426253|EPI_ISL_11115106|Denmark|BA.2|2022-03-10	99,81
EPI_ISL_11116357	BA.2	BA.2	BA.2_1	DCGC-426868|EPI_ISL_11116357|Denmark|BA.2|2022-03-10	99,81
EPI_ISL_11116743	BA.1.1	A	A_3	C113349|EPI_ISL_11116743|Kenya|BA.1.1|2021-12-22	61,90
EPI_ISL_11117320	BA.1.1.2	BA.1.1	BA.1.1_2	TKYkbm9877|EPI_ISL_11117320|Japan|BA.1.1.2|2022-02-12	99,64
EPI_ISL_11123109	BA.1.1	BA.1.1	BA.1.1_1	LSPA-3A4295F|EPI_ISL_11123109|United Kingdom|BA.1.1|2022-03-11	99,64
EPI_ISL_11123399	BA.2	A	A_14	PLYM-3A2E27C|EPI_ISL_11123399|United Kingdom|BA.2|2022-03-11	99,81
EPI_ISL_11123822	BA.2	BA.2	BA.2_1	PLYM-3A2E28B|EPI_ISL_11123822|United Kingdom|BA.2|2022-03-11	99,81
EPI_ISL_11124644	BA.2	BA.2	BA.2_1	MILK-3A0815D|EPI_ISL_11124644|United Kingdom|BA.2|2022-03-09	99,81
EPI_ISL_11125397	BA.1.1	BA.1.1	BA.1.1_2	GA-CDC-MMB14435871|EPI_ISL_11125397|USA|BA.1.1|2022-02-17	98,32

EPI_ISL_11126474	BA.1.1.2	BA.1.1	BA.1.1_3	CO-CDC-MMB14620120|EPI_ISL_11126474|USA|BA.1.1.2|2022-03-03	99,64
EPI_ISL_11126536	BA.2	A	A_14	MILK-3A0F6EE|EPI_ISL_11126536|United Kingdom|BA.2|2022-03-09	99,81
EPI_ISL_11126805	BA.2	BA.2	BA.2_1	PLYM-39FC25A|EPI_ISL_11126805|United Kingdom|BA.2|2022-03-08	94,09
EPI_ISL_11127146	BA.2	A	A_17	NEWC-39F8D5D|EPI_ISL_11127146|United Kingdom|BA.2|2022-03-08	89,03
EPI_ISL_11129630	BA.2.3	A	A_14	MILK-3A11F39|EPI_ISL_11129630|United Kingdom|BA.2.3|2022-03-10	99,81
EPI_ISL_11130899	BA.2	A	A_14	MILK-3A0BAAA|EPI_ISL_11130899|United Kingdom|BA.2|2022-03-08	99,81
EPI_ISL_11133431	BA.2.37	A	A_4	QEUH-399AA0B|EPI_ISL_11133431|United Kingdom|BA.2.37|2022-03-07	99,81
EPI_ISL_11134342	BA.1.1	BA.1	BA.1_4	CA-CDPH-3000313860|EPI_ISL_11134342|USA|BA.1.1|2022-02-11	84,30
EPI_ISL_11134550	BA.1.1.14	BA.1.1	BA.1.1_3	CA-CDPH-3000320597|EPI_ISL_11134550|USA|BA.1.1.14|2022-02-16	88,07
EPI_ISL_11135044	BA.1.15	BA.1.15	BA.1.15	CA-CDPH-500046964|EPI_ISL_11135044|USA|BA.1.15|2022-01-17	99,64
EPI_ISL_11135253	BA.1.1.18	BA.1.1	BA.1.1_3	CA-CDPH-500047284|EPI_ISL_11135253|USA|BA.1.1.18|2022-01-16	99,64
EPI_ISL_11138151	BA.1.1	BA.1.1	BA.1.1_2	UT-UPHL-220315365034|EPI_ISL_11138151|USA|BA.1.1|2022-03-04	99,64
EPI_ISL_11138960	BA.1.1	BA.1.1	BA.1.1_3	VC-FISABIO-103283|EPI_ISL_11138960|Spain|BA.1.1|2022-01-25	99,64
EPI_ISL_11139831	BA.1.17	BA.1.15	BA.1.15	VC-FISABIO-103232|EPI_ISL_11139831|Spain|BA.1.17|2022-01-23	99,64
EPI_ISL_11140879	BA.1.1	BA.1.1	BA.1.1_2	VC-FISABIO-103556|EPI_ISL_11140879|Spain|BA.1.1|2022-02-04	96,03
EPI_ISL_11144114	BA.1.1.2	BA.1.1	BA.1.1_2	TKYkbm10492|EPI_ISL_11144114|Japan|BA.1.1.2|2022-02-17	99,64
EPI_ISL_11144908	BA.1.1	BA.1	BA.1_4	SD-UMGC-35407|EPI_ISL_11144908|USA|BA.1.1|2022-01-03	90,73
EPI_ISL_11145090	BA.1.1	A	A_1	MN-UMGC-29638|EPI_ISL_11145090|USA|BA.1.1|2021-12-30	66,93
EPI_ISL_11145982	BA.1.1	BA.1.1	BA.1.1_3	CA-SEARCH-75569|EPI_ISL_11145982|USA|BA.1.1|2022-01-03	99,48
EPI_ISL_11148214	BA.1	BA.1	BA.1_3	SP-FIOCRUZ-64029CE|EPI_ISL_11148214|Brazil|BA.1|2022-01-15	99,52
EPI_ISL_11148296	BA.1.2	BA.1	BA.1_1	IDF-ChSV-2220222357|EPI_ISL_11148296|France|BA.1.2|2022-02-22	79,72
EPI_ISL_11148460	BA.1.1	BA.1.1	BA.1.1_2	AZ-ASU53480|EPI_ISL_11148460|USA|BA.1.1|2022-01-15	93,15
EPI_ISL_11148754	BA.2.3	BA.2	BA.2_1	KDCA31666|EPI_ISL_11148754|South Korea|BA.2.3|2022-02-18	99,79
EPI_ISL_11148935	BA.2	BA.2	BA.2_1	KDCA31847|EPI_ISL_11148935|South Korea|BA.2|2022-02-20	99,79
EPI_ISL_11149729	BA.2	BA.2	BA.2_1	SIC-AOUP-UNIPA_97022_2022|EPI_ISL_11149729|Italy|BA.2|2022-02-23	97,48
EPI_ISL_11150585	BA.2	A	A_14	MILK-3A4DCC4|EPI_ISL_11150585|United Kingdom|BA.2|2022-03-11	99,81
EPI_ISL_11150690	BA.2	A	A_17	MILK-3A4F943|EPI_ISL_11150690|United Kingdom|BA.2|2022-03-11	99,81
EPI_ISL_11152417	BA.2	A	A_14	PLYM-3A41B3C|EPI_ISL_11152417|United Kingdom|BA.2|2022-03-10	99,81
EPI_ISL_11153500	BA.2	A	A_14	LSPA-3A449B7|EPI_ISL_11153500|United Kingdom|BA.2|2022-03-11	99,81
EPI_ISL_11153562	BA.2	BA.2.10	BA.2.10	LSPA-3A44AF0|EPI_ISL_11153562|United Kingdom|BA.2|2022-03-11	99,81
EPI_ISL_11154679	BA.2	BA.2	BA.2_1	TL-13590|EPI_ISL_11154679|Romania|BA.2|2022-02-18	99,81
EPI_ISL_11155062	BA.1.17	BA.1.15	BA.1.15	PHEC-5X03AZC0|EPI_ISL_11155062|United Kingdom|BA.1.17|2022-01-08	96,81
EPI_ISL_11155276	BA.1.1.1	BA.1	BA.1_4	PHEC-5X04AZ0B|EPI_ISL_11155276|United Kingdom|BA.1.1.1|2022-01-06	96,81
EPI_ISL_11157143	BA.1.17.2	BA.1.17.2	BA.1.17.2	PHWC-PE7YF3|EPI_ISL_11157143|United Kingdom|BA.1.17.2|2022-02-02	99,64
EPI_ISL_11159344	BA.1.1	BA.1.1	BA.1.1_2	UT-EMC-545|EPI_ISL_11159344|Netherlands|BA.1.1|2022-03-02	95,99
EPI_ISL_11160412	BA.2	BA.2.10	BA.2.10	1070235|EPI_ISL_11160412|South Africa|BA.2|2022-01-06	96,03
EPI_ISL_11163499	BA.1.20	BA.1.15	BA.1.15	OR-IVY-A93J27T4|EPI_ISL_11163499|USA|BA.1.20|2022-02-03	99,64
EPI_ISL_11163802	BA.1.20	BA.1	BA.1_3	WV-WVU-WV129063|EPI_ISL_11163802|USA|BA.1.20|2022-01-10	96,03
EPI_ISL_11164457	BA.1.1	BA.1.1	BA.1.1_2	WV-WVU-WV129565|EPI_ISL_11164457|USA|BA.1.1|2022-01-19	99,64
EPI_ISL_11168883	BA.1.1.1	BA.1.1	BA.1.1_2	AG-ETHZ-36573197|EPI_ISL_11168883|Switzerland|BA.1.1.1|2022-03-01	96,79
EPI_ISL_11170825	BA.1.1	BA.1.1	BA.1.1_2	CA-HLX-STM-8GRB29F2V|EPI_ISL_11170825|USA|BA.1.1|2022-02-23	97,82
EPI_ISL_11172188	BA.1	BA.1	BA.1_3	IL-RIPHL_90199_G|EPI_ISL_11172188|USA|BA.1|2022-01-25	91,97
EPI_ISL_11173206	BA.1.15	BA.1.15	BA.1.15	TX-HMH-MCoV-85843|EPI_ISL_11173206|USA|BA.1.15|2021-12-23	99,64
EPI_ISL_11173789	BA.1.15	BA.1	BA.1_4	TX-HMH-MCoV-72533|EPI_ISL_11173789|USA|BA.1.15|2021-12-25	98,26
EPI_ISL_11176305	BA.1	BA.1.1	BA.1.1_3	VIC44708|EPI_ISL_11176305|Australia|BA.1|2022-02-21	99,64
EPI_ISL_11176804	BA.2.3	XAL	XAL	VIC45125|EPI_ISL_11176804|Australia|BA.2.3|2022-03-04	99,81
EPI_ISL_11176922	BA.1.1	BA.1	BA.1_4	CMX-INMEGEN-62-272|EPI_ISL_11176922|Mexico|BA.1.1|2022-01-03	95,25
EPI_ISL_11178291	BA.1.1	BA.1.1	BA.1.1_2	CA-CDC-STM-QBJ22C3C4|EPI_ISL_11178291|USA|BA.1.1|2022-03-06	99,64
EPI_ISL_11178948	BA.2	A	A_14	WI-UW-9879|EPI_ISL_11178948|USA|BA.2|2022-03-01	91,18
EPI_ISL_11179841	BA.2	BA.2	BA.2_1	MBLG-CTMAPF21756935|EPI_ISL_11179841|Belgium|BA.2|2022-03-13	99,81
EPI_ISL_11181882	BA.2	BA.2	BA.2_1	PLYM-3A70211|EPI_ISL_11181882|United Kingdom|BA.2|2022-03-12	99,81
EPI_ISL_11182795	BA.2	A	A_14	BRBR-3A4C278|EPI_ISL_11182795|United Kingdom|BA.2|2022-03-10	99,81
EPI_ISL_11184998	BA.2	BA.2	BA.2_1	QEUH-3A5DE70|EPI_ISL_11184998|United Kingdom|BA.2|2022-03-12	99,81
EPI_ISL_11185740	BA.2	A	A_14	BRBR-3A75452|EPI_ISL_11185740|United Kingdom|BA.2|2022-03-13	99,81
EPI_ISL_11185980	BA.2	A	A_17	QEUH-3A5BAAB|EPI_ISL_11185980|United Kingdom|BA.2|2022-03-12	99,81
EPI_ISL_11186116	BA.2	BA.2	BA.2_1	BRBR-3A6F172|EPI_ISL_11186116|United Kingdom|BA.2|2022-03-12	99,81
EPI_ISL_11186997	BA.2	A	A_14	QEUH-3A67D49|EPI_ISL_11186997|United Kingdom|BA.2|2022-03-12	99,81
EPI_ISL_11187842	BA.2	A	A_17	MILK-3A5F315|EPI_ISL_11187842|United Kingdom|BA.2|2022-03-13	99,81
EPI_ISL_11188490	BA.2.9	A	A_17	LSPA-3A621AC|EPI_ISL_11188490|United Kingdom|BA.2.9|2022-03-12	99,81
EPI_ISL_11192592	BA.1.1	BA.1	BA.1_4	CA-HLX-STM-MB8RNSJG7|EPI_ISL_11192592|USA|BA.1.1|2022-02-26	76,66
EPI_ISL_11194167	BD.1	BD.1	BD.1	PLYM-3A2E85C|EPI_ISL_11194167|United Kingdom|BD.1|2022-03-10	99,64
EPI_ISL_11194854	BA.2	A	A_14	HSLL-3A9B0BC|EPI_ISL_11194854|United Kingdom|BA.2|2022-03-13	99,81
EPI_ISL_11195587	BA.2	BA.2	BA.2_1	MILK-3A971DB|EPI_ISL_11195587|United Kingdom|BA.2|2022-03-13	94,09
EPI_ISL_11197349	BA.2.37	A	A_14	MILK-3A87684|EPI_ISL_11197349|United Kingdom|BA.2.37|2022-03-13	99,81
EPI_ISL_11198007	BA.2	A	A_14	MILK-3A8D33C|EPI_ISL_11198007|United Kingdom|BA.2|2022-03-13	99,81
EPI_ISL_11198462	BA.2	BA.2	BA.2_1	QEUH-3A8C591|EPI_ISL_11198462|United Kingdom|BA.2|2022-03-13	99,81
EPI_ISL_11199082	XE	A	A_14	MILK-3A928DB|EPI_ISL_11199082|United Kingdom|XE|2022-03-13	99,81
EPI_ISL_11199427	BA.1.1.18	BA.1.1	BA.1.1_3	NY-SUNYQB-5017045|EPI_ISL_11199427|USA|BA.1.1.18|2022-01-28	99,64
EPI_ISL_11202252	BA.1.1	BA.1.1	BA.1.1_2	NY-SUNYQB-61210500809174|EPI_ISL_11202252|USA|BA.1.1|2022-01-04	96,64
EPI_ISL_11202393	BA.1.15	BA.1.15	BA.1.15	NY-SUNYQB-61210500906277|EPI_ISL_11202393|USA|BA.1.15|2022-01-19	94,89
EPI_ISL_11202635	BA.1.1	BA.1.1	BA.1.1_2	NY-SUNYQB-61210500916033|EPI_ISL_11202635|USA|BA.1.1|2022-01-16	93,76
EPI_ISL_11203306	BA.1.15	BA.1	BA.1_4	NY-SUNYQB-61210501507202|EPI_ISL_11203306|USA|BA.1.15|2022-02-05	99,64
EPI_ISL_11204093	BA.1.1	BA.1.1	BA.1.1_2	NY-SUNYQB-61210550406690|EPI_ISL_11204093|USA|BA.1.1|2022-01-15	96,64
EPI_ISL_11204290	BA.1.1	BA.1.1	BA.1.1_2	NY-SUNYQB-61210552802382|EPI_ISL_11204290|USA|BA.1.1|2022-01-04	88,27
EPI_ISL_11204485	BA.1.1	BA.1	BA.1_4	NY-SUNYQB-61210553614580|EPI_ISL_11204485|USA|BA.1.1|2022-01-20	93,30
EPI_ISL_11207850	BA.1.1	BA.1.1	BA.1.1_1	NIC_SNI_SEQ7945|EPI_ISL_11207850|Thailand|BA.1.1|2022-01-24	99,64
EPI_ISL_11208209	BA.2.9	A	A_17	DCGC-430925|EPI_ISL_11208209|Denmark|BA.2.9|2022-03-14	99,81
EPI_ISL_11208732	BA.2	BA.2	BA.2_1	DCGC-431454|EPI_ISL_11208732|Denmark|BA.2|2022-01-29	99,81
EPI_ISL_11208785	BA.2.9	A	A_17	DCGC-431508|EPI_ISL_11208785|Denmark|BA.2.9|2022-03-14	99,79
EPI_ISL_11209052	BA.2	A	A_17	DCGC-431777|EPI_ISL_11209052|Denmark|BA.2|2022-03-13	99,79
EPI_ISL_11209101	BA.2.9	BA.2	BA.2_1	DCGC-431826|EPI_ISL_11209101|Denmark|BA.2.9|2022-03-13	99,79
EPI_ISL_11210721	BA.2	A	A_17	DCGC-433332|EPI_ISL_11210721|Denmark|BA.2|2022-03-09	99,98
EPI_ISL_11210989	BA.2	BA.2	BA.2_1	DCGC-433602|EPI_ISL_11210989|Denmark|BA.2|2022-01-31	99,81
EPI_ISL_11211152	BA.2	BA.2	BA.2_1	DCGC-433766|EPI_ISL_11211152|Denmark|BA.2|2022-03-14	99,81
EPI_ISL_11212519	BA.1.1	BA.1.1	BA.1.1_2	NIC_NKI_SEQ8780|EPI_ISL_11212519|Thailand|BA.1.1|2022-02	96,60
EPI_ISL_11212662	BA.2	BA.2	BA.2_1	NEWC-3ABE235|EPI_ISL_11212662|United Kingdom|BA.2|2022-03-14	99,81
EPI_ISL_11213060	BA.2.9	BA.2	BA.2_1	NEWC-3AB816B|EPI_ISL_11213060|United Kingdom|BA.2.9|2022-03-14	99,81
EPI_ISL_11215298	BA.2.23	BA.2.10	BA.2.10	ALDP-3AA8A63|EPI_ISL_11215298|United Kingdom|BA.2.23|2022-03-14	99,81
EPI_ISL_11215568	BA.2	BA.2	BA.2_1	BRBR-3AA7DA7|EPI_ISL_11215568|United Kingdom|BA.2|2022-03-14	99,81
EPI_ISL_11217094	BA.2	A	A_14	HDF-biopath-7742776862|EPI_ISL_11217094|France|BA.2|2022-03-10	99,03
EPI_ISL_11217185	BA.2	BA.2	BA.2_1	HDF-biopath-7747896574|EPI_ISL_11217185|France|BA.2|2022-03-10	99,03
EPI_ISL_11217469	BA.2	BA.2	BA.2_1	HDF-biopath-7746638829|EPI_ISL_11217469|France|BA.2|2022-03-06	98,23
EPI_ISL_11218625	BA.2	A	A_14	HDF-biopath-7739444961|EPI_ISL_11218625|France|BA.2|2022-03-04	99,12
EPI_ISL_11219240	BA.1.17	BA.1	BA.1_4	NC-CHN-01006109|EPI_ISL_11219240|Spain|BA.1.17|2022-03-04	96,72
EPI_ISL_11219488	BA.2	BA.2	BA.2_1	NC-CHN-01006358|EPI_ISL_11219488|Spain|BA.2|2022-03-03	99,81
EPI_ISL_11219498	BA.2	A	A_17	NC-CHN-01006368|EPI_ISL_11219498|Spain|BA.2|2022-02-25	99,81
EPI_ISL_11219871	BA.1.1	BA.1.1	BA.1.1_3	CVL-HMN-22032020441|EPI_ISL_11219871|France|BA.1.1|2022-02-07	80,18
EPI_ISL_11220475	BA.1.1	BA.1.1	BA.1.1_2	SS6615|EPI_ISL_11220475|Kenya|BA.1.1|2022-01-04	84,91
EPI_ISL_11220512	BA.1.1	BA.1.1	BA.1.1_2	SS6677|EPI_ISL_11220512|Kenya|BA.1.1|2022-01-20	88,51
EPI_ISL_11221130	BA.1.1.14	BA.1.1	BA.1.1_3	CVR14218|EPI_ISL_11221130|United Kingdom|BA.1.1.14|2022-02-21	99,56
EPI_ISL_11222237	BA.2	A	A_14	NIRE-01af2f|EPI_ISL_11222237|United Kingdom|BA.2|2022-03-05	99,12

EPI_ISL_11223013	BA.2	BA.2	BA.2_1	HDF-biopath-7743543030|EPI_ISL_11223013|France|BA.2|2022-02-21	97,18
EPI_ISL_11223104	BA.1.1	BA.1.1	BA.1.1_2	PHEC-5X076Z5F|EPI_ISL_11223104|United Kingdom|BA.1.1|2022	99,12
EPI_ISL_11223298	BA.1.17.2	BA.1.17.2	BA.1.17.2	PHEC-YYD8CZW|EPI_ISL_11223298|United Kingdom|BA.1.17.2|2022-03-03	99,64
EPI_ISL_11224181	BA.2	BA.2	BA.2_1	PHEC-YYDENCU|EPI_ISL_11224181|United Kingdom|BA.2|2022	99,77
EPI_ISL_11224392	BA.2	A	A_14	PHEC-YYDEW8M|EPI_ISL_11224392|United Kingdom|BA.2|2022	99,79
EPI_ISL_11226701	BA.1.1	BA.1.1	BA.1.1_3	PHEP-YYRCTZR|EPI_ISL_11226701|United Kingdom|BA.1.1|2022-03-07	99,58
EPI_ISL_11226911	BA.2	BA.2	BA.2_1	PHEP-YYRPYPE|EPI_ISL_11226911|United Kingdom|BA.2|2022-02-28	99,81
EPI_ISL_11227028	BA.1.1	BA.1.1	BA.1.1_2	PHWC-PE66HK|EPI_ISL_11227028|United Kingdom|BA.1.1|2022-03-02	99,62
EPI_ISL_11228638	BA.2.10	BA.2	BA.2_1	AP-CCMB-CIC6268|EPI_ISL_11228638|India|BA.2.10|2022-02-15	99,16
EPI_ISL_11233153	BA.2	A	A_14	PHWC-PJYMDC|EPI_ISL_11233153|United Kingdom|BA.2|2022-03-03	99,81
EPI_ISL_11235631	BA.1.15	BA.1	BA.1_2	TX-HMH-MCoV-95810|EPI_ISL_11235631|USA|BA.1.15|2022-03-09	99,64
EPI_ISL_11242083	BA.2	A	A_14	ARA-HCL022040632301|EPI_ISL_11242083|France|BA.2|2022-03-03	99,81
EPI_ISL_11243258	BA.2	A	A_17	NAQ-HCL722001063001|EPI_ISL_11243258|France|BA.2|2022-02-28	99,81
EPI_ISL_11243370	BA.1.1	BA.1.1	BA.1.1_3	NAQ-HCL722001074701|EPI_ISL_11243370|France|BA.1.1|2022-02-28	99,64
EPI_ISL_11243973	BA.1.18	BA.1	BA.1_4	ARA-HCL722001120901|EPI_ISL_11243973|France|BA.1.18|2022-02-14	99,64
EPI_ISL_11247358	BA.1.15	BA.1.15	BA.1.15	NJ-GBW-EWR000191|EPI_ISL_11247358|USA|BA.1.15|2022-01-13	89,16
EPI_ISL_11247536	BA.1.18	BA.1	BA.1_1	NY-NYCPHL-010703|EPI_ISL_11247536|USA|BA.1.18|2022-03-03	88,88
EPI_ISL_11248604	BA.2.9	A	A_17	IDF-IPP17080|EPI_ISL_11248604|France|BA.2.9|2022-03-04	99,81
EPI_ISL_11248799	BA.2	BA.2	BA.2_1	AZDelta-2210-18312|EPI_ISL_11248799|Belgium|BA.2|2022-03-17	97,94
EPI_ISL_11249348	BA.1	BA.1.1	BA.1.1_2	ON-PHL-22-11677|EPI_ISL_11249348|Canada|BA.1|2022-03-07	90,98
EPI_ISL_11249584	BA.1.1	BA.1.1	BA.1.1_2	ON-PHL-22-11918|EPI_ISL_11249584|Canada|BA.1.1|2022-03-10	90,98
EPI_ISL_11250176	BA.1.1	BA.1.1	BA.1.1_2	ON-PHL-22-12703|EPI_ISL_11250176|Canada|BA.1.1|2022-03-15	90,94
EPI_ISL_11250360	BA.2.38	BA.2.12.1	BA.2.12.1	ON-PHL-22-12899|EPI_ISL_11250360|Canada|BA.2.38|2022-03-11	88,06
EPI_ISL_11250910	BA.1.1	BA.1.1	BA.1.1_3	MA-NEIDL-04834|EPI_ISL_11250910|USA|BA.1.1|2022-02-20	96,87
EPI_ISL_11250943	BA.1.1	BA.1.1	BA.1.1_2	MA-NEIDL-04867|EPI_ISL_11250943|USA|BA.1.1|2022-02-21	96,70
EPI_ISL_11253003	BA.1.1	BA.1.1	BA.1.1_2	NV-CDC-QDX34496293|EPI_ISL_11253003|USA|BA.1.1|2022-03-04	99,64
EPI_ISL_11260576	BA.1	BA.1.15	BA.1.15	LHUB-ULB_25206754|EPI_ISL_11260576|Belgium|BA.1|2022-01-16	99,64
EPI_ISL_11263785	BA.2	A	A_17	31479|EPI_ISL_11263785|Croatia|BA.2|2022-02-15	98,03
EPI_ISL_11263807	BA.1.1	BA.1.1	BA.1.1_2	NORT-YNN8OQY|EPI_ISL_11263807|United Kingdom|BA.1.1|2022	99,64
EPI_ISL_11268849	BA.1.1	BA.1.1	BA.1.1_2	FL-BPHL-3821|EPI_ISL_11268849|USA|BA.1.1|2022-01-10	99,64
EPI_ISL_11269535	BA.2	A	A_14	QEUH-3AD9C09|EPI_ISL_11269535|United Kingdom|BA.2|2022-03-14	99,81
EPI_ISL_11269687	BA.2	BA.2	BA.2_1	QEUH-3AD9902|EPI_ISL_11269687|United Kingdom|BA.2|2022-03-14	99,81
EPI_ISL_11271065	BA.2	A	A_17	MILK-3AE4BC2|EPI_ISL_11271065|United Kingdom|BA.2|2022-03-15	99,81
EPI_ISL_11271135	BA.2	BA.2	BA.2_1	QEUH-3ADF580|EPI_ISL_11271135|United Kingdom|BA.2|2022-03-14	99,81
EPI_ISL_11272822	BA.2.1	A	A_14	ALDP-3AC87FD|EPI_ISL_11272822|United Kingdom|BA.2.1|2022-03-14	99,81
EPI_ISL_11273845	BA.2	A	A_14	QEUH-3AB0B38|EPI_ISL_11273845|United Kingdom|BA.2|2022-03-14	99,81
EPI_ISL_11273962	BA.2.9	A	A_17	DCGC-434273|EPI_ISL_11273962|Denmark|BA.2.9|2022-03-16	99,81
EPI_ISL_11274585	BA.2	A	A_17	BRBR-3A7F1F7|EPI_ISL_11274585|United Kingdom|BA.2|2022-03-13	99,81
EPI_ISL_11275761	BA.2	A	A_17	BRBR-3ACF680|EPI_ISL_11275761|United Kingdom|BA.2|2022-03-15	99,81
EPI_ISL_11275866	BA.1.1	BA.1.1	BA.1.1_2	LSPA-3ACFAEE|EPI_ISL_11275866|United Kingdom|BA.1.1|2022-03-15	99,64
EPI_ISL_11276045	BA.2	A	A_14	QEUH-3AC02F4|EPI_ISL_11276045|United Kingdom|BA.2|2022-03-13	99,81
EPI_ISL_11278101	BA.2	A	A_17	ALDP-3ACB271|EPI_ISL_11278101|United Kingdom|BA.2|2022-03-14	99,81
EPI_ISL_11279470	BA.2	BA.2	BA.2_1	QEUH-3AB59DF|EPI_ISL_11279470|United Kingdom|BA.2|2022-03-14	99,81
EPI_ISL_11280122	BA.1.1	BA.1.1	BA.1.1_2	FL-BPHL-3883|EPI_ISL_11280122|USA|BA.1.1|2022-02-25	99,64
EPI_ISL_11280206	BA.2	BA.2	BA.2_1	PLYM-3AD4F50|EPI_ISL_11280206|United Kingdom|BA.2|2022-03-14	99,81
EPI_ISL_11280518	BA.2	BA.2.10	BA.2.10	NEWC-3AD4CA4|EPI_ISL_11280518|United Kingdom|BA.2|2022-03-15	94,60
EPI_ISL_11280784	BA.2.9	A	A_17	DCGC-436333|EPI_ISL_11280784|Denmark|BA.2.9|2022-03-14	99,81
EPI_ISL_11281432	BA.2	A	A_17	DCGC-436580|EPI_ISL_11281432|Denmark|BA.2|2022-03-15	99,81
EPI_ISL_11284933	BA.1.1	BA.1.1	BA.1.1_2	QEUH-3A2A999|EPI_ISL_11284933|United Kingdom|BA.1.1|2022-03-10	99,64
EPI_ISL_11289096	BA.2	A	A_14	QEUH-3A7AAD3|EPI_ISL_11289096|United Kingdom|BA.2|2022-03-13	99,81
EPI_ISL_11293139	BA.1.1	BA.1.1	BA.1.1_2	UT-UPHL-220318516114|EPI_ISL_11293139|USA|BA.1.1|2022-02-15	99,64
EPI_ISL_11293286	BA.1.15	BA.1	BA.1_3	UT-UPHL-220318862932|EPI_ISL_11293286|USA|BA.1.15|2022-02-14	99,64
EPI_ISL_11293440	BA.1.1	BA.1.1	BA.1.1_2	UT-UPHL-220318199186|EPI_ISL_11293440|USA|BA.1.1|2022-02-10	99,64
EPI_ISL_11295367	BA.2.9.2	BA.2	BA.2_1	CT-Broad-CRSP_JQ46GV3M2GLKTJTC|EPI_ISL_11295367|USA|BA.2.9.2|2022-03-10	95,15
EPI_ISL_11295825	BA.1.1	BA.1.1	BA.1.1_2	MA-Broad-CRSP_JH25BWLO675U73D6|EPI_ISL_11295825|USA|BA.1.1|2022-03-11	99,64
EPI_ISL_11296005	BA.1.1	BA.1.1	BA.1.1_2	SMC-7043695|EPI_ISL_11296005|Israel|BA.1.1|2022-01-22	91,81
EPI_ISL_11296834	BA.2	BA.2	BA.2_1	VT-Broad-CRSP_RT24UCVUOKHNFIWA|EPI_ISL_11296834|USA|BA.2|2022-03-09	96,30
EPI_ISL_11297428	BA.2	A	A_17	01_SE100_22CS103146|EPI_ISL_11297428|Sweden|BA.2|2022-03-09	99,79
EPI_ISL_11298585	BA.2	A	A_4	BL-ETHZ-36549368|EPI_ISL_11298585|Switzerland|BA.2|2022-02-28	99,81
EPI_ISL_11298662	BA.1.1	BA.1.1	BA.1.1_2	TI-ETHZ-36542130|EPI_ISL_11298662|Switzerland|BA.1.1|2022-02-28	96,81
EPI_ISL_11299170	BA.1.1	BA.1	BA.1_4	CA-HLX-STM-8BZ8TFKXF|EPI_ISL_11299170|USA|BA.1.1|2022-02-25	97,44
EPI_ISL_11299250	BA.1.1	BA.1.1	BA.1.1_2	CA-HLX-STM-RK7Q2YCTQ|EPI_ISL_11299250|USA|BA.1.1|2022-03-01	96,45
EPI_ISL_11302598	BA.2.64	A	A_14	IC-5830|EPI_ISL_11302598|Japan|BA.2.64|2022-02-26	99,81
EPI_ISL_11303676	BA.2	BA.2	BA.2_1	QEUH-3B0F1BF|EPI_ISL_11303676|United Kingdom|BA.2|2022-03-17	99,81
EPI_ISL_11303838	BA.2	A	A_17	PLYM-3B0F629|EPI_ISL_11303838|United Kingdom|BA.2|2022-03-14	99,81
EPI_ISL_11305306	BA.2	A	A_14	QEUH-3AEC6D8|EPI_ISL_11305306|United Kingdom|BA.2|2022-03-16	99,81
EPI_ISL_11307160	BA.2.3	BA.2	BA.2_1	BRBR-3B0858D|EPI_ISL_11307160|United Kingdom|BA.2.3|2022-03-15	99,81
EPI_ISL_11309307	BA.2.3	A	A_14	QEUH-3ABAEAE|EPI_ISL_11309307|United Kingdom|BA.2.3|2022-03-14	99,81
EPI_ISL_11310024	BA.1.1	BA.1	BA.1_3	BA_22_00015470|EPI_ISL_11310024|Slovakia|BA.1.1|2022-02-27	96,20
EPI_ISL_11310057	BA.2	A	A_4	BA_22_00016172|EPI_ISL_11310057|Slovakia|BA.2|2022-02-23	99,81
EPI_ISL_11310248	BA.2.67	A	A_14	BA_22_00014440|EPI_ISL_11310248|Slovakia|BA.2.67|2022-02-10	99,81
EPI_ISL_11312215	BA.2.9	BA.2	BA.2_1	SMC-7049659|EPI_ISL_11312215|Israel|BA.2.9|2022-03-02	87,85
EPI_ISL_11313720	BA.1.1	BA.1	BA.1_4	SMC-7049459|EPI_ISL_11313720|Israel|BA.1.1|2022-02-22	89,24
EPI_ISL_11314591	BA.1.17.2	BA.1.17.2	BA.1.17.2	PHEC-YYDKCUS|EPI_ISL_11314591|United Kingdom|BA.1.17.2|2022-03-06	94,43
EPI_ISL_11317049	BA.2	BA.2	BA.2_1	DCGC-439654|EPI_ISL_11317049|Denmark|BA.2|2022-03-12	99,79
EPI_ISL_11317239	BA.2	BA.2	BA.2_1	GES-HMN-22032040156|EPI_ISL_11317239|France|BA.2|2022-02-28	99,81
EPI_ISL_11317402	BA.2	BA.2	BA.2_1	ARA-HMN-22032080185|EPI_ISL_11317402|France|BA.2|2022-02-28	95,17
EPI_ISL_11319428	BA.1.1	BA.1.1	BA.1.1_2	SMC-7047828|EPI_ISL_11319428|Israel|BA.1.1|2022-02-22	96,26
EPI_ISL_11322188	BA.2	BA.2	BA.2_1	PHWC-PJNFH5|EPI_ISL_11322188|United Kingdom|BA.2|2022-03-07	89,05
EPI_ISL_11327637	BA.2	A	A_14	QC-L00439354001|EPI_ISL_11327637|Canada|BA.2|2022-01-21	99,81
EPI_ISL_11327838	BA.1.15	BA.1.15	BA.1.15	QC-1nIFO-U1317069IA|EPI_ISL_11327838|Canada|BA.1.15|2022-01-31	99,64
EPI_ISL_11328062	BA.1.1	BA.1.1	BA.1.1_2	WA-S19118|EPI_ISL_11328062|USA|BA.1.1|2022-01-16	99,64
EPI_ISL_11328239	BA.2	BA.2	BA.2_1	BRE-IPP18048|EPI_ISL_11328239|France|BA.2|2022-03-07	99,81
EPI_ISL_11328878	BA.1.15	BA.1	BA.1_4	CA-SEARCH-116902|EPI_ISL_11328878|USA|BA.1.15|2021-12-26	99,03
EPI_ISL_11330232	BA.1.1	BA.1.1	BA.1.1_2	CA-OC-FG-270562|EPI_ISL_11330232|USA|BA.1.1|2022-01-24	91,03
EPI_ISL_11332098	BA.1.1.14	BA.1.1	BA.1.1_3	QLD0x00D39B|EPI_ISL_11332098|Australia|BA.1.1.14|2022-02-24	94,10
EPI_ISL_11332638	BA.2	A	A_14	UNIMAS-GHML1363|EPI_ISL_11332638|Malaysia|BA.2|2022-02-23	90,52
EPI_ISL_11333453	BA.2	BA.2	BA.2_1	CAM_IZSM_COLLI_TIGEM_RD20702409957|EPI_ISL_11333453|Italy|BA.2|2022-03-16	99,64
EPI_ISL_11335586	BA.2	BA.2	BA.2_1	LSPA-3B3364A|EPI_ISL_11335586|United Kingdom|BA.2|2022-03-17	99,81
EPI_ISL_11335838	BA.2	A	A_14	LSPA-3B32B2E|EPI_ISL_11335838|United Kingdom|BA.2|2022-03-17	99,81
EPI_ISL_11336443	BA.1.17.2	BA.1.17.2	BA.1.17.2	TN-ICMR-INSACOG-SPHL0643|EPI_ISL_11336443|India|BA.1.17.2|2021-12-23	99,50
EPI_ISL_11336588	BA.1.1	BA.1.1	BA.1.1_2	ALDP-3B38FE0|EPI_ISL_11336588|United Kingdom|BA.1.1|2022-03-17	99,64
EPI_ISL_11336683	BA.2	XAR	XAR	MILK-3B38C07|EPI_ISL_11336683|United Kingdom|BA.2|2022-03-18	99,81
EPI_ISL_11337081	BA.2	BA.2	BA.2_1	NEWC-3B1CFC4|EPI_ISL_11337081|United Kingdom|BA.2|2022-03-16	99,81
EPI_ISL_11337302	BA.2	A	A_17	ALDP-3B23EEB|EPI_ISL_11337302|United Kingdom|BA.2|2022-03-16	99,81
EPI_ISL_11337897	BA.2	A	A_4	LSPA-3B0D54C|EPI_ISL_11337897|United Kingdom|BA.2|2022-03-14	99,81
EPI_ISL_11338262	BA.2.1	BA.2	BA.2_1	LSPA-3B27AF2|EPI_ISL_11338262|United Kingdom|BA.2.1|2022-03-17	94,09
EPI_ISL_11339366	BA.2.9	BA.2	BA.2_1	PLYM-3B21348|EPI_ISL_11339366|United Kingdom|BA.2.9|2022-03-16	94,09
EPI_ISL_11340838	BA.2.23	BA.2	BA.2_1	LSPA-3B2F78A|EPI_ISL_11340838|United Kingdom|BA.2.23|2022-03-17	99,81
EPI_ISL_11341249	BA.2	BA.2	BA.2_1	LSPA-3AF5D40|EPI_ISL_11341249|United Kingdom|BA.2|2022-03-14	99,81
EPI_ISL_11342325	BA.1.1	BA.1.1	BA.1.1_2	CA-CDC-STM-BDNMDKT9Q|EPI_ISL_11342325|USA|BA.1.1|2022-02-25	99,64

EPI_ISL_11342624	BA.1.1.18	BA.1.1	BA.1.1_2	PA-CDC-STM-5MR64D2GV|EPI_ISL_11342624|USA|BA.1.1.18|2022-02-26	99,64
EPI_ISL_11342672	BA.1.1	BA.1.1	BA.1.1_2	FL-CDC-STM-KEXSYBVCK|EPI_ISL_11342672|USA|BA.1.1|2022-02-26	99,64
EPI_ISL_11343138	BA.1.1	BA.1.1	BA.1.1_2	AZ-CDC-STM-GNG3PYEBF|EPI_ISL_11343138|USA|BA.1.1|2022-03-01	99,64
EPI_ISL_11344511	BA.2	BA.2	BA.2_1	PHEC-YYDMFIZ|EPI_ISL_11344511|United Kingdom|BA.2|2022-03-09	99,77
EPI_ISL_11345482	BA.2	A	A_17	DCGC-440738|EPI_ISL_11345482|Denmark|BA.2|2022-03-16	99,81
EPI_ISL_11346128	BA.1.1.14	BA.1.1	BA.1.1_3	DCGC-441360|EPI_ISL_11346128|Denmark|BA.1.1.14|2022-03-19	99,64
EPI_ISL_11346204	BA.2.10	A	A_1	MN-IBSD-TBL-P14-043|EPI_ISL_11346204|India|BA.2.10|2022-01-18	56,38
EPI_ISL_11347008	BA.2	A	A_17	DCGC-441846|EPI_ISL_11347008|Denmark|BA.2|2022-03-19	99,81
EPI_ISL_11347135	BA.2.10.1	BA.2	BA.2_1	SCOT-6954|EPI_ISL_11347135|United Kingdom|BA.2.10.1|2022-03-03	99,79
EPI_ISL_11348530	BA.1.17.2	BA.1.17.2	BA.1.17.2	CERI-KRISP-K037319|EPI_ISL_11348530|South Africa|BA.1.17.2|2021-12-16	99,64
EPI_ISL_11349891	BA.1	BA.1.15	BA.1.15	UKDD-ZUUL-D202205549|EPI_ISL_11349891|Czech Republic|BA.1|2022-02-23	97,31
EPI_ISL_11350872	BA.1.1	BA.1.1	BA.1.1_2	NH-CDC-LC0562946|EPI_ISL_11350872|USA|BA.1.1|2022-03-05	99,64
EPI_ISL_11354695	BA.1.1	BA.1.1	BA.1.1_2	MA-CDC-STM-M43MM5VZ6|EPI_ISL_11354695|USA|BA.1.1|2022-03-10	99,64
EPI_ISL_11354729	BA.1.1	BA.1	BA.1_4	CA-CDC-STM-YYMP6Z67U|EPI_ISL_11354729|USA|BA.1.1|2022-03-10	96,11
EPI_ISL_11355144	BA.1.1	BA.1	BA.1_4	CA-CDC-STM-9ASVETEGA|EPI_ISL_11355144|USA|BA.1.1|2022-03-12	91,74
EPI_ISL_11360957	BA.2.3	BA.2	BA.2_1	PNG7591|EPI_ISL_11360957|Papua New Guinea|BA.2.3|2022-02-03	99,81
EPI_ISL_11361084	BA.2.3	BA.2.15	BA.2.15	PNG7676|EPI_ISL_11361084|Papua New Guinea|BA.2.3|2022-02-03	99,81
EPI_ISL_11366583	BA.1.1	BA.1.1	BA.1.1_3	BC-BCCDC-374442|EPI_ISL_11366583|Canada|BA.1.1|2022-02-16	99,64
EPI_ISL_11370032	BA.1.1	BA.1.1	BA.1.1_2	QC-1nDJLG-S93127156W|EPI_ISL_11370032|Canada|BA.1.1|2022-01-31	96,03
EPI_ISL_11371178	BA.2	A	A_14	NAQ-AG-2203020067|EPI_ISL_11371178|France|BA.2|2022-02-28	99,81
EPI_ISL_11371618	BA.2	BA.2	BA.2_1	NAQ-AG-2202160098|EPI_ISL_11371618|France|BA.2|2022-02-14	97,92
EPI_ISL_11371872	BA.1.17	BA.1	BA.1_3	NAQ-AG-2201260008|EPI_ISL_11371872|France|BA.1.17|2022-01-26	96,64
EPI_ISL_11372548	BA.2	BA.2	BA.2_1	BE-IFIK-220321_os_85|EPI_ISL_11372548|Switzerland|BA.2|2022-03-14	99,81
EPI_ISL_11376648	BA.2	A	A_17	PHEC-YYDCQH6|EPI_ISL_11376648|United Kingdom|BA.2|2022-03-10	99,29
EPI_ISL_11376841	BA.1.1	BA.1.1	BA.1.1_2	PHEC-YYDCWOO|EPI_ISL_11376841|United Kingdom|BA.1.1|2022-03-11	99,62
EPI_ISL_11376971	BA.2	A	A_4	PHEC-YYDM31J|EPI_ISL_11376971|United Kingdom|BA.2|2022-03-05	99,79
EPI_ISL_11378663	BA.2	BA.2.12	BA.2.12	RJ-SMS-ICMR-INSACOG-TS-13014|EPI_ISL_11378663|India|BA.2|2022-03-13	96,51
EPI_ISL_11378697	BA.2	BA.2.12	BA.2.12	RJ-SMS-ICMR-INSACOG-TS-13084|EPI_ISL_11378697|India|BA.2|2022-03-15	96,51
EPI_ISL_11379674	BA.2	BA.2	BA.2_1	RJ-SMS-ICMR-INSACOG-TS-3983|EPI_ISL_11379674|India|BA.2|2022-01-08	98,57
EPI_ISL_11381102	BA.2	BA.2	BA.2_1	DCGC-444457|EPI_ISL_11381102|Denmark|BA.2|2022-03-17	99,56
EPI_ISL_11381669	BA.2	A	A_17	DCGC-444920|EPI_ISL_11381669|Denmark|BA.2|2022-03-17	99,98
EPI_ISL_11384416	BA.1.1	BA.1.1	BA.1.1_2	BE-IFIK-7582-4644|EPI_ISL_11384416|Switzerland|BA.1.1|2022-03-21	99,60
EPI_ISL_11384776	BA.1.15	BA.1.15	BA.1.15	RJ-LVM63175|EPI_ISL_11384776|Brazil|BA.1.15|2022-01-19	78,76
EPI_ISL_11385306	BA.2	A	A_14	OCC-CHU-TLS-2206411297|EPI_ISL_11385306|France|BA.2|2022-03-05	70,50
EPI_ISL_11386298	BA.2.7	A	A_17	MA-Broad-CRSP_27SGVNLF64U2C7Z5|EPI_ISL_11386298|USA|BA.2.7|2022-03-15	99,81
EPI_ISL_11386567	BA.2	BA.2	BA.2_1	MA-Broad-CRSP_7DJ2HM3SNGTSJRLY|EPI_ISL_11386567|USA|BA.2|2022-03-15	96,26
EPI_ISL_11390467	BA.1.1	BA.1.1	BA.1.1_2	CO-CDPHE-2103006867|EPI_ISL_11390467|USA|BA.1.1|2022-01-07	99,64
EPI_ISL_11391049	BA.2	A	A_14	PLYM-3B56948|EPI_ISL_11391049|United Kingdom|BA.2|2022-03-17	99,81
EPI_ISL_11391490	BA.2.3	A	A_14	MILK-3B6088A|EPI_ISL_11391490|United Kingdom|BA.2.3|2022-03-19	99,81
EPI_ISL_11391903	BA.2	A	A_14	QEUH-3B68451|EPI_ISL_11391903|United Kingdom|BA.2|2022-03-18	99,81
EPI_ISL_11392133	BA.2	A	A_14	MILK-3B69924|EPI_ISL_11392133|United Kingdom|BA.2|2022-03-19	99,81
EPI_ISL_11393721	BA.2.9	A	A_17	MILK-3B41F02|EPI_ISL_11393721|United Kingdom|BA.2.9|2022-03-16	99,81
EPI_ISL_11393786	BA.2	BA.2	BA.2_1	MILK-3B42E22|EPI_ISL_11393786|United Kingdom|BA.2|2022-03-18	99,81
EPI_ISL_11395176	BA.2	A	A_14	MILK-3B468A8|EPI_ISL_11395176|United Kingdom|BA.2|2022-03-18	99,81
EPI_ISL_11395885	BA.2	A	A_17	LSPA-3B4AF45|EPI_ISL_11395885|United Kingdom|BA.2|2022-03-17	99,81
EPI_ISL_11396911	BA.2.16	A	A_4	MILK-3B5441C|EPI_ISL_11396911|United Kingdom|BA.2.16|2022-03-18	99,81
EPI_ISL_11397883	BA.1.1	BA.1.1	BA.1.1_3	NEWC-3B20859|EPI_ISL_11397883|United Kingdom|BA.1.1|2022-03-17	99,64
EPI_ISL_11398179	BA.2	BA.2	BA.2_1	QEUH-3B0F02B|EPI_ISL_11398179|United Kingdom|BA.2|2022-03-17	99,81
EPI_ISL_11399595	BA.2.9	A	A_17	QEUH-3B269C9|EPI_ISL_11399595|United Kingdom|BA.2.9|2022-03-17	99,81
EPI_ISL_11400276	BA.1.1	BA.1.1	BA.1.1_3	QEUH-3B12D88|EPI_ISL_11400276|United Kingdom|BA.1.1|2022-03-17	99,64
EPI_ISL_11404803	BA.1.1.18	BA.1.1	BA.1.1_2	NM-TG1276388|EPI_ISL_11404803|USA|BA.1.1.18|2022-02-23	99,62
EPI_ISL_11405136	BA.2.3.14	A	A_14	KDCA32502|EPI_ISL_11405136|South Korea|BA.2.3.14|2022-02-25	99,81
EPI_ISL_11408850	BA.2	BA.2	BA.2_1	FL-Risch-2231801594|EPI_ISL_11408850|Liechtenstein|BA.2|2022-02-28	90,52
EPI_ISL_11409799	BA.1.15	BA.1	BA.1_3	CA-SEARCH-118003|EPI_ISL_11409799|USA|BA.1.15|2022-01-07	93,72
EPI_ISL_11410189	BA.1.1	BA.1.1	BA.1.1_3	BCN-SEARCH-118468|EPI_ISL_11410189|Mexico|BA.1.1|2022-01-12	96,66
EPI_ISL_11411149	BA.1	BA.1	BA.1_2	CA-OC-FG-232228|EPI_ISL_11411149|USA|BA.1|2021-12-24	99,52
EPI_ISL_11411300	BA.1.1	BA.1.1	BA.1.1_2	CA-OC-FG-238017|EPI_ISL_11411300|USA|BA.1.1|2021-12-27	96,58
EPI_ISL_11413071	BA.1.1	BA.1.1	BA.1.1_3	CA-TCPHL-030222-02|EPI_ISL_11413071|USA|BA.1.1|2022-02-22	99,64
EPI_ISL_11413349	BA.1.1	BA.1.1	BA.1.1_2	NY-URMC-22034818-1|EPI_ISL_11413349|USA|BA.1.1|2022-03-12	96,03
EPI_ISL_11413805	BA.2.2	BA.2.2	BA.2.2	HKU-220326-071|EPI_ISL_11413805|Hong Kong|BA.2.2|2022-02-08	99,81
EPI_ISL_11414283	BA.1.1	BA.1.1	BA.1.1_2	MA-NEIDL-04900|EPI_ISL_11414283|USA|BA.1.1|2022-02-12	96,68
EPI_ISL_11416895	BA.2.9	A	A_17	PLYM-3B80D3A|EPI_ISL_11416895|United Kingdom|BA.2.9|2022-03-19	99,81
EPI_ISL_11418478	BA.2	BA.2	BA.2_1	LSPA-3B7B558|EPI_ISL_11418478|United Kingdom|BA.2|2022-03-19	99,81
EPI_ISL_11418999	BA.1.1	BA.1.1	BA.1.1_2	LSPA-3B82020|EPI_ISL_11418999|United Kingdom|BA.1.1|2022-03-20	99,64
EPI_ISL_11423206	BA.2	BA.2	BA.2_1	LSPA-3B6F0DE|EPI_ISL_11423206|United Kingdom|BA.2|2022-03-19	99,81
EPI_ISL_11423298	BA.2	A	A_14	LSPA-3B70D88|EPI_ISL_11423298|United Kingdom|BA.2|2022-03-18	99,81
EPI_ISL_11425101	BA.2.49	A	A_17	ARA-HCL722001365801|EPI_ISL_11425101|France|BA.2.49|2022-03-07	99,81
EPI_ISL_11425935	BA.2	A	A_14	ULB-IBC_CV8422929524|EPI_ISL_11425935|Belgium|BA.2|2022-03-20	99,79
EPI_ISL_11427414	BA.2	A	A_14	UT-UPHL-220324498088|EPI_ISL_11427414|USA|BA.2|2022-03-02	99,81
EPI_ISL_11428663	BA.2	A	A_14	LSPA-3BB76F3|EPI_ISL_11428663|United Kingdom|BA.2|2022-03-21	99,81
EPI_ISL_11428689	BA.2.9	BA.2	BA.2_1	LSPA-3BB7541|EPI_ISL_11428689|United Kingdom|BA.2.9|2022-03-21	99,81
EPI_ISL_11431440	BA.1.1.14	BA.1.1	BA.1.1_2	QEUH-3B9FB89|EPI_ISL_11431440|United Kingdom|BA.1.1.14|2022-03-19	99,64
EPI_ISL_11432615	BA.2.10	A	A_14	LSPA-3B925F1|EPI_ISL_11432615|United Kingdom|BA.2.10|2022-03-19	99,81
EPI_ISL_11432782	BA.2	BA.2	BA.2_1	BRBR-3B926FE|EPI_ISL_11432782|United Kingdom|BA.2|2022-03-19	99,81
EPI_ISL_11433618	BA.2	A	A_17	BRBR-3B9D295|EPI_ISL_11433618|United Kingdom|BA.2|2022-03-19	99,81
EPI_ISL_11433646	BA.2	BA.2	BA.2_1	LSPA-3B9CDB3|EPI_ISL_11433646|United Kingdom|BA.2|2022-03-19	99,81
EPI_ISL_11434707	BA.1.1	BA.1.1	BA.1.1_3	LSPA-3B97B63|EPI_ISL_11434707|United Kingdom|BA.1.1|2022-03-20	99,64
EPI_ISL_11435892	BA.1.1.18	BA.1.1	BA.1.1_3	CO-CDPHE-2103024564|EPI_ISL_11435892|USA|BA.1.1.18|2022-03-03	87,04
EPI_ISL_11436236	BA.1.15	BA.1	BA.1_4	CO-CDPHE-2102759519|EPI_ISL_11436236|USA|BA.1.15|2022-01-20	97,79
EPI_ISL_11443244	BA.2.9	A	A_17	DCGC-446128|EPI_ISL_11443244|Denmark|BA.2.9|2022-03-20	99,81
EPI_ISL_11443961	BA.2.14	A	A_17	DCGC-446792|EPI_ISL_11443961|Denmark|BA.2.14|2022-03-20	99,79
EPI_ISL_11444533	BA.2	A	A_14	DCGC-447363|EPI_ISL_11444533|Denmark|BA.2|2022-03-20	99,52
EPI_ISL_11448213	BA.2.10	A	A_14	LSPA-3BAE9E7|EPI_ISL_11448213|United Kingdom|BA.2.10|2022-03-21	99,81
EPI_ISL_11449685	BA.1	BA.1.15	BA.1.15	NIC_PKT_SEQ9511|EPI_ISL_11449685|Thailand|BA.1|2022-01-14	81,11
EPI_ISL_11451057	BA.2	A	A_14	NW-RKI-I-609217|EPI_ISL_11451057|Germany|BA.2|2022-03-05	99,81
EPI_ISL_11453923	BA.2	A	A_17	SH-RKI-I-612268|EPI_ISL_11453923|Germany|BA.2|2022-02-28	99,81
EPI_ISL_11454294	BA.2	BA.2	BA.2_1	NW-RKI-I-612875|EPI_ISL_11454294|Germany|BA.2|2022-03-08	99,81
EPI_ISL_11454501	BA.1.1	BA.1.1	BA.1.1_2	NW-RKI-I-613058|EPI_ISL_11454501|Germany|BA.1.1|2022-03-08	99,64
EPI_ISL_11455229	BA.2	A	A_17	SH-RKI-I-614473|EPI_ISL_11455229|Germany|BA.2|2022-03-05	96,89
EPI_ISL_11457178	BA.1.1.1	BA.1.1	BA.1.1_3	BW-RKI-I-615919|EPI_ISL_11457178|Germany|BA.1.1.1|2022-02-17	99,31
EPI_ISL_11464353	BA.2	A	A_14	BW-RKI-I-621529|EPI_ISL_11464353|Germany|BA.2|2022-03-05	99,81
EPI_ISL_11466500	BA.2	A	A_14	NORT-YNNQSGH|EPI_ISL_11466500|United Kingdom|BA.2|2022	99,81
EPI_ISL_11466534	BA.2.9	A	A_17	BY-RKI-I-623566|EPI_ISL_11466534|Germany|BA.2.9|2022-03-03	99,81
EPI_ISL_11468698	BA.1.1	BA.1.15	BA.1.15	NI-RKI-I-625655|EPI_ISL_11468698|Germany|BA.1.1|2022-03-04	75,69
EPI_ISL_11469294	BA.2	BA.2	BA.2_1	BE-RKI-I-626588|EPI_ISL_11469294|Germany|BA.2|2022-02-22	99,71
EPI_ISL_11469787	BA.1	BA.1.15	BA.1.15	PHEC-5X0A1Z4C|EPI_ISL_11469787|United Kingdom|BA.1|2022	98,17
EPI_ISL_11470877	BA.2	A	A_14	BW-RKI-I-628015|EPI_ISL_11470877|Germany|BA.2|2022-03-08	99,81
EPI_ISL_11471089	BA.1.18	BA.1	BA.1_4	NW-RKI-I-628162|EPI_ISL_11471089|Germany|BA.1.18|2022-03-01	99,52
EPI_ISL_11471205	BA.2	A	A_17	BY-RKI-I-628291|EPI_ISL_11471205|Germany|BA.2|2022-03-07	99,81
EPI_ISL_11472687	BA.2	BA.2	BA.2_1	BW-RKI-I-629153|EPI_ISL_11472687|Germany|BA.2|2022-03-09	95,17
EPI_ISL_11472773	BA.1.1	BA.1.1	BA.1.1_3	NW-RKI-I-629229|EPI_ISL_11472773|Germany|BA.1.1|2022-03-09	99,64

EPI_ISL_11473759	BA.1.21	BA.1.15	BA.1.15	NW-RKI-I-630030|EPI_ISL_11473759|Germany|BA.1.21|2022-03-05	99,64
EPI_ISL_11474811	BA.2.5	BA.2	BA.2_1	ST-RKI-I-631339|EPI_ISL_11474811|Germany|BA.2.5|2022-03-07	94,75
EPI_ISL_11475475	BA.2.9	A	A_17	BB-RKI-I-632324|EPI_ISL_11475475|Germany|BA.2.9|2022-03-10	93,26
EPI_ISL_11476123	BA.2	A	A_17	BB-RKI-I-633012|EPI_ISL_11476123|Germany|BA.2|2022-03-11	99,81
EPI_ISL_11477026	BA.2.3	BA.2	BA.2_1	NI-RKI-I-633876|EPI_ISL_11477026|Germany|BA.2.3|2022-03-08	95,21
EPI_ISL_11477192	BA.1.1	BA.1.1	BA.1.1_3	PHEC-YYDQ9HJ|EPI_ISL_11477192|United Kingdom|BA.1.1|2022-03-09	99,64
EPI_ISL_11478399	BA.2.9	A	A_17	NW-RKI-I-635051|EPI_ISL_11478399|Germany|BA.2.9|2022-03-15	99,81
EPI_ISL_11482040	BA.2.9	A	A_17	PHEC-YYDXDMG|EPI_ISL_11482040|United Kingdom|BA.2.9|2022-03-14	99,79
EPI_ISL_11482237	BA.2	A	A_14	BW-RKI-I-639059|EPI_ISL_11482237|Germany|BA.2|2022-02-14	98,51
EPI_ISL_11482892	BA.1	BA.1.15	BA.1.15	BW-RKI-I-639593|EPI_ISL_11482892|Germany|BA.1|2022-02-14	99,64
EPI_ISL_11483746	BA.2.9	A	A_17	BW-RKI-I-640301|EPI_ISL_11483746|Germany|BA.2.9|2022-02-14	98,51
EPI_ISL_11484329	BA.1.1	BA.1.1	BA.1.1_2	BW-RKI-I-640659|EPI_ISL_11484329|Germany|BA.1.1|2022-03-03	90,90
EPI_ISL_11484909	BA.1.1	BA.1.1	BA.1.1_2	TH-RKI-I-641121|EPI_ISL_11484909|Germany|BA.1.1|2022-02-03	99,62
EPI_ISL_11485229	BA.1.1	BA.1	BA.1_4	BY-RKI-I-641340|EPI_ISL_11485229|Germany|BA.1.1|2022-01-24	95,54
EPI_ISL_11487689	BA.1.1	BA.1.1	BA.1.1_2	WI-UW-9909|EPI_ISL_11487689|USA|BA.1.1|2022-03-11	99,64
EPI_ISL_11488480	BA.1.1.1	BA.1.1	BA.1.1_3	WI-CDC-ASC210734496|EPI_ISL_11488480|USA|BA.1.1.1|2022-03-11	99,64
EPI_ISL_11489007	BA.2.9	A	A_17	01_SE100_22CS503968|EPI_ISL_11489007|Sweden|BA.2.9|2022-03-16	99,79
EPI_ISL_11491941	BA.2	A	A_14	PHEP-YYRXI9K|EPI_ISL_11491941|United Kingdom|BA.2|2022-03-11	99,81
EPI_ISL_11493816	BA.2	BA.2	BA.2_1	PHWC-PJFCDB|EPI_ISL_11493816|United Kingdom|BA.2|2022-03-09	99,81
EPI_ISL_11494453	BA.1.1.1	BA.1.1	BA.1.1_3	PHWC-PJFPCQ|EPI_ISL_11494453|United Kingdom|BA.1.1.1|2022-03-08	99,64
EPI_ISL_11500412	BA.1.1	BA.1.1	BA.1.1_2	ON-PHL-22-13994|EPI_ISL_11500412|Canada|BA.1.1|2022-03-16	87,52
EPI_ISL_11509860	BA.2	A	A_14	HDF-CERBAHC-03651837|EPI_ISL_11509860|France|BA.2|2022-03-14	99,81
EPI_ISL_11515047	BA.2.3	A	A_14	7122067042|EPI_ISL_11515047|Brunei|BA.2.3|2022-03-05	95,99
EPI_ISL_11516646	BA.2.12	BA.2.12	BA.2.12	NORT-YNNX54C|EPI_ISL_11516646|United Kingdom|BA.2.12|2022	99,81
EPI_ISL_11518659	BA.2	A	A_17	DCGC-450649|EPI_ISL_11518659|Denmark|BA.2|2022-03-21	99,96
EPI_ISL_11519974	BA.2	A	A_17	DCGC-451765|EPI_ISL_11519974|Denmark|BA.2|2022-03-22	99,98
EPI_ISL_11520225	BA.2	BA.2.10	BA.2.10	DCGC-451941|EPI_ISL_11520225|Denmark|BA.2|2022-03-22	99,79
EPI_ISL_11520710	BA.2	BA.2	BA.2_1	PHEC-YYDTF9S|EPI_ISL_11520710|United Kingdom|BA.2|2022-03-14	99,79
EPI_ISL_11522478	BA.2.9	BA.2	BA.2_1	3295437164|EPI_ISL_11522478|Sweden|BA.2.9|2022-03-12	90,40
EPI_ISL_11528881	BA.2	BA.2	BA.2_1	VD-CHUV-GEN11158|EPI_ISL_11528881|Switzerland|BA.2|2022-03-16	99,81
EPI_ISL_11530494	BA.2.10.1	BA.2.10.1	BA.2.10.1	VA-CDC-LC0564223|EPI_ISL_11530494|USA|BA.2.10.1|2022-03-16	99,81
EPI_ISL_11531409	BA.1.1	BA.1.1	BA.1.1_2	UT-UPHL-220326827100|EPI_ISL_11531409|USA|BA.1.1|2022-01-22	99,64
EPI_ISL_11531437	BA.1.15	BA.1	BA.1_3	UT-UPHL-220326204908|EPI_ISL_11531437|USA|BA.1.15|2022-01-24	99,64
EPI_ISL_11532313	BA.2	BA.2	BA.2_1	AZDelta-2212-04343|EPI_ISL_11532313|Belgium|BA.2|2022-03-24	95,33
EPI_ISL_11533627	BA.1.17	BA.1.15	BA.1.15	VA_FBCH_2302|EPI_ISL_11533627|USA|BA.1.17|2022-01-20	99,64
EPI_ISL_11534691	BA.1.1	BA.1.1	BA.1.1_2	CA-SEARCH-80021|EPI_ISL_11534691|USA|BA.1.1|2022-01-06	99,43
EPI_ISL_11534905	BA.1.1	BA.1.1	BA.1.1_2	CA-SEARCH-80241|EPI_ISL_11534905|USA|BA.1.1|2022-01-08	99,48
EPI_ISL_11535201	BA.1.1	BA.1.1	BA.1.1_2	CA-SEARCH-80542|EPI_ISL_11535201|USA|BA.1.1|2022-01-08	99,48
EPI_ISL_11535216	BA.1.15	BA.1.15	BA.1.15	CA-SEARCH-80557|EPI_ISL_11535216|USA|BA.1.15|2022-01-08	99,48
EPI_ISL_11535989	BA.1.1.2	BA.1.1	BA.1.1_3	TKYkbm12681|EPI_ISL_11535989|Japan|BA.1.1.2|2022-03-05	99,64
EPI_ISL_11536004	BA.1.1.2	BA.1.1	BA.1.1_3	TKYkbm12696|EPI_ISL_11536004|Japan|BA.1.1.2|2022-03-05	99,64
EPI_ISL_11536182	BA.1.1.2	BA.1.1	BA.1.1_2	TKYkbm11995|EPI_ISL_11536182|Japan|BA.1.1.2|2022-03-01	99,64
EPI_ISL_11536201	BA.1.1	BA.1.1	BA.1.1_3	TKYkbm12014|EPI_ISL_11536201|Japan|BA.1.1|2022-03-01	99,64
EPI_ISL_11536335	BA.1.1.2	BA.1.1	BA.1.1_1	SZ-NIG-Y213493|EPI_ISL_11536335|Japan|BA.1.1.2|2022-02-09	99,64
EPI_ISL_11536577	BA.1.1.2	BA.1.1	BA.1.1_3	SZ-NIG-4-C4531|EPI_ISL_11536577|Japan|BA.1.1.2|2022-02-20	99,64
EPI_ISL_11537484	BA.1.18	BA.1.15	BA.1.15	CO-CDPHE-2103057666|EPI_ISL_11537484|USA|BA.1.18|2022-01-20	90,98
EPI_ISL_11537673	BA.1.1	BA.1.1	BA.1.1_2	MS-SRL_331298982|EPI_ISL_11537673|USA|BA.1.1|2022-01-03	99,64
EPI_ISL_11538374	BA.1	BA.1.15	BA.1.15	CO-CDPHE-2103061872|EPI_ISL_11538374|USA|BA.1|2022-01-16	70,39
EPI_ISL_11539108	BA.1	A	A_1	CO-CDPHE-2103047581|EPI_ISL_11539108|USA|BA.1|2022-03-11	28,01
EPI_ISL_11541992	BA.2.10.1	BA.2.10.1	BA.2.10.1	KA-SLS-SEQ-29174|EPI_ISL_11541992|India|BA.2.10.1|2022-01	97,77
EPI_ISL_11542403	BA.1	BA.1.1	BA.1.1_2	NICD-N30901|EPI_ISL_11542403|South Africa|BA.1|2022-01-27	74,17
EPI_ISL_11543693	BA.2	BA.2	BA.2_1	LSPA-3C05CC6|EPI_ISL_11543693|United Kingdom|BA.2|2022-03-22	99,81
EPI_ISL_11543732	BA.2	BA.2	BA.2_1	LSPA-3C06B22|EPI_ISL_11543732|United Kingdom|BA.2|2022-03-23	99,81
EPI_ISL_11543891	BA.2	A	A_14	LSPA-3C0FC26|EPI_ISL_11543891|United Kingdom|BA.2|2022-03-23	99,81
EPI_ISL_11545304	BA.1.1	BA.2	BA.2_1	LSPA-3C24D28|EPI_ISL_11545304|United Kingdom|BA.1.1|2022-03-24	99,08
EPI_ISL_11545801	BA.2.18	BA.2.18	BA.2.18	LSPA-3BFF7FC|EPI_ISL_11545801|United Kingdom|BA.2.18|2022-03-22	99,81
EPI_ISL_11546466	BA.1.1	BA.1.1	BA.1.1_3	151837|EPI_ISL_11546466|Slovenia|BA.1.1|2022-02-01	99,43
EPI_ISL_11549039	BA.2	A	A_17	DCGC-452300|EPI_ISL_11549039|Denmark|BA.2|2022-03-24	99,75
EPI_ISL_11551215	BA.2.1	BA.2	BA.2_1	LSPA-3C0B4AA|EPI_ISL_11551215|United Kingdom|BA.2.1|2022-03-22	99,81
EPI_ISL_11554402	BA.2	A	A_17	DCGC-453860|EPI_ISL_11554402|Denmark|BA.2|2022-03-24	99,81
EPI_ISL_11558183	BA.2	A	A_17	QEUH-3BA4DC7|EPI_ISL_11558183|United Kingdom|BA.2|2022-03-19	99,81
EPI_ISL_11558618	BA.2	A	A_14	PHEP-YYROAHK|EPI_ISL_11558618|United Kingdom|BA.2|2022-03-12	99,79
EPI_ISL_11558787	BA.2	A	A_14	PHEP-YYROJ9U|EPI_ISL_11558787|United Kingdom|BA.2|2022-03-14	99,81
EPI_ISL_11564472	BA.2	BA.2	BA.2_1	UMB-IZSGC-31858-1-55|EPI_ISL_11564472|Italy|BA.2|2022-03-21	99,81
EPI_ISL_11565153	BA.2.36	BA.2	BA.2_2	CHUNamur14095535|EPI_ISL_11565153|Belgium|BA.2.36|2022-03-23	99,81
EPI_ISL_11565938	BA.2.10	A	A_14	CA-CDPH-3000306064|EPI_ISL_11565938|USA|BA.2.10|2022-02-01	86,91
EPI_ISL_11565958	BA.1.1	BA.1.1	BA.1.1_2	CA-CDPH-3000330234|EPI_ISL_11565958|USA|BA.1.1|2022-02-02	78,29
EPI_ISL_11568234	BA.1	BA.1	BA.1_4	CA-CDPH-3000328520|EPI_ISL_11568234|USA|BA.1|2022-02-02	88,76
EPI_ISL_11570851	BA.2.7	A	A_17	FL-CDC-ASC210736140|EPI_ISL_11570851|USA|BA.2.7|2022-03-16	99,81
EPI_ISL_11571346	BA.1.1	BA.1.1	BA.1.1_2	IL-CDC-ASC210845636|EPI_ISL_11571346|USA|BA.1.1|2022-03-17	99,64
EPI_ISL_11571840	BA.1.1	BA.1.1	BA.1.1_2	CA-SEARCH-80853|EPI_ISL_11571840|USA|BA.1.1|2022-01-09	99,48
EPI_ISL_11572565	BA.1	BA.1	BA.1_4	CA-SEARCH-81519|EPI_ISL_11572565|USA|BA.1|2022-01-14	99,48
EPI_ISL_11573181	BA.1.1	BA.1.1	BA.1.1_2	CA-SEARCH-81880|EPI_ISL_11573181|USA|BA.1.1|2022-01-20	99,48
EPI_ISL_11576713	BA.1.15	BA.1.15	BA.1.15	VA-CAV_VAS3N_00007550_01|EPI_ISL_11576713|USA|BA.1.15|2022-01	74,83
EPI_ISL_11576721	BA.1.1	BA.1.1	BA.1.1_3	VA-CAV_VAS3N_00007560_01|EPI_ISL_11576721|USA|BA.1.1|2022-01	87,10
EPI_ISL_11577159	BA.1.15	BA.1.15	BA.1.15	VA-CAV_VAS3N_00009034_01|EPI_ISL_11577159|USA|BA.1.15|2022-02	87,24
EPI_ISL_11578354	BA.1.1	BA.1.1	BA.1.1_2	CA-SEARCH-83409|EPI_ISL_11578354|USA|BA.1.1|2022-01-22	99,48
EPI_ISL_11580963	BA.1.1	BA.1.1	BA.1.1_2	08-087792-MB|EPI_ISL_11580963|Slovenia|BA.1.1|2022-02-25	96,99
EPI_ISL_11581810	BA.1	B.1.623	B.1.623	LS-NVRL-ecS22IRL00089138|EPI_ISL_11581810|Ireland|BA.1|2022-01-26	64,05
EPI_ISL_11582584	BA.2.9	BA.2	BA.2_1	GES-HMN-22032140707|EPI_ISL_11582584|France|BA.2.9|2022-03-07	87,20
EPI_ISL_11582750	BA.1.1	BA.1	BA.1_4	HDF-HMN-22032160214|EPI_ISL_11582750|France|BA.1.1|2022-03-07	95,59
EPI_ISL_11583231	BA.2.3.2	BA.2	BA.2_1	BMH-310322-sample11|EPI_ISL_11583231|Vietnam|BA.2.3.2|2022-03-21	95,17
EPI_ISL_11583392	BA.1.15	BA.2	BA.2_1	LA-TUSOM-220329_S09|EPI_ISL_11583392|USA|BA.1.15|2022-03-22	80,94
EPI_ISL_11583417	BA.2	A	A_14	CHUNamur14098749|EPI_ISL_11583417|Belgium|BA.2|2022-03-25	98,74
EPI_ISL_11585232	BA.2.9	BA.2	BA.2_1	EDB50147|EPI_ISL_11585232|United Kingdom|BA.2.9|2022-03-07	94,09
EPI_ISL_11588498	BA.1.1	BA.1.1	BA.1.1_2	166435|EPI_ISL_11588498|Slovenia|BA.1.1|2022-02-18	99,64
EPI_ISL_11588547	BA.1.1	BA.1.1	BA.1.1_3	166633|EPI_ISL_11588547|Slovenia|BA.1.1|2022-02-18	99,64
EPI_ISL_11588716	BA.2	BA.2.12	BA.2.12	RJ-SMS-ICMR-INSACOG-TS-2467|EPI_ISL_11588716|India|BA.2|2022-01-03	96,51
EPI_ISL_11589291	BA.2	BA.2	BA.2_1	BE-RKI-I-643388|EPI_ISL_11589291|Germany|BA.2|2022-03-03	95,15
EPI_ISL_11589475	BA.2	A	A_17	SH-RKI-I-643535|EPI_ISL_11589475|Germany|BA.2|2022-02-22	99,81
EPI_ISL_11591517	BA.2	BA.2	BA.2_1	LSPA-3C48386|EPI_ISL_11591517|United Kingdom|BA.2|2022-03-24	99,81
EPI_ISL_11592245	BA.1.1	BA.1.1	BA.1.1_2	NW-RKI-I-644341|EPI_ISL_11592245|Germany|BA.1.1|2022-03-11	99,56
EPI_ISL_11595262	BA.1.1	BA.1.1	BA.1.1_2	159092|EPI_ISL_11595262|Slovenia|BA.1.1|2022-02-08	99,64
EPI_ISL_11595531	BA.2	BA.2	BA.2_1	LSPA-3C37F86|EPI_ISL_11595531|United Kingdom|BA.2|2022-03-24	99,81
EPI_ISL_11596530	BA.2.18	BA.2.18	BA.2.18	QEUH-3C19E6F|EPI_ISL_11596530|United Kingdom|BA.2.18|2022-03-22	99,81
EPI_ISL_11597172	BA.1.1	BA.1.1	BA.1.1_2	BW-RKI-I-647900|EPI_ISL_11597172|Germany|BA.1.1|2022-02-21	99,64
EPI_ISL_11599366	BA.2.9	A	A_17	HH-RKI-I-648855|EPI_ISL_11599366|Germany|BA.2.9|2022-03-11	99,58
EPI_ISL_11600418	BA.2	BA.2	BA.2_1	NW-RKI-I-649271|EPI_ISL_11600418|Germany|BA.2|2022-03-03	95,12
EPI_ISL_11600634	BA.2.23	A	A_14	QEUH-3C29CF9|EPI_ISL_11600634|United Kingdom|BA.2.23|2022-03-23	99,81
EPI_ISL_11601235	BA.2	BA.2	BA.2_1	BY-RKI-I-649553|EPI_ISL_11601235|Germany|BA.2|2022-01-27	93,68
EPI_ISL_11601589	BA.2.1	BA.2	BA.2_1	LSPA-3C2B83F|EPI_ISL_11601589|United Kingdom|BA.2.1|2022-03-24	99,81

EPI_ISL_11602421	XR	A	A_14	PHWC-PJ88RY|EPI_ISL_11602421|United Kingdom|XR|2022-03-06	99,81
EPI_ISL_11603148	BA.2	BA.2	BA.2_1	QEUH-3C022D6|EPI_ISL_11603148|United Kingdom|BA.2|2022-03-22	99,81
EPI_ISL_11605555	BA.2	A	A_14	PHWC-PJE8U9|EPI_ISL_11605555|United Kingdom|BA.2|2022-03-07	99,81
EPI_ISL_11606005	BA.2	A	A_14	BW-RKI-I-650873|EPI_ISL_11606005|Germany|BA.2|2022-02-24	98,51
EPI_ISL_11609899	BA.2.9	A	A_17	HH-RKI-I-652182|EPI_ISL_11609899|Germany|BA.2.9|2022-03-13	99,29
EPI_ISL_11610101	BA.2	A	A_14	PHWC-PJJQUS|EPI_ISL_11610101|United Kingdom|BA.2|2022-03-11	99,81
EPI_ISL_11610327	BA.1.1.18	BA.1.1	BA.1.1_3	FL-BPHL-4411|EPI_ISL_11610327|USA|BA.1.1.18|2022-01-31	99,64
EPI_ISL_11610484	BA.2	BA.2	BA.2_1	NW-RKI-I-652701|EPI_ISL_11610484|Germany|BA.2|2022-03-02	99,73
EPI_ISL_11612846	BA.2	A	A_17	SH-RKI-I-655238|EPI_ISL_11612846|Germany|BA.2|2022-03-07	99,81
EPI_ISL_11614032	BA.2	A	A_17	SH-RKI-I-656006|EPI_ISL_11614032|Germany|BA.2|2022-03-04	99,81
EPI_ISL_11614601	BA.2	BA.2	BA.2_1	NW-RKI-I-656561|EPI_ISL_11614601|Germany|BA.2|2022-03-14	95,33
EPI_ISL_11615022	BA.1.1	BA.1.1	BA.1.1_1	NW-RKI-I-656930|EPI_ISL_11615022|Germany|BA.1.1|2022-03-07	99,48
EPI_ISL_11617482	BA.1.1	BA.1.1	BA.1.1_2	BY-RKI-I-660689|EPI_ISL_11617482|Germany|BA.1.1|2022-03-09	99,58
EPI_ISL_11617715	BA.2.5	BA.2	BA.2_1	SN-RKI-I-660938|EPI_ISL_11617715|Germany|BA.2.5|2022-03-08	99,71
EPI_ISL_11618571	BA.2	BA.2	BA.2_1	NI-RKI-I-662035|EPI_ISL_11618571|Germany|BA.2|2022-03-09	99,81
EPI_ISL_11619333	BA.2	A	A_14	BY-RKI-I-662698|EPI_ISL_11619333|Germany|BA.2|2022-03-10	99,81
EPI_ISL_11623446	BA.1.1.14	BA.1.1	BA.1.1_2	NS-NML-382300|EPI_ISL_11623446|Canada|BA.1.1.14|2022-01-05	79,62
EPI_ISL_11625435	BA.1.1.2	BA.1.1	BA.1.1_2	RIMD06819|EPI_ISL_11625435|Japan|BA.1.1.2|2022-01-25	99,64
EPI_ISL_11626773	BA.1.15	BA.1.15	BA.1.15	CO-CDPHE-2103033215|EPI_ISL_11626773|USA|BA.1.15|2021-12-22	99,64
EPI_ISL_11627826	BA.1.1	BA.1.1	BA.1.1_2	AMA-INS-15723|EPI_ISL_11627826|Peru|BA.1.1|2022-02-23	99,64
EPI_ISL_11628717	BA.1.1	BA.1.1	BA.1.1_2	NV-CDC-QDX34992974|EPI_ISL_11628717|USA|BA.1.1|2022-03-19	99,64
EPI_ISL_11629031	BA.2.3	A	A_14	NM-NMDOH-2022024372|EPI_ISL_11629031|USA|BA.2.3|2022-01-26	99,81
EPI_ISL_11629034	BA.1.1	BA.1.1	BA.1.1_2	NM-NMDOH-2022025779|EPI_ISL_11629034|USA|BA.1.1|2022-01-27	99,64
EPI_ISL_11630263	BA.2	A	A_14	VIC47003|EPI_ISL_11630263|Australia|BA.2|2022-03-13	99,39
EPI_ISL_11630469	BA.2.9	BA.2	BA.2_1	VIC47210|EPI_ISL_11630469|Australia|BA.2.9|2022-03-15	99,81
EPI_ISL_11630818	BA.2	A	A_14	VIC47405|EPI_ISL_11630818|Australia|BA.2|2022-03-17	99,81
EPI_ISL_11632739	BA.1.17	BA.1.15	BA.1.15	QLD0x00D825|EPI_ISL_11632739|Australia|BA.1.17|2022-03-05	99,64
EPI_ISL_11633467	BA.2	A	A_4	PAC-CERBAHC-03808189|EPI_ISL_11633467|France|BA.2|2022-03-21	99,81
EPI_ISL_11634741	BA.2.3.17	A	A_14	WA-PHL-017027|EPI_ISL_11634741|USA|BA.2.3.17|2022-03-18	96,20
EPI_ISL_11638955	XQ	A	A_14	LSPA-3C5821D|EPI_ISL_11638955|United Kingdom|XQ|2022-03-24	99,81
EPI_ISL_11638981	BA.2	A	A_17	BW-RKI-I-665908|EPI_ISL_11638981|Germany|BA.2|2022-03-14	99,07
EPI_ISL_11639180	BA.2	A	A_17	BW-RKI-I-665975|EPI_ISL_11639180|Germany|BA.2|2022-03-21	99,07
EPI_ISL_11639270	BA.1.1	BA.1.1	BA.1.1_3	LSPA-3C6B50F|EPI_ISL_11639270|United Kingdom|BA.1.1|2022-03-24	99,64
EPI_ISL_11643949	BA.2	BA.2	BA.2_1	DCGC-455423|EPI_ISL_11643949|Denmark|BA.2|2022-03-25	99,98
EPI_ISL_11644242	BA.2.9	A	A_17	ALDP-3BE6A3B|EPI_ISL_11644242|United Kingdom|BA.2.9|2022-03-21	99,81
EPI_ISL_11648193	BA.2	A	A_17	DCGC-456439|EPI_ISL_11648193|Denmark|BA.2|2022-03-27	99,81
EPI_ISL_11648520	BA.2	A	A_14	QEUH-3C4719C|EPI_ISL_11648520|United Kingdom|BA.2|2022-03-25	99,81
EPI_ISL_11650438	BA.2.3	BA.2	BA.2_1	NEWC-3C57D86|EPI_ISL_11650438|United Kingdom|BA.2.3|2022-03-23	99,81
EPI_ISL_11651144	BA.2	A	A_17	DCGC-457124|EPI_ISL_11651144|Denmark|BA.2|2022-03-23	99,81
EPI_ISL_11652457	BA.2	BA.2	BA.2_1	DCGC-457322|EPI_ISL_11652457|Denmark|BA.2|2022-03-26	99,73
EPI_ISL_11652984	BA.2	A	A_17	BW-RKI-I-670484|EPI_ISL_11652984|Germany|BA.2|2022-03-06	98,51
EPI_ISL_11653163	BA.1.1	BA.1.1	BA.1.1_2	QEUH-3C29E6C|EPI_ISL_11653163|United Kingdom|BA.1.1|2022-03-24	99,62
EPI_ISL_11653228	BA.1.1	BA.1	BA.1_4	RP-RKI-I-670568|EPI_ISL_11653228|Germany|BA.1.1|2022-03-16	90,63
EPI_ISL_11653661	BA.2.36	BA.2	BA.2_1	DCGC-457608|EPI_ISL_11653661|Denmark|BA.2.36|2022-03-27	99,79
EPI_ISL_11654188	BA.2	A	A_17	DCGC-457736|EPI_ISL_11654188|Denmark|BA.2|2022-03-27	99,79
EPI_ISL_11654782	BA.2	BA.2	BA.2_1	QEUH-3C19D71|EPI_ISL_11654782|United Kingdom|BA.2|2022-03-23	99,81
EPI_ISL_11654837	BA.2	BA.2	BA.2_1	BY-RKI-I-671071|EPI_ISL_11654837|Germany|BA.2|2022-03-17	99,77
EPI_ISL_11655933	BA.2	A	A_4	PHEC-YYDU7HY|EPI_ISL_11655933|United Kingdom|BA.2|2022-03-16	99,79
EPI_ISL_11657886	BA.1	BA.1.15	BA.1.15	PHEC-YYDUQO8|EPI_ISL_11657886|United Kingdom|BA.1|2022-03-18	99,58
EPI_ISL_11660594	BA.2.3	BA.2	BA.2_1	FL-RIVM-94311|EPI_ISL_11660594|Netherlands|BA.2.3|2022-03-16	99,81
EPI_ISL_11661809	BA.2	BA.2	BA.2_1	SN-RKI-I-675793|EPI_ISL_11661809|Germany|BA.2|2022-03-21	90,54
EPI_ISL_11663356	BA.2	A	A_17	NW-RKI-I-677738|EPI_ISL_11663356|Germany|BA.2|2022-03-25	90,52
EPI_ISL_11665025	BA.2	BA.2	BA.2_1	BY-RKI-I-679224|EPI_ISL_11665025|Germany|BA.2|2022-03-13	99,81
EPI_ISL_11665114	BA.2	BA.2	BA.2_1	BY-RKI-I-679317|EPI_ISL_11665114|Germany|BA.2|2022-03-15	97,42
EPI_ISL_11665567	BA.1.1	BA.1.1	BA.1.1_2	KDCA33259|EPI_ISL_11665567|South Korea|BA.1.1|2022-03-04	99,64
EPI_ISL_11665615	BA.2	A	A_14	KDCA33306|EPI_ISL_11665615|South Korea|BA.2|2022-03-04	99,81
EPI_ISL_11668189	BA.2.9	BA.2	BA.2_1	SN-RKI-I-682263|EPI_ISL_11668189|Germany|BA.2.9|2022-03-15	99,73
EPI_ISL_11670749	BA.2	A	A_17	MV-RKI-I-683754|EPI_ISL_11670749|Germany|BA.2|2022-03-16	99,81
EPI_ISL_11671246	BA.2	BA.2	BA.2_1	NW-RKI-I-684323|EPI_ISL_11671246|Germany|BA.2|2022-03-11	99,79
EPI_ISL_11674902	BA.2.9	A	A_17	NI-RKI-I-686732|EPI_ISL_11674902|Germany|BA.2.9|2022-03-21	99,81
EPI_ISL_11675162	BA.1.1	BA.1	BA.1_2	NI-RKI-I-686955|EPI_ISL_11675162|Germany|BA.1.1|2022-03-16	99,20
EPI_ISL_11675624	BA.2.12	BA.2.12	BA.2.12	SH-RKI-I-687310|EPI_ISL_11675624|Germany|BA.2.12|2022-03-07	99,81
EPI_ISL_11679476	BA.1	BA.1	BA.1_4	8734361-GMI|EPI_ISL_11679476|Belize|BA.1|2021-12-28	99,81
EPI_ISL_11679569	BA.2	A	A_14	27SVAN38051|EPI_ISL_11679569|Czech Republic|BA.2|2022-03-15	96,70
EPI_ISL_11679971	BA.2.36	BA.2	BA.2_2	ULG-25512|EPI_ISL_11679971|Belgium|BA.2.36|2022-03-22	97,52
EPI_ISL_11680465	BA.1	BA.1.15	BA.1.15	NY-CUIMC-NP-9781|EPI_ISL_11680465|USA|BA.1|2022-01-10	99,58
EPI_ISL_11682789	BA.2.10	BA.2.10	BA.2.10	WA-Curative-261014|EPI_ISL_11682789|USA|BA.2.10|2022-03-18	93,84
EPI_ISL_11684116	BA.1	BA.1	BA.1_2	CA-HLX-STM-JGPJ4ZSU8|EPI_ISL_11684116|USA|BA.1|2021-12-24	89,28
EPI_ISL_11686834	BA.1.1	BA.1.1	BA.1.1_2	UT-UPHL-220331597071|EPI_ISL_11686834|USA|BA.1.1|2022-01-22	99,64
EPI_ISL_11687510	BA.1.1	BA.1.1	BA.1.1_2	UT-UPHL-220331049508|EPI_ISL_11687510|USA|BA.1.1|2022-01-26	99,64
EPI_ISL_11687527	BA.1.1	BA.1.1	BA.1.1_2	UT-UPHL-220331964846|EPI_ISL_11687527|USA|BA.1.1|2022-03-03	99,64
EPI_ISL_11688398	BA.2	BA.2	BA.2_1	BRBR-3C7A488|EPI_ISL_11688398|United Kingdom|BA.2|2022-03-25	99,81
EPI_ISL_11688731	BA.2.9	BA.2	BA.2_1	LSPA-3C78FD4|EPI_ISL_11688731|United Kingdom|BA.2.9|2022-03-25	99,81
EPI_ISL_11689163	BA.2.10.1	BA.2.10.1	BA.2.10.1	LSPA-3C91C65|EPI_ISL_11689163|United Kingdom|BA.2.10.1|2022-03-26	94,09
EPI_ISL_11692655	BA.2	A	A_14	QEUH-3C620F8|EPI_ISL_11692655|United Kingdom|BA.2|2022-03-26	99,81
EPI_ISL_11694791	BA.2	A	A_17	LSPA-3C2E33B|EPI_ISL_11694791|United Kingdom|BA.2|2022-03-23	99,81
EPI_ISL_11694890	BA.2	BA.2	BA.2_1	LSPA-3C47A91|EPI_ISL_11694890|United Kingdom|BA.2|2022-03-25	99,81
EPI_ISL_11696571	BA.2	A	A_14	NJ-CDC-LC0566243|EPI_ISL_11696571|USA|BA.2|2022-03-21	99,81
EPI_ISL_11697176	BA.2	A	A_17	CA-CDC-QDX35038438|EPI_ISL_11697176|USA|BA.2|2022-03-16	99,81
EPI_ISL_11702838	BA.2	A	A_14	LSPA-3C99122|EPI_ISL_11702838|United Kingdom|BA.2|2022-03-26	99,81
EPI_ISL_11703070	BA.2	A	A_14	QEUH-3C9632F|EPI_ISL_11703070|United Kingdom|BA.2|2022-03-27	99,81
EPI_ISL_11703088	BA.2	A	A_14	LSPA-3C97D5D|EPI_ISL_11703088|United Kingdom|BA.2|2022-03-27	99,81
EPI_ISL_11703390	BA.2.9	A	A_17	LSPA-3C8AF93|EPI_ISL_11703390|United Kingdom|BA.2.9|2022-03-27	99,81
EPI_ISL_11703618	XE	A	A_14	LSPA-3C8A045|EPI_ISL_11703618|United Kingdom|XE|2022-03-24	99,81
EPI_ISL_11703842	BA.2	BA.2.10	BA.2.10	NEWC-3C4F0F1|EPI_ISL_11703842|United Kingdom|BA.2|2022-03-25	99,81
EPI_ISL_11707105	BA.1.1.2	BA.1.1	BA.1.1_3	PG-206264|EPI_ISL_11707105|Japan|BA.1.1.2|2022-01-27	99,64
EPI_ISL_11707333	BA.1.1.2	BA.1.1	BA.1.1_3	PG-206492|EPI_ISL_11707333|Japan|BA.1.1.2|2022-02-02	99,64
EPI_ISL_11707892	BA.1.1.2	BA.1.1	BA.1.1_2	PG-208635|EPI_ISL_11707892|Japan|BA.1.1.2|2022-01-28	99,64
EPI_ISL_11711063	BA.1.1.2	BA.1.1	BA.1.1_3	PG-215576|EPI_ISL_11711063|Japan|BA.1.1.2|2022	99,64
EPI_ISL_11712245	BA.1.1.2	BA.1.1	BA.1.1_2	PG-200069|EPI_ISL_11712245|Japan|BA.1.1.2|2022-02-15	99,64
EPI_ISL_11712938	BC.1	BC.1	BC.1	PG-207932|EPI_ISL_11712938|Japan|BC.1|2022-02-23	99,64
EPI_ISL_11713870	BA.1.1.2	BA.1.1	BA.1.1_2	PG-212256|EPI_ISL_11713870|Japan|BA.1.1.2|2022-03-09	99,64
EPI_ISL_11713887	BA.2.3.13	A	A_14	PG-212273|EPI_ISL_11713887|Japan|BA.2.3.13|2022-03-10	99,81
EPI_ISL_11713947	BA.1.1.2	BA.1.1	BA.1.1_3	PG-215476|EPI_ISL_11713947|Japan|BA.1.1.2|2022-03-16	99,64
EPI_ISL_11714087	BA.1.1.2	BA.1.1	BA.1.1_3	PG-214330|EPI_ISL_11714087|Japan|BA.1.1.2|2022-03-06	99,64
EPI_ISL_11714334	BA.1.1.2	BA.1.1	BA.1.1_1	PG-209970|EPI_ISL_11714334|Japan|BA.1.1.2|2022-02-28	99,64
EPI_ISL_11714469	BA.1.1	BA.1.1	BA.1.1_2	PG-214367|EPI_ISL_11714469|Japan|BA.1.1|2022-03-07	99,64
EPI_ISL_11714623	BA.1.1.2	BA.1.1	BA.1.1_2	PG-207638|EPI_ISL_11714623|Japan|BA.1.1.2|2022-02-12	99,64
EPI_ISL_11715199	BA.1.1	BA.1.1	BA.1.1_3	PG-193159|EPI_ISL_11715199|Japan|BA.1.1|2022-02-06	99,16
EPI_ISL_11715705	BA.1.1.2	BA.1.1	BA.1.1_3	PG-201905|EPI_ISL_11715705|Japan|BA.1.1.2|2022-02-10	99,64
EPI_ISL_11716068	BA.1.1.2	BA.1.1	BA.1.1_3	PG-207398|EPI_ISL_11716068|Japan|BA.1.1.2|2022-02-17	99,64

EPI_ISL_11716334	BA.1.1	BA.1	BA.1_4	PG-210566|EPI_ISL_11716334|Japan|BA.1.1|2022-02-24	99,64
EPI_ISL_11716425	BA.1.1.2	BA.1.1	BA.1.1_2	PG-210657|EPI_ISL_11716425|Japan|BA.1.1.2|2022-03-06	99,64
EPI_ISL_11716991	BA.1.1.2	BA.1.1	BA.1.1_2	PG-208225|EPI_ISL_11716991|Japan|BA.1.1.2|2022-02-08	99,64
EPI_ISL_11721461	BA.2.9	A	A_17	DCGC-458525|EPI_ISL_11721461|Denmark|BA.2.9|2022-03-28	99,81
EPI_ISL_11721909	BA.2	BA.2	BA.2_1	DCGC-458823|EPI_ISL_11721909|Denmark|BA.2|2022-03-27	99,98
EPI_ISL_11721994	BA.1.1.2	BA.1.1	BA.1.1_2	PG-210067|EPI_ISL_11721994|Japan|BA.1.1.2|2022-03-08	99,64
EPI_ISL_11722281	BA.2	BA.2	BA.2_1	DCGC-459069|EPI_ISL_11722281|Denmark|BA.2|2022-03-26	99,39
EPI_ISL_11722420	BA.2.14	BA.2.14	BA.2.14	DCGC-459156|EPI_ISL_11722420|Denmark|BA.2.14|2022-03-28	99,79
EPI_ISL_11723018	BA.2.9	BA.2	BA.2_1	DCGC-459532|EPI_ISL_11723018|Denmark|BA.2.9|2022-03-25	94,22
EPI_ISL_11724204	BA.2	A	A_17	DCGC-460302|EPI_ISL_11724204|Denmark|BA.2|2022-03-28	99,81
EPI_ISL_11724549	BA.1.1	BA.1.1	BA.1.1_2	PG-196018|EPI_ISL_11724549|Japan|BA.1.1|2022-02-06	99,64
EPI_ISL_11726995	BA.1.1	BA.1.1	BA.1.1_2	LSPA-3CCDC15|EPI_ISL_11726995|United Kingdom|BA.1.1|2022-03-28	99,64
EPI_ISL_11727000	BA.2.37	A	A_14	LSPA-3CCDB27|EPI_ISL_11727000|United Kingdom|BA.2.37|2022-03-28	99,81
EPI_ISL_11727458	BA.2	BA.2	BA.2_1	IDF-SLS-762203071479|EPI_ISL_11727458|France|BA.2|2022-03-22	91,32
EPI_ISL_11728774	BA.2	BA.2	BA.2_1	LSPA-3CC0887|EPI_ISL_11728774|United Kingdom|BA.2|2022-03-26	99,81
EPI_ISL_11729399	BA.2.1	A	A_14	LSPA-3CBCF6B|EPI_ISL_11729399|United Kingdom|BA.2.1|2022-03-28	99,81
EPI_ISL_11730430	BA.2	A	A_14	LSPA-3CC4100|EPI_ISL_11730430|United Kingdom|BA.2|2022-03-27	99,81
EPI_ISL_11731544	BA.2	A	A_17	LSPA-3CB07AB|EPI_ISL_11731544|United Kingdom|BA.2|2022-03-28	99,81
EPI_ISL_11733089	BA.2.1	A	A_4	PT31071|EPI_ISL_11733089|Portugal|BA.2.1|2022-03-22	99,81
EPI_ISL_11733760	BA.1.1	BA.1.1	BA.1.1_3	22BG-EU_016308_PI152|EPI_ISL_11733760|Bulgaria|BA.1.1|2022-01-21	99,64
EPI_ISL_11734232	BA.1.1	BA.1.1	BA.1.1_3	22BG-EU_016822_PI158|EPI_ISL_11734232|Bulgaria|BA.1.1|2022-01-26	99,64
EPI_ISL_11735152	BA.2	A	A_1	KA-RFNB-6671|EPI_ISL_11735152|India|BA.2|2022-02	66,69
EPI_ISL_11736938	BA.2	BA.2	BA.2_1	RC0633721|EPI_ISL_11736938|Estonia|BA.2|2022-03-11	98,51
EPI_ISL_11739216	BA.2	BA.2	BA.2_1	ARCH-002983C7|EPI_ISL_11739216|United Kingdom|BA.2|2022	99,81
EPI_ISL_11740110	BA.2	BA.2	BA.2_1	EDB51418|EPI_ISL_11740110|United Kingdom|BA.2|2022-03-07	94,09
EPI_ISL_11740330	BA.1.1.18	BA.1.1	BA.1.1_3	ND-NDDH-13370|EPI_ISL_11740330|USA|BA.1.1.18|2022-02-01	96,09
EPI_ISL_11741186	BA.2	BA.2	BA.2_1	NORT-YNN1YP5|EPI_ISL_11741186|United Kingdom|BA.2|2022	99,81
EPI_ISL_11741285	BA.2.3	A	A_14	NORT-YNNO6DI|EPI_ISL_11741285|United Kingdom|BA.2.3|2022	99,81
EPI_ISL_11743151	BA.2	A	A_17	BS-UHB-43419641|EPI_ISL_11743151|Switzerland|BA.2|2022-03-23	99,79
EPI_ISL_11744375	BA.1.1	BA.1.1	BA.1.1_3	PHEC-YYDIDHS|EPI_ISL_11744375|United Kingdom|BA.1.1|2022-03-10	93,23
EPI_ISL_11745593	BA.2	A	A_17	PHEC-YYDIOAT|EPI_ISL_11745593|United Kingdom|BA.2|2022-03-19	89,66
EPI_ISL_11746102	BA.2.9	A	A_17	PHEC-YYDSGJA|EPI_ISL_11746102|United Kingdom|BA.2.9|2022-03-19	99,81
EPI_ISL_11746344	BA.2	BA.2	BA.2_1	PHEC-YYDSQW3|EPI_ISL_11746344|United Kingdom|BA.2|2022-03-16	97,65
EPI_ISL_11747568	BA.1.1	BA.1.1	BA.1.1_2	NY-PRL-2022_0329_00E24|EPI_ISL_11747568|USA|BA.1.1|2022-03-25	99,64
EPI_ISL_11747724	BA.1.1	BA.1.1	BA.1.1_2	PHEC-YYDZAN1|EPI_ISL_11747724|United Kingdom|BA.1.1|2022-03-21	99,64
EPI_ISL_11748128	BA.2	A	A_4	PHEC-YYDZRYK|EPI_ISL_11748128|United Kingdom|BA.2|2022-03-21	99,77
EPI_ISL_11749111	BA.2	A	A_14	PHEP-YYRT9IX|EPI_ISL_11749111|United Kingdom|BA.2|2022-03-19	99,81
EPI_ISL_11749821	BA.1.1.15	BA.1.1	BA.1.1_2	QLD0x00D908|EPI_ISL_11749821|Australia|BA.1.1.15|2022-03-04	99,64
EPI_ISL_11750512	BA.2	BA.2	BA.2_1	PHWC-PJC5T8|EPI_ISL_11750512|United Kingdom|BA.2|2022-03-14	99,62
EPI_ISL_11750840	BA.2	A	A_14	PHWC-PJCJSX|EPI_ISL_11750840|United Kingdom|BA.2|2022-03-15	99,81
EPI_ISL_11750955	BA.2	A	A_14	PHWC-PJCP65|EPI_ISL_11750955|United Kingdom|BA.2|2022-03-16	99,81
EPI_ISL_11752867	BA.1.18	BA.1.15	BA.1.15	PHWC-PJM6WY|EPI_ISL_11752867|United Kingdom|BA.1.18|2022-03-14	99,64
EPI_ISL_11753932	BA.2	BA.2	BA.2_1	SCOT-7134|EPI_ISL_11753932|United Kingdom|BA.2|2022-03-08	99,79
EPI_ISL_11754063	BA.2.9	BA.2	BA.2_1	SCOT-7356|EPI_ISL_11754063|United Kingdom|BA.2.9|2022-03-09	94,71
EPI_ISL_11754563	BA.2.9.5	BA.2	BA.2_1	NIC_LRI_12044|EPI_ISL_11754563|Thailand|BA.2.9.5|2022-03	93,68
EPI_ISL_11754716	BA.1.1	BA.1.1	BA.1.1_3	A454|EPI_ISL_11754716|Malaysia|BA.1.1|2021-12-30	99,64
EPI_ISL_11756176	BA.1.1	BA.1.1	BA.1.1_3	GA-CDC-STM-RUXU48BFT|EPI_ISL_11756176|USA|BA.1.1|2022-03-19	99,64
EPI_ISL_11760501	BA.2	BA.2	BA.2_1	VD-CHUV-GEN11262|EPI_ISL_11760501|Switzerland|BA.2|2022-03-21	99,81
EPI_ISL_11760638	BA.1.1	BA.1.1	BA.1.1_2	NIC-INSPI-112676|EPI_ISL_11760638|Ecuador|BA.1.1|2022-03-11	99,64
EPI_ISL_11762060	BA.2	A	A_14	ON-PHL-22-15080|EPI_ISL_11762060|Canada|BA.2|2022-03-23	94,41
EPI_ISL_11762467	BA.1.1	BA.1.1	BA.1.1_2	ON-PHL-22-15505|EPI_ISL_11762467|Canada|BA.1.1|2022-03-21	90,96
EPI_ISL_11762516	BA.2	A	A_14	ON-PHL-22-15557|EPI_ISL_11762516|Canada|BA.2|2022-03-25	91,15
EPI_ISL_11764175	BA.2	A	A_14	TX-HMH-MCoV-96224|EPI_ISL_11764175|USA|BA.2|2022-03-23	99,81
EPI_ISL_11764782	BA.2	BA.2	BA.2_1	NW-HHU-22886|EPI_ISL_11764782|Germany|BA.2|2022-03-19	94,26
EPI_ISL_11765840	BA.2.10	BA.2.10	BA.2.10	CO-CDPHE-2103058293|EPI_ISL_11765840|USA|BA.2.10|2022-03-14	99,79
EPI_ISL_11767056	BA.2.3	A	A_14	9222098652|EPI_ISL_11767056|Brunei|BA.2.3|2022-02-23	95,97
EPI_ISL_11768961	BA.1.1	BA.1.1	BA.1.1_3	LSPA-3CE9633|EPI_ISL_11768961|United Kingdom|BA.1.1|2022-03-27	99,64
EPI_ISL_11769950	BA.2	BA.2	BA.2_1	LSPA-3CD90CF|EPI_ISL_11769950|United Kingdom|BA.2|2022-03-28	99,81
EPI_ISL_11771253	BA.2	BA.2	BA.2_1	QEUH-3CD5403|EPI_ISL_11771253|United Kingdom|BA.2|2022-03-28	99,81
EPI_ISL_11773698	BA.2	BA.2	BA.2_1	QEUH-3CBA361|EPI_ISL_11773698|United Kingdom|BA.2|2022-03-28	99,81
EPI_ISL_11775143	BA.2	A	A_14	QEUH-3C7DB44|EPI_ISL_11775143|United Kingdom|BA.2|2022-03-25	99,81
EPI_ISL_11775867	BA.2.23	A	A_14	QEUH-3C9DD2A|EPI_ISL_11775867|United Kingdom|BA.2.23|2022-03-27	99,81
EPI_ISL_11778930	BA.1.1	BA.1.1	BA.1.1_3	OR-OHSU-16515|EPI_ISL_11778930|USA|BA.1.1|2022-01-07	99,64
EPI_ISL_11780290	BA.2	BA.2	BA.2_1	SIC-3422024092_S64|EPI_ISL_11780290|Italy|BA.2|2022-03-28	90,56
EPI_ISL_11781425	BA.1.1	BA.1.1	BA.1.1_3	DR-GD-02242007|EPI_ISL_11781425|Netherlands|BA.1.1|2022-02-11	79,30
EPI_ISL_11781520	BA.2	BA.2	BA.2_1	NORT-YNN19KN|EPI_ISL_11781520|United Kingdom|BA.2|2022	99,81
EPI_ISL_11782012	BA.2	BA.2	BA.2_1	NORT-YNN1TGA|EPI_ISL_11782012|United Kingdom|BA.2|2022-03-16	91,17
EPI_ISL_11784806	BA.2.9	A	A_17	DCGC-462342|EPI_ISL_11784806|Denmark|BA.2.9|2022-03-22	99,96
EPI_ISL_11784958	BA.2.3	A	A_14	DCGC-462420|EPI_ISL_11784958|Denmark|BA.2.3|2022-03-30	99,81
EPI_ISL_11785214	BA.2	A	A_17	DCGC-462672|EPI_ISL_11785214|Denmark|BA.2|2022-03-28	99,77
EPI_ISL_11786819	BA.2	A	A_14	DCGC-464076|EPI_ISL_11786819|Denmark|BA.2|2022-03-22	99,81
EPI_ISL_11786993	BA.2	BA.2	BA.2_1	DCGC-464246|EPI_ISL_11786993|Denmark|BA.2|2022-03-22	99,79
EPI_ISL_11787832	BA.2.9	BA.2	BA.2_1	DCGC-464786|EPI_ISL_11787832|Denmark|BA.2.9|2022-03-30	97,94
EPI_ISL_11789624	BA.2	BA.2	BA.2_1	CT-HUJT-RB32202|EPI_ISL_11789624|Spain|BA.2|2022-03-23	95,82
EPI_ISL_11789733	BA.2	BA.2	BA.2_1	Jessa_11-2213-005164|EPI_ISL_11789733|Belgium|BA.2|2022-03-31	98,19
EPI_ISL_11790674	BA.2	BA.2	BA.2_1	MI-CDC-LC0568116|EPI_ISL_11790674|USA|BA.2|2022-03-21	99,81
EPI_ISL_11791431	BA.2	BA.2	BA.2_1	NY-CDC-LC0567753|EPI_ISL_11791431|USA|BA.2|2022-03-24	99,81
EPI_ISL_11793125	BA.2	BA.2	BA.2_1	BW-FR3182|EPI_ISL_11793125|Germany|BA.2|2022-03-09	95,23
EPI_ISL_11798318	BA.1.1	BA.1.1	BA.1.1_2	CA-UCSF-JD1887|EPI_ISL_11798318|USA|BA.1.1|2022-02-28	99,62
EPI_ISL_11798891	BA.2	A	A_4	BA_22_00019982|EPI_ISL_11798891|Slovakia|BA.2|2022-03-17	99,81
EPI_ISL_11799234	BA.1	BA.1.15	BA.1.15	LAZ-AMC-2203182637-DS|EPI_ISL_11799234|Italy|BA.1|2022-03-18	92,08
EPI_ISL_11800281	BA.2.10.1	BA.2.10.1	BA.2.10.1	WA2598|EPI_ISL_11800281|Australia|BA.2.10.1|2022-03-28	99,37
EPI_ISL_11800923	BA.2	A	A_14	QEUH-3CF29A7|EPI_ISL_11800923|United Kingdom|BA.2|2022-03-29	99,81
EPI_ISL_11802104	BA.2	BA.2.2	BA.2.2	HSLL-3BE445D|EPI_ISL_11802104|United Kingdom|BA.2|2022-03-22	99,81
EPI_ISL_11802179	BA.2.9	A	A_17	HSLL-3BE4639|EPI_ISL_11802179|United Kingdom|BA.2.9|2022-03-22	99,81
EPI_ISL_11805031	BA.1.1	BA.1.1	BA.1.1_2	UNIMAS-HBTU555|EPI_ISL_11805031|Malaysia|BA.1.1|2022-03-12	99,62
EPI_ISL_11805668	BA.1.1.14	BA.1	BA.1_1	KA-SLS-SEQ-17010|EPI_ISL_11805668|India|BA.1.1.14|2021-12-24	85,65
EPI_ISL_11806600	BA.2	A	A_14	KE-NVRL-S22IRL00238919|EPI_ISL_11806600|Ireland|BA.2|2022-03-15	90,52
EPI_ISL_11807204	BA.1	BA.1.15	BA.1.15	CVR14783|EPI_ISL_11807204|United Kingdom|BA.1|2022-01-03	99,56
EPI_ISL_11807624	BA.2	A	A_14	CVR15008|EPI_ISL_11807624|United Kingdom|BA.2|2022-02-23	99,73
EPI_ISL_11808298	BA.2	A	A_17	DCGC-467586|EPI_ISL_11808298|Denmark|BA.2|2022-04-01	99,81
EPI_ISL_11809110	BA.2	A	A_14	NORT-YNNWP19|EPI_ISL_11809110|United Kingdom|BA.2|2022	99,81
EPI_ISL_11809160	BA.2	BA.2	BA.2_1	NORT-YNNWQYD|EPI_ISL_11809160|United Kingdom|BA.2|2022	99,81
EPI_ISL_11809962	BA.2.3	BA.2	BA.2_1	PHEC-YYDAHKH|EPI_ISL_11809962|United Kingdom|BA.2.3|2022-03-15	87,27
EPI_ISL_11810749	BA.2	BA.2	BA.2_1	PHEP-YYRWYUC|EPI_ISL_11810749|United Kingdom|BA.2|2022-03-20	99,77
EPI_ISL_11812223	BA.1.1	BA.1.1	BA.1.1_2	CT-Yale-18387|EPI_ISL_11812223|USA|BA.1.1|2022-03-19	99,64
EPI_ISL_11813212	BA.1.1	BA.1.1	BA.1.1_3	THL-202209087|EPI_ISL_11813212|Finland|BA.1.1|2022-03-03	99,92
EPI_ISL_11813718	BA.2.3	A	A_14	16941|EPI_ISL_11813718|Greece|BA.2.3|2022-03-17	99,69
EPI_ISL_11814484	BA.2	A	A_14	RI-RISHL-D08794|EPI_ISL_11814484|USA|BA.2|2022-03-15	99,81
EPI_ISL_11816392	BA.1.1	BA.1.1	BA.1.1_2	VA-VTVAS3-GSC30649|EPI_ISL_11816392|USA|BA.1.1|2022-01-12	99,64
EPI_ISL_11816401	BA.1	BA.1.15	BA.1.15	VA-VTVAS3-GSC30658|EPI_ISL_11816401|USA|BA.1|2022-01-12	99,64

EPI_ISL_11818017	BA.2	BA.2	BA.2_1	NW-RKI-I-690558|EPI_ISL_11818017|Germany|BA.2|2022-03-21	99,05
EPI_ISL_11818193	BA.1	BA.1.15	BA.1.15	NW-RKI-I-690740|EPI_ISL_11818193|Germany|BA.1|2022-03-21	99,64
EPI_ISL_11818999	BA.2	BA.2	BA.2_1	NW-RKI-I-691560|EPI_ISL_11818999|Germany|BA.2|2022-03-21	95,15
EPI_ISL_11820402	BA.2	A	A_17	HE-RKI-I-693422|EPI_ISL_11820402|Germany|BA.2|2022-03-22	99,77
EPI_ISL_11820870	BA.2	A	A_14	HH-RKI-I-693935|EPI_ISL_11820870|Germany|BA.2|2022-03-27	99,75
EPI_ISL_11822423	BA.1.1.1	BA.1.1	BA.1.1_3	ST-RKI-I-696102|EPI_ISL_11822423|Germany|BA.1.1.1|2022-03-02	99,64
EPI_ISL_11824460	BA.2	A	A_17	CVL-HCL722001557601|EPI_ISL_11824460|France|BA.2|2022-03-14	98,28
EPI_ISL_11824879	BA.2	BA.2	BA.2_1	NAQ-HCL722001593701|EPI_ISL_11824879|France|BA.2|2022-03-21	99,81
EPI_ISL_11826299	BA.1.1	BA.1.1	BA.1.1_2	NLE_IBT_IMSS_6842|EPI_ISL_11826299|Mexico|BA.1.1|2022-03-10	98,91
EPI_ISL_11827635	BA.2	A	A_14	CO-NVRL-S22IRL00239363|EPI_ISL_11827635|Ireland|BA.2|2022-03-15	90,52
EPI_ISL_11827989	BA.2	A	A_14	ARCH-002975F3|EPI_ISL_11827989|United Kingdom|BA.2|2022	99,81
EPI_ISL_11829075	BA.2	A	A_4	PHEC-YYD3FOR|EPI_ISL_11829075|United Kingdom|BA.2|2022-03-21	98,63
EPI_ISL_11830197	BA.2	BA.2	BA.2_1	AZDelta-2212-17018|EPI_ISL_11830197|Belgium|BA.2|2022-03-29	98,02
EPI_ISL_11830209	BA.2	BA.2	BA.2_1	AZDelta-2212-17165|EPI_ISL_11830209|Belgium|BA.2|2022-03-29	98,11
EPI_ISL_11830215	BA.2	BA.2.12	BA.2.12	AZDelta-2212-17174|EPI_ISL_11830215|Belgium|BA.2|2022-03-29	93,49
EPI_ISL_11830416	BA.2	BA.2	BA.2_1	AZDelta-2213-00740|EPI_ISL_11830416|Belgium|BA.2|2022-03-30	98,23
EPI_ISL_11830735	BA.2	A	A_17	AZDelta-2213-11623|EPI_ISL_11830735|Belgium|BA.2|2022-04-04	98,74
EPI_ISL_11833595	BA.2.9	A	A_17	DCGC-468863|EPI_ISL_11833595|Denmark|BA.2.9|2022-04-02	99,81
EPI_ISL_11833719	BA.2	A	A_17	DCGC-468988|EPI_ISL_11833719|Denmark|BA.2|2022-04-02	99,81
EPI_ISL_11834027	BA.1.1	BA.1.1	BA.1.1_2	AZ-ASU58383|EPI_ISL_11834027|USA|BA.1.1|2022-01-12	90,98
EPI_ISL_11834049	BA.1.1	BA.1.1	BA.1.1_2	AZ-ASU58410|EPI_ISL_11834049|USA|BA.1.1|2022-01-12	96,01
EPI_ISL_11834363	BA.1.21	BA.1	BA.1_1	TAS001284|EPI_ISL_11834363|Australia|BA.1.21|2022-03-10	99,64
EPI_ISL_11834433	BA.2.3	A	A_14	TAS001357|EPI_ISL_11834433|Australia|BA.2.3|2022-03-22	99,81
EPI_ISL_11835853	BA.2	A	A_17	AG_507895|EPI_ISL_11835853|Romania|BA.2|2022-02-22	99,81
EPI_ISL_11837949	BA.1.1	BA.1.1	BA.1.1_3	PHWC-PJXSAA|EPI_ISL_11837949|United Kingdom|BA.1.1|2022-03-15	98,91
EPI_ISL_11838731	BA.2	A	A_14	UZA-UA-3816908480|EPI_ISL_11838731|Belgium|BA.2|2022-03-17	99,45
EPI_ISL_11838935	BA.1.15	BA.1.15	BA.1.15	CA-CDPH-500050544|EPI_ISL_11838935|USA|BA.1.15|2022-01-29	78,11
EPI_ISL_11839317	BA.1.1	BA.1.15	BA.1.15	CA-CDPH-500054557|EPI_ISL_11839317|USA|BA.1.1|2022-02-28	79,30
EPI_ISL_11842711	BA.1.1	BA.1.1	BA.1.1_2	PA-CDC-STM-PZKY39MZE|EPI_ISL_11842711|USA|BA.1.1|2022-03-22	99,35
EPI_ISL_11843619	BA.2.18	BA.2.18	BA.2.18	FL-CDC-STM-4AYEUY5N7|EPI_ISL_11843619|USA|BA.2.18|2022-03-27	99,81
EPI_ISL_11845729	BA.2	BA.1.15	BA.1.15	22-014266|EPI_ISL_11845729|Sweden|BA.2|2022-03-10	91,93
EPI_ISL_11846503	BA.2	A	A_14	DA10614012|EPI_ISL_11846503|Sweden|BA.2|2022-03-14	92,16
EPI_ISL_11850698	BA.2	BA.2	BA.2_1	ICH-741208221|EPI_ISL_11850698|Israel|BA.2|2022-03-19	99,81
EPI_ISL_11852665	BA.2	BA.2	BA.2_1	461847870157|EPI_ISL_11852665|Sweden|BA.2|2022-01-11	99,81
EPI_ISL_11853413	BA.2	A	A_17	461847853660|EPI_ISL_11853413|Sweden|BA.2|2022-02-07	99,81
EPI_ISL_11855388	BA.2	A	A_14	ICH-741207608|EPI_ISL_11855388|Israel|BA.2|2022-03-13	99,81
EPI_ISL_11857113	BA.1.1	BA.1.1	BA.1.1_2	461847894317|EPI_ISL_11857113|Sweden|BA.1.1|2022-01-10	99,64
EPI_ISL_11857131	BA.1.17	BA.1.15	BA.1.15	461847793872|EPI_ISL_11857131|Sweden|BA.1.17|2022-01-13	99,64
EPI_ISL_11858478	BA.2	BA.2	BA.2_1	461848004294|EPI_ISL_11858478|Sweden|BA.2|2022-02-04	94,70
EPI_ISL_11859921	BA.2	BA.2	BA.2_1	ICH-741211417|EPI_ISL_11859921|Israel|BA.2|2022-03-23	98,55
EPI_ISL_11860537	BA.2.3	BA.2	BA.2_1	ICH-741209789|EPI_ISL_11860537|Israel|BA.2.3|2022-03-19	99,81
EPI_ISL_11860793	BA.2.3	A	A_4	ICH-741212624|EPI_ISL_11860793|Israel|BA.2.3|2022-03-27	99,52
EPI_ISL_11864763	BA.2.9	A	A_17	MEX-INMEGEN-65-30|EPI_ISL_11864763|Mexico|BA.2.9|2022-03-29	99,81
EPI_ISL_11866362	BA.2	BA.2	BA.2_1	IL-33016|EPI_ISL_11866362|USA|BA.2|2022-03	98,05
EPI_ISL_11868849	BA.2	A	A_4	HDF-CERBAHC-03786491|EPI_ISL_11868849|France|BA.2|2022-03-21	99,81
EPI_ISL_11869895	BA.2	BA.2	BA.2_1	MA-Broad-CRSP_HV5FM5HN3E652FSW|EPI_ISL_11869895|USA|BA.2|2022-03-23	96,72
EPI_ISL_11871154	BA.2.27	A	A_14	DMSc-09514|EPI_ISL_11871154|Thailand|BA.2.27|2022-03-10	96,93
EPI_ISL_11873230	BA.2	A	A_17	DCGC-469504|EPI_ISL_11873230|Denmark|BA.2|2022-03-31	99,98
EPI_ISL_11876954	BA.2.47	BA.2.12	BA.2.12	DCGC-470650|EPI_ISL_11876954|Denmark|BA.2.47|2022-03-31	97,48
EPI_ISL_11878210	BA.2	A	A_17	DCGC-471271|EPI_ISL_11878210|Denmark|BA.2|2022-03-31	99,77
EPI_ISL_11879292	BA.1	BA.1	BA.1_3	PHEC-YYDHBTY|EPI_ISL_11879292|United Kingdom|BA.1|2022-03-19	99,64
EPI_ISL_11880244	XG	BA.2	BA.2_1	DCGC-472102|EPI_ISL_11880244|Denmark|XG|2022-04-03	86,91
EPI_ISL_11880713	BA.2	BA.2	BA.2_1	PHEP-YYRWCOG|EPI_ISL_11880713|United Kingdom|BA.2|2022-03-14	99,77
EPI_ISL_11883665	BA.2	A	A_14	TN_22_00001061|EPI_ISL_11883665|Slovakia|BA.2|2022-03-31	99,81
EPI_ISL_11883676	BA.2.67	A	A_4	TN_22_00001068|EPI_ISL_11883676|Slovakia|BA.2.67|2022-04-01	99,81
EPI_ISL_11889443	BA.1.15	BA.1.15	BA.1.15	FL-BPHL-4657|EPI_ISL_11889443|USA|BA.1.15|2022-01-27	99,64
EPI_ISL_11890624	BA.1.1	BA.1.1	BA.1.1_2	MI-UM-10043536696|EPI_ISL_11890624|USA|BA.1.1|2022-01-11	99,64
EPI_ISL_11890940	BA.1	BA.1.15	BA.1.15	UT-UPHL-220407790264|EPI_ISL_11890940|USA|BA.1|2022-01-08	99,64
EPI_ISL_11892408	BA.2	BA.2	BA.2_1	NAQ-CERBAHC-03916681|EPI_ISL_11892408|France|BA.2|2022-03-28	99,81
EPI_ISL_11893583	BA.1.1	BA.1.1	BA.1.1_2	CA-SC-266206|EPI_ISL_11893583|USA|BA.1.1|2022-02-24	94,43
EPI_ISL_11893713	BA.1.17	BA.1	BA.1_4	CA-SC-266460|EPI_ISL_11893713|USA|BA.1.17|2022-02-06	96,05
EPI_ISL_11894838	BA.2	A	A_17	NAQ-IPP23163|EPI_ISL_11894838|France|BA.2|2022-03-21	99,81
EPI_ISL_11895207	BA.1.15	BA.1	BA.1_4	BA-LACEN-BA1395-292202222|EPI_ISL_11895207|Brazil|BA.1.15|2022-01-29	99,31
EPI_ISL_11899798	BA.2.10	BA.2	BA.2_1	CT-YPL22-25561|EPI_ISL_11899798|USA|BA.2.10|2022-03-29	99,31
EPI_ISL_11903144	BA.2.27	A	A_14	CONI-2646|EPI_ISL_11903144|Thailand|BA.2.27|2022-03-28	99,81
EPI_ISL_11903310	BA.2	A	A_14	CT-Broad-CRSP_QZG7Y725ZH6J7FST|EPI_ISL_11903310|USA|BA.2|2022-03-29	99,81
EPI_ISL_11903769	BA.2	BA.2	BA.2_1	MA-Broad-CRSP_D6E5P2T6GHLMVBLM|EPI_ISL_11903769|USA|BA.2|2022-03-30	99,81
EPI_ISL_11907459	BA.1.1	BA.1.1	BA.1.1_3	ME-HETL-J14062|EPI_ISL_11907459|USA|BA.1.1|2022-01-13	99,64
EPI_ISL_11907560	BA.1.1	BA.1	BA.1_4	ME-HETL-J14295|EPI_ISL_11907560|USA|BA.1.1|2022-02-15	84,51
EPI_ISL_11908178	BA.1.1	BA.1	BA.1_4	CA-CDPH-500051890|EPI_ISL_11908178|USA|BA.1.1|2022-02-07	76,57
EPI_ISL_11909341	BA.1	BA.1	BA.1_4	CA-CDPH-3000322395|EPI_ISL_11909341|USA|BA.1|2022-01-31	92,33
EPI_ISL_11909364	BA.1	BA.1	BA.1_4	CA-CDPH-3000322489|EPI_ISL_11909364|USA|BA.1|2022-01-31	92,12
EPI_ISL_11910798	BA.1.1.2	BA.1	BA.1_4	CA-CDPH-3000325884|EPI_ISL_11910798|USA|BA.1.1.2|2022-02-01	89,87
EPI_ISL_11911737	BA.1.1	BA.1	BA.1_4	CA-CDPH-3000325535|EPI_ISL_11911737|USA|BA.1.1|2022-02-02	87,56
EPI_ISL_11912423	BA.1.1	BA.1.1	BA.1.1_3	CA-CDPH-3000322866|EPI_ISL_11912423|USA|BA.1.1|2022-01-31	79,11
EPI_ISL_11914156	BA.2	BA.2	BA.2_1	DCGC-473399|EPI_ISL_11914156|Denmark|BA.2|2022-04-04	99,81
EPI_ISL_11915837	BA.1.1	BA.1	BA.1_4	EMR-IZSLER-2022-098097-013-01|EPI_ISL_11915837|Italy|BA.1.1|2022-03-27	93,70
EPI_ISL_11915965	BA.2	BA.2	BA.2_1	ARA-HCL022054615601|EPI_ISL_11915965|France|BA.2|2022-03-24	99,81
EPI_ISL_11916019	BA.2	BA.2	BA.2_1	ARA-HCL022055230401|EPI_ISL_11916019|France|BA.2|2022-03-26	99,81
EPI_ISL_11917170	BA.2.9	A	A_17	LSPA-3D1F84C|EPI_ISL_11917170|United Kingdom|BA.2.9|2022-04-02	99,81
EPI_ISL_11917687	BA.2	BA.2	BA.2_1	QEUH-3D14337|EPI_ISL_11917687|United Kingdom|BA.2|2022-04-04	89,62
EPI_ISL_11917907	BA.2	A	A_17	LSPA-3D0E8AA|EPI_ISL_11917907|United Kingdom|BA.2|2022-04-02	99,81
EPI_ISL_11917918	BA.2	A	A_14	LSPA-3D0E52B|EPI_ISL_11917918|United Kingdom|BA.2|2022-04-02	99,81
EPI_ISL_11918871	BA.2	A	A_14	LSPA-3D016CB|EPI_ISL_11918871|United Kingdom|BA.2|2022-03-30	99,81
EPI_ISL_11923960	BA.1.1	BA.1.1	BA.1.1_3	ARCH-002999F7|EPI_ISL_11923960|United Kingdom|BA.1.1|2022	99,64
EPI_ISL_11924963	BA.2	A	A_14	CVR15363|EPI_ISL_11924963|United Kingdom|BA.2|2022-03-22	99,73
EPI_ISL_11926353	BA.1.1	BA.1	BA.1_1	DHSC-CYBB47H|EPI_ISL_11926353|United Kingdom|BA.1.1|2022-01-23	95,17
EPI_ISL_11926546	BA.1.1	BA.1	BA.1_1	DHSC-CYBC13S|EPI_ISL_11926546|United Kingdom|BA.1.1|2022-01-14	95,02
EPI_ISL_11931264	BA.1.1.14	BA.2	BA.2_1	DHSC-CYBNHM7|EPI_ISL_11931264|United Kingdom|BA.1.1.14|2022-02-16	95,19
EPI_ISL_11933379	BA.2	A	A_14	DHSC-CYBZMJ8|EPI_ISL_11933379|United Kingdom|BA.2|2022-02-20	99,81
EPI_ISL_11933446	BA.2	BA.2	BA.2_1	DHSC-CYD1AWM|EPI_ISL_11933446|United Kingdom|BA.2|2022-02-24	99,81
EPI_ISL_11935100	BA.1	BA.1	BA.1_1	DHSC-CYD733K|EPI_ISL_11935100|United Kingdom|BA.1|2022-02-13	95,15
EPI_ISL_11935662	BA.1.17.2	BA.1.17.2	BA.1.17.2	DHSC-CYDASDR|EPI_ISL_11935662|United Kingdom|BA.1.17.2|2022-01-11	98,87
EPI_ISL_11938870	BA.1.17.2	BA.1.17.2	BA.1.17.2	DHSC-CYDMJK6|EPI_ISL_11938870|United Kingdom|BA.1.17.2|2022-02-04	95,15
EPI_ISL_11939846	BA.1.1	BA.1	BA.1_1	DHSC-CYDQ9N1|EPI_ISL_11939846|United Kingdom|BA.1.1|2022-02-09	96,57
EPI_ISL_11941845	BA.2	A	A_14	PT31655|EPI_ISL_11941845|Portugal|BA.2|2022-03-27	99,81
EPI_ISL_11942909	BA.1.17.2	BA.1.17.2	BA.1.17.2	DHSC-CYDY4MB|EPI_ISL_11942909|United Kingdom|BA.1.17.2|2022-01-03	95,19
EPI_ISL_11945716	BA.1	BA.2	BA.2_1	DHSC-CYNE1TP|EPI_ISL_11945716|United Kingdom|BA.1|2022-01-26	95,19
EPI_ISL_11946834	BA.1.1	BA.1	BA.1_1	DHSC-CYNJU6O|EPI_ISL_11946834|United Kingdom|BA.1.1|2022-02-15	95,94
EPI_ISL_11946904	BA.1.1.14	BA.1	BA.1_1	DHSC-CYNKBQB|EPI_ISL_11946904|United Kingdom|BA.1.1.14|2022-02-06	95,76
EPI_ISL_11946956	BA.1.1	BA.1	BA.1_1	DHSC-CYNKKUP|EPI_ISL_11946956|United Kingdom|BA.1.1|2022-02-01	95,17

EPI_ISL_11947087	BA.1	BA.1.1	BA.1.1_2	DHSC-CYNNGPQ|EPI_ISL_11947087|United Kingdom|BA.1|2022-01-13	94,83
EPI_ISL_11947151	BA.1	BA.1	BA.1_1	DHSC-CYNNYU8|EPI_ISL_11947151|United Kingdom|BA.1|2022-01-11	95,12
EPI_ISL_11947878	BA.1.1	BA.1	BA.1_1	DHSC-CYNSSCZ|EPI_ISL_11947878|United Kingdom|BA.1.1|2022-02-01	95,38
EPI_ISL_11947915	BD.1	BD.1	BD.1	DHSC-CYNSYAW|EPI_ISL_11947915|United Kingdom|BD.1|2022-02-11	95,19
EPI_ISL_11949223	BA.1.1	BA.1	BA.1_1	DHSC-CYY3W89|EPI_ISL_11949223|United Kingdom|BA.1.1|2022-01-19	95,17
EPI_ISL_11949232	BA.1	BA.1	BA.1_1	DHSC-CYY3XDC|EPI_ISL_11949232|United Kingdom|BA.1|2022-01-05	85,12
EPI_ISL_11949868	BA.1.1	BA.1	BA.1_1	DHSC-CYY7YKF|EPI_ISL_11949868|United Kingdom|BA.1.1|2022-01-23	95,59
EPI_ISL_11952911	BA.1.1	BA.1	BA.1_1	DHSC-CYYIK5E|EPI_ISL_11952911|United Kingdom|BA.1.1|2022-01-04	95,17
EPI_ISL_11953493	BA.1.1	BA.1	BA.1_1	DHSC-CYYKGSP|EPI_ISL_11953493|United Kingdom|BA.1.1|2022-02-01	94,16
EPI_ISL_11953870	BA.2	A	A_17	01_SE100_22CS504435|EPI_ISL_11953870|Sweden|BA.2|2022-04-01	95,69
EPI_ISL_11957359	BA.2	A	A_14	PHEC-YYD6BC8|EPI_ISL_11957359|United Kingdom|BA.2|2022-03-24	92,86
EPI_ISL_11958050	BA.2	BA.2	BA.2_1	PHEC-YYD7CUX|EPI_ISL_11958050|United Kingdom|BA.2|2022-03-25	95,29
EPI_ISL_11958258	BA.2	BA.2	BA.2_1	PHEC-YYD7P1O|EPI_ISL_11958258|United Kingdom|BA.2|2022-03-20	99,79
EPI_ISL_11958800	BA.2	BA.2	BA.2_1	PHEC-YYD9FTS|EPI_ISL_11958800|United Kingdom|BA.2|2022-03-24	92,58
EPI_ISL_11959217	BA.2	A	A_4	PHEC-YYD9UKZ|EPI_ISL_11959217|United Kingdom|BA.2|2022-03-25	99,79
EPI_ISL_11960029	BA.2.3	A	A_14	PHEP-YYRINXT|EPI_ISL_11960029|United Kingdom|BA.2.3|2022	96,32
EPI_ISL_11960601	BA.2.3.15	BA.2.12	BA.2.12	CA-Curative-257404|EPI_ISL_11960601|USA|BA.2.3.15|2022-03-26	83,46
EPI_ISL_11962099	BA.2	A	A_17	BW-RKI-I-698293|EPI_ISL_11962099|Germany|BA.2|2022-03-23	99,07
EPI_ISL_11964086	BA.2	A	A_14	BW-RKI-I-700236|EPI_ISL_11964086|Germany|BA.2|2022-03-28	99,81
EPI_ISL_11964523	BA.1.1	BA.1.1	BA.1.1_2	QC-L00454514001|EPI_ISL_11964523|Canada|BA.1.1|2022-03-21	99,64
EPI_ISL_11964786	BA.1.1	BA.1.1	BA.1.1_3	QC-L00454519001|EPI_ISL_11964786|Canada|BA.1.1|2022-03-22	99,64
EPI_ISL_11965161	BA.1.1.1	BA.1.1	BA.1.1_3	BW-RKI-I-701002|EPI_ISL_11965161|Germany|BA.1.1.1|2022-03-29	99,64
EPI_ISL_11965540	BA.1.17.2	BA.1.17.2	BA.1.17.2	QC-L00456541001|EPI_ISL_11965540|Canada|BA.1.17.2|2022-03-28	99,64
EPI_ISL_11965719	BA.2	BA.2	BA.2_1	BW-RKI-I-701359|EPI_ISL_11965719|Germany|BA.2|2022-03-26	99,07
EPI_ISL_11965781	BA.2	BA.2	BA.2_1	NI-RKI-I-701431|EPI_ISL_11965781|Germany|BA.2|2022-03-15	95,13
EPI_ISL_11968684	BA.2	BA.2	BA.2_1	PHWC-PJ1SOB|EPI_ISL_11968684|United Kingdom|BA.2|2022-03-21	99,81
EPI_ISL_11969725	BA.2	BA.2	BA.2_1	ICH-741211928|EPI_ISL_11969725|Israel|BA.2|2022-03-24	98,42
EPI_ISL_11970167	BA.1.1	BA.1.1	BA.1.1_2	ICH-741204737|EPI_ISL_11970167|Israel|BA.1.1|2022-03-08	91,91
EPI_ISL_11970253	BA.2	A	A_14	ICH-741205095|EPI_ISL_11970253|Israel|BA.2|2022-03-09	92,04
EPI_ISL_11970564	BA.1.1	BA.1.1	BA.1.1_2	RS-LACENRS-431673097|EPI_ISL_11970564|Brazil|BA.1.1|2022-02-08	90,73
EPI_ISL_11972842	BA.1.15	BA.1.15	BA.1.15	CA-CDPH-6000008614|EPI_ISL_11972842|USA|BA.1.15|2021-12-27	89,20
EPI_ISL_11972855	BA.1.15.2	BA.1.15.2	BA.1.15.2	CA-CDPH-6000008629|EPI_ISL_11972855|USA|BA.1.15.2|2021-12-27	99,62
EPI_ISL_11973439	BA.1.1	BA.1.1	BA.1.1_2	CA-CDPH-2000065504|EPI_ISL_11973439|USA|BA.1.1|2022-03-09	99,64
EPI_ISL_11973470	BA.1.1	BA.1.1	BA.1.1_2	CA-CDPH-2000065539|EPI_ISL_11973470|USA|BA.1.1|2022-03-08	99,64
EPI_ISL_11979328	BA.2	BA.2	BA.2_1	LSPA-3D22F9C|EPI_ISL_11979328|United Kingdom|BA.2|2022-04-04	99,69
EPI_ISL_11982401	BA.1.1.15	BA.1.1.7	BA.1.1.7	LH-NVRL-ecS22IRL00107529|EPI_ISL_11982401|Ireland|BA.1.1.15|2022-02-01	99,71
EPI_ISL_11983390	BA.1.17.2	BA.1	BA.1_2	AM-FIOCRUZ-ILMD2203898|EPI_ISL_11983390|Brazil|BA.1.17.2|2022-02-07	99,64
EPI_ISL_11986006	BA.2.9	A	A_17	LIG-265075782|EPI_ISL_11986006|Italy|BA.2.9|2022-04-04	99,81
EPI_ISL_11986024	BA.2	A	A_17	LIG-264833074|EPI_ISL_11986024|Italy|BA.2|2022-04-04	99,81
EPI_ISL_11986563	BA.2	A	A_17	DCGC-474493|EPI_ISL_11986563|Denmark|BA.2|2022-04-05	99,81
EPI_ISL_11987006	BA.2.9	A	A_17	DCGC-474852|EPI_ISL_11987006|Denmark|BA.2.9|2022-04-05	99,81
EPI_ISL_11987164	BA.2.9	A	A_17	DCGC-475013|EPI_ISL_11987164|Denmark|BA.2.9|2022-04-04	99,81
EPI_ISL_11987382	BA.2	A	A_17	DCGC-475135|EPI_ISL_11987382|Denmark|BA.2|2022-04-05	99,77
EPI_ISL_11988591	BA.2	BA.2	BA.2_1	DCGC-475998|EPI_ISL_11988591|Denmark|BA.2|2022-04-05	99,77
EPI_ISL_11988833	BA.2.9	A	A_17	DCGC-476125|EPI_ISL_11988833|Denmark|BA.2.9|2022-04-04	99,81
EPI_ISL_11988862	BA.2	BA.2	BA.2_1	ARCH-003003C1|EPI_ISL_11988862|United Kingdom|BA.2|2022	99,81
EPI_ISL_11990552	BA.2.9	A	A_17	DCGC-476889|EPI_ISL_11990552|Denmark|BA.2.9|2022-04-04	99,81
EPI_ISL_11990878	BA.2	A	A_17	DCGC-477285|EPI_ISL_11990878|Denmark|BA.2|2022-04-05	99,77
EPI_ISL_11991140	BA.2	A	A_17	DCGC-477465|EPI_ISL_11991140|Denmark|BA.2|2022-04-05	99,79
EPI_ISL_12000378	BA.1.15	BA.1	BA.1_4	CA-SEARCH-119653|EPI_ISL_12000378|USA|BA.1.15|2022-01-12	99,03
EPI_ISL_12001178	BA.2	A	A_14	KY-SDx-X2200919078|EPI_ISL_12001178|USA|BA.2|2022-03-02	99,81
EPI_ISL_12001197	BA.2	BA.2	BA.2_1	MA-NEIDL-05436|EPI_ISL_12001197|USA|BA.2|2022-03-22	98,07
EPI_ISL_12006017	BA.2.9	A	A_17	LHUB-ULB_31264753|EPI_ISL_12006017|Belgium|BA.2.9|2022-03-25	70,46
EPI_ISL_12006087	BA.2	A	A_14	LHUB-ULB_SNI237|EPI_ISL_12006087|Belgium|BA.2|2022-03-30	70,48
EPI_ISL_12008155	BA.2	BA.2	BA.2_1	D-NVRL-S22IRL00264122|EPI_ISL_12008155|Ireland|BA.2|2022-03-22	90,50
EPI_ISL_12009524	BA.2	BA.2	BA.2_1	DC-Curative-253061|EPI_ISL_12009524|USA|BA.2|2022-03-31	72,05
EPI_ISL_12009584	BA.2	A	A_1	MA-Curative-263034|EPI_ISL_12009584|USA|BA.2|2022-03-27	58,25
EPI_ISL_12010589	BA.2	BA.2	BA.2_1	QEUH-3CFE844|EPI_ISL_12010589|United Kingdom|BA.2|2022-03-31	99,81
EPI_ISL_12010636	BA.2	A	A_14	QEUH-3CEC22D|EPI_ISL_12010636|United Kingdom|BA.2|2022-03-22	99,81
EPI_ISL_12010697	BA.2	A	A_14	QEUH-3CF876B|EPI_ISL_12010697|United Kingdom|BA.2|2022-03-29	99,81
EPI_ISL_12017588	BA.2.9	A	A_17	NW-RKI-I-707568|EPI_ISL_12017588|Germany|BA.2.9|2022-03-26	99,81
EPI_ISL_12019583	XM	A	A_14	SH-RKI-I-709371|EPI_ISL_12019583|Germany|XM|2022-04-01	99,77
EPI_ISL_12021738	BA.2	BA.2	BA.2_1	SN-RKI-I-710735|EPI_ISL_12021738|Germany|BA.2|2022-03-23	99,73
EPI_ISL_12021909	BA.2.10.1	BA.2	BA.2_1	SN-RKI-I-710827|EPI_ISL_12021909|Germany|BA.2.10.1|2022-03-24	99,77
EPI_ISL_12022710	BA.2	A	A_17	DCGC-478306|EPI_ISL_12022710|Denmark|BA.2|2022-04-08	99,81
EPI_ISL_12024315	BA.2	A	A_14	Austria|EPI_ISL_12024315|Austria|BA.2|2022-03-22	99,81
EPI_ISL_12027177	BA.2.9	A	A_17	NW-RKI-I-715287|EPI_ISL_12027177|Germany|BA.2.9|2022-03-30	99,81
EPI_ISL_12027187	BA.2	A	A_17	NW-RKI-I-715297|EPI_ISL_12027187|Germany|BA.2|2022-03-31	99,81
EPI_ISL_12028410	BA.1.1.18	BA.1.1	BA.1.1_3	CO-CDPHE-2103089678|EPI_ISL_12028410|USA|BA.1.1.18|2022-01-09	81,22
EPI_ISL_12028704	BA.1	BA.1	BA.1_1	MOH-K-OPD13-MN-174|EPI_ISL_12028704|Kenya|BA.1|2021-12-14	94,66
EPI_ISL_12031189	BA.2	A	A_14	PHWC-PJU8QU|EPI_ISL_12031189|United Kingdom|BA.2|2022-03-23	99,81
EPI_ISL_12033125	BA.2	BA.2	BA.2_1	NSW-ICPMR-23596|EPI_ISL_12033125|Australia|BA.2|2022-04-03	79,36
EPI_ISL_12034907	BA.1.1.18	BA.1.1	BA.1.1_3	CO-CDPHE-2103101415|EPI_ISL_12034907|USA|BA.1.1.18|2022-01-01	94,33
EPI_ISL_12035818	BA.1.1	BA.1	BA.1_4	CO-CDPHE-2103089681|EPI_ISL_12035818|USA|BA.1.1|2022-01-17	89,72
EPI_ISL_12036089	BA.2	BA.2	BA.2_1	CAM-TIGEM-IZSM-COLLI-29330|EPI_ISL_12036089|Italy|BA.2|2022	99,56
EPI_ISL_12042241	BA.2.9	BA.1.15	BA.1.15	3861154924|EPI_ISL_12042241|Sweden|BA.2.9|2022-03-31	93,61
EPI_ISL_12044317	BA.2	BA.2	BA.2_1	PHEC-YYRNA15|EPI_ISL_12044317|United Kingdom|BA.2|2022-03-20	99,81
EPI_ISL_12044870	BA.2	BA.2	BA.2_1	PHEC-YYRYCTP|EPI_ISL_12044870|United Kingdom|BA.2|2022-03-28	99,71
EPI_ISL_12046872	BA.2.9	A	A_17	AZ-CDC-LC0569472|EPI_ISL_12046872|USA|BA.2.9|2022-03-21	99,81
EPI_ISL_12047122	BA.1.17	BA.1	BA.1_2	WA-CDC-LC0569954|EPI_ISL_12047122|USA|BA.1.17|2022-03-28	99,64
EPI_ISL_12047707	BA.2	A	A_14	SJ3220325354|EPI_ISL_12047707|Belgium|BA.2|2022-03-14	99,81
EPI_ISL_12048610	BA.2.62	A	A_1	QLD0x00E012|EPI_ISL_12048610|Australia|BA.2.62|2022-03-21	66,27
EPI_ISL_12049100	BA.2	A	A_14	NJ-PHEL-V22016543|EPI_ISL_12049100|USA|BA.2|2022-04-02	90,52
EPI_ISL_12049122	BA.1.1	BA.1.1	BA.1.1_2	OR-CDC-ASC210735761|EPI_ISL_12049122|USA|BA.1.1|2022-03-16	98,28
EPI_ISL_12049599	BA.2	A	A_14	MA-CDC-ASC210738778|EPI_ISL_12049599|USA|BA.2|2022-03-24	99,81
EPI_ISL_12050688	BA.2.10	A	A_14	VA-CDC-ASC210848361|EPI_ISL_12050688|USA|BA.2.10|2022-03-27	99,81
EPI_ISL_12052050	BA.2	BA.2.10	BA.2.10	SCOT-8633|EPI_ISL_12052050|United Kingdom|BA.2|2022-03-19	99,81
EPI_ISL_12052936	BA.1.15	BA.1	BA.1_4	CA-CDPH-500040117|EPI_ISL_12052936|USA|BA.1.15|2021-12-16	98,05
EPI_ISL_12060162	BA.1	BA.1.15	BA.1.15	SC-FIOCRUZ-81077CE|EPI_ISL_12060162|Brazil|BA.1|2022-01-21	99,64
EPI_ISL_12060279	BA.1	BA.1	BA.1_4	MO-MSPHL-007743|EPI_ISL_12060279|USA|BA.1|2022-01-18	85,65
EPI_ISL_12063769	BA.1.18	BA.1.15	BA.1.15	BY-MVP-000012833|EPI_ISL_12063769|Germany|BA.1.18|2022-03-01	99,64
EPI_ISL_12070356	BA.2.9	BA.2	BA.2_1	VER-INMEGEN-66-122|EPI_ISL_12070356|Mexico|BA.2.9|2022-03-28	93,09
EPI_ISL_12080076	BA.2	A	A_14	PHEP-YYRZN3A|EPI_ISL_12080076|United Kingdom|BA.2|2022-03-30	99,77
EPI_ISL_12083794	BA.2.3	BA.2.10	BA.2.10	DC-Curative-236801|EPI_ISL_12083794|USA|BA.2.3|2022-04-06	93,80
EPI_ISL_12088218	BA.1.1.18	BA.1.1	BA.1.1_3	CO-CDPHE-2103085378|EPI_ISL_12088218|USA|BA.1.1.18|2022-01-11	99,64
EPI_ISL_12090201	BA.1.1	BA.1.15	BA.1.15	AZ-ASU61591|EPI_ISL_12090201|USA|BA.1.1|2022-01-06	99,56
EPI_ISL_12090758	BA.1.17.2	BA.1.15	BA.1.15	CVL-8011877|EPI_ISL_12090758|Israel|BA.1.17.2|2022-03-16	73,80
EPI_ISL_12091824	BA.2	BA.2	BA.2_1	CVL-9002445|EPI_ISL_12091824|Israel|BA.2|2022-03-24	99,39
EPI_ISL_12092618	BA.1.1	BA.1.1	BA.1.1_3	CVL-8010253|EPI_ISL_12092618|Israel|BA.1.1|2022-02-17	99,64
EPI_ISL_12094469	BA.1.1	BA.1.1	BA.1.1_2	CVL-26730|EPI_ISL_12094469|Israel|BA.1.1|2022-03-06	99,62
EPI_ISL_12094702	BA.2	BA.2	BA.2_1	CVL-26993|EPI_ISL_12094702|Israel|BA.2|2022-03-06	99,81

EPI_ISL_12095029	BA.1.1	BA.1.15	BA.1.15	CVL-9001457|EPI_ISL_12095029|Israel|BA.1.1|2022-03-11	78,92
EPI_ISL_12096665	BA.2	BA.2	BA.2_1	LSPA-3D3FEDB|EPI_ISL_12096665|United Kingdom|BA.2|2022-04-10	99,81
EPI_ISL_12097400	BA.2	BA.2	BA.2_1	CERI-KRISP-K039824|EPI_ISL_12097400|South Africa|BA.2|2022-04-08	90,77
EPI_ISL_12097486	BA.2	BA.2	BA.2_1	LSPA-3D53141|EPI_ISL_12097486|United Kingdom|BA.2|2022-04-12	93,78
EPI_ISL_12097522	BA.2	A	A_14	LSPA-3D53017|EPI_ISL_12097522|United Kingdom|BA.2|2022-04-09	99,81
EPI_ISL_12098538	BA.1.1.2	BA.1.1	BA.1.1_3	RIMD08041|EPI_ISL_12098538|Japan|BA.1.1.2|2022-02-12	99,64
EPI_ISL_12098588	BC.1	BC.1	BC.1	RIMD08091|EPI_ISL_12098588|Japan|BC.1|2022-02-15	99,64
EPI_ISL_12099443	BA.2	BA.2	BA.2_1	LB-R00070-S297|EPI_ISL_12099443|Austria|BA.2|2022-04-12	94,73
EPI_ISL_12103368	BA.2	BA.2	BA.2_1	PHEC-YYREKDH|EPI_ISL_12103368|United Kingdom|BA.2|2022-03-23	93,89
EPI_ISL_12104410	BA.2	BA.2	BA.2_1	PHEC-YYRK3CZ|EPI_ISL_12104410|United Kingdom|BA.2|2022-03-30	95,44
EPI_ISL_12106536	BA.2	A	A_14	PHEP-YYRAFS3|EPI_ISL_12106536|United Kingdom|BA.2|2022-04-03	99,79
EPI_ISL_12109996	BA.2.3	BA.1	BA.1_3	DC-Curative-260569|EPI_ISL_12109996|USA|BA.2.3|2022-04-08	87,06
EPI_ISL_12110417	BA.2.3	A	A_14	VIC48368|EPI_ISL_12110417|Australia|BA.2.3|2022-03-21	99,81
EPI_ISL_12111063	BA.2.3	A	A_14	VIC50074|EPI_ISL_12111063|Australia|BA.2.3|2022-03-25	99,81
EPI_ISL_12112533	BA.2	BA.2.10	BA.2.10	NJ-CDC-ASC210848936|EPI_ISL_12112533|USA|BA.2|2022-03-29	99,81
EPI_ISL_12113419	BA.2.3	A	A_14	AK-CDC-ASC210722317|EPI_ISL_12113419|USA|BA.2.3|2022-04-01	99,81
EPI_ISL_12113808	BA.2.57	A	A_14	VIC49928|EPI_ISL_12113808|Australia|BA.2.57|2022-03-29	99,81
EPI_ISL_12114406	BA.2.7	A	A_17	MA-CDC-ASC210851304|EPI_ISL_12114406|USA|BA.2.7|2022-04-02	99,81
EPI_ISL_12115158	BA.2	BA.2	BA.2_1	VIC50932|EPI_ISL_12115158|Australia|BA.2|2022-04-04	99,81
EPI_ISL_12115364	BA.2.3	A	A_4	NY-CDC-ASC210851216|EPI_ISL_12115364|USA|BA.2.3|2022-04-03	99,81
EPI_ISL_12115469	BA.2.31	BA.2	BA.2_1	UT-CDC-ASC210723070|EPI_ISL_12115469|USA|BA.2.31|2022-04-04	99,81
EPI_ISL_12116021	BA.2	BA.2.3.6	BA.2.3.6	MI-CDC-ASC210852201|EPI_ISL_12116021|USA|BA.2|2022-04-04	99,81
EPI_ISL_12116423	BA.2.3.4	BA.2.3.4	BA.2.3.4	NJ-CDC-ASC210723024|EPI_ISL_12116423|USA|BA.2.3.4|2022-04-05	99,81
EPI_ISL_12116474	BA.2.12.1	BA.2.12.1	BA.2.12.1	OR-CDC-ASC210852291|EPI_ISL_12116474|USA|BA.2.12.1|2022-04-05	99,81
EPI_ISL_12117250	BA.2.10.1	BA.2.10.1	BA.2.10.1	MA-CDC-ASC210852428|EPI_ISL_12117250|USA|BA.2.10.1|2022-04-06	99,81
EPI_ISL_12118471	BA.2	A	A_14	APU-IZSPB-PT6787|EPI_ISL_12118471|Italy|BA.2|2022-04-07	95,65
EPI_ISL_12121752	BA.2	BA.2	BA.2_1	LSPA-3D57769|EPI_ISL_12121752|United Kingdom|BA.2|2022-04-12	95,29
EPI_ISL_12122076	BA.2	A	A_17	LSPA-3D5B03D|EPI_ISL_12122076|United Kingdom|BA.2|2022-04-13	99,81
EPI_ISL_12122152	BA.2	BA.2	BA.2_1	LSPA-3D5A9AC|EPI_ISL_12122152|United Kingdom|BA.2|2022-04-11	99,81
EPI_ISL_12122869	BA.2	BA.2	BA.2_1	LSPA-3D515A1|EPI_ISL_12122869|United Kingdom|BA.2|2022-04-11	99,81
EPI_ISL_12123230	BA.2	BA.2	BA.2_1	QEUH-3D4ED81|EPI_ISL_12123230|United Kingdom|BA.2|2022-04-12	99,81
EPI_ISL_12123417	BA.1.1	BA.1.1	BA.1.1_2	VA-VTVAS3-GSC32858|EPI_ISL_12123417|USA|BA.1.1|2022-01-25	92,23
EPI_ISL_12127360	BA.2.35	BA.2	BA.2_3	PT32040|EPI_ISL_12127360|Portugal|BA.2.35|2022-04-05	99,81
EPI_ISL_12131566	BA.2	BA.2	BA.2_1	CO-NVRL-S22IRL00267484|EPI_ISL_12131566|Ireland|BA.2|2022-03-22	90,52
EPI_ISL_12132072	BA.2.10	BA.2	BA.2_1	ARA-HMN-22032300430|EPI_ISL_12132072|France|BA.2.10|2022-03-07	95,17
EPI_ISL_12134285	BA.2	A	A_14	PDL-IPP25467|EPI_ISL_12134285|France|BA.2|2022-03-28	99,81
EPI_ISL_12138041	BA.2	BA.2	BA.2_1	DCGC-479338|EPI_ISL_12138041|Denmark|BA.2|2022-04-09	99,79
EPI_ISL_12138203	BA.2	BA.2	BA.2_1	DCGC-479467|EPI_ISL_12138203|Denmark|BA.2|2022-02-03	94,26
EPI_ISL_12138665	BA.2	BA.2	BA.2_1	BA_22_00025258|EPI_ISL_12138665|Slovakia|BA.2|2022-04-06	86,95
EPI_ISL_12139983	BA.2.9.5	A	A_17	NIC_PTE_14850|EPI_ISL_12139983|Thailand|BA.2.9.5|2022-03	99,81
EPI_ISL_12141682	BA.2	A	A_17	NE-NPHL22-14505|EPI_ISL_12141682|USA|BA.2|2022-04-14	90,52
EPI_ISL_12143813	BA.1.1	BA.1	BA.1_4	WY-WSVL-0382622393|EPI_ISL_12143813|USA|BA.1.1|2022-01-20	68,52
EPI_ISL_12146012	BA.1.1	BA.1.1	BA.1.1_2	VA-VTVAS3-GSC30874|EPI_ISL_12146012|USA|BA.1.1|2022-01-06	99,64
EPI_ISL_12146649	BA.1.1	BA.1.1	BA.1.1_2	QC-L00458748001|EPI_ISL_12146649|Canada|BA.1.1|2022-03-25	99,64
EPI_ISL_12147262	BA.1.1	BA.1.1	BA.1.1_2	CA-SEARCH-84494|EPI_ISL_12147262|USA|BA.1.1|2022-01-31	99,48
EPI_ISL_12147419	BA.1.1	BA.1.1	BA.1.1_2	CA-SEARCH-84792|EPI_ISL_12147419|USA|BA.1.1|2022-02-11	99,48
EPI_ISL_12147613	BA.1.1	BA.1.1	BA.1.1_2	CA-SEARCH-84965|EPI_ISL_12147613|USA|BA.1.1|2022-02-13	99,48
EPI_ISL_12148143	BA.2	BA.2	BA.2_1	MA-CADIUMAG-02490|EPI_ISL_12148143|Chile|BA.2|2022-02-02	90,38
EPI_ISL_12148297	BA.2.32	BA.2.32	BA.2.32	IMR_WC221581|EPI_ISL_12148297|Malaysia|BA.2.32|2022-03-26	99,81
EPI_ISL_12149766	BA.2.7	A	A_17	TX-CDC-QDX35528640|EPI_ISL_12149766|USA|BA.2.7|2022-04-03	99,81
EPI_ISL_12153381	BA.2.9	BA.2	BA.2_1	ST-MD7460|EPI_ISL_12153381|Germany|BA.2.9|2022-03-01	94,26
EPI_ISL_12155479	BA.2.10	BA.2	BA.2_1	UP-ICMR-MCL-22-602_9197|EPI_ISL_12155479|India|BA.2.10|2022-02	99,81
EPI_ISL_12155563	BA.2	BA.2	BA.2_1	AZDelta-2215-00834|EPI_ISL_12155563|Belgium|BA.2|2022-04-14	97,67
EPI_ISL_12157415	BA.2.69	A	A_17	IDF-IPP26562|EPI_ISL_12157415|France|BA.2.69|2022-03-27	99,81
EPI_ISL_12161043	BA.2.18	BA.2.18	BA.2.18	PHEP-YYRA4RJ|EPI_ISL_12161043|United Kingdom|BA.2.18|2022-03-27	99,77
EPI_ISL_12161551	BA.2	A	A_14	PHEP-YYRATFO|EPI_ISL_12161551|United Kingdom|BA.2|2022-04-03	99,73
EPI_ISL_12162280	BA.2.9	BA.2	BA.2_1	DCGC-483183|EPI_ISL_12162280|Denmark|BA.2.9|2022-04-09	99,79
EPI_ISL_12162424	BA.2.9	BA.2	BA.2_1	DCGC-483330|EPI_ISL_12162424|Denmark|BA.2.9|2022-04-10	99,77
EPI_ISL_12162958	BA.2	BA.2	BA.2_1	DCGC-483835|EPI_ISL_12162958|Denmark|BA.2|2022-04-12	99,81
EPI_ISL_12164075	BA.2.9	A	A_17	DCGC-484880|EPI_ISL_12164075|Denmark|BA.2.9|2022-04-12	99,98
EPI_ISL_12164673	BA.2.9	A	A_17	DCGC-485403|EPI_ISL_12164673|Denmark|BA.2.9|2022-04-05	99,98
EPI_ISL_12165281	BA.2	BA.2	BA.2_1	DCGC-486016|EPI_ISL_12165281|Denmark|BA.2|2022-04-12	99,75
EPI_ISL_12165322	BA.2.9	A	A_17	DCGC-486057|EPI_ISL_12165322|Denmark|BA.2.9|2022-04-10	99,75
EPI_ISL_12165430	BA.2	A	A_17	DCGC-486166|EPI_ISL_12165430|Denmark|BA.2|2022-04-12	95,82
EPI_ISL_12166067	BA.2.9	A	A_17	DCGC-486811|EPI_ISL_12166067|Denmark|BA.2.9|2022-04-07	99,81
EPI_ISL_12166778	BA.2.30	BA.2	BA.2_1	AZ-CDC-STM-SE5DPDDKT|EPI_ISL_12166778|USA|BA.2.30|2022-04-02	99,81
EPI_ISL_12168977	BA.2.12.1	BA.2.12.1	BA.2.12.1	NJ-CDC-LC0575446|EPI_ISL_12168977|USA|BA.2.12.1|2022-04-07	99,81
EPI_ISL_12169046	BA.2	A	A_14	VA-CDC-LC0575039|EPI_ISL_12169046|USA|BA.2|2022-04-07	93,68
EPI_ISL_12169469	BA.2	A	A_4	LSMULKKGMMK42C14|EPI_ISL_12169469|Lithuania|BA.2|2022-03-29	99,79
EPI_ISL_12169765	BA.2.3	A	A_14	HI-CDC-LC0573674|EPI_ISL_12169765|USA|BA.2.3|2022-04-09	99,81
EPI_ISL_12169787	BA.2.10	A	A_14	OR-CDC-LC0573607|EPI_ISL_12169787|USA|BA.2.10|2022-04-09	99,81
EPI_ISL_12171043	BA.2	A	A_14	LSMULKKGMMK44C2|EPI_ISL_12171043|Lithuania|BA.2|2022-04-09	99,81
EPI_ISL_12173005	BA.2	A	A_14	ON-KHS-22-04520-v1|EPI_ISL_12173005|Canada|BA.2|2022-04-03	99,81
EPI_ISL_12173038	BA.2	BA.2	BA.2_1	ON-KHS-22-04567-v1|EPI_ISL_12173038|Canada|BA.2|2022-04-01	99,81
EPI_ISL_12173547	BA.1.1	BA.1.1	BA.1.1_3	OR-OSPHL05460|EPI_ISL_12173547|USA|BA.1.1|2022-04-10	99,60
EPI_ISL_12174241	BA.1.1	BA.1.1	BA.1.1_2	WA-CDC-UW22040196610|EPI_ISL_12174241|USA|BA.1.1|2022-04-01	92,46
EPI_ISL_12174350	BA.2.3	BA.2	BA.2_1	WA-CDC-UW22033066340|EPI_ISL_12174350|USA|BA.2.3|2022-03-30	88,27
EPI_ISL_12179520	BA.2	BA.2	BA.2_1	NAT-22-23084|EPI_ISL_12179520|Czech Republic|BA.2|2022-03-21	99,81
EPI_ISL_12179561	BA.1.1	BA.1	BA.1_1	NAT-22-23238|EPI_ISL_12179561|Czech Republic|BA.1.1|2022-03-23	95,04
EPI_ISL_12179768	BA.2	A	A_14	ARA-IPP26907|EPI_ISL_12179768|France|BA.2|2022-03-28	99,81
EPI_ISL_12184143	BA.2	BA.2	BA.2_1	IL-CDC-ASC210725059|EPI_ISL_12184143|USA|BA.2|2022-04-08	99,81
EPI_ISL_12185494	BA.2	A	A_14	RP-RKI-I-716022|EPI_ISL_12185494|Germany|BA.2|2022-04-01	99,77
EPI_ISL_12185857	BA.1.1	BA.1.1	BA.1.1_2	BW-RKI-I-716468|EPI_ISL_12185857|Germany|BA.1.1|2022-03-20	99,64
EPI_ISL_12186815	BA.2.9	A	A_17	BW-RKI-I-717416|EPI_ISL_12186815|Germany|BA.2.9|2022-03-21	98,51
EPI_ISL_12187211	BA.2	A	A_17	BY-RKI-I-717949|EPI_ISL_12187211|Germany|BA.2|2022-03-20	99,81
EPI_ISL_12187960	BA.1.18	BA.1	BA.1_2	NW-RKI-I-718848|EPI_ISL_12187960|Germany|BA.1.18|2022-03-26	99,52
EPI_ISL_12188983	BA.2	BA.2	BA.2_1	SH-RKI-I-720364|EPI_ISL_12188983|Germany|BA.2|2022-04-05	94,26
EPI_ISL_12189187	BA.1.1	BA.1.1	BA.1.1_3	BW-RKI-I-720699|EPI_ISL_12189187|Germany|BA.1.1|2022-02-03	99,43
EPI_ISL_12191265	BA.2	A	A_17	BY-RKI-I-722625|EPI_ISL_12191265|Germany|BA.2|2022-04-08	99,81
EPI_ISL_12192184	BA.2	BA.2	BA.2_1	NW-RKI-I-723153|EPI_ISL_12192184|Germany|BA.2|2022-03-31	99,79
EPI_ISL_12193528	BA.2.10	BA.2	BA.2_1	NW-RKI-I-724375|EPI_ISL_12193528|Germany|BA.2.10|2022-04-04	99,81
EPI_ISL_12195863	BA.2.9	BA.2	BA.2_1	BW-RKI-I-726375|EPI_ISL_12195863|Germany|BA.2.9|2022-04-06	99,81
EPI_ISL_12196266	BA.1.1	BA.1.1	BA.1.1_3	BW-RKI-I-726694|EPI_ISL_12196266|Germany|BA.1.1|2022-04-07	99,64
EPI_ISL_12196889	BA.2.9.5	A	A_12	HE-RKI-I-727236|EPI_ISL_12196889|Germany|BA.2.9.5|2022-04-02	99,75
EPI_ISL_12197159	BA.2	BA.2.12	BA.2.12	SH-RKI-I-727564|EPI_ISL_12197159|Germany|BA.2|2022-04-11	94,20
EPI_ISL_12197763	BA.2.9	A	A_17	NW-RKI-I-728068|EPI_ISL_12197763|Germany|BA.2.9|2022-04-06	99,01
EPI_ISL_12198732	BA.2.9	BA.2	BA.2_1	SH-RKI-I-729354|EPI_ISL_12198732|Germany|BA.2.9|2022-04-04	99,81
EPI_ISL_12198759	BA.2.9	A	A_17	SH-RKI-I-729383|EPI_ISL_12198759|Germany|BA.2.9|2022-04-05	99,81
EPI_ISL_12200491	BA.1.1	BA.1.1	BA.1.1_2	AZ-ASU63003|EPI_ISL_12200491|USA|BA.1.1|2022-01-07	92,92
EPI_ISL_12202003	BA.1.1	BA.1	BA.1_4	CO-CDPHE-2103124999|EPI_ISL_12202003|USA|BA.1.1|2022-03-14	83,40
EPI_ISL_12202552	BA.2.9	A	A_17	ON-KHS-22-04974-v1|EPI_ISL_12202552|Canada|BA.2.9|2022-04-07	99,79

EPI_ISL_12203225	BA.1.15	BA.1	BA.1_2	CA-SC-265750|EPI_ISL_12203225|USA|BA.1.15|2022-02-23	94,58
EPI_ISL_12204218	BA.2	BA.2	BA.2_1	PHEC-YYRO8ZF|EPI_ISL_12204218|United Kingdom|BA.2|2022-04-01	98,51
EPI_ISL_12205160	BA.2	A	A_17	PHEC-YYRX9WS|EPI_ISL_12205160|United Kingdom|BA.2|2022-04-04	99,81
EPI_ISL_12205366	BA.2	BA.2	BA.2_1	AZ-TG1315450|EPI_ISL_12205366|USA|BA.2|2022-03-27	99,81
EPI_ISL_12208795	BA.2.9	BA.2	BA.2_1	UZB_548202204158|EPI_ISL_12208795|Belgium|BA.2.9|2022-04-13	99,81
EPI_ISL_12210978	BA.2.18	BA.2.18	BA.2.18	LSPA-3D78586|EPI_ISL_12210978|United Kingdom|BA.2.18|2022-04-15	99,81
EPI_ISL_12211657	BA.2	A	A_14	QEUH-3D70E74|EPI_ISL_12211657|United Kingdom|BA.2|2022-04-15	99,81
EPI_ISL_12211678	BA.2.23	A	A_14	QEUH-3D70D95|EPI_ISL_12211678|United Kingdom|BA.2.23|2022-04-14	99,81
EPI_ISL_12213472	BA.2	A	A_14	C19UMB1945|EPI_ISL_12213472|Malaysia|BA.2|2022-04-04	90,52
EPI_ISL_12214377	BA.1.17.2	BA.1.17.2	BA.1.17.2	ON-KHS-22-04217-v1|EPI_ISL_12214377|Canada|BA.1.17.2|2022-03-30	91,20
EPI_ISL_12215823	BA.2	BA.2	BA.2_1	ARCH-003056G9|EPI_ISL_12215823|United Kingdom|BA.2|2022	99,81
EPI_ISL_12216276	BA.2	A	A_14	MD-HGUGM-6563538|EPI_ISL_12216276|Spain|BA.2|2022-03-31	99,81
EPI_ISL_12216734	BA.2.12.1	BA.2.12.1	BA.2.12.1	MA-Broad-CRSP_CFMX5BUXQXPA3HDM|EPI_ISL_12216734|USA|BA.2.12.1|2022-04-03	99,81
EPI_ISL_12219139	BA.2	BA.2	BA.2_1	CMX-INMEGEN-67-54|EPI_ISL_12219139|Mexico|BA.2|2022-04-12	86,49
EPI_ISL_12221155	BA.2	A	A_14	EDB55506|EPI_ISL_12221155|United Kingdom|BA.2|2022-03-25	99,81
EPI_ISL_12221534	BA.2.65	BA.2	BA.2_1	NHLS-UCT-GP-O584|EPI_ISL_12221534|South Africa|BA.2.65|2022-03-28	95,55
EPI_ISL_12222471	BA.2	A	A_14	PHEC-YYR1WBB|EPI_ISL_12222471|United Kingdom|BA.2|2022-04-06	99,81
EPI_ISL_12223021	BA.2	BA.2	BA.2_1	PHEC-YYRTZOB|EPI_ISL_12223021|United Kingdom|BA.2|2022-03-29	88,55
EPI_ISL_12223291	BA.2	A	A_14	PHEP-YYR3EP8|EPI_ISL_12223291|United Kingdom|BA.2|2022-04-05	99,81
EPI_ISL_12225568	BA.1	BA.1.1	BA.1.1_3	SK-RRPL-400590|EPI_ISL_12225568|Canada|BA.1|2022-03-22	99,64
EPI_ISL_12228065	BA.2.47	BA.2.47	BA.2.47	IA-SHL-2080507|EPI_ISL_12228065|USA|BA.2.47|2022-04-05	90,52
EPI_ISL_12228953	BA.2.23	BA.2.12.1	BA.2.12.1	WA-PHL-017630|EPI_ISL_12228953|USA|BA.2.23|2022-03-25	76,23
EPI_ISL_12232075	BA.1.1	BA.1.1	BA.1.1_2	MA-Broad-CRSP_34BWZ3ZTKUDYDKIV|EPI_ISL_12232075|USA|BA.1.1|2022-04-08	99,64
EPI_ISL_12232320	BA.1.1	BA.1.1	BA.1.1_2	MA-Broad-CRSP_NS35Q6CDUMXAF7DV|EPI_ISL_12232320|USA|BA.1.1|2022-04-08	99,64
EPI_ISL_12235165	BA.1.1	BA.1.1	BA.1.1_2	DF-NVBS13676GENOV828739795475|EPI_ISL_12235165|Brazil|BA.1.1|2022-02-01	99,64
EPI_ISL_12236257	BA.2	A	A_14	LSPA-3D7F6D7|EPI_ISL_12236257|United Kingdom|BA.2|2022-04-16	99,81
EPI_ISL_12236913	BA.2	A	A_14	LSPA-3D72CE1|EPI_ISL_12236913|United Kingdom|BA.2|2022-04-16	99,81
EPI_ISL_12237152	BA.2	A	A_14	LSPA-3D7851D|EPI_ISL_12237152|United Kingdom|BA.2|2022-04-16	99,81
EPI_ISL_12237747	BA.2	A	A_14	QEUH-3D7B3E3|EPI_ISL_12237747|United Kingdom|BA.2|2022-04-16	99,81
EPI_ISL_12237942	BA.2	BA.2	BA.2_1	QEUH-3D7A302|EPI_ISL_12237942|United Kingdom|BA.2|2022-04-16	99,81
EPI_ISL_12238051	BA.2	A	A_17	CA-LACPHL-AF08665|EPI_ISL_12238051|USA|BA.2|2022-03-28	90,52
EPI_ISL_12238559	BA.1.1	BA.1.1	BA.1.1_3	ON-PHL-22-18041|EPI_ISL_12238559|Canada|BA.1.1|2022-04-05	99,64
EPI_ISL_12239320	BA.2	BA.2	BA.2_1	ON-PHL-22-18851|EPI_ISL_12239320|Canada|BA.2|2022-04-07	94,09
EPI_ISL_12239398	BA.1.1	BA.1.1	BA.1.1_3	ON-PHL-22-18930|EPI_ISL_12239398|Canada|BA.1.1|2022-04-08	99,64
EPI_ISL_12241009	BA.2.36	BA.2	BA.2_2	UMONS-P062655401|EPI_ISL_12241009|Belgium|BA.2.36|2022-04-19	99,81
EPI_ISL_12243238	BA.2	A	A_14	IC-7776|EPI_ISL_12243238|Japan|BA.2|2022-04-01	99,81
EPI_ISL_12244519	BA.2	BA.2	BA.2_1	DCGC-489046|EPI_ISL_12244519|Denmark|BA.2|2022-04-17	99,81
EPI_ISL_12244919	BA.2.9	BA.2	BA.2_1	DCGC-489448|EPI_ISL_12244919|Denmark|BA.2.9|2022-04-11	92,54
EPI_ISL_12245158	BA.2	BA.2	BA.2_1	DCGC-489688|EPI_ISL_12245158|Denmark|BA.2|2022-04-17	99,77
EPI_ISL_12248100	BA.2	A	A_17	DCGC-492648|EPI_ISL_12248100|Denmark|BA.2|2022-04-13	99,94
EPI_ISL_12248854	BA.2	A	A_17	PAC-CHUNICE-2204100927|EPI_ISL_12248854|France|BA.2|2022-04-10	99,81
EPI_ISL_12252212	BA.2	A	A_14	CT-HUB04716|EPI_ISL_12252212|Spain|BA.2|2022-03-30	99,81
EPI_ISL_12253566	BA.2	A	A_14	LSPA-3D8ECF1|EPI_ISL_12253566|United Kingdom|BA.2|2022-04-19	99,81
EPI_ISL_12254131	BA.2.9	A	A_17	LSMULKKGMMK45C150|EPI_ISL_12254131|Lithuania|BA.2.9|2022-04-11	99,79
EPI_ISL_12254551	BA.2	BA.2	BA.2_1	NH-NMDL-01633|EPI_ISL_12254551|Netherlands|BA.2|2022-04-06	98,51
EPI_ISL_12255478	BA.2	BA.2	BA.2_1	NY-PRL-2022_0413_00F05|EPI_ISL_12255478|USA|BA.2|2022-04-10	99,81
EPI_ISL_12258127	BA.2.18	BA.2.18	BA.2.18	NY-PRL-2022_0418_01D19|EPI_ISL_12258127|USA|BA.2.18|2022-04-17	99,81
EPI_ISL_12258823	BA.2	A	A_4	PHEC-YYRICKT|EPI_ISL_12258823|United Kingdom|BA.2|2022-04-08	99,77
EPI_ISL_12259812	BA.2	BA.2	BA.2_1	PHEC-YYRIK6K|EPI_ISL_12259812|United Kingdom|BA.2|2022-03-31	99,77
EPI_ISL_12260934	BA.1.17	BA.1.15	BA.1.15	CVL-HMN-22032290414|EPI_ISL_12260934|France|BA.1.17|2022-01-17	99,48
EPI_ISL_12263175	BA.2	BA.2	BA.2_1	ARA-HCL722002158801|EPI_ISL_12263175|France|BA.2|2022-04-07	96,07
EPI_ISL_12263638	BA.2.9	A	A_17	BA_22_00025900|EPI_ISL_12263638|Slovakia|BA.2.9|2022-04-08	99,81
EPI_ISL_12264302	BA.2.47	BA.2.47	BA.2.47	PHEP-YYR4NTN|EPI_ISL_12264302|United Kingdom|BA.2.47|2022-04-03	94,05
EPI_ISL_12264649	BA.2	A	A_14	MI-CDC-QDX35734937|EPI_ISL_12264649|USA|BA.2|2022-04-07	99,81
EPI_ISL_12264808	BA.2.12.1	BA.2.12.1	BA.2.12.1	NJ-CDC-QDX35776562|EPI_ISL_12264808|USA|BA.2.12.1|2022-04-09	99,81
EPI_ISL_12268603	BA.2	BA.2.67	BA.2.67	ARA-HMN-22032300249|EPI_ISL_12268603|France|BA.2|2022-03-07	99,71
EPI_ISL_12273303	BA.2.3	A	A_14	TX-CDC-QDX35823890|EPI_ISL_12273303|USA|BA.2.3|2022-04-09	99,81
EPI_ISL_12273594	BA.2.9	A	A_17	NY-CDC-QDX35823586|EPI_ISL_12273594|USA|BA.2.9|2022-04-10	99,81
EPI_ISL_12273613	BA.2.3	A	A_14	TX-CDC-QDX35823922|EPI_ISL_12273613|USA|BA.2.3|2022-04-10	99,81
EPI_ISL_12274284	BA.2	A	A_14	CA-CDC-QDX35824658|EPI_ISL_12274284|USA|BA.2|2022-04-11	99,81
EPI_ISL_12274968	BA.2.3	A	A_14	MP-CDC-2-5773534|EPI_ISL_12274968|Northern Mariana Islands|BA.2.3|2022-02-25	93,70
EPI_ISL_12276145	BA.2	A	A_14	VIC52188|EPI_ISL_12276145|Australia|BA.2|2022-03-31	99,81
EPI_ISL_12276268	BA.2.10	A	A_14	VIC51985|EPI_ISL_12276268|Australia|BA.2.10|2022-04-04	99,81
EPI_ISL_12276295	BA.2.10	A	A_14	VIC52011|EPI_ISL_12276295|Australia|BA.2.10|2022-03-28	99,81
EPI_ISL_12276416	BA.2.3	BA.2	BA.2_1	VIC52317|EPI_ISL_12276416|Australia|BA.2.3|2022-04-03	99,81
EPI_ISL_12277618	BA.2.10	BA.2.12	BA.2.12	MA-Curative-271438|EPI_ISL_12277618|USA|BA.2.10|2022-04-10	85,46
EPI_ISL_12278170	BA.2	BA.1	BA.1_3	DE-Curative-202252|EPI_ISL_12278170|USA|BA.2|2022-04-15	92,25
EPI_ISL_12281132	BA.2.3	A	A_14	LSPA-3D9D7D0|EPI_ISL_12281132|United Kingdom|BA.2.3|2022-04-20	99,81
EPI_ISL_12281487	BA.2	A	A_14	QEUH-3D92647|EPI_ISL_12281487|United Kingdom|BA.2|2022-04-19	99,81
EPI_ISL_12282402	BA.2	A	A_14	QEUH-3D93C80|EPI_ISL_12282402|United Kingdom|BA.2|2022-04-19	99,81
EPI_ISL_12282405	BA.2	BA.2	BA.2_2	QEUH-3D93312|EPI_ISL_12282405|United Kingdom|BA.2|2022-04-19	99,81
EPI_ISL_12282866	BA.2.9	A	A_17	QEUH-3D950DD|EPI_ISL_12282866|United Kingdom|BA.2.9|2022-04-19	99,81
EPI_ISL_12283787	BA.2	A	A_14	QEUH-3D85D33|EPI_ISL_12283787|United Kingdom|BA.2|2022-04-18	99,81
EPI_ISL_12284255	BA.2.36	BA.2	BA.2_2	QEUH-3D84E22|EPI_ISL_12284255|United Kingdom|BA.2.36|2022-04-18	99,81
EPI_ISL_12285492	BA.2	A	A_4	NAQ-CERBAHC-0415062|EPI_ISL_12285492|France|BA.2|2022-04-04	99,81
EPI_ISL_12288107	BA.2	A	A_17	DCGC-494337|EPI_ISL_12288107|Denmark|BA.2|2022-04-11	99,98
EPI_ISL_12288361	BA.2.3.15	BA.2.12	BA.2.12	SIC-CQRC-3422026924|EPI_ISL_12288361|Italy|BA.2.3.15|2022-04-09	88,97
EPI_ISL_12288380	BA.2.9	A	A_17	DCGC-494391|EPI_ISL_12288380|Denmark|BA.2.9|2022-04-12	99,50
EPI_ISL_12288676	BA.2	BA.2	BA.2_1	BY-LGL-158eace3-2fec-4186-9cbe-20a760e21607|EPI_ISL_12288676|Germany|BA.2|2022-04-05	99,81
EPI_ISL_12288953	BA.2.9	BA.2	BA.2_1	DCGC-494710|EPI_ISL_12288953|Denmark|BA.2.9|2022-04-19	99,79
EPI_ISL_12297303	BA.2.12.1	BA.2.12.1	BA.2.12.1	CA-CDC-STM-Q8WTDZQ3Y|EPI_ISL_12297303|USA|BA.2.12.1|2022-04-09	99,81
EPI_ISL_12300568	BA.2.9	A	A_17	CA-CDC-STM-QCCBKJEJ3|EPI_ISL_12300568|USA|BA.2.9|2022-04-11	99,81
EPI_ISL_12301857	BA.2	BA.2	BA.2_1	RI-CDC-LC0576527|EPI_ISL_12301857|USA|BA.2|2022-04-08	99,81
EPI_ISL_12302121	BA.2.12.1	BA.2.12.1	BA.2.12.1	NC-CDC-LC0578161|EPI_ISL_12302121|USA|BA.2.12.1|2022-04-10	99,81
EPI_ISL_12304416	BA.2.3	BA.2	BA.2_1	CA-CDC-LC0580451|EPI_ISL_12304416|USA|BA.2.3|2022-04-13	99,81
EPI_ISL_12305009	BA.1.1	BA.1.1	BA.1.1_2	NC-CDC-LC0578716|EPI_ISL_12305009|USA|BA.1.1|2022-04-16	99,64
EPI_ISL_12305940	BA.1.1.18	BA.1.1	BA.1.1_3	CO-CDPHE-2102994405|EPI_ISL_12305940|USA|BA.1.1.18|2022-02-27	88,95
EPI_ISL_12305987	BA.1.1	BA.1.1	BA.1.1_2	CA-SJCPHL-2206074551|EPI_ISL_12305987|USA|BA.1.1|2022-03-22	93,30
EPI_ISL_12307774	BA.2.12.1	BA.2.12.1	BA.2.12.1	NY-Wadsworth-22017619-01|EPI_ISL_12307774|USA|BA.2.12.1|2022-04-12	99,81
EPI_ISL_12309506	BA.2.10	A	A_14	TKYkbm15383|EPI_ISL_12309506|Japan|BA.2.10|2022-03-26	99,81
EPI_ISL_12309741	BA.2	BA.2	BA.2_1	SIC-CQRC-3422029109-2021|EPI_ISL_12309741|Italy|BA.2|2022-04-20	93,70
EPI_ISL_12310476	BA.2	BA.2.10	BA.2.10	ARA-HCL022068924701|EPI_ISL_12310476|France|BA.2|2022-04-17	99,81
EPI_ISL_12310770	BA.2	BA.2	BA.2_1	NAQ-HCL722002196801|EPI_ISL_12310770|France|BA.2|2022-04-11	99,81
EPI_ISL_12316837	BA.2	A	A_17	KE-NVRL-S22IRL00264279|EPI_ISL_12316837|Ireland|BA.2|2022-03-22	90,50
EPI_ISL_12316841	BA.2	BA.2	BA.2_1	KE-NVRL-S22IRL00264289|EPI_ISL_12316841|Ireland|BA.2|2022-03-22	90,50
EPI_ISL_12317032	BA.2	A	A_17	AGES-663562|EPI_ISL_12317032|Austria|BA.2|2022-02-28	90,52
EPI_ISL_12317540	BA.2	BA.2	BA.2_1	DCGC-496315|EPI_ISL_12317540|Denmark|BA.2|2022-04-19	99,81
EPI_ISL_12319745	BA.1.1	BA.1.1	BA.1.1_3	PHEC-YYRS9RE|EPI_ISL_12319745|United Kingdom|BA.1.1|2022-04-12	99,64
EPI_ISL_12322033	BA.2	A	A_14	IMR_CV04258|EPI_ISL_12322033|Malaysia|BA.2|2022-03-09	90,52
EPI_ISL_12322302	BA.2	A	A_14	IMR_WC191405|EPI_ISL_12322302|Malaysia|BA.2|2022-03-16	99,81
EPI_ISL_12324645	BA.1.15	BA.1	BA.1_1	TX-HHD-2203082111|EPI_ISL_12324645|USA|BA.1.15|2022-01-05	89,93

EPI_ISL_12324740	BA.1.15	BA.1	BA.1_1	TX-HHD-2203229919|EPI_ISL_12324740|USA|BA.1.15|2022-01-08	95,13
EPI_ISL_12326761	BA.1.1	BA.1	BA.1_1	TX-HHD-2203229034|EPI_ISL_12326761|USA|BA.1.1|2022-01-08	93,67
EPI_ISL_12328560	BA.2.10	A	A_14	CO-CDPHE-2103111652|EPI_ISL_12328560|USA|BA.2.10|2022-03-29	99,81
EPI_ISL_12330977	BA.2	A	A_17	OH-CDC-QDX35823375|EPI_ISL_12330977|USA|BA.2|2022-04-10	99,81
EPI_ISL_12333690	BA.2	BA.2	BA.2_1	BY-RKI-I-732683|EPI_ISL_12333690|Germany|BA.2|2022-04-12	99,81
EPI_ISL_12333882	BA.2	BA.2.31	BA.2.31	BY-RKI-I-732934|EPI_ISL_12333882|Germany|BA.2|2022-04-12	99,69
EPI_ISL_12334616	BA.2.9	BA.2.12	BA.2.12	NW-RKI-I-734263|EPI_ISL_12334616|Germany|BA.2.9|2022-04-15	95,21
EPI_ISL_12335075	BA.2	A	A_17	BY-RKI-I-734813|EPI_ISL_12335075|Germany|BA.2|2022-03-27	99,81
EPI_ISL_12335146	BA.2.9	A	A_17	BY-RKI-I-734886|EPI_ISL_12335146|Germany|BA.2.9|2022-03-30	99,81
EPI_ISL_12337178	BA.2	A	A_14	LSPA-3DAC3DA|EPI_ISL_12337178|United Kingdom|BA.2|2022-04-21	99,81
EPI_ISL_12338131	BA.2	A	A_14	QEUH-3D83157|EPI_ISL_12338131|United Kingdom|BA.2|2022-04-18	99,81
EPI_ISL_12339941	BA.2	A	A_4	ZH-ETHZ-37151537|EPI_ISL_12339941|Switzerland|BA.2|2022-04-11	99,41
EPI_ISL_12340120	BA.2	A	A_4	ZH-ETHZ-37150793|EPI_ISL_12340120|Switzerland|BA.2|2022-04-11	99,81
EPI_ISL_12340170	BA.2	BA.2	BA.2_1	SN-RKI-I-734920|EPI_ISL_12340170|Germany|BA.2|2022-03-28	99,60
EPI_ISL_12340518	BA.2.9	BA.2	BA.2_1	SL-RKI-I-735256|EPI_ISL_12340518|Germany|BA.2.9|2022-03-26	99,73
EPI_ISL_12340855	BA.1.1.1	BA.1.1	BA.1.1_2	SN-RKI-I-735619|EPI_ISL_12340855|Germany|BA.1.1.1|2022-03-29	99,60
EPI_ISL_12341285	BA.2	BA.2	BA.2_1	SN-RKI-I-735865|EPI_ISL_12341285|Germany|BA.2|2022-04-01	99,75
EPI_ISL_12342160	BA.2	A	A_17	DCGC-498006|EPI_ISL_12342160|Denmark|BA.2|2022-04-23	99,75
EPI_ISL_12342615	BA.2	BA.2	BA.2_1	DCGC-498225|EPI_ISL_12342615|Denmark|BA.2|2022-04-20	90,29
EPI_ISL_12343250	BA.2	A	A_17	DCGC-498445|EPI_ISL_12343250|Denmark|BA.2|2022-04-23	99,75
EPI_ISL_12343457	BA.2.9	A	A_17	BW-RKI-I-737904|EPI_ISL_12343457|Germany|BA.2.9|2022-04-18	99,81
EPI_ISL_12346336	BA.2	BA.2	BA.2_2	NW-RKI-I-739571|EPI_ISL_12346336|Germany|BA.2|2022-04-16	91,13
EPI_ISL_12347179	BA.2	A	A_17	DCGC-500076|EPI_ISL_12347179|Denmark|BA.2|2022-04-06	99,81
EPI_ISL_12347313	BA.2	A	A_14	PHEC-YYRZOKJ|EPI_ISL_12347313|United Kingdom|BA.2|2022	99,81
EPI_ISL_12349060	BA.2	BA.2	BA.2_1	PHEP-YYR4AHS|EPI_ISL_12349060|United Kingdom|BA.2|2022-03-30	99,77
EPI_ISL_12351266	BA.2	BA.2	BA.2_1	CL-27960|EPI_ISL_12351266|Spain|BA.2|2022-02-27	99,83
EPI_ISL_12351415	BA.2	A	A_14	CL-28872|EPI_ISL_12351415|Spain|BA.2|2022-04-17	91,09
EPI_ISL_12351690	BA.2.3	A	A_14	CL-27891|EPI_ISL_12351690|Spain|BA.2.3|2022-03-21	99,83
EPI_ISL_12352394	BA.2	BA.2	BA.2_1	NW-RKI-I-742533|EPI_ISL_12352394|Germany|BA.2|2022-04-04	98,13
EPI_ISL_12352505	BA.1.1	BA.1	BA.1_4	ANT-CWOHC-VG-SEC2893G|EPI_ISL_12352505|Colombia|BA.1.1|2022-01-03	79,53
EPI_ISL_12354441	BA.2	A	A_14	BW-RKI-I-744293|EPI_ISL_12354441|Germany|BA.2|2022-04-19	99,81
EPI_ISL_12355231	BA.2	BA.2	BA.2_1	BY-RKI-I-744983|EPI_ISL_12355231|Germany|BA.2|2022-04-11	99,68
EPI_ISL_12356107	BA.2.12	BA.2.12	BA.2.12	HB-RKI-I-745845|EPI_ISL_12356107|Germany|BA.2.12|2022-03-23	99,71
EPI_ISL_12358415	BA.2	A	A_17	BW-RKI-I-747922|EPI_ISL_12358415|Germany|BA.2|2022-04-12	99,07
EPI_ISL_12359638	BA.2	BA.2	BA.2_1	BW-RKI-I-748541|EPI_ISL_12359638|Germany|BA.2|2022-04-18	95,69
EPI_ISL_12359999	BA.2	A	A_17	BW-RKI-I-748940|EPI_ISL_12359999|Germany|BA.2|2022-04-18	99,81
EPI_ISL_12361964	BA.2	BA.2	BA.2_1	HH-RKI-I-750375|EPI_ISL_12361964|Germany|BA.2|2022-04-10	99,73
EPI_ISL_12364858	BA.2	BA.2	BA.2_1	HH-RKI-I-753350|EPI_ISL_12364858|Germany|BA.2|2022-03-31	99,75
EPI_ISL_12364987	BA.2	BA.2	BA.2_1	SN-RKI-I-753483|EPI_ISL_12364987|Germany|BA.2|2022-03-29	99,77
EPI_ISL_12365366	BA.1.1	BA.1.1	BA.1.1_2	VA-VTVAS3-GSC31674|EPI_ISL_12365366|USA|BA.1.1|2021-12-27	99,52
EPI_ISL_12365683	BA.1.1	BA.1.1	BA.1.1_3	NW-RKI-I-754195|EPI_ISL_12365683|Germany|BA.1.1|2022-01-27	99,52
EPI_ISL_12366107	BA.2	A	A_14	RP-RKI-I-754568|EPI_ISL_12366107|Germany|BA.2|2022-04-09	99,03
EPI_ISL_12368187	BA.2	BA.2	BA.2_1	NW-RKI-I-756921|EPI_ISL_12368187|Germany|BA.2|2022-03-09	99,73
EPI_ISL_12368306	BA.2.36	BA.2	BA.2_2	BW-RKI-I-757054|EPI_ISL_12368306|Germany|BA.2.36|2022-04-20	99,81
EPI_ISL_12368807	BA.2	A	A_14	NW-RKI-I-757576|EPI_ISL_12368807|Germany|BA.2|2022-04-11	99,81
EPI_ISL_12368932	BA.2.9	A	A_17	NW-RKI-I-757703|EPI_ISL_12368932|Germany|BA.2.9|2022-04-21	99,79
EPI_ISL_12370956	BA.2	BA.2	BA.2_1	ZNA_25346390|EPI_ISL_12370956|Belgium|BA.2|2022-04-14	99,03
EPI_ISL_12374766	BA.1.1	BA.2	BA.2_1	WA-S19618|EPI_ISL_12374766|USA|BA.1.1|2022-04-10	99,31
EPI_ISL_12375221	BA.1.1	BA.1.1	BA.1.1_1	NS-NML-399478|EPI_ISL_12375221|Canada|BA.1.1|2022-03-21	99,64
EPI_ISL_12379106	BA.1.15	BA.1.15	BA.1.15	BC-BCCDC-380999|EPI_ISL_12379106|Canada|BA.1.15|2022-02-28	99,64
EPI_ISL_12380051	BA.2	BA.2	BA.2_1	BC-BCCDC-385901|EPI_ISL_12380051|Canada|BA.2|2022-03-06	99,81
EPI_ISL_12380164	BA.1.1	BA.1.1	BA.1.1_2	BC-BCCDC-386034|EPI_ISL_12380164|Canada|BA.1.1|2022-03-05	99,64
EPI_ISL_12382151	BA.2	A	A_14	BC-BCCDC-395384|EPI_ISL_12382151|Canada|BA.2|2022-03-20	99,81
EPI_ISL_12383985	BA.2	BA.2	BA.2_1	BC-BCCDC-408114|EPI_ISL_12383985|Canada|BA.2|2022-04-03	99,81
EPI_ISL_12384071	BA.1.1	BA.1.1	BA.1.1_2	BC-BCCDC-408213|EPI_ISL_12384071|Canada|BA.1.1|2022-04-01	99,64
EPI_ISL_12385841	BA.2	A	A_14	TAS001629|EPI_ISL_12385841|Australia|BA.2|2022-04-07	99,81
EPI_ISL_12389462	BA.1.1	BA.1.1	BA.1.1_2	UN-276675|EPI_ISL_12389462|Canada|BA.1.1|2021-12-18	62,47
EPI_ISL_12389489	BA.1.1	BA.1.1	BA.1.1_3	UN-276950|EPI_ISL_12389489|Canada|BA.1.1|2021-12-20	99,64
EPI_ISL_12389518	BA.1	BA.1	BA.1_4	UN-280169|EPI_ISL_12389518|Canada|BA.1|2021-12-21	99,60
EPI_ISL_12389871	BA.1.15	BA.1	BA.1_4	UN-285599|EPI_ISL_12389871|Canada|BA.1.15|2021-12-28	79,62
EPI_ISL_12390120	BA.1.15.1	BA.1.15.1	BA.1.15.1	UN-295304|EPI_ISL_12390120|Canada|BA.1.15.1|2022-01-02	99,60
EPI_ISL_12390126	BA.1.1	BA.1.1	BA.1.1_3	UN-295310|EPI_ISL_12390126|Canada|BA.1.1|2022-01-02	99,64
EPI_ISL_12390948	BA.1.1	BA.1.1	BA.1.1_4	UN-346817|EPI_ISL_12390948|Canada|BA.1.1|2022-01-08	79,62
EPI_ISL_12391492	BA.2	A	A_14	VIC53068|EPI_ISL_12391492|Australia|BA.2|2022-04-10	99,81
EPI_ISL_12393079	BA.1.15	BA.1	BA.1_4	UN-285205|EPI_ISL_12393079|Canada|BA.1.15|2021-12-28	79,62
EPI_ISL_12393090	BA.1.1	BA.1.1	BA.1.1_2	UN-285218|EPI_ISL_12393090|Canada|BA.1.1|2021-12-27	99,64
EPI_ISL_12394092	BA.1.1	BA.1.1	BA.1.1_2	UN-319460|EPI_ISL_12394092|Canada|BA.1.1|2022-01-14	93,89
EPI_ISL_12396803	BA.2.36	BA.2	BA.2_2	LSPA-3DA9DA2|EPI_ISL_12396803|United Kingdom|BA.2.36|2022-04-21	99,81
EPI_ISL_12399780	BA.2	A	A_17	DCGC-500444|EPI_ISL_12399780|Denmark|BA.2|2022-04-19	99,96
EPI_ISL_12400774	BA.2.9	A	A_17	UMTM471554|EPI_ISL_12400774|Czech Republic|BA.2.9|2022-03-26	95,99
EPI_ISL_12400825	BA.2.9	A	A_17	UMTM475247|EPI_ISL_12400825|Czech Republic|BA.2.9|2022-04-05	97,86
EPI_ISL_12401913	BA.2	A	A_17	DCGC-502062|EPI_ISL_12401913|Denmark|BA.2|2022-04-19	99,98
EPI_ISL_12403400	BA.2.22	BA.2.22	BA.2.22	PHEC-YYR3RWH|EPI_ISL_12403400|United Kingdom|BA.2.22|2022-03-29	99,77
EPI_ISL_12406089	BA.1.18	BA.1.15	BA.1.15	KA-NIMH-4687|EPI_ISL_12406089|India|BA.1.18|2022-01-10	91,51
EPI_ISL_12407225	BA.2	A	A_17	OCC-CHU-TLS-6002908238|EPI_ISL_12407225|France|BA.2|2022-04-19	70,50
EPI_ISL_12407238	BA.1.1	BA.1.1	BA.1.1_2	JAM-UWIMicro-0636|EPI_ISL_12407238|Jamaica|BA.1.1|2022-01-08	96,70
EPI_ISL_12408244	BA.2.3	A	A_14	WA-UW-22040266303|EPI_ISL_12408244|USA|BA.2.3|2022-04-02	92,44
EPI_ISL_12409746	BA.2.9	BA.1	BA.1_4	22-551127|EPI_ISL_12409746|Sweden|BA.2.9|2022-04-04	96,34
EPI_ISL_12410350	BA.2	BA.1	BA.1_4	DA10333641|EPI_ISL_12410350|Sweden|BA.2|2022-03-01	95,94
EPI_ISL_12412712	BA.2	BA.2	BA.2_1	PDL-HMN-22042111160|EPI_ISL_12412712|France|BA.2|2022-03-14	97,61
EPI_ISL_12413285	BA.2	BA.2	BA.2_1	SCOT-10492|EPI_ISL_12413285|United Kingdom|BA.2|2022-04-06	99,81
EPI_ISL_12414068	BA.2.3	A	A_14	KDCA38421|EPI_ISL_12414068|South Korea|BA.2.3|2022-04-01	99,81
EPI_ISL_12414195	BA.2.3	A	A_14	KDCA38558|EPI_ISL_12414195|South Korea|BA.2.3|2022-04-04	99,81
EPI_ISL_12414246	BA.2	BA.2	BA.2_1	KDCA38623|EPI_ISL_12414246|South Korea|BA.2|2022-04-05	99,77
EPI_ISL_12416866	BA.2	BA.2	BA.2_1	MBLG-CTMAOT04191440|EPI_ISL_12416866|Belgium|BA.2|2022-04-17	96,78
EPI_ISL_12418303	BA.2.2	BA.2.2	BA.2.2	HK-HKPU-PU22MM012083|EPI_ISL_12418303|Hong Kong|BA.2.2|2022-02-05	99,81
EPI_ISL_12418630	BA.2.2	BA.2.2	BA.2.2	HK-HKPU-PU22MC626060|EPI_ISL_12418630|Hong Kong|BA.2.2|2022-02-09	99,81
EPI_ISL_12420384	BA.2	BA.2	BA.2_1	NC-ECU-113082799|EPI_ISL_12420384|USA|BA.2|2022-04-20	99,79
EPI_ISL_12422473	BA.2	A	A_14	NY-MSHSPSP-PV57588|EPI_ISL_12422473|USA|BA.2|2022-04-09	99,81
EPI_ISL_12422710	BA.1.15	BA.1.15	BA.1.15	SP-NVBS15549GENOV828882585688|EPI_ISL_12422710|Brazil|BA.1.15|2022-03-02	99,64
EPI_ISL_12423570	BA.2	A	A_14	AB-ABPHL-61808|EPI_ISL_12423570|Canada|BA.2|2022-03-23	70,50
EPI_ISL_12426816	BA.1	BA.1.15	BA.1.15	NY-CUIMC-NP-8967|EPI_ISL_12426816|USA|BA.1|2021-12-28	99,64
EPI_ISL_12427628	BA.2	BA.2	BA.2_1	WI-UW-10360|EPI_ISL_12427628|USA|BA.2|2022-04-04	90,52
EPI_ISL_12428321	BA.1.1.18	BA.1.1	BA.1.1_2	AZ-ASU64414|EPI_ISL_12428321|USA|BA.1.1.18|2022-01-05	89,32
EPI_ISL_12428865	BA.2	BA.2	BA.2_1	LSPA-3DB7BA7|EPI_ISL_12428865|United Kingdom|BA.2|2022-04-22	99,81
EPI_ISL_12428912	BA.2	BA.2	BA.2_1	LSPA-3DB8EBC|EPI_ISL_12428912|United Kingdom|BA.2|2022-04-20	99,81
EPI_ISL_12429440	BA.2	BA.2	BA.2_1	AZ-ASU65791|EPI_ISL_12429440|USA|BA.2|2022-04-25	94,70
EPI_ISL_12429933	BA.2	A	A_14	OR-OHSU-221090575|EPI_ISL_12429933|USA|BA.2|2022-04-05	99,52
EPI_ISL_12431141	BA.2	BA.2	BA.2_1	WI-MHDL-2022040303|EPI_ISL_12431141|USA|BA.2|2022-04-11	97,04
EPI_ISL_12432669	BA.1.1	BA.1.1	BA.1.1_2	AZ-ASU65472|EPI_ISL_12432669|USA|BA.1.1|2022-01-03	99,64
EPI_ISL_12433641	BA.2	BA.2	BA.2_1	WA-PHL-017333|EPI_ISL_12433641|USA|BA.2|2022-03-22	92,60

EPI_ISL_12434246	BA.2	A	A_14	ACT4872|EPI_ISL_12434246|Australia|BA.2|2022-04-03	99,81
EPI_ISL_12434319	BA.2.3	A	A_14	ACT4977|EPI_ISL_12434319|Australia|BA.2.3|2022-04-07	99,81
EPI_ISL_12435595	BA.2	A	A_17	DCGC-503354|EPI_ISL_12435595|Denmark|BA.2|2022-04-25	99,81
EPI_ISL_12436780	BA.1.1.18	BA.1.1	BA.1.1_2	CO-CDPHE-2103139878|EPI_ISL_12436780|USA|BA.1.1.18|2022-01-16	90,98
EPI_ISL_12437025	BA.1.1.18	BA.1.1	BA.1.1_3	CO-CDPHE-2103145010|EPI_ISL_12437025|USA|BA.1.1.18|2022-01-04	99,64
EPI_ISL_12437157	BA.1.1	BA.1.1	BA.1.1_3	CO-CDPHE-2103140650|EPI_ISL_12437157|USA|BA.1.1|2022-01-19	99,64
EPI_ISL_12438143	BA.2	BA.2	BA.2_1	IDF-LBZCentre-YW2204190059|EPI_ISL_12438143|France|BA.2|2022-04-19	99,81
EPI_ISL_12442410	BA.2	BA.2.12.1	BA.2.12.1	ARA-CERBAHC-04197737|EPI_ISL_12442410|France|BA.2|2022-04-11	89,49
EPI_ISL_12442714	BA.2.10.1	BA.2.10.1	BA.2.10.1	SA225508|EPI_ISL_12442714|Australia|BA.2.10.1|2022-04-11	99,81
EPI_ISL_12442932	BA.2.12	A	A_4	SA232459|EPI_ISL_12442932|Australia|BA.2.12|2022-04-13	99,81
EPI_ISL_12443692	BA.1.1.1	BA.1.15	BA.1.15	LNS1034053|EPI_ISL_12443692|Luxembourg|BA.1.1.1|2022-03-01	77,73
EPI_ISL_12444597	BA.2	BA.2	BA.2_1	IL-S22WGS1435|EPI_ISL_12444597|USA|BA.2|2022-04-11	99,81
EPI_ISL_12444612	BA.2	BA.2	BA.2_1	IL-S22WGS1488|EPI_ISL_12444612|USA|BA.2|2022-04-16	99,75
EPI_ISL_12445962	BA.2.19	A	A_4	LNS5097413|EPI_ISL_12445962|Luxembourg|BA.2.19|2022-03-01	99,81
EPI_ISL_12446034	BA.2	A	A_14	LNS2769576|EPI_ISL_12446034|Luxembourg|BA.2|2022-03-15	99,81
EPI_ISL_12447404	BA.2	A	A_14	LNS0897360|EPI_ISL_12447404|Luxembourg|BA.2|2022-02-07	99,79
EPI_ISL_12447995	BA.2	A	A_17	LNS9935472|EPI_ISL_12447995|Luxembourg|BA.2|2022-04-11	99,81
EPI_ISL_12448930	BA.1.17	BA.1	BA.1_2	LNS1276322|EPI_ISL_12448930|Luxembourg|BA.1.17|2022-02-15	92,81
EPI_ISL_12449822	BA.2	A	A_17	18835|EPI_ISL_12449822|Norway|BA.2|2022-04-04	99,81
EPI_ISL_12450089	BA.2	BA.2	BA.2_1	35381|EPI_ISL_12450089|Croatia|BA.2|2022-03-30	98,51
EPI_ISL_12452522	BA.2	A	A_17	LNS9352667|EPI_ISL_12452522|Luxembourg|BA.2|2022-02-25	99,81
EPI_ISL_12453304	BA.2.44	BA.2.44	BA.2.44	LNS1325481|EPI_ISL_12453304|Luxembourg|BA.2.44|2022-03-28	99,81
EPI_ISL_12453474	BA.2	A	A_17	ARA-CFD220023717101|EPI_ISL_12453474|France|BA.2|2022-04-24	99,81
EPI_ISL_12456068	BA.1.1	BA.1.1	BA.1.1_2	MI-UM-10043444565|EPI_ISL_12456068|USA|BA.1.1|2022-01-05	99,64
EPI_ISL_12456798	BA.2	A	A_14	ICH-741221163|EPI_ISL_12456798|Israel|BA.2|2022-04-24	96,58
EPI_ISL_12457275	BA.2.5	A	A_4	Orebro-20821530|EPI_ISL_12457275|Sweden|BA.2.5|2022-04-17	90,52
EPI_ISL_12462223	BA.2.12.1	BA.2.12.1	BA.2.12.1	NY-PRL-0425_01E24|EPI_ISL_12462223|USA|BA.2.12.1|2022-04-24	99,81
EPI_ISL_12462528	BA.2.3	A	A_14	NY-PRL-0425_02J02|EPI_ISL_12462528|USA|BA.2.3|2022-04-21	99,81
EPI_ISL_12465437	BA.2	BA.2.32	BA.2.32	GES-IPP28474|EPI_ISL_12465437|France|BA.2|2022-04-04	99,81
EPI_ISL_12469157	BA.2	BA.2	BA.2_1	G-NVRL-S22IRL00278854|EPI_ISL_12469157|Ireland|BA.2|2022-03-29	90,52
EPI_ISL_12472610	BA.2	BA.2	BA.2_1	PDL-LBZCentre-AQ2204190055|EPI_ISL_12472610|France|BA.2|2022-04-19	99,79
EPI_ISL_12473163	BA.2.9	A	A_17	DCGC-504845|EPI_ISL_12473163|Denmark|BA.2.9|2022-04-26	99,81
EPI_ISL_12474754	BA.1.1	BA.1.1	BA.1.1_2	VER_IBT_IMSS_7155|EPI_ISL_12474754|Mexico|BA.1.1|2022-02-08	99,64
EPI_ISL_12475504	BA.2	A	A_14	MR-LUHS-42-07|EPI_ISL_12475504|Lithuania|BA.2|2022-04-19	90,52
EPI_ISL_12476349	BA.2	A	A_14	NH-NMDL-01645|EPI_ISL_12476349|Netherlands|BA.2|2022-04-13	98,51
EPI_ISL_12477742	BA.2	A	A_4	LSPA-3DD511A|EPI_ISL_12477742|United Kingdom|BA.2|2022-04-27	99,81
EPI_ISL_12478503	BA.2	A	A_17	QEUH-3DCDB17|EPI_ISL_12478503|United Kingdom|BA.2|2022-04-26	99,81
EPI_ISL_12478908	BA.2	A	A_14	QEUH-3DD09DB|EPI_ISL_12478908|United Kingdom|BA.2|2022-04-26	99,81
EPI_ISL_12479372	BA.2	A	A_14	QEUH-3DBCEB8|EPI_ISL_12479372|United Kingdom|BA.2|2022-04-24	99,81
EPI_ISL_12480002	BA.2	A	A_14	QEUH-3DBB420|EPI_ISL_12480002|United Kingdom|BA.2|2022-04-24	99,81
EPI_ISL_12480739	BA.2.9	A	A_17	QEUH-3DC44E8|EPI_ISL_12480739|United Kingdom|BA.2.9|2022-04-25	99,81
EPI_ISL_12485425	BA.2.12.1	BA.2.12.1	BA.2.12.1	CA-CDPH-500056827|EPI_ISL_12485425|USA|BA.2.12.1|2022-04-07	99,81
EPI_ISL_12487820	BA.2.12.1	BA.2.12.1	BA.2.12.1	MA-Broad-CRSP_DKSFCSWP2A2QC6NK|EPI_ISL_12487820|USA|BA.2.12.1|2022-04-14	96,28
EPI_ISL_12488098	BA.2.12.1	BA.2.12.1	BA.2.12.1	MA-Broad-CRSP_IUYMLGOPILU3PSI5|EPI_ISL_12488098|USA|BA.2.12.1|2022-04-16	94,26
EPI_ISL_12488528	BA.2.7	BA.2	BA.2_1	MA-Broad-CRSP_QEFQOZXUYLZADKJC|EPI_ISL_12488528|USA|BA.2.7|2022-04-15	88,88
EPI_ISL_12489283	BA.1	BA.1.15	BA.1.15	VT-Broad-CRSP_2XTIPV2PG6R7CBEZ|EPI_ISL_12489283|USA|BA.1|2022-04-11	99,64
EPI_ISL_12489406	BA.2.12.1	BA.2.12.1	BA.2.12.1	NY-Broad-CRSP_5M6S3TUIQBVXTYUF|EPI_ISL_12489406|USA|BA.2.12.1|2022-04-18	94,45
EPI_ISL_12490064	BA.2	BA.2	BA.2_1	LSPA-3DE039E|EPI_ISL_12490064|United Kingdom|BA.2|2022-04-27	99,81
EPI_ISL_12490335	BA.2	A	A_14	QEUH-3DD5CEC|EPI_ISL_12490335|United Kingdom|BA.2|2022-04-27	99,81
EPI_ISL_12493088	BA.2.36	BA.2	BA.2_2	CHUNamur14134503|EPI_ISL_12493088|Belgium|BA.2.36|2022-04-21	99,81
EPI_ISL_12495416	BA.2	BA.2.12	BA.2.12	PDL-HMN-22042150265|EPI_ISL_12495416|France|BA.2|2022-04-11	90,61
EPI_ISL_12496191	BA.1.1	BA.1.1	BA.1.1_2	TX-DSHS-19411|EPI_ISL_12496191|USA|BA.1.1|2021-12-30	82,18
EPI_ISL_12496723	BA.2	A	A_14	NC-CDC-LC0581515|EPI_ISL_12496723|USA|BA.2|2022-04-13	99,81
EPI_ISL_12496888	BA.2	BA.2	BA.2_1	WA-CDC-LC0582201|EPI_ISL_12496888|USA|BA.2|2022-04-14	99,81
EPI_ISL_12496891	BA.2	A	A_14	WA-CDC-LC0582203|EPI_ISL_12496891|USA|BA.2|2022-04-14	99,81
EPI_ISL_12498274	BA.2.12.1	BA.2.12.1	BA.2.12.1	CO-CDC-MMB14991675|EPI_ISL_12498274|USA|BA.2.12.1|2022-04-12	99,81
EPI_ISL_12499508	BA.2	A	A_14	WA-CDC-QDX35917463|EPI_ISL_12499508|USA|BA.2|2022-04-12	99,81
EPI_ISL_12501940	BA.2.3.17	A	A_14	CO-CDC-MMB15087628|EPI_ISL_12501940|USA|BA.2.3.17|2022-04-22	99,81
EPI_ISL_12502910	BA.2.3	A	A_14	WI-CDC-QDX36018883|EPI_ISL_12502910|USA|BA.2.3|2022-04-14	99,81
EPI_ISL_12503618	BA.2	BA.2	BA.2_1	DC-LSPSDS-VG-212710|EPI_ISL_12503618|Colombia|BA.2|2022-03-14	74,72
EPI_ISL_12505011	BA.2	A	A_15	NY-CDC-LC0584541|EPI_ISL_12505011|USA|BA.2|2022-04-15	99,81
EPI_ISL_12505510	BA.2.3	A	A_14	MI-CDC-LC0589003|EPI_ISL_12505510|USA|BA.2.3|2022-04-17	98,42
EPI_ISL_12506116	BA.2.12.1	BA.2.12.1	BA.2.12.1	NC-CDC-LC0586704|EPI_ISL_12506116|USA|BA.2.12.1|2022-04-18	99,81
EPI_ISL_12506892	BA.2	A	A_14	VA-CDC-LC0587303|EPI_ISL_12506892|USA|BA.2|2022-04-20	99,81
EPI_ISL_12507150	BA.2	A	A_14	NY-CDC-LC0588091|EPI_ISL_12507150|USA|BA.2|2022-04-20	99,81
EPI_ISL_12507745	BA.2	A	A_14	FL-CDC-STM-SU6NUMH32|EPI_ISL_12507745|USA|BA.2|2022-04-16	99,79
EPI_ISL_12507838	BA.2	BA.2	BA.2_1	CA-CDC-STM-CH22VRK3K|EPI_ISL_12507838|USA|BA.2|2022-04-18	99,79
EPI_ISL_12507880	BA.2.9	A	A_17	FL-CDC-STM-VCXKW4WF3|EPI_ISL_12507880|USA|BA.2.9|2022-04-18	99,81
EPI_ISL_12508055	BA.2.10	A	A_14	CA-CDC-STM-6P9EXG5P2|EPI_ISL_12508055|USA|BA.2.10|2022-04-18	99,79
EPI_ISL_12508322	BA.1.15	BA.1	BA.1_4	WV-VTVAS3-GSC31767|EPI_ISL_12508322|USA|BA.1.15|2022-01-08	96,13
EPI_ISL_12508627	BA.2	BA.2	BA.2_1	MA-Broad-CRSP_3BN53IXWNUAWMSV7|EPI_ISL_12508627|USA|BA.2|2022-04-21	95,17
EPI_ISL_12508832	BA.2	BA.2	BA.2_1	MA-Broad-CRSP_AMCDQ43SDGZBOMCY|EPI_ISL_12508832|USA|BA.2|2022-04-24	95,80
EPI_ISL_12508931	BA.2.12.1	BA.2.12.1	BA.2.12.1	MA-Broad-CRSP_CXX2C3ZZWSZJM2QB|EPI_ISL_12508931|USA|BA.2.12.1|2022-04-20	97,46
EPI_ISL_12509396	BA.2.12.1	BA.2.12.1	BA.2.12.1	MA-Broad-CRSP_QJ3ALLCU6JTAM7OG|EPI_ISL_12509396|USA|BA.2.12.1|2022-04-21	95,34
EPI_ISL_12510723	BA.2	BA.2.12.1	BA.2.12.1	WA-UW-22041070917|EPI_ISL_12510723|USA|BA.2|2022-04-10	90,27
EPI_ISL_12513538	BA.2	A	A_17	BB-RKI-I-758975|EPI_ISL_12513538|Germany|BA.2|2022-04-25	99,81
EPI_ISL_12513781	BA.2.9	BA.2	BA.2_1	NW-RKI-I-760249|EPI_ISL_12513781|Germany|BA.2.9|2022-04-21	95,15
EPI_ISL_12514600	BA.5.2.1	A	A_3	LSPA-3DE32CB|EPI_ISL_12514600|United Kingdom|BA.5.2.1|2022-04-29	99,69
EPI_ISL_12515953	BA.2	A	A_17	BW-RKI-I-761851|EPI_ISL_12515953|Germany|BA.2|2022-04-21	99,81
EPI_ISL_12518593	BA.2	BA.2	BA.2_1	SH-RKI-I-764323|EPI_ISL_12518593|Germany|BA.2|2022-04-19	99,81
EPI_ISL_12521596	BA.1.1	BA.1.1	BA.1.1_2	NW-RKI-I-767264|EPI_ISL_12521596|Germany|BA.1.1|2022-02-02	99,54
EPI_ISL_12521797	BA.1.1	BA.1.1	BA.1.1_3	NW-RKI-I-767472|EPI_ISL_12521797|Germany|BA.1.1|2022-02-02	99,31
EPI_ISL_12522355	BA.2	BA.2	BA.2_1	SL-RKI-I-768079|EPI_ISL_12522355|Germany|BA.2|2022-04-09	99,73
EPI_ISL_12524181	BA.2	BA.2	BA.2_1	SL-RKI-I-770697|EPI_ISL_12524181|Germany|BA.2|2022-03-28	99,75
EPI_ISL_12525006	BA.2	BA.2	BA.2_1	HE-RKI-I-771579|EPI_ISL_12525006|Germany|BA.2|2022-04-04	99,77
EPI_ISL_12525300	BA.2.8	BA.2	BA.2_1	SL-RKI-I-771902|EPI_ISL_12525300|Germany|BA.2.8|2022-04-06	99,77
EPI_ISL_12526874	BA.2.9	A	A_17	BB-RKI-I-773426|EPI_ISL_12526874|Germany|BA.2.9|2022-04-06	97,63
EPI_ISL_12526968	BA.2.9	A	A_17	BW-RKI-I-773523|EPI_ISL_12526968|Germany|BA.2.9|2022-04-28	99,81
EPI_ISL_12527529	BA.2	BA.2	BA.2_1	SN-RKI-I-774119|EPI_ISL_12527529|Germany|BA.2|2022-04-15	99,73
EPI_ISL_12530004	BA.2	BA.2	BA.2_1	PHEC-YYR6SCT|EPI_ISL_12530004|United Kingdom|BA.2|2022-04-13	96,78
EPI_ISL_12531299	BA.2.12.1	BA.2.12.1	BA.2.12.1	IN-CDC-LC0591144|EPI_ISL_12531299|USA|BA.2.12.1|2022-04-21	99,81
EPI_ISL_12532430	BA.2.12.1	BA.2.12.1	BA.2.12.1	KY-CDC-LC0591881|EPI_ISL_12532430|USA|BA.2.12.1|2022-04-25	99,81
EPI_ISL_12532479	BA.2.12.1	BA.2.12.1	BA.2.12.1	MI-CDC-LC0591377|EPI_ISL_12532479|USA|BA.2.12.1|2022-04-25	99,81
EPI_ISL_12532714	BA.2	BA.2.12.1	BA.2.12.1	BB_22_00001421|EPI_ISL_12532714|Slovakia|BA.2|2022-04-20	86,45
EPI_ISL_12533923	BA.2.9	A	A_17	DCGC-506053|EPI_ISL_12533923|Denmark|BA.2.9|2022-04-21	99,29
EPI_ISL_12534025	BA.2	A	A_17	DCGC-506157|EPI_ISL_12534025|Denmark|BA.2|2022-04-21	99,81
EPI_ISL_12535243	BA.2	BA.2	BA.2_1	CVL-8005204|EPI_ISL_12535243|Israel|BA.2|2022-04-01	93,47
EPI_ISL_12535378	BA.2	A	A_4	CVL-9003689|EPI_ISL_12535378|Israel|BA.2|2022-04-20	99,81
EPI_ISL_12535722	BA.2.9	BA.2	BA.2_1	CVL-9003253|EPI_ISL_12535722|Israel|BA.2.9|2022-04-07	91,20
EPI_ISL_12536958	BA.2	A	A_17	ZE-MVD-CWGS2201925|EPI_ISL_12536958|Netherlands|BA.2|2022-04-16	99,60
EPI_ISL_12537921	BA.2.9	A	A_17	MD-IGS-062210301065A|EPI_ISL_12537921|USA|BA.2.9|2022-04-13	99,81

EPI_ISL_12541170	BA.2.10	A	A_14	CA-CDC-QDX36065689|EPI_ISL_12541170|USA|BA.2.10|2022-04-16	99,81
EPI_ISL_12541182	BA.2.3.10	BA.2	BA.2_1	VA-CDC-QDX36065061|EPI_ISL_12541182|USA|BA.2.3.10|2022-04-16	99,81
EPI_ISL_12544061	BA.1.1	BA.1.1	BA.1.1_2	AZ-TG1321687|EPI_ISL_12544061|USA|BA.1.1|2022-01-20	99,62
EPI_ISL_12544729	BA.1	BA.1.15	BA.1.15	SC-FIOCRUZ-84015CE|EPI_ISL_12544729|Brazil|BA.1|2022-01-19	99,64
EPI_ISL_12545041	BA.2	A	A_17	22CH3408|EPI_ISL_12545041|New Zealand|BA.2|2022-04-29	90,52
EPI_ISL_12545161	BA.2.10	BA.2	BA.2_1	22CH2550|EPI_ISL_12545161|New Zealand|BA.2.10|2022-04-04	85,79
EPI_ISL_12546993	BA.2	BA.2	BA.2_1	QEUH-3DE54F0|EPI_ISL_12546993|United Kingdom|BA.2|2022-04-29	99,81
EPI_ISL_12547538	BA.2	A	A_14	QEUH-3DE7CC9|EPI_ISL_12547538|United Kingdom|BA.2|2022-04-29	99,81
EPI_ISL_12548460	BA.2	BA.2	BA.2_1	QEUH-3DC09ED|EPI_ISL_12548460|United Kingdom|BA.2|2022-04-25	99,81
EPI_ISL_12552989	BA.2	A	A_14	QEUH-3D64444|EPI_ISL_12552989|United Kingdom|BA.2|2022-04-13	99,81
EPI_ISL_12554923	BA.2	A	A_17	QEUH-3D34CAF|EPI_ISL_12554923|United Kingdom|BA.2|2022-04-08	99,73
EPI_ISL_12556070	BA.2	A	A_17	QEUH-3D21D93|EPI_ISL_12556070|United Kingdom|BA.2|2022-04-05	99,81
EPI_ISL_12557047	BA.2	A	A_17	DCGC-506943|EPI_ISL_12557047|Denmark|BA.2|2022-05-01	99,81
EPI_ISL_12557054	BA.2	A	A_17	DCGC-506950|EPI_ISL_12557054|Denmark|BA.2|2022-05-01	99,81
EPI_ISL_12558573	BA.2	A	A_17	DCGC-507907|EPI_ISL_12558573|Denmark|BA.2|2022-04-27	99,79
EPI_ISL_12558954	BA.2	BA.2	BA.2_1	DCGC-508168|EPI_ISL_12558954|Denmark|BA.2|2022-04-28	99,98
EPI_ISL_12559042	BA.2.1	A	A_14	EDB57854|EPI_ISL_12559042|United Kingdom|BA.2.1|2022-04-09	99,81
EPI_ISL_12562346	BA.2	BA.2.12.1	BA.2.12.1	BB_22_00001526|EPI_ISL_12562346|Slovakia|BA.2|2022-04-28	90,80
EPI_ISL_12562593	BA.2.9	BA.1	BA.1_4	PEP396424|EPI_ISL_12562593|Sweden|BA.2.9|2022-04-19	96,34
EPI_ISL_12562891	BA.2	A	A_14	PHWC-PJ3I6A|EPI_ISL_12562891|United Kingdom|BA.2|2022-04-14	94,85
EPI_ISL_12563355	BA.2	A	A_14	PHWC-PJ4FN9|EPI_ISL_12563355|United Kingdom|BA.2|2022-04-17	99,81
EPI_ISL_12566650	BA.1.18	BA.1	BA.1_3	NW-HHU-24927|EPI_ISL_12566650|Germany|BA.1.18|2022-04-25	99,64
EPI_ISL_12566837	BA.1.14	BA.1	BA.1_1	ND-NDDH-14548|EPI_ISL_12566837|USA|BA.1.14|2022-01-17	93,44
EPI_ISL_12567778	BA.2	A	A_14	ND-NDDH-14532|EPI_ISL_12567778|USA|BA.2|2022-04-28	90,52
EPI_ISL_12568209	BA.2	A	A_14	FL-TGH-3160|EPI_ISL_12568209|USA|BA.2|2022-04-15	96,89
EPI_ISL_12569372	BA.2.9	A	A_17	UT-UPHL-220505031801|EPI_ISL_12569372|USA|BA.2.9|2022-04-15	99,81
EPI_ISL_12569682	BA.1.1	BA.1.1	BA.1.1_2	UT-UPHL-220505340589|EPI_ISL_12569682|USA|BA.1.1|2022-01-20	99,64
EPI_ISL_12570237	BA.2.23	A	A_14	LSPA-3DEDCF0|EPI_ISL_12570237|United Kingdom|BA.2.23|2022-04-30	99,81
EPI_ISL_12570288	BA.2	BA.2	BA.2_1	LSPA-3DECFE9|EPI_ISL_12570288|United Kingdom|BA.2|2022-04-29	94,43
EPI_ISL_12570575	BA.2	BA.2	BA.2_1	QEUH-3DEBEED|EPI_ISL_12570575|United Kingdom|BA.2|2022-04-30	97,96
EPI_ISL_12572867	BA.1.1	BA.1.1	BA.1.1_3	CV5001|EPI_ISL_12572867|Nigeria|BA.1.1|2021-12-19	95,08
EPI_ISL_12574464	BA.2	A	A_14	GA-CHUVI-34680791|EPI_ISL_12574464|Spain|BA.2|2022-03-30	99,81
EPI_ISL_12574886	BA.2	BA.2.10	BA.2.10	DC-Curative-237690|EPI_ISL_12574886|USA|BA.2|2022-04-26	95,38
EPI_ISL_12575342	BA.2.12.1	BA.2.12.1	BA.2.12.1	CO-CDPHE-2103147187|EPI_ISL_12575342|USA|BA.2.12.1|2022-04-14	88,97
EPI_ISL_12575542	BA.1.1.18	BA.1.1	BA.1.1_3	CO-CDPHE-2103139747|EPI_ISL_12575542|USA|BA.1.1.18|2022-01-03	99,64
EPI_ISL_12575956	BA.1	BA.1	BA.1_1	22SNR2599_wsserze|EPI_ISL_12575956|Poland|BA.1|2022-02-04	93,99
EPI_ISL_12578693	BA.2.3.14	A	A_14	IC-8696|EPI_ISL_12578693|Japan|BA.2.3.14|2022-04-17	99,81
EPI_ISL_12578946	BA.2	A	A_17	DCGC-508248|EPI_ISL_12578946|Denmark|BA.2|2022-04-01	99,81
EPI_ISL_12580130	BA.2	BA.2	BA.2_1	NW-HHU-25536|EPI_ISL_12580130|Germany|BA.2|2022-04-28	94,26
EPI_ISL_12582135	BA.2.9	BA.2	BA.2_1	PHEC-YYFBJSD|EPI_ISL_12582135|United Kingdom|BA.2.9|2022-04-21	94,18
EPI_ISL_12582867	BA.2.3	A	A_14	PHEC-YYFBWSA|EPI_ISL_12582867|United Kingdom|BA.2.3|2022-04-20	99,81
EPI_ISL_12583098	BA.2.3	BA.2	BA.2_1	PHEC-YYFBYSC|EPI_ISL_12583098|United Kingdom|BA.2.3|2022-04-23	99,79
EPI_ISL_12583540	BA.2.23.1	A	A_4	PHEC-YYFYIXE|EPI_ISL_12583540|United Kingdom|BA.2.23.1|2022-04-14	99,79
EPI_ISL_12584622	BA.2	BA.2	BA.2_1	CO-NVRL-ecS22IRL00267334|EPI_ISL_12584622|Ireland|BA.2|2022-03-22	96,53
EPI_ISL_12584967	BA.2	BA.2	BA.2_1	D-NVRL-ecS22IRL00263581|EPI_ISL_12584967|Ireland|BA.2|2022-03-21	96,53
EPI_ISL_12587404	BA.4	A	A_3	SU-NHLS_4745|EPI_ISL_12587404|South Africa|BA.4|2022-04-27	70,35
EPI_ISL_12589109	BA.2.12.1	BA.2.12.1	BA.2.12.1	NY-PRL-0502_02F07|EPI_ISL_12589109|USA|BA.2.12.1|2022-04-28	99,81
EPI_ISL_12589189	BA.2	BA.2	BA.2_1	NY-PRL-0502_02J12|EPI_ISL_12589189|USA|BA.2|2022-04-28	99,69
EPI_ISL_12591213	BA.2.12.1	BA.2.12.1	BA.2.12.1	IN-LH000216087|EPI_ISL_12591213|USA|BA.2.12.1|2022-04-26	99,81
EPI_ISL_12592400	BA.1	BA.1.15	BA.1.15	WY-WYPHL-22008162|EPI_ISL_12592400|USA|BA.1|2022-01-03	99,50
EPI_ISL_12592420	BA.2	A	A_4	NV-CDC-QDX35974988|EPI_ISL_12592420|USA|BA.2|2022-04-15	99,81
EPI_ISL_12594584	BA.2	BA.2	BA.2_1	NJ-CDC-LC0592886|EPI_ISL_12594584|USA|BA.2|2022-04-23	99,81
EPI_ISL_12595940	BA.2.12.1	BA.2.12.1	BA.2.12.1	NY-CDC-QDX36112132|EPI_ISL_12595940|USA|BA.2.12.1|2022-04-18	99,81
EPI_ISL_12597793	BA.2.3	AY.53	AY.53	MB-CPL-414337|EPI_ISL_12597793|Canada|BA.2.3|2022-04-15	79,79
EPI_ISL_12599321	BA.2	A	A_14	MD-HP30744-PIDPTSQHYA|EPI_ISL_12599321|USA|BA.2|2022-04-13	99,81
EPI_ISL_12599608	BA.2.12.1	BA.2.12.1	BA.2.12.1	AB-ABPHL-62385|EPI_ISL_12599608|Canada|BA.2.12.1|2022-04-02	90,52
EPI_ISL_12599823	BA.2.3	AY.53	AY.53	AB-ABPHL-62630|EPI_ISL_12599823|Canada|BA.2.3|2022-04-05	72,01
EPI_ISL_12600008	BA.1.1.16	BA.1.1	BA.1.1_2	AB-ABPHL-62861|EPI_ISL_12600008|Canada|BA.1.1.16|2022-03-30	99,64
EPI_ISL_12600023	BA.2.3	A	A_4	AB-ABPHL-62880|EPI_ISL_12600023|Canada|BA.2.3|2022-03-30	90,52
EPI_ISL_12600384	BA.2	BA.2	BA.2_1	AB-ABPHL-63310|EPI_ISL_12600384|Canada|BA.2|2022-04-07	90,52
EPI_ISL_12601560	BA.2	BA.2.61	BA.2.61	CA-LACPHL-AF09164|EPI_ISL_12601560|USA|BA.2|2022-04-22	90,52
EPI_ISL_12603240	BA.2.3.16	A	A_1	MS-15719|EPI_ISL_12603240|Romania|BA.2.3.16|2022-03-06	60,96
EPI_ISL_12603337	BA.2	BA.2	BA.2_1	B-16432|EPI_ISL_12603337|Romania|BA.2|2022-03-17	99,81
EPI_ISL_12604070	BA.1.1.2	BA.1.1	BA.1.1_3	SZ-NIG-Y214174|EPI_ISL_12604070|Japan|BA.1.1.2|2022-03-22	99,64
EPI_ISL_12605574	BA.2	BA.2	BA.2_1	FL-CDC-QDX36065499|EPI_ISL_12605574|USA|BA.2|2022-04-17	99,75
EPI_ISL_12605629	BA.2	A	A_14	CA-CDC-QDX36065411|EPI_ISL_12605629|USA|BA.2|2022-04-18	97,18
EPI_ISL_12606229	BA.2.41	BA.2.41	BA.2.41	LSPA-3DFAEEB|EPI_ISL_12606229|United Kingdom|BA.2.41|2022-05-03	99,79
EPI_ISL_12606693	BA.2	BA.2	BA.2_1	LSPA-3DF8199|EPI_ISL_12606693|United Kingdom|BA.2|2022-05-02	99,79
EPI_ISL_12607729	BA.1	BA.1	BA.1_3	CPHL-21878|EPI_ISL_12607729|Egypt|BA.1|2022-02-23	58,21
EPI_ISL_12609660	BA.2	A	A_17	DCGC-509799|EPI_ISL_12609660|Denmark|BA.2|2022-05-03	99,81
EPI_ISL_12610055	BA.2.12.1	BA.2.12.1	BA.2.12.1	DCGC-510019|EPI_ISL_12610055|Denmark|BA.2.12.1|2022-05-03	99,81
EPI_ISL_12610590	BA.5.2.2	A	A_9	LB-R00073-S233|EPI_ISL_12610590|Austria|BA.5.2.2|2022-05-04	95,04
EPI_ISL_12613386	BA.2.12.1	BA.2.12.1	BA.2.12.1	PA-CDC-QDX36261468|EPI_ISL_12613386|USA|BA.2.12.1|2022-04-24	99,81
EPI_ISL_12613511	BA.2	A	A_17	CT-HUB04957|EPI_ISL_12613511|Spain|BA.2|2022-04-29	99,81
EPI_ISL_12614241	BA.2	A	A_14	PA-BOL-M22073803|EPI_ISL_12614241|USA|BA.2|2022-04-26	96,13
EPI_ISL_12614729	BA.2	A	A_14	PL_P1317|EPI_ISL_12614729|Poland|BA.2|2022-02-04	99,81
EPI_ISL_12615394	BA.2.3	BA.2	BA.2_1	CA-Stanford-75_S29|EPI_ISL_12615394|USA|BA.2.3|2022-04-06	99,81
EPI_ISL_12619413	BA.2.12.1	BA.2.12.1	BA.2.12.1	CA-CDC-LC0601399|EPI_ISL_12619413|USA|BA.2.12.1|2022-04-24	97,50
EPI_ISL_12621234	BA.1	BA.1.15	BA.1.15	FL-BPHL-6460|EPI_ISL_12621234|USA|BA.1|2022-01-05	99,64
EPI_ISL_12621446	BA.2.3	A	A_14	CA-CDC-LC0603474|EPI_ISL_12621446|USA|BA.2.3|2022-04-27	89,96
EPI_ISL_12626056	BA.1.1	BA.1.1	BA.1.1_2	OR_UO_MAP004058_S54_L001|EPI_ISL_12626056|USA|BA.1.1|2022-02-28	99,68
EPI_ISL_12628194	BA.2.12.1	BA.2.12.1	BA.2.12.1	PH-RITM-1338|EPI_ISL_12628194|Philippines|BA.2.12.1|2022-04-29	99,81
EPI_ISL_12628700	BA.2	A	A_17	NAQ-HCL722002477201|EPI_ISL_12628700|France|BA.2|2022-04-25	99,81
EPI_ISL_12629096	BA.2	A	A_17	LSPA-3DFBFD8|EPI_ISL_12629096|United Kingdom|BA.2|2022-05-03	99,81
EPI_ISL_12629129	BA.2	BA.2	BA.2_1	LSPA-3DFB9BC|EPI_ISL_12629129|United Kingdom|BA.2|2022-05-03	99,81
EPI_ISL_12630798	BA.2	A	A_14	QEUH-3DF089C|EPI_ISL_12630798|United Kingdom|BA.2|2022-04-27	99,81
EPI_ISL_12631315	BA.1.1	BA.1.1	BA.1.1_3	THL-202209495|EPI_ISL_12631315|Finland|BA.1.1|2022-02-07	99,64
EPI_ISL_12633125	BA.2	BA.2	BA.2_1	ICH-741223260|EPI_ISL_12633125|Israel|BA.2|2022-04-30	86,80
EPI_ISL_12633472	BA.2.31	A	A_14	ICH-741223435|EPI_ISL_12633472|Israel|BA.2.31|2022-05-01	92,01
EPI_ISL_12635452	BA.2.13	BA.2.13	BA.2.13	THL-202212172|EPI_ISL_12635452|Finland|BA.2.13|2022-04-06	99,81
EPI_ISL_12635514	BA.2	BA.2	BA.2_1	THL-202212244|EPI_ISL_12635514|Finland|BA.2|2022-04-04	99,81
EPI_ISL_12636971	BA.2.12.1	BA.2.12.1	BA.2.12.1	KS-KHEL-10431|EPI_ISL_12636971|USA|BA.2.12.1|2022-05-04	90,52
EPI_ISL_12637078	BA.2.9	BA.2	BA.2_1	ICH-741223042|EPI_ISL_12637078|Israel|BA.2.9|2022-04-26	84,35
EPI_ISL_12637296	BA.2	A	A_14	ICH-741222831|EPI_ISL_12637296|Israel|BA.2|2022-04-29	96,18
EPI_ISL_12637676	BA.2	BA.2	BA.2_1	BRE-IPP31294|EPI_ISL_12637676|France|BA.2|2022-04-19	99,81
EPI_ISL_12637803	BA.2	A	A_17	MA-CDC-STM-CP5BVXTTS|EPI_ISL_12637803|USA|BA.2|2022-04-25	99,81
EPI_ISL_12638070	BA.2.12.1	BA.2.12.1	BA.2.12.1	PA-CDC-STM-3D7XGNJDJ|EPI_ISL_12638070|USA|BA.2.12.1|2022-04-26	99,81
EPI_ISL_12638161	BA.2.12.1	BA.2.12.1	BA.2.12.1	PA-CDC-STM-9FY6Z3TAW|EPI_ISL_12638161|USA|BA.2.12.1|2022-04-26	99,81
EPI_ISL_12638784	BA.2.3	A	A_14	CA-CDC-STM-KR57R4VJ6|EPI_ISL_12638784|USA|BA.2.3|2022-04-29	99,81
EPI_ISL_12639094	BA.2	BA.2	BA.2_1	MI-CDC-STM-XP8DCGWJ5|EPI_ISL_12639094|USA|BA.2|2022-05-01	99,81
EPI_ISL_12639473	BA.2.12.1	BA.2.12.1	BA.2.12.1	QC-L00469082001|EPI_ISL_12639473|Canada|BA.2.12.1|2022-04-25	99,81

EPI_ISL_12640217	BA.2	A	A_17	NRL_s2663|EPI_ISL_12640217|Czech Republic|BA.2|2022-04-08	98,51
EPI_ISL_12640224	BA.2.9	BA.2	BA.2_1	NRL_s2672|EPI_ISL_12640224|Czech Republic|BA.2.9|2022-04-14	96,51
EPI_ISL_12640884	BA.2.12.1	BA.2.12.1	BA.2.12.1	CA-CDC-QDX36316743|EPI_ISL_12640884|USA|BA.2.12.1|2022-04-24	99,81
EPI_ISL_12641501	BA.2.3	A	A_14	ND-NDDH-15059|EPI_ISL_12641501|USA|BA.2.3|2022-05-05	90,52
EPI_ISL_12646525	BA.2	A	A_14	VEN-IZSVe-22RS8158-8_VR|EPI_ISL_12646525|Italy|BA.2|2022-05-02	99,81
EPI_ISL_12646940	BA.2.9	A	A_17	MH-NVRL-S22IRL00291102|EPI_ISL_12646940|Ireland|BA.2.9|2022-04-06	90,52
EPI_ISL_12648672	BA.2	A	A_17	DCGC-511336|EPI_ISL_12648672|Denmark|BA.2|2022-05-03	99,81
EPI_ISL_12648750	BA.2.9	BA.2	BA.2_1	DCGC-511415|EPI_ISL_12648750|Denmark|BA.2.9|2022-05-07	99,73
EPI_ISL_12649442	BA.2	A	A_14	PHEC-YYFNZKC|EPI_ISL_12649442|United Kingdom|BA.2|2022-04-26	99,81
EPI_ISL_12649483	BA.1.21	BA.1	BA.1_4	7911|EPI_ISL_12649483|Norway|BA.1.21|2022-02-08	99,64
EPI_ISL_12649533	BA.1.1	BA.1.1	BA.1.1_2	10922|EPI_ISL_12649533|Norway|BA.1.1|2022-02-08	96,72
EPI_ISL_12649794	BA.2	A	A_17	15452|EPI_ISL_12649794|Norway|BA.2|2022-03-11	99,81
EPI_ISL_12651957	BA.2	A	A_17	NB-GD-03719010|EPI_ISL_12651957|Netherlands|BA.2|2022-05-03	99,33
EPI_ISL_12652429	BA.2	BA.2.12	BA.2.12	NY-UB-KSL-04813|EPI_ISL_12652429|USA|BA.2|2022-03-28	93,05
EPI_ISL_12652676	BA.2.12.1	BA.2.12.1	BA.2.12.1	WI-UW-10825|EPI_ISL_12652676|USA|BA.2.12.1|2022-04-27	90,52
EPI_ISL_12652982	BA.2	A	A_17	HDF-IPP31608|EPI_ISL_12652982|France|BA.2|2022-04-25	99,81
EPI_ISL_12653017	BA.2.9	BA.2	BA.2_1	PDL-IPP31675|EPI_ISL_12653017|France|BA.2.9|2022-04-25	99,81
EPI_ISL_12654225	BA.2	A	A_14	PA-CDC-LC0604950|EPI_ISL_12654225|USA|BA.2|2022-04-28	99,81
EPI_ISL_12654534	BA.2.12.1	BA.2.12.1	BA.2.12.1	NY-CDC-LC0605477|EPI_ISL_12654534|USA|BA.2.12.1|2022-04-28	99,81
EPI_ISL_12654846	BA.2	A	A_14	FL-CDC-LC0608615|EPI_ISL_12654846|USA|BA.2|2022-04-29	93,17
EPI_ISL_12655033	BA.2	A	A_14	WA-CDC-LC0608395|EPI_ISL_12655033|USA|BA.2|2022-04-29	99,81
EPI_ISL_12656659	BA.2	BA.2	BA.2_1	CO-CDPHE-2103160350|EPI_ISL_12656659|USA|BA.2|2022-04-19	99,81
EPI_ISL_12657178	BA.2	A	A_14	WA-CDC-LC0609602|EPI_ISL_12657178|USA|BA.2|2022-05-02	99,81
EPI_ISL_12658209	BA.2.3	A	A_4	AR-50422|EPI_ISL_12658209|Chile|BA.2.3|2022-04-13	99,79
EPI_ISL_12659398	BA.2.9	A	A_17	CA-CDC-QDX36360589|EPI_ISL_12659398|USA|BA.2.9|2022-04-23	99,81
EPI_ISL_12660613	BA.1.1	BA.1.1	BA.1.1_2	FL-BPHL-6763|EPI_ISL_12660613|USA|BA.1.1|2022-01-18	99,64
EPI_ISL_12660787	BA.2.3.17	A	A_14	HI-H2210485|EPI_ISL_12660787|USA|BA.2.3.17|2022-04-18	99,50
EPI_ISL_12661820	BA.2.3	A	A_14	BC-BCCDC-416233|EPI_ISL_12661820|Canada|BA.2.3|2022-04-06	99,81
EPI_ISL_12662516	BA.2.3	BA.2	BA.2_1	BC-BCCDC-418572|EPI_ISL_12662516|Canada|BA.2.3|2022-04-13	99,81
EPI_ISL_12663135	BA.2	A	A_14	BC-BCCDC-423566|EPI_ISL_12663135|Canada|BA.2|2022-04-14	99,81
EPI_ISL_12663561	BA.2.3	A	A_14	BC-BCCDC-422511|EPI_ISL_12663561|Canada|BA.2.3|2022-04-15	99,81
EPI_ISL_12664535	BA.2	BA.2	BA.2_1	BC-BCCDC-424034|EPI_ISL_12664535|Canada|BA.2|2022-04-19	99,81
EPI_ISL_12665298	BA.2	A	A_14	IMR_WC196823|EPI_ISL_12665298|Malaysia|BA.2|2022-03-17	90,52
EPI_ISL_12665733	BA.2	A	A_14	BC-BCCDC-424724|EPI_ISL_12665733|Canada|BA.2|2022-04-26	99,81
EPI_ISL_12666770	BA.2	A	A_14	LSPA-3E02465|EPI_ISL_12666770|United Kingdom|BA.2|2022-05-04	99,81
EPI_ISL_12668459	BA.2.9	BA.2	BA.2_1	HB-RKI-I-777745|EPI_ISL_12668459|Germany|BA.2.9|2022-04-15	99,75
EPI_ISL_12668669	BA.2	BA.2	BA.2_1	BE-RKI-I-777962|EPI_ISL_12668669|Germany|BA.2|2022-04-12	99,69
EPI_ISL_12669216	BA.2	BA.2	BA.2_1	HB-RKI-I-778544|EPI_ISL_12669216|Germany|BA.2|2022-02-28	99,71
EPI_ISL_12669278	BA.1.15	BA.1	BA.1_3	TH-RKI-I-778611|EPI_ISL_12669278|Germany|BA.1.15|2022-02-23	99,58
EPI_ISL_12669636	BA.2.9	BA.2	BA.2_1	SH-RKI-I-778978|EPI_ISL_12669636|Germany|BA.2.9|2022-04-25	99,81
EPI_ISL_12669782	BA.1.1.1	BA.1.1	BA.1.1_3	NW-RKI-I-779331|EPI_ISL_12669782|Germany|BA.1.1.1|2022-02-04	99,60
EPI_ISL_12670013	BA.1.1	BA.1.1	BA.1.1_2	NW-RKI-I-779600|EPI_ISL_12670013|Germany|BA.1.1|2022-02-05	99,54
EPI_ISL_12670016	BA.1.1.1	BA.1.1	BA.1.1_3	NW-RKI-I-779603|EPI_ISL_12670016|Germany|BA.1.1.1|2022-02-03	99,62
EPI_ISL_12670454	BA.2	BA.2	BA.2_1	RP-RKI-I-780024|EPI_ISL_12670454|Germany|BA.2|2022-04-25	98,36
EPI_ISL_12670810	BA.2.9	BA.2	BA.2_1	BW-RKI-I-780308|EPI_ISL_12670810|Germany|BA.2.9|2022-04-24	99,81
EPI_ISL_12670835	BA.2.3	A	A_14	BW-RKI-I-780335|EPI_ISL_12670835|Germany|BA.2.3|2022-04-23	99,81
EPI_ISL_12672054	BA.2	A	A_14	SH-RKI-I-782010|EPI_ISL_12672054|Germany|BA.2|2022-04-26	99,81
EPI_ISL_12672401	BA.2	A	A_17	NI-RKI-I-782382|EPI_ISL_12672401|Germany|BA.2|2022-04-16	99,81
EPI_ISL_12673568	BA.2	BA.2	BA.2_1	BY-RKI-I-783570|EPI_ISL_12673568|Germany|BA.2|2022-04-29	99,81
EPI_ISL_12673619	BA.2.23	BA.2	BA.2_1	BY-RKI-I-783649|EPI_ISL_12673619|Germany|BA.2.23|2022-05-01	99,81
EPI_ISL_12673736	BA.2	A	A_14	RP-RKI-I-783830|EPI_ISL_12673736|Germany|BA.2|2022-04-29	98,21
EPI_ISL_12675587	BA.2.9	BA.2	BA.2_1	BW-RKI-I-786059|EPI_ISL_12675587|Germany|BA.2.9|2022-04-29	99,81
EPI_ISL_12676624	BA.2.9	BA.2	BA.2_1	RP-RKI-I-787160|EPI_ISL_12676624|Germany|BA.2.9|2022-04-29	99,03
EPI_ISL_12677932	BA.2	BA.2	BA.2_1	NW-RKI-I-788851|EPI_ISL_12677932|Germany|BA.2|2022-05-03	96,55
EPI_ISL_12679527	BA.1	BA.1.15	BA.1.15	RJ-FIOCRUZ-8976|EPI_ISL_12679527|Brazil|BA.1|2022-01-28	95,23
EPI_ISL_12680573	BA.1.1.14	BA.1.1	BA.1.1_3	PR-FIOCRUZ-9088|EPI_ISL_12680573|Brazil|BA.1.1.14|2022-03-13	99,64
EPI_ISL_12684029	BA.2	A	A_14	ZH-EMC-5422|EPI_ISL_12684029|Netherlands|BA.2|2022-03-22	99,81
EPI_ISL_12684188	BA.2	A	A_14	ZH-EMC-5633|EPI_ISL_12684188|Netherlands|BA.2|2022-04-14	99,81
EPI_ISL_12684959	BA.2	BA.2.2	BA.2.2	TKYkbm16538|EPI_ISL_12684959|Japan|BA.2|2022-03-29	99,81
EPI_ISL_12685625	BA.2.10.1	BA.2.10.1	BA.2.10.1	TKYkbm17110|EPI_ISL_12685625|Japan|BA.2.10.1|2022-04-01	99,81
EPI_ISL_12686800	BA.1.1	BA.1	BA.1_4	RM-47424|EPI_ISL_12686800|Chile|BA.1.1|2022-04-08	89,81
EPI_ISL_12688422	BA.2	BA.2.12	BA.2.12	PAC-LBZCentre-AW2204195028|EPI_ISL_12688422|France|BA.2|2022-04-19	94,26
EPI_ISL_12688634	BA.2	BA.2	BA.2_1	ULG-26211|EPI_ISL_12688634|Belgium|BA.2|2022-05-04	99,81
EPI_ISL_12688913	BA.2.10	BA.2	BA.2_1	GES-HMN-22042200231|EPI_ISL_12688913|France|BA.2.10|2022-04-11	90,40
EPI_ISL_12690526	BA.2.12.1	BA.2.12.1	BA.2.12.1	MN-MDH-24433|EPI_ISL_12690526|USA|BA.2.12.1|2022-04-06	99,81
EPI_ISL_12691897	BA.2.9	A	A_17	IL-RIPHL_60869_G|EPI_ISL_12691897|USA|BA.2.9|2022-03-30	99,45
EPI_ISL_12692052	BA.1	BA.1	BA.1_3	WACCBIP-GS2640|EPI_ISL_12692052|Ghana|BA.1|2021-12-21	96,03
EPI_ISL_12692835	BA.2.12.1	BA.2.12.1	BA.2.12.1	CA-HLX-STM-43NWB9TJE|EPI_ISL_12692835|USA|BA.2.12.1|2022-04-13	99,79
EPI_ISL_12693389	BA.2	BA.2	BA.2_1	CA-CDPH-6000009309|EPI_ISL_12693389|USA|BA.2|2022-02-25	90,52
EPI_ISL_12695784	BA.2	A	A_14	WA-PHL-018755|EPI_ISL_12695784|USA|BA.2|2022-04-08	99,79
EPI_ISL_12695940	BA.2	A	A_14	WA-PHL-019661|EPI_ISL_12695940|USA|BA.2|2022-05-02	95,36
EPI_ISL_12699530	BA.2	A	A_17	ICH-741224303|EPI_ISL_12699530|Israel|BA.2|2022-05-06	99,81
EPI_ISL_12700111	BA.2.9	A	A_17	ICH-741223637|EPI_ISL_12700111|Israel|BA.2.9|2022-05-07	99,81
EPI_ISL_12701055	BA.2	A	A_17	DCGC-512297|EPI_ISL_12701055|Denmark|BA.2|2022-05-08	99,81
EPI_ISL_12701088	BA.2.9.3	BA.2.9.3	BA.2.9.3	DCGC-512330|EPI_ISL_12701088|Denmark|BA.2.9.3|2022-05-04	99,81
EPI_ISL_12702566	BA.2	BA.2	BA.2_1	LNS4962302|EPI_ISL_12702566|Luxembourg|BA.2|2022-04-19	99,81
EPI_ISL_12703280	BA.2	BA.2	BA.2_2	LNS2713177|EPI_ISL_12703280|Luxembourg|BA.2|2022-04-19	97,98
EPI_ISL_12703405	BA.2	BA.2	BA.2_1	EDB59936|EPI_ISL_12703405|United Kingdom|BA.2|2022-04-17	94,09
EPI_ISL_12703714	BA.2.23	A	A_14	EDB60722|EPI_ISL_12703714|United Kingdom|BA.2.23|2022-04-25	99,81
EPI_ISL_12705341	BA.2	BA.2	BA.2_1	PHEC-YYFGRH1|EPI_ISL_12705341|United Kingdom|BA.2|2022-04-28	99,81
EPI_ISL_12707334	BA.2.12.1	BA.2.12.1	BA.2.12.1	NY-PRL-0506_00J03|EPI_ISL_12707334|USA|BA.2.12.1|2022-05-02	99,81
EPI_ISL_12708334	BA.2.12.1	BA.2.12.1	BA.2.12.1	NY-PRL-2022_0509_02C01|EPI_ISL_12708334|USA|BA.2.12.1|2022-05-04	99,81
EPI_ISL_12708617	BA.1.1	BA.1.1	BA.1.1_2	Tb-sNGS5027|EPI_ISL_12708617|Georgia|BA.1.1|2022-01-12	96,03
EPI_ISL_12709804	BA.1	BA.1	BA.1_4	LHUB-ULB_SWN932|EPI_ISL_12709804|Belgium|BA.1|2022-01-18	86,62
EPI_ISL_12711415	BA.2	A	A_14	IN-CDC-STM-ZBDUN39Q6|EPI_ISL_12711415|USA|BA.2|2022-05-02	99,81
EPI_ISL_12711646	BA.2	BA.2	BA.2_1	IN-CDC-STM-AX2R5N4JK|EPI_ISL_12711646|USA|BA.2|2022-05-02	99,43
EPI_ISL_12711695	BA.2.9.2	BA.2	BA.2_1	GA-CDC-STM-77UXWRZNX|EPI_ISL_12711695|USA|BA.2.9.2|2022-05-02	99,81
EPI_ISL_12712185	BA.2.12	BA.2.12	BA.2.12	IL-CDC-STM-TTBMZUXU6|EPI_ISL_12712185|USA|BA.2.12|2022-05-03	99,81
EPI_ISL_12712442	BA.2	A	A_14	MN-CDC-STM-YEWSB9KTR|EPI_ISL_12712442|USA|BA.2|2022-05-04	99,62
EPI_ISL_12713064	BA.2.48	BA.2.48	BA.2.48	NJ-PHEL-V22020528|EPI_ISL_12713064|USA|BA.2.48|2022-04-27	90,52
EPI_ISL_12715119	BA.2.7	BA.2.12	BA.2.12	MA-UMASSMED-202150842|EPI_ISL_12715119|USA|BA.2.7|2022-03-30	95,27
EPI_ISL_12718086	BA.2.3.17	A	A_14	MP-CDC-2-5776264|EPI_ISL_12718086|Northern Mariana Islands|BA.2.3.17|2022-02-21	98,63
EPI_ISL_12718197	BA.1.1	BA.1.1	BA.1.1_2	TKYnat0322|EPI_ISL_12718197|Japan|BA.1.1|2022-04-02	99,64
EPI_ISL_12719778	BA.2	A	A_14	TX-CDC-QDX36405968|EPI_ISL_12719778|USA|BA.2|2022-04-27	99,81
EPI_ISL_12722284	BA.2	BA.2	BA.2_1	ON-PHL-22-21573|EPI_ISL_12722284|Canada|BA.2|2022-05-06	99,81
EPI_ISL_12723344	BA.2.12.1	BA.2.12.1	BA.2.12.1	LSPA-3E0DD05|EPI_ISL_12723344|United Kingdom|BA.2.12.1|2022-05-09	99,81
EPI_ISL_12724503	BA.2	A	A_17	QEUH-3DFC872|EPI_ISL_12724503|United Kingdom|BA.2|2022-05-03	99,81
EPI_ISL_12724635	BA.2.10.1	BA.2.10.1	BA.2.10.1	IC-8756|EPI_ISL_12724635|Japan|BA.2.10.1|2022-04-20	99,81
EPI_ISL_12724873	BA.2.3	A	A_14	IC-8993|EPI_ISL_12724873|Japan|BA.2.3|2022-04-22	99,81
EPI_ISL_12726113	BA.2	BA.2	BA.2_1	CHU722033115503|EPI_ISL_12726113|Reunion|BA.2|2022-03-30	90,52
EPI_ISL_12726187	BA.2	A	A_14	CHU722032235803|EPI_ISL_12726187|Reunion|BA.2|2022-03-22	90,52

EPI_ISL_12726444	BA.2.49	BA.2	BA.2_1	CHU79800012045202|EPI_ISL_12726444|Reunion|BA.2.49|2022-03-17	90,52
EPI_ISL_12726458	BA.1.1.1	BA.1.1	BA.1.1_2	CHU79800011880503|EPI_ISL_12726458|Reunion|BA.1.1.1|2022-03-16	89,75
EPI_ISL_12726601	BA.2	BA.2.61	BA.2.61	TN-CDFD-W8-34|EPI_ISL_12726601|India|BA.2|2022-05-06	90,52
EPI_ISL_12727789	BA.2	BA.2	BA.2_1	CeMM28818|EPI_ISL_12727789|Austria|BA.2|2022-04-25	95,23
EPI_ISL_12728416	BA.2	BA.2	BA.2_1	MD-HRYC-12301637|EPI_ISL_12728416|Spain|BA.2|2022-04-28	99,81
EPI_ISL_12729473	BA.2	A	A_17	BB-RKI-I-790936|EPI_ISL_12729473|Germany|BA.2|2022-05-09	99,81
EPI_ISL_12730408	BA.2	BA.2.31	BA.2.31	NI-RKI-I-791905|EPI_ISL_12730408|Germany|BA.2|2022-05-02	99,81
EPI_ISL_12731153	BA.2	XAR	XAR	CHU79800011813802|EPI_ISL_12731153|Reunion|BA.2|2022-04-18	90,52
EPI_ISL_12731736	BA.2.9	A	A_14	SL-RKI-I-793301|EPI_ISL_12731736|Germany|BA.2.9|2022-04-23	99,60
EPI_ISL_12731830	BA.2.9.3	BA.2.9.3	BA.2.9.3	HE-RKI-I-793403|EPI_ISL_12731830|Germany|BA.2.9.3|2022-04-26	99,73
EPI_ISL_12734454	BA.2.56	BA.2.12	BA.2.12	WB-INSACOG-1930301778957|EPI_ISL_12734454|India|BA.2.56|2022-01-08	75,83
EPI_ISL_12735698	BA.2.9	BA.2	BA.2_1	SN-RKI-I-796617|EPI_ISL_12735698|Germany|BA.2.9|2022-02-25	99,73
EPI_ISL_12736434	BA.2.46	BA.2	BA.2_1	SN-RKI-I-797263|EPI_ISL_12736434|Germany|BA.2.46|2022-05-03	99,81
EPI_ISL_12736702	BA.2	BA.2	BA.2_1	RP-RKI-I-797527|EPI_ISL_12736702|Germany|BA.2|2022-05-03	99,29
EPI_ISL_12737504	BA.2.9	A	A_17	RP-RKI-I-798275|EPI_ISL_12737504|Germany|BA.2.9|2022-05-06	99,79
EPI_ISL_12737557	BA.2	A	A_17	RP-RKI-I-798339|EPI_ISL_12737557|Germany|BA.2|2022-05-05	99,79
EPI_ISL_12739489	BA.2.12.1	BA.2.12.1	BA.2.12.1	CT-Yale-19454|EPI_ISL_12739489|USA|BA.2.12.1|2022-04-28	99,81
EPI_ISL_12739654	BA.2.10	BA.2.10	BA.2.10	UNIMAS-B13263|EPI_ISL_12739654|Malaysia|BA.2.10|2022-02-18	99,79
EPI_ISL_12740127	BA.2.12.1	BA.2.12.1	BA.2.12.1	IN-LH000207151|EPI_ISL_12740127|USA|BA.2.12.1|2022-05-05	99,81
EPI_ISL_12741351	BA.2.3	BA.2.12	BA.2.12	CVL-8006708|EPI_ISL_12741351|Israel|BA.2.3|2022-04-28	88,30
EPI_ISL_12741875	BA.2	BA.2	BA.2_1	CVL-8006918|EPI_ISL_12741875|Israel|BA.2|2022-05-09	98,78
EPI_ISL_12742238	BA.2	BA.2	BA.2_1	CVL-9004337|EPI_ISL_12742238|Israel|BA.2|2022-05-09	97,08
EPI_ISL_12743426	BA.2.3	BA.2	BA.2_1	LM-NVRL-ecS22IRL00288813|EPI_ISL_12743426|Ireland|BA.2.3|2022-04-04	96,53
EPI_ISL_12743431	BA.2	BA.2	BA.2_1	LM-NVRL-ecS22IRL00288798|EPI_ISL_12743431|Ireland|BA.2|2022-04-04	96,30
EPI_ISL_12743506	BA.2.9	XAM	XAM	MO-NVRL-ecS22IRL00279834|EPI_ISL_12743506|Ireland|BA.2.9|2022-03-29	96,53
EPI_ISL_12744342	BA.2.12.1	BA.2.12.1	BA.2.12.1	NC-CORVASEQ-CLT-007778|EPI_ISL_12744342|USA|BA.2.12.1|2022-04-22	94,26
EPI_ISL_12745243	BA.2.3	A	A_14	CA-CDC-QDX36515008|EPI_ISL_12745243|USA|BA.2.3|2022-04-29	99,81
EPI_ISL_12745409	BA.2	BA.2	BA.2_1	TX-CDC-QDX36513729|EPI_ISL_12745409|USA|BA.2|2022-04-29	99,81
EPI_ISL_12745434	BA.2.1	A	A_4	TX-CDC-QDX36514531|EPI_ISL_12745434|USA|BA.2.1|2022-04-29	99,81
EPI_ISL_12747239	BA.2	BA.2.12	BA.2.12	IDF-LBZCentre-CH2203140093|EPI_ISL_12747239|France|BA.2|2022-03-14	94,26
EPI_ISL_12747391	BA.2	BA.2	BA.2_1	CVL-LBZCentre-A390078920|EPI_ISL_12747391|France|BA.2|2022-05-09	99,81
EPI_ISL_12749507	BA.2	A	A_17	PHEC-YYF8JD6|EPI_ISL_12749507|United Kingdom|BA.2|2022-05-01	99,79
EPI_ISL_12749567	BA.2	BA.2	BA.2_1	PHEP-YYR64NB|EPI_ISL_12749567|United Kingdom|BA.2|2022-04-27	99,77
EPI_ISL_12750517	BA.2	BA.2	BA.2_1	DCGC-514417|EPI_ISL_12750517|Denmark|BA.2|2022-05-12	99,81
EPI_ISL_12750652	BA.2.38	BA.2.40.1	BA.2.40.1	DCGC-514552|EPI_ISL_12750652|Denmark|BA.2.38|2022-05-12	99,79
EPI_ISL_12751140	BA.1	BA.1.15	BA.1.15	DCGC-515050|EPI_ISL_12751140|Denmark|BA.1|2021-12-13	99,64
EPI_ISL_12751473	BA.2	BA.2	BA.2_1	BRE-IPP33013|EPI_ISL_12751473|France|BA.2|2022-05-02	99,81
EPI_ISL_12752172	BA.2	A	A_17	19997|EPI_ISL_12752172|Norway|BA.2|2022-04-12	99,81
EPI_ISL_12753717	BA.2.9	A	A_17	QC-L00472334001|EPI_ISL_12753717|Canada|BA.2.9|2022-05-02	99,81
EPI_ISL_12756795	BA.2.12.1	BA.2.12.1	BA.2.12.1	FL-CDC-LC0612690|EPI_ISL_12756795|USA|BA.2.12.1|2022-05-01	99,81
EPI_ISL_12757354	BA.2.65	BA.2	BA.2_1	CA-CDC-LC0617374|EPI_ISL_12757354|USA|BA.2.65|2022-05-02	99,81
EPI_ISL_12757895	BA.2.12.1	BA.2.12.1	BA.2.12.1	NJ-CDC-LC0609745|EPI_ISL_12757895|USA|BA.2.12.1|2022-05-02	99,81
EPI_ISL_12758080	BA.2	A	A_14	AZKLINA122-100129|EPI_ISL_12758080|Belgium|BA.2|2022-04-28	98,24
EPI_ISL_12758087	BA.4.1	A	A_3	AZKLINA122-102493|EPI_ISL_12758087|Belgium|BA.4.1|2022-05-03	98,13
EPI_ISL_12759843	BA.2.3	A	A_14	CA-CDC-LC0617405|EPI_ISL_12759843|USA|BA.2.3|2022-05-03	99,81
EPI_ISL_12762985	BA.1	AY.43	AY.43_1	IMBA_IMP-1870_C07|EPI_ISL_12762985|Austria|BA.1|2022-05-02	49,80
EPI_ISL_12766433	BA.2	BA.2	BA.2_1	MA-MGB-05880|EPI_ISL_12766433|USA|BA.2|2022-04-27	99,81
EPI_ISL_12766727	BA.1.1	BA.1.1	BA.1.1_2	FL-BPHL-7037|EPI_ISL_12766727|USA|BA.1.1|2022-01-18	99,64
EPI_ISL_12769489	BA.2	BA.2.12	BA.2.12	MA-Broad-CRSP_N465S3LLPSEWGXDG|EPI_ISL_12769489|USA|BA.2|2022-05-02	94,73
EPI_ISL_12769600	BA.2.12.1	BA.2.12.1	BA.2.12.1	MA-Broad-CRSP_TIYFZM6GQQDZDJ4Z|EPI_ISL_12769600|USA|BA.2.12.1|2022-05-02	95,19
EPI_ISL_12769717	BA.2.12.1	BA.2.12.1	BA.2.12.1	ME-Broad-CRSP_FXG276N3D45IUI6Z|EPI_ISL_12769717|USA|BA.2.12.1|2022-05-02	95,19
EPI_ISL_12769828	BA.2.12.1	BA.2.12.1	BA.2.12.1	VT-Broad-CRSP_U5VAUC6VDKOFBJ3O|EPI_ISL_12769828|USA|BA.2.12.1|2022-05-03	95,23
EPI_ISL_12770311	BA.2.12.1	BA.2.12.1	BA.2.12.1	IL-CDC-LC0620792|EPI_ISL_12770311|USA|BA.2.12.1|2022-05-05	99,81
EPI_ISL_12770859	BA.2	A	A_4	WA3456|EPI_ISL_12770859|Australia|BA.2|2022-05-04	99,37
EPI_ISL_12772991	BA.2	A	A_17	QEUH-3D1412E|EPI_ISL_12772991|United Kingdom|BA.2|2022-04-02	99,81
EPI_ISL_12773120	BA.2.3	A	A_14	QEUH-3D1309B|EPI_ISL_12773120|United Kingdom|BA.2.3|2022-04-02	99,81
EPI_ISL_12775118	BA.2	A	A_14	QEUH-3CFDF22|EPI_ISL_12775118|United Kingdom|BA.2|2022-03-29	99,81
EPI_ISL_12780545	BA.2	BA.2.10	BA.2.10	DCGC-515318|EPI_ISL_12780545|Denmark|BA.2|2022-05-12	99,83
EPI_ISL_12782289	BA.2.9	BA.2	BA.2_1	DCGC-516815|EPI_ISL_12782289|Denmark|BA.2.9|2022-05-14	99,77
EPI_ISL_12783558	BA.2.3	BA.2	BA.2_1	OV-RIVM-99697|EPI_ISL_12783558|Netherlands|BA.2.3|2022-04-30	99,81
EPI_ISL_12785357	B.1.1.529	BA.2.10	BA.2.10	TN-INSACOG-CSIR-NEERI1709|EPI_ISL_12785357|India|B.1.1.529|2022-05-05	79,13
EPI_ISL_12785520	BA.2.38	BA.2.38	BA.2.38	UP-ICMR-INSACOG-KGMU-ZY63-659|EPI_ISL_12785520|India|BA.2.38|2022-04-29	83,72
EPI_ISL_12787260	BA.2	BA.2	BA.2_1	BFC-HMN-22042220768|EPI_ISL_12787260|France|BA.2|2022-03-27	99,81
EPI_ISL_12788197	BA.2	BA.2	BA.2_1	08-094263-MB|EPI_ISL_12788197|Slovenia|BA.2|2022-05-06	99,81
EPI_ISL_12788974	BA.2.3	A	A_14	AR-CDC-QDX36606240|EPI_ISL_12788974|USA|BA.2.3|2022-05-03	99,81
EPI_ISL_12789484	BA.2.12.1	BA.2.12.1	BA.2.12.1	MD-CDC-QDX36605212|EPI_ISL_12789484|USA|BA.2.12.1|2022-05-03	99,81
EPI_ISL_12789668	BA.2.9	A	A_17	NC-CDC-QDX36604822|EPI_ISL_12789668|USA|BA.2.9|2022-05-03	99,81
EPI_ISL_12791048	BA.2.12.1	BA.2.12.1	BA.2.12.1	MA-Broad-CRSP_SGDHQVYBR2GM6GWD|EPI_ISL_12791048|USA|BA.2.12.1|2022-05-04	99,81
EPI_ISL_12791491	BA.2.12.1	BA.2.12.1	BA.2.12.1	VT-Broad-CRSP_CEZO4LOZGE2IJVNF|EPI_ISL_12791491|USA|BA.2.12.1|2022-05-07	98,09
EPI_ISL_12791829	BA.2	A	A_4	WA-UW-22042138407|EPI_ISL_12791829|USA|BA.2|2022-04-21	99,81
EPI_ISL_12792144	BA.2.10	BA.2.12	BA.2.12	TG-RFCH05185_CIC9399|EPI_ISL_12792144|India|BA.2.10|2022-01-17	91,76
EPI_ISL_12796685	BA.1	BA.1.1	BA.1.1_2	KDCA15512s|EPI_ISL_12796685|South Korea|BA.1|2022-01-26	72,58
EPI_ISL_12800893	BA.2	A	A_14	CO-CDPHE-2103149208|EPI_ISL_12800893|USA|BA.2|2022-04-15	99,81
EPI_ISL_12801305	BA.2	BA.2.12.1	BA.2.12.1	CO-CDPHE-2103183278|EPI_ISL_12801305|USA|BA.2|2022-04-18	75,73
EPI_ISL_12802640	BA.2	BA.2.10	BA.2.10	MG-FIOCRUZ-1309|EPI_ISL_12802640|Brazil|BA.2|2022-04-19	99,79
EPI_ISL_12803209	BA.2.12.1	BA.2.12.1	BA.2.12.1	CO-CDPHE-2103147371|EPI_ISL_12803209|USA|BA.2.12.1|2022-04-15	94,54
EPI_ISL_12803446	BA.5.5	BA.5.5	BA.5.5_1	UT-UPHL-220513863262|EPI_ISL_12803446|USA|BA.5.5|2022-04-23	99,69
EPI_ISL_12806398	BA.2.12.1	BA.2.12.1	BA.2.12.1	CA-SLOPH-C1314|EPI_ISL_12806398|USA|BA.2.12.1|2022-05-10	90,52
EPI_ISL_12806448	BA.2.12.1	BA.2.12.1	BA.2.12.1	HI-H2210625|EPI_ISL_12806448|USA|BA.2.12.1|2022-04-27	99,50
EPI_ISL_12810461	BA.2.9	BA.2	BA.2_1	EDB61064|EPI_ISL_12810461|United Kingdom|BA.2.9|2022-04-25	94,09
EPI_ISL_12810531	BA.2	BA.2	BA.2_1	NIRE-01ce1a|EPI_ISL_12810531|United Kingdom|BA.2|2022-04-18	99,81
EPI_ISL_12811725	BA.2	A	A_17	DCGC-517506|EPI_ISL_12811725|Denmark|BA.2|2022-01-07	99,81
EPI_ISL_12811766	BA.2	BA.2	BA.2_1	NB-58743|EPI_ISL_12811766|Chile|BA.2|2022-05-05	99,75
EPI_ISL_12812083	BA.2.12	BA.2.12	BA.2.12	12208687701|EPI_ISL_12812083|Belgium|BA.2.12|2022-05-13	96,32
EPI_ISL_12814136	BA.5.1	A	A_3	SN-RKI-I-801325|EPI_ISL_12814136|Germany|BA.5.1|2022-04-25	99,56
EPI_ISL_12814607	BA.2	BA.2	BA.2_1	BY-RKI-I-801825|EPI_ISL_12814607|Germany|BA.2|2022-03-09	99,73
EPI_ISL_12815135	BA.2	BA.2	BA.2_1	SN-RKI-I-802390|EPI_ISL_12815135|Germany|BA.2|2022-04-21	99,73
EPI_ISL_12816311	BA.2	A	A_17	HH-RKI-I-804192|EPI_ISL_12816311|Germany|BA.2|2022-04-29	99,39
EPI_ISL_12816766	BA.2.9	BA.2	BA.2_1	SL-RKI-I-804661|EPI_ISL_12816766|Germany|BA.2.9|2022-04-18	99,73
EPI_ISL_12817161	BA.1.1	BA.1.1	BA.1.1_2	BY-RKI-I-805083|EPI_ISL_12817161|Germany|BA.1.1|2022-02-22	99,58
EPI_ISL_12817604	BA.1.1	BA.1.1	BA.1.1_2	BW-RKI-I-805565|EPI_ISL_12817604|Germany|BA.1.1|2022-02-28	99,62
EPI_ISL_12818026	BA.2	A	A_14	SN-RKI-I-806011|EPI_ISL_12818026|Germany|BA.2|2022-04-19	99,68
EPI_ISL_12818794	BA.2	BA.2	BA.2_1	SN-RKI-I-806812|EPI_ISL_12818794|Germany|BA.2|2022-03-03	99,75
EPI_ISL_12819534	BA.2	BA.1.17.2	BA.1.17.2	BY-RKI-I-808043|EPI_ISL_12819534|Germany|BA.2|2022-03-23	95,50
EPI_ISL_12821283	BA.2	A	A_14	BW-RKI-I-810041|EPI_ISL_12821283|Germany|BA.2|2022-04-28	99,81
EPI_ISL_12821783	BA.2	BA.2	BA.2_1	SN-RKI-I-810570|EPI_ISL_12821783|Germany|BA.2|2022-04-28	99,66
EPI_ISL_12822549	BA.1.1	BA.1.1	BA.1.1_2	SN-RKI-I-811215|EPI_ISL_12822549|Germany|BA.1.1|2022-02-25	99,58
EPI_ISL_12824526	BA.2.9	A	A_17	NI-RKI-I-813643|EPI_ISL_12824526|Germany|BA.2.9|2022-05-06	99,81
EPI_ISL_12825529	BA.2	BA.2	BA.2_1	SN-RKI-I-814956|EPI_ISL_12825529|Germany|BA.2|2022-02-21	99,71
EPI_ISL_12826156	BA.2	BA.2	BA.2_1	SH-RKI-I-815642|EPI_ISL_12826156|Germany|BA.2|2022-05-06	99,73
EPI_ISL_12826534	BA.1.1	BA.1.1	BA.1.1_2	NW-RKI-I-816087|EPI_ISL_12826534|Germany|BA.1.1|2022-02-18	99,35

EPI_ISL_12830662	BA.1.18	BA.1.15	BA.1.15	SC-MUSC-05325|EPI_ISL_12830662|USA|BA.1.18|2021-12-21	99,64
EPI_ISL_12830722	BA.1.1	BA.1.1	BA.1.1_2	SC-MUSC-06979|EPI_ISL_12830722|USA|BA.1.1|2022-03-03	99,64
EPI_ISL_12832142	BA.2	AY.53	AY.53	AB-ABPHL-65794|EPI_ISL_12832142|Canada|BA.2|2022-05-01	78,23
EPI_ISL_12834877	BA.2	BA.2	BA.2_1	CA-HLX-STM-N47BDG2EA|EPI_ISL_12834877|USA|BA.2|2022-04-22	99,81
EPI_ISL_12834957	BA.1	BA.1.15	BA.1.15	CA-HLX-STM-SEKJUFRJ4|EPI_ISL_12834957|USA|BA.1|2021-12-30	96,16
EPI_ISL_12835567	BA.2.12.1	BA.2.12.1	BA.2.12.1	NY-MSK-5537|EPI_ISL_12835567|USA|BA.2.12.1|2022-05-11	94,47
EPI_ISL_12835661	BA.2	A	A_17	TX-DSHS-20387|EPI_ISL_12835661|USA|BA.2|2022-05-05	86,97
EPI_ISL_12837602	BA.2.9	BA.2	BA.2_1	LNS9466634|EPI_ISL_12837602|Luxembourg|BA.2.9|2022-04-27	99,81
EPI_ISL_12838459	BA.2	A	A_14	LSPA-3E16176|EPI_ISL_12838459|United Kingdom|BA.2|2022-05-12	99,81
EPI_ISL_12840168	BA.2	A	A_14	IL-NM-29496|EPI_ISL_12840168|USA|BA.2|2022-05-07	99,81
EPI_ISL_12841115	BA.1	BA.1	BA.1_3	PR-FIOCRUZ-ICC921|EPI_ISL_12841115|Brazil|BA.1|2021-12-28	91,17
EPI_ISL_12842055	BA.1.1	BA.1	BA.1_1	PR-FIOCRUZ-ICC1260|EPI_ISL_12842055|Brazil|BA.1.1|2022-01-07	97,90
EPI_ISL_12843215	BA.1	BA.1	BA.1_4	PR-FIOCRUZ-ICC2359|EPI_ISL_12843215|Brazil|BA.1|2022-03-01	99,64
EPI_ISL_12843577	BA.1.1	BA.1.1	BA.1.1_2	PR-FIOCRUZ-ICC2852|EPI_ISL_12843577|Brazil|BA.1.1|2022-01-06	99,64
EPI_ISL_12848811	BA.2.3	A	A_14	VIC56109|EPI_ISL_12848811|Australia|BA.2.3|2022-04-20	99,81
EPI_ISL_12849238	BA.2	A	A_14	VIC56549|EPI_ISL_12849238|Australia|BA.2|2022-04-27	99,81
EPI_ISL_12850656	BA.2	BA.2	BA.2_1	DCGC-517562|EPI_ISL_12850656|Denmark|BA.2|2022-05-01	99,81
EPI_ISL_12851661	BA.2	BA.2	BA.2_1	PDL-IPP33690|EPI_ISL_12851661|France|BA.2|2022-05-02	99,81
EPI_ISL_12852708	BA.2.3	BA.2	BA.2_1	PGCV_008_0374|EPI_ISL_12852708|Philippines|BA.2.3|2022-02-28	99,22
EPI_ISL_12854325	BA.2	BA.2	BA.2_1	MD-H12O_220675|EPI_ISL_12854325|Spain|BA.2|2022-05-04	77,20
EPI_ISL_12854984	BA.2	BA.2	BA.2_1	PDL-HMN-22052020487|EPI_ISL_12854984|France|BA.2|2022-03-28	95,02
EPI_ISL_12856159	BA.2.36	BA.2	BA.2_1	PDL-HMN-22052020553|EPI_ISL_12856159|France|BA.2.36|2022-03-28	96,05
EPI_ISL_12857305	BA.2.12.1	BA.2.12.1	BA.2.12.1	NY-PRL-220516_02E24|EPI_ISL_12857305|USA|BA.2.12.1|2022-05-15	99,81
EPI_ISL_12857815	BA.2.12.1	BA.2.12.1	BA.2.12.1	NY-PRL-220516_05B14|EPI_ISL_12857815|USA|BA.2.12.1|2022-05-13	99,81
EPI_ISL_12858483	BA.2	A	A_14	NY-PRL-220511_07C14|EPI_ISL_12858483|USA|BA.2|2022-05-10	99,81
EPI_ISL_12858663	BA.2.12.1	BA.2.12.1	BA.2.12.1	NY-PRL-220511_08A18|EPI_ISL_12858663|USA|BA.2.12.1|2022-05-10	99,81
EPI_ISL_12859131	BA.2	A	A_14	NY-PRL-220511_00J20|EPI_ISL_12859131|USA|BA.2|2022-05-06	99,81
EPI_ISL_12859145	BA.2.12.1	BA.2.12.1	BA.2.12.1	NY-PRL-220511_00K18|EPI_ISL_12859145|USA|BA.2.12.1|2022-05-05	99,81
EPI_ISL_12860024	BA.2	A	A_17	MC-HCUVA-89088215|EPI_ISL_12860024|Spain|BA.2|2022-04-19	97,37
EPI_ISL_12862245	BA.2.9.5	A	A_14	NIC_NBI_18848|EPI_ISL_12862245|Thailand|BA.2.9.5|2022-05	95,65
EPI_ISL_12863344	BA.2	BA.2	BA.2_1	PT34802|EPI_ISL_12863344|Portugal|BA.2|2022-05-08	99,81
EPI_ISL_12863527	BA.5.1	A	A_3	PT35007|EPI_ISL_12863527|Portugal|BA.5.1|2022-05-09	99,69
EPI_ISL_12865831	BA.4.1	A	A_3	IA-SHL-2097075|EPI_ISL_12865831|USA|BA.4.1|2022-05-09	90,40
EPI_ISL_12867841	BA.2.12.1	BA.2.12.1	BA.2.12.1	CA-CDC-QDX36759603|EPI_ISL_12867841|USA|BA.2.12.1|2022-05-08	99,81
EPI_ISL_12868046	BA.2.12.1	BA.2.12.1	BA.2.12.1	VA-CDC-QDX36758842|EPI_ISL_12868046|USA|BA.2.12.1|2022-05-08	99,81
EPI_ISL_12871289	BA.2	BA.2	BA.2_1	SMC-7067104|EPI_ISL_12871289|Israel|BA.2|2022-05-01	91,47
EPI_ISL_12871692	BA.2.9	BA.2.12.1	BA.2.12.1	SMC-7068308|EPI_ISL_12871692|Israel|BA.2.9|2022-05-04	87,43
EPI_ISL_12873178	BA.2	BA.2	BA.2_1	NC-CHN-01007021|EPI_ISL_12873178|Spain|BA.2|2022-05-06	99,81
EPI_ISL_12877645	BA.1.1	BA.1.1	BA.1.1_2	NJ-PHEL-V22020064|EPI_ISL_12877645|USA|BA.1.1|2022-02-18	91,64
EPI_ISL_12878948	BA.2.12.1	BA.2.12.1	BA.2.12.1	CT-Yale-19599-5|EPI_ISL_12878948|USA|BA.2.12.1|2022-05-05	99,81
EPI_ISL_12879613	BA.2	A	A_14	NW-HHU-26880|EPI_ISL_12879613|Germany|BA.2|2022-05-02	99,81
EPI_ISL_12880889	BA.1.1	BA.1.1	BA.1.1_2	UT-UPHL-220519471671|EPI_ISL_12880889|USA|BA.1.1|2022-01-21	99,75
EPI_ISL_12881097	BA.2.12.1	BA.2.12.1	BA.2.12.1	MD-HP31698-PIDUFCUXLN|EPI_ISL_12881097|USA|BA.2.12.1|2022-05-20	99,81
EPI_ISL_12881474	BA.2	A	A_14	ON-PHL-22-22458|EPI_ISL_12881474|Canada|BA.2|2022-05-16	99,81
EPI_ISL_12882073	BA.1.1	BA.1.1	BA.1.1_2	UT-UPHL-220519297202|EPI_ISL_12882073|USA|BA.1.1|2022-01-25	99,64
EPI_ISL_12882933	BA.2.9	BA.2.12	BA.2.12	NC-MCPH-CLT-007910|EPI_ISL_12882933|USA|BA.2.9|2022-05-10	94,24
EPI_ISL_12884032	BA.2.12.1	BA.2.12.1	BA.2.12.1	NJ-CDC-LC0623950|EPI_ISL_12884032|USA|BA.2.12.1|2022-05-05	87,86
EPI_ISL_12886569	XAA	A	A_14	NJ-CDC-LC0627130|EPI_ISL_12886569|USA|XAA|2022-05-09	99,81
EPI_ISL_12888574	BA.2.12.1	BA.2.12.1	BA.2.12.1	CA-CDC-LC0633305|EPI_ISL_12888574|USA|BA.2.12.1|2022-05-11	99,81
EPI_ISL_12889350	BA.2.12.1	BA.2.12.1	BA.2.12.1	SC-CDC-LC0629521|EPI_ISL_12889350|USA|BA.2.12.1|2022-05-11	99,81
EPI_ISL_12890174	BA.2	BA.2	BA.2_1	CA-CDC-LC0633181|EPI_ISL_12890174|USA|BA.2|2022-05-12	99,81
EPI_ISL_12890936	BA.2.12.1	BA.2.12.1	BA.2.12.1	CA-SEARCH-121498|EPI_ISL_12890936|USA|BA.2.12.1|2022-05-03	96,47
EPI_ISL_12891231	BA.2.3	A	A_14	ND-NDDH-15323|EPI_ISL_12891231|USA|BA.2.3|2022-05-13	90,52
EPI_ISL_12891918	BA.1.17.2	BA.1	BA.1_4	CA-61210750107493|EPI_ISL_12891918|USA|BA.1.17.2|2022-01-16	86,62
EPI_ISL_12891974	BA.1.1	BA.1	BA.1_4	CA-61210800210598|EPI_ISL_12891974|USA|BA.1.1|2022-01-18	90,08
EPI_ISL_12894363	BA.2	A	A_14	PHEC-YYFJCB5|EPI_ISL_12894363|United Kingdom|BA.2|2022-05-09	99,81
EPI_ISL_12895179	BA.2.12.1	BA.2.12.1	BA.2.12.1	SCOT-12487|EPI_ISL_12895179|United Kingdom|BA.2.12.1|2022-05-06	86,11
EPI_ISL_12895205	BA.2.3	BA.2.48	BA.2.48	SCOT-12519|EPI_ISL_12895205|United Kingdom|BA.2.3|2022-04-28	99,81
EPI_ISL_12897126	BA.1.1.2	BA.1.1	BA.1.1_3	TKYkbm18030|EPI_ISL_12897126|Japan|BA.1.1.2|2022-04-08	99,64
EPI_ISL_12897750	BA.1.1	BA.1.1	BA.1.1_3	TKYkbm18135|EPI_ISL_12897750|Japan|BA.1.1|2022-04-11	99,64
EPI_ISL_12898272	BA.2	A	A_14	5251|EPI_ISL_12898272|Singapore|BA.2|2022-05-12	99,75
EPI_ISL_12898331	BA.2.3	BA.2	BA.2_1	5310|EPI_ISL_12898331|Singapore|BA.2.3|2022-05-16	95,65
EPI_ISL_12898421	BA.2.52	A	A_17	UN-NML-419271|EPI_ISL_12898421|Canada|BA.2.52|2022-04-26	99,81
EPI_ISL_12901862	BA.2	BA.2	BA.2_1	PV-HUD-78294315|EPI_ISL_12901862|Spain|BA.2|2022-05-02	99,81
EPI_ISL_12902094	BA.2	A	A_14	SMC-7065376|EPI_ISL_12902094|Israel|BA.2|2022-04-25	99,81
EPI_ISL_12902847	BA.2	BA.2.10	BA.2.10	SMC-7064407|EPI_ISL_12902847|Israel|BA.2|2022-04-19	99,81
EPI_ISL_12905182	BA.2	AY.53	AY.53	AND-255_221111383901-GC|EPI_ISL_12905182|Andorra|BA.2|2022-04-08	70,56
EPI_ISL_12907779	BA.1.1	BA.1	BA.1_4	CA-CDPH-3000338517|EPI_ISL_12907779|USA|BA.1.1|2022-02-10	78,65
EPI_ISL_12910580	BA.2	BA.2	BA.2_1	MA-CDCBI-CRSP_BHA32TPEL5SVZYIR|EPI_ISL_12910580|USA|BA.2|2022-05-08	94,26
EPI_ISL_12911807	BA.2	BA.2	BA.2_1	IMR_WC196390|EPI_ISL_12911807|Malaysia|BA.2|2022-03-17	99,81
EPI_ISL_12913004	BA.1.15	BA.1	BA.1_2	WY-WYPHL-22005607|EPI_ISL_12913004|USA|BA.1.15|2022-01-10	99,47
EPI_ISL_12915021	BA.2.9	A	A_1	VA-Curative-327943|EPI_ISL_12915021|USA|BA.2.9|2022-05-12	77,16
EPI_ISL_12915258	BA.2.12.1	BA.2.12.1	BA.2.12.1	DC-Curative-363898|EPI_ISL_12915258|USA|BA.2.12.1|2022-05-14	90,48
EPI_ISL_12917277	BA.2.12.1	BA.2.12.1	BA.2.12.1	AZ-ASU75655|EPI_ISL_12917277|USA|BA.2.12.1|2022-05-18	99,81
EPI_ISL_12918150	BA.2	BA.2	BA.2_1	PHEC-YYFJH4A|EPI_ISL_12918150|United Kingdom|BA.2|2022-05-09	94,43
EPI_ISL_12918633	BA.2.3	BA.2	BA.2_1	17-037248-KR|EPI_ISL_12918633|Slovenia|BA.2.3|2022-05-12	99,81
EPI_ISL_12919013	BA.2.10.1	BA.2.10.1	BA.2.10.1	IMR_OS177|EPI_ISL_12919013|Malaysia|BA.2.10.1|2022-04-13	99,81
EPI_ISL_12919031	BA.2.3	BA.2	BA.2_1	IMR_OS195|EPI_ISL_12919031|Malaysia|BA.2.3|2022-04-03	99,81
EPI_ISL_12919525	BA.4.1	A	A_3	LSPA-3E2737C|EPI_ISL_12919525|United Kingdom|BA.4.1|2022-05-18	99,69
EPI_ISL_12919813	BA.2	BA.2	BA.2_1	LSPA-3E25341|EPI_ISL_12919813|United Kingdom|BA.2|2022-05-19	99,77
EPI_ISL_12919962	BA.2	A	A_14	LSPA-3E2452E|EPI_ISL_12919962|United Kingdom|BA.2|2022-05-17	99,81
EPI_ISL_12921445	BA.1.1	BA.1	BA.1_4	CA-CDPH-3000338672|EPI_ISL_12921445|USA|BA.1.1|2022-02-09	74,47
EPI_ISL_12922070	BA.1.1	BA.1.1	BA.1.1_2	CA-CDPH-2000062311|EPI_ISL_12922070|USA|BA.1.1|2022-02-15	99,62
EPI_ISL_12925781	BA.2.12.1	BA.2.12.1	BA.2.12.1	CA-CDC-LC0640296|EPI_ISL_12925781|USA|BA.2.12.1|2022-05-11	99,81
EPI_ISL_12926500	BA.2	A	A_14	WA-CDC-LC0639126|EPI_ISL_12926500|USA|BA.2|2022-05-12	99,81
EPI_ISL_12926707	BA.2	A	A_14	NH-CDC-LC0637990|EPI_ISL_12926707|USA|BA.2|2022-05-13	99,81
EPI_ISL_12927010	BA.2	A	A_14	NY-CDC-LC0638158|EPI_ISL_12927010|USA|BA.2|2022-05-13	96,58
EPI_ISL_12928388	BA.1.1	BA.1.1	BA.1.1_2	CA-CDPH-3000338419|EPI_ISL_12928388|USA|BA.1.1|2022-02-09	91,36
EPI_ISL_12928696	BA.1.1.18	BA.1.1	BA.1.1_3	CA-CDPH-3000340487|EPI_ISL_12928696|USA|BA.1.1.18|2022-02-10	81,32
EPI_ISL_12929341	BA.1.1	BA.1.1	BA.1.1_2	CA-CDPH-3000323801|EPI_ISL_12929341|USA|BA.1.1|2022-01-31	81,63
EPI_ISL_12929929	BA.1	BA.1.1	BA.1.1_3	SK-RRPL-434391|EPI_ISL_12929929|Canada|BA.1|2022-01-19	99,62
EPI_ISL_12933648	BA.2.12.1	BA.2.12.1	BA.2.12.1	UT-UPHL-220525359103|EPI_ISL_12933648|USA|BA.2.12.1|2022-05-14	99,81
EPI_ISL_12938570	BA.1.1	BA.1.1	BA.1.1_2	PG-232118|EPI_ISL_12938570|Japan|BA.1.1|2022-04-11	99,64
EPI_ISL_12938709	BA.2.3.13	A	A_14	PG-232256|EPI_ISL_12938709|Japan|BA.2.3.13|2022-04-08	99,81
EPI_ISL_12938838	BA.2.3.1	BA.2.3.1	BA.2.3.1	PG-232948|EPI_ISL_12938838|Japan|BA.2.3.1|2022-04-13	99,81
EPI_ISL_12940402	BA.2	A	A_14	PG-237924|EPI_ISL_12940402|Japan|BA.2|2022-04-20	99,81
EPI_ISL_12940848	BA.2.24	BA.2.24	BA.2.24	PG-238853|EPI_ISL_12940848|Japan|BA.2.24|2022-04-27	99,81
EPI_ISL_12942206	BA.2.3	A	A_14	PG-218401|EPI_ISL_12942206|Japan|BA.2.3|2022-03-07	99,81
EPI_ISL_12942400	BA.1.1	BA.1.1	BA.1.1_3	PG-225850|EPI_ISL_12942400|Japan|BA.1.1|2022-03-22	99,64
EPI_ISL_12942707	BA.2.23	A	A_14	IMR_OS364|EPI_ISL_12942707|Malaysia|BA.2.23|2022-04-15	99,81
EPI_ISL_12944307	BA.1.1.2	BA.1	BA.1_2	PG-226861|EPI_ISL_12944307|Japan|BA.1.1.2|2022-03-22	99,64

EPI_ISL_12944587	BA.1.1.2	BA.1.1	BA.1.1_3	PG-223036|EPI_ISL_12944587|Japan|BA.1.1.2|2022-03-25	99,64
EPI_ISL_12947599	BA.2	A	A_17	PG-237174|EPI_ISL_12947599|Japan|BA.2|2022-04-04	99,81
EPI_ISL_12948047	BA.2.3.1	BA.2.3.1	BA.2.3.1	PG-240927|EPI_ISL_12948047|Japan|BA.2.3.1|2022-04-28	99,81
EPI_ISL_12948565	BA.2.10	A	A_14	PG-222990|EPI_ISL_12948565|Japan|BA.2.10|2022-03-27	99,81
EPI_ISL_12949288	BA.1.1.2	BA.1.1	BA.1.1_3	PG-230375|EPI_ISL_12949288|Japan|BA.1.1.2|2022-03-30	99,64
EPI_ISL_12949313	BA.2	A	A_14	PG-230400|EPI_ISL_12949313|Japan|BA.2|2022-04-09	99,81
EPI_ISL_12950108	BA.2.3.13	A	A_14	PG-237485|EPI_ISL_12950108|Japan|BA.2.3.13|2022-04-19	99,81
EPI_ISL_12950723	BA.2.3	A	A_14	PG-226909|EPI_ISL_12950723|Japan|BA.2.3|2022-03-28	99,81
EPI_ISL_12953141	BA.2	BA.2.12	BA.2.12	TG-GMCH-ICMR-INSACOG-CVDKR24001343|EPI_ISL_12953141|India|BA.2|2022-05-05	95,31
EPI_ISL_12954487	BA.2	BA.2.10	BA.2.10	MH-ICMR-NIV-INSACOG-GSEQ-10219|EPI_ISL_12954487|India|BA.2|2022-02-19	80,81
EPI_ISL_12954573	BA.2	BA.2	BA.2_1	MH-ICMR-NIV-INSACOG-GSEQ-9816|EPI_ISL_12954573|India|BA.2|2022-02-12	99,98
EPI_ISL_12957514	BA.2	A	A_17	INEI120363|EPI_ISL_12957514|Argentina|BA.2|2022-04-23	99,81
EPI_ISL_12959906	BA.2.12.1	BA.2.12.1	BA.2.12.1	MD-IGS-122213000770A|EPI_ISL_12959906|USA|BA.2.12.1|2022-05-10	99,81
EPI_ISL_12960217	BA.2.29	BA.2	BA.2_1	JP-33-USAFSAM-S16366|EPI_ISL_12960217|Japan|BA.2.29|2022-05-09	90,94
EPI_ISL_12961326	BA.2	BA.2	BA.2_1	BRE-HMN-22052100372|EPI_ISL_12961326|France|BA.2|2022-04-19	82,92
EPI_ISL_12961348	BA.2	BA.2.12	BA.2.12	BRE-HMN-22052100535|EPI_ISL_12961348|France|BA.2|2022-04-19	71,55
EPI_ISL_12961383	BA.2.36	BA.2	BA.2_1	GES-HMN-22042140127|EPI_ISL_12961383|France|BA.2.36|2022-04-04	95,02
EPI_ISL_12961875	BA.2	A	A_14	CA-HLX-STM-YZPP739QX|EPI_ISL_12961875|USA|BA.2|2022-05-09	99,81
EPI_ISL_12961883	BA.2	A	A_12	CA-HLX-STM-ZZQU4SZCR|EPI_ISL_12961883|USA|BA.2|2022-05-09	99,81
EPI_ISL_12961907	BA.1	BA.1.15	BA.1.15	CA-HLX-STM-4H9GU5YGZ|EPI_ISL_12961907|USA|BA.1|2021-12-31	99,56
EPI_ISL_12962149	BA.2	BA.1.15	BA.1.15	CA-HLX-STM-RJ6KUJDFN|EPI_ISL_12962149|USA|BA.2|2022-05-06	94,81
EPI_ISL_12962867	BA.2.3	A	A_14	CA-HLX-STM-3R3FHNK8X|EPI_ISL_12962867|USA|BA.2.3|2022-05-03	99,81
EPI_ISL_12964882	BA.2	BA.2.10	BA.2.10	CA-HLX-STM-2868FZU6U|EPI_ISL_12964882|USA|BA.2|2022-04-25	91,51
EPI_ISL_12965448	BA.1.1	BA.1.1	BA.1.1_2	CA-HLX-STM-PTJUQCPG3|EPI_ISL_12965448|USA|BA.1.1|2021-12-28	99,62
EPI_ISL_12966226	BA.2	A	A_14	BC-BCCDC-429304|EPI_ISL_12966226|Canada|BA.2|2022-04-22	99,81
EPI_ISL_12967674	BA.2.12.1	BA.2.12.1	BA.2.12.1	BC-BCCDC-431732|EPI_ISL_12967674|Canada|BA.2.12.1|2022-04-30	99,81
EPI_ISL_12970153	BA.2	A	A_14	QEUH-3E1D661|EPI_ISL_12970153|United Kingdom|BA.2|2022-05-17	99,81
EPI_ISL_12971759	BA.2	A	A_1	DC-Curative-328979|EPI_ISL_12971759|USA|BA.2|2022-05-17	81,70
EPI_ISL_12971794	BA.2	BA.2.12	BA.2.12	DC-Curative-321156|EPI_ISL_12971794|USA|BA.2|2022-05-16	85,06
EPI_ISL_12971920	BA.2.12.1	BA.2.12.1	BA.2.12.1	DC-Curative-314378|EPI_ISL_12971920|USA|BA.2.12.1|2022-05-16	87,06
EPI_ISL_12972022	BA.2.12.1	BA.2.12.1	BA.2.12.1	VA-Curative-353082|EPI_ISL_12972022|USA|BA.2.12.1|2022-05-17	93,65
EPI_ISL_12972603	BA.2.3	BA.2	BA.2_1	IMR_WC194253|EPI_ISL_12972603|Malaysia|BA.2.3|2022-03-16	99,81
EPI_ISL_12973104	BA.2	BA.2	BA.2_1	IMR_OS858|EPI_ISL_12973104|Malaysia|BA.2|2022-04-07	99,50
EPI_ISL_12974071	BA.2.12.1	BA.2.12.1	BA.2.12.1	NJ-CDC-LC0645697|EPI_ISL_12974071|USA|BA.2.12.1|2022-05-13	99,81
EPI_ISL_12974718	BA.2	A	A_14	FL-CDC-LC0644716|EPI_ISL_12974718|USA|BA.2|2022-05-14	99,81
EPI_ISL_12979266	BA.2	A	A_14	VIC58115|EPI_ISL_12979266|Australia|BA.2|2022-04-15	94,05
EPI_ISL_12979930	BA.2	A	A_14	VIC59037|EPI_ISL_12979930|Australia|BA.2|2022-05-10	99,81
EPI_ISL_12980408	BA.2	BA.2	BA.2_1	SUS0011776|EPI_ISL_12980408|Sweden|BA.2|2022-05-16	95,44
EPI_ISL_12981536	BA.2.10	A	A_14	15137|EPI_ISL_12981536|Norway|BA.2.10|2022-03-07	99,81
EPI_ISL_12982194	BA.2	A	A_17	NB-60912|EPI_ISL_12982194|Chile|BA.2|2022-05-14	99,77
EPI_ISL_12983981	BA.2.3.1	BA.2.3.1	BA.2.3.1	PG-226538|EPI_ISL_12983981|Japan|BA.2.3.1|2022-04-03	99,81
EPI_ISL_12984313	BA.2.3.1	BA.2.3.1	BA.2.3.1	PG-228579|EPI_ISL_12984313|Japan|BA.2.3.1|2022-03-25	99,81
EPI_ISL_12985458	BA.2.29	BA.2	BA.2_1	PG-222860|EPI_ISL_12985458|Japan|BA.2.29|2022-03-25	99,47
EPI_ISL_12985502	BA.2.3.11	A	A_14	PG-232572|EPI_ISL_12985502|Japan|BA.2.3.11|2022-04-14	99,81
EPI_ISL_12986469	BA.2	BA.2	BA.2_1	PG-232691|EPI_ISL_12986469|Japan|BA.2|2022-04-09	99,81
EPI_ISL_12986651	BA.1.1.2	BA.1.1	BA.1.1_3	PG-219594|EPI_ISL_12986651|Japan|BA.1.1.2|2022-02-27	99,64
EPI_ISL_12987039	BA.2.3	BA.2	BA.2_1	LOMWRU-0073|EPI_ISL_12987039|Laos|BA.2.3|2022-03-22	90,46
EPI_ISL_12987473	BA.2.24	BA.2.24	BA.2.24	PG-240809|EPI_ISL_12987473|Japan|BA.2.24|2022-05-02	99,81
EPI_ISL_12987519	BA.1.1.2	BA.1.1	BA.1.1_3	PG-221591|EPI_ISL_12987519|Japan|BA.1.1.2|2022-01-12	99,64
EPI_ISL_12989198	BA.1.1.2	BA.1.1	BA.1.1_2	PG-216677|EPI_ISL_12989198|Japan|BA.1.1.2|2022-01-21	99,64
EPI_ISL_12991391	BA.2.3	BA.2	BA.2_1	PG-240666|EPI_ISL_12991391|Japan|BA.2.3|2022-05-04	99,81
EPI_ISL_12991985	BA.1.1.2	BA.1.1	BA.1.1_3	PG-222179|EPI_ISL_12991985|Japan|BA.1.1.2|2022-02-04	99,64
EPI_ISL_12992499	BA.1.1.2	BA.1.1	BA.1.1_3	PG-231571|EPI_ISL_12992499|Japan|BA.1.1.2|2022-03-23	99,64
EPI_ISL_12992962	BA.1.1.2	BA.1.1	BA.1.1_3	PG-226798|EPI_ISL_12992962|Japan|BA.1.1.2|2022-02-25	99,64
EPI_ISL_12994337	BA.2.56	BA.2.56	BA.2.56	DCGC-520164|EPI_ISL_12994337|Denmark|BA.2.56|2022-05-21	99,81
EPI_ISL_12994375	BA.2	BA.2	BA.2_1	DCGC-520202|EPI_ISL_12994375|Denmark|BA.2|2022-05-10	99,73
EPI_ISL_12994594	BA.2.9	A	A_17	DCGC-520421|EPI_ISL_12994594|Denmark|BA.2.9|2022-05-22	99,81
EPI_ISL_12994802	BA.2	A	A_17	DCGC-520630|EPI_ISL_12994802|Denmark|BA.2|2022-05-09	99,81
EPI_ISL_12997647	BA.2.9	A	A_17	BW-RKI-I-820033|EPI_ISL_12997647|Germany|BA.2.9|2022-05-13	99,81
EPI_ISL_12998371	BA.2	BA.2	BA.2_1	SH-RKI-I-820796|EPI_ISL_12998371|Germany|BA.2|2022-05-09	99,77
EPI_ISL_12998619	BA.2	BA.2	BA.2_1	NW-RKI-I-821477|EPI_ISL_12998619|Germany|BA.2|2022-03-18	95,12
EPI_ISL_12999427	BA.2	BA.2	BA.2_1	EDB61787|EPI_ISL_12999427|United Kingdom|BA.2|2022-05-03	99,81
EPI_ISL_13000059	BA.2.23	BA.2.10	BA.2.10	PHEC-YYFKIGG|EPI_ISL_13000059|United Kingdom|BA.2.23|2022-05-11	95,17
EPI_ISL_13001050	BA.2	BA.2	BA.2_1	BY-RKI-I-822997|EPI_ISL_13001050|Germany|BA.2|2022-02-15	99,68
EPI_ISL_13001054	BA.1.1	BA.1.1	BA.1.1_2	SN-RKI-I-823000|EPI_ISL_13001054|Germany|BA.1.1|2022-02-15	99,60
EPI_ISL_13001512	BA.1.1	BA.1.1	BA.1.1_2	SN-RKI-I-823237|EPI_ISL_13001512|Germany|BA.1.1|2022-02-15	99,62
EPI_ISL_13002395	BA.2	BA.2	BA.2_1	CT-HUJT-RB32743|EPI_ISL_13002395|Spain|BA.2|2022-05-11	99,81
EPI_ISL_13002993	BA.2.9.3	BA.2.9.3	BA.2.9.3	ARA-CFD700000297336|EPI_ISL_13002993|France|BA.2.9.3|2022-05-23	99,81
EPI_ISL_13004156	BA.2	BA.2	BA.2_1	BY-RKI-I-825911|EPI_ISL_13004156|Germany|BA.2|2022-05-09	99,81
EPI_ISL_13009063	BA.1.17.2	BA.1.17.2	BA.1.17.2	BY-RKI-I-827801|EPI_ISL_13009063|Germany|BA.1.17.2|2022-02-10	99,60
EPI_ISL_13012316	BA.1	BA.1	BA.1_3	SN-RKI-I-830331|EPI_ISL_13012316|Germany|BA.1|2022-02-09	99,62
EPI_ISL_13012574	BA.2	BA.2	BA.2_1	NW-RKI-I-830676|EPI_ISL_13012574|Germany|BA.2|2022-04-07	95,21
EPI_ISL_13012749	BA.2.68	A	A_14	NW-RKI-I-830974|EPI_ISL_13012749|Germany|BA.2.68|2022-05-19	98,93
EPI_ISL_13013777	BA.2	A	A_17	BY-RKI-I-832014|EPI_ISL_13013777|Germany|BA.2|2022-05-20	99,81
EPI_ISL_13014799	BA.1.1	BA.1.1	BA.1.1_2	NW-RKI-I-833264|EPI_ISL_13014799|Germany|BA.1.1|2022-02-12	99,16
EPI_ISL_13016821	BA.2	A	A_1	BFC-HMN-22052160315|EPI_ISL_13016821|France|BA.2|2022-05-09	67,45
EPI_ISL_13016844	BA.2	A	A_17	CVL-HMN-22052111302|EPI_ISL_13016844|France|BA.2|2022-05-02	99,81
EPI_ISL_13018231	BA.1.17.2	BA.1.17.2	BA.1.17.2	LAZ-ID762UTV|EPI_ISL_13018231|Italy|BA.1.17.2|2022-01-26	95,46
EPI_ISL_13019148	BA.2	A	A_14	PT35538|EPI_ISL_13019148|Portugal|BA.2|2022-05-14	99,81
EPI_ISL_13019406	BA.1.15	BA.1	BA.1_3	BA-FIOCRUZ-10047|EPI_ISL_13019406|Brazil|BA.1.15|2022-05-11	99,64
EPI_ISL_13021253	BA.2.12.1	BA.2.12.1	BA.2.12.1	OH-PLMI-HMNY7-10095|EPI_ISL_13021253|USA|BA.2.12.1|2022	89,75
EPI_ISL_13021574	BA.2.9	A	A_17	CO-CDPHE-2103223147|EPI_ISL_13021574|USA|BA.2.9|2022-05-12	99,73
EPI_ISL_13022102	BA.2.60	BA.2	BA.2_1	LIM-INS-17291|EPI_ISL_13022102|Peru|BA.2.60|2022-05-10	99,81
EPI_ISL_13023925	BA.2	A	A_14	KDCA45310|EPI_ISL_13023925|South Korea|BA.2|2022-04-29	99,81
EPI_ISL_13025338	BA.2	BA.2	BA.2_1	KDCA59943|EPI_ISL_13025338|South Korea|BA.2|2022-05-11	99,81
EPI_ISL_13027579	BA.2	BA.2	BA.2_1	IC-10350|EPI_ISL_13027579|Japan|BA.2|2022-05-15	99,81
EPI_ISL_13029181	BA.2.9	A	A_17	GA-CHUVI-34739727|EPI_ISL_13029181|Spain|BA.2.9|2022-05-01	99,81
EPI_ISL_13030285	BA.2.9	A	A_17	DCGC-521411|EPI_ISL_13030285|Denmark|BA.2.9|2022-02-18	99,81
EPI_ISL_13030948	BA.2.56	BA.2.56	BA.2.56	LNS0938020|EPI_ISL_13030948|Luxembourg|BA.2.56|2022-05-16	99,81
EPI_ISL_13031036	BA.2.14	BA.2.14	BA.2.14	LNS7294790|EPI_ISL_13031036|Luxembourg|BA.2.14|2022-05-05	99,81
EPI_ISL_13031052	BA.2.56	BA.2.56	BA.2.56	LNS8447924|EPI_ISL_13031052|Luxembourg|BA.2.56|2022-05-16	99,81
EPI_ISL_13031343	BA.2	BA.2	BA.2_2	LNS5256956|EPI_ISL_13031343|Luxembourg|BA.2|2022-05-16	99,81
EPI_ISL_13033431	BA.2	BA.2.13	BA.2.13	FL-BPHL-7925|EPI_ISL_13033431|USA|BA.2|2022-05-06	99,81
EPI_ISL_13034712	BA.2	BA.2	BA.2_1	NW-RKI-I-836544|EPI_ISL_13034712|Germany|BA.2|2022-05-11	99,81
EPI_ISL_13035502	BA.5.3.2	BA.5.3.2	BA.5.3.2	BY-RKI-I-837803|EPI_ISL_13035502|Germany|BA.5.3.2|2022-05-18	99,69
EPI_ISL_13035779	BA.2	BA.2	BA.2_1	SN-RKI-I-838088|EPI_ISL_13035779|Germany|BA.2|2022-04-19	90,36
EPI_ISL_13036049	BA.2	A	A_14	ST-RKI-I-838361|EPI_ISL_13036049|Germany|BA.2|2022-05-03	99,81
EPI_ISL_13036162	BA.2	A	A_17	SN-RKI-I-838477|EPI_ISL_13036162|Germany|BA.2|2022-04-11	90,35
EPI_ISL_13036802	BA.1.1.18	BA.1.1	BA.1.1_3	CA-CDPH-7000011377|EPI_ISL_13036802|USA|BA.1.1.18|2022-02-06	99,64
EPI_ISL_13037615	BA.2.9	A	A_17	UMTM483258|EPI_ISL_13037615|Czech Republic|BA.2.9|2022-05-04	99,81
EPI_ISL_13037802	BA.2.12.1	BA.2.12.1	BA.2.12.1	MN-MDH-25224|EPI_ISL_13037802|USA|BA.2.12.1|2022-05-11	99,79

EPI_ISL_13038900	BA.2.12.1	BA.2.12.1	BA.2.12.1	NY-SUNYQB-61020060703944|EPI_ISL_13038900|USA|BA.2.12.1|2022-04-07	99,81
EPI_ISL_13039625	BA.2.12.1	BA.2.12.1	BA.2.12.1	NY-SUNYQB-61210550408580|EPI_ISL_13039625|USA|BA.2.12.1|2022-04-29	99,81
EPI_ISL_13040231	BA.2.9	BA.2	BA.2_1	CA-OC-2663|EPI_ISL_13040231|USA|BA.2.9|2022-05-10	99,27
EPI_ISL_13041226	BA.2	BA.2	BA.2_1	NY-PRL-220519_03J10|EPI_ISL_13041226|USA|BA.2|2022-05-17	99,81
EPI_ISL_13041825	BA.2.12.1	BA.2.12.1	BA.2.12.1	NY-PRL-220519_02K05|EPI_ISL_13041825|USA|BA.2.12.1|2022-05-17	99,81
EPI_ISL_13042697	BA.2.12.1	BA.2.12.1	BA.2.12.1	NY-PRL-220520_02F10|EPI_ISL_13042697|USA|BA.2.12.1|2022-05-18	99,81
EPI_ISL_13044776	BA.2.12.1	BA.2.12.1	BA.2.12.1	NY-PRL-220522_02I11|EPI_ISL_13044776|USA|BA.2.12.1|2022-05-20	99,81
EPI_ISL_13045942	BA.2	BA.2	BA.2_1	SA292540|EPI_ISL_13045942|Australia|BA.2|2022-05-14	99,81
EPI_ISL_13046332	BA.2.12.1	BA.2.12.1	BA.2.12.1	MI-UM-10045422694|EPI_ISL_13046332|USA|BA.2.12.1|2022-05-09	99,81
EPI_ISL_13046970	BA.2.9	A	A_17	THL-202213397|EPI_ISL_13046970|Finland|BA.2.9|2022-05-07	99,81
EPI_ISL_13048175	BA.5.1	A	A_3	LSPA-3E30D45|EPI_ISL_13048175|United Kingdom|BA.5.1|2022-05-25	99,69
EPI_ISL_13049161	BA.2.9	A	A_17	DCGC-521577|EPI_ISL_13049161|Denmark|BA.2.9|2022-05-25	99,81
EPI_ISL_13049914	BA.2	A	A_17	DCGC-522343|EPI_ISL_13049914|Denmark|BA.2|2022-05-23	99,81
EPI_ISL_13051185	BA.2	A	A_17	NW-HHU-27510|EPI_ISL_13051185|Germany|BA.2|2022-05-19	99,81
EPI_ISL_13052074	BA.2	A	A_17	UGent-3793|EPI_ISL_13052074|Belgium|BA.2|2022-04-07	99,81
EPI_ISL_13052136	BA.2	A	A_14	UGent-3858|EPI_ISL_13052136|Belgium|BA.2|2022-05-03	99,81
EPI_ISL_13052768	BA.2.12.1	BA.2.12.1	BA.2.12.1	RI-RISHL-E04382|EPI_ISL_13052768|USA|BA.2.12.1|2022-05-11	99,81
EPI_ISL_13053097	BA.2	BA.1	BA.1_3	DC-Curative-458616|EPI_ISL_13053097|USA|BA.2|2022-05-20	92,06
EPI_ISL_13055949	BA.1.17.2	BA.1.17.2	BA.1.17.2	CAM-TIGEM-IZSM-COLLI-31018|EPI_ISL_13055949|Italy|BA.1.17.2|2022-02-05	97,77
EPI_ISL_13059321	BA.2	A	A_14	NRL_s3099|EPI_ISL_13059321|Czech Republic|BA.2|2022-04-29	98,51
EPI_ISL_13061054	BA.2.18	BA.2.18	BA.2.18	CA-CDC-QDX36866889|EPI_ISL_13061054|USA|BA.2.18|2022-05-09	99,81
EPI_ISL_13062304	BA.2	A	A_14	CA-CDC-QDX36867028|EPI_ISL_13062304|USA|BA.2|2022-05-10	99,81
EPI_ISL_13063703	BA.2	BA.2.3.6	BA.2.3.6	KS-CDC-QDX36901041|EPI_ISL_13063703|USA|BA.2|2022-05-10	99,81
EPI_ISL_13064224	BA.2.12.1	BA.2.12.1	BA.2.12.1	NJ-CDC-QDX36964492|EPI_ISL_13064224|USA|BA.2.12.1|2022-05-13	99,81
EPI_ISL_13066118	BA.2	A	A_17	RC0702666|EPI_ISL_13066118|Estonia|BA.2|2022-05-09	98,30
EPI_ISL_13066613	BA.2	A	A_4	PHWC-PJHXFM|EPI_ISL_13066613|United Kingdom|BA.2|2022-05-11	99,54
EPI_ISL_13067377	BA.2	A	A_14	DCGC-523162|EPI_ISL_13067377|Denmark|BA.2|2022-05-28	99,81
EPI_ISL_13067751	BA.2	BA.2	BA.2_1	DCGC-523537|EPI_ISL_13067751|Denmark|BA.2|2022-05-27	99,81
EPI_ISL_13069307	BA.1.1	BA.1	BA.1_4	BFC-HMN-22042260651|EPI_ISL_13069307|France|BA.1.1|2022-02-21	98,15
EPI_ISL_13069577	BA.2	BA.2	BA.2_1	GES-HMN-22042290419|EPI_ISL_13069577|France|BA.2|2022-04-25	95,75
EPI_ISL_13069780	BA.2	BA.2.12	BA.2.12	OCC-ChuMtp-99221191816|EPI_ISL_13069780|France|BA.2|2022-04-29	97,73
EPI_ISL_13070986	BA.2.12.1	BA.2.12.1	BA.2.12.1	NJ-CDC-STM-GRP8S3UHJ|EPI_ISL_13070986|USA|BA.2.12.1|2022-05-19	99,81
EPI_ISL_13071568	BA.1	BA.1	BA.1_1	BA-LACENBA-292436213|EPI_ISL_13071568|Brazil|BA.1|2022-01-21	99,18
EPI_ISL_13072391	BA.2.3	A	A_14	AL-CDC-STM-H43JSE5AZ|EPI_ISL_13072391|USA|BA.2.3|2022-05-22	99,81
EPI_ISL_13072520	BA.2	A	A_17	ICH-741226269|EPI_ISL_13072520|Israel|BA.2|2022-05-23	99,81
EPI_ISL_13072712	BA.2	A	A_14	ICH-741226395|EPI_ISL_13072712|Israel|BA.2|2022-05-22	99,81
EPI_ISL_13073184	BA.2	A	A_14	IN-CDC-LC0658142|EPI_ISL_13073184|USA|BA.2|2022-05-13	99,81
EPI_ISL_13073332	BA.2.12.1	BA.2.12.1	BA.2.12.1	WA-CDC-LC0658321|EPI_ISL_13073332|USA|BA.2.12.1|2022-05-16	99,81
EPI_ISL_13073373	BA.2	A	A_14	WA-CDC-LC0658661|EPI_ISL_13073373|USA|BA.2|2022-05-16	99,81
EPI_ISL_13074174	BA.5.5	BA.5.5	BA.5.5_1	NC-CDC-LC0656344|EPI_ISL_13074174|USA|BA.5.5|2022-05-18	99,69
EPI_ISL_13078230	BA.2.12.1	BA.2.12.1	BA.2.12.1	KS-KSU-2680|EPI_ISL_13078230|USA|BA.2.12.1|2022-05-10	99,81
EPI_ISL_13080646	BA.5.5	BA.5.5	BA.5.5_1	TX-CDC-LC0659551|EPI_ISL_13080646|USA|BA.5.5|2022-05-23	99,69
EPI_ISL_13081543	BA.2	BA.2	BA.2_1	37461-LNS|EPI_ISL_13081543|Guatemala|BA.2|2022-04-19	99,81
EPI_ISL_13084268	BA.2	BA.2	BA.2_1	PAC-IHU-077007_Nova1|EPI_ISL_13084268|France|BA.2|2022-04	98,89
EPI_ISL_13084756	BA.2.56	BA.2.56	BA.2.56	PAC-IHU-077504_Nova1|EPI_ISL_13084756|France|BA.2.56|2022-04	98,80
EPI_ISL_13084810	BA.2	A	A_17	PAC-IHU-077558_Nova1|EPI_ISL_13084810|France|BA.2|2022-04	98,89
EPI_ISL_13086705	BA.2.12.1	BA.2.12.1	BA.2.12.1	MD-HP32047-PIDWUHFVRY|EPI_ISL_13086705|USA|BA.2.12.1|2022-05-21	99,81
EPI_ISL_13099626	BA.2	A	A_14	NV-SNPHL-612149|EPI_ISL_13099626|USA|BA.2|2022-05-16	95,75
EPI_ISL_13100777	BA.2	A	A_4	NM-NMDOH-2022072263|EPI_ISL_13100777|USA|BA.2|2022-05-05	96,36
EPI_ISL_13100846	BA.2.3	A	A_14	NM-NMDOH-2022073530|EPI_ISL_13100846|USA|BA.2.3|2022-04-26	99,81
EPI_ISL_13103278	BA.2.3	A	A_14	AB-ABPHL-66652|EPI_ISL_13103278|Canada|BA.2.3|2022-05-05	90,52
EPI_ISL_13105981	BA.2.9	BA.2	BA.2_2	VA-CAV_VAS3N_00010615_01|EPI_ISL_13105981|USA|BA.2.9|2022-05	89,75
EPI_ISL_13106358	BA.2.12.1	BA.2.12.1	BA.2.12.1	VA-CAV_VAS3N_00010153_01|EPI_ISL_13106358|USA|BA.2.12.1|2022-04	96,34
EPI_ISL_13107766	BA.5.1	A	A_3	IB-HUSE-05510|EPI_ISL_13107766|Spain|BA.5.1|2022-05-25	99,69
EPI_ISL_13108313	BA.2	BA.2	BA.2_1	PHEC-YYFCUW8|EPI_ISL_13108313|United Kingdom|BA.2|2022-05-18	99,77
EPI_ISL_13108882	BA.5.1	A	A_3	PT36149|EPI_ISL_13108882|Portugal|BA.5.1|2022-05-24	99,69
EPI_ISL_13110108	BA.2.23	BA.2.12	BA.2.12	SMC-7069503|EPI_ISL_13110108|Israel|BA.2.23|2022-05-10	87,48
EPI_ISL_13112523	BA.2	BA.2	BA.2_1	SMC-7070737|EPI_ISL_13112523|Israel|BA.2|2022-05-16	89,58
EPI_ISL_13112846	BA.2	A	A_14	HSGM-IST-00265|EPI_ISL_13112846|Turkey|BA.2|2022-04-24	99,81
EPI_ISL_13112897	BA.2	BA.2	BA.2_1	HSGM-IST-00441|EPI_ISL_13112897|Turkey|BA.2|2022-05-09	99,75
EPI_ISL_13113108	BA.2	BA.2	BA.2_2	HSGM-IST-01481|EPI_ISL_13113108|Turkey|BA.2|2022-05-25	99,81
EPI_ISL_13113456	BA.2	BA.2	BA.2_1	SMC-7070764|EPI_ISL_13113456|Israel|BA.2|2022-05-17	72,20
EPI_ISL_13115016	BA.2.12.1	BA.2.12.1	BA.2.12.1	FL-CDC-QDX37055506|EPI_ISL_13115016|USA|BA.2.12.1|2022-05-14	99,81
EPI_ISL_13115654	BA.2.12.1	BA.2.12.1	BA.2.12.1	MI-CDC-QDX37056460|EPI_ISL_13115654|USA|BA.2.12.1|2022-05-16	99,81
EPI_ISL_13116446	BA.2.12.1	BA.2.12.1	BA.2.12.1	CA-CDC-QDX37010212|EPI_ISL_13116446|USA|BA.2.12.1|2022-05-14	99,81
EPI_ISL_13117267	BA.2.12.1	BA.2.12.1	BA.2.12.1	NJ-CDC-QDX37143954|EPI_ISL_13117267|USA|BA.2.12.1|2022-05-18	99,81
EPI_ISL_13117523	BA.2.12.1	BA.2.12.1	BA.2.12.1	NJ-CDC-LC0663231|EPI_ISL_13117523|USA|BA.2.12.1|2022-05-15	81,59
EPI_ISL_13117820	BA.2	A	A_14	CT-CDC-LC0662875|EPI_ISL_13117820|USA|BA.2|2022-05-16	99,81
EPI_ISL_13119038	BA.2.9	XAM	XAM	AZ-TG1353906|EPI_ISL_13119038|USA|BA.2.9|2022-04-27	89,49
EPI_ISL_13122045	BA.2	A	A_17	NY-PRL-220526_00K15|EPI_ISL_13122045|USA|BA.2|2022-05-24	99,81
EPI_ISL_13125938	BA.1.1	BA.1.1	BA.1.1_2	CA-CDPH-2000060010|EPI_ISL_13125938|USA|BA.1.1|2022-01-31	99,01
EPI_ISL_13127064	BA.2.29	A	A_14	TKYkbm19958|EPI_ISL_13127064|Japan|BA.2.29|2022-04-18	99,81
EPI_ISL_13127098	BA.2.10	BA.2	BA.2_1	TKYkbm19992|EPI_ISL_13127098|Japan|BA.2.10|2022-04-18	99,81
EPI_ISL_13127192	BA.2.3.1	BA.2.3.1	BA.2.3.1	TKYkbm20086|EPI_ISL_13127192|Japan|BA.2.3.1|2022-04-25	99,81
EPI_ISL_13127386	BA.2.3	BA.2	BA.2_1	TKYkbm20286|EPI_ISL_13127386|Japan|BA.2.3|2022-04-25	99,81
EPI_ISL_13128182	BA.2	BA.2	BA.2_1	CT-YPL22-34835|EPI_ISL_13128182|USA|BA.2|2022-05-26	98,87
EPI_ISL_13130433	BA.2	BA.2	BA.2_1	QEUH-3E3729A|EPI_ISL_13130433|United Kingdom|BA.2|2022-04-19	99,81
EPI_ISL_13131941	BA.2	BA.2.12	BA.2.12	VAC-CSB-94538010|EPI_ISL_13131941|Colombia|BA.2|2022-03-17	69,87
EPI_ISL_13133125	BA.2.9	A	A_17	ZH-EMC-6119|EPI_ISL_13133125|Netherlands|BA.2.9|2022-05-27	95,94
EPI_ISL_13135178	BA.2	A	A_14	ON-PHL-22-23187|EPI_ISL_13135178|Canada|BA.2|2022-05-16	99,81
EPI_ISL_13135966	BA.2.12.1	BA.2.12.1	BA.2.12.1	MA-NEIDL-06674|EPI_ISL_13135966|USA|BA.2.12.1|2022-05-02	99,81
EPI_ISL_13140105	BA.2	BA.2.12	BA.2.12	Austria|EPI_ISL_13140105|Austria|BA.2|2022-05-17	95,25
EPI_ISL_13140110	BA.2	BA.2	BA.2_1	Austria|EPI_ISL_13140110|Austria|BA.2|2022-05-18	95,23
EPI_ISL_13140540	BA.2	BA.2	BA.2_1	HK-HKPU-PUU038205377|EPI_ISL_13140540|Hong Kong|BA.2|2022-05-24	99,69
EPI_ISL_13141446	BA.1.1	BA.1.1	BA.1.1_2	SN-RKI-I-839083|EPI_ISL_13141446|Germany|BA.1.1|2022-02-28	99,58
EPI_ISL_13141922	BA.4	A	A_9	BW-RKI-I-839845|EPI_ISL_13141922|Germany|BA.4|2022-05-23	99,69
EPI_ISL_13141965	BA.2.36	BA.2	BA.2_2	NW-RKI-I-839898|EPI_ISL_13141965|Germany|BA.2.36|2022-05-23	99,81
EPI_ISL_13142651	BA.2.3	A	A_14	SP-IB_157550|EPI_ISL_13142651|Brazil|BA.2.3|2022-05-18	99,81
EPI_ISL_13142716	BA.2	BA.2	BA.2_1	NW-RKI-I-840045|EPI_ISL_13142716|Germany|BA.2|2022-05-26	99,81
EPI_ISL_13146392	BA.2.12.1	BA.2.12.1	BA.2.12.1	NJ-CDC-STM-8B47ARRJC|EPI_ISL_13146392|USA|BA.2.12.1|2022-05-24	99,81
EPI_ISL_13146397	BA.2.12.1	BA.2.12.1	BA.2.12.1	GA-CDC-STM-7XC3SFNHR|EPI_ISL_13146397|USA|BA.2.12.1|2022-05-24	99,81
EPI_ISL_13146756	BA.2.12.1	BA.2.12.1	BA.2.12.1	TX-CDC-STM-PG84GQQ94|EPI_ISL_13146756|USA|BA.2.12.1|2022-05-25	99,81
EPI_ISL_13147472	BA.2.12.1	BA.2.12.1	BA.2.12.1	TN-SPHL-3208|EPI_ISL_13147472|USA|BA.2.12.1|2022-05-23	99,81
EPI_ISL_13147708	BA.2	BA.2	BA.2_1	LD-Enfer-250522010_H5|EPI_ISL_13147708|Ireland|BA.2|2022-05-25	99,81
EPI_ISL_13147758	BA.2	BA.2	BA.2_1	LD-Enfer-280522003_B2|EPI_ISL_13147758|Ireland|BA.2|2022-05-28	99,81
EPI_ISL_13149170	BA.2.12.1	BA.2.12.1	BA.2.12.1	VA-CDC-LC0647063|EPI_ISL_13149170|USA|BA.2.12.1|2022-05-16	99,81
EPI_ISL_13149298	BA.5	A	A_3	ILRI_M03291|EPI_ISL_13149298|Eswatini|BA.5|2022-04-23	99,68
EPI_ISL_13149397	BA.2.12.1	BA.2.12.1	BA.2.12.1	SC-CDC-LC0647259|EPI_ISL_13149397|USA|BA.2.12.1|2022-05-14	99,81
EPI_ISL_13150779	BA.2.12.1	BA.2.12.1	BA.2.12.1	NJ-CDC-LC0650020|EPI_ISL_13150779|USA|BA.2.12.1|2022-05-18	96,64
EPI_ISL_13151530	BA.2	A	A_4	VA-CDC-LC0651384|EPI_ISL_13151530|USA|BA.2|2022-05-21	99,81
EPI_ISL_13152652	BA.5.5	BA.5.5	BA.5.5_1	IL-CDC-LC0653758|EPI_ISL_13152652|USA|BA.5.5|2022-05-14	99,33

EPI_ISL_13154445	BA.2.12.1	BA.2.12.1	BA.2.12.1	ND-NDDH-16126|EPI_ISL_13154445|USA|BA.2.12.1|2022-06-01	90,52
EPI_ISL_13154768	BA.2	A	A_4	RM-65717|EPI_ISL_13154768|Chile|BA.2|2022-05-26	99,79
EPI_ISL_13155258	BA.2.12.1	BA.2.12.1	BA.2.12.1	TA-64325|EPI_ISL_13155258|Chile|BA.2.12.1|2022-05-23	99,77
EPI_ISL_13156096	BA.1.1	BA.1.1	BA.1.1_2	WA-PHL-021325|EPI_ISL_13156096|USA|BA.1.1|2022-03-12	99,64
EPI_ISL_13156876	BE.1	A	A_3	QEUH-3E3BD2C|EPI_ISL_13156876|United Kingdom|BE.1|2022-05-31	99,69
EPI_ISL_13158668	BA.2.10	BA.2	BA.2_1	KA-SLS-36050|EPI_ISL_13158668|India|BA.2.10|2022-05-06	90,56
EPI_ISL_13159849	BA.4.1	A	A_3	SU-NHLS_5115|EPI_ISL_13159849|South Africa|BA.4.1|2022-05-26	90,40
EPI_ISL_13159965	BA.2.64	A	A_14	CAM-20220526472_IZSM_COLLI_TIGEM|EPI_ISL_13159965|Italy|BA.2.64|2022-05-19	99,81
EPI_ISL_13161049	BA.2	A	A_17	VA-CDC-LC0667999|EPI_ISL_13161049|USA|BA.2|2022-05-22	99,81
EPI_ISL_13162065	BA.2	BA.2	BA.2_1	CT-CDC-LC0667351|EPI_ISL_13162065|USA|BA.2|2022-05-23	99,81
EPI_ISL_13162547	BA.2.12.1	BA.2.12.1	BA.2.12.1	NC-CDC-LC0668563|EPI_ISL_13162547|USA|BA.2.12.1|2022-05-24	99,81
EPI_ISL_13163077	BA.2.12.1	BA.2.12.1	BA.2.12.1	NC-CDC-LC0668219|EPI_ISL_13163077|USA|BA.2.12.1|2022-05-25	99,81
EPI_ISL_13163506	BA.2	A	A_14	MI-UM-10045579166|EPI_ISL_13163506|USA|BA.2|2022-05-18	99,81
EPI_ISL_13164802	BA.2.12.1	BA.2.12.1	BA.2.12.1	MN-MDH-25824|EPI_ISL_13164802|USA|BA.2.12.1|2022-05-31	99,81
EPI_ISL_13164848	BA.2	BA.2	BA.2_1	MN-MDH-25870|EPI_ISL_13164848|USA|BA.2|2022-05-23	99,81
EPI_ISL_13164854	BA.2.12.1	BA.2.12.1	BA.2.12.1	MN-MDH-25876|EPI_ISL_13164854|USA|BA.2.12.1|2022-05-16	99,81
EPI_ISL_13165091	BA.2	BA.2	BA.2_1	CO-CDPHE-2103226989|EPI_ISL_13165091|USA|BA.2|2022-05-12	99,81
EPI_ISL_13171201	BA.2.3	BA.2	BA.2_1	PH-PGC-110127|EPI_ISL_13171201|Philippines|BA.2.3|2022-01-28	96,18
EPI_ISL_13173086	BA.2.3	BA.2	BA.2_1	PH-PGC-107632|EPI_ISL_13173086|Philippines|BA.2.3|2022-01-11	86,01
EPI_ISL_13174049	BA.2.3	A	A_14	PH-PGC-106257|EPI_ISL_13174049|Philippines|BA.2.3|2022-01-07	99,81
EPI_ISL_13174743	BA.2.3	BA.2	BA.2_1	PH-PGC-104638|EPI_ISL_13174743|Philippines|BA.2.3|2022-01-04	99,81
EPI_ISL_13175065	BA.1.1.15	BA.1.1	BA.1.1_3	PH-PGC-107117|EPI_ISL_13175065|Philippines|BA.1.1.15|2021-12-31	97,04
EPI_ISL_13177036	BA.2	A	A_17	NAQ-HCL722002922901|EPI_ISL_13177036|France|BA.2|2022-05-23	99,16
EPI_ISL_13178372	BA.2	BA.2	BA.2_1	MH-NVRL-ecS22IRL00297710|EPI_ISL_13178372|Ireland|BA.2|2022-04-12	96,30
EPI_ISL_13179590	BA.1	BA.1	BA.1_4	22BG-EU_018027_Pl170|EPI_ISL_13179590|Bulgaria|BA.1|2022-02-08	98,97
EPI_ISL_13180390	BA.1.17	BA.1	BA.1_4	22BG-EU_018968_Pl179|EPI_ISL_13180390|Bulgaria|BA.1.17|2022-02-14	99,64
EPI_ISL_13181676	BA.2	A	A_14	21520|EPI_ISL_13181676|Norway|BA.2|2022-04-19	98,44
EPI_ISL_13184436	BA.1.1	BA.1	BA.1_4	DA10332105|EPI_ISL_13184436|Sweden|BA.1.1|2022-02-16	98,70
EPI_ISL_13184622	BA.2	BA.1	BA.1_4	M053763|EPI_ISL_13184622|Sweden|BA.2|2022-05-02	96,18
EPI_ISL_13184736	BA.2	BA.2	BA.2_1	261756170665|EPI_ISL_13184736|Sweden|BA.2|2022-05-08	94,31
EPI_ISL_13187483	BA.2.12.1	BA.2.12.1	BA.2.12.1	CA-CDC-STM-FD72FMTAT|EPI_ISL_13187483|USA|BA.2.12.1|2022-05-25	99,81
EPI_ISL_13188442	BA.2.12.1	BA.2.12.1	BA.2.12.1	CA-CDC-STM-ZTEFFW4BE|EPI_ISL_13188442|USA|BA.2.12.1|2022-05-27	99,81
EPI_ISL_13191223	BA.2	A	A_14	FL-CDC-STM-8B5ZJJE3U|EPI_ISL_13191223|USA|BA.2|2022-05-31	99,79
EPI_ISL_13191718	BA.1.1	BA.1.1	BA.1.1_2	FL-BPHL-8610|EPI_ISL_13191718|USA|BA.1.1|2022-01-15	96,03
EPI_ISL_13191939	BA.1.1	BA.1.1	BA.1.1_2	FL-BPHL-8833|EPI_ISL_13191939|USA|BA.1.1|2022-01-06	91,30
EPI_ISL_13192152	BA.2.18	BA.2.18	BA.2.18	CHH_InDRE_FB14290_E08316895935_S13415|EPI_ISL_13192152|Mexico|BA.2.18|2022-05-18	99,81
EPI_ISL_13192317	BA.2.12.1	BA.2.12.1	BA.2.12.1	NY-SUNYQB-61210553611119|EPI_ISL_13192317|USA|BA.2.12.1|2022-05-12	95,86
EPI_ISL_13192430	BA.2.12.1	BA.2.12.1	BA.2.12.1	NY-SUNYQB-61210501807425|EPI_ISL_13192430|USA|BA.2.12.1|2022-05-06	99,81
EPI_ISL_13193888	BA.2	BA.2	BA.2_1	CA-HLX-STM-FXUJ5BH7Q|EPI_ISL_13193888|USA|BA.2|2022-05-16	99,81
EPI_ISL_13194928	BA.2	BA.2	BA.2_1	MA-CDCBI-CRSP_JGWVEW4FDQTCINBB|EPI_ISL_13194928|USA|BA.2|2022-05-12	97,58
EPI_ISL_13198073	BA.2.12.1	BA.2.12.1	BA.2.12.1	NY-NYCPHL-011783|EPI_ISL_13198073|USA|BA.2.12.1|2022-04-28	98,44
EPI_ISL_13198097	BA.2.12.1	BA.2.12.1	BA.2.12.1	NY-NYCPHL-011896|EPI_ISL_13198097|USA|BA.2.12.1|2022-05-03	99,81
EPI_ISL_13200774	BA.2.3	BA.2	BA.2_1	KDCA66857|EPI_ISL_13200774|South Korea|BA.2.3|2022-05-20	99,81
EPI_ISL_13201266	BA.2.9	A	A_17	DCGC-526301|EPI_ISL_13201266|Denmark|BA.2.9|2022-06-05	99,81
EPI_ISL_13201460	BA.2	A	A_4	DCGC-526495|EPI_ISL_13201460|Denmark|BA.2|2022-05-31	99,79
EPI_ISL_13204862	BA.2	BA.2	BA.2_1	PG-244593|EPI_ISL_13204862|Japan|BA.2|2022-05-05	99,81
EPI_ISL_13205271	BA.2	BA.2.56	BA.2.56	PG-245943|EPI_ISL_13205271|Japan|BA.2|2022-05-13	99,81
EPI_ISL_13205493	BA.2.29	BA.2	BA.2_1	PG-248252|EPI_ISL_13205493|Japan|BA.2.29|2022-05-09	99,81
EPI_ISL_13206085	BA.2.10.2	A	A_14	PG-249370|EPI_ISL_13206085|Japan|BA.2.10.2|2022-05-14	99,81
EPI_ISL_13206467	BA.2	BA.2	BA.2_1	SP-IB_158070|EPI_ISL_13206467|Brazil|BA.2|2022-05-24	99,81
EPI_ISL_13207245	BA.2.51	BA.2.51	BA.2.51	PG-252303|EPI_ISL_13207245|Japan|BA.2.51|2022-05-17	99,81
EPI_ISL_13212281	BA.2.56	BA.2.56	BA.2.56	PG-245268|EPI_ISL_13212281|Japan|BA.2.56|2022-05-09	99,81
EPI_ISL_13213223	BA.2.3.11	A	A_14	PG-250544|EPI_ISL_13213223|Japan|BA.2.3.11|2022-05-19	99,81
EPI_ISL_13213288	BA.2.3	BA.2	BA.2_1	PG-251245|EPI_ISL_13213288|Japan|BA.2.3|2022-04-05	99,81
EPI_ISL_13213326	BA.2.29	A	A_14	PG-246745|EPI_ISL_13213326|Japan|BA.2.29|2022-05-02	99,81
EPI_ISL_13213950	BA.2.3.13	BA.2.10	BA.2.10	PG-244136|EPI_ISL_13213950|Japan|BA.2.3.13|2022-04-12	99,81
EPI_ISL_13214031	BA.2.3.13	A	A_4	PG-242241|EPI_ISL_13214031|Japan|BA.2.3.13|2022-04-25	99,81
EPI_ISL_13219404	BA.2	A	A_14	MA-CDC-LC0675598|EPI_ISL_13219404|USA|BA.2|2022-05-26	97,08
EPI_ISL_13224476	BA.2.12.1	BA.2.12.1	BA.2.12.1	MD-CDC-QDX37255143|EPI_ISL_13224476|USA|BA.2.12.1|2022-05-22	99,81
EPI_ISL_13226691	BA.2.12.1	BA.2.12.1	BA.2.12.1	TX-CDC-QDX37298757|EPI_ISL_13226691|USA|BA.2.12.1|2022-05-21	99,81
EPI_ISL_13226940	BA.2.12.1	BA.2.12.1	BA.2.12.1	VA-CDC-QDX37255189|EPI_ISL_13226940|USA|BA.2.12.1|2022-05-22	99,81
EPI_ISL_13227864	BA.2	BA.2	BA.2_1	ACT5650|EPI_ISL_13227864|Australia|BA.2|2022-05-05	99,81
EPI_ISL_13228827	BA.1	BA.1.15	BA.1.15	SC-FIOCRUZ-11765|EPI_ISL_13228827|Brazil|BA.1|2022-02-26	99,64
EPI_ISL_13229001	BA.2	A	A_17	SP-FIOCRUZ-11996|EPI_ISL_13229001|Brazil|BA.2|2022-05-04	99,81
EPI_ISL_13232219	BA.1.15	BA.1.15	BA.1.15	CA-61210701911696|EPI_ISL_13232219|USA|BA.1.15|2022-02-01	76,40
EPI_ISL_13232576	BA.2.20	BA.2.20	BA.2.20	MI-CDC-QDX37301177|EPI_ISL_13232576|USA|BA.2.20|2022-05-23	99,81
EPI_ISL_13233637	BA.2	A	A_14	NJ-CDC-QDX37439013|EPI_ISL_13233637|USA|BA.2|2022-05-27	99,81
EPI_ISL_13234376	BA.1.1	BA.1.1	BA.1.1_2	OH-ODH-SC2043795|EPI_ISL_13234376|USA|BA.1.1|2022-01-25	84,72
EPI_ISL_13235114	BA.5.3	A	A_9	BY-RKI-I-841343|EPI_ISL_13235114|Germany|BA.5.3|2022-05-25	91,93
EPI_ISL_13235125	BA.2	A	A_17	BY-RKI-I-841354|EPI_ISL_13235125|Germany|BA.2|2022-05-26	99,81
EPI_ISL_13235142	BA.5.2	A	A_9	BY-RKI-I-841372|EPI_ISL_13235142|Germany|BA.5.2|2022-05-26	99,69
EPI_ISL_13235794	BA.2	BA.2	BA.2_1	BW-RKI-I-842080|EPI_ISL_13235794|Germany|BA.2|2022-05-27	99,81
EPI_ISL_13236669	BA.1.15	BA.1.15	BA.1.15	BY-RKI-I-843016|EPI_ISL_13236669|Germany|BA.1.15|2022-02-08	99,60
EPI_ISL_13237063	BA.2.9	BA.2	BA.2_1	HH-RKI-I-843427|EPI_ISL_13237063|Germany|BA.2.9|2022-05-20	99,79
EPI_ISL_13237679	BA.1.1	BA.1	BA.1_4	NW-RKI-I-844084|EPI_ISL_13237679|Germany|BA.1.1|2022-01-27	99,52
EPI_ISL_13237907	BA.1.1	BA.1.1	BA.1.1_2	SN-RKI-I-844340|EPI_ISL_13237907|Germany|BA.1.1|2022-01-24	99,60
EPI_ISL_13238946	BA.2	BA.2	BA.2_1	RP-RKI-I-845250|EPI_ISL_13238946|Germany|BA.2|2022-05-17	94,01
EPI_ISL_13239082	BA.2.9	BA.2	BA.2_1	BW-RKI-I-845401|EPI_ISL_13239082|Germany|BA.2.9|2022-05-23	99,73
EPI_ISL_13240577	BA.2	BA.2	BA.2_1	BY-RKI-I-847191|EPI_ISL_13240577|Germany|BA.2|2022-05-12	97,92
EPI_ISL_13242921	BA.2	BA.2.12.1	BA.2.12.1	WA-UW-22053045422|EPI_ISL_13242921|USA|BA.2|2022-05-30	90,25
EPI_ISL_13244762	BA.2	BA.2.12	BA.2.12	NW-RKI-I-849295|EPI_ISL_13244762|Germany|BA.2|2022-06-01	90,42
EPI_ISL_13245094	BA.2	BA.2	BA.2_1	HH-RKI-I-850042|EPI_ISL_13245094|Germany|BA.2|2022-05-25	99,68
EPI_ISL_13246605	BA.2	A	A_17	RP-RKI-I-851693|EPI_ISL_13246605|Germany|BA.2|2022-05-28	99,79
EPI_ISL_13249367	BA.2	BA.2	BA.2_1	HE-RKI-I-855453|EPI_ISL_13249367|Germany|BA.2|2022-05-17	99,64
EPI_ISL_13251231	BA.2	A	A_14	180922|EPI_ISL_13251231|Slovenia|BA.2|2022-03-22	99,81
EPI_ISL_13251707	BA.2.56	BA.2.56	BA.2.56	ULG-26668|EPI_ISL_13251707|Belgium|BA.2.56|2022-05-31	99,81
EPI_ISL_13252283	BA.2.9	A	A_17	FL-BPHL-8977|EPI_ISL_13252283|USA|BA.2.9|2022-05-31	90,52
EPI_ISL_13252691	BA.2.23.1	BA.2.10	BA.2.10	LK-NVRL-S22IRL00335979|EPI_ISL_13252691|Ireland|BA.2.23.1|2022-05-25	99,79
EPI_ISL_13252754	BA.2.12.1	BA.2.12.1	BA.2.12.1	TA-NVRL-S22IRL00323025|EPI_ISL_13252754|Ireland|BA.2.12.1|2022-05-09	99,79
EPI_ISL_13254111	BA.2.38	BA.2.38	BA.2.38	TN-CDFD-W8-711|EPI_ISL_13254111|India|BA.2.38|2022-05-30	99,81
EPI_ISL_13254361	BA.2.12.1	BA.2.12.1	BA.2.12.1	NY-PRL-220603_02H03|EPI_ISL_13254361|USA|BA.2.12.1|2022-05-31	99,81
EPI_ISL_13257015	BA.5.3.2	A	A_3	NY-PRL-220606_05F08|EPI_ISL_13257015|USA|BA.5.3.2|2022-06-05	99,69
EPI_ISL_13257778	BA.2	A	A_14	47-19052022-05|EPI_ISL_13257778|Serbia|BA.2|2022-05-19	99,81
EPI_ISL_13259342	BE.3	A	A_3	TX-CDC-STM-CS6DPNSGV|EPI_ISL_13259342|USA|BE.3|2022-06-02	99,69
EPI_ISL_13259797	BA.2.9	A	A_17	FL-CDC-LC0677945|EPI_ISL_13259797|USA|BA.2.9|2022-05-24	99,81
EPI_ISL_13260220	BA.2.12.1	BA.2.12.1	BA.2.12.1	NJ-CDC-LC0677390|EPI_ISL_13260220|USA|BA.2.12.1|2022-05-27	98,87
EPI_ISL_13260627	BA.2.12.1	B.1.1.228	B.1.1.228	TX-Curative-410851|EPI_ISL_13260627|USA|BA.2.12.1|2022-05-21	65,08
EPI_ISL_13260886	BA.2.12.1	BA.2.12.1	BA.2.12.1	VA-Curative-494897|EPI_ISL_13260886|USA|BA.2.12.1|2022-05-31	90,48
EPI_ISL_13261038	BA.5.5	BA.5.5	BA.5.5_1	NC-CDC-LC0678177|EPI_ISL_13261038|USA|BA.5.5|2022-05-31	99,69
EPI_ISL_13261960	BA.2.12.1	BA.2.12.1	BA.2.12.1	MD-CDC-QDX37298358|EPI_ISL_13261960|USA|BA.2.12.1|2022-05-23	99,81
EPI_ISL_13262343	BA.2	BA.2	BA.2_1	CA-CDC-QDX37346556|EPI_ISL_13262343|USA|BA.2|2022-05-24	99,81

EPI_ISL_13263014	BA.2	BA.2	BA.2_1	ARA-HMN-22042210295|EPI_ISL_13263014|France|BA.2|2022-04-11	87,85
EPI_ISL_13264320	BA.2.3	A	A_14	IA-SHL-2106542|EPI_ISL_13264320|USA|BA.2.3|2022-05-23	90,52
EPI_ISL_13266130	BA.2.9	A	A_17	SH-RKI-I-857768|EPI_ISL_13266130|Germany|BA.2.9|2022-05-16	99,81
EPI_ISL_13266146	BA.2	BA.2.73	BA.2.73	SH-RKI-I-857784|EPI_ISL_13266146|Germany|BA.2|2022-05-23	99,81
EPI_ISL_13266319	BA.2	A	A_14	NW-RKI-I-858017|EPI_ISL_13266319|Germany|BA.2|2022-05-18	99,81
EPI_ISL_13266939	BA.5.1	A	A_9	SH-RKI-I-858761|EPI_ISL_13266939|Germany|BA.5.1|2022-06-07	94,12
EPI_ISL_13268482	BA.5.1	A	A_3	NW-RKI-I-860439|EPI_ISL_13268482|Germany|BA.5.1|2022-06-07	99,69
EPI_ISL_13270238	BA.2.3	A	A_14	TX-HMH-MCoV-99546|EPI_ISL_13270238|USA|BA.2.3|2022-06-04	99,81
EPI_ISL_13270315	BA.2	BA.2	BA.2_1	TX-HMH-MCoV-99120|EPI_ISL_13270315|USA|BA.2|2022-05-31	99,77
EPI_ISL_13270670	BA.2.12.1	BA.2.12.1	BA.2.12.1	ON-PHL-22-23412|EPI_ISL_13270670|Canada|BA.2.12.1|2022-05-26	99,81
EPI_ISL_13271504	BA.2.12.1	BA.2.12.1	BA.2.12.1	MN-MDH-26080|EPI_ISL_13271504|USA|BA.2.12.1|2022-05-18	99,81
EPI_ISL_13271520	BA.2.12.1	BA.2.12.1	BA.2.12.1	MN-MDH-26096|EPI_ISL_13271520|USA|BA.2.12.1|2022-05-24	99,81
EPI_ISL_13271874	BA.2	A	A_14	185739|EPI_ISL_13271874|Slovenia|BA.2|2022-04-01	99,81
EPI_ISL_13274266	BA.2.10	BA.2	BA.2_1	VIC59358|EPI_ISL_13274266|Australia|BA.2.10|2022-05-09	99,81
EPI_ISL_13274408	BA.2	A	A_17	VIC59465|EPI_ISL_13274408|Australia|BA.2|2022-05-11	99,81
EPI_ISL_13274581	BA.2	BA.2	BA.2_1	VIC59587|EPI_ISL_13274581|Australia|BA.2|2022-05-16	99,81
EPI_ISL_13276168	BA.2	BA.2	BA.2_1	VIC61431|EPI_ISL_13276168|Australia|BA.2|2022-05-29	99,81
EPI_ISL_13276499	BA.2	A	A_14	VIC61795|EPI_ISL_13276499|Australia|BA.2|2022-05-29	99,81
EPI_ISL_13277415	BA.5.1	A	A_3	LSPA-3E47A62|EPI_ISL_13277415|United Kingdom|BA.5.1|2022-06-05	99,69
EPI_ISL_13277787	BA.4.1	A	A_3	LSPA-3E40F97|EPI_ISL_13277787|United Kingdom|BA.4.1|2022-06-04	99,69
EPI_ISL_13278050	BA.2.12.1	BA.2.12.1	BA.2.12.1	WA-PHL-021460|EPI_ISL_13278050|USA|BA.2.12.1|2022-06-01	99,39
EPI_ISL_13278559	BA.2	BA.2	BA.2_1	CO-CDPHE-2103260412|EPI_ISL_13278559|USA|BA.2|2022-05-21	99,81
EPI_ISL_13280875	BA.5.2	A	A_3	DCGC-527470|EPI_ISL_13280875|Denmark|BA.5.2|2022-06-04	99,69
EPI_ISL_13281024	BA.2	BA.2	BA.2_1	DCGC-527619|EPI_ISL_13281024|Denmark|BA.2|2022-06-07	99,81
EPI_ISL_13281829	BA.1	BA.1	BA.1_3	KZY-NRL-S1484|EPI_ISL_13281829|Kazakhstan|BA.1|2022-04-12	99,60
EPI_ISL_13285448	BA.2.12.1	BA.2.12.1	BA.2.12.1	RI-RISHL-064906|EPI_ISL_13285448|USA|BA.2.12.1|2022-05-21	99,75
EPI_ISL_13285993	BA.2.10	BA.2.12	BA.2.12	TG-RFCH06822_CID1920|EPI_ISL_13285993|India|BA.2.10|2022-02-08	86,70
EPI_ISL_13286133	BA.2	BA.2	BA.2_1	TG-RFCH07000_CID2336|EPI_ISL_13286133|India|BA.2|2022-04-02	99,33
EPI_ISL_13289223	BA.2.12.1	BA.2.12.1	BA.2.12.1	ON-KHS-22-07581-v1|EPI_ISL_13289223|Canada|BA.2.12.1|2022-06-03	99,77
EPI_ISL_13289520	BA.2.12.1	BA.2.12.1	BA.2.12.1	NC-CORVASEQ-CLT-008617|EPI_ISL_13289520|USA|BA.2.12.1|2022-05-27	99,81
EPI_ISL_13289746	BA.2.12.1	BA.2.12.1	BA.2.12.1	CA-Stanford-83_S21|EPI_ISL_13289746|USA|BA.2.12.1|2022-05-26	99,81
EPI_ISL_13289861	BA.2.12.1	BA.2.12.1	BA.2.12.1	NY-CDC-QDX37459833|EPI_ISL_13289861|USA|BA.2.12.1|2022-05-26	99,81
EPI_ISL_13290213	BA.2	A	A_14	FL-CDC-QDX37458851|EPI_ISL_13290213|USA|BA.2|2022-05-27	99,81
EPI_ISL_13290936	BA.2.12.1	BA.2.12.1	BA.2.12.1	NY-CDC-QDX37458158|EPI_ISL_13290936|USA|BA.2.12.1|2022-05-29	99,81
EPI_ISL_13293775	BA.2.3	A	A_14	TKYkbm21674|EPI_ISL_13293775|Japan|BA.2.3|2022-05-06	99,81
EPI_ISL_13293994	BA.2	A	A_14	TKYkbm22388|EPI_ISL_13293994|Japan|BA.2|2022-05-09	99,81
EPI_ISL_13294451	BA.2.3.13	A	A_4	TKYkbm22781|EPI_ISL_13294451|Japan|BA.2.3.13|2022-05-06	99,79
EPI_ISL_13295531	BA.2.12.1	BA.2.12.1	BA.2.12.1	AK-PHL20692|EPI_ISL_13295531|USA|BA.2.12.1|2022-05-17	92,75
EPI_ISL_13297749	BA.2	A	A_14	SMC-7073771|EPI_ISL_13297749|Israel|BA.2|2022-06-02	99,81
EPI_ISL_13298740	BA.2.9	BA.2	BA.2_1	ZNA_25429027|EPI_ISL_13298740|Belgium|BA.2.9|2022-05-20	98,23
EPI_ISL_13298838	BA.2	BA.2	BA.2_1	D-NVRL-S22IRL00336484|EPI_ISL_13298838|Ireland|BA.2|2022-05-24	99,79
EPI_ISL_13303723	BA.2	BA.2	BA.2_1	QC-L00482962001|EPI_ISL_13303723|Canada|BA.2|2022-05-31	99,81
EPI_ISL_13303818	BA.2	A	A_14	QC-L00481296001|EPI_ISL_13303818|Canada|BA.2|2022-05-30	99,81
EPI_ISL_13303890	BA.1.1	BA.1.1	BA.1.1_2	rega-22779|EPI_ISL_13303890|Belgium|BA.1.1|2021-12-15	82,48
EPI_ISL_13305676	BA.2.12.1	BA.2.12.1	BA.2.12.1	FL-CDC-STM-U7BSTJWT7|EPI_ISL_13305676|USA|BA.2.12.1|2022-06-02	99,79
EPI_ISL_13307384	BA.2.9	A	A_17	CA-CDC-LC0688519|EPI_ISL_13307384|USA|BA.2.9|2022-05-24	99,81
EPI_ISL_13309041	BA.2.12.1	BA.2.12.1	BA.2.12.1	NC-CDC-LC0684378|EPI_ISL_13309041|USA|BA.2.12.1|2022-05-29	99,81
EPI_ISL_13309701	BA.2.12.1	BA.2.12.1	BA.2.12.1	NJ-CDC-LC0684950|EPI_ISL_13309701|USA|BA.2.12.1|2022-05-31	98,42
EPI_ISL_13310350	BA.2	BA.2	BA.2_1	CA-CDC-LC0686612|EPI_ISL_13310350|USA|BA.2|2022-05-31	99,81
EPI_ISL_13310856	BA.5.1	A	A_3	NC-CDC-LC0683128|EPI_ISL_13310856|USA|BA.5.1|2022-06-01	99,69
EPI_ISL_13313359	BA.2.9	BA.2.12.1	BA.2.12.1	SMC-7073212|EPI_ISL_13313359|Israel|BA.2.9|2022-05-30	86,32
EPI_ISL_13314028	BA.2.12.1	BA.2.12.1	BA.2.12.1	ARE-INS-17811|EPI_ISL_13314028|Peru|BA.2.12.1|2022-05-23	99,81
EPI_ISL_13314177	BA.2.9	BA.2	BA.2_1	LIM-INS-17961|EPI_ISL_13314177|Peru|BA.2.9|2022-05-23	99,81
EPI_ISL_13314594	BA.4	BA.2.12	BA.2.12	IL-Curative-454823|EPI_ISL_13314594|USA|BA.4|2022-05-25	88,17
EPI_ISL_13314781	BA.5.2.1	A	A_9	NY-CDC-LC0685330|EPI_ISL_13314781|USA|BA.5.2.1|2022-06-01	99,69
EPI_ISL_13315794	BA.2.12.1	BA.2.12.1	BA.2.12.1	RI-CDC-LC0682416|EPI_ISL_13315794|USA|BA.2.12.1|2022-06-04	99,81
EPI_ISL_13315840	BA.2.12.1	BA.2.12.1	BA.2.12.1	SC-CDC-LC0683367|EPI_ISL_13315840|USA|BA.2.12.1|2022-06-04	99,81
EPI_ISL_13318439	BA.2.12.1	BA.2.12.1	BA.2.12.1	BC-BCCDC-436525|EPI_ISL_13318439|Canada|BA.2.12.1|2022-05-06	99,81
EPI_ISL_13319440	BA.2	A	A_14	BC-BCCDC-440409|EPI_ISL_13319440|Canada|BA.2|2022-05-13	99,81
EPI_ISL_13319604	BA.2.12.1	BA.2.12.1	BA.2.12.1	BC-BCCDC-440502|EPI_ISL_13319604|Canada|BA.2.12.1|2022-05-15	99,81
EPI_ISL_13323584	BA.5.2.1	A	A_3	ICH-741238137|EPI_ISL_13323584|Israel|BA.5.2.1|2022-06-07	99,69
EPI_ISL_13323787	BA.5.2.1	A	A_3	PAC-ChTo-T222321258|EPI_ISL_13323787|France|BA.5.2.1|2022-06-07	99,69
EPI_ISL_13324846	BA.5.2	A	A_3	LSPA-3E4CC49|EPI_ISL_13324846|United Kingdom|BA.5.2|2022-06-08	99,69
EPI_ISL_13327776	BA.5.2	A	A_3	DCGC-529389|EPI_ISL_13327776|Denmark|BA.5.2|2022-06-09	99,69
EPI_ISL_13328283	BA.1.21	BA.1.15	BA.1.15	36143|EPI_ISL_13328283|Norway|BA.1.21|2021-12-20	99,64
EPI_ISL_13328729	BA.2	BA.2	BA.2_1	NOR-HMN-22052160844|EPI_ISL_13328729|France|BA.2|2022-05-09	99,81
EPI_ISL_13330715	BA.2.11	BA.2	BA.2_3	BRE-HMN-22052130655|EPI_ISL_13330715|France|BA.2.11|2022-05-05	99,81
EPI_ISL_13332386	BA.2	BA.2	BA.2_1	GE-RIVM-102177|EPI_ISL_13332386|Netherlands|BA.2|2022-05-18	99,81
EPI_ISL_13332924	BA.2.12.1	BA.2.12.1	BA.2.12.1	MS-MSPHL-1160|EPI_ISL_13332924|USA|BA.2.12.1|2022-06-01	99,81
EPI_ISL_13333268	BA.2	BA.2	BA.2_1	RP-USAFSAM-S16757|EPI_ISL_13333268|Germany|BA.2|2022-05-09	90,94
EPI_ISL_13333856	BA.2	BA.2	BA.2_1	BAS-ASM-251-2022|EPI_ISL_13333856|Italy|BA.2|2022-06-07	98,66
EPI_ISL_13337147	BA.2.42	BA.2	BA.2_2	HI-H2211773|EPI_ISL_13337147|USA|BA.2.42|2022-05-23	99,81
EPI_ISL_13338443	BA.2.12.1	BA.2.12.1	BA.2.12.1	LSPA-3E5481D|EPI_ISL_13338443|United Kingdom|BA.2.12.1|2022-06-10	99,81
EPI_ISL_13340790	BA.1.1	BA.1.1	BA.1.1_2	rega-24058|EPI_ISL_13340790|Belgium|BA.1.1|2021-12-30	99,64
EPI_ISL_13342650	BA.5.1	A	A_3	CVL-HCL722002954101|EPI_ISL_13342650|France|BA.5.1|2022-05-30	99,69
EPI_ISL_13345537	BA.2.12.1	BA.2.12.1	BA.2.12.1	NC-CORVASEQ-1086-3072|EPI_ISL_13345537|USA|BA.2.12.1|2022-06-10	95,21
EPI_ISL_13345970	BA.2	BA.2	BA.2_1	NL-PHML-447062|EPI_ISL_13345970|Canada|BA.2|2022-05-24	90,52
EPI_ISL_13346113	BA.2.3	A	A_14	NS-NSH-439771|EPI_ISL_13346113|Canada|BA.2.3|2022-04-20	90,06
EPI_ISL_13349101	BA.2	BA.2.10	BA.2.10	AB-ABPHL-68659|EPI_ISL_13349101|Canada|BA.2|2022-05-17	99,79
EPI_ISL_13349841	BA.2	BA.2.67	BA.2.67	ARA-HMN-22042110622|EPI_ISL_13349841|France|BA.2|2022-04-04	98,34
EPI_ISL_13353255	BA.2.12.1	BA.2.12.1	BA.2.12.1	KDCA71864|EPI_ISL_13353255|South Korea|BA.2.12.1|2022-05-25	99,81
EPI_ISL_13354659	BA.5.2.1	A	A_3	SMC-7075063|EPI_ISL_13354659|Israel|BA.5.2.1|2022-06-13	99,69
EPI_ISL_13355015	BA.4	A	A_9	SMC-7075644|EPI_ISL_13355015|Israel|BA.4|2022-06-11	99,69
EPI_ISL_13356814	BA.2.3	A	A_14	WA-UW-22052424684|EPI_ISL_13356814|USA|BA.2.3|2022-05-24	96,74
EPI_ISL_13356894	BA.2.3.17	A	A_14	WA-UW-22042227003|EPI_ISL_13356894|USA|BA.2.3.17|2022-04-22	99,52
EPI_ISL_13357409	BA.5.1	A	A_3	DCGC-530448|EPI_ISL_13357409|Denmark|BA.5.1|2022-06-12	99,69
EPI_ISL_13360346	BA.2	A	A_14	6753|EPI_ISL_13360346|Singapore|BA.2|2022-06-14	99,81
EPI_ISL_13360396	BA.5.1	A	A_3	6803|EPI_ISL_13360396|Singapore|BA.5.1|2022-06-15	99,69
EPI_ISL_13360781	BA.2.9	BA.2	BA.2_1	rega-42573|EPI_ISL_13360781|Belgium|BA.2.9|2022-05-23	99,81
EPI_ISL_13362835	BA.1.1	BA.2.12.1	BA.2.12.1	22V-00311|EPI_ISL_13362835|Cameroon|BA.1.1|2022-01-03	99,68
EPI_ISL_13362860	BA.1.14	BA.1	BA.1_3	BA-LACENBA-292436979|EPI_ISL_13362860|Brazil|BA.1.14|2022-02-08	99,64
EPI_ISL_13363791	BA.2	BA.2.10	BA.2.10	SC-NVBS17954GENOV045618771317|EPI_ISL_13363791|Brazil|BA.2|2022-04-14	97,79
EPI_ISL_13364463	BA.2.9	A	A_17	NY-PRL-220527_02J23|EPI_ISL_13364463|USA|BA.2.9|2022-05-26	99,39
EPI_ISL_13366593	BA.4	A	A_3	NY-PRL-220613_02E20|EPI_ISL_13366593|USA|BA.4|2022-06-10	99,69
EPI_ISL_13367659	BA.1	BA.1.15	BA.1.15	rega-25325|EPI_ISL_13367659|Belgium|BA.1|2021-12-19	99,64
EPI_ISL_13370422	BA.2	BA.2	BA.2_1	ARA-CFD220032477901|EPI_ISL_13370422|France|BA.2|2022-06-08	97,67
EPI_ISL_13370649	BA.2.54	BA.2.54	BA.2.54	MD-HRYC-12350050|EPI_ISL_13370649|Spain|BA.2.54|2022-06-07	99,81
EPI_ISL_13373056	BA.2.76	BA.2.76	BA.2.76	KA-RFNB-10052|EPI_ISL_13373056|India|BA.2.76|2022-06	99,81
EPI_ISL_13374756	BA.1	A	A_1	rega-26483|EPI_ISL_13374756|Belgium|BA.1|2022-01-08	82,45
EPI_ISL_13376461	BF.1	A	A_3	LSPA-3E5CC64|EPI_ISL_13376461|United Kingdom|BF.1|2022-06-15	99,68
EPI_ISL_13376951	BA.2	BA.2	BA.2_1	QEUH-3E56C88|EPI_ISL_13376951|United Kingdom|BA.2|2022-04-13	99,81

EPI_ISL_13378438	BA.2	A	A_14	BW-RKI-I-863521|EPI_ISL_13378438|Germany|BA.2|2022-06-01	99,81
EPI_ISL_13378851	BA.5.2	A	A_3	BW-RKI-I-863738|EPI_ISL_13378851|Germany|BA.5.2|2022-05-27	99,69
EPI_ISL_13379283	BA.5.1	A	A_9	SH-RKI-I-864192|EPI_ISL_13379283|Germany|BA.5.1|2022-06-08	89,98
EPI_ISL_13381278	BA.4.1	A	A_3	NW-RKI-I-867491|EPI_ISL_13381278|Germany|BA.4.1|2022-06-07	99,69
EPI_ISL_13381388	BA.2	BA.2	BA.2_1	BW-RKI-I-867606|EPI_ISL_13381388|Germany|BA.2|2022-06-09	99,81
EPI_ISL_13381427	BE.1.1	A	A_3	NI-RKI-I-867646|EPI_ISL_13381427|Germany|BE.1.1|2022-05-31	99,69
EPI_ISL_13382163	BE.1.1	A	A_3	NW-RKI-I-868906|EPI_ISL_13382163|Germany|BE.1.1|2022-06-10	99,69
EPI_ISL_13382176	BE.1.1	A	A_3	BY-RKI-I-868922|EPI_ISL_13382176|Germany|BE.1.1|2022-06-10	99,69
EPI_ISL_13382410	BA.2	A	A_17	NW-RKI-I-869185|EPI_ISL_13382410|Germany|BA.2|2022-06-03	99,81
EPI_ISL_13383338	BA.2	BA.2	BA.2_1	NW-RKI-I-870209|EPI_ISL_13383338|Germany|BA.2|2022-04-25	99,37
EPI_ISL_13384928	BE.1.1	A	A_9	SH-RKI-I-872004|EPI_ISL_13384928|Germany|BE.1.1|2022-06-13	94,12
EPI_ISL_13385020	BA.2.6	A	A_17	PDL-HMN-22052240448|EPI_ISL_13385020|France|BA.2.6|2022-05-16	99,54
EPI_ISL_13386148	BA.5	A	A_3	RP-RKI-I-873061|EPI_ISL_13386148|Germany|BA.5|2022-06-09	99,68
EPI_ISL_13388259	BA.4	A	A_9	PHEC-YYFXK7A|EPI_ISL_13388259|United Kingdom|BA.4|2022-06-06	99,68
EPI_ISL_13388673	BA.1	BA.1.15	BA.1.15	rega-26683|EPI_ISL_13388673|Belgium|BA.1|2022-01-13	99,64
EPI_ISL_13392998	BA.5.1.3	BA.5.1.3	BA.5.1.3	IB-HUSE-05700|EPI_ISL_13392998|Spain|BA.5.1.3|2022-06-06	99,69
EPI_ISL_13393172	BA.5.1	A	A_3	NW-HHU-28894|EPI_ISL_13393172|Germany|BA.5.1|2022-06-08	99,69
EPI_ISL_13396193	BA.2.12.1	BA.2.12.1	BA.2.12.1	UT-UPHL-220619146444|EPI_ISL_13396193|USA|BA.2.12.1|2022-06-11	99,81
EPI_ISL_13399994	BA.2	A	A_14	DCGC-531178|EPI_ISL_13399994|Denmark|BA.2|2022-06-13	95,75
EPI_ISL_13402790	BA.2	BA.2	BA.2_1	Jessa_55-2224-000056|EPI_ISL_13402790|Belgium|BA.2|2022-06-16	99,01
EPI_ISL_13406438	BA.2.12.1	BA.2.12.1	BA.2.12.1	SMC-7077674|EPI_ISL_13406438|Israel|BA.2.12.1|2022-06-13	95,71
EPI_ISL_13408431	BA.2.13	BA.2.13	BA.2.13	LIM-INS-18597|EPI_ISL_13408431|Peru|BA.2.13|2022-06-05	99,81
EPI_ISL_13410031	BA.4.1	BA.4.1	BA.4.1_1	LIM-INS-18936|EPI_ISL_13410031|Peru|BA.4.1|2022-06-07	99,69
EPI_ISL_13411514	BA.2.12.1	BA.2.12.1	BA.2.12.1	BC-BCCDC-448562|EPI_ISL_13411514|Canada|BA.2.12.1|2022-05-19	99,81
EPI_ISL_13411557	BA.2.12.1	BA.2.12.1	BA.2.12.1	BC-BCCDC-448616|EPI_ISL_13411557|Canada|BA.2.12.1|2022-05-23	99,81
EPI_ISL_13412402	BA.2.12.1	BA.2.12.1	BA.2.12.1	BC-BCCDC-450666|EPI_ISL_13412402|Canada|BA.2.12.1|2022-05-30	99,81
EPI_ISL_13412516	BA.2.13	BA.2.13	BA.2.13	BC-BCCDC-450771|EPI_ISL_13412516|Canada|BA.2.13|2022-05-30	99,81
EPI_ISL_13412577	BA.2.12.1	BA.2.12.1	BA.2.12.1	BC-BCCDC-450843|EPI_ISL_13412577|Canada|BA.2.12.1|2022-05-30	99,81
EPI_ISL_13412906	BA.5.2.2	A	A_3	BC-BCCDC-451316|EPI_ISL_13412906|Canada|BA.5.2.2|2022-06-04	99,69
EPI_ISL_13413033	BA.5.5	BA.5.5	BA.5.5_1	BC-BCCDC-451607|EPI_ISL_13413033|Canada|BA.5.5|2022-06-03	99,69
EPI_ISL_13413635	BA.2.12.1	BA.2.12.1	BA.2.12.1	TX-Curative-490632|EPI_ISL_13413635|USA|BA.2.12.1|2022-06-03	90,98
EPI_ISL_13416477	BA.2	BA.2	BA.2_1	CA-HLX-STM-TXGR4SDFC|EPI_ISL_13416477|USA|BA.2|2022-05-26	99,81
EPI_ISL_13416621	BA.2.12.1	BA.2.12	BA.2.12	CA-HLX-STM-9CBBH8AYQ|EPI_ISL_13416621|USA|BA.2.12.1|2022-05-30	88,40
EPI_ISL_13416888	BA.2.12.1	BA.2.12.1	BA.2.12.1	CA-HLX-STM-RFEGDJ4E7|EPI_ISL_13416888|USA|BA.2.12.1|2022-05-31	99,71
EPI_ISL_13416945	BA.2.9	A	A_17	CA-HLX-STM-MXJFWZG3J|EPI_ISL_13416945|USA|BA.2.9|2022-05-30	99,77
EPI_ISL_13419480	BA.5.2.1	A	A_3	LSPA-3E67343|EPI_ISL_13419480|United Kingdom|BA.5.2.1|2022-06-17	99,69
EPI_ISL_13419508	BA.2.23	BA.2	BA.2_1	LSPA-3E67000|EPI_ISL_13419508|United Kingdom|BA.2.23|2022-06-14	99,81
EPI_ISL_13420926	BA.5.1	A	A_3	DCGC-534216|EPI_ISL_13420926|Denmark|BA.5.1|2022-06-14	99,62
EPI_ISL_13421068	BA.2	A	A_14	QEUH-3E30BB4|EPI_ISL_13421068|United Kingdom|BA.2|2022-05-26	99,81
EPI_ISL_13421178	BA.2	BA.2	BA.2_1	PHEC-YYFORIM|EPI_ISL_13421178|United Kingdom|BA.2|2022-05-30	99,79
EPI_ISL_13423973	BA.2.12.1	BA.2.12.1	BA.2.12.1	FL-CDC-STM-4BYKZQVRM|EPI_ISL_13423973|USA|BA.2.12.1|2022-06-06	99,81
EPI_ISL_13426879	BA.5.2.1	A	A_3	NAQ-HCL022100208901|EPI_ISL_13426879|France|BA.5.2.1|2022-06-06	99,69
EPI_ISL_13427579	BA.5.1	A	A_9	Jessa_11-2225-001192|EPI_ISL_13427579|Belgium|BA.5.1|2022-06-20	98,89
EPI_ISL_13429384	BA.2	A	A_4	ICH-741228949|EPI_ISL_13429384|Israel|BA.2|2022-06-09	99,81
EPI_ISL_13432711	BA.5.5	BA.5.5	BA.5.5_1	MO-CDC-QDX37663578|EPI_ISL_13432711|USA|BA.5.5|2022-06-01	99,69
EPI_ISL_13432749	BA.2.12.1	BA.2.12.1	BA.2.12.1	NY-CDC-QDX37664434|EPI_ISL_13432749|USA|BA.2.12.1|2022-06-01	99,81
EPI_ISL_13435896	BA.2.12.1	BA.2.12.1	BA.2.12.1	CA-CDPH-6000011272|EPI_ISL_13435896|USA|BA.2.12.1|2022-05-17	90,52
EPI_ISL_13436664	BA.2	BA.2.10	BA.2.10	22CH5015|EPI_ISL_13436664|New Zealand|BA.2|2022-06-10	99,79
EPI_ISL_13437605	BA.5.2.1	A	A_3	22CH4500|EPI_ISL_13437605|New Zealand|BA.5.2.1|2022-05-26	99,68
EPI_ISL_13437915	BA.2	BA.2.10	BA.2.10	22CV5196|EPI_ISL_13437915|New Zealand|BA.2|2022-05-29	99,79
EPI_ISL_13439695	BA.5.1	A	A_3	NSW-ICPMR-27746|EPI_ISL_13439695|Australia|BA.5.1|2022-06-15	90,52
EPI_ISL_13440911	BA.2.3.8	BA.2	BA.2_1	KDCA76595|EPI_ISL_13440911|South Korea|BA.2.3.8|2022-05-31	99,81
EPI_ISL_13442834	BA.5.1.2	A	A_9	DCGC-534773|EPI_ISL_13442834|Denmark|BA.5.1.2|2022-06-19	99,66
EPI_ISL_13443264	BA.2	BA.2	BA.2_1	PHEC-YYFO6HD|EPI_ISL_13443264|United Kingdom|BA.2|2022-06-03	99,79
EPI_ISL_13443750	BA.2.44	BA.2.44	BA.2.44	DCGC-535358|EPI_ISL_13443750|Denmark|BA.2.44|2022-06-17	99,77
EPI_ISL_13443951	BA.2.18	BA.2.18	BA.2.18	PHEC-YYFOXRD|EPI_ISL_13443951|United Kingdom|BA.2.18|2022-05-30	99,79
EPI_ISL_13444079	BA.2	A	A_14	PHEP-YYFBEZN|EPI_ISL_13444079|United Kingdom|BA.2|2022-05-27	99,77
EPI_ISL_13446491	BA.2.12.1	BA.2.12.1	BA.2.12.1	WX-NVRL-S22IRL00343806|EPI_ISL_13446491|Ireland|BA.2.12.1|2022-06-01	99,79
EPI_ISL_13452546	BA.5.1	A	A_3	CO-CDC-STM-BGDVT9BF8|EPI_ISL_13452546|USA|BA.5.1|2022-06-13	99,69
EPI_ISL_13454943	BA.2.3	A	A_14	AB-ABPHL-69241|EPI_ISL_13454943|Canada|BA.2.3|2022-05-24	99,81
EPI_ISL_13457976	BA.2.12.2	BA.2.12.2	BA.2.12.2	VIC62844|EPI_ISL_13457976|Australia|BA.2.12.2|2022-05-24	99,81
EPI_ISL_13458916	BA.4	BA.4.4	BA.4.4	VIC63836|EPI_ISL_13458916|Australia|BA.4|2022-06-06	99,69
EPI_ISL_13459775	BA.2.12.1	BA.2.12.1	BA.2.12.1	NC-CORVASEQ-1086-3130|EPI_ISL_13459775|USA|BA.2.12.1|2022-06-13	99,81
EPI_ISL_13459884	BA.2	A	A_17	SC-FIOCRUZ-13640|EPI_ISL_13459884|Brazil|BA.2|2022-05-30	99,81
EPI_ISL_13460981	BA.2.12.1	BA.2.12.1	BA.2.12.1	ICH-741233014|EPI_ISL_13460981|Israel|BA.2.12.1|2022-06-21	99,81
EPI_ISL_13463236	BA.2.3	A	A_14	KY-GD_02709|EPI_ISL_13463236|USA|BA.2.3|2022-04-25	99,81
EPI_ISL_13463887	BA.2.38	BA.2.38	BA.2.38	UP-MRU-ICMR-Insacog-97|EPI_ISL_13463887|India|BA.2.38|2022-06-19	71,57
EPI_ISL_13465790	BA.2.18	BA.2.18	BA.2.18	DCGC-535842|EPI_ISL_13465790|Denmark|BA.2.18|2022-06-20	99,77
EPI_ISL_13467242	BA.2.3.10	A	A_14	QC-L00486139001|EPI_ISL_13467242|Canada|BA.2.3.10|2022-06-06	99,81
EPI_ISL_13467328	BA.4.1	A	A_3	QC-L00485031001|EPI_ISL_13467328|Canada|BA.4.1|2022-06-07	99,69
EPI_ISL_13467656	BA.2	BA.2	BA.2_1	QC-L00484858001|EPI_ISL_13467656|Canada|BA.2|2022-06-06	99,81
EPI_ISL_13468034	BA.5.2.1	A	A_3	ICH-741233641|EPI_ISL_13468034|Israel|BA.5.2.1|2022-06-22	99,69
EPI_ISL_13470727	BA.5.2.1	A	A_3	LSPA-3E71513|EPI_ISL_13470727|United Kingdom|BA.5.2.1|2022-06-19	99,69
EPI_ISL_13475203	BA.1.1	BA.1.1	BA.1.1_2	rega-28951|EPI_ISL_13475203|Belgium|BA.1.1|2022-01-30	82,48
EPI_ISL_13475850	BA.4	BA.5.1.5	BA.5.1.5	PHEP-YYFBIF4|EPI_ISL_13475850|United Kingdom|BA.4|2022-06-11	99,60
EPI_ISL_13476183	BE.1.1	A	A_9	PHEP-YYFNFMH|EPI_ISL_13476183|United Kingdom|BE.1.1|2022-06-12	99,66
EPI_ISL_13476725	B.1.1.529	BA.2	BA.2_1	rega-29325|EPI_ISL_13476725|Belgium|B.1.1.529|2022-02-02	99,71
EPI_ISL_13477808	BA.2	BA.2	BA.2_1	37589|EPI_ISL_13477808|Croatia|BA.2|2022-05-12	98,51
EPI_ISL_13478370	BA.2.56	BA.2.56	BA.2.56	12210417801|EPI_ISL_13478370|Belgium|BA.2.56|2022-06-14	96,36
EPI_ISL_13480249	BA.2.12.1	BA.2.12.1	BA.2.12.1	NY-PRL-220621_01F15|EPI_ISL_13480249|USA|BA.2.12.1|2022-06-17	99,81
EPI_ISL_13481422	BA.4	BA.4.4	BA.4.4	TX-HMH-MCoV-101078|EPI_ISL_13481422|USA|BA.4|2022-06-14	99,68
EPI_ISL_13481804	BA.1	BA.1.15	BA.1.15	rega-29519|EPI_ISL_13481804|Belgium|BA.1|2022-01-21	99,64
EPI_ISL_13482920	BA.2	A	A_3	TX-HMH-MCoV-101772|EPI_ISL_13482920|USA|BA.2|2022-06-18	99,66
EPI_ISL_13485721	BA.2.12.1	BA.2.12.1	BA.2.12.1	FL-CDC-STM-G44Y5PF84|EPI_ISL_13485721|USA|BA.2.12.1|2022-06-10	99,81
EPI_ISL_13486762	BA.5.6	A	A_3	LA-CDC-STM-85JUNC64C|EPI_ISL_13486762|USA|BA.5.6|2022-06-14	99,69
EPI_ISL_13486769	BA.2.12.1	BA.2.12.1	BA.2.12.1	TX-CDC-STM-97SEJJ9F6|EPI_ISL_13486769|USA|BA.2.12.1|2022-06-14	99,81
EPI_ISL_13487049	BA.1.1	BA.1.1	BA.1.1_3	ON-KHS-22-00357-v1|EPI_ISL_13487049|Canada|BA.1.1|2022-01-19	89,05
EPI_ISL_13488365	BA.1.1	BA.1.1	BA.1.1_2	ON-KHS-22-01868-v1|EPI_ISL_13488365|Canada|BA.1.1|2022-02-16	99,39
EPI_ISL_13488436	BA.1.1.14	BA.1	BA.1_4	ON-KHS-22-01962-v1|EPI_ISL_13488436|Canada|BA.1.1.14|2022-02-18	97,48
EPI_ISL_13489074	BA.1.1	BA.1.1	BA.1.1_2	ON-KHS-22-02695-v1|EPI_ISL_13489074|Canada|BA.1.1|2022-03-03	98,87
EPI_ISL_13489543	BA.1.1	BA.1.1	BA.1.1_2	ON-KHS-22-03239-v1|EPI_ISL_13489543|Canada|BA.1.1|2022-03-12	99,62
EPI_ISL_13490796	BA.2.9	BA.2	BA.2_1	CeMM24620|EPI_ISL_13490796|Austria|BA.2.9|2022-02-14	96,39
EPI_ISL_13490840	BA.1.1	BA.1.1	BA.1.1_3	CeMM25094|EPI_ISL_13490840|Austria|BA.1.1|2022-02-22	96,51
EPI_ISL_13493499	BA.2.76	BA.2.76	BA.2.76	KA-RFNB-10988|EPI_ISL_13493499|India|BA.2.76|2022-06	99,81
EPI_ISL_13497672	BA.1.1	BA.1.1	BA.1.1_2	AZ-ASU83460|EPI_ISL_13497672|USA|BA.1.1|2022-01-06	99,64
EPI_ISL_13497860	BA.2.12.1	BA.2.12.1	BA.2.12.1	NV-SNPHL-613203|EPI_ISL_13497860|USA|BA.2.12.1|2022-05-18	85,12
EPI_ISL_13498545	BE.1	BA.5.3.2	BA.5.3.2	LSPA-3E74176|EPI_ISL_13498545|United Kingdom|BE.1|2022-06-19	99,69
EPI_ISL_13500310	BA.2	BA.2	BA.2_1	7066|EPI_ISL_13500310|Singapore|BA.2|2022-06-19	99,81
EPI_ISL_13501028	BA.4.1.1	A	A_3	DCGC-537120|EPI_ISL_13501028|Denmark|BA.4.1.1|2022-06-20	99,69
EPI_ISL_13503659	BA.5.2.1	A	A_3	ON-KHS-22-08328-v1|EPI_ISL_13503659|Canada|BA.5.2.1|2022-06-20	99,69
EPI_ISL_13505003	BA.1.1	BA.1.1	BA.1.1_3	rega-30600|EPI_ISL_13505003|Belgium|BA.1.1|2022-02-15	96,89

EPI_ISL_13505046	BA.2.40.1	BA.2.38	BA.2.38	UNIMAS-HSIBUML258|EPI_ISL_13505046|Malaysia|BA.2.40.1|2022-05-21	99,79
EPI_ISL_13505150	BA.2.12.1	BA.2.12.1	BA.2.12.1	NV-NSPHL-22-00059119|EPI_ISL_13505150|USA|BA.2.12.1|2022-06-14	90,52
EPI_ISL_13506817	BA.2.12.1	BA.2.12.1	BA.2.12.1	CA-HLX-STM-R8SRJD27Y|EPI_ISL_13506817|USA|BA.2.12.1|2022-06-06	99,79
EPI_ISL_13507101	BA.2	BA.2	BA.2_1	CA-HLX-STM-BTJTEPU4C|EPI_ISL_13507101|USA|BA.2|2022-05-25	99,81
EPI_ISL_13509061	BA.2.9	BA.2	BA.2_1	INC-4876-758437|EPI_ISL_13509061|Costa Rica|BA.2.9|2022-06-03	99,81
EPI_ISL_13510282	BA.2.12.1	BA.2.12.1	BA.2.12.1	IL-CDC-QDX37911838|EPI_ISL_13510282|USA|BA.2.12.1|2022-06-08	99,81
EPI_ISL_13510627	BA.2.12.1	BA.2.12.1	BA.2.12.1	OH-CDC-QDX37912174|EPI_ISL_13510627|USA|BA.2.12.1|2022-06-09	99,81
EPI_ISL_13510667	BA.2.12.1	BA.2.12.1	BA.2.12.1	IL-CDC-QDX37912082|EPI_ISL_13510667|USA|BA.2.12.1|2022-06-09	99,81
EPI_ISL_13511961	BA.2	BA.2	BA.2_1	CA-CDPH-2000072507|EPI_ISL_13511961|USA|BA.2|2022-05-02	97,21
EPI_ISL_13512058	BA.2.12.1	BA.2.12.1	BA.2.12.1	CA-CDPH-FS25454739|EPI_ISL_13512058|USA|BA.2.12.1|2022-05-12	94,54
EPI_ISL_13512265	BA.2.40.1	BA.2.38	BA.2.38	7153|EPI_ISL_13512265|Singapore|BA.2.40.1|2022-06-19	99,79
EPI_ISL_13513769	BA.2.3.1	BA.2.3.1	BA.2.3.1	TKYkbm25581|EPI_ISL_13513769|Japan|BA.2.3.1|2022-06-08	99,79
EPI_ISL_13514931	BA.5.2	A	A_3	OCC-GENBIO-M89GN1710104|EPI_ISL_13514931|France|BA.5.2|2022-06-20	99,69
EPI_ISL_13515269	BA.5.1	A	A_3	CVL-LBZCentre-A300561226|EPI_ISL_13515269|France|BA.5.1|2022-06-20	99,71
EPI_ISL_13515954	BA.5	A	A_3	LSPA-3E77726|EPI_ISL_13515954|United Kingdom|BA.5|2022-06-21	94,20
EPI_ISL_13516877	BA.2	BA.2	BA.2_1	QEUH-3E74BE1|EPI_ISL_13516877|United Kingdom|BA.2|2022-06-20	94,09
EPI_ISL_13516952	BA.5.1	A	A_3	CVL-ChuTo-9800088986|EPI_ISL_13516952|France|BA.5.1|2022-06-10	99,69
EPI_ISL_13519334	BA.5.6	A	A_3	DCGC-538097|EPI_ISL_13519334|Denmark|BA.5.6|2022-06-23	99,69
EPI_ISL_13520883	BA.2.12.1	BA.2.12.1	BA.2.12.1	MH-NVRL-ecS22IRL00321310|EPI_ISL_13520883|Ireland|BA.2.12.1|2022-05-08	96,28
EPI_ISL_13521806	BA.5.2.2	A	A_3	GES-HMN-22062090941|EPI_ISL_13521806|France|BA.5.2.2|2022-06-07	99,69
EPI_ISL_13522083	BA.2.12.1	BA.2.12.1	BA.2.12.1	FL-BPHL-9808|EPI_ISL_13522083|USA|BA.2.12.1|2022-05-31	99,81
EPI_ISL_13522551	BA.2.45	BA.1	BA.1_4	48514454X5|EPI_ISL_13522551|Sweden|BA.2.45|2022-05-25	95,02
EPI_ISL_13523450	BA.4	A	A_9	22-123206|EPI_ISL_13523450|Sweden|BA.4|2022-05-24	96,22
EPI_ISL_13525098	BA.5.2	A	A_3	HDF-IPP40102|EPI_ISL_13525098|France|BA.5.2|2022-06-13	99,69
EPI_ISL_13525359	BA.4.1	A	A_3	CA-CDC-LC0710178|EPI_ISL_13525359|USA|BA.4.1|2022-06-07	99,69
EPI_ISL_13533318	BA.2.12.1	BA.2.12.1	BA.2.12.1	CO-CDPHE-2103286772|EPI_ISL_13533318|USA|BA.2.12.1|2022-05-24	80,88
EPI_ISL_13533432	Unassigned	BA.2.12.1	BA.2.12.1	CO-CDPHE-2103270796|EPI_ISL_13533432|USA|Unassigned|2022-05-18	48,88
EPI_ISL_13533770	BA.4.1	A	A_3	CO-CDPHE-2103354854|EPI_ISL_13533770|USA|BA.4.1|2022-06-09	99,69
EPI_ISL_13534482	BA.5.1	BA.5.1.5	BA.5.1.5	DCGC-538797|EPI_ISL_13534482|Denmark|BA.5.1|2022-06-24	94,92
EPI_ISL_13535119	BA.4.1	A	A_3	DCGC-539443|EPI_ISL_13535119|Denmark|BA.4.1|2022-06-25	99,69
EPI_ISL_13536587	BA.2.38	BA.2.12	BA.2.12	WB-INSACOG-1931503186602|EPI_ISL_13536587|India|BA.2.38|2022-06-06	90,14
EPI_ISL_13537072	BA.2.76	BA.2.76	BA.2.76	WB-INSACOG-1931503195501|EPI_ISL_13537072|India|BA.2.76|2022-06-16	99,79
EPI_ISL_13538464	BA.2	BA.2	BA.2_1	QEUH-3E6A656|EPI_ISL_13538464|United Kingdom|BA.2|2022-06-15	97,63
EPI_ISL_13542110	BA.5.5	BA.5.5	BA.5.5_1	NM-CDC-LC0716892|EPI_ISL_13542110|USA|BA.5.5|2022-06-13	99,69
EPI_ISL_13542132	BA.4.1	A	A_3	IL-CDC-LC0717576|EPI_ISL_13542132|USA|BA.4.1|2022-06-13	99,69
EPI_ISL_13542712	BA.2.12.1	BA.2.12.1	BA.2.12.1	NIC-INSPI-93903|EPI_ISL_13542712|Ecuador|BA.2.12.1|2022-06-07	99,81
EPI_ISL_13542903	BA.5.6	A	A_3	NY-CDC-LC0712611|EPI_ISL_13542903|USA|BA.5.6|2022-06-14	98,87
EPI_ISL_13543175	BA.2.9	A	A_17	NC-CDC-LC0714511|EPI_ISL_13543175|USA|BA.2.9|2022-06-14	99,81
EPI_ISL_13544601	BA.5.5	BA.5.5	BA.5.5_1	CA-CDC-LC0691321|EPI_ISL_13544601|USA|BA.5.5|2022-05-26	99,69
EPI_ISL_13545265	BA.2.12.1	BA.2.12.1	BA.2.12.1	CA-CDC-LC0691235|EPI_ISL_13545265|USA|BA.2.12.1|2022-05-28	99,81
EPI_ISL_13548210	BA.5.2.1	A	A_3	WA-CDC-LC0696530|EPI_ISL_13548210|USA|BA.5.2.1|2022-06-04	99,69
EPI_ISL_13549409	BA.2	A	A_14	RI-CDC-LC0694437|EPI_ISL_13549409|USA|BA.2|2022-06-07	99,81
EPI_ISL_13549659	BA.2.12.1	BA.2.12.1	BA.2.12.1	NC-CDC-LC0695047|EPI_ISL_13549659|USA|BA.2.12.1|2022-06-08	99,81
EPI_ISL_13550075	BA.2.12.1	BA.2.12.1	BA.2.12.1	OH-CDC-QDX37597055|EPI_ISL_13550075|USA|BA.2.12.1|2022-05-31	99,81
EPI_ISL_13550242	BA.2	BA.2	BA.2_1	MI-CDC-QDX37597133|EPI_ISL_13550242|USA|BA.2|2022-05-31	99,81
EPI_ISL_13552005	BA.2.12.1	BA.2.12.1	BA.2.12.1	FL-CDC-LC0702531|EPI_ISL_13552005|USA|BA.2.12.1|2022-06-06	99,81
EPI_ISL_13552989	BA.2.9	A	A_17	NC-CDC-LC0698137|EPI_ISL_13552989|USA|BA.2.9|2022-06-07	99,81
EPI_ISL_13553482	BA.5.2	A	A_3	NJ-CDC-LC0701248|EPI_ISL_13553482|USA|BA.5.2|2022-06-08	99,69
EPI_ISL_13553827	BA.2.9	BA.2	BA.2_1	NJ-CDC-LC0698655|EPI_ISL_13553827|USA|BA.2.9|2022-06-08	99,81
EPI_ISL_13555771	BA.4	A	A_9	NC-CDC-LC0700105|EPI_ISL_13555771|USA|BA.4|2022-06-10	99,69
EPI_ISL_13556612	BA.2.12.1	BA.2.12.1	BA.2.12.1	VA-CDC-QDX37595328|EPI_ISL_13556612|USA|BA.2.12.1|2022-05-31	99,81
EPI_ISL_13556960	BA.2.12.1	BA.2.12.1	BA.2.12.1	NJ-CDC-QDX37595684|EPI_ISL_13556960|USA|BA.2.12.1|2022-06-01	99,81
EPI_ISL_13558456	BA.2.12.1	BA.2.12.1	BA.2.12.1	WA-CDC-LC0706793|EPI_ISL_13558456|USA|BA.2.12.1|2022-06-08	99,81
EPI_ISL_13559442	BA.2.12.1	BA.2.12.1	BA.2.12.1	WA-CDC-UW22061382894|EPI_ISL_13559442|USA|BA.2.12.1|2022-06-13	99,81
EPI_ISL_13565685	BA.2.12.1	BA.2.12.1	BA.2.12.1	IC-11761|EPI_ISL_13565685|Japan|BA.2.12.1|2022-05-30	99,81
EPI_ISL_13566832	BA.5.2.1	A	A_3	LSPA-3E7F050|EPI_ISL_13566832|United Kingdom|BA.5.2.1|2022-06-22	99,69
EPI_ISL_13568083	BA.2	A	A_9	SH-RKI-I-876090|EPI_ISL_13568083|Germany|BA.2|2022-06-23	94,12
EPI_ISL_13568437	BA.5.1	A	A_9	SH-RKI-I-876647|EPI_ISL_13568437|Germany|BA.5.1|2022-06-25	94,09
EPI_ISL_13569523	BA.4.1.1	A	A_3	NI-RKI-I-878240|EPI_ISL_13569523|Germany|BA.4.1.1|2022-06-23	99,69
EPI_ISL_13573194	BA.5.1	A	A_3	DCGC-539928|EPI_ISL_13573194|Denmark|BA.5.1|2022-06-26	97,39
EPI_ISL_13573568	BA.5.1	A	A_3	DCGC-540302|EPI_ISL_13573568|Denmark|BA.5.1|2022-06-25	99,69
EPI_ISL_13574095	BA.4.1	A	A_3	PE-FIOCRUZ-IAM10053|EPI_ISL_13574095|Brazil|BA.4.1|2022-05-27	95,40
EPI_ISL_13574724	BA.2.38	B.1.1.519	B.1.1.519	MP-AIIMS_B-ICMR-INSACOG-WGS-936|EPI_ISL_13574724|India|BA.2.38|2022	77,08
EPI_ISL_13574854	BA.5.1	A	A_3	D-NVRL-S22IRL00356857|EPI_ISL_13574854|Ireland|BA.5.1|2022-06-14	99,68
EPI_ISL_13575500	BA.5	A	A_3	YUC_IBT_IMSS_7951|EPI_ISL_13575500|Mexico|BA.5|2022-06-13	99,60
EPI_ISL_13575849	BA.2.3	A	A_14	RIMD09816|EPI_ISL_13575849|Japan|BA.2.3|2022-04-06	99,81
EPI_ISL_13575962	BA.4	A	A_3	JK-GSILab-1077350|EPI_ISL_13575962|Indonesia|BA.4|2022-06-12	99,69
EPI_ISL_13576273	BA.2.3	A	A_14	RIMD09554|EPI_ISL_13576273|Japan|BA.2.3|2022-03-28	99,81
EPI_ISL_13576414	BA.2.3.13	BA.2	BA.2_1	RIMD09597|EPI_ISL_13576414|Japan|BA.2.3.13|2022-03-25	99,98
EPI_ISL_13576641	BA.4	A	A_9	SP-IB_160008|EPI_ISL_13576641|Brazil|BA.4|2022-06-15	99,69
EPI_ISL_13576964	BA.5.1	A	A_3	NH-AUMC-022926|EPI_ISL_13576964|Netherlands|BA.5.1|2022-06-13	99,69
EPI_ISL_13577514	BA.2	BA.2	BA.2_1	RIMD09967|EPI_ISL_13577514|Japan|BA.2|2022-03-28	99,81
EPI_ISL_13577601	BA.2.38	P.1	P.1_2	RJ-SMS-ICMR-INSACOG-TS-13980|EPI_ISL_13577601|India|BA.2.38|2022-05-04	95,82
EPI_ISL_13577850	BA.5.3.3	A	A_3	NH-AUMC-022979|EPI_ISL_13577850|Netherlands|BA.5.3.3|2022-06-19	99,69
EPI_ISL_13580863	BA.4	A	A_3	KY-Enfer-250622008_D8|EPI_ISL_13580863|Ireland|BA.4|2022-06-25	99,69
EPI_ISL_13581792	BE.1	A	A_3	TX-CDC-STM-ADS3655UR|EPI_ISL_13581792|USA|BE.1|2022-06-18	99,69
EPI_ISL_13581854	BA.2.12.1	BA.2.12.1	BA.2.12.1	AL-CDC-STM-H9HVZA879|EPI_ISL_13581854|USA|BA.2.12.1|2022-06-19	99,81
EPI_ISL_13581919	BA.5	A	A_3	AL-CDC-STM-BM4W5XXKF|EPI_ISL_13581919|USA|BA.5|2022-06-19	99,69
EPI_ISL_13582952	BA.5.1.1	A	A_3	IL-CDC-LC0721042|EPI_ISL_13582952|USA|BA.5.1.1|2022-06-13	99,69
EPI_ISL_13583863	BA.5.2.1	A	A_3	CA-CDC-LC0721400|EPI_ISL_13583863|USA|BA.5.2.1|2022-06-15	99,69
EPI_ISL_13584433	BA.5.1	A	A_3	CA-CDC-LC0722601|EPI_ISL_13584433|USA|BA.5.1|2022-06-16	99,69
EPI_ISL_13586869	BA.2.12.1	BA.2.12.1	BA.2.12.1	CA-HLX-STM-QXSD4MPWQ|EPI_ISL_13586869|USA|BA.2.12.1|2022-06-09	99,81
EPI_ISL_13588996	BA.2.56	BA.2.56	BA.2.56	RJ-LACENRJ-330962925|EPI_ISL_13588996|Brazil|BA.2.56|2022	98,07
EPI_ISL_13589503	BA.2.12.1	BA.2.12.1	BA.2.12.1	WI-MHDL-2022060517|EPI_ISL_13589503|USA|BA.2.12.1|2022-05-05	90,52
EPI_ISL_13591252	BA.2.12.1	BA.2.12.1	BA.2.12.1	TX-CDC-QDX37978352|EPI_ISL_13591252|USA|BA.2.12.1|2022-06-10	99,81
EPI_ISL_13592042	BA.2.12.1	BA.2.12.1	BA.2.12.1	NY-CDC-QDX38006936|EPI_ISL_13592042|USA|BA.2.12.1|2022-06-12	99,81
EPI_ISL_13592564	BA.2.12.1	BA.2.12.1	BA.2.12.1	CA-CDC-QDX38045905|EPI_ISL_13592564|USA|BA.2.12.1|2022-06-14	99,81
EPI_ISL_13597817	BA.1.1	BA.1.1	BA.1.1_2	MN-Mayo-13825|EPI_ISL_13597817|USA|BA.1.1|2021-12-28	99,64
EPI_ISL_13605524	BA.2.31	BA.2	BA.2_1	ICH-741234412|EPI_ISL_13605524|Israel|BA.2.31|2022-06-21	95,19
EPI_ISL_13606896	BA.2.12.1	BA.2.12.1	BA.2.12.1	IA-SHL-2121207|EPI_ISL_13606896|USA|BA.2.12.1|2022-06-20	90,52
EPI_ISL_13607130	BA.2.12.1	BA.2.12.1	BA.2.12.1	CO-CDPHE-2103341385|EPI_ISL_13607130|USA|BA.2.12.1|2022-06-07	99,81
EPI_ISL_13607489	BA.2	BA.2	BA.2_2	CO-CDPHE-2103340911|EPI_ISL_13607489|USA|BA.2|2022-06-05	94,07
EPI_ISL_13607660	BA.2.12.1	BA.2.12.1	BA.2.12.1	CO-CDPHE-2103358810|EPI_ISL_13607660|USA|BA.2.12.1|2022-06-13	80,88
EPI_ISL_13609222	BA.5.1	A	A_3	GRO-INMEGEN-77-5|EPI_ISL_13609222|Mexico|BA.5.1|2022-06-15	96,87
EPI_ISL_13609424	BA.2.12.1	BA.2.12.1	BA.2.12.1	CMX-INMEGEN-77-216|EPI_ISL_13609424|Mexico|BA.2.12.1|2022-06-23	98,93
EPI_ISL_13609427	BA.5.1	A	A_3	CMX-INMEGEN-77-219|EPI_ISL_13609427|Mexico|BA.5.1|2022-06-23	99,69
EPI_ISL_13611697	BA.5.1	A	A_3	KE-NVRL-S22IRL00356104|EPI_ISL_13611697|Ireland|BA.5.1|2022-06-13	99,68
EPI_ISL_13611892	BA.2.3.1	BA.2.3.1	BA.2.3.1	TKYnat1462|EPI_ISL_13611892|Japan|BA.2.3.1|2022-05-20	99,81
EPI_ISL_13612932	BA.2	BA.4.1	BA.4.1_1	WA4725|EPI_ISL_13612932|Australia|BA.2|2022-06-17	88,02
EPI_ISL_13613073	BA.2.64	BA.2.61	BA.2.61	WA4866|EPI_ISL_13613073|Australia|BA.2.64|2022-06-23	92,83
EPI_ISL_13614185	BA.5.1	DE.1	DE.1	ARA-GENBIO-14291801655|EPI_ISL_13614185|France|BA.5.1|2022-06-29	99,69

EPI_ISL_13615257	BA.2	A	A_17	SIC-CQRC-3422036815|EPI_ISL_13615257|Italy|BA.2|2022-06-10	99,81
EPI_ISL_13616088	BA.4	A	A_3	PHEC-YYFWFJ5|EPI_ISL_13616088|United Kingdom|BA.4|2022-06-20	99,68
EPI_ISL_13616304	BA.2.3	BA.2	BA.2_1	PHEC-YYFWUPF|EPI_ISL_13616304|United Kingdom|BA.2.3|2022-06-21	99,81
EPI_ISL_13616521	BF.1	A	A_3	PHEP-YYFDJOM|EPI_ISL_13616521|United Kingdom|BF.1|2022-06-17	93,91
EPI_ISL_13622410	BA.4.1	A	A_3	NY-PRL-220624_01L02|EPI_ISL_13622410|USA|BA.4.1|2022-06-21	99,69
EPI_ISL_13622522	BA.4	BA.4.4	BA.4.4	NY-PRL-220624_02E06|EPI_ISL_13622522|USA|BA.4|2022-06-21	99,69
EPI_ISL_13622539	BA.2.12.1	BA.2.12.1	BA.2.12.1	NY-PRL-220624_02F03|EPI_ISL_13622539|USA|BA.2.12.1|2022-06-21	99,81
EPI_ISL_13623357	BA.5.5	BA.5.5	BA.5.5_1	ON-PHL-22-25617|EPI_ISL_13623357|Canada|BA.5.5|2022-06-20	99,69
EPI_ISL_13623420	BA.2.12.1	BA.2.12.1	BA.2.12.1	ON-PHL-22-25683|EPI_ISL_13623420|Canada|BA.2.12.1|2022-06-20	99,81
EPI_ISL_13623857	BA.5.1	A	A_3	ON-PHL-22-26136|EPI_ISL_13623857|Canada|BA.5.1|2022-06-18	99,69
EPI_ISL_13623917	BA.2.12.1	BA.2.12.1	BA.2.12.1	ON-PHL-22-26198|EPI_ISL_13623917|Canada|BA.2.12.1|2022-06-23	99,81
EPI_ISL_13625084	BA.5.1.1	A	A_3	TX-HMH-MCoV-102680|EPI_ISL_13625084|USA|BA.5.1.1|2022-06-23	99,69
EPI_ISL_13625139	BA.2.12.1	BA.2.12.1	BA.2.12.1	TX-HMH-MCoV-102575|EPI_ISL_13625139|USA|BA.2.12.1|2022-06-23	99,79
EPI_ISL_13625937	BA.2.9	BA.2	BA.2_1	01_SE100_22CS104416|EPI_ISL_13625937|Sweden|BA.2.9|2022-06-16	99,79
EPI_ISL_13626286	BA.2	BA.2	BA.2_1	7630|EPI_ISL_13626286|Singapore|BA.2|2022-06-28	99,81
EPI_ISL_13627208	BA.2	BA.2	BA.2_1	742505|EPI_ISL_13627208|Latvia|BA.2|2022-04-08	91,11
EPI_ISL_13627347	BA.1.17	BA.1	BA.1_4	22068390NO|EPI_ISL_13627347|Latvia|BA.1.17|2022-02-04	99,64
EPI_ISL_13628457	BA.2	BA.2	BA.2_1	22082684NO|EPI_ISL_13628457|Latvia|BA.2|2022-02-11	98,51
EPI_ISL_13629099	BA.2	BA.2	BA.2_1	22101217NO|EPI_ISL_13629099|Latvia|BA.2|2022-02-21	98,47
EPI_ISL_13629986	BA.1	BA.1	BA.1_4	2201078585|EPI_ISL_13629986|Latvia|BA.1|2022-01-21	99,64
EPI_ISL_13633178	BA.1.13	BA.1.15	BA.1.15	03_SE600_000200294990N2|EPI_ISL_13633178|Sweden|BA.1.13|2022-01-06	85,98
EPI_ISL_13634451	BA.5.2.1	A	A_3	LSPA-3E95BEA|EPI_ISL_13634451|United Kingdom|BA.5.2.1|2022-06-28	99,69
EPI_ISL_13640005	BA.5.1	A	A_9	HSGM-GS5083|EPI_ISL_13640005|Turkey|BA.5.1|2022-06-16	98,55
EPI_ISL_13640973	BA.5.2	A	A_3	NV-CDC-LC0725478|EPI_ISL_13640973|USA|BA.5.2|2022-06-14	99,69
EPI_ISL_13641642	BA.2.12.1	BA.2.12.1	BA.2.12.1	CA-CDC-QDX38046165|EPI_ISL_13641642|USA|BA.2.12.1|2022-06-11	99,81
EPI_ISL_13646692	BA.2.12.1	BA.2.12.1	BA.2.12.1	CA-CDC-LC0729157|EPI_ISL_13646692|USA|BA.2.12.1|2022-06-23	99,81
EPI_ISL_13647453	BA.2.12.1	BA.2.12.1	BA.2.12.1	OH-CDC-QDX38155956|EPI_ISL_13647453|USA|BA.2.12.1|2022-06-18	99,81
EPI_ISL_13648979	BA.2.3	A	A_14	HH-hpi-p13092|EPI_ISL_13648979|Germany|BA.2.3|2022-02	99,35
EPI_ISL_13652621	BA.2	BA.2	BA.2_1	TKYnat1977|EPI_ISL_13652621|Japan|BA.2|2022-06-02	99,81
EPI_ISL_13653125	BA.2	BA.2	BA.2_1	RN-LACENRN-240807503|EPI_ISL_13653125|Brazil|BA.2|2022-05-25	99,81
EPI_ISL_13653632	BA.4.1	BA.4.1	BA.4.1_1	RJ-FIOCRUZ-14796|EPI_ISL_13653632|Brazil|BA.4.1|2022-06-15	99,69
EPI_ISL_13654237	BA.2.12.1	BA.2.12.1	BA.2.12.1	NJ-CDC-2-6048466|EPI_ISL_13654237|USA|BA.2.12.1|2022-05-17	99,81
EPI_ISL_13655805	BA.5.1	A	A_3	LSPA-3EA2B71|EPI_ISL_13655805|United Kingdom|BA.5.1|2022-06-29	99,69
EPI_ISL_13656114	BA.5.1.3	BA.5.1.3	BA.5.1.3	LSPA-3EA6339|EPI_ISL_13656114|United Kingdom|BA.5.1.3|2022-06-30	99,69
EPI_ISL_13656520	BA.4	A	A_3	LSPA-3EA6F71|EPI_ISL_13656520|United Kingdom|BA.4|2022-06-29	99,69
EPI_ISL_13657182	BA.5.2	A	A_3	BA-SANGLAHUNUD-C.17.0063|EPI_ISL_13657182|Indonesia|BA.5.2|2022-06-21	99,69
EPI_ISL_13657278	BA.2.76	BA.2.76	BA.2.76	TN-CDFD-J-857|EPI_ISL_13657278|India|BA.2.76|2022-06-07	86,93
EPI_ISL_13659921	BA.2	A	A_3	DL-ILBS-WGS5530|EPI_ISL_13659921|India|BA.2|2022-06-17	95,21
EPI_ISL_13662149	BA.2	BA.2	BA.2_1	GES-HMN-22062140063|EPI_ISL_13662149|France|BA.2|2022-06-07	99,81
EPI_ISL_13665743	BA.2.12.1	BA.2.12.1	BA.2.12.1	TX-HHD-2206298311|EPI_ISL_13665743|USA|BA.2.12.1|2022-06-21	95,19
EPI_ISL_13665777	BA.4.4	A	A_9	TX-HHD-2206299431|EPI_ISL_13665777|USA|BA.4.4|2022-06-22	95,06
EPI_ISL_13666028	BA.2.12.1	BA.2.12.1	BA.2.12.1	CT-Yale-20927|EPI_ISL_13666028|USA|BA.2.12.1|2022-06-20	99,81
EPI_ISL_13666626	BA.5.1	A	A_3	LNS3640343|EPI_ISL_13666626|Luxembourg|BA.5.1|2022-06-13	99,69
EPI_ISL_13667218	BA.5.1	A	A_3	LNS1610235|EPI_ISL_13667218|Luxembourg|BA.5.1|2022-06-21	99,69
EPI_ISL_13673152	BA.2.12.1	BA.2.12.1	BA.2.12.1	IA-SHL-2124614|EPI_ISL_13673152|USA|BA.2.12.1|2022-06-27	90,52
EPI_ISL_13677260	BA.1.1	BA.1.1	BA.1.1_2	MN-Mayo23392|EPI_ISL_13677260|USA|BA.1.1|2022-02-01	99,64
EPI_ISL_13686603	BA.2.12.1	BA.2.12.1	BA.2.12.1	NY-UB-KSL-05407|EPI_ISL_13686603|USA|BA.2.12.1|2022-04-30	95,17
EPI_ISL_13690852	BA.2.12.1	BA.2.12.1	BA.2.12.1	CA-LACPHL-AF11695|EPI_ISL_13690852|USA|BA.2.12.1|2022-06-02	90,52
EPI_ISL_13690914	BA.5.2.1	A	A_3	CA-LACPHL-AF11799|EPI_ISL_13690914|USA|BA.5.2.1|2022-06-10	90,40
EPI_ISL_13691472	BA.5.1	A	A_3	CO-CDPHE-2103388172|EPI_ISL_13691472|USA|BA.5.1|2022-06-20	99,69
EPI_ISL_13693425	BA.5.1	A	A_3	LSPA-3EA6BB9|EPI_ISL_13693425|United Kingdom|BA.5.1|2022-06-30	99,69
EPI_ISL_13694267	BF.1	A	A_3	EDB64270|EPI_ISL_13694267|United Kingdom|BF.1|2022-06-12	93,97
EPI_ISL_13694323	BA.5.1	A	A_9	PHEC-YYFIPUH|EPI_ISL_13694323|United Kingdom|BA.5.1|2022-06-23	99,69
EPI_ISL_13695715	BA.2.9	BA.2	BA.2_1	DCGC-543019|EPI_ISL_13695715|Denmark|BA.2.9|2022-07-01	99,79
EPI_ISL_13696918	BA.2.38	BA.2.38	BA.2.38	GJ-INSACOG-GBRC7099|EPI_ISL_13696918|India|BA.2.38|2022-05-19	99,81
EPI_ISL_13697604	BA.5.2.1	A	A_3	Ahus-3916|EPI_ISL_13697604|Norway|BA.5.2.1|2022-06-20	99,69
EPI_ISL_13698696	BA.5	A	A_9	NICD-R07190|EPI_ISL_13698696|South Africa|BA.5|2022-06-10	91,15
EPI_ISL_13699105	BA.2.12.1	BA.2.12.1	BA.2.12.1	NC-ECU-CORVASEQ-113915771|EPI_ISL_13699105|USA|BA.2.12.1|2022-06-17	99,79
EPI_ISL_13700860	BA.5.1	A	A_9	ZH-UZH-IMV-467d0823|EPI_ISL_13700860|Switzerland|BA.5.1|2022-06-16	99,69
EPI_ISL_13701860	BA.4.6	BA.4.6	BA.4.6_1	CA-HLX-STM-CZ3Y3MYP7|EPI_ISL_13701860|USA|BA.4.6|2022-06-20	97,46
EPI_ISL_13702858	BA.2.12.1	BA.1.15	BA.1.15	CA-HLX-STM-A8HDFQVZK|EPI_ISL_13702858|USA|BA.2.12.1|2022-06-22	96,24
EPI_ISL_13703820	BA.2.36	BA.2	BA.2_2	PUE_InDRE_FB15339_E21917174328_S13782|EPI_ISL_13703820|Mexico|BA.2.36|2022-06-14	99,81
EPI_ISL_13704075	BA.2.12.1	BA.2.12.1	BA.2.12.1	NM-NMDOH-2022076539|EPI_ISL_13704075|USA|BA.2.12.1|2022-05-07	99,81
EPI_ISL_13704776	BA.5.6	A	A_3	CA-CDC-QDX38153454|EPI_ISL_13704776|USA|BA.5.6|2022-06-16	99,69
EPI_ISL_13706282	BA.4.1	A	A_3	WA-CDC-LC0736031|EPI_ISL_13706282|USA|BA.4.1|2022-06-21	99,69
EPI_ISL_13706515	BA.2.12.1	BA.2.12.1	BA.2.12.1	WA-CDC-LC0736102|EPI_ISL_13706515|USA|BA.2.12.1|2022-06-21	99,81
EPI_ISL_13710186	BA.4.6	BA.4.6	BA.4.6_1	VIC65119|EPI_ISL_13710186|Australia|BA.4.6|2022-06-30	99,69
EPI_ISL_13711724	BA.4	A	A_3	LSPA-3EAEC54|EPI_ISL_13711724|United Kingdom|BA.4|2022-07-02	99,69
EPI_ISL_13712419	BA.2	A	A_14	QEUH-3EAB237|EPI_ISL_13712419|United Kingdom|BA.2|2022-06-29	99,81
EPI_ISL_13712479	BA.2.9	BA.2	BA.2_1	QEUH-3D1AE8B|EPI_ISL_13712479|United Kingdom|BA.2.9|2022-04-03	99,81
EPI_ISL_13712511	BA.2.9	BA.2	BA.2_1	QEUH-3D1B97A|EPI_ISL_13712511|United Kingdom|BA.2.9|2022-04-03	94,41
EPI_ISL_13712800	BA.2	A	A_14	QEUH-3D20839|EPI_ISL_13712800|United Kingdom|BA.2|2022-04-04	99,81
EPI_ISL_13714963	BA.5.2.1	BF.6	BF.6	PHEC-YYFI1HF|EPI_ISL_13714963|United Kingdom|BA.5.2.1|2022-06-21	99,68
EPI_ISL_13715729	BA.2.12.1	BA.2.12.1	BA.2.12.1	PHEC-YYFSU9H|EPI_ISL_13715729|United Kingdom|BA.2.12.1|2022-06-20	99,79
EPI_ISL_13717329	BA.2.37	A	A_14	PR-CVL-003053|EPI_ISL_13717329|Puerto Rico|BA.2.37|2022-05-06	90,35
EPI_ISL_13717918	BA.1.1	BA.1.1	BA.1.1_2	NLA_barcode61-15-MN908947_3|EPI_ISL_13717918|Kazakhstan|BA.1.1|2022-01-13	82,48
EPI_ISL_13719665	BA.2.3	A	A_14	NAMRU3_G793|EPI_ISL_13719665|Djibouti|BA.2.3|2022-04-27	99,81
EPI_ISL_13720764	BA.4.1	A	A_3	PA-CDC-LC0738549|EPI_ISL_13720764|USA|BA.4.1|2022-06-22	98,87
EPI_ISL_13720844	BA.2.12.1	BA.2.12.1	BA.2.12.1	KY-CDC-LC0739047|EPI_ISL_13720844|USA|BA.2.12.1|2022-06-22	99,81
EPI_ISL_13721586	BA.2.3.7	A	A_14	NSW-ICPMR-28746|EPI_ISL_13721586|Australia|BA.2.3.7|2022-06-28	78,97
EPI_ISL_13722642	BA.5.6	A	A_3	CA-CDC-LC0740115|EPI_ISL_13722642|USA|BA.5.6|2022-06-25	99,69
EPI_ISL_13724228	BA.5.5	BA.2.12.1	BA.2.12.1	CO-CDPHE-2103417347|EPI_ISL_13724228|USA|BA.5.5|2022-06-18	93,53
EPI_ISL_13724611	BA.2.9	A	A_17	TA-65983|EPI_ISL_13724611|Chile|BA.2.9|2022-05-25	99,77
EPI_ISL_13726029	BA.2.12.1	BA.2.12.1	BA.2.12.1	AN-70923|EPI_ISL_13726029|Chile|BA.2.12.1|2022-06-04	99,77
EPI_ISL_13726064	BA.4.1	BA.4.1	BA.4.1_1	LI-70564|EPI_ISL_13726064|Chile|BA.4.1|2022-06-05	99,66
EPI_ISL_13726684	BA.2.12.1	BA.2.12.1	BA.2.12.1	CA-SC-271264|EPI_ISL_13726684|USA|BA.2.12.1|2022-06-20	94,75
EPI_ISL_13727004	BA.5.1	A	A_3	HSGM-GS5684|EPI_ISL_13727004|Turkey|BA.5.1|2022-06-28	99,69
EPI_ISL_13727244	BA.2.12.1	BA.2.12.1	BA.2.12.1	LL-72855|EPI_ISL_13727244|Chile|BA.2.12.1|2022-06-07	99,77
EPI_ISL_13729630	BA.5.3.1	A	A_3	ICH-741236163|EPI_ISL_13729630|Israel|BA.5.3.1|2022-06-27	99,69
EPI_ISL_13730620	BF.5	A	A_25	ICH-741241804|EPI_ISL_13730620|Israel|BF.5|2022-07-03	95,67
EPI_ISL_13732353	BF.5	A	A_25	ICH-741240586|EPI_ISL_13732353|Israel|BF.5|2022-07-01	99,69
EPI_ISL_13733115	BA.4	BA.4.4	BA.4.4	LSPA-3EB514C|EPI_ISL_13733115|United Kingdom|BA.4|2022-07-02	99,69
EPI_ISL_13733434	BA.5.1	A	A_9	LSPA-3EB8FD6|EPI_ISL_13733434|United Kingdom|BA.5.1|2022-07-04	99,69
EPI_ISL_13736715	BA.5.2.1	A	A_3	RM-79179|EPI_ISL_13736715|Chile|BA.5.2.1|2022-06-21	99,66
EPI_ISL_13736974	BA.5.2.1	A	A_3	DCGC-544050|EPI_ISL_13736974|Denmark|BA.5.2.1|2022-07-03	99,69
EPI_ISL_13737344	BA.4.1	A	A_3	DCGC-544424|EPI_ISL_13737344|Denmark|BA.4.1|2022-07-03	99,69
EPI_ISL_13737601	BA.5.1	A	A_3	DCGC-544683|EPI_ISL_13737601|Denmark|BA.5.1|2022-07-04	99,69
EPI_ISL_13738505	BA.5.5	BA.5.5	BA.5.5_1	KDCA86618|EPI_ISL_13738505|South Korea|BA.5.5|2022-06-09	99,69
EPI_ISL_13739743	BA.5.2.1	A	A_3	QEUH-3EB3472|EPI_ISL_13739743|United Kingdom|BA.5.2.1|2022-07-01	99,69
EPI_ISL_13739896	BA.5.2	A	A_9	QEUH-3EB46D5|EPI_ISL_13739896|United Kingdom|BA.5.2|2022-06-30	99,68
EPI_ISL_13742890	BA.2.12.1	BA.2.12.1	BA.2.12.1	PHEP-YYF8W8M|EPI_ISL_13742890|United Kingdom|BA.2.12.1|2022-06-26	99,81

EPI_ISL_13743085	BA.5.2.1	A	A_3	PHEP-YYFGHXI|EPI_ISL_13743085|United Kingdom|BA.5.2.1|2022	99,62
EPI_ISL_13743181	BA.4	A	A_3	PHWC-PJ6YRA|EPI_ISL_13743181|United Kingdom|BA.4|2022-06-21	99,69
EPI_ISL_13746065	BA.5.1	A	A_3	NY-PRL-220705_06F19|EPI_ISL_13746065|USA|BA.5.1|2022-06-29	99,69
EPI_ISL_13746564	BA.2.12.1	BA.2.12.1	BA.2.12.1	NY-PRL-220627_02K22|EPI_ISL_13746564|USA|BA.2.12.1|2022-06-21	99,81
EPI_ISL_13747428	BA.5.1	A	A_3	THL-202214197|EPI_ISL_13747428|Finland|BA.5.1|2022-05-26	99,69
EPI_ISL_13747700	BA.2.12.1	BA.2.12.1	BA.2.12.1	NC-MCPH-CLT-009427|EPI_ISL_13747700|USA|BA.2.12.1|2022-06-20	99,79
EPI_ISL_13749653	BE.1.1	A	A_3	NY-PRL-220630_00J19|EPI_ISL_13749653|USA|BE.1.1|2022-06-24	99,69
EPI_ISL_13749785	BA.5.1.3	BA.5.1.3	BA.5.1.3	NY-PRL-220705_01F20|EPI_ISL_13749785|USA|BA.5.1.3|2022-06-23	99,69
EPI_ISL_13750388	BA.2	A	A_14	NIC_SKW_20899|EPI_ISL_13750388|Thailand|BA.2|2022-06-15	99,81
EPI_ISL_13754867	BA.4.1	A	A_3	CO-USAFSAM-S17623|EPI_ISL_13754867|USA|BA.4.1|2022-06-22	99,69
EPI_ISL_13756172	BE.3	A	A_3	ON-PHL-22-26969|EPI_ISL_13756172|Canada|BE.3|2022-06-27	99,69
EPI_ISL_13756334	BA.5.2.1	A	A_3	ON-PHL-22-27404|EPI_ISL_13756334|Canada|BA.5.2.1|2022-06-28	99,69
EPI_ISL_13757452	BA.2.12.1	BA.2.12.1	BA.2.12.1	TX-CDC-STM-W8RUWV6S2|EPI_ISL_13757452|USA|BA.2.12.1|2022-06-23	99,81
EPI_ISL_13757896	BA.2	A	A_14	TIDREC-NSC1595|EPI_ISL_13757896|Malaysia|BA.2|2022-05-22	99,43
EPI_ISL_13759123	BA.4.1	A	A_3	IN-CDC-STM-V8J8TUSJK|EPI_ISL_13759123|USA|BA.4.1|2022-06-27	99,69
EPI_ISL_13759562	BA.2.12.1	BA.2.12.1	BA.2.12.1	TN-CDC-STM-M5M9H95J4|EPI_ISL_13759562|USA|BA.2.12.1|2022-06-28	99,81
EPI_ISL_13759633	BA.5.5	BA.5.5	BA.5.5_1	IL-CDC-STM-UDKV6JGW8|EPI_ISL_13759633|USA|BA.5.5|2022-06-29	99,69
EPI_ISL_13760010	BA.4	A	A_3	UT-RIVM-104263|EPI_ISL_13760010|Netherlands|BA.4|2022-06-21	99,69
EPI_ISL_13760579	BA.5.6	A	A_3	ZH-RIVM-104090|EPI_ISL_13760579|Netherlands|BA.5.6|2022-06-21	99,69
EPI_ISL_13761293	BA.5.1	A	A_3	PT38354|EPI_ISL_13761293|Portugal|BA.5.1|2022-06-25	99,69
EPI_ISL_13761392	BA.5.1.2	A	A_3	PT38704|EPI_ISL_13761392|Portugal|BA.5.1.2|2022-06-24	99,69
EPI_ISL_13761459	BA.5.1	A	A_3	PT38771|EPI_ISL_13761459|Portugal|BA.5.1|2022-06-28	99,69
EPI_ISL_13762811	BA.2.3.13	A	A_4	YCH1302|EPI_ISL_13762811|Japan|BA.2.3.13|2022-07-06	99,81
EPI_ISL_13763413	BA.5.5	BA.5.5	BA.5.5_1	UT-UPHL-220708816367|EPI_ISL_13763413|USA|BA.5.5|2022-06-27	99,69
EPI_ISL_13764226	BA.5.2.1	A	A_3	22MV3442|EPI_ISL_13764226|New Zealand|BA.5.2.1|2022-06-28	99,68
EPI_ISL_13764447	BA.2	BA.2.10	BA.2.10	22CV6105|EPI_ISL_13764447|New Zealand|BA.2|2022-06-14	99,79
EPI_ISL_13768152	BA.5.1	A	A_3	LSPA-3EC68E9|EPI_ISL_13768152|United Kingdom|BA.5.1|2022-07-05	99,69
EPI_ISL_13769604	BA.5.1	A	A_3	GA-CHOP-51906661|EPI_ISL_13769604|Spain|BA.5.1|2022-06-22	95,44
EPI_ISL_13769918	BA.2	BA.2	BA.2_1	GA-CHUVI-19544670|EPI_ISL_13769918|Spain|BA.2|2022-06-14	99,81
EPI_ISL_13772485	BA.4	A	A_9	DCGC-545425|EPI_ISL_13772485|Denmark|BA.4|2022-07-05	99,69
EPI_ISL_13773297	BA.5.2	A	A_3	8214|EPI_ISL_13773297|Singapore|BA.5.2|2022-07-04	99,69
EPI_ISL_13773352	BA.4.1	A	A_3	GES-IPP41737|EPI_ISL_13773352|France|BA.4.1|2022-06-20	99,22
EPI_ISL_13773472	BA.5.2.1	A	A_3	IDF-IPP42192|EPI_ISL_13773472|France|BA.5.2.1|2022-06-27	99,69
EPI_ISL_13774511	BA.5.1	A	A_3	UZA-UA-CV8520307319|EPI_ISL_13774511|Belgium|BA.5.1|2022-06-30	99,69
EPI_ISL_13774785	BA.5.3	A	A_3	38282|EPI_ISL_13774785|Croatia|BA.5.3|2022-06-10	98,51
EPI_ISL_13775053	BA.2	BA.2	BA.2_1	D-NVRL-ecS22IRL00324459|EPI_ISL_13775053|Ireland|BA.2|2022-05-11	92,46
EPI_ISL_13775288	BA.2.23	BA.2	BA.2_1	D-NVRL-ecS22IRL00327920|EPI_ISL_13775288|Ireland|BA.2.23|2022-05-17	96,53
EPI_ISL_13775608	BA.2	BA.2	BA.2_1	MH-NVRL-ecS22IRL00323143|EPI_ISL_13775608|Ireland|BA.2|2022-05-09	96,30
EPI_ISL_13775809	BA.5.2.1	A	A_3	SMC-7087917|EPI_ISL_13775809|Israel|BA.5.2.1|2022-07-04	99,69
EPI_ISL_13775871	BE.1	A	A_3	SMC-7087647|EPI_ISL_13775871|Israel|BE.1|2022-07-05	96,36
EPI_ISL_13779488	BA.2.76	BA.2.76	BA.2.76	WB-INSACOG-1931503196273|EPI_ISL_13779488|India|BA.2.76|2022-06-18	99,81
EPI_ISL_13780427	BA.2.12.1	BA.2.12.1	BA.2.12.1	RI-CDC-LC0742562|EPI_ISL_13780427|USA|BA.2.12.1|2022-06-23	99,81
EPI_ISL_13782031	BA.4.1	A	A_3	MD-HP34358-PIDLSZQINA|EPI_ISL_13782031|USA|BA.4.1|2022-06-27	99,69
EPI_ISL_13783408	BA.2.12.1	BA.2.12.1	BA.2.12.1	AR-PHL-2216500000133-31-F1-1|EPI_ISL_13783408|USA|BA.2.12.1|2022-06-14	99,81
EPI_ISL_13784696	BA.2.12.1	B.1.1.228	B.1.1.228	DC-Curative-425589|EPI_ISL_13784696|USA|BA.2.12.1|2022-06-25	82,79
EPI_ISL_13785053	BA.2.12.1	BA.2.12.1	BA.2.12.1	VA-Curative-403473|EPI_ISL_13785053|USA|BA.2.12.1|2022-06-28	92,67
EPI_ISL_13786470	BA.2	BA.2	BA.2_1	VA-CAV_VAS3N_00010750_01|EPI_ISL_13786470|USA|BA.2|2022-05-04	99,81
EPI_ISL_13786798	BA.1.1	BA.1.1	BA.1.1_3	CA-CDPH-3000337678|EPI_ISL_13786798|USA|BA.1.1|2022-02-07	75,35
EPI_ISL_13788257	BA.1.1	BA.1.1	BA.1.1_2	CA-CDPH-3000294206|EPI_ISL_13788257|USA|BA.1.1|2021-12-30	99,64
EPI_ISL_13788277	BA.1.1	BA.1	BA.1_4	CA-CDPH-3000294381|EPI_ISL_13788277|USA|BA.1.1|2021-12-22	81,95
EPI_ISL_13789370	BA.1.1	BA.1.1	BA.1.1_2	CA-CDPH-3000302446|EPI_ISL_13789370|USA|BA.1.1|2022-01-17	98,61
EPI_ISL_13790094	BA.1.1	BA.1	BA.1_4	CA-CDPH-3000303676|EPI_ISL_13790094|USA|BA.1.1|2022-01-27	85,38
EPI_ISL_13790393	BA.1	BA.1	BA.1_3	CA-CDPH-3000304474|EPI_ISL_13790393|USA|BA.1|2022-01-30	98,17
EPI_ISL_13790535	BA.1.1	BA.1.1	BA.1.1_2	CA-CDPH-3000305589|EPI_ISL_13790535|USA|BA.1.1|2022-02-01	99,64
EPI_ISL_13791956	BA.1.1	BA.1	BA.1_4	CA-CDPH-3000311757|EPI_ISL_13791956|USA|BA.1.1|2022-02-09	79,58
EPI_ISL_13792108	BA.1.1.18	BA.1.1	BA.1.1_2	CA-CDPH-3000312406|EPI_ISL_13792108|USA|BA.1.1.18|2022-02-08	85,37
EPI_ISL_13792286	BA.1.1.18	BA.1.1	BA.1.1_3	CA-CDPH-3000313268|EPI_ISL_13792286|USA|BA.1.1.18|2022-02-10	80,92
EPI_ISL_13793861	BA.1.1	BA.1	BA.1_4	CA-CDPH-3000341801|EPI_ISL_13793861|USA|BA.1.1|2022-03-03	78,88
EPI_ISL_13794288	BA.1.1	BA.1.1	BA.1.1_2	CA-CDPH-3000321443|EPI_ISL_13794288|USA|BA.1.1|2022-01-31	93,91
EPI_ISL_13794507	BA.1.1	BA.1.1	BA.1.1_2	CA-CDPH-3000341322|EPI_ISL_13794507|USA|BA.1.1|2022-03-03	93,82
EPI_ISL_13796843	BA.2	BA.2.13	BA.2.13	NE-CUMC_22185057|EPI_ISL_13796843|USA|BA.2|2022-06-29	99,81
EPI_ISL_13797363	BA.1.1	BA.1.15	BA.1.15	CA-CDPH-500043575|EPI_ISL_13797363|USA|BA.1.1|2021-12-29	68,75
EPI_ISL_13798315	BA.5.2.1	A	A_3	PHEC-YYF3RMQ|EPI_ISL_13798315|United Kingdom|BA.5.2.1|2022	99,68
EPI_ISL_13798685	BA.4.1	BA.4.1	BA.4.1_1	PHEP-YYF8HAY|EPI_ISL_13798685|United Kingdom|BA.4.1|2022-06-27	96,24
EPI_ISL_13800600	BA.2	BA.2	BA.2_1	208931|EPI_ISL_13800600|Romania|BA.2|2022-04-14	99,81
EPI_ISL_13800707	BA.5	BA.5.3.2	BA.5.3.2	LNS6706318|EPI_ISL_13800707|Luxembourg|BA.5|2022-06-27	99,69
EPI_ISL_13800940	BA.5.1	A	A_9	LNS5497851|EPI_ISL_13800940|Luxembourg|BA.5.1|2022-06-27	99,69
EPI_ISL_13801573	BA.5.2	A	A_9	OCC-CHU-TLS-2217812507|EPI_ISL_13801573|France|BA.5.2|2022-06-27	72,09
EPI_ISL_13802914	BA.2	BA.2	BA.2_1	ROO_LANGEBIO_IMSS_9264|EPI_ISL_13802914|Mexico|BA.2|2022-06-22	97,88
EPI_ISL_13803593	BA.1.1	BA.1.1	BA.1.1_2	rega-31332|EPI_ISL_13803593|Belgium|BA.1.1|2022-02-07	96,16
EPI_ISL_13803663	BA.2	BA.2	BA.2_1	rega-31401|EPI_ISL_13803663|Belgium|BA.2|2022-02-09	90,52
EPI_ISL_13805465	BA.5.2	A	A_3	RC0708139|EPI_ISL_13805465|Estonia|BA.5.2|2022-06-07	98,51
EPI_ISL_13805643	BA.5.1	A	A_9	FVG-PN-64045678|EPI_ISL_13805643|Italy|BA.5.1|2022-07-05	99,69
EPI_ISL_13806813	BA.2.38	BA.2.38	BA.2.38	CA-CDC-STM-3Z279AS9C|EPI_ISL_13806813|USA|BA.2.38|2022-06-30	99,81
EPI_ISL_13807791	BA.2.12.1	BA.2.12.1	BA.2.12.1	WA-CDC-LC0749838|EPI_ISL_13807791|USA|BA.2.12.1|2022-06-27	99,01
EPI_ISL_13807804	BA.5.2	A	A_9	CA-CDC-LC0749154|EPI_ISL_13807804|USA|BA.5.2|2022-06-27	99,69
EPI_ISL_13808951	BA.5.2.1	A	A_3	AZ-CDC-LC0752044|EPI_ISL_13808951|USA|BA.5.2.1|2022-06-28	99,69
EPI_ISL_13808981	BA.5.5	BA.5.5	BA.5.5_1	CA-CDC-LC0750645|EPI_ISL_13808981|USA|BA.5.5|2022-06-28	99,69
EPI_ISL_13809589	BA.5.5	BA.5.5	BA.5.5_1	NC-CDC-LC0748328|EPI_ISL_13809589|USA|BA.5.5|2022-06-29	99,69
EPI_ISL_13810983	BA.4.1	A	A_3	CA-CDC-LC0753936|EPI_ISL_13810983|USA|BA.4.1|2022-07-01	99,69
EPI_ISL_13811371	BA.2.12.1	BA.2.12.1	BA.2.12.1	CA-CDC-LC0754390|EPI_ISL_13811371|USA|BA.2.12.1|2022-07-04	99,81
EPI_ISL_13811470	BE.1.1	A	A_9	ULG-27197|EPI_ISL_13811470|Belgium|BE.1.1|2022-07-05	99,68
EPI_ISL_13811514	BA.5.2.1	A	A_3	ULG-27241|EPI_ISL_13811514|Belgium|BA.5.2.1|2022-07-04	99,68
EPI_ISL_13811528	BA.5.1	A	A_3	ULG-27255|EPI_ISL_13811528|Belgium|BA.5.1|2022-07-06	99,68
EPI_ISL_13811582	BA.2.12.1	BA.2.12.1	BA.2.12.1	SD-SDPHL-1412|EPI_ISL_13811582|USA|BA.2.12.1|2022-06-14	98,49
EPI_ISL_13812380	BA.2.3.6	BA.2.3.6	BA.2.3.6	CA-LACPHL-AF12144|EPI_ISL_13812380|USA|BA.2.3.6|2022-05-14	90,50
EPI_ISL_13813397	BA.5.1	A	A_9	NC-CORVASEQ-CLT-009880|EPI_ISL_13813397|USA|BA.5.1|2022-06-29	99,68
EPI_ISL_13815424	BA.1.1	BA.1.1	BA.1.1_3	CA-CDPH-3000329563|EPI_ISL_13815424|USA|BA.1.1|2022-02-02	82,66
EPI_ISL_13815669	BA.1.1	BA.1.1	BA.1.1_2	CA-CDPH-3000337303|EPI_ISL_13815669|USA|BA.1.1|2022-02-07	88,95
EPI_ISL_13819441	BA.5.6	A	A_3	LIM-INS-20855|EPI_ISL_13819441|Peru|BA.5.6|2022-06-22	99,68
EPI_ISL_13819761	BA.2.12.1	BA.2.12.1	BA.2.12.1	HI-H2213026|EPI_ISL_13819761|USA|BA.2.12.1|2022-06-24	99,50
EPI_ISL_13820628	BA.1.20	BA.1	BA.1_4	CA-CDPH-3000318181|EPI_ISL_13820628|USA|BA.1.20|2022-02-17	89,28
EPI_ISL_13820860	BA.1	BA.1	BA.1_4	CA-CDPH-3000320102|EPI_ISL_13820860|USA|BA.1|2022-02-14	88,53
EPI_ISL_13821250	BA.2.12.1	BA.2.12.1	BA.2.12.1	LIM-INS-21144|EPI_ISL_13821250|Peru|BA.2.12.1|2022-06-28	92,73
EPI_ISL_13821432	BA.5.1	A	A_3	LIM-INS-21326|EPI_ISL_13821432|Peru|BA.5.1|2022-06-23	99,68
EPI_ISL_13822062	BA.2.12.1	BA.2.12.1	BA.2.12.1	CMX-INMEGEN-79-163|EPI_ISL_13822062|Mexico|BA.2.12.1|2022-06-30	83,40
EPI_ISL_13822249	BA.4.1	BA.4.1	BA.4.1_1	PR-CVL-004543|EPI_ISL_13822249|Puerto Rico|BA.4.1|2022-06-01	90,38
EPI_ISL_13822647	BA.5.2	A	A_9	PIE_IRCC_15885127|EPI_ISL_13822647|Italy|BA.5.2|2022-07-05	99,08
EPI_ISL_13823453	BA.5.1	A	A_3	EDB64898|EPI_ISL_13823453|United Kingdom|BA.5.1|2022-06-22	99,69
EPI_ISL_13824944	BA.5.6	A	A_3	26838|EPI_ISL_13824944|Greece|BA.5.6|2022-06-14	98,51
EPI_ISL_13824991	BA.4.1	A	A_3	26888_2|EPI_ISL_13824991|Greece|BA.4.1|2022-06-14	98,51

EPI_ISL_13825937	BA.5	A	A_9	LB-R00087-S046|EPI_ISL_13825937|Austria|BA.5|2022-06-28	98,57
EPI_ISL_13826582	BA.2.9	BA.2	BA.2_1	LAZ-AMC-220504754-DS|EPI_ISL_13826582|Italy|BA.2.9|2022-05-04	98,68
EPI_ISL_13826857	BA.2.56	BA.2.56	BA.2.56	LAZ-AMC-220623819-DS|EPI_ISL_13826857|Italy|BA.2.56|2022-06-23	99,81
EPI_ISL_13827067	BA.2.3	BA.2	BA.2_1	6115611-02|EPI_ISL_13827067|Estonia|BA.2.3|2022-06-15	98,51
EPI_ISL_13829172	Unassigned	A	A_9	SIC-AOUME-UOSD-GCL-299904915-4854|EPI_ISL_13829172|Italy|Unassigned|2022-07-05	94,60
EPI_ISL_13829463	BA.2	BA.2	BA.2_1	AS-252241623|EPI_ISL_13829463|Spain|BA.2|2022-06-29	99,81
EPI_ISL_13830847	BA.5.2.1	A	A_3	CA-OC-3095|EPI_ISL_13830847|USA|BA.5.2.1|2022-06-24	99,60
EPI_ISL_13831277	BA.2	BA.4.6	BA.4.6_1	CA-HLX-STM-TG9N23RCA|EPI_ISL_13831277|USA|BA.2|2022-06-24	97,61
EPI_ISL_13831607	Unassigned	BA.1.9	BA.1.9	CA-HLX-STM-CEFU3F2B5|EPI_ISL_13831607|USA|Unassigned|2022-06-16	92,67
EPI_ISL_13833275	BA.5.5	BA.5.5	BA.5.5_1	KS-KHEL-11332|EPI_ISL_13833275|USA|BA.5.5|2022-07-06	90,40
EPI_ISL_13833628	BA.1.1	BA.1.1	BA.1.1_2	ES-LACENES-321117357|EPI_ISL_13833628|Brazil|BA.1.1|2022-01-07	99,64
EPI_ISL_13833938	BA.2.12.1	BA.2.12.1	BA.2.12.1	CA-CDPH-6000011136|EPI_ISL_13833938|USA|BA.2.12.1|2022-05-03	90,52
EPI_ISL_13834085	BA.2.12.1	BA.2.12.1	BA.2.12.1	CA-CDPH-6000011682|EPI_ISL_13834085|USA|BA.2.12.1|2022-06-15	90,52
EPI_ISL_13839652	BA.2.12.1	BA.2.12.1	BA.2.12.1	INC-LNSP-233|EPI_ISL_13839652|El Salvador|BA.2.12.1|2022-06-02	99,81
EPI_ISL_13841570	BA.5.2.1	A	A_3	PHEC-YYF4QD1|EPI_ISL_13841570|United Kingdom|BA.5.2.1|2022-07-01	99,66
EPI_ISL_13843908	BA.5.2.3	A	A_3	77SVUPHA_7769580479|EPI_ISL_13843908|Czech Republic|BA.5.2.3|2022-06-13	99,68
EPI_ISL_13845589	BA.5.1	A	A_9	UZA-UA-MI22271595|EPI_ISL_13845589|Belgium|BA.5.1|2022-07-08	99,69
EPI_ISL_13846439	Unassigned	A	A_3	NH-inBiome-211850|EPI_ISL_13846439|Netherlands|Unassigned|2022-07-09	90,38
EPI_ISL_13848599	BA.2.12.1	BA.2.12.1	BA.2.12.1	AZ-CDC-STM-3QPY2K6ZV|EPI_ISL_13848599|USA|BA.2.12.1|2022-07-01	99,81
EPI_ISL_13849979	BA.2.12.1	BA.2.12.1	BA.2.12.1	NC-CORVASEQ-UNC-7112|EPI_ISL_13849979|USA|BA.2.12.1|2022-07-05	99,81
EPI_ISL_13850037	BA.5.3	A	A_3	MN-MDH-27432|EPI_ISL_13850037|USA|BA.5.3|2022-06-30	99,69
EPI_ISL_13850326	BA.2.12.1	BA.2.12.1	BA.2.12.1	NY-NYCPHL-013409|EPI_ISL_13850326|USA|BA.2.12.1|2022-06-08	90,52
EPI_ISL_13854018	BA.2.12.1	BA.2.12.1	BA.2.12.1	BFC-HMN-22062300278|EPI_ISL_13854018|France|BA.2.12.1|2022-06-20	99,54
EPI_ISL_13855115	BA.5.1.3	BA.5.1.3	BA.5.1.3	OV-RIVM-105470|EPI_ISL_13855115|Netherlands|BA.5.1.3|2022-07-04	99,64
EPI_ISL_13856621	BA.4.1	A	A_3	MS-MSPHL-1319|EPI_ISL_13856621|USA|BA.4.1|2022-07-05	99,69
EPI_ISL_13858304	BA.5.2	A	A_3	CE-FIOCRUZ-94166CE|EPI_ISL_13858304|Brazil|BA.5.2|2022-06-24	99,69
EPI_ISL_13859166	BA.2	BA.2	BA.2_1	QLD0x00FD6F|EPI_ISL_13859166|Australia|BA.2|2022-06-14	99,81
EPI_ISL_13859738	BA.4.1	A	A_3	MN-CDC-2-6167026|EPI_ISL_13859738|USA|BA.4.1|2022-06-19	99,69
EPI_ISL_13862460	BA.2.12.1	BA.2.12.1	BA.2.12.1	NY-NYULH7524|EPI_ISL_13862460|USA|BA.2.12.1|2022-06-21	91,93
EPI_ISL_13863353	BA.2.12.1	BA.2.12.1	BA.2.12.1	VA-CDC-LC0755931|EPI_ISL_13863353|USA|BA.2.12.1|2022-06-27	99,81
EPI_ISL_13863444	BA.5.1	A	A_3	NJ-CDC-LC0755713|EPI_ISL_13863444|USA|BA.5.1|2022-06-28	99,69
EPI_ISL_13863705	BA.2	A	A_4	NJ-CDC-LC0755836|EPI_ISL_13863705|USA|BA.2|2022-06-29	99,64
EPI_ISL_13863862	BA.5.2.1	A	A_3	WI-CDC-LC0756334|EPI_ISL_13863862|USA|BA.5.2.1|2022-06-30	99,69
EPI_ISL_13864299	BA.5.2	A	A_3	KY-CDC-LC0757176|EPI_ISL_13864299|USA|BA.5.2|2022-07-03	99,69
EPI_ISL_13865241	BA.5.1	A	A_3	BA_22_00032309|EPI_ISL_13865241|Slovakia|BA.5.1|2022-06-19	99,69
EPI_ISL_13865687	BA.2.12.1	BA.2.12.1	BA.2.12.1	CO-CDPHE-2103417067|EPI_ISL_13865687|USA|BA.2.12.1|2022-06-20	92,69
EPI_ISL_13866204	BA.4.1	A	A_3	CO-CDPHE-2103399506|EPI_ISL_13866204|USA|BA.4.1|2022-06-20	99,69
EPI_ISL_6647962	BA.1	BA.1.15	BA.1.15	NICD-N21607-DX64624|EPI_ISL_6647962|South Africa|BA.1|2021-11-16	96,62
EPI_ISL_7574872	BA.1.15	BA.1.15	BA.1.15	MILK-2D71EA3|EPI_ISL_7574872|United Kingdom|BA.1.15|2021-12-06	90,98
EPI_ISL_7720982	BA.1.17.2	BA.1.17.2	BA.1.17.2	BRBR-2E02B18|EPI_ISL_7720982|United Kingdom|BA.1.17.2|2021-12-10	96,03
EPI_ISL_7762763	BA.1	BA.1.15	BA.1.15	MILK-2E0FDF7|EPI_ISL_7762763|United Kingdom|BA.1|2021-12-09	90,98
EPI_ISL_7762856	BA.1.15.1	BA.1.15	BA.1.15	MILK-2E0FC27|EPI_ISL_7762856|United Kingdom|BA.1.15.1|2021-12-10	94,09
EPI_ISL_7818384	BA.1.1	BA.1.1	BA.1.1_2	MILK-2E25757|EPI_ISL_7818384|United Kingdom|BA.1.1|2021-12-10	96,03
EPI_ISL_7834134	BA.1	BA.1	BA.1_4	DC-Curative-129089|EPI_ISL_7834134|USA|BA.1|2021-12-07	96,03
EPI_ISL_7837956	BA.1	BA.1	BA.1_4	MILK-2E5340E|EPI_ISL_7837956|United Kingdom|BA.1|2021-12-12	96,03
EPI_ISL_7841923	BA.1.15.1	BA.1.15	BA.1.15	MILK-2E55AEC|EPI_ISL_7841923|United Kingdom|BA.1.15.1|2021-12-11	96,03
EPI_ISL_7842061	BA.1	BA.1.15	BA.1.15	QEUH-2E4D391|EPI_ISL_7842061|United Kingdom|BA.1|2021-12-11	90,98
EPI_ISL_7863686	BA.1.15.1	BA.1.15	BA.1.15	MILK-2E82CA4|EPI_ISL_7863686|United Kingdom|BA.1.15.1|2021-12-13	90,98
EPI_ISL_7865452	BA.1.15	BA.1.15	BA.1.15	MILK-2E7713A|EPI_ISL_7865452|United Kingdom|BA.1.15|2021-12-13	90,98
EPI_ISL_7877429	BA.1.17.2	BA.1.17.2	BA.1.17.2	9080|EPI_ISL_7877429|Singapore|BA.1.17.2|2021-12-14	99,64
EPI_ISL_7881898	BA.1.17	A	A_1	Tygerberg_3413|EPI_ISL_7881898|South Africa|BA.1.17|2021-12-08	61,10
EPI_ISL_7886760	BA.1.20	BA.1.15	BA.1.15	WI-UW-7270|EPI_ISL_7886760|USA|BA.1.20|2021-12-16	99,64
EPI_ISL_7893311	BA.1.16	BA.1	BA.1_3	MILK-2EA9ABC|EPI_ISL_7893311|United Kingdom|BA.1.16|2021-12-14	96,03
EPI_ISL_7893633	BA.1	BA.1	BA.1_3	ALDP-2EA6ABF|EPI_ISL_7893633|United Kingdom|BA.1|2021-12-13	95,55
EPI_ISL_7895509	BA.1.17.2	BA.1.17.2	BA.1.17.2	MILK-2E8122B|EPI_ISL_7895509|United Kingdom|BA.1.17.2|2021-12-13	96,03
EPI_ISL_7896668	BA.1	BA.1	BA.1_3	MILK-2E727C4|EPI_ISL_7896668|United Kingdom|BA.1|2021-12-13	96,03
EPI_ISL_7896817	BA.1.17	BA.1	BA.1_4	MD-HRYC-47501771|EPI_ISL_7896817|Spain|BA.1.17|2021-12-09	99,64
EPI_ISL_7901915	BA.1.17.2	BA.1	BA.1_4	DCGC-267987|EPI_ISL_7901915|Denmark|BA.1.17.2|2021-12-10	96,34
EPI_ISL_7906407	BA.1.17.2	BA.1.15	BA.1.15	DCGC-270927|EPI_ISL_7906407|Denmark|BA.1.17.2|2021-12-05	96,57
EPI_ISL_7906867	BA.1	BA.1.15	BA.1.15	DCGC-271398|EPI_ISL_7906867|Denmark|BA.1|2021-12-08	93,97
EPI_ISL_7956039	BA.1	BA.1.15	BA.1.15	MILK-2EC6819|EPI_ISL_7956039|United Kingdom|BA.1|2021-12-14	94,09
EPI_ISL_7956821	BA.1.15	BA.1.15	BA.1.15	BRBR-2EC9AF2|EPI_ISL_7956821|United Kingdom|BA.1.15|2021-12-13	90,98
EPI_ISL_7956867	BA.1.16	BA.1.15	BA.1.15	BRBR-2EC9DDB|EPI_ISL_7956867|United Kingdom|BA.1.16|2021-12-14	94,09
EPI_ISL_7962439	BA.1	BA.1	BA.1_3	QEUH-2EBE191|EPI_ISL_7962439|United Kingdom|BA.1|2021-12-15	95,97
EPI_ISL_7962724	BA.1	BA.1.15	BA.1.15	MILK-2EBEDE8|EPI_ISL_7962724|United Kingdom|BA.1|2021-12-14	94,09
EPI_ISL_7969023	BA.1	BA.1	BA.1_3	ALDP-2E91DBE|EPI_ISL_7969023|United Kingdom|BA.1|2021-12-12	96,03
EPI_ISL_7972737	BA.1.17.2	BA.1.17.2	BA.1.17.2	QEUH-2EB6A34|EPI_ISL_7972737|United Kingdom|BA.1.17.2|2021-12-14	93,70
EPI_ISL_7974011	BA.1	BA.1	BA.1_3	ALDP-2EAFF20|EPI_ISL_7974011|United Kingdom|BA.1|2021-12-12	96,03
EPI_ISL_7980819	BA.1	BA.1	BA.1_1	MA-CDCBI-CRSP_LLUOH7MLCFN6LNQK|EPI_ISL_7980819|USA|BA.1|2021-12-12	95,10
EPI_ISL_7987732	BA.1.17	BA.1.15	BA.1.15	NSW-ICPMR-17908|EPI_ISL_7987732|Australia|BA.1.17|2021-12-13	94,05
EPI_ISL_7991183	BA.1.17.2	BA.1.17.2	BA.1.17.2	ALDP-2EDBB27|EPI_ISL_7991183|United Kingdom|BA.1.17.2|2021-12-14	94,09
EPI_ISL_7992603	BA.1	BA.1.15	BA.1.15	MILK-2EEBB60|EPI_ISL_7992603|United Kingdom|BA.1|2021-12-15	90,98
EPI_ISL_8017638	BA.1	BA.1.15	BA.1.15	CT-JAX-JAX-2151-088|EPI_ISL_8017638|USA|BA.1|2021-12-16	92,65
EPI_ISL_8021994	BA.1	BA.1	BA.1_1	CA-CDC-FG-193826|EPI_ISL_8021994|USA|BA.1|2021-12-14	99,52
EPI_ISL_8040649	BA.1	BA.1.15	BA.1.15	MILK-2F146E4|EPI_ISL_8040649|United Kingdom|BA.1|2021-12-14	90,98
EPI_ISL_8040651	BA.1.15.1	BA.1.15	BA.1.15	MILK-2F15795|EPI_ISL_8040651|United Kingdom|BA.1.15.1|2021-12-16	94,09
EPI_ISL_8041184	BA.1.15.1	BA.1.15	BA.1.15	QEUH-2F18659|EPI_ISL_8041184|United Kingdom|BA.1.15.1|2021-12-16	96,03
EPI_ISL_8067220	BA.1.17.2	BA.1.17.2	BA.1.17.2	MILK-2F449C4|EPI_ISL_8067220|United Kingdom|BA.1.17.2|2021-12-16	96,03
EPI_ISL_8067240	BA.1.17.2	BA.1.17.2	BA.1.17.2	QEUH-2F3F73B|EPI_ISL_8067240|United Kingdom|BA.1.17.2|2021-12-18	96,03
EPI_ISL_8072567	BA.1	BA.1.15	BA.1.15	BRBR-2F245C6|EPI_ISL_8072567|United Kingdom|BA.1|2021-12-17	90,99
EPI_ISL_8076391	BA.1	BA.1.15	BA.1.15	PHEP-YYNWRPZ|EPI_ISL_8076391|United Kingdom|BA.1|2021-12-09	84,47
EPI_ISL_8077447	BA.1.15	BA.1.15	BA.1.15	TX-CDC-ASC210534035|EPI_ISL_8077447|USA|BA.1.15|2021-12-12	99,64
EPI_ISL_8078730	BA.1.20	BA.1	BA.1_4	CO-CDC-MMB11844971|EPI_ISL_8078730|USA|BA.1.20|2021-11-26	96,99
EPI_ISL_8080553	BA.1.1	BA.1.1	BA.1.1_2	CA-CDC-FG-198073|EPI_ISL_8080553|USA|BA.1.1|2021-12-16	88,17
EPI_ISL_8080931	BA.1.18	BA.1	BA.1_3	CA-CDC-FG-197476|EPI_ISL_8080931|USA|BA.1.18|2021-12-16	97,18
EPI_ISL_8080947	BA.1	BA.1.15	BA.1.15	CA-CDC-FG-197704|EPI_ISL_8080947|USA|BA.1|2021-12-16	99,52
EPI_ISL_8082219	BA.1.15	BA.1.15	BA.1.15	TX-CDC-ASC210611728|EPI_ISL_8082219|USA|BA.1.15|2021-12-13	99,64
EPI_ISL_8083834	BA.1	BA.1.15	BA.1.15	LA-BIE-LSUH002338|EPI_ISL_8083834|USA|BA.1|2021-12-14	79,66
EPI_ISL_8084023	BA.1.17.2	BA.1.17.2	BA.1.17.2	PLYM-2F4A81C|EPI_ISL_8084023|United Kingdom|BA.1.17.2|2021-12-18	94,09
EPI_ISL_8084541	BA.1	BA.1.15	BA.1.15	HSLL-2F6476A|EPI_ISL_8084541|United Kingdom|BA.1|2021-12-19	90,98
EPI_ISL_8088181	BA.1	BA.1.15	BA.1.15	BRBR-2F3E5AB|EPI_ISL_8088181|United Kingdom|BA.1|2021-12-18	94,09
EPI_ISL_8091492	BA.1.15	BA.1	BA.1_1	CA-CDC-FG-200334|EPI_ISL_8091492|USA|BA.1.15|2021-12-17	99,52
EPI_ISL_8092379	BA.1.1	BA.1.1	BA.1.1_2	CA-CDC-FG-199933|EPI_ISL_8092379|USA|BA.1.1|2021-12-17	99,52
EPI_ISL_8092451	BA.1.1	BA.1.1	BA.1.1_2	CA-CDC-FG-199884|EPI_ISL_8092451|USA|BA.1.1|2021-12-17	98,57
EPI_ISL_8092534	BA.1.15	BA.1	BA.1_2	TX-CDC-FG-199530|EPI_ISL_8092534|USA|BA.1.15|2021-12-18	99,52
EPI_ISL_8092872	BA.1.1	BA.1.1	BA.1.1_2	MBLG-CTMAPF21834881|EPI_ISL_8092872|Belgium|BA.1.1|2021-12-16	99,64
EPI_ISL_8092920	BA.1	BA.1	BA.1_2	MBLG-CTMAPF48672990|EPI_ISL_8092920|Belgium|BA.1|2021-12-16	97,77
EPI_ISL_8093714	BA.1.17.2	BA.1.17.2	BA.1.17.2	NY-PRL-2021_1215_01F21|EPI_ISL_8093714|USA|BA.1.17.2|2021-12-13	99,52
EPI_ISL_8099176	BA.1.18	BA.1.15	BA.1.15	BE-ChVir-LB-211223-84638|EPI_ISL_8099176|Germany|BA.1.18|2021-12-17	95,10
EPI_ISL_8100674	BA.1	BA.1.15	BA.1.15	PLYM-2F7D4B2|EPI_ISL_8100674|United Kingdom|BA.1|2021-12-20	90,56
EPI_ISL_8102138	BA.1.15	BA.1.15	BA.1.15	MILK-2F9250F|EPI_ISL_8102138|United Kingdom|BA.1.15|2021-12-20	90,98

EPI_ISL_8102398	BA.1.1	BA.1.1	BA.1.1_2	MILK-2F9245D|EPI_ISL_8102398|United Kingdom|BA.1.1|2021-12-20	96,03
EPI_ISL_8104334	BA.1.17.2	BA.1.15	BA.1.15	LSPA-2F8DB6F|EPI_ISL_8104334|United Kingdom|BA.1.17.2|2021-12-16	90,98
EPI_ISL_8104575	BA.1	BA.1.15	BA.1.15	MILK-2F87BFC|EPI_ISL_8104575|United Kingdom|BA.1|2021-12-19	90,98
EPI_ISL_8107168	BA.1	BA.1.15	BA.1.15	MILK-2F702A6|EPI_ISL_8107168|United Kingdom|BA.1|2021-12-20	94,09
EPI_ISL_8107727	BA.1.17.2	BA.1.15	BA.1.15	ICH-741106928|EPI_ISL_8107727|Israel|BA.1.17.2|2021-12-21	77,68
EPI_ISL_8111125	BA.1.1	BA.1.15	BA.1.15	SMC-7027198|EPI_ISL_8111125|Israel|BA.1.1|2021-12-13	88,90
EPI_ISL_8124434	BA.1.15	BA.1	BA.1_4	TX-GD-122121-357|EPI_ISL_8124434|USA|BA.1.15|2021-12-17	84,18
EPI_ISL_8126589	BA.1.17	BA.1	BA.1_2	MD-CDC-MMB12124189|EPI_ISL_8126589|USA|BA.1.17|2021-12-13	98,28
EPI_ISL_8132777	BA.1.17	BA.1.15	BA.1.15	QLD3295|EPI_ISL_8132777|Australia|BA.1.17|2021-12-18	99,64
EPI_ISL_8141367	BA.1.1.13	BA.1.1	BA.1.1_2	MILK-2F982C6|EPI_ISL_8141367|United Kingdom|BA.1.1.13|2021-12-20	94,09
EPI_ISL_8150207	BA.1.15	BA.1.15	BA.1.15	MA-CDC-ASC210624409|EPI_ISL_8150207|USA|BA.1.15|2021-12-14	99,64
EPI_ISL_8156024	BA.1.1	BA.1.1	BA.1.1_2	DC-CDC-LC0430186|EPI_ISL_8156024|USA|BA.1.1|2021-12-15	99,64
EPI_ISL_8156392	BA.1	BA.1.15	BA.1.15	CT-CDC-LC0433109|EPI_ISL_8156392|USA|BA.1|2021-12-16	99,64
EPI_ISL_8157377	BA.1.1	BA.1.1	BA.1.1_2	CA-CDC-FG-202280|EPI_ISL_8157377|USA|BA.1.1|2021-12-19	97,18
EPI_ISL_8157598	BA.1	BA.1.15	BA.1.15	CA-CDC-FG-203011|EPI_ISL_8157598|USA|BA.1|2021-12-20	95,61
EPI_ISL_8159357	BA.1.15	BA.1.15	BA.1.15	CO-CDPHE-2102473518|EPI_ISL_8159357|USA|BA.1.15|2021-12-18	90,98
EPI_ISL_8160202	BA.1.20	BA.1	BA.1_1	MD-HP22549-PIDEKTXRYJ|EPI_ISL_8160202|USA|BA.1.20|2021-12-13	82,71
EPI_ISL_8168202	BA.1	BA.1	BA.1_3	ALDP-2F81CFF|EPI_ISL_8168202|United Kingdom|BA.1|2021-12-19	96,03
EPI_ISL_8172482	BA.1	BA.1.15	BA.1.15	DCGC-286851|EPI_ISL_8172482|Denmark|BA.1|2021-12-14	84,53
EPI_ISL_8172645	BA.1	BA.1.15	BA.1.15	DCGC-287336|EPI_ISL_8172645|Denmark|BA.1|2021-12-21	84,53
EPI_ISL_8178967	BA.1.1	BA.1.1	BA.1.1_3	NC-CDC-ASC210625877|EPI_ISL_8178967|USA|BA.1.1|2021-12-15	99,64
EPI_ISL_8179029	BA.1.1	BA.1.1	BA.1.1_2	FL-CDC-ASC210625057|EPI_ISL_8179029|USA|BA.1.1|2021-12-15	96,95
EPI_ISL_8182478	BA.1.15	BA.1	BA.1_4	MI-CDC-STM-28RJDRZPQ|EPI_ISL_8182478|USA|BA.1.15|2021-12-16	99,64
EPI_ISL_8186845	BA.1.1	BA.1.1	BA.1.1_2	MEX-INMEGEN-45-85|EPI_ISL_8186845|Mexico|BA.1.1|2021-12-18	99,08
EPI_ISL_8189914	BA.1.17.2	BA.1.17.2	BA.1.17.2	LOM-ASSTMonza-627953-20211221072200|EPI_ISL_8189914|Italy|BA.1.17.2|2021-12-21	95,75
EPI_ISL_8194536	BA.1	BA.1	BA.1_3	MILK-2FB7874|EPI_ISL_8194536|United Kingdom|BA.1|2021-12-20	96,03
EPI_ISL_8195959	BA.1.16	BA.1	BA.1_3	MILK-2FB36D8|EPI_ISL_8195959|United Kingdom|BA.1.16|2021-12-20	96,03
EPI_ISL_8197207	BA.1	BA.1.15	BA.1.15	QEUH-2F9FF5F|EPI_ISL_8197207|United Kingdom|BA.1|2021-12-21	92,25
EPI_ISL_8201818	BA.1.17.2	BA.1.17.2	BA.1.17.2	QEUH-2F6EF2C|EPI_ISL_8201818|United Kingdom|BA.1.17.2|2021-12-18	94,09
EPI_ISL_8206827	BA.1	BA.2	BA.2_1	ICH-741108638|EPI_ISL_8206827|Israel|BA.1|2021-12-22	92,20
EPI_ISL_8206828	BA.1	B.1.621.1	B.1.621.1	ICH-741108552|EPI_ISL_8206828|Israel|BA.1|2021-12-23	52,09
EPI_ISL_8207343	BA.1.1	BA.1	BA.1_4	NJ-PHEL-V21035149|EPI_ISL_8207343|USA|BA.1.1|2021-12-08	84,05
EPI_ISL_8210812	BA.1	BA.1	BA.1_1	CT-CDCBI-CRSP_XC4EK2QO3Z52YY3P|EPI_ISL_8210812|USA|BA.1|2021-12-20	93,93
EPI_ISL_8214484	BA.1.15	BA.1	BA.1_1	MA-CDCBI-CRSP_DAZAXGL624B4ARCP|EPI_ISL_8214484|USA|BA.1.15|2021-12-22	91,51
EPI_ISL_8215523	BA.1.17.2	BA.1.17.2	BA.1.17.2	MA-CDCBI-CRSP_V734RZL3U7MLUUDF|EPI_ISL_8215523|USA|BA.1.17.2|2021-12-17	92,29
EPI_ISL_8233630	BA.1.15	BA.1	BA.1_3	LSPA-2FC2B8C|EPI_ISL_8233630|United Kingdom|BA.1.15|2021-12-21	96,03
EPI_ISL_8238164	BA.1	BA.1.15	BA.1.15	LSPA-2FC6890|EPI_ISL_8238164|United Kingdom|BA.1|2021-12-20	93,53
EPI_ISL_8239225	BA.1	BA.1	BA.1_3	MILK-2FB9F40|EPI_ISL_8239225|United Kingdom|BA.1|2021-12-21	96,03
EPI_ISL_8243795	BA.1.17	BA.1	BA.1_3	PLYM-2FC13FE|EPI_ISL_8243795|United Kingdom|BA.1.17|2021-12-22	96,03
EPI_ISL_8243934	BA.1.17	BA.1	BA.1_3	PLYM-2FC4FCA|EPI_ISL_8243934|United Kingdom|BA.1.17|2021-12-22	96,03
EPI_ISL_8248830	BA.1.18	BA.1.15	BA.1.15	BW-RKI-I-410489|EPI_ISL_8248830|Germany|BA.1.18|2021-12-17	88,36
EPI_ISL_8249036	BA.1.1	BA.1.1	BA.1.1_2	HB-RKI-I-410725|EPI_ISL_8249036|Germany|BA.1.1|2021-12-15	99,64
EPI_ISL_8249445	BA.1.17.2	BA.1	BA.1_2	DCGC-288535|EPI_ISL_8249445|Denmark|BA.1.17.2|2021-12-24	99,68
EPI_ISL_8252026	BA.1	BA.1.15	BA.1.15	DCGC-287971|EPI_ISL_8252026|Denmark|BA.1|2021-12-20	87,41
EPI_ISL_8253078	BA.1.13	BA.1.15	BA.1.15	4058126464|EPI_ISL_8253078|Sweden|BA.1.13|2021-12-20	74,72
EPI_ISL_8254386	BA.1.18	BA.1.15	BA.1.15	AZDelta-2151-16420|EPI_ISL_8254386|Belgium|BA.1.18|2021-12-29	92,63
EPI_ISL_8262306	BA.1.1	BA.1	BA.1_4	OH-CDC-MMB12111758|EPI_ISL_8262306|USA|BA.1.1|2021-12-16	93,44
EPI_ISL_8268702	BA.1	BA.1	BA.1_3	NORT-YBN5WZ|EPI_ISL_8268702|United Kingdom|BA.1|2021-12-18	94,56
EPI_ISL_8270956	BA.1.15	BA.1.15	BA.1.15	MILK-3006A03|EPI_ISL_8270956|United Kingdom|BA.1.15|2021-12-27	94,09
EPI_ISL_8271001	BA.1	BA.1.15	BA.1.15	MILK-3006DEC|EPI_ISL_8271001|United Kingdom|BA.1|2021-12-28	94,09
EPI_ISL_8271999	BA.1.17.2	BA.1.17.2	BA.1.17.2	HSLL-2FFE871|EPI_ISL_8271999|United Kingdom|BA.1.17.2|2021-12-25	96,03
EPI_ISL_8272175	BA.1.17.2	BA.1.17.2	BA.1.17.2	LSPA-3000CA9|EPI_ISL_8272175|United Kingdom|BA.1.17.2|2021-12-22	94,09
EPI_ISL_8272692	BA.1.17.2	BA.1.17.2	BA.1.17.2	MILK-3002E1A|EPI_ISL_8272692|United Kingdom|BA.1.17.2|2021-12-28	94,09
EPI_ISL_8273660	BA.1.17.2	BA.1.17.2	BA.1.17.2	MILK-3002199|EPI_ISL_8273660|United Kingdom|BA.1.17.2|2021-12-22	96,01
EPI_ISL_8279452	BA.1	BA.1.15	BA.1.15	QEUH-2FE0739|EPI_ISL_8279452|United Kingdom|BA.1|2021-12-21	90,98
EPI_ISL_8281280	BA.1	BA.1.15	BA.1.15	MILK-2FA431F|EPI_ISL_8281280|United Kingdom|BA.1|2021-12-20	90,19
EPI_ISL_8281282	BA.1.16	BA.1	BA.1_3	MILK-2FA4458|EPI_ISL_8281282|United Kingdom|BA.1.16|2021-12-20	93,99
EPI_ISL_8281410	BA.1.1	BA.1.1	BA.1.1_2	CA-CDC-FG-206221|EPI_ISL_8281410|USA|BA.1.1|2021-12-20	99,52
EPI_ISL_8281956	BA.1.1.8	BA.1.1	BA.1.1_2	CA-CDC-FG-207359|EPI_ISL_8281956|USA|BA.1.1.8|2021-12-21	99,52
EPI_ISL_8282473	BA.1.1	BA.1.1	BA.1.1_2	CA-CDC-FG-207627|EPI_ISL_8282473|USA|BA.1.1|2021-12-21	98,59
EPI_ISL_8282503	BA.1.1	BA.1.1	BA.1.1_2	CA-CDC-FG-207781|EPI_ISL_8282503|USA|BA.1.1|2021-12-21	98,74
EPI_ISL_8282633	BA.1	BA.1	BA.1_2	CA-CDC-FG-206777|EPI_ISL_8282633|USA|BA.1|2021-12-21	99,52
EPI_ISL_8284789	BA.1.15	BA.1.15	BA.1.15	CA-CDC-FG-210454|EPI_ISL_8284789|USA|BA.1.15|2021-12-22	90,54
EPI_ISL_8290793	BA.1	BA.1	BA.1_4	CA-CDC-FG-214978|EPI_ISL_8290793|USA|BA.1|2021-12-21	99,64
EPI_ISL_8291470	BA.1.20	BA.1.15	BA.1.15	CA-CDC-FG-213259|EPI_ISL_8291470|USA|BA.1.20|2021-12-21	99,50
EPI_ISL_8291922	BA.1.15	BA.1	BA.1_4	TX-CDC-FG-214822|EPI_ISL_8291922|USA|BA.1.15|2021-12-22	99,64
EPI_ISL_8292522	BA.1.15	BA.1.15	BA.1.15	CA-CDC-FG-208438|EPI_ISL_8292522|USA|BA.1.15|2021-12-22	95,61
EPI_ISL_8292812	BA.1.1	BA.1.1	BA.1.1_2	CA-CDC-FG-211431|EPI_ISL_8292812|USA|BA.1.1|2021-12-22	98,74
EPI_ISL_8293793	BA.1.1	BA.1.1	BA.1.1_2	CA-CDC-FG-211658|EPI_ISL_8293793|USA|BA.1.1|2021-12-22	93,13
EPI_ISL_8294954	BA.1.1	BA.1.1	BA.1.1_3	CA-CCPHL-1190|EPI_ISL_8294954|USA|BA.1.1|2021-12-16	90,23
EPI_ISL_8295539	BA.1	BA.1	BA.1_3	AZ-ASU29927|EPI_ISL_8295539|USA|BA.1|2021-12-21	95,23
EPI_ISL_8299948	BA.1.15	BA.1	BA.1_3	MILK-3022C47|EPI_ISL_8299948|United Kingdom|BA.1.15|2021-12-28	96,03
EPI_ISL_8300732	BA.1	BA.1	BA.1_3	MILK-302147D|EPI_ISL_8300732|United Kingdom|BA.1|2021-12-28	96,03
EPI_ISL_8301217	BA.1.1.12	BA.1.1	BA.1.1_2	LSPA-3025F3C|EPI_ISL_8301217|United Kingdom|BA.1.1.12|2021-12-22	90,98
EPI_ISL_8302918	BA.1	BA.1	BA.1_3	MILK-300FF74|EPI_ISL_8302918|United Kingdom|BA.1|2021-12-27	96,03
EPI_ISL_8303478	BA.1	BA.1	BA.1_3	MILK-3014A11|EPI_ISL_8303478|United Kingdom|BA.1|2021-12-27	96,03
EPI_ISL_8306947	BA.1.17	BA.1	BA.1_4	MD-HGUGM-5974045|EPI_ISL_8306947|Spain|BA.1.17|2021-12-22	96,72
EPI_ISL_8310902	BA.1.1.14	BA.1.1	BA.1.1_2	VT-CDC-ASC210632272|EPI_ISL_8310902|USA|BA.1.1.14|2021-12-18	98,70
EPI_ISL_8311892	BA.1.1	BA.1.1	BA.1.1_2	NC-CDC-ASC210636384|EPI_ISL_8311892|USA|BA.1.1|2021-12-19	99,64
EPI_ISL_8312346	BA.1	BA.1.15	BA.1.15	1925244164|EPI_ISL_8312346|Sweden|BA.1|2021-12-20	92,92
EPI_ISL_8312961	BA.1	BA.1	BA.1_2	LNS4126077|EPI_ISL_8312961|Luxembourg|BA.1|2021-12-21	90,71
EPI_ISL_8315311	BA.1.20	BA.1.15	BA.1.15	WI-UW-7953|EPI_ISL_8315311|USA|BA.1.20|2021-12-24	99,64
EPI_ISL_8324784	BA.1.1	BA.1.1	BA.1.1_3	TX-DSHS-13001|EPI_ISL_8324784|USA|BA.1.1|2021-12-17	98,74
EPI_ISL_8331841	BA.1.17	BA.1	BA.1_1	ACT-2498|EPI_ISL_8331841|Australia|BA.1.17|2021-12-30	89,58
EPI_ISL_8334191	BA.1.17	BA.1.15	BA.1.15	NSW-ICPMR-18740|EPI_ISL_8334191|Australia|BA.1.17|2021-12-22	99,64
EPI_ISL_8337172	BA.1.1	BA.1.1	BA.1.1_2	CO-CDPHE-2102485112|EPI_ISL_8337172|USA|BA.1.1|2021-12-21	99,64
EPI_ISL_8340849	BA.1.1.14	BA.1.1	BA.1.1_2	MILK-302E06D|EPI_ISL_8340849|United Kingdom|BA.1.1.14|2021-12-28	96,03
EPI_ISL_8342109	BA.1.17.2	BA.1.17.2	BA.1.17.2	ALDP-302EAF6|EPI_ISL_8342109|United Kingdom|BA.1.17.2|2021-12-27	96,03
EPI_ISL_8347011	BA.1	BA.1	BA.1_1	DCGC-293462|EPI_ISL_8347011|Denmark|BA.1|2021-12-18	99,37
EPI_ISL_8347524	BA.1.17.2	BA.1.17.2	BA.1.17.2	DCGC-294039|EPI_ISL_8347524|Denmark|BA.1.17.2|2021-12-27	98,26
EPI_ISL_8347652	BA.1.18	BA.1	BA.1_4	OCC-CHU-TLS-213561338201|EPI_ISL_8347652|France|BA.1.18|2021-12-22	99,64
EPI_ISL_8354827	BA.1	BA.1	BA.1_4	WA-CDC-UW21122045566|EPI_ISL_8354827|USA|BA.1|2021-12-20	91,38
EPI_ISL_8371050	BA.1.17.2	BA.1.17.2	BA.1.17.2	BC-BCCDC-276368|EPI_ISL_8371050|Canada|BA.1.17.2|2021-12-08	99,64
EPI_ISL_8375300	BA.1.1	BA.1.1	BA.1.1_3	NIC_NBI_SEQ23508|EPI_ISL_8375300|Thailand|BA.1.1|2021-12-20	92,83
EPI_ISL_8378588	BA.1.1.11	BA.1.1	BA.1.1_3	ZH-UZH-IMV-3ba59439|EPI_ISL_8378588|Switzerland|BA.1.1.11|2021-12-19	99,62
EPI_ISL_8379043	BA.1.1.18	BA.1.1	BA.1.1_2	CO-CDPHE-2102485358|EPI_ISL_8379043|USA|BA.1.1.18|2021-12-22	96,03
EPI_ISL_8380854	BA.1	BA.1	BA.1_3	PHEC-YYBSGDG|EPI_ISL_8380854|United Kingdom|BA.1|2021-12-20	94,14
EPI_ISL_8382972	BA.1.17.2	A	A_1	DCGC-294909|EPI_ISL_8382972|Denmark|BA.1.17.2|2021-12-28	82,20
EPI_ISL_8385097	BA.1.17.2	BA.1.15	BA.1.15	MILK-303DF5F|EPI_ISL_8385097|United Kingdom|BA.1.17.2|2021-12-28	90,98
EPI_ISL_8387389	BA.1	BA.1.15	BA.1.15	MILK-3015DEA|EPI_ISL_8387389|United Kingdom|BA.1|2021-12-27	94,09
EPI_ISL_8391994	BA.1.15	BA.1.15	BA.1.15	CA-CDC-FG-219874|EPI_ISL_8391994|USA|BA.1.15|2021-12-23	95,40

EPI_ISL_8393515	BA.1	BA.1	BA.1_3	CA-CDC-FG-218387|EPI_ISL_8393515|USA|BA.1|2021-12-23	93,59
EPI_ISL_8393703	BA.1	BA.1.15	BA.1.15	CA-CDC-FG-220469|EPI_ISL_8393703|USA|BA.1|2021-12-24	76,47
EPI_ISL_8394485	BA.1.1	BA.1.1	BA.1.1_2	CA-CDC-ASC210632737|EPI_ISL_8394485|USA|BA.1.1|2021-12-21	99,18
EPI_ISL_8411475	BA.1	BA.1	BA.1_1	BW-RKI-I-412736|EPI_ISL_8411475|Germany|BA.1|2021-12-26	91,24
EPI_ISL_8414821	BA.1	BA.1	BA.1_3	IDF-CERBAHC-1214718|EPI_ISL_8414821|France|BA.1|2021-12-13	96,70
EPI_ISL_8416092	BA.1.1.15	BA.1.1	BA.1.1_3	PHEP-YYN5IXW|EPI_ISL_8416092|United Kingdom|BA.1.1.15|2021-12-21	84,47
EPI_ISL_8416217	BA.1.18	BA.1.15	BA.1.15	IDF-HMN-21122300610|EPI_ISL_8416217|France|BA.1.18|2021-12-30	88,88
EPI_ISL_8420735	BA.1.15	BA.1.15	BA.1.15	DCGC-296286|EPI_ISL_8420735|Denmark|BA.1.15|2021-12-22	95,06
EPI_ISL_8423072	BA.1.17	BA.1	BA.1_4	ZH-EMC-4533|EPI_ISL_8423072|Netherlands|BA.1.17|2021-12-30	95,97
EPI_ISL_8425086	BA.1.1	BA.1.1	BA.1.1_2	FL-CDC-ASC210640476|EPI_ISL_8425086|USA|BA.1.1|2021-12-21	98,15
EPI_ISL_8425192	BA.1.15	BA.1.15	BA.1.15	TN-CDC-ASC210638986|EPI_ISL_8425192|USA|BA.1.15|2021-12-21	99,64
EPI_ISL_8426014	BA.1.15	BA.1	BA.1_4	CA-CDC-ASC210638392|EPI_ISL_8426014|USA|BA.1.15|2021-12-22	99,64
EPI_ISL_8427206	BA.1.18	BA.1.15	BA.1.15	JU-Risch-21C2714225|EPI_ISL_8427206|Switzerland|BA.1.18|2021-12-23	99,64
EPI_ISL_8427557	BA.1.1	BA.1.1	BA.1.1_3	PA-CDC-STM-USXD3Q3MB|EPI_ISL_8427557|USA|BA.1.1|2021-12-20	99,64
EPI_ISL_8428537	BA.1.20	BA.1.15	BA.1.15	IA-SHL-1987829|EPI_ISL_8428537|USA|BA.1.20|2021-12-29	84,05
EPI_ISL_8429099	BA.1.1	BA.1	BA.1_4	OR-OHSU-213640476|EPI_ISL_8429099|USA|BA.1.1|2021-12-25	91,39
EPI_ISL_8431835	BA.1.17.2	BA.1.17.2	BA.1.17.2	MA-CDCBI-CRSP_765OSIMOJFGRGKH6|EPI_ISL_8431835|USA|BA.1.17.2|2021-12-27	95,13
EPI_ISL_8432062	BA.1.1	BA.1.1	BA.1.1_2	MA-CDCBI-CRSP_A3HSOPF6SL2RHXV6|EPI_ISL_8432062|USA|BA.1.1|2021-12-26	95,13
EPI_ISL_8433075	BA.1.1	BA.1.1	BA.1.1_2	MA-CDCBI-CRSP_E4HGMA3GXUGR3EBS|EPI_ISL_8433075|USA|BA.1.1|2021-12-27	94,52
EPI_ISL_8434789	BA.1.15	BA.1	BA.1_1	VT-CDCBI-CRSP_XUNX54ZIQTBEIKXD|EPI_ISL_8434789|USA|BA.1.15|2021-12-26	94,54
EPI_ISL_8436015	BA.1.1	BA.1.1	BA.1.1_2	MEX-INMEGEN-47-51|EPI_ISL_8436015|Mexico|BA.1.1|2021-12-26	98,87
EPI_ISL_8437411	BA.1	BA.1.15	BA.1.15	DMSc-05997|EPI_ISL_8437411|Thailand|BA.1|2021-12-21	83,92
EPI_ISL_8440160	BA.1.17.2	BA.1.15	BA.1.15	MILK-3055ABA|EPI_ISL_8440160|United Kingdom|BA.1.17.2|2021-12-29	90,98
EPI_ISL_8440833	BA.1.17.2	BA.1.17.2	BA.1.17.2	QEUH-30892C4|EPI_ISL_8440833|United Kingdom|BA.1.17.2|2021-12-27	94,09
EPI_ISL_8441468	BA.1.15.1	BA.1.15	BA.1.15	ALDP-309251A|EPI_ISL_8441468|United Kingdom|BA.1.15.1|2021-12-30	95,40
EPI_ISL_8441719	BA.1.1.15	BA.1.1	BA.1.1_2	MILK-307E15C|EPI_ISL_8441719|United Kingdom|BA.1.1.15|2021-12-29	96,03
EPI_ISL_8441745	BA.1.17.2	BA.1.17.2	BA.1.17.2	MILK-307BE3A|EPI_ISL_8441745|United Kingdom|BA.1.17.2|2021-12-30	96,03
EPI_ISL_8442002	BA.1.15.1	BA.1.15	BA.1.15	QEUH-3079A74|EPI_ISL_8442002|United Kingdom|BA.1.15.1|2021-12-30	96,01
EPI_ISL_8442649	BA.1.17.2	BA.1.17.2	BA.1.17.2	QEUH-3074C37|EPI_ISL_8442649|United Kingdom|BA.1.17.2|2021-12-30	94,09
EPI_ISL_8442984	BA.1.17.2	BA.1.17.2	BA.1.17.2	LSPA-3069664|EPI_ISL_8442984|United Kingdom|BA.1.17.2|2021-12-27	94,09
EPI_ISL_8443154	BA.1.17.2	BA.1.17.2	BA.1.17.2	LSPA-306BE3D|EPI_ISL_8443154|United Kingdom|BA.1.17.2|2021-12-28	96,03
EPI_ISL_8443264	BA.1.15.1	BA.1.15	BA.1.15	LSPA-306BED3|EPI_ISL_8443264|United Kingdom|BA.1.15.1|2021-12-27	96,03
EPI_ISL_8446317	BA.1.1	BA.1.1	BA.1.1_2	ALDP-3089877|EPI_ISL_8446317|United Kingdom|BA.1.1|2021-12-30	94,09
EPI_ISL_8447104	BA.1	BA.1	BA.1_3	QEUH-30725F2|EPI_ISL_8447104|United Kingdom|BA.1|2021-12-29	95,92
EPI_ISL_8447475	BA.1	BA.1.15	BA.1.15	QEUH-3072167|EPI_ISL_8447475|United Kingdom|BA.1|2021-12-27	90,98
EPI_ISL_8449780	BA.1.1	BA.1.1	BA.1.1_2	MILK-3082058|EPI_ISL_8449780|United Kingdom|BA.1.1|2021-12-30	94,52
EPI_ISL_8449791	BA.1.17.2	BA.1.15	BA.1.15	MILK-3081D9E|EPI_ISL_8449791|United Kingdom|BA.1.17.2|2021-12-29	90,63
EPI_ISL_8449910	BA.1.1	BA.1.1	BA.1.1_3	53|EPI_ISL_8449910|Singapore|BA.1.1|2022-01-02	99,64
EPI_ISL_8450117	BA.1.17.2	BA.1.17.2	BA.1.17.2	LSPA-3065AD5|EPI_ISL_8450117|United Kingdom|BA.1.17.2|2021-12-27	94,09
EPI_ISL_8450739	BA.1.1	BA.1.1	BA.1.1_2	BRBR-304665E|EPI_ISL_8450739|United Kingdom|BA.1.1|2021-12-29	90,98
EPI_ISL_8451022	BA.1.17.2	BA.1.15	BA.1.15	BRBR-304D0E0|EPI_ISL_8451022|United Kingdom|BA.1.17.2|2021-12-28	90,98
EPI_ISL_8451344	BA.1	BA.1.15	BA.1.15	PHEP-YYN7EW1|EPI_ISL_8451344|United Kingdom|BA.1|2021-12-17	84,47
EPI_ISL_8452054	BA.1.17.2	BA.1.17.2	BA.1.17.2	ALDP-30634E8|EPI_ISL_8452054|United Kingdom|BA.1.17.2|2021-12-29	96,03
EPI_ISL_8453599	BA.1.15	BA.1.15	BA.1.15	MILK-305EFEF|EPI_ISL_8453599|United Kingdom|BA.1.15|2021-12-29	93,57
EPI_ISL_8453617	BA.1.15	BA.1.15	BA.1.15	LSPA-3060982|EPI_ISL_8453617|United Kingdom|BA.1.15|2021-12-28	94,09
EPI_ISL_8454548	BA.1.15	BA.1.15	BA.1.15	QEUH-305E32F|EPI_ISL_8454548|United Kingdom|BA.1.15|2021-12-29	96,03
EPI_ISL_8455191	BA.1	BA.1.15	BA.1.15	BRBR-303FFE4|EPI_ISL_8455191|United Kingdom|BA.1|2021-12-28	90,98
EPI_ISL_8455896	BA.1.17.2	BA.1.17.2	BA.1.17.2	MILK-303D46F|EPI_ISL_8455896|United Kingdom|BA.1.17.2|2021-12-28	95,94
EPI_ISL_8456587	BA.1.15.1	BA.1.15	BA.1.15	QEUH-3029EB3|EPI_ISL_8456587|United Kingdom|BA.1.15.1|2021-12-28	96,03
EPI_ISL_8457912	BA.1	BA.1.15	BA.1.15	DCGC-297296|EPI_ISL_8457912|Denmark|BA.1|2021-12-30	87,08
EPI_ISL_8458062	BA.1.17.2	BA.1.15	BA.1.15	DCGC-297410|EPI_ISL_8458062|Denmark|BA.1.17.2|2021-12-31	90,99
EPI_ISL_8464381	BA.1.17.2	BA.1.17.2	BA.1.17.2	PHWC-PGMO4F|EPI_ISL_8464381|United Kingdom|BA.1.17.2|2021-12-16	99,64
EPI_ISL_8464551	BA.1.17.2	BA.1.17.2	BA.1.17.2	PHWC-PGMUH6|EPI_ISL_8464551|United Kingdom|BA.1.17.2|2021-12-16	96,03
EPI_ISL_8464666	BA.1.18	BA.1	BA.1_4	OCC-CHU-TLS-213631385101|EPI_ISL_8464666|France|BA.1.18|2021-12-27	79,62
EPI_ISL_8465498	BA.2	BA.2	BA.2_1	DCGC-298892|EPI_ISL_8465498|Denmark|BA.2|2022-01-02	99,81
EPI_ISL_8465788	BA.1	BA.1.15	BA.1.15	DCGC-299002|EPI_ISL_8465788|Denmark|BA.1|2022-01-02	91,47
EPI_ISL_8466254	BA.1.1.1	BA.1.1	BA.1.1_2	GE-HUG-36578554|EPI_ISL_8466254|Switzerland|BA.1.1.1|2021-12-29	99,64
EPI_ISL_8466537	BA.1.1	BA.1.1	BA.1.1_2	CA-CDC-FG-223545|EPI_ISL_8466537|USA|BA.1.1|2021-12-23	95,61
EPI_ISL_8466836	BA.1.1	BA.1	BA.1_3	PE-FIOCRUZ-IAM6311|EPI_ISL_8466836|Brazil|BA.1.1|2021-12-26	88,49
EPI_ISL_8467535	BA.1.17.2	BA.1.15	BA.1.15	DCGC-297755|EPI_ISL_8467535|Denmark|BA.1.17.2|2021-12-29	84,47
EPI_ISL_8468012	BA.1.17	BA.1.15	BA.1.15	DCGC-297651|EPI_ISL_8468012|Denmark|BA.1.17|2022-01-02	89,70
EPI_ISL_8468144	BA.1.17	BA.1.15	BA.1.15	DCGC-298949|EPI_ISL_8468144|Denmark|BA.1.17|2022-01-02	84,58
EPI_ISL_8470675	BA.1.1	BA.1.1	BA.1.1_2	CA-CDC-LC0446187|EPI_ISL_8470675|USA|BA.1.1|2021-12-28	99,64
EPI_ISL_8472233	BA.1.1	BA.1.1	BA.1.1_2	OH-CDC-ASC210568208|EPI_ISL_8472233|USA|BA.1.1|2021-12-23	99,64
EPI_ISL_8473062	BA.1.1.11	BA.1.1	BA.1.1_2	SG-CLM-12296394|EPI_ISL_8473062|Switzerland|BA.1.1.11|2021-12-29	96,89
EPI_ISL_8473171	BA.1.17	BA.1.15	BA.1.15	SG-CLM-12314258|EPI_ISL_8473171|Switzerland|BA.1.17|2021-12-31	89,20
EPI_ISL_8473577	BA.1	BA.1	BA.1_2	CA-CDC-FG-205529|EPI_ISL_8473577|USA|BA.1|2021-12-20	99,52
EPI_ISL_8473765	BA.1.1	BA.1	BA.1_1	MD-HP23050-PIDSZLZZMM|EPI_ISL_8473765|USA|BA.1.1|2021-12-27	82,71
EPI_ISL_8474570	BA.1.15	BA.1	BA.1_2	TX-CDC-FG-204080|EPI_ISL_8474570|USA|BA.1.15|2021-12-20	99,52
EPI_ISL_8475363	BA.1.15	BA.1	BA.1_3	TX-CDC-FG-204225|EPI_ISL_8475363|USA|BA.1.15|2021-12-21	95,21
EPI_ISL_8481071	BA.1.1	BA.1.1	BA.1.1_2	GA-GPHL-2701|EPI_ISL_8481071|USA|BA.1.1|2021-12-21	95,67
EPI_ISL_8484338	BA.1.1.18	BA.1	BA.1_1	MA-CDCBI-CRSP_ODKGXY6GARDRZDFT|EPI_ISL_8484338|USA|BA.1.1.18|2021-12-29	94,54
EPI_ISL_8487355	BA.1	BA.1	BA.1_1	CT-JAX-JAX-2201.01-126|EPI_ISL_8487355|USA|BA.1|2021-12-29	91,97
EPI_ISL_8488042	BA.1.15.1	BA.1.15.1	BA.1.15.1	PHEC-YYBS6RC|EPI_ISL_8488042|United Kingdom|BA.1.15.1|2021-12-26	99,58
EPI_ISL_8488540	BA.1.17.2	BA.1.15	BA.1.15	PHEP-YYN6DAP|EPI_ISL_8488540|United Kingdom|BA.1.17.2|2021-12-15	84,47
EPI_ISL_8488698	BA.1.1	BA.1.1	BA.1.1_3	PHEP-YYN6Q7A|EPI_ISL_8488698|United Kingdom|BA.1.1|2021-12-20	84,47
EPI_ISL_8489116	BA.1	BA.1	BA.1_3	QEUH-30BFFDD|EPI_ISL_8489116|United Kingdom|BA.1|2022-01-02	96,03
EPI_ISL_8489291	BA.1.1	BA.1.1	BA.1.1_2	MILK-30BF517|EPI_ISL_8489291|United Kingdom|BA.1.1|2021-12-31	96,03
EPI_ISL_8490271	BA.1.15.1	BA.1.15	BA.1.15	PLYM-30AD4E2|EPI_ISL_8490271|United Kingdom|BA.1.15.1|2022-01-01	90,98
EPI_ISL_8493585	BA.1	BA.1.15	BA.1.15	LSPA-30B47B2|EPI_ISL_8493585|United Kingdom|BA.1|2021-12-29	94,10
EPI_ISL_8494562	BA.1.17.2	BA.1.17.2	BA.1.17.2	QEUH-30962B5|EPI_ISL_8494562|United Kingdom|BA.1.17.2|2021-12-29	94,09
EPI_ISL_8496406	BA.1.15	BA.1	BA.1_3	MILK-30955F9|EPI_ISL_8496406|United Kingdom|BA.1.15|2022-01-01	96,03
EPI_ISL_8497672	BA.1.17.2	BA.1.17.2	BA.1.17.2	MILK-30A3D4B|EPI_ISL_8497672|United Kingdom|BA.1.17.2|2022-01-01	94,09
EPI_ISL_8497823	BA.1	BA.1	BA.1_3	MILK-3099FAC|EPI_ISL_8497823|United Kingdom|BA.1|2021-12-31	96,03
EPI_ISL_8497862	BA.1.17.2	BA.1.17.2	BA.1.17.2	LSPA-30A4C89|EPI_ISL_8497862|United Kingdom|BA.1.17.2|2021-12-29	96,03
EPI_ISL_8498222	BA.1.17.2	BA.1.17.2	BA.1.17.2	QEUH-30AB4C6|EPI_ISL_8498222|United Kingdom|BA.1.17.2|2021-12-29	96,03
EPI_ISL_8499324	BA.1.15.1	BA.1.15	BA.1.15	LSPA-3099838|EPI_ISL_8499324|United Kingdom|BA.1.15.1|2021-12-29	94,09
EPI_ISL_8499572	BA.1	BA.1.15	BA.1.15	ALDP-3092486|EPI_ISL_8499572|United Kingdom|BA.1|2021-12-31	94,09
EPI_ISL_8500548	BA.1.1	BA.1.1	BA.1.1_2	QEUH-3091E7A|EPI_ISL_8500548|United Kingdom|BA.1.1|2021-12-30	96,03
EPI_ISL_8505315	BA.1.1	BA.1.1	BA.1.1_2	NJ-CDC-IBX737684673786|EPI_ISL_8505315|USA|BA.1.1|2021-12-22	96,64
EPI_ISL_8509127	BA.1.1	BA.1.1	BA.1.1_2	AZ-ASU31544|EPI_ISL_8509127|USA|BA.1.1|2022-01-03	96,03
EPI_ISL_8511658	BA.1.1	BA.1	BA.1_1	MA-CDCBI-CRSP_2DJCYYD6VO3DKU7I|EPI_ISL_8511658|USA|BA.1.1|2022-01-02	95,12
EPI_ISL_8511675	BA.1	BA.1	BA.1_1	MA-CDCBI-CRSP_55ZDYONCWSEHHUTI|EPI_ISL_8511675|USA|BA.1|2022-01-02	93,93
EPI_ISL_8511921	BA.1	BA.1	BA.1_1	MA-CDCBI-CRSP_6W22RJVQTZIJX7MI|EPI_ISL_8511921|USA|BA.1|2021-12-30	89,35
EPI_ISL_8513378	BA.1.17.2	BA.1.17.2	BA.1.17.2	MA-CDCBI-CRSP_RFTYALGZ7RFNFSDG|EPI_ISL_8513378|USA|BA.1.17.2|2021-12-31	93,93
EPI_ISL_8519512	BA.1.1	BA.1.15	BA.1.15	ICH-741110982|EPI_ISL_8519512|Israel|BA.1.1|2022-01-02	65,66
EPI_ISL_8520724	BA.1	BA.1.15	BA.1.15	MILK-30CF927|EPI_ISL_8520724|United Kingdom|BA.1|2021-12-31	92,75
EPI_ISL_8522485	BA.1	BA.1.15	BA.1.15	LSPA-30C5790|EPI_ISL_8522485|United Kingdom|BA.1|2021-12-30	90,96
EPI_ISL_8522839	BA.1.1	BA.1.1	BA.1.1_2	QEUH-30CC711|EPI_ISL_8522839|United Kingdom|BA.1.1|2021-12-31	96,03
EPI_ISL_8522900	BA.1.17.2	BA.1.15	BA.1.15	LSPA-30CC1AA|EPI_ISL_8522900|United Kingdom|BA.1.17.2|2021-12-29	90,98
EPI_ISL_8523208	BA.1.17.2	BA.1.17.2	BA.1.17.2	QEUH-30CB4FD|EPI_ISL_8523208|United Kingdom|BA.1.17.2|2021-12-31	96,03

EPI_ISL_8523303	BA.1.15.1	BA.1.15	BA.1.15	MILK-30C6DCA|EPI_ISL_8523303|United Kingdom|BA.1.15.1|2022-01-02	94,09
EPI_ISL_8524502	BA.1.1	BA.1.15	BA.1.15	HSGM-F13542|EPI_ISL_8524502|Turkey|BA.1.1|2021-12-30	99,69
EPI_ISL_8530149	BA.1	BA.1.15	BA.1.15	DCGC-301606|EPI_ISL_8530149|Denmark|BA.1|2021-12-28	83,88
EPI_ISL_8530273	BA.1.21	BA.1.15	BA.1.15	DCGC-300157|EPI_ISL_8530273|Denmark|BA.1.21|2022-01-03	88,90
EPI_ISL_8531273	BA.1.1	BA.1.1	BA.1.1_2	QEUH-31001C7|EPI_ISL_8531273|United Kingdom|BA.1.1|2022-01-02	93,02
EPI_ISL_8532025	BA.1.17.2	BA.1.15	BA.1.15	QEUH-3103D75|EPI_ISL_8532025|United Kingdom|BA.1.17.2|2021-12-31	90,99
EPI_ISL_8532434	BA.1.1.1	BA.1.1	BA.1.1_2	LI-MUMC-4780|EPI_ISL_8532434|Netherlands|BA.1.1.1|2021-12-29	99,64
EPI_ISL_8532795	BA.1.1.15	BA.1.1	BA.1.1_2	MILK-310EDB6|EPI_ISL_8532795|United Kingdom|BA.1.1.15|2022-01-03	93,36
EPI_ISL_8535355	BA.1.17.2	BA.1.17.2	BA.1.17.2	MILK-30EE56A|EPI_ISL_8535355|United Kingdom|BA.1.17.2|2022-01-03	96,03
EPI_ISL_8535574	BA.1.15.1	BA.1.15	BA.1.15	ALDP-30F32F2|EPI_ISL_8535574|United Kingdom|BA.1.15.1|2022-01-02	90,98
EPI_ISL_8538311	BA.1	BA.1	BA.1_3	QEUH-30D34F2|EPI_ISL_8538311|United Kingdom|BA.1|2021-12-29	96,03
EPI_ISL_8538783	BA.1.17	BA.1.15	BA.1.15	SUS0007598|EPI_ISL_8538783|Sweden|BA.1.17|2021-12-26	92,22
EPI_ISL_8541456	BA.1.17.2	BA.1.15	BA.1.15	CSQ4529|EPI_ISL_8541456|Czech Republic|BA.1.17.2|2021-12-31	94,09
EPI_ISL_8543467	BA.1.20	BA.1.15	BA.1.15	WI-UW-8324|EPI_ISL_8543467|USA|BA.1.20|2021-12-24	99,64
EPI_ISL_8543568	BA.1.15	BA.1.15	BA.1.15	WI-UW-8261|EPI_ISL_8543568|USA|BA.1.15|2021-12-30	99,64
EPI_ISL_8544066	BA.1.1	BA.1.1	BA.1.1_2	NY-PRL-2021_1223_07I05|EPI_ISL_8544066|USA|BA.1.1|2021-12-19	68,54
EPI_ISL_8555062	BA.1.15	BA.1.15	BA.1.15	TN-CDC-ASC210568910|EPI_ISL_8555062|USA|BA.1.15|2021-12-27	99,64
EPI_ISL_8556578	BA.1.1	BA.1.1	BA.1.1_2	NJ-CDC-IBX051529883171|EPI_ISL_8556578|USA|BA.1.1|2021-12-21	96,64
EPI_ISL_8559311	BA.1	BA.1	BA.1_3	QIBZIM-71341|EPI_ISL_8559311|Zimbabwe|BA.1|2021-12-01	95,99
EPI_ISL_8559725	BA.1.1	BA.1.1	BA.1.1_3	IC-2630|EPI_ISL_8559725|Japan|BA.1.1|2021-12-27	99,64
EPI_ISL_8561967	BA.1.15	BA.1.15	BA.1.15	FL-BPHL-18767|EPI_ISL_8561967|USA|BA.1.15|2021-12-13	99,64
EPI_ISL_8563106	BA.1	BA.1	BA.1_4	CT-Yale-15327|EPI_ISL_8563106|USA|BA.1|2021-12-21	92,58
EPI_ISL_8563123	BA.1.1	BA.1.1	BA.1.1_2	CT-Yale-15344|EPI_ISL_8563123|USA|BA.1.1|2021-12-23	95,46
EPI_ISL_8564076	BA.1.1.18	BA.1.15	BA.1.15	CO-CDPHE-2102513267|EPI_ISL_8564076|USA|BA.1.1.18|2021-12-16	85,00
EPI_ISL_8564307	BA.1.1	BA.1.1	BA.1.1_2	WA-PHL-009212|EPI_ISL_8564307|USA|BA.1.1|2021-12-27	99,48
EPI_ISL_8565045	BA.2	A	A_14	DL-ILBS-WGS1140|EPI_ISL_8565045|India|BA.2|2021-12-25	99,81
EPI_ISL_8566705	BA.1.1	BA.1.1	BA.1.1_2	WA-PHL-008933|EPI_ISL_8566705|USA|BA.1.1|2021-12-20	94,26
EPI_ISL_8569659	BA.1.17.2	BA.1.15	BA.1.15	MILK-3106A4E|EPI_ISL_8569659|United Kingdom|BA.1.17.2|2022-01-03	90,96
EPI_ISL_8570093	BA.1.17.2	BA.1.17.2	BA.1.17.2	ALDP-3126F94|EPI_ISL_8570093|United Kingdom|BA.1.17.2|2022-01-02	96,01
EPI_ISL_8571509	BA.1.1	BA.1.1	BA.1.1_2	BRBR-311727F|EPI_ISL_8571509|United Kingdom|BA.1.1|2022-01-03	90,98
EPI_ISL_8572474	BA.1.17.2	BA.1.15	BA.1.15	PLYM-310CE79|EPI_ISL_8572474|United Kingdom|BA.1.17.2|2022-01-03	90,98
EPI_ISL_8572547	BA.1.17.2	BA.1.17.2	BA.1.17.2	ALDP-3111C86|EPI_ISL_8572547|United Kingdom|BA.1.17.2|2022-01-03	96,03
EPI_ISL_8573778	BA.1.17.2	BA.1.15	BA.1.15	PHEP-YYN98RO|EPI_ISL_8573778|United Kingdom|BA.1.17.2|2021-12-17	89,26
EPI_ISL_8575314	BA.1	BA.1	BA.1_3	MILK-310F2D4|EPI_ISL_8575314|United Kingdom|BA.1|2022-01-04	96,03
EPI_ISL_8575594	BA.1.17.2	BA.1.15	BA.1.15	QEUH-30F1133|EPI_ISL_8575594|United Kingdom|BA.1.17.2|2022-01-03	90,98
EPI_ISL_8575951	BA.1	BA.1	BA.1_3	MILK-30BC6DB|EPI_ISL_8575951|United Kingdom|BA.1|2022-01-02	96,03
EPI_ISL_8576657	BA.1.17.2	BA.1.17.2	BA.1.17.2	NC-ECU-CORVASEQ-111474774|EPI_ISL_8576657|USA|BA.1.17.2|2022-01-04	99,62
EPI_ISL_8578509	BA.1	BA.1	BA.1_1	DCGC-302241|EPI_ISL_8578509|Denmark|BA.1|2022-01-02	99,69
EPI_ISL_8578514	BA.1.18	BA.1	BA.1_1	DCGC-302246|EPI_ISL_8578514|Denmark|BA.1.18|2022-01-02	99,68
EPI_ISL_8578558	BA.2.9	A	A_17	DCGC-302297|EPI_ISL_8578558|Denmark|BA.2.9|2022-01-02	99,81
EPI_ISL_8579062	BA.1.18	BA.1	BA.1_3	DCGC-302903|EPI_ISL_8579062|Denmark|BA.1.18|2022-01-03	99,62
EPI_ISL_8579563	BA.1.17.2	BA.1	BA.1_2	DCGC-303515|EPI_ISL_8579563|Denmark|BA.1.17.2|2022-01-02	99,66
EPI_ISL_8580694	BA.1.17.2	BA.1.15	BA.1.15	PHWC-PGTXID|EPI_ISL_8580694|United Kingdom|BA.1.17.2|2021-12-15	91,97
EPI_ISL_8581428	BA.1.15	BA.1	BA.1_4	DCGC-304600|EPI_ISL_8581428|Denmark|BA.1.15|2022-01-02	98,05
EPI_ISL_8582291	BA.2	A	A_17	DCGC-305086|EPI_ISL_8582291|Denmark|BA.2|2022-01-02	99,79
EPI_ISL_8582823	BA.1	BA.1	BA.1_1	DCGC-305737|EPI_ISL_8582823|Denmark|BA.1|2022-01-02	99,69
EPI_ISL_8583171	BA.2	A	A_17	DCGC-306173|EPI_ISL_8583171|Denmark|BA.2|2022-01-02	99,98
EPI_ISL_8585083	BA.1	A	A_1	DCGC-306277|EPI_ISL_8585083|Denmark|BA.1|2022-01-02	72,05
EPI_ISL_8590187	BA.1.17	BA.1.15	BA.1.15	TI-ETHZ-35508699|EPI_ISL_8590187|Switzerland|BA.1.17|2021-12-30	90,23
EPI_ISL_8592832	BA.1.17.2	BA.1.17.2	BA.1.17.2	NY-CDC-FG-226782|EPI_ISL_8592832|USA|BA.1.17.2|2021-12-28	99,52
EPI_ISL_8595364	BA.1.1.16	BA.1.1	BA.1.1_2	AB-ABPHL-47033|EPI_ISL_8595364|Canada|BA.1.1.16|2021-12-21	99,64
EPI_ISL_8596841	BA.1.1	BA.1.1	BA.1.1_2	MA-CDCBI-CRSP_CKOJCMLQUXYI2BQV|EPI_ISL_8596841|USA|BA.1.1|2022-01-03	93,53
EPI_ISL_8598977	BA.1.1	BA.2	BA.2_1	NY-UB-KSL-04405|EPI_ISL_8598977|USA|BA.1.1|2021-12-14	91,20
EPI_ISL_8599237	BA.1.1	BA.1.1	BA.1.1_2	UT-CDC-LC0452138|EPI_ISL_8599237|USA|BA.1.1|2021-12-21	99,64
EPI_ISL_8599330	BA.1.1	BA.1.1	BA.1.1_3	NJ-CDC-LC0447737|EPI_ISL_8599330|USA|BA.1.1|2021-12-21	99,64
EPI_ISL_8600073	BA.1.15	BA.1.15	BA.1.15	FL-CDC-LC0448018|EPI_ISL_8600073|USA|BA.1.15|2021-12-26	99,64
EPI_ISL_8600587	BA.1.1	BA.1.1	BA.1.1_3	FL-CDC-LC0450186|EPI_ISL_8600587|USA|BA.1.1|2021-12-28	99,64
EPI_ISL_8602146	BA.1.1.18	BA.1.1	BA.1.1_3	SC-CDC-LC0450488|EPI_ISL_8602146|USA|BA.1.1.18|2021-12-29	99,64
EPI_ISL_8602755	BA.1.1	BA.1.1	BA.1.1_2	GA-CDC-LC0450600|EPI_ISL_8602755|USA|BA.1.1|2021-12-29	99,64
EPI_ISL_8604858	BA.1.1	BA.1.1	BA.1.1_3	NJ-PHEL-V21036989|EPI_ISL_8604858|USA|BA.1.1|2021-12-12	79,53
EPI_ISL_8605049	BA.1.13	BA.1	BA.1_2	CAM-IZSM-RD048772D56-IZSM-COLLI-TIGEM|EPI_ISL_8605049|Italy|BA.1.13|2021-12-21	92,12
EPI_ISL_8607551	BA.1.1.14	BA.1.1	BA.1.1_2	CA-CDC-FG-225018|EPI_ISL_8607551|USA|BA.1.1.14|2021-12-27	95,61
EPI_ISL_8612357	BA.1.17.2	BA.1.15	BA.1.15	MILK-3106CDF|EPI_ISL_8612357|United Kingdom|BA.1.17.2|2022-01-03	90,98
EPI_ISL_8614746	BA.1.17.2	BA.1.17.2	BA.1.17.2	ALDP-3131822|EPI_ISL_8614746|United Kingdom|BA.1.17.2|2022-01-04	95,99
EPI_ISL_8615666	BA.1.17.2	BA.1.17.2	BA.1.17.2	ALDP-311D5F8|EPI_ISL_8615666|United Kingdom|BA.1.17.2|2022-01-03	94,09
EPI_ISL_8619040	BA.1	BA.1	BA.1_3	MILK-312FC67|EPI_ISL_8619040|United Kingdom|BA.1|2022-01-04	96,03
EPI_ISL_8619943	BA.1.1	BA.1.1	BA.1.1_2	MILK-30EC186|EPI_ISL_8619943|United Kingdom|BA.1.1|2022-01-03	96,03
EPI_ISL_8621000	BA.1	BA.1	BA.1_3	ALDP-30F490B|EPI_ISL_8621000|United Kingdom|BA.1|2022-01-02	95,75
EPI_ISL_8622056	BA.1.1	BA.1.1	BA.1.1_3	WA-CDC-UW21122983967|EPI_ISL_8622056|USA|BA.1.1|2021-12-29	87,14
EPI_ISL_8623625	BA.1.17.2	A	A_2	PIE-IRCC-15868968|EPI_ISL_8623625|Italy|BA.1.17.2|2021-12-27	96,87
EPI_ISL_8625871	BA.1	BA.1.15	BA.1.15	MAR_UNIVPM_Marche_75588-03-01-22|EPI_ISL_8625871|Italy|BA.1|2022-01-03	72,56
EPI_ISL_8628871	BA.1.1	BA.1.1	BA.1.1_3	SIN-INMEGEN-48-184|EPI_ISL_8628871|Mexico|BA.1.1|2021-12-26	95,73
EPI_ISL_8629111	BA.1.1	BA.1.1	BA.1.1_2	ID-IBL-779508|EPI_ISL_8629111|USA|BA.1.1|2021-12-27	99,60
EPI_ISL_8630577	BA.1.1	BA.1.1	BA.1.1_2	CA-CDC-FG-229464|EPI_ISL_8630577|USA|BA.1.1|2021-12-31	98,09
EPI_ISL_8630702	BA.1.1	BA.1	BA.1_4	NY-NYCPHL-008735|EPI_ISL_8630702|USA|BA.1.1|2021-12-29	88,84
EPI_ISL_8632160	BA.1.20	BA.1	BA.1_3	CA-CDC-FG-230712|EPI_ISL_8632160|USA|BA.1.20|2022-01-02	97,20
EPI_ISL_8632235	BA.1.1	BA.1.1	BA.1.1_2	CA-CDC-FG-229816|EPI_ISL_8632235|USA|BA.1.1|2022-01-02	98,09
EPI_ISL_8632585	BA.1.15	BA.1	BA.1_3	CA-CDC-FG-230786|EPI_ISL_8632585|USA|BA.1.15|2022-01-03	95,52
EPI_ISL_8632733	BA.1.18	BA.1	BA.1_3	PA-CDC-ASC210569315|EPI_ISL_8632733|USA|BA.1.18|2021-12-26	97,81
EPI_ISL_8638158	BA.1.1.18	A	A_1	CA-Curative-172596|EPI_ISL_8638158|USA|BA.1.1.18|2022-01-02	62,24
EPI_ISL_8643328	BA.1.17	BA.1.15	BA.1.15	VIC32803|EPI_ISL_8643328|Australia|BA.1.17|2021-12-25	93,25
EPI_ISL_8645955	BA.1.17	BA.1	BA.1_1	VIC34130|EPI_ISL_8645955|Australia|BA.1.17|2021-12-29	97,06
EPI_ISL_8647975	BA.1.17.2	BA.1.15	BA.1.15	CVL-23525|EPI_ISL_8647975|Israel|BA.1.17.2|2022-01-01	73,48
EPI_ISL_8648921	BA.1.21	BA.1.15	BA.1.15	Ahus-2769|EPI_ISL_8648921|Norway|BA.1.21|2022-01-09	90,98
EPI_ISL_8652392	BA.1.18	BA.1.15	BA.1.15	MH-RFIP07358|EPI_ISL_8652392|India|BA.1.18|2021-12-27	81,07
EPI_ISL_8655465	BA.1.17.2	BA.1.17.2	BA.1.17.2	MILK-314FD9B|EPI_ISL_8655465|United Kingdom|BA.1.17.2|2022-01-06	96,03
EPI_ISL_8657219	BA.1.16	BA.1	BA.1_3	BRBR-3143E2B|EPI_ISL_8657219|United Kingdom|BA.1.16|2022-01-04	96,03
EPI_ISL_8658660	BA.2.9	BA.2	BA.2_1	DCGC-309737|EPI_ISL_8658660|Denmark|BA.2.9|2022-01-05	99,79
EPI_ISL_8659294	BA.1.17.2	BA.1.15	BA.1.15	NEWC-3154BD5|EPI_ISL_8659294|United Kingdom|BA.1.17.2|2022-01-06	90,98
EPI_ISL_8659361	BA.1	BA.1	BA.1_3	ALDP-31550C6|EPI_ISL_8659361|United Kingdom|BA.1|2022-01-03	96,03
EPI_ISL_8659714	BA.1	BA.1.15	BA.1.15	NEWC-3161CE1|EPI_ISL_8659714|United Kingdom|BA.1|2022-01-06	90,98
EPI_ISL_8660173	BA.1.15.1	BA.1.15	BA.1.15	LSPA-30A1DD4|EPI_ISL_8660173|United Kingdom|BA.1.15.1|2021-12-28	96,01
EPI_ISL_8660705	BA.1.17.2	BA.1.17.2	BA.1.17.2	QEUH-315D5B0|EPI_ISL_8660705|United Kingdom|BA.1.17.2|2022-01-05	96,03
EPI_ISL_8663554	BA.1.15.1	BA.1.15	BA.1.15	PLYM-312BB7D|EPI_ISL_8663554|United Kingdom|BA.1.15.1|2021-12-09	92,88
EPI_ISL_8665114	BA.1	BA.1	BA.1_3	MILK-30E75DA|EPI_ISL_8665114|United Kingdom|BA.1|2022-01-03	96,03
EPI_ISL_8665467	BA.1	BA.1.15	BA.1.15	BW-RKI-I-418871|EPI_ISL_8665467|Germany|BA.1|2021-12-27	91,49
EPI_ISL_8667511	BA.1	BA.1.15	BA.1.15	NW-RKI-I-420976|EPI_ISL_8667511|Germany|BA.1|2021-12-30	98,95
EPI_ISL_8668961	BA.1	BA.1	BA.1_2	BW-RKI-I-422830|EPI_ISL_8668961|Germany|BA.1|2021-12-22	99,20
EPI_ISL_8670937	BA.1.17.2	BA.1.15	BA.1.15	BW-RKI-I-423898|EPI_ISL_8670937|Germany|BA.1.17.2|2021-12-29	91,49
EPI_ISL_8672721	BA.1.18	BA.1.15	BA.1.15	NAQ-HCL022001921101|EPI_ISL_8672721|France|BA.1.18|2022-01-03	91,30
EPI_ISL_8676660	BA.1.1	BA.1.1	BA.1.1_2	WA-PHL-009774|EPI_ISL_8676660|USA|BA.1.1|2021-12-17	96,03

EPI_ISL_8680744	BA.1.18	BA.1.15	BA.1.15	BW-RKI-I-431856|EPI_ISL_8680744|Germany|BA.1.18|2021-12-30	90,19
EPI_ISL_8681806	BA.1	BA.1.15	BA.1.15	VS-ICH-2211274862|EPI_ISL_8681806|Switzerland|BA.1|2021-12-25	99,64
EPI_ISL_8684142	BA.1.17.2	BA.1.17.2	BA.1.17.2	NW-RKI-I-435137|EPI_ISL_8684142|Germany|BA.1.17.2|2021-12-31	99,07
EPI_ISL_8685553	BA.1.1	BA.1.1	BA.1.1_2	SH-RKI-I-436360|EPI_ISL_8685553|Germany|BA.1.1|2021-12-28	99,64
EPI_ISL_8688488	BA.2	BA.2	BA.2_1	AZDelta-2201-13110|EPI_ISL_8688488|Belgium|BA.2|2022-01-10	97,61
EPI_ISL_8690228	BA.1	BA.1	BA.1_1	MA-CDCBI-CRSP_336FMYWVTVPKENA2|EPI_ISL_8690228|USA|BA.1|2022-01-04	93,70
EPI_ISL_8692118	BA.1	BA.1	BA.1_1	NY-CDCBI-CRSP_JHUWBFSE57Y4N72F|EPI_ISL_8692118|USA|BA.1|2022-01-04	93,28
EPI_ISL_8695730	BA.1	BA.1.15	BA.1.15	LSPA-31741A0|EPI_ISL_8695730|United Kingdom|BA.1|2022-01-06	94,09
EPI_ISL_8697325	BA.1	BA.1.15	BA.1.15	LSPA-316E67D|EPI_ISL_8697325|United Kingdom|BA.1|2022-01-04	94,09
EPI_ISL_8698104	BA.1	BA.1	BA.1_3	BRBR-3148688|EPI_ISL_8698104|United Kingdom|BA.1|2022-01-02	96,03
EPI_ISL_8698892	BA.1.17.2	BA.1.17.2	BA.1.17.2	QEUH-3171334|EPI_ISL_8698892|United Kingdom|BA.1.17.2|2022-01-04	96,03
EPI_ISL_8699666	BA.1	BA.1.15	BA.1.15	QEUH-3167832|EPI_ISL_8699666|United Kingdom|BA.1|2022-01-05	90,98
EPI_ISL_8699960	BA.1.17.2	BA.1.17.2	BA.1.17.2	PLYM-316C3F0|EPI_ISL_8699960|United Kingdom|BA.1.17.2|2022-01-04	94,85
EPI_ISL_8700102	BA.1	BA.1.15	BA.1.15	QEUH-31582CD|EPI_ISL_8700102|United Kingdom|BA.1|2022-01-05	90,98
EPI_ISL_8700213	BA.1.1.15	BA.1.1	BA.1.1_2	MILK-315A261|EPI_ISL_8700213|United Kingdom|BA.1.1.15|2022-01-05	96,01
EPI_ISL_8701584	BA.1	BA.1.15	BA.1.15	QEUH-316163E|EPI_ISL_8701584|United Kingdom|BA.1|2022-01-05	92,88
EPI_ISL_8702994	BA.1.15	BA.1.15	BA.1.15	NORT-YBED18|EPI_ISL_8702994|United Kingdom|BA.1.15|2021-12-22	90,98
EPI_ISL_8703326	BA.1.15.1	BA.1.15	BA.1.15	NORT-YBEJPC|EPI_ISL_8703326|United Kingdom|BA.1.15.1|2021-12-23	96,03
EPI_ISL_8704064	BA.1.1	BA.1.1	BA.1.1_2	QEUH-31591DE|EPI_ISL_8704064|United Kingdom|BA.1.1|2022-01-05	96,03
EPI_ISL_8704441	BA.1.1	BA.1.1	BA.1.1_2	QEUH-31661CF|EPI_ISL_8704441|United Kingdom|BA.1.1|2022-01-06	96,03
EPI_ISL_8705062	BA.2	BA.2	BA.2_1	DCGC-311656|EPI_ISL_8705062|Denmark|BA.2|2022-01-09	99,81
EPI_ISL_8705437	BA.1	BA.1	BA.1_3	QEUH-3177D8B|EPI_ISL_8705437|United Kingdom|BA.1|2022-01-05	96,03
EPI_ISL_8706830	BA.1.15.1	BA.1.15	BA.1.15	PLYM-2FD836D|EPI_ISL_8706830|United Kingdom|BA.1.15.1|2021-12-23	94,10
EPI_ISL_8707065	BA.1	BA.1.15	BA.1.15	PLYM-2FDB106|EPI_ISL_8707065|United Kingdom|BA.1|2021-12-23	94,09
EPI_ISL_8708271	BA.1	BA.1.15	BA.1.15	DCGC-309245|EPI_ISL_8708271|Denmark|BA.1|2022-01-07	84,53
EPI_ISL_8709640	BA.1.18	BA.1	BA.1_3	PAC-IHU-57797_Nova1|EPI_ISL_8709640|France|BA.1.18|2022-01	99,18
EPI_ISL_8711888	BA.1.13	BA.1.15	BA.1.15	BY-LGL-34ddccd|EPI_ISL_8711888|Germany|BA.1.13|2021-12-03	91,76
EPI_ISL_8716089	BA.1.15	BA.1	BA.1_4	LIM-INS-11288|EPI_ISL_8716089|Peru|BA.1.15|2021-12-20	99,64
EPI_ISL_8717523	BA.1.20	BA.1	BA.1_4	AZ-CDC-LC0457041|EPI_ISL_8717523|USA|BA.1.20|2021-12-21	99,64
EPI_ISL_8717701	BA.1.1.18	BA.1.1	BA.1.1_3	IA-CDC-LC0455315|EPI_ISL_8717701|USA|BA.1.1.18|2021-12-26	99,64
EPI_ISL_8717918	BA.1.1.1	BA.1.1	BA.1.1_2	MD-HGUGM-6454790|EPI_ISL_8717918|Spain|BA.1.1.1|2022-01-06	91,91
EPI_ISL_8723790	BA.1	BA.1.15	BA.1.15	ULG-23578|EPI_ISL_8723790|Belgium|BA.1|2022-01-07	93,30
EPI_ISL_8725449	BA.1	BA.1.15	BA.1.15	CT-JAX-JAX-2202.02-070|EPI_ISL_8725449|USA|BA.1|2021-12-30	99,64
EPI_ISL_8726358	BA.1.15	BA.1	BA.1_1	MA-CDCBI-CRSP_3PLE5PF4YVXQAQW6|EPI_ISL_8726358|USA|BA.1.15|2022-01-05	95,12
EPI_ISL_8730028	BA.1	BA.1.15	BA.1.15	UT-UPHL-220113732666|EPI_ISL_8730028|USA|BA.1|2022-01-05	99,64
EPI_ISL_8730185	BA.1.1	BA.1.1	BA.1.1_2	UT-UPHL-220113492214|EPI_ISL_8730185|USA|BA.1.1|2022-01-05	99,64
EPI_ISL_8733812	BA.1.16	BA.1	BA.1_3	MILK-31ABF1F|EPI_ISL_8733812|United Kingdom|BA.1.16|2022-01-05	96,03
EPI_ISL_8733939	BA.1.1	BA.1.1	BA.1.1_2	BRBR-31A40B9|EPI_ISL_8733939|United Kingdom|BA.1.1|2022-01-05	94,09
EPI_ISL_8735724	BA.1.1	BA.1.1	BA.1.1_2	ALDP-31AB401|EPI_ISL_8735724|United Kingdom|BA.1.1|2022-01-05	94,09
EPI_ISL_8736165	BA.1.1	BA.1.1	BA.1.1_2	BRBR-319EB57|EPI_ISL_8736165|United Kingdom|BA.1.1|2022-01-06	95,15
EPI_ISL_8739632	BA.1.15.1	BA.1.15	BA.1.15	BRBR-3190A76|EPI_ISL_8739632|United Kingdom|BA.1.15.1|2022-01-06	90,98
EPI_ISL_8741824	BA.1.17.2	BA.1.17.2	BA.1.17.2	MILK-319DC19|EPI_ISL_8741824|United Kingdom|BA.1.17.2|2022-01-08	96,03
EPI_ISL_8742311	BA.1	BA.1	BA.1_3	QEUH-3186FDE|EPI_ISL_8742311|United Kingdom|BA.1|2022-01-05	96,03
EPI_ISL_8744671	BA.1.21	BA.1.15	BA.1.15	33044|EPI_ISL_8744671|Norway|BA.1.21|2021-12-16	99,64
EPI_ISL_8752739	BA.1.1	BA.1	BA.1_1	MA-CDCBI-CRSP_YRP6VBE7AWYRRJ2G|EPI_ISL_8752739|USA|BA.1.1|2022-01-06	94,94
EPI_ISL_8757857	BA.1.1	BA.1.1	BA.1.1_3	MILK-31C4077|EPI_ISL_8757857|United Kingdom|BA.1.1|2022-01-09	99,64
EPI_ISL_8757906	BA.1.17.2	BA.1.17.2	BA.1.17.2	MILK-31C3F20|EPI_ISL_8757906|United Kingdom|BA.1.17.2|2022-01-09	99,64
EPI_ISL_8758908	BA.1.17.2	BA.1.17.2	BA.1.17.2	ALDP-31C3A4D|EPI_ISL_8758908|United Kingdom|BA.1.17.2|2022-01-06	99,64
EPI_ISL_8759702	BA.1.17.2	BA.1.17.2	BA.1.17.2	NEWC-31B3A04|EPI_ISL_8759702|United Kingdom|BA.1.17.2|2022-01-07	90,48
EPI_ISL_8762781	BA.1.15	BA.1	BA.1_1	GA-EVTL10496|EPI_ISL_8762781|USA|BA.1.15|2021-12-20	93,36
EPI_ISL_8763238	BA.1.15.1	BA.1.15	BA.1.15	PHWC-PGU1BF|EPI_ISL_8763238|United Kingdom|BA.1.15.1|2021-12-20	96,03
EPI_ISL_8763299	BA.1	BA.1	BA.1_4	PHWC-PGUB3F|EPI_ISL_8763299|United Kingdom|BA.1|2021-12-20	95,92
EPI_ISL_8766581	BA.1.1	BA.1.1	BA.1.1_2	FL-CDC-ASC210576248|EPI_ISL_8766581|USA|BA.1.1|2021-12-30	98,24
EPI_ISL_8766658	BA.1.1	BA.1.1	BA.1.1_2	CA-CDC-ASC210575770|EPI_ISL_8766658|USA|BA.1.1|2021-12-30	99,64
EPI_ISL_8767471	BA.2	BA.2	BA.2_1	DCGC-312760|EPI_ISL_8767471|Denmark|BA.2|2022-01-07	99,79
EPI_ISL_8768435	BA.1.17	BA.1.15	BA.1.15	ACT2611|EPI_ISL_8768435|Australia|BA.1.17|2022-01-02	99,60
EPI_ISL_8770509	BA.2	A	A_14	507|EPI_ISL_8770509|Singapore|BA.2|2022-01-11	99,81
EPI_ISL_8772541	BA.1.17.2	BA.1.17.2	BA.1.17.2	MILK-31F61D3|EPI_ISL_8772541|United Kingdom|BA.1.17.2|2022-01-10	99,64
EPI_ISL_8772944	BA.1	BA.1.15	BA.1.15	BRBR-31DA68B|EPI_ISL_8772944|United Kingdom|BA.1|2022-01-08	99,56
EPI_ISL_8773610	BA.1.17	BA.1.15	BA.1.15	318997|EPI_ISL_8773610|Greece|BA.1.17|2021-12-22	99,64
EPI_ISL_8775808	BA.1.18	BA.1.15	BA.1.15	MILK-31E957D|EPI_ISL_8775808|United Kingdom|BA.1.18|2022-01-10	99,64
EPI_ISL_8775845	BA.1	BA.1.15	BA.1.15	QEUH-31E9FE8|EPI_ISL_8775845|United Kingdom|BA.1|2022-01-06	99,64
EPI_ISL_8776111	BA.1.17.2	BA.1.17.2	BA.1.17.2	LSPA-31E3018|EPI_ISL_8776111|United Kingdom|BA.1.17.2|2022-01-06	99,64
EPI_ISL_8776988	BA.1	BA.1	BA.1_4	MILK-31CD9CE|EPI_ISL_8776988|United Kingdom|BA.1|2022-01-09	99,64
EPI_ISL_8778179	BA.1.1.15	BA.1.1	BA.1.1_3	LSPA-31EDD53|EPI_ISL_8778179|United Kingdom|BA.1.1.15|2022-01-06	99,64
EPI_ISL_8779285	BA.1.17	BA.1.15	BA.1.15	MILK-31E2750|EPI_ISL_8779285|United Kingdom|BA.1.17|2022-01-10	99,64
EPI_ISL_8779439	BA.1.17.2	BA.1.17.2	BA.1.17.2	LSPA-31EE46C|EPI_ISL_8779439|United Kingdom|BA.1.17.2|2022-01-06	99,64
EPI_ISL_8780954	BA.1	BA.1.15	BA.1.15	NORW-312323E|EPI_ISL_8780954|United Kingdom|BA.1|2021-12-20	83,92
EPI_ISL_8784099	BA.1.18	BA.1.15	BA.1.15	PHWC-PGIDNQ|EPI_ISL_8784099|United Kingdom|BA.1.18|2021-12-25	90,98
EPI_ISL_8785703	BA.1	BA.2	BA.2_1	BL-ETHZ-35456364|EPI_ISL_8785703|Switzerland|BA.1|2021-12-26	91,78
EPI_ISL_8789290	BA.1.18	BA.1	BA.1_4	IPP52797|EPI_ISL_8789290|Guadeloupe|BA.1.18|2021-12-17	99,64
EPI_ISL_8790008	BA.1.17.2	BA.1.17.2	BA.1.17.2	TI-ETHZ-35515991|EPI_ISL_8790008|Switzerland|BA.1.17.2|2021-12-31	99,69
EPI_ISL_8791549	BA.1	BA.1.15	BA.1.15	LAZ-AMC-220103922-DS|EPI_ISL_8791549|Italy|BA.1|2022-01-03	91,28
EPI_ISL_8794699	BA.1.17.2	BA.1.17.2	BA.1.17.2	BC-BCCDC-298206|EPI_ISL_8794699|Canada|BA.1.17.2|2021-12-17	99,64
EPI_ISL_8798171	BA.1.1	BA.1.1	BA.1.1_2	CO-CDPHE-2102576727|EPI_ISL_8798171|USA|BA.1.1|2022-01-03	92,14
EPI_ISL_8798530	BA.1.1	BA.1.1	BA.1.1_2	WA-PHL-010396|EPI_ISL_8798530|USA|BA.1.1|2022-01-07	96,03
EPI_ISL_8799295	BA.1	BA.1.15	BA.1.15	NICD-R18866|EPI_ISL_8799295|South Africa|BA.1|2021-12-22	99,64
EPI_ISL_8799349	BA.1.18	BA.1.15	BA.1.15	CERI-KRISP-K034159|EPI_ISL_8799349|Zimbabwe|BA.1.18|2021-12-13	99,64
EPI_ISL_8799762	BA.1	BA.1.15	BA.1.15	PZH-GUM-6058|EPI_ISL_8799762|Poland|BA.1|2022-01-07	99,64
EPI_ISL_8804124	BA.1.19	BA.1	BA.1_3	DCGC-313821|EPI_ISL_8804124|Denmark|BA.1.19|2022-01-10	96,01
EPI_ISL_8804158	BA.1	BA.1	BA.1_3	NORT-YBKHDJ|EPI_ISL_8804158|United Kingdom|BA.1|2021-12-27	94,92
EPI_ISL_8805076	BA.1	BA.1.15	BA.1.15	DCGC-314240|EPI_ISL_8805076|Denmark|BA.1|2021-12-27	99,64
EPI_ISL_8813152	BA.1.15	BA.1	BA.1_3	PHWC-PGSSGT|EPI_ISL_8813152|United Kingdom|BA.1.15|2021-12-27	94,91
EPI_ISL_8813786	BA.1.1	BA.1.1	BA.1.1_2	PHWC-PGZF33|EPI_ISL_8813786|United Kingdom|BA.1.1|2021-12-27	91,76
EPI_ISL_8815763	BA.1.18	BA.1.15	BA.1.15	CA-CDC-ASC210574936|EPI_ISL_8815763|USA|BA.1.18|2021-12-29	99,64
EPI_ISL_8815859	BA.1.1	BA.1.1	BA.1.1_2	WI-CDC-ASC210500120|EPI_ISL_8815859|USA|BA.1.1|2021-12-30	99,64
EPI_ISL_8817742	BA.1.16	BA.1	BA.1_3	LSPA-320E2D4|EPI_ISL_8817742|United Kingdom|BA.1.16|2022-01-06	99,64
EPI_ISL_8817925	BA.1.1.13	BA.1.1	BA.1.1_2	MILK-320F8D1|EPI_ISL_8817925|United Kingdom|BA.1.1.13|2022-01-11	99,64
EPI_ISL_8818628	BA.1	BA.1.15	BA.1.15	MILK-32309A7|EPI_ISL_8818628|United Kingdom|BA.1|2022-01-13	99,64
EPI_ISL_8818645	BA.1.17	BA.1.15	BA.1.15	AS-232204521|EPI_ISL_8818645|Spain|BA.1.17|2021-12-21	98,42
EPI_ISL_8819512	BA.1.1	BA.1.1	BA.1.1_2	SC-MUSC-05829|EPI_ISL_8819512|USA|BA.1.1|2021-12-29	99,64
EPI_ISL_8819839	BA.2	A	A_14	BRBR-321D6D6|EPI_ISL_8819839|United Kingdom|BA.2|2022-01-10	99,81
EPI_ISL_8821854	BA.1.17.2	BA.1.17.2	BA.1.17.2	HSLL-32022D0|EPI_ISL_8821854|United Kingdom|BA.1.17.2|2022-01-10	99,64
EPI_ISL_8822120	BA.1	BA.1	BA.1_4	LSPA-3201ECE|EPI_ISL_8822120|United Kingdom|BA.1|2022-01-08	99,64
EPI_ISL_8822325	BA.1.1	BA.1	BA.1_4	WA-S16009|EPI_ISL_8822325|USA|BA.1.1|2021-12-30	91,62
EPI_ISL_8822506	BA.1.15	BA.1	BA.1_3	WA-S16186|EPI_ISL_8822506|USA|BA.1.15|2021-12-22	96,60
EPI_ISL_8824540	BA.1	BA.1.15	BA.1.15	AZ-TG1154413|EPI_ISL_8824540|USA|BA.1|2022-01-03	99,64
EPI_ISL_8831607	BA.1.1.18	BA.1.1	BA.1.1_3	TN-CDC-ASC210559009|EPI_ISL_8831607|USA|BA.1.1.18|2021-12-30	99,64
EPI_ISL_8831802	BA.1.20	BA.1.15	BA.1.15	TN-CDC-ASC210560236|EPI_ISL_8831802|USA|BA.1.20|2021-12-30	99,64
EPI_ISL_8831812	BA.1.17.2	BA.1.17.2	BA.1.17.2	DCGC-315436|EPI_ISL_8831812|Denmark|BA.1.17.2|2022-01-13	99,64
EPI_ISL_8832461	BA.2	A	A_17	DCGC-315848|EPI_ISL_8832461|Denmark|BA.2|2022-01-12	97,21

EPI_ISL_8834725	BA.1.17.2	BA.1.15	BA.1.15	DCGC-317175|EPI_ISL_8834725|Denmark|BA.1.17.2|2022-01-13	95,44
EPI_ISL_8834905	BA.1.17.2	BA.1.17.2	BA.1.17.2	DCGC-317284|EPI_ISL_8834905|Denmark|BA.1.17.2|2022-01-11	99,64
EPI_ISL_8836127	BA.1.1	BA.1.1	BA.1.1_2	CA-CDC-FG-231982|EPI_ISL_8836127|USA|BA.1.1|2022-01-03	96,87
EPI_ISL_8836775	BA.1.1	BA.1.1	BA.1.1_2	CA-CDC-FG-231735|EPI_ISL_8836775|USA|BA.1.1|2022-01-03	90,99
EPI_ISL_8837278	BA.1	BA.1.15	BA.1.15	CA-CDC-FG-232328|EPI_ISL_8837278|USA|BA.1|2022-01-03	95,42
EPI_ISL_8838094	BA.1.17.2	BA.1	BA.1_4	2201322427744|EPI_ISL_8838094|Poland|BA.1.17.2|2022-01-18	93,34
EPI_ISL_8839573	BA.1.1	BA.1.1	BA.1.1_2	PHEC-4M050Z53|EPI_ISL_8839573|United Kingdom|BA.1.1|2022	96,20
EPI_ISL_8839799	BD.1	BD.1	BD.1	PHEC-4M06BZ51|EPI_ISL_8839799|United Kingdom|BD.1|2022-01-02	99,54
EPI_ISL_8839950	BA.1.17	BA.1	BA.1_4	PHEC-4M075Z9E|EPI_ISL_8839950|United Kingdom|BA.1.17|2022	92,65
EPI_ISL_8840133	BA.1.1	BA.1.15	BA.1.15	PHEC-4M07DZ1E|EPI_ISL_8840133|United Kingdom|BA.1.1|2022-01-02	73,97
EPI_ISL_8847300	BA.1.1	BA.1.1	BA.1.1_2	MI-MDHHS-SC38353|EPI_ISL_8847300|USA|BA.1.1|2022-01-07	96,30
EPI_ISL_8847541	BA.1	BA.1.15	BA.1.15	MILK-326BE4A|EPI_ISL_8847541|United Kingdom|BA.1|2022-01-13	99,64
EPI_ISL_8852477	BA.1.15.1	BA.1.15	BA.1.15	PHWC-PGAKR7|EPI_ISL_8852477|United Kingdom|BA.1.15.1|2021-12-27	96,01
EPI_ISL_8852485	BA.1	BA.1	BA.1_4	ALDP-323FDCA|EPI_ISL_8852485|United Kingdom|BA.1|2022-01-11	99,64
EPI_ISL_8853742	BA.1	BA.1.15	BA.1.15	PHWC-PGZH4Y|EPI_ISL_8853742|United Kingdom|BA.1|2021-12-28	90,98
EPI_ISL_8855117	BA.1.15	BA.1.15	BA.1.15	08-268797-MB|EPI_ISL_8855117|Slovenia|BA.1.15|2021-12-31	97,06
EPI_ISL_8855591	BA.1.17.2	BA.1.17.2	BA.1.17.2	17-091184-KR|EPI_ISL_8855591|Slovenia|BA.1.17.2|2021-12-28	96,39
EPI_ISL_8857014	BA.1.17.2	BA.1.17.2	BA.1.17.2	90-081185-NM|EPI_ISL_8857014|Slovenia|BA.1.17.2|2021-12-30	97,06
EPI_ISL_8857275	BA.1.17.2	BA.1.17.2	BA.1.17.2	PLYM-3225705|EPI_ISL_8857275|United Kingdom|BA.1.17.2|2022-01-10	99,64
EPI_ISL_8858033	BA.1.21	BA.1.15	BA.1.15	UMB-IZSGC-3419_1_17|EPI_ISL_8858033|Italy|BA.1.21|2022-01-11	99,50
EPI_ISL_8858500	BA.1.17.2	BA.1.17.2	BA.1.17.2	ALDP-322F817|EPI_ISL_8858500|United Kingdom|BA.1.17.2|2022-01-08	99,64
EPI_ISL_8859346	BA.1.17.2	BA.1.17.2	BA.1.17.2	QEUH-3206852|EPI_ISL_8859346|United Kingdom|BA.1.17.2|2022-01-10	99,64
EPI_ISL_8860251	BA.1.1.14	BA.1.1	BA.1.1_3	NEWC-3225D5E|EPI_ISL_8860251|United Kingdom|BA.1.1.14|2022-01-08	99,64
EPI_ISL_8861779	BA.1	BA.1.15	BA.1.15	MILK-31AF2A9|EPI_ISL_8861779|United Kingdom|BA.1|2022-01-06	93,00
EPI_ISL_8862141	BA.1.1	BA.1.1	BA.1.1_2	NJ-CDC-IBX910589506906|EPI_ISL_8862141|USA|BA.1.1|2022-01-05	96,57
EPI_ISL_8862219	BA.1	BA.1	BA.1_4	ALDP-3233AA1|EPI_ISL_8862219|United Kingdom|BA.1|2022-01-11	99,64
EPI_ISL_8862778	BA.1.15	BA.1.15	BA.1.15	ALDP-323D816|EPI_ISL_8862778|United Kingdom|BA.1.15|2022-01-11	99,64
EPI_ISL_8866166	BA.1.17.2	BA.1.17.2	BA.1.17.2	ALDP-31B754B|EPI_ISL_8866166|United Kingdom|BA.1.17.2|2022-01-05	96,03
EPI_ISL_8866270	BA.1.15.1	BA.1.15	BA.1.15	PLYM-31B7B94|EPI_ISL_8866270|United Kingdom|BA.1.15.1|2022-01-07	94,09
EPI_ISL_8872073	BA.1	BA.1.15	BA.1.15	FL-CDC-ASC210574534|EPI_ISL_8872073|USA|BA.1|2021-12-28	99,64
EPI_ISL_8872853	BA.1.1	BA.1.1	BA.1.1_2	MN-CDC-QDX32371085|EPI_ISL_8872853|USA|BA.1.1|2021-12-22	99,64
EPI_ISL_8874216	BA.1.1	BA.1.1	BA.1.1_3	TX-CDC-QDX32498513|EPI_ISL_8874216|USA|BA.1.1|2021-12-29	99,64
EPI_ISL_8874423	BA.1.1	BA.1.1	BA.1.1_2	AZ-TG1156489|EPI_ISL_8874423|USA|BA.1.1|2022-01-03	99,58
EPI_ISL_8875379	BA.1	BA.1	BA.1_1	MD-IGS-52134900479A|EPI_ISL_8875379|USA|BA.1|2021-12-15	96,53
EPI_ISL_8878203	BA.1.1	BA.1.1	BA.1.1_2	MA-CDCBI-CRSP_SFMIPYXUAKLGU4YY|EPI_ISL_8878203|USA|BA.1.1|2022-01-08	95,12
EPI_ISL_8878300	BA.1.1.18	BA.1	BA.1_1	VT-CDCBI-CRSP_2G6NQRYVLTSVFPT6|EPI_ISL_8878300|USA|BA.1.1.18|2022-01-08	90,73
EPI_ISL_8878512	BA.1.1	BA.1	BA.1_4	VT-CDCBI-CRSP_KHSZYEAK5MPXEFDG|EPI_ISL_8878512|USA|BA.1.1|2022-01-08	91,66
EPI_ISL_8879573	BA.1.17	BA.2	BA.2_1	DC-LSPSDS-VG-41724|EPI_ISL_8879573|Colombia|BA.1.17|2022-01-02	90,69
EPI_ISL_8879772	BA.1.15	BA.1.15	BA.1.15	CT-SEMA4-2560|EPI_ISL_8879772|USA|BA.1.15|2022-01-03	99,64
EPI_ISL_8881348	BA.1.1	B.1.617.2	B.1.617.2_2	DL-IGIB1120213627769TV|EPI_ISL_8881348|India|BA.1.1|2021-12	61,27
EPI_ISL_8881400	BA.2	BA.2.10	BA.2.10	HR-IGIB1210607600537402TV|EPI_ISL_8881400|India|BA.2|2022-01	99,54
EPI_ISL_8881412	BA.1	BA.1.17.2	BA.1.17.2	DL-IGIB1120213640083TV|EPI_ISL_8881412|India|BA.1|2022-01	76,80
EPI_ISL_8881766	BA.1	BA.2.12	BA.2.12	DL-IGIB1120220080037TV|EPI_ISL_8881766|India|BA.1|2022-01	73,36
EPI_ISL_8882014	BA.1.17	BA.1	BA.1_4	TAS000556|EPI_ISL_8882014|Australia|BA.1.17|2022	99,64
EPI_ISL_8883714	BA.1.17.2	BA.1.17.2	BA.1.17.2	NORT-YBCDCC|EPI_ISL_8883714|United Kingdom|BA.1.17.2|2022	92,88
EPI_ISL_8883739	BA.1.1	BA.1.1	BA.1.1_2	NORT-YBCH4S|EPI_ISL_8883739|United Kingdom|BA.1.1|2022-01-02	96,03
EPI_ISL_8884201	BA.1	BA.1	BA.1_3	NORT-YBMIUP|EPI_ISL_8884201|United Kingdom|BA.1|2021-12-31	96,03
EPI_ISL_8884560	BA.1.17.2	BA.1.15	BA.1.15	PHEC-YYB4AY1|EPI_ISL_8884560|United Kingdom|BA.1.17.2|2022-01-03	86,28
EPI_ISL_8885775	BA.1.1	BA.1	BA.1_4	DCGC-317613|EPI_ISL_8885775|Denmark|BA.1.1|2022-01-12	94,77
EPI_ISL_8886788	BA.1.1	BA.1.1	BA.1.1_3	DCGC-318002|EPI_ISL_8886788|Denmark|BA.1.1|2022-01-14	92,06
EPI_ISL_8887194	BA.2	A	A_17	DCGC-318381|EPI_ISL_8887194|Denmark|BA.2|2022-01-13	99,81
EPI_ISL_8889434	BA.1.18	BA.1	BA.1_3	IDF-CERBAHC-12940328|EPI_ISL_8889434|France|BA.1.18|2021-12-08	97,33
EPI_ISL_8890597	BA.1	BA.1.15	BA.1.15	UFS-180122BC71|EPI_ISL_8890597|South Africa|BA.1|2021-12-13	86,51
EPI_ISL_8890796	BA.1	BA.1	BA.1_4	PHWC-PG378N|EPI_ISL_8890796|United Kingdom|BA.1|2021-12-29	96,03
EPI_ISL_8891717	BA.1	BA.1	BA.1_3	GE-HUG-36706444|EPI_ISL_8891717|Switzerland|BA.1|2022-01-09	98,80
EPI_ISL_8891809	BA.1.1.1	BA.1.1	BA.1.1_2	GE-HUG-36730250|EPI_ISL_8891809|Switzerland|BA.1.1.1|2022-01-10	99,27
EPI_ISL_8892184	BA.1.1	BA.1.1	BA.1.1_2	MI-MDHHS-SC38269|EPI_ISL_8892184|USA|BA.1.1|2022-01-03	99,35
EPI_ISL_8895293	BA.1	BA.1.15	BA.1.15	QEUH-3242F73|EPI_ISL_8895293|United Kingdom|BA.1|2022-01-10	99,64
EPI_ISL_8896775	BA.1.17	BA.1.15	BA.1.15	01_SE100_22CS100210|EPI_ISL_8896775|Sweden|BA.1.17|2022-01-09	85,35
EPI_ISL_8902295	BA.1.15	BA.1	BA.1_1	AR-UMGC-31174|EPI_ISL_8902295|USA|BA.1.15|2022-01-04	90,73
EPI_ISL_8902590	BA.1	BA.1.1	BA.1.1_2	AR-UMGC-30803|EPI_ISL_8902590|USA|BA.1|2022-01-05	95,95
EPI_ISL_8903991	BA.1	BA.1.15	BA.1.15	BC-BCCDC-304797|EPI_ISL_8903991|Canada|BA.1|2021-12-12	99,64
EPI_ISL_8906748	BA.1	BA.1.15	BA.1.15	BC-BCCDC-311491|EPI_ISL_8906748|Canada|BA.1|2021-12-17	99,64
EPI_ISL_8906899	BA.1.17.2	BA.1.17.2	BA.1.17.2	BC-BCCDC-311685|EPI_ISL_8906899|Canada|BA.1.17.2|2021-12-16	99,64
EPI_ISL_8906946	BA.1.1	BA.1.1	BA.1.1_2	BC-BCCDC-311763|EPI_ISL_8906946|Canada|BA.1.1|2021-12-18	86,05
EPI_ISL_8909816	BA.1.17.2	BA.1.17.2	BA.1.17.2	BC-BCCDC-307690|EPI_ISL_8909816|Canada|BA.1.17.2|2021-12-17	99,64
EPI_ISL_8909844	BA.1	BA.1.15	BA.1.15	BC-BCCDC-307729|EPI_ISL_8909844|Canada|BA.1|2021-12-22	99,64
EPI_ISL_8909847	BA.1	BA.1.15	BA.1.15	BC-BCCDC-307735|EPI_ISL_8909847|Canada|BA.1|2021-12-22	99,64
EPI_ISL_8914380	BA.1.3	BA.1	BA.1_4	BC-BCCDC-315555|EPI_ISL_8914380|Canada|BA.1.3|2021-12-30	99,64
EPI_ISL_8914678	BA.1.17.2	BA.1.17.2	BA.1.17.2	BC-BCCDC-316885|EPI_ISL_8914678|Canada|BA.1.17.2|2021-12-31	99,64
EPI_ISL_8917786	BA.1.1	BA.1.1	BA.1.1_2	USA|EPI_ISL_8917786|USA|BA.1.1|2022-01-01	95,65
EPI_ISL_8919243	BA.1.1	BA.1.1	BA.1.1_2	CA-SEARCH-64777|EPI_ISL_8919243|USA|BA.1.1|2022-01-03	99,48
EPI_ISL_8920594	BA.1	BA.1.15	BA.1.15	EDB35370|EPI_ISL_8920594|United Kingdom|BA.1|2021-12-28	90,94
EPI_ISL_8921287	BA.1.1.10	BA.1.15	BA.1.15	PHEC-4M0B7Z76|EPI_ISL_8921287|United Kingdom|BA.1.1.10|2022	74,41
EPI_ISL_8921422	BA.2	A	A_17	DCGC-318834|EPI_ISL_8921422|Denmark|BA.2|2022-01-13	99,81
EPI_ISL_8922387	BA.2.9	A	A_17	DCGC-319382|EPI_ISL_8922387|Denmark|BA.2.9|2022-01-14	99,81
EPI_ISL_8922499	BA.2	A	A_14	PHEC-4N051ZBA|EPI_ISL_8922499|United Kingdom|BA.2|2022	99,81
EPI_ISL_8924996	BA.2.9	A	A_17	DCGC-320235|EPI_ISL_8924996|Denmark|BA.2.9|2022-01-13	99,81
EPI_ISL_8925394	BA.1.1.14	BA.1	BA.1_4	WSSEGDA-200|EPI_ISL_8925394|Poland|BA.1.1.14|2022-01-17	82,62
EPI_ISL_8925633	BA.1.1	BA.1	BA.1_4	ZNA_25075868|EPI_ISL_8925633|Belgium|BA.1.1|2022-01-06	88,57
EPI_ISL_8926879	BA.2.9	A	A_17	DCGC-320776|EPI_ISL_8926879|Denmark|BA.2.9|2022-01-14	99,79
EPI_ISL_8927170	BA.1.17.2	BA.1.17.2	BA.1.17.2	666|EPI_ISL_8927170|Singapore|BA.1.17.2|2022-01-10	99,64
EPI_ISL_8927198	BA.1.17	BA.1	BA.1_3	705|EPI_ISL_8927198|Singapore|BA.1.17|2022-01-16	99,64
EPI_ISL_8927716	BA.1.1	BA.1.1	BA.1.1_2	ZH-EMC-4691|EPI_ISL_8927716|Netherlands|BA.1.1|2022-01-10	95,95
EPI_ISL_8928468	BA.1.21	BA.1	BA.1_1	OUS-19559|EPI_ISL_8928468|Norway|BA.1.21|2022-01-11	99,64
EPI_ISL_8933296	BA.1	BA.1.15	BA.1.15	MILK-327F881|EPI_ISL_8933296|United Kingdom|BA.1|2022-01-14	99,64
EPI_ISL_8935002	BA.1.1	BA.1.1	BA.1.1_2	CA-CDC-FG-234839|EPI_ISL_8935002|USA|BA.1.1|2022-01-04	97,92
EPI_ISL_8935299	BA.1.1	BA.1.1	BA.1.1_3	NEWC-3249BF0|EPI_ISL_8935299|United Kingdom|BA.1.1|2022-01-12	99,60
EPI_ISL_8936644	BA.1.1	BA.1.1	BA.1.1_3	TKYkbm0005|EPI_ISL_8936644|Japan|BA.1.1|2022-01-05	99,64
EPI_ISL_8937921	BA.1.17.2	BA.1.17.2	BA.1.17.2	NY-NYULH5228|EPI_ISL_8937921|USA|BA.1.17.2|2022-01-10	95,90
EPI_ISL_8938289	BA.1.1	BA.1.1	BA.1.1_2	PAIS-G0689|EPI_ISL_8938289|Argentina|BA.1.1|2021-12-21	87,62
EPI_ISL_8940237	BA.1	BA.1.15	BA.1.15	MILK-3273401|EPI_ISL_8940237|United Kingdom|BA.1|2022-01-13	99,64
EPI_ISL_8942196	BA.1.16	BA.1.1	BA.1.1_2	ALDP-325EFED|EPI_ISL_8942196|United Kingdom|BA.1.16|2022-01-10	99,64
EPI_ISL_8943202	BA.1	BA.1.15	BA.1.15	ALDP-32638EA|EPI_ISL_8943202|United Kingdom|BA.1|2022-01-09	99,64
EPI_ISL_8944364	BA.1.15.1	BA.1.15.1	BA.1.15.1	BRBR-3264C47|EPI_ISL_8944364|United Kingdom|BA.1.15.1|2022-01-12	99,60
EPI_ISL_8944444	BA.1.1.14	BA.1.1	BA.1.1_2	MILK-3267A2C|EPI_ISL_8944444|United Kingdom|BA.1.1.14|2022-01-13	99,64
EPI_ISL_8945545	BA.1	BA.1.15	BA.1.15	QEUH-3259A78|EPI_ISL_8945545|United Kingdom|BA.1|2022-01-13	94,58
EPI_ISL_8945966	BA.1.15	BA.1.15	BA.1.15	NEWC-3251C2F|EPI_ISL_8945966|United Kingdom|BA.1.15|2022-01-12	99,64
EPI_ISL_8946828	BA.1.20	BA.1.15	BA.1.15	MN-CDC-IBX748350051916|EPI_ISL_8946828|USA|BA.1.20|2021-12-28	96,64
EPI_ISL_8947017	BA.1.1	BA.1.1	BA.1.1_2	NJ-CDC-IBX792229315235|EPI_ISL_8947017|USA|BA.1.1|2022-01-04	96,57
EPI_ISL_8947949	BA.1.1.18	BA.1.1	BA.1.1_2	CO-CDPHE-2102640013|EPI_ISL_8947949|USA|BA.1.1.18|2022-01-10	97,06

EPI_ISL_8948520	BA.1.15	BA.1	BA.1_4	CO-CDPHE-2102642350|EPI_ISL_8948520|USA|BA.1.15|2022-01-11	99,64
EPI_ISL_8948653	BA.1.1	BA.1.1	BA.1.1_2	CO-CDPHE-2102620632|EPI_ISL_8948653|USA|BA.1.1|2021-12-29	99,64
EPI_ISL_8949091	BA.1.1	BA.1.1	BA.1.1_2	CO-CDPHE-2102601427|EPI_ISL_8949091|USA|BA.1.1|2022-01-04	99,64
EPI_ISL_8951238	BA.1.20	BA.1	BA.1_3	AK-CDC-ASC210499797|EPI_ISL_8951238|USA|BA.1.20|2021-12-30	97,79
EPI_ISL_8952363	BA.1.1	BA.1.1	BA.1.1_2	UT-UPHL-220119019964|EPI_ISL_8952363|USA|BA.1.1|2022-01-10	99,64
EPI_ISL_8954886	BA.1.1	BA.1.1	BA.1.1_3	VIC34904|EPI_ISL_8954886|Australia|BA.1.1|2022-01-06	99,64
EPI_ISL_8956533	BA.1	BA.1	BA.1_1	MA-CDCBI-CRSP_EPMXVNVNO3NRDR56|EPI_ISL_8956533|USA|BA.1|2022-01-09	89,93
EPI_ISL_8958021	BA.1.1.14	BA.1.1	BA.1.1_3	LOND-YYBYCBT|EPI_ISL_8958021|United Kingdom|BA.1.1.14|2022-01-04	95,92
EPI_ISL_8958891	BA.1.1	BA.1.1	BA.1.1_2	NORT-YBXJOZ|EPI_ISL_8958891|United Kingdom|BA.1.1|2022	94,47
EPI_ISL_8959727	BA.1.17.2	BA.1.17.2	BA.1.17.2	PHEC-YYBHNGA|EPI_ISL_8959727|United Kingdom|BA.1.17.2|2022-01-02	96,70
EPI_ISL_8959733	BA.1	BA.1	BA.1_3	PHEC-YYBHNRH|EPI_ISL_8959733|United Kingdom|BA.1|2022-01-04	96,79
EPI_ISL_8961058	BA.1.17.2	BA.1.15	BA.1.15	PHWC-PG5JD4|EPI_ISL_8961058|United Kingdom|BA.1.17.2|2021-12-28	91,89
EPI_ISL_8961093	BA.1.15.1	BA.1.15	BA.1.15	PHWC-PG5M4J|EPI_ISL_8961093|United Kingdom|BA.1.15.1|2021-12-29	96,03
EPI_ISL_8961347	BA.1.17.2	BA.1.17.2	BA.1.17.2	PHWC-PG5RHC|EPI_ISL_8961347|United Kingdom|BA.1.17.2|2021-12-29	96,03
EPI_ISL_8962204	BA.1.17.2	BA.1.17.2	BA.1.17.2	MILK-32A4AD8|EPI_ISL_8962204|United Kingdom|BA.1.17.2|2022-01-15	99,64
EPI_ISL_8964841	BA.1.1	BA.1.1	BA.1.1_2	QEUH-328B6D3|EPI_ISL_8964841|United Kingdom|BA.1.1|2022-01-13	99,64
EPI_ISL_8966324	BA.1.17.2	BA.1.17.2	BA.1.17.2	QEUH-3299256|EPI_ISL_8966324|United Kingdom|BA.1.17.2|2022-01-13	99,64
EPI_ISL_8967201	BA.1.1	BA.1.1	BA.1.1_2	BRBR-329D841|EPI_ISL_8967201|United Kingdom|BA.1.1|2022-01-15	91,26
EPI_ISL_8967595	BA.1.15	BA.1.15	BA.1.15	QEUH-3290C9D|EPI_ISL_8967595|United Kingdom|BA.1.15|2022-01-15	99,64
EPI_ISL_8968096	BA.1.1	BA.1.1	BA.1.1_2	QEUH-3294C5D|EPI_ISL_8968096|United Kingdom|BA.1.1|2022-01-14	99,64
EPI_ISL_8968097	BA.1.20	BA.1.15	BA.1.15	BRBR-328BDFC|EPI_ISL_8968097|United Kingdom|BA.1.20|2022-01-14	99,64
EPI_ISL_8969429	BA.1.1	BA.1.1	BA.1.1_2	NEWC-32AD6DB|EPI_ISL_8969429|United Kingdom|BA.1.1|2022-01-15	99,64
EPI_ISL_8969581	BA.1.1	BA.1.1	BA.1.1_3	NEWC-32AB7F8|EPI_ISL_8969581|United Kingdom|BA.1.1|2022-01-15	99,62
EPI_ISL_8972629	BA.1.1	BA.1.1	BA.1.1_3	NEWC-324F644|EPI_ISL_8972629|United Kingdom|BA.1.1|2022-01-12	99,64
EPI_ISL_8973843	BA.1.15	BA.1.15	BA.1.15	MILK-326943B|EPI_ISL_8973843|United Kingdom|BA.1.15|2022-01-13	99,64
EPI_ISL_8974489	BA.1.18	BA.1	BA.1_4	MD-IGS-2108882093|EPI_ISL_8974489|USA|BA.1.18|2021-12-20	90,57
EPI_ISL_8975794	BA.1.17.2	BA.1.17.2	BA.1.17.2	PZH-UMB-7331|EPI_ISL_8975794|Poland|BA.1.17.2|2022-01-05	99,64
EPI_ISL_8976461	BA.1.15	BA.1.15.1	BA.1.15.1	RP-USAFSAM-S10718|EPI_ISL_8976461|Germany|BA.1.15|2021-12-21	99,64
EPI_ISL_8976517	BA.1.15	BA.1.15	BA.1.15	OH-USAFSAM-S10617|EPI_ISL_8976517|USA|BA.1.15|2021-12-30	99,64
EPI_ISL_8976647	BA.1.1	BA.1.1	BA.1.1_2	IL-C22WGS0063|EPI_ISL_8976647|USA|BA.1.1|2022-01-09	99,35
EPI_ISL_8978725	BA.1.1	BA.1.1	BA.1.1_2	CO-CDPHE-2102634830|EPI_ISL_8978725|USA|BA.1.1|2022-01-07	90,98
EPI_ISL_8978729	BA.1.1.18	BA.1.1	BA.1.1_2	CO-CDPHE-2102634146|EPI_ISL_8978729|USA|BA.1.1.18|2022-01-07	90,98
EPI_ISL_8979106	BA.1.20	BA.1	BA.1_4	MN-CDC-IBX180255284099|EPI_ISL_8979106|USA|BA.1.20|2021-12-29	92,39
EPI_ISL_8981181	BA.1.1	BA.1.1	BA.1.1_2	NV-CDC-LC0473633|EPI_ISL_8981181|USA|BA.1.1|2022-01-04	99,64
EPI_ISL_8982791	BA.1.1	BA.1.1	BA.1.1_2	CA-CDC-QDX32670916|EPI_ISL_8982791|USA|BA.1.1|2022-01-06	99,64
EPI_ISL_8984023	BA.1.1	BA.1.1	BA.1.1_2	MA-CDCBI-CRSP_SYMH46VKXOLGBKHA|EPI_ISL_8984023|USA|BA.1.1|2022-01-11	88,30
EPI_ISL_8984182	BA.1.1	BA.1.1	BA.1.1_2	MA-CDCBI-CRSP_YLZ2XQVUTYSNFSP6|EPI_ISL_8984182|USA|BA.1.1|2022-01-11	92,31
EPI_ISL_8984988	BA.1.1	BA.1.1	BA.1.1_2	VT-CDCBI-CRSP_7JQBZ7OBIZ4HS4D4|EPI_ISL_8984988|USA|BA.1.1|2022-01-11	86,05
EPI_ISL_8985406	BA.1	BA.1.15	BA.1.15	Kce-415|EPI_ISL_8985406|Poland|BA.1|2022-01-18	78,11
EPI_ISL_8985943	BA.1.18	BA.1.15	BA.1.15	UZA-UA-CV2324538817|EPI_ISL_8985943|Belgium|BA.1.18|2022-01-01	99,64
EPI_ISL_8986168	BA.1.1	BA.1.1	BA.1.1_3	WA-CDC-UW22010237245|EPI_ISL_8986168|USA|BA.1.1|2022-01-02	91,62
EPI_ISL_8986861	BA.1.1	BA.1.1	BA.1.1_2	ICH-741114762|EPI_ISL_8986861|Israel|BA.1.1|2022-01-13	99,39
EPI_ISL_8988063	BA.1	BA.1.15	BA.1.15	ICH-741113201|EPI_ISL_8988063|Israel|BA.1|2022-01-09	60,33
EPI_ISL_8991441	BA.1	BA.1.15	BA.1.15	PHEC-4P032Z8C|EPI_ISL_8991441|United Kingdom|BA.1|2022	89,52
EPI_ISL_8991500	BA.1.1	BA.1.1	BA.1.1_2	PHEC-4P036Z71|EPI_ISL_8991500|United Kingdom|BA.1.1|2022-01-01	99,37
EPI_ISL_8992546	BA.1	BA.1.15	BA.1.15	PHEC-YYB7RO9|EPI_ISL_8992546|United Kingdom|BA.1|2022-01-04	94,24
EPI_ISL_8992683	BA.1	BA.1.15	BA.1.15	PHEC-YYBH6HX|EPI_ISL_8992683|United Kingdom|BA.1|2022-01-06	99,58
EPI_ISL_8996946	BA.1	BA.1	BA.1_4	QEUH-32C5FA5|EPI_ISL_8996946|United Kingdom|BA.1|2022-01-15	99,64
EPI_ISL_8998684	BA.1	BA.1.15	BA.1.15	QEUH-32CDB9A|EPI_ISL_8998684|United Kingdom|BA.1|2022-01-16	99,64
EPI_ISL_8998942	BA.1	BA.1	BA.1_3	PHWC-PG7IGR|EPI_ISL_8998942|United Kingdom|BA.1|2021-12-30	96,01
EPI_ISL_8999202	BA.1	BA.1	BA.1_3	PHWC-PG7MIX|EPI_ISL_8999202|United Kingdom|BA.1|2022-01-02	96,32
EPI_ISL_8999977	BA.1	BA.1.15	BA.1.15	QEUH-32BE956|EPI_ISL_8999977|United Kingdom|BA.1|2022-01-13	99,64
EPI_ISL_9000255	BA.1	BA.1	BA.1_4	PHWC-PG9YB9|EPI_ISL_9000255|United Kingdom|BA.1|2022-01-02	95,90
EPI_ISL_9001952	BA.1.17.2	BA.1.17.2	BA.1.17.2	MILK-32CE2D0|EPI_ISL_9001952|United Kingdom|BA.1.17.2|2022-01-16	99,64
EPI_ISL_9004347	BA.1	BA.1	BA.1_4	NY-PRL-0110_01A19|EPI_ISL_9004347|USA|BA.1|2022-01-07	99,64
EPI_ISL_9006323	BA.1	BA.1.15	BA.1.15	DCGC-321273|EPI_ISL_9006323|Denmark|BA.1|2022-01-14	99,73
EPI_ISL_9007935	BA.2	A	A_17	DCGC-321907|EPI_ISL_9007935|Denmark|BA.2|2022-01-15	99,81
EPI_ISL_9010390	BA.1	BA.1	BA.1_1	DCGC-323233|EPI_ISL_9010390|Denmark|BA.1|2022-01-14	99,68
EPI_ISL_9010873	BA.1	BA.1	BA.1_1	DCGC-323730|EPI_ISL_9010873|Denmark|BA.1|2022-01-17	94,92
EPI_ISL_9014201	BA.1.17.2	BA.1.15	BA.1.15	LAZ-UCBM-1386|EPI_ISL_9014201|Italy|BA.1.17.2|2021-12-21	95,82
EPI_ISL_9019759	BA.1	BA.1	BA.1_4	PLYM-3301A14|EPI_ISL_9019759|United Kingdom|BA.1|2022-01-17	99,64
EPI_ISL_9019797	BA.1	BA.1	BA.1_2	PLYM-32F98DA|EPI_ISL_9019797|United Kingdom|BA.1|2022-01-17	95,17
EPI_ISL_9020035	BA.1.1	BA.1.1	BA.1.1_3	MILK-32F961C|EPI_ISL_9020035|United Kingdom|BA.1.1|2022-01-17	99,64
EPI_ISL_9020801	BA.1.1	BA.1.1	BA.1.1_2	MILK-32F8EAC|EPI_ISL_9020801|United Kingdom|BA.1.1|2022-01-15	99,64
EPI_ISL_9021703	BA.1	BA.1	BA.1_1	22SNR151_wsserze|EPI_ISL_9021703|Poland|BA.1|2022-01-03	88,84
EPI_ISL_9022063	BA.1	BA.1.15	BA.1.15	MILK-32F9FB7|EPI_ISL_9022063|United Kingdom|BA.1|2022-01-17	99,64
EPI_ISL_9024408	BA.1.1	BA.1.1	BA.1.1_3	BRBR-32FAE9B|EPI_ISL_9024408|United Kingdom|BA.1.1|2022-01-17	99,64
EPI_ISL_9024824	BA.1.17.2	BA.1.17.2	BA.1.17.2	PHWC-P8DRNT|EPI_ISL_9024824|United Kingdom|BA.1.17.2|2022-01-04	95,46
EPI_ISL_9025283	BA.1.17.2	BA.1.17.2	BA.1.17.2	MILK-32F24DD|EPI_ISL_9025283|United Kingdom|BA.1.17.2|2022-01-17	99,64
EPI_ISL_9026455	BA.1	BA.1.15	BA.1.15	ALDP-32BBA83|EPI_ISL_9026455|United Kingdom|BA.1|2022-01-14	99,64
EPI_ISL_9027773	BA.1.16	BA.1.15	BA.1.15	PHWC-P8NZTB|EPI_ISL_9027773|United Kingdom|BA.1.16|2022-01-03	92,02
EPI_ISL_9028546	BA.1.1	BA.1.1	BA.1.1_3	QA-QU-18-33-D3|EPI_ISL_9028546|Qatar|BA.1.1|2021-12-14	99,52
EPI_ISL_9028650	BA.1.17.2	BA.1.17.2	BA.1.17.2	PHWC-P8YJTU|EPI_ISL_9028650|United Kingdom|BA.1.17.2|2021-12-31	96,03
EPI_ISL_9030379	BA.1.1	BA.1.1	BA.1.1_2	PA-CDC-LC0469474|EPI_ISL_9030379|USA|BA.1.1|2021-12-27	99,64
EPI_ISL_9030951	BA.1.15	BA.1	BA.1_4	KS-KHEL-9032|EPI_ISL_9030951|USA|BA.1.15|2022-01-14	99,64
EPI_ISL_9031512	BA.1.1	BA.1.1	BA.1.1_2	ND-NDDH-9733|EPI_ISL_9031512|USA|BA.1.1|2022-01-09	92,50
EPI_ISL_9032794	BA.1	BA.1.15	BA.1.15	PHWC-PG9OJ9|EPI_ISL_9032794|United Kingdom|BA.1|2021-12-27	90,98
EPI_ISL_9034020	BA.1	BA.1.15	BA.1.15	OH-22AM-013CO02115|EPI_ISL_9034020|USA|BA.1|2022-01-14	90,50
EPI_ISL_9034107	BA.1.1	BA.1.1	BA.1.1_3	OH-22AM-018CO00121|EPI_ISL_9034107|USA|BA.1.1|2022-01-18	99,64
EPI_ISL_9034484	BA.1.20	BA.1.15	BA.1.15	IL-NM-17662|EPI_ISL_9034484|USA|BA.1.20|2022-01-12	99,64
EPI_ISL_9035682	BA.1.1	BA.1.1	BA.1.1_3	MD-IGS-142136300627A|EPI_ISL_9035682|USA|BA.1.1|2021-12-29	97,86
EPI_ISL_9037999	BA.1	BA.1	BA.1_4	CA-CDC-LC0469619|EPI_ISL_9037999|USA|BA.1|2022-01-03	99,64
EPI_ISL_9038131	BA.1	BA.1.15	BA.1.15	DE-CDC-LC0461093|EPI_ISL_9038131|USA|BA.1|2022-01-03	92,22
EPI_ISL_9040610	BA.1.1	BA.1.1	BA.1.1_3	VA-CDC-LC0464085|EPI_ISL_9040610|USA|BA.1.1|2022-01-04	99,64
EPI_ISL_9040615	BA.1.1	BA.1.1	BA.1.1_2	AZ-TG1169442|EPI_ISL_9040615|USA|BA.1.1|2022-01-08	99,60
EPI_ISL_9041864	BA.1.1	BA.1.1	BA.1.1_2	CT-CDC-LC0462449|EPI_ISL_9041864|USA|BA.1.1|2022-01-05	99,64
EPI_ISL_9041923	BA.1.15	BA.1	BA.1_4	OH-CDC-LC0466403|EPI_ISL_9041923|USA|BA.1.15|2022-01-06	99,64
EPI_ISL_9042598	BA.1.1	BA.1.1	BA.1.1_2	FL-CDC-STM-JKN5UXDNU|EPI_ISL_9042598|USA|BA.1.1|2022-01-02	99,64
EPI_ISL_9043566	BA.1.1	BA.1.1	BA.1.1_2	FL-CDC-STM-JXXD4WXP2|EPI_ISL_9043566|USA|BA.1.1|2022-01-06	99,64
EPI_ISL_9044069	BA.1.1	BA.1.1	BA.1.1_2	FL-CDC-STM-EQSHMTRKG|EPI_ISL_9044069|USA|BA.1.1|2022-01-09	99,64
EPI_ISL_9044714	BA.1.15	BA.1	BA.1_3	LA-CDC-ASC210561973|EPI_ISL_9044714|USA|BA.1.15|2021-12-30	98,57
EPI_ISL_9044911	BA.1.1.2	BA.1.1	BA.1.1_2	CA-CDC-ASC210561406|EPI_ISL_9044911|USA|BA.1.1.2|2021-12-30	96,64
EPI_ISL_9045348	BA.1	BA.1.15	BA.1.15	NE-CDC-ASC210562146|EPI_ISL_9045348|USA|BA.1|2021-12-30	98,70
EPI_ISL_9045551	BA.1	BA.1.15	BA.1.15	PA-CDC-ASC210561892|EPI_ISL_9045551|USA|BA.1|2021-12-30	99,64
EPI_ISL_9045929	BA.1.20	BA.1	BA.1_4	GA-CDC-MMB12564302|EPI_ISL_9045929|USA|BA.1.20|2021-12-29	93,34
EPI_ISL_9046777	BA.1	BA.1.15	BA.1.15	NY-CDC-ASC210505516|EPI_ISL_9046777|USA|BA.1|2022-01-02	99,60
EPI_ISL_9048106	BA.1	BA.1.15	BA.1.15	AG-ETHZ-35536717|EPI_ISL_9048106|Switzerland|BA.1|2022-01-03	99,64
EPI_ISL_9048761	BA.1.1	BA.1.1	BA.1.1_2	BL-ETHZ-35720511|EPI_ISL_9048761|Switzerland|BA.1.1|2022-01-12	99,64
EPI_ISL_9052485	BA.1.1	BA.1.1	BA.1.1_2	OR-CDC-LC0471450|EPI_ISL_9052485|USA|BA.1.1|2022-01-04	99,64
EPI_ISL_9052828	BA.1.1	BA.1.1	BA.1.1_2	CA-CDC-LC0470618|EPI_ISL_9052828|USA|BA.1.1|2022-01-07	90,92
EPI_ISL_9061511	BA.1	BA.1	BA.1_3	BS-ETHZ-35602047|EPI_ISL_9061511|Switzerland|BA.1|2022-01-06	99,64

EPI_ISL_9063729	BA.2	A	A_17	DCGC-324666|EPI_ISL_9063729|Denmark|BA.2|2022-01-18	99,79
EPI_ISL_9064452	BA.2	A	A_17	MILK-332260A|EPI_ISL_9064452|United Kingdom|BA.2|2022-01-18	99,81
EPI_ISL_9066716	BA.1.1	BA.1.1	BA.1.1_3	NEWC-330A082|EPI_ISL_9066716|United Kingdom|BA.1.1|2022-01-17	99,62
EPI_ISL_9067130	BA.2	A	A_17	DCGC-325891|EPI_ISL_9067130|Denmark|BA.2|2022-01-17	99,81
EPI_ISL_9067147	BA.1.1	BA.1.1	BA.1.1_3	HSLL-3305F20|EPI_ISL_9067147|United Kingdom|BA.1.1|2022-01-16	99,64
EPI_ISL_9067302	BA.1	BA.1.15	BA.1.15	DCGC-325971|EPI_ISL_9067302|Denmark|BA.1|2022-01-12	99,64
EPI_ISL_9068575	BA.2.9	A	A_17	DCGC-326418|EPI_ISL_9068575|Denmark|BA.2.9|2022-01-17	99,81
EPI_ISL_9069951	BA.1.1	BA.1.1	BA.1.1_3	MILK-3324398|EPI_ISL_9069951|United Kingdom|BA.1.1|2022-01-17	99,64
EPI_ISL_9073417	BA.1	BA.1	BA.1_4	HSGM-F16553|EPI_ISL_9073417|Turkey|BA.1|2022-01-10	99,58
EPI_ISL_9074014	BA.1	BA.1	BA.1_4	NEWC-3305403|EPI_ISL_9074014|United Kingdom|BA.1|2022-01-17	99,64
EPI_ISL_9077412	BA.1	BA.1	BA.1_4	ALDP-330D887|EPI_ISL_9077412|United Kingdom|BA.1|2022-01-17	99,64
EPI_ISL_9077963	BA.1.17	BA.1	BA.1_3	CL-COV21862|EPI_ISL_9077963|Spain|BA.1.17|2021-12-14	94,45
EPI_ISL_9080409	BA.1.1	BA.1.1	BA.1.1_2	NW-RKI-I-444277|EPI_ISL_9080409|Germany|BA.1.1|2022-01-08	99,29
EPI_ISL_9083891	BA.1.18	BA.1.15	BA.1.15	IDF-HMN-22012110179|EPI_ISL_9083891|France|BA.1.18|2022-01-04	92,98
EPI_ISL_9090030	BA.1	BA.1.15	BA.1.15	AK-PHL16886|EPI_ISL_9090030|USA|BA.1|2022-01-10	92,90
EPI_ISL_9091766	BA.1.1.18	BA.1.1	BA.1.1_2	NC-ECU-CORVASEQ-111650328|EPI_ISL_9091766|USA|BA.1.1.18|2022-01-13	99,62
EPI_ISL_9101414	BA.1.1	BA.1.1	BA.1.1_2	MA-CDCBI-CRSP_6XYPZ7MLZW624YNY|EPI_ISL_9101414|USA|BA.1.1|2022-01-13	93,02
EPI_ISL_9102006	BA.1.1	BA.1	BA.1_1	MA-CDCBI-CRSP_SKFVEHQL6ZI5GTBO|EPI_ISL_9102006|USA|BA.1.1|2022-01-14	91,47
EPI_ISL_9102900	BA.1	BA.1.1	BA.1.1_2	UT-UPHL-220121483203|EPI_ISL_9102900|USA|BA.1|2021-12-23	99,64
EPI_ISL_9103449	BA.1.17	BA.1	BA.1_3	CT-HUGTiPM072UY8G9|EPI_ISL_9103449|Spain|BA.1.17|2022-01-14	96,64
EPI_ISL_9103520	BA.1.17.2	BA.1	BA.1_4	BW-RKI-I-447695|EPI_ISL_9103520|Germany|BA.1.17.2|2022-01-05	90,44
EPI_ISL_9104609	BA.1.1	BA.1.1	BA.1.1_2	UT-UPHL-220121773336|EPI_ISL_9104609|USA|BA.1.1|2022-01-12	99,64
EPI_ISL_9105696	BA.1.17.2	BA.1.17.2	BA.1.17.2	SN-RKI-I-449646|EPI_ISL_9105696|Germany|BA.1.17.2|2021-12-30	99,64
EPI_ISL_9107695	BA.1.1	BA.1.1	BA.1.1_2	BW-RKI-I-453138|EPI_ISL_9107695|Germany|BA.1.1|2022-01-11	99,62
EPI_ISL_9110692	BA.1.1	BA.1.1	BA.1.1_3	BB-RKI-I-456305|EPI_ISL_9110692|Germany|BA.1.1|2022-01-14	99,64
EPI_ISL_9110719	BA.1	BA.1	BA.1_4	BB-RKI-I-456316|EPI_ISL_9110719|Germany|BA.1|2022-01-14	99,64
EPI_ISL_9111817	BA.1.17.2	BA.1.17.2	BA.1.17.2	PZH-GUM-7113|EPI_ISL_9111817|Poland|BA.1.17.2|2022-01-14	99,64
EPI_ISL_9112121	BA.1	BA.1	BA.1_3	NEWC-334BF5B|EPI_ISL_9112121|United Kingdom|BA.1|2022-01-18	96,03
EPI_ISL_9112338	BA.1.17.2	BA.1.17.2	BA.1.17.2	ALDP-3352689|EPI_ISL_9112338|United Kingdom|BA.1.17.2|2022-01-19	99,64
EPI_ISL_9112752	BA.1.15	BA.1	BA.1_1	TX-HHD-2201145171|EPI_ISL_9112752|USA|BA.1.15|2021-12-21	91,51
EPI_ISL_9113097	BA.1.15	BA.1	BA.1_1	TX-HHD-2201058686|EPI_ISL_9113097|USA|BA.1.15|2021-12-22	94,83
EPI_ISL_9113136	BA.1.15	BA.1	BA.1_1	TX-HHD-2201119039|EPI_ISL_9113136|USA|BA.1.15|2021-12-23	91,11
EPI_ISL_9115295	BA.1.16	BA.1.15	BA.1.15	ALDP-33497EC|EPI_ISL_9115295|United Kingdom|BA.1.16|2022-01-18	99,64
EPI_ISL_9115356	BA.1.1	BA.1.1	BA.1.1_2	NORT-YB14FS|EPI_ISL_9115356|United Kingdom|BA.1.1|2022	90,98
EPI_ISL_9115969	BA.1	BA.1	BA.1_3	NORT-YB1GKY|EPI_ISL_9115969|United Kingdom|BA.1|2022-01-03	96,03
EPI_ISL_9117605	BA.1.1	BA.1.1	BA.1.1_3	ALDP-333632A|EPI_ISL_9117605|United Kingdom|BA.1.1|2022-01-18	99,62
EPI_ISL_9118705	BA.1	BA.1.15	BA.1.15	ALDP-33375BA|EPI_ISL_9118705|United Kingdom|BA.1|2022-01-19	99,64
EPI_ISL_9119480	BA.1.15	BA.1.15	BA.1.15	NEWC-333DAA6|EPI_ISL_9119480|United Kingdom|BA.1.15|2022-01-18	99,64
EPI_ISL_9119497	BA.1.17.2	BA.1.17.2	BA.1.17.2	BRBR-333F400|EPI_ISL_9119497|United Kingdom|BA.1.17.2|2022-01-19	99,64
EPI_ISL_9121679	BA.1.1.1	BA.1	BA.1_4	WSSEGorzow-22S0381|EPI_ISL_9121679|Poland|BA.1.1.1|2022-01-10	81,59
EPI_ISL_9124067	BA.1	BA.1	BA.1_3	ALDP-333E7CC|EPI_ISL_9124067|United Kingdom|BA.1|2022-01-18	95,55
EPI_ISL_9124216	BA.1.17.2	BA.1.17.2	BA.1.17.2	MILK-33180BE|EPI_ISL_9124216|United Kingdom|BA.1.17.2|2022-01-18	99,64
EPI_ISL_9124832	BA.2	BA.2	BA.2_1	DCGC-330176|EPI_ISL_9124832|Denmark|BA.2|2022-01-19	99,81
EPI_ISL_9124859	BA.2	BA.2	BA.2_1	DCGC-330186|EPI_ISL_9124859|Denmark|BA.2|2022-01-18	99,81
EPI_ISL_9124892	BA.1.1	BA.1.1	BA.1.1_2	NEWC-332F5D3|EPI_ISL_9124892|United Kingdom|BA.1.1|2022-01-17	99,64
EPI_ISL_9125399	BA.1.1	BA.1.1	BA.1.1_3	ALDP-33247E7|EPI_ISL_9125399|United Kingdom|BA.1.1|2022-01-17	99,64
EPI_ISL_9126906	BA.1.18	BA.1.15	BA.1.15	MILK-331AFEC|EPI_ISL_9126906|United Kingdom|BA.1.18|2022-01-18	99,64
EPI_ISL_9128371	BA.2.9	A	A_17	DCGC-331007|EPI_ISL_9128371|Denmark|BA.2.9|2022-01-14	99,81
EPI_ISL_9128392	BA.1.17.2	BA.1.17.2	BA.1.17.2	ALDP-333CE32|EPI_ISL_9128392|United Kingdom|BA.1.17.2|2022-01-18	99,64
EPI_ISL_9128778	BA.1	BA.1	BA.1_1	DCGC-331177|EPI_ISL_9128778|Denmark|BA.1|2022-01-12	99,66
EPI_ISL_9128940	BA.1	BA.1.15	BA.1.15	MILK-332FB4A|EPI_ISL_9128940|United Kingdom|BA.1|2022-01-18	99,64
EPI_ISL_9133065	BA.1.1	BA.1	BA.1.1_2	NY-SUNYQBR-61210501720454|EPI_ISL_9133065|USA|BA.1.1|2021-12-31	99,16
EPI_ISL_9133248	BA.1	BA.1.15	BA.1.15	NY-SUNYQBR-61210550502295|EPI_ISL_9133248|USA|BA.1|2021-12-19	97,50
EPI_ISL_9134732	BA.1.1	BA.1.1	BA.1.1_3	NC-CHN-01005793|EPI_ISL_9134732|Spain|BA.1.1|2021-12-14	96,70
EPI_ISL_9135930	BA.1	BA.1.15	BA.1.15	ON-PHL-22-00336|EPI_ISL_9135930|Canada|BA.1|2021-12-13	90,98
EPI_ISL_9135969	BA.1	BA.1.15	BA.1.15	ON-PHL-22-00375|EPI_ISL_9135969|Canada|BA.1|2021-12-14	93,63
EPI_ISL_9137783	BA.1.1	BA.1.1	BA.1.1_2	ON-PHL-22-02418|EPI_ISL_9137783|Canada|BA.1.1|2022-01-06	95,67
EPI_ISL_9139256	BA.1	BA.2.10	BA.2.10	SU-NHLS_3829|EPI_ISL_9139256|South Africa|BA.1|2022-01-12	62,45
EPI_ISL_9140004	BA.1.1	BA.1.1	BA.1.1_3	TX-CDC-ASC210581628|EPI_ISL_9140004|USA|BA.1.1|2022-01-10	91,55
EPI_ISL_9141059	BA.1.1	BA.1.1	BA.1.1_2	CA-CDC-FG-238355|EPI_ISL_9141059|USA|BA.1.1|2022-01-15	91,11
EPI_ISL_9142287	BA.1	BA.1.15	BA.1.15	CA-CDC-FG-240389|EPI_ISL_9142287|USA|BA.1|2022-01-17	97,08
EPI_ISL_9142392	BA.1.1	BA.1.1	BA.1.1_3	NC-SLPH-0973|EPI_ISL_9142392|USA|BA.1.1|2022-01-06	93,05
EPI_ISL_9143379	BA.1.1.18	BA.1.1	BA.1.1_3	NE-NPHL22-7532|EPI_ISL_9143379|USA|BA.1.1.18|2022-01-21	84,53
EPI_ISL_9144986	BA.1.1	BA.1.1	BA.1.1_2	WA-PHL-010621|EPI_ISL_9144986|USA|BA.1.1|2022-01-03	98,02
EPI_ISL_9146577	BA.1.1	BA.1.1	BA.1.1_2	AZ-ASU36830|EPI_ISL_9146577|USA|BA.1.1|2022-01-18	91,13
EPI_ISL_9147277	BA.1	BA.1	BA.1_4	AR-UMGC-32191|EPI_ISL_9147277|USA|BA.1|2022-01-05	79,97
EPI_ISL_9149685	BA.1.1	BA.1.1	BA.1.1_3	MN-CDC-QDX32751286|EPI_ISL_9149685|USA|BA.1.1|2022-01-08	99,64
EPI_ISL_9152663	BA.1.15	BA.1.15	BA.1.15	AZ-TG1170265|EPI_ISL_9152663|USA|BA.1.15|2021-12-27	99,31
EPI_ISL_9152981	BA.1.17.2	BA.1.17.2	BA.1.17.2	ID-IBL-785185|EPI_ISL_9152981|USA|BA.1.17.2|2022-01-18	99,60
EPI_ISL_9154222	BA.1.1	BA.1.1	BA.1.1_2	PZH-UMB-7695|EPI_ISL_9154222|Poland|BA.1.1|2022-01-10	99,64
EPI_ISL_9154930	BA.1.1.11	BA.1.1	BA.1.1_3	PZH-UMB-8411|EPI_ISL_9154930|Poland|BA.1.1.11|2022-01-07	96,87
EPI_ISL_9155719	BA.1	BA.1.15	BA.1.15	OCC-HCL022006086201|EPI_ISL_9155719|France|BA.1|2022-01-02	92,81
EPI_ISL_9155896	BA.1	BA.1.15	BA.1.15	NAQ-HCL022006636401|EPI_ISL_9155896|France|BA.1|2022-01-03	91,24
EPI_ISL_9156337	BA.1.15	BA.1.1	BA.1.1_2	CO-197233|EPI_ISL_9156337|Chile|BA.1.15|2021-12-22	99,62
EPI_ISL_9156942	BA.1.17.2	BA.1.17.2	BA.1.17.2	2201-904_wsselodz|EPI_ISL_9156942|Poland|BA.1.17.2|2022-01-05	93,99
EPI_ISL_9158856	BA.1.1	BA.1.1	BA.1.1_3	PHEC-4Q03DZ21|EPI_ISL_9158856|United Kingdom|BA.1.1|2022	99,24
EPI_ISL_9159429	BA.1.15	BA.1	BA.1_3	PHEC-YYB9D6Y|EPI_ISL_9159429|United Kingdom|BA.1.15|2022-01-03	97,48
EPI_ISL_9161299	BA.1.15	BA.1.15	BA.1.15	RIMD04404|EPI_ISL_9161299|Japan|BA.1.15|2022-01-07	99,64
EPI_ISL_9162358	BA.2	A	A_17	DCGC-331308|EPI_ISL_9162358|Denmark|BA.2|2022-01-20	99,81
EPI_ISL_9163528	BA.2.9	A	A_17	DCGC-331844|EPI_ISL_9163528|Denmark|BA.2.9|2022-01-21	99,75
EPI_ISL_9164207	BA.1.17.2	BA.1.17.2	BA.1.17.2	PHWC-P8FTTB|EPI_ISL_9164207|United Kingdom|BA.1.17.2|2022-01-06	95,94
EPI_ISL_9165200	BA.1	BA.1	BA.1_3	PHWC-P8GF1J|EPI_ISL_9165200|United Kingdom|BA.1|2022	96,03
EPI_ISL_9165218	BA.1.17.2	BA.1.17.2	BA.1.17.2	PHWC-P8GFID|EPI_ISL_9165218|United Kingdom|BA.1.17.2|2022-01-04	96,03
EPI_ISL_9165623	BA.1.1.14	BA.1.1	BA.1.1_2	PHWC-P8GN7S|EPI_ISL_9165623|United Kingdom|BA.1.1.14|2022-01-04	96,03
EPI_ISL_9168012	BA.1	BA.1	BA.1_4	DCGC-333770|EPI_ISL_9168012|Denmark|BA.1|2022-01-19	99,64
EPI_ISL_9169375	BA.2	A	A_17	DCGC-334770|EPI_ISL_9169375|Denmark|BA.2|2022-01-19	99,96
EPI_ISL_9171490	BA.1.15	BA.1	BA.1_4	INC-2706-729637|EPI_ISL_9171490|Costa Rica|BA.1.15|2022-01-06	99,64
EPI_ISL_9171989	BA.1	BA.1	BA.1_4	PLYM-335EF37|EPI_ISL_9171989|United Kingdom|BA.1|2022-01-20	99,64
EPI_ISL_9173705	BA.1.15	BA.1.15	BA.1.15	ALDP-335E641|EPI_ISL_9173705|United Kingdom|BA.1.15|2022-01-20	99,64
EPI_ISL_9174878	BA.1	BA.1	BA.1_4	QEUH-336C103|EPI_ISL_9174878|United Kingdom|BA.1|2022-01-20	99,64
EPI_ISL_9175511	BA.1	BA.1	BA.1_4	MILK-3357912|EPI_ISL_9175511|United Kingdom|BA.1|2022-01-17	99,64
EPI_ISL_9176465	BA.1.15	BA.1.15	BA.1.15	MILK-335A742|EPI_ISL_9176465|United Kingdom|BA.1.15|2022-01-21	99,64
EPI_ISL_9178055	BA.1.1.13	BA.1.1	BA.1.1_3	MILK-3355729|EPI_ISL_9178055|United Kingdom|BA.1.1.13|2022-01-19	99,64
EPI_ISL_9178691	BA.1.1.18	BA.1.1	BA.1.1_3	NE-CDC-LC0483167|EPI_ISL_9178691|USA|BA.1.1.18|2022-01-04	99,64
EPI_ISL_9178737	BA.1.20	BA.1	BA.1_3	AZ-CDC-LC0481592|EPI_ISL_9178737|USA|BA.1.20|2022-01-04	99,58
EPI_ISL_9179851	BA.1.20	BA.1.15	BA.1.15	AK-CDC-LC0482294|EPI_ISL_9179851|USA|BA.1.20|2022-01-06	99,64
EPI_ISL_9179883	BA.1.1.1	BA.1.1	BA.1.1_3	AZ-CDC-LC0484663|EPI_ISL_9179883|USA|BA.1.1.1|2022-01-06	99,64
EPI_ISL_9180805	BA.1.1	BA.1.1	BA.1.1_2	OR-CDC-LC0482724|EPI_ISL_9180805|USA|BA.1.1|2022-01-07	99,64
EPI_ISL_9180987	BA.1.1.18	BA.1.1	BA.1.1_3	IL-S22WGS522|EPI_ISL_9180987|USA|BA.1.1.18|2022-01-18	99,45
EPI_ISL_9183069	BA.1.1.18	BA.1.1	BA.1.1_2	CO-CDPHE-2102664309|EPI_ISL_9183069|USA|BA.1.1.18|2022-01-11	90,98
EPI_ISL_9183854	BA.1.1.18	BA.1.1	BA.1.1_2	CO-CDPHE-2102620466|EPI_ISL_9183854|USA|BA.1.1.18|2021-12-29	90,98

EPI_ISL_9183928	BA.1.1	BA.1.1	BA.1.1_2	CO-CDPHE-2102657748|EPI_ISL_9183928|USA|BA.1.1|2021-12-30	90,98
EPI_ISL_9184591	BA.1.15	BA.1.15	BA.1.15	LI-198679|EPI_ISL_9184591|Chile|BA.1.15|2021-12-27	99,60
EPI_ISL_9185298	BA.1.1	BA.1.1	BA.1.1_3	LI-RIVM-83389|EPI_ISL_9185298|Netherlands|BA.1.1|2022-01-14	94,87
EPI_ISL_9186508	BA.1.15	BA.1.15	BA.1.15	ARA-HMN-22012110178|EPI_ISL_9186508|France|BA.1.15|2021-12-27	79,30
EPI_ISL_9186558	BA.1.1.1	BA.1.1	BA.1.1_3	GES-HMN-22012130556|EPI_ISL_9186558|France|BA.1.1.1|2022-01-10	87,62
EPI_ISL_9186877	BA.1.20	BA.1.15	BA.1.15	WA-CDC-LC0484273|EPI_ISL_9186877|USA|BA.1.20|2022-01-10	99,64
EPI_ISL_9187860	BA.1.15	BA.1.15	BA.1.15	MS-UMMC-M221D4-528179|EPI_ISL_9187860|USA|BA.1.15|2022-01-11	94,85
EPI_ISL_9188813	BA.1.1	BA.1.1	BA.1.1_2	TN-CDC-LC0478432|EPI_ISL_9188813|USA|BA.1.1|2022-01-11	99,64
EPI_ISL_9189936	BA.1	BA.1	BA.1_3	BI-3715|EPI_ISL_9189936|Chile|BA.1|2022-01-06	84,72
EPI_ISL_9190512	BA.1.1	BA.1.1	BA.1.1_2	NJ-CDC-LC0479629|EPI_ISL_9190512|USA|BA.1.1|2022-01-11	99,64
EPI_ISL_9191112	BA.1	BA.1	BA.1_4	ML-1692|EPI_ISL_9191112|Chile|BA.1|2022-01-03	99,62
EPI_ISL_9192040	BA.1.1	BA.1.1	BA.1.1_2	INEI117300|EPI_ISL_9192040|Argentina|BA.1.1|2021-12-29	99,64
EPI_ISL_9192779	BA.1	BA.1	BA.1_4	NY-CDC-LC0479653|EPI_ISL_9192779|USA|BA.1|2022-01-12	99,64
EPI_ISL_9193985	BA.1.1	BA.1.1	BA.1.1_3	GA-CDC-LC0478281|EPI_ISL_9193985|USA|BA.1.1|2022-01-12	99,64
EPI_ISL_9197947	BA.1.15	BA.1	BA.1_3	TX-HMH-MCoV-70408|EPI_ISL_9197947|USA|BA.1.15|2021-12-19	99,64
EPI_ISL_9197991	BA.1.15	BA.1.15	BA.1.15	TX-HMH-MCoV-70452|EPI_ISL_9197991|USA|BA.1.15|2021-12-19	99,64
EPI_ISL_9198777	BA.1.15	BA.1.15	BA.1.15	TX-HMH-MCoV-71235|EPI_ISL_9198777|USA|BA.1.15|2021-12-20	95,94
EPI_ISL_9198976	BA.1.15	BA.1	BA.1_3	TX-HMH-MCoV-71434|EPI_ISL_9198976|USA|BA.1.15|2021-12-21	96,53
EPI_ISL_9199313	BA.1.15	BA.1	BA.1_3	TX-HMH-MCoV-71771|EPI_ISL_9199313|USA|BA.1.15|2021-12-22	97,33
EPI_ISL_9200455	BA.1.1	BA.1.1	BA.1.1_2	TX-HMH-MCoV-73517|EPI_ISL_9200455|USA|BA.1.1|2022-01-01	96,99
EPI_ISL_9200747	BA.1.15	BA.1.15	BA.1.15	TX-HMH-MCoV-73979|EPI_ISL_9200747|USA|BA.1.15|2022-01-03	99,64
EPI_ISL_9203732	BA.1.15	BA.1.15	BA.1.15	TX-HMH-MCoV-74487|EPI_ISL_9203732|USA|BA.1.15|2022-01-03	99,47
EPI_ISL_9205129	BA.1.15	BA.1	BA.1_3	TX-HMH-MCoV-75837|EPI_ISL_9205129|USA|BA.1.15|2022-01-05	96,53
EPI_ISL_9206498	BA.1.1	BA.1.1	BA.1.1_2	SK-RRPL-322132|EPI_ISL_9206498|Canada|BA.1.1|2022-01-03	99,64
EPI_ISL_9206514	BA.1.1.18	BA.1.1	BA.1.1_2	SK-RRPL-321933|EPI_ISL_9206514|Canada|BA.1.1.18|2022-01-03	95,99
EPI_ISL_9209477	BA.1.1.2	BA.1.1	BA.1.1_3	PG-179879|EPI_ISL_9209477|Japan|BA.1.1.2|2022-01-07	99,64
EPI_ISL_9210472	BA.1.1.2	BA.1.1	BA.1.1_3	PG-180196|EPI_ISL_9210472|Japan|BA.1.1.2|2022-01-07	99,64
EPI_ISL_9210782	BA.1.18	BA.2	BA.2_1	HE-RKI-I-458879|EPI_ISL_9210782|Germany|BA.1.18|2022-01-15	93,00
EPI_ISL_9211138	BA.1.1.2	BA.1.1	BA.1.1_2	PG-180426|EPI_ISL_9211138|Japan|BA.1.1.2|2022-01-07	99,64
EPI_ISL_9211439	BA.1.17	BA.1.15	BA.1.15	BW-RKI-I-459257|EPI_ISL_9211439|Germany|BA.1.17|2022-01-05	99,64
EPI_ISL_9211726	BA.1.1	BA.1.1	BA.1.1_2	HH-RKI-I-459441|EPI_ISL_9211726|Germany|BA.1.1|2022-01-10	99,64
EPI_ISL_9213200	BA.1.15	BA.1	BA.1_4	WA-Curative-128166|EPI_ISL_9213200|USA|BA.1.15|2021-12-23	96,43
EPI_ISL_9214328	BA.1	BA.1.15	BA.1.15	NI-RKI-I-461725|EPI_ISL_9214328|Germany|BA.1|2022-01-12	87,39
EPI_ISL_9215430	BA.1	BA.1.15	BA.1.15	MD-Curative-101198|EPI_ISL_9215430|USA|BA.1|2021-12-17	71,34
EPI_ISL_9216074	BA.1.1	BA.1.1	BA.1.1_2	BW-RKI-I-462503|EPI_ISL_9216074|Germany|BA.1.1|2022-01-04	99,43
EPI_ISL_9216509	BA.1.1.1	BA.1.1	BA.1.1_3	BW-RKI-I-462804|EPI_ISL_9216509|Germany|BA.1.1.1|2022-01-06	99,64
EPI_ISL_9217775	BA.1.17.2	BA.1.15	BA.1.15	TH-RKI-I-464594|EPI_ISL_9217775|Germany|BA.1.17.2|2022-01-17	90,08
EPI_ISL_9218168	BA.1.1.2	BA.1.1	BA.1.1_3	PG-177666|EPI_ISL_9218168|Japan|BA.1.1.2|2022-01-07	99,64
EPI_ISL_9223485	BA.1.15	BA.1	BA.1_4	PG-175274|EPI_ISL_9223485|Japan|BA.1.15|2021-12-31	99,64
EPI_ISL_9223978	BA.1.1	BA.1.1	BA.1.1_2	BB-RKI-I-470074|EPI_ISL_9223978|Germany|BA.1.1|2022-01-20	99,64
EPI_ISL_9227129	BA.1.1	BA.1.1	BA.1.1_3	34624|EPI_ISL_9227129|Norway|BA.1.1|2021-12-29	99,52
EPI_ISL_9230280	BA.1.1	BA.1.1	BA.1.1_2	PHEC-4Q0B3Z45|EPI_ISL_9230280|United Kingdom|BA.1.1|2022	99,64
EPI_ISL_9231039	BA.1	BA.1	BA.1_1	BY-RKI-I-473897|EPI_ISL_9231039|Germany|BA.1|2022-01-13	94,71
EPI_ISL_9234748	BC.1	BA.1.1	BA.1.1_2	PG-175796|EPI_ISL_9234748|Japan|BC.1|2021-12-31	99,64
EPI_ISL_9235175	BA.1	BA.1.15	BA.1.15	PHEP-YYD16HB|EPI_ISL_9235175|United Kingdom|BA.1|2022-01-02	79,20
EPI_ISL_9235929	BA.1.1	BA.1.1	BA.1.1_2	PHWC-P884FP|EPI_ISL_9235929|United Kingdom|BA.1.1|2022-01-10	96,03
EPI_ISL_9237096	BA.2	A	A_17	DCGC-335247|EPI_ISL_9237096|Denmark|BA.2|2022-01-22	99,81
EPI_ISL_9237794	BA.1.15	BA.1	BA.1_1	MA-CDCBI-CRSP_6IRJNFSAJSMCAF2X|EPI_ISL_9237794|USA|BA.1.15|2022-01-16	88,84
EPI_ISL_9238195	BA.2	A	A_17	DCGC-335476|EPI_ISL_9238195|Denmark|BA.2|2022-01-22	99,31
EPI_ISL_9238767	BA.1.1.8	BA.1	BA.1_1	MA-CDCBI-CRSP_I3REGG77RAOLCX3E|EPI_ISL_9238767|USA|BA.1.1.8|2022-01-17	89,93
EPI_ISL_9238978	BA.1.1	BA.1.1	BA.1.1_2	PHWC-P8E7UM|EPI_ISL_9238978|United Kingdom|BA.1.1|2022-01-07	96,03
EPI_ISL_9239639	BA.1.17.2	BA.1.17.2	BA.1.17.2	MA-CDCBI-CRSP_NTWBH6TGJF2KNZRS|EPI_ISL_9239639|USA|BA.1.17.2|2022-01-12	88,93
EPI_ISL_9240079	BA.1.1	BA.1	BA.1_1	MA-CDCBI-CRSP_TKBDU23N7BGIL36Q|EPI_ISL_9240079|USA|BA.1.1|2022-01-15	87,29
EPI_ISL_9240801	BA.1.1	BA.1.1	BA.1.1_2	NH-CDCBI-CRSP_FLIRZE2LXE5S7HOE|EPI_ISL_9240801|USA|BA.1.1|2022-01-15	86,13
EPI_ISL_9241905	BA.1	BA.1	BA.1_3	PHWC-P8EUSX|EPI_ISL_9241905|United Kingdom|BA.1|2022-01-07	96,03
EPI_ISL_9243291	BA.1	BA.1	BA.1_3	PHWC-P8JQEX|EPI_ISL_9243291|United Kingdom|BA.1|2022-01-08	96,03
EPI_ISL_9246217	BA.1.1	BA.1.1	BA.1.1_3	BE-IFIK-6170-0233|EPI_ISL_9246217|Switzerland|BA.1.1|2022-01-23	99,64
EPI_ISL_9246893	BA.1.15.1	BA.1.15	BA.1.15	QEUH-33A2DB2|EPI_ISL_9246893|United Kingdom|BA.1.15.1|2022-01-20	96,03
EPI_ISL_9250715	BA.1.1.15	BA.1.1	BA.1.1_2	MILK-337981D|EPI_ISL_9250715|United Kingdom|BA.1.1.15|2022-01-20	99,64
EPI_ISL_9251287	BA.1.1.15	BA.1.1	BA.1.1_3	MILK-3390E5A|EPI_ISL_9251287|United Kingdom|BA.1.1.15|2022-01-21	99,64
EPI_ISL_9251434	BA.1	BA.1.15	BA.1.15	MILK-3374BA0|EPI_ISL_9251434|United Kingdom|BA.1|2022-01-17	99,64
EPI_ISL_9251634	BA.1.17.2	BA.1.17.2	BA.1.17.2	BRBR-3375E88|EPI_ISL_9251634|United Kingdom|BA.1.17.2|2022-01-19	99,64
EPI_ISL_9253694	BA.1	BA.1.15	BA.1.15	ALDP-338BE61|EPI_ISL_9253694|United Kingdom|BA.1|2022-01-19	99,64
EPI_ISL_9255073	BA.1.17.2	BA.1.17.2	BA.1.17.2	ALDP-337EA6D|EPI_ISL_9255073|United Kingdom|BA.1.17.2|2022-01-20	99,64
EPI_ISL_9255166	BA.1.1	BA.1.1	BA.1.1_3	ALDP-337E9D9|EPI_ISL_9255166|United Kingdom|BA.1.1|2022-01-20	99,64
EPI_ISL_9255482	BA.2	BA.2	BA.2_1	ALDP-33A0200|EPI_ISL_9255482|United Kingdom|BA.2|2022-01-20	94,09
EPI_ISL_9255737	BA.1.1	BA.1.1	BA.1.1_2	ALDP-339F0BF|EPI_ISL_9255737|United Kingdom|BA.1.1|2022-01-21	99,64
EPI_ISL_9256479	BA.1	BA.1	BA.1_4	MILK-3389847|EPI_ISL_9256479|United Kingdom|BA.1|2022-01-21	99,64
EPI_ISL_9256720	BA.1.15.1	BA.1.15.1	BA.1.15.1	MILK-3388D85|EPI_ISL_9256720|United Kingdom|BA.1.15.1|2022-01-21	99,64
EPI_ISL_9257531	BA.1.18	BA.1.15	BA.1.15	BE-SRO-500006_9573|EPI_ISL_9257531|Switzerland|BA.1.18|2022-01-12	99,64
EPI_ISL_9257930	BA.1.1	BA.1.1	BA.1.1_2	IDF-CERBAHC-12135911|EPI_ISL_9257930|France|BA.1.1|2021-12-20	92,46
EPI_ISL_9258233	BA.1.1.18	BA.1.1	BA.1.1_2	CO-CDPHE-2102614652|EPI_ISL_9258233|USA|BA.1.1.18|2022-01-05	90,98
EPI_ISL_9264202	BA.1.1	BA.1.1	BA.1.1_3	ME-HETL-J11771|EPI_ISL_9264202|USA|BA.1.1|2022-01-10	99,64
EPI_ISL_9267060	BA.1.1	BA.1.1	BA.1.1_2	PZH-GUM-7461|EPI_ISL_9267060|Poland|BA.1.1|2022-01-12	99,64
EPI_ISL_9268133	BA.1.1	BA.1.1	BA.1.1_3	PHEP-YYD1C4Z|EPI_ISL_9268133|United Kingdom|BA.1.1|2022-01-03	90,65
EPI_ISL_9270664	BA.1	BA.1	BA.1_1	DCGC-338651|EPI_ISL_9270664|Denmark|BA.1|2022-01-18	99,68
EPI_ISL_9273027	BA.1	BA.1	BA.1_1	CA-CDPH-3000296204|EPI_ISL_9273027|USA|BA.1|2021-12-28	91,55
EPI_ISL_9274688	BA.1.1	BA.1.1	BA.1.1_3	JK-GS-GSILab-879744|EPI_ISL_9274688|Indonesia|BA.1.1|2022-01-19	99,64
EPI_ISL_9274734	BA.1.15	BA.1.15	BA.1.15	JK-GS-GSILab-876136|EPI_ISL_9274734|Indonesia|BA.1.15|2022-01-17	99,64
EPI_ISL_9275938	BA.1.15	BA.1.15	BA.1.15	BRBR-33728AB|EPI_ISL_9275938|United Kingdom|BA.1.15|2022-01-20	91,57
EPI_ISL_9276633	BA.1.1.12	BA.1.1	BA.1.1_3	MILK-33ADF66|EPI_ISL_9276633|United Kingdom|BA.1.1.12|2022-01-22	99,64
EPI_ISL_9276637	BA.1.17.2	BA.1.17.2	BA.1.17.2	ALDP-33AE8D3|EPI_ISL_9276637|United Kingdom|BA.1.17.2|2022-01-21	99,64
EPI_ISL_9277145	BA.1.17.2	BA.1.17.2	BA.1.17.2	PLYM-33B9750|EPI_ISL_9277145|United Kingdom|BA.1.17.2|2022-01-22	99,64
EPI_ISL_9280841	BA.1	BA.1.15	BA.1.15	MILK-339D28E|EPI_ISL_9280841|United Kingdom|BA.1|2022-01-21	99,54
EPI_ISL_9280908	BA.1.16	BA.1.15	BA.1.15	BRBR-339BC55|EPI_ISL_9280908|United Kingdom|BA.1.16|2022-01-21	99,64
EPI_ISL_9282016	BA.1	BA.1.15	BA.1.15	BRBR-33AA057|EPI_ISL_9282016|United Kingdom|BA.1|2022-01-21	99,64
EPI_ISL_9283862	BA.1.17.2	BA.1.17.2	BA.1.17.2	ALDP-33AB28D|EPI_ISL_9283862|United Kingdom|BA.1.17.2|2022-01-21	99,64
EPI_ISL_9284331	BA.1.1	BA.2	BA.2_1	BRBR-33978DD|EPI_ISL_9284331|United Kingdom|BA.1.1|2022-01-21	99,08
EPI_ISL_9284976	BA.1.17	BA.1	BA.1_2	MILK-33B02FE|EPI_ISL_9284976|United Kingdom|BA.1.17|2022-01-21	99,64
EPI_ISL_9284994	BA.1	BA.1.15	BA.1.15	MILK-33B0D0C|EPI_ISL_9284994|United Kingdom|BA.1|2022-01-23	99,62
EPI_ISL_9285168	BA.1	BA.1.15	BA.1.15	MILK-33B20A7|EPI_ISL_9285168|United Kingdom|BA.1|2022-01-22	99,64
EPI_ISL_9285649	BA.1.1	BA.1.15	BA.1.15	CMX-INMEGEN-53-299|EPI_ISL_9285649|Mexico|BA.1.1|2022-01-17	83,74
EPI_ISL_9285976	BA.1.21	BA.1	BA.1_1	aicbu4168|EPI_ISL_9285976|Sri Lanka|BA.1.21|2022-01-18	99,64
EPI_ISL_9286203	BA.1.17.2	BA.1.17.2	BA.1.17.2	LOND-YYBN6M8|EPI_ISL_9286203|United Kingdom|BA.1.17.2|2022-01-13	95,92
EPI_ISL_9286291	BA.1.1	BA.1.1	BA.1.1_2	LOND-YYBNHPF|EPI_ISL_9286291|United Kingdom|BA.1.1|2022-01-09	96,03
EPI_ISL_9286866	BA.1.1.7	BA.1.1	BA.1.1_3	PHEC-4R050ZCC|EPI_ISL_9286866|United Kingdom|BA.1.1.7|2022	99,64
EPI_ISL_9288417	BA.1.17.2	BA.1.17.2	BA.1.17.2	ALDP-33E4A17|EPI_ISL_9288417|United Kingdom|BA.1.17.2|2022-01-22	99,64
EPI_ISL_9291956	BA.1	BA.1.15	BA.1.15	PHEC-YYNDUAW|EPI_ISL_9291956|United Kingdom|BA.1|2022-01-03	95,33
EPI_ISL_9292065	BA.1.17.2	BA.1.17.2	BA.1.17.2	ALDP-33A6A99|EPI_ISL_9292065|United Kingdom|BA.1.17.2|2022-01-21	99,64
EPI_ISL_9292081	BA.1.1	BA.1.1	BA.1.1_3	QEUH-33AB88B|EPI_ISL_9292081|United Kingdom|BA.1.1|2022-01-21	99,64
EPI_ISL_9292242	BA.1.1	BA.1.1	BA.1.1_3	QEUH-33ABD04|EPI_ISL_9292242|United Kingdom|BA.1.1|2022-01-20	99,64

EPI_ISL_9292347	BA.1.1.15	BA.1.1	BA.1.1_2	NEWC-33DB415|EPI_ISL_9292347|United Kingdom|BA.1.1.15|2022-01-24	99,64
EPI_ISL_9294118	BA.1.1	BA.1.1	BA.1.1_3	BRBR-33D85AC|EPI_ISL_9294118|United Kingdom|BA.1.1|2022-01-23	99,64
EPI_ISL_9294703	BA.1	BA.1.15	BA.1.15	QEUH-33CD3CE|EPI_ISL_9294703|United Kingdom|BA.1|2022-01-22	99,64
EPI_ISL_9296346	BA.1.1	BA.1.1	BA.1.1_3	QEUH-33B9C42|EPI_ISL_9296346|United Kingdom|BA.1.1|2022-01-20	99,64
EPI_ISL_9296881	BA.1.1	BA.1.1	BA.1.1_2	PLYM-33BB78B|EPI_ISL_9296881|United Kingdom|BA.1.1|2022-01-22	99,64
EPI_ISL_9299618	BA.1.1.15	BA.1.1	BA.1.1_3	QEUH-33C6529|EPI_ISL_9299618|United Kingdom|BA.1.1.15|2022-01-20	99,64
EPI_ISL_9299883	BA.1.1	BA.1.1	BA.1.1_3	QEUH-33C79FF|EPI_ISL_9299883|United Kingdom|BA.1.1|2022-01-20	99,64
EPI_ISL_9300509	BA.1.1	BA.1.1	BA.1.1_3	SCOT-3473|EPI_ISL_9300509|United Kingdom|BA.1.1|2022-01-10	94,81
EPI_ISL_9301019	BA.1.17.2	BA.1.17.2	BA.1.17.2	PT26313|EPI_ISL_9301019|Portugal|BA.1.17.2|2022-01-16	99,64
EPI_ISL_9301098	BA.1.17	BA.1.15	BA.1.15	PT26084|EPI_ISL_9301098|Portugal|BA.1.17|2022-01-16	97,46
EPI_ISL_9301821	BA.1.15	BA.1.15	BA.1.15	WA-UW-22010482468|EPI_ISL_9301821|USA|BA.1.15|2022-01-04	93,80
EPI_ISL_9302656	BA.1	BA.1.15	BA.1.15	VIC37267|EPI_ISL_9302656|Australia|BA.1|2022-01-16	92,16
EPI_ISL_9304091	BA.1	BA.1.15	BA.1.15	SP-IB_148141|EPI_ISL_9304091|Brazil|BA.1|2022-01-10	99,64
EPI_ISL_9304271	BA.1	B.1.617.2	B.1.617.2_5	SP-IB_148334|EPI_ISL_9304271|Brazil|BA.1|2022-01-07	77,85
EPI_ISL_9304763	BA.1.1	BA.1.1	BA.1.1_2	SP-IB_148878|EPI_ISL_9304763|Brazil|BA.1.1|2022-01-12	99,64
EPI_ISL_9304775	BA.1.14.1	BA.1.15	BA.1.15	SP-IB_148890|EPI_ISL_9304775|Brazil|BA.1.14.1|2022-01-12	95,21
EPI_ISL_9305408	BA.1.1	BA.1.1	BA.1.1_2	SP-IB_147176|EPI_ISL_9305408|Brazil|BA.1.1|2022-01-07	99,64
EPI_ISL_9306662	BA.1.1	BA.1.15	BA.1.15	CVL-8002676|EPI_ISL_9306662|Israel|BA.1.1|2022-01-16	59,32
EPI_ISL_9307806	BA.1	BA.1	BA.1_4	ALDP-34251E7|EPI_ISL_9307806|United Kingdom|BA.1|2022-01-24	99,64
EPI_ISL_9308300	BA.1	BA.1.15	BA.1.15	PLYM-340C1B9|EPI_ISL_9308300|United Kingdom|BA.1|2022-01-24	99,64
EPI_ISL_9310767	BA.1	BA.1.15	BA.1.15	KK-NVRL-S21IRL00605460|EPI_ISL_9310767|Ireland|BA.1|2021-12-28	98,61
EPI_ISL_9310812	BA.1.17	BA.1	BA.1_4	KK-NVRL-S21IRL00606425|EPI_ISL_9310812|Ireland|BA.1.17|2021-12-28	99,64
EPI_ISL_9313283	BA.1.1	BA.1.1	BA.1.1_3	MILK-33FCD9B|EPI_ISL_9313283|United Kingdom|BA.1.1|2022-01-24	99,64
EPI_ISL_9314222	BA.1.15.1	BA.1.15.1	BA.1.15.1	MILK-3409AC0|EPI_ISL_9314222|United Kingdom|BA.1.15.1|2022-01-23	99,64
EPI_ISL_9314748	BA.1.1.7	BA.1.1	BA.1.1_2	PLYM-3408BBF|EPI_ISL_9314748|United Kingdom|BA.1.1.7|2022-01-24	99,64
EPI_ISL_9315100	BA.1.1	BA.1.1	BA.1.1_3	MILK-33EF1AE|EPI_ISL_9315100|United Kingdom|BA.1.1|2022-01-22	99,64
EPI_ISL_9317034	BA.1	BA.1.15	BA.1.15	MILK-33FF703|EPI_ISL_9317034|United Kingdom|BA.1|2022-01-24	99,60
EPI_ISL_9320472	BA.1.1	BA.1.1	BA.1.1_2	ARA-HCL022010912901|EPI_ISL_9320472|France|BA.1.1|2022-01-10	96,03
EPI_ISL_9321257	BA.1	BA.1.15	BA.1.15	RJ-LNN07884|EPI_ISL_9321257|Brazil|BA.1|2022-01-03	99,64
EPI_ISL_9321646	BA.1.1	BA.1.15	BA.1.15	LB-R00056-S023|EPI_ISL_9321646|Austria|BA.1.1|2022-01-21	91,26
EPI_ISL_9322241	BA.1.17	BA.1	BA.1_3	ARA-HCL022014422501|EPI_ISL_9322241|France|BA.1.17|2022-01-10	95,57
EPI_ISL_9322292	BA.1.1.1	BA.1.1	BA.1.1_2	ARA-HCL722000019901|EPI_ISL_9322292|France|BA.1.1.1|2022-01-17	96,03
EPI_ISL_9324989	BA.1.18	BA.1	BA.1_3	ZH-UZH-IMV-3ba5c239|EPI_ISL_9324989|Switzerland|BA.1.18|2022-01-20	99,60
EPI_ISL_9326015	BA.1.20	BA.1	BA.1_4	GA-CDC-MMB12576386|EPI_ISL_9326015|USA|BA.1.20|2021-12-29	97,20
EPI_ISL_9326063	BA.1.1	BA.1	BA.1_4	GA-CDC-MMB12588685|EPI_ISL_9326063|USA|BA.1.1|2021-12-29	91,05
EPI_ISL_9327426	BA.1.1	BA.1.1	BA.1.1_2	CA-CDC-FG-245476|EPI_ISL_9327426|USA|BA.1.1|2022-01-15	99,52
EPI_ISL_9327543	BA.1.15	BA.1.15	BA.1.15	TX-CDC-FG-242907|EPI_ISL_9327543|USA|BA.1.15|2022-01-15	96,36
EPI_ISL_9327988	BA.1.15	BA.1.15	BA.1.15	TX-CDC-FG-244912|EPI_ISL_9327988|USA|BA.1.15|2022-01-16	96,91
EPI_ISL_9329003	BA.1.1	BA.1.1	BA.1.1_2	CA-CDC-FG-246663|EPI_ISL_9329003|USA|BA.1.1|2022-01-18	92,92
EPI_ISL_9329164	BA.1.17	BA.1	BA.1_2	DC-CDC-2-5440802|EPI_ISL_9329164|USA|BA.1.17|2021-12-09	98,74
EPI_ISL_9329859	BA.2.10	BA.2	BA.2_1	KA-RFNB-4221|EPI_ISL_9329859|India|BA.2.10|2022-01-17	83,53
EPI_ISL_9330336	BA.1.15	BA.1.15	BA.1.15	TX-CDC-FG-244804|EPI_ISL_9330336|USA|BA.1.15|2022-01-11	93,47
EPI_ISL_9331022	BA.1.1	BA.1.1	BA.1.1_2	GA-CDC-MMB12571119|EPI_ISL_9331022|USA|BA.1.1|2021-12-29	97,67
EPI_ISL_9331742	BA.1.15	BA.1	BA.1_3	FL-CDC-ASC210586850|EPI_ISL_9331742|USA|BA.1.15|2022-01-06	96,93
EPI_ISL_9331974	BA.1.1	BA.1.1	BA.1.1_2	WA-CDC-FG-239743|EPI_ISL_9331974|USA|BA.1.1|2022-01-10	99,52
EPI_ISL_9333092	BA.1.1	BA.1.1	BA.1.1_2	FL-CDC-ASC210588950|EPI_ISL_9333092|USA|BA.1.1|2022-01-10	99,64
EPI_ISL_9333340	BA.1	BA.1	BA.1_3	LA-CDC-ASC210581119|EPI_ISL_9333340|USA|BA.1|2022-01-10	96,85
EPI_ISL_9333378	BA.1.1	BA.1.1	BA.1.1_2	CA-CDC-FG-239923|EPI_ISL_9333378|USA|BA.1.1|2022-01-16	90,99
EPI_ISL_9333901	BA.1.1	BA.1.1	BA.1.1_2	NY-Wadsworth-22000786-01|EPI_ISL_9333901|USA|BA.1.1|2021-12-27	96,15
EPI_ISL_9335589	BA.1.1.8	BA.1	BA.1_4	AL-CDC-ASC210592954|EPI_ISL_9335589|USA|BA.1.1.8|2022-01-16	99,64
EPI_ISL_9336590	BA.1.1	BA.1.1	BA.1.1_2	MN-CDC-IBX163128251312|EPI_ISL_9336590|USA|BA.1.1|2022-01-12	96,57
EPI_ISL_9337616	BA.1.15	BA.1.15	BA.1.15	CA-CDC-FG-246743|EPI_ISL_9337616|USA|BA.1.15|2022-01-22	94,66
EPI_ISL_9338208	BA.1.17.2	BA.1.17.2	BA.1.17.2	CA-CDC-FG-246716|EPI_ISL_9338208|USA|BA.1.17.2|2022-01-22	93,47
EPI_ISL_9338700	BA.1.1	BA.1.1	BA.1.1_2	MT-CDC-LC0489438|EPI_ISL_9338700|USA|BA.1.1|2022-01-10	99,64
EPI_ISL_9338874	BA.1.1	BA.1.1	BA.1.1_2	OR-CDC-LC0489207|EPI_ISL_9338874|USA|BA.1.1|2022-01-10	99,64
EPI_ISL_9340417	BA.1.1.18	BA.1.1	BA.1.1_3	AR-CDC-LC0488535|EPI_ISL_9340417|USA|BA.1.1.18|2022-01-11	99,64
EPI_ISL_9342500	BA.1.15	BA.1.15	BA.1.15	NV-CDC-LC0490312|EPI_ISL_9342500|USA|BA.1.15|2022-01-11	99,64
EPI_ISL_9345365	BA.1.20	BA.1.15	BA.1.15	MN-MDH-21481|EPI_ISL_9345365|USA|BA.1.20|2022-01-22	96,03
EPI_ISL_9346474	BA.1	BA.1.15	BA.1.15	NE-CUMC_22024090|EPI_ISL_9346474|USA|BA.1|2022-01-21	81,40
EPI_ISL_9346611	BA.1.1	BA.1.1	BA.1.1_2	FL-BPHL-20017|EPI_ISL_9346611|USA|BA.1.1|2021-12-21	97,23
EPI_ISL_9347505	BA.1.1	BA.1	BA.1_1	LA-EVTL11482|EPI_ISL_9347505|USA|BA.1.1|2021-12-28	95,13
EPI_ISL_9348294	BA.1.1	BA.1.1	BA.1.1_2	NY-PRL-2022_0110_16M06|EPI_ISL_9348294|USA|BA.1.1|2022-01-07	81,26
EPI_ISL_9349355	BA.1.17.2	BA.1.17.2	BA.1.17.2	NY-PRL-2022_0120_05H22|EPI_ISL_9349355|USA|BA.1.17.2|2022-01-15	99,26
EPI_ISL_9350790	BA.1	BA.1	BA.1_4	NY-PRL-2022_0118_13A09|EPI_ISL_9350790|USA|BA.1|2022-01-13	99,62
EPI_ISL_9351049	BA.1.1	BA.1.1	BA.1.1_2	NY-PRL-2022_0118_09G11|EPI_ISL_9351049|USA|BA.1.1|2022-01-12	85,10
EPI_ISL_9351842	BA.1.1	BA.1.1	BA.1.1_2	IA-SHL-2022395|EPI_ISL_9351842|USA|BA.1.1|2022-01-22	99,64
EPI_ISL_9354142	BA.1.17	BA.1.15	BA.1.15	UZA-UA-CV8321254023|EPI_ISL_9354142|Belgium|BA.1.17|2022-01-04	97,10
EPI_ISL_9355209	BA.1	BA.1	BA.1_2	NY-PRL-2022_0120_07C04|EPI_ISL_9355209|USA|BA.1|2022-01-14	99,52
EPI_ISL_9355946	BA.1.1.14	BA.1.1	BA.1.1_3	PHEP-YYDIOHJ|EPI_ISL_9355946|United Kingdom|BA.1.1.14|2022-01-08	84,47
EPI_ISL_9356306	BA.1.1	BA.1.1	BA.1.1_2	MN-MDH-21612|EPI_ISL_9356306|USA|BA.1.1|2022-01-10	96,03
EPI_ISL_9358330	BA.1	BA.1.15	BA.1.15	MILK-3461B1E|EPI_ISL_9358330|United Kingdom|BA.1|2022-01-27	99,64
EPI_ISL_9359221	BA.2	BA.2	BA.2_1	DCGC-340055|EPI_ISL_9359221|Denmark|BA.2|2022-01-23	99,81
EPI_ISL_9361977	BA.1.1	BA.1.1	BA.1.1_2	BRBR-344A207|EPI_ISL_9361977|United Kingdom|BA.1.1|2022-01-26	99,64
EPI_ISL_9362279	BA.1	BA.1	BA.1_4	DCGC-340647|EPI_ISL_9362279|Denmark|BA.1|2022-01-21	99,64
EPI_ISL_9362931	BA.1.17	BA.1.15	BA.1.15	NSW-ICPMR-19717|EPI_ISL_9362931|Australia|BA.1.17|2022-01-14	99,64
EPI_ISL_9365063	BA.1.1	BA.1.1	BA.1.1_2	CT-HUGTiPM074SV9H7|EPI_ISL_9365063|Spain|BA.1.1|2022-01-22	96,64
EPI_ISL_9367341	BA.1.17	BA.1	BA.1_2	ALDP-3447149|EPI_ISL_9367341|United Kingdom|BA.1.17|2022-01-25	99,64
EPI_ISL_9368893	BA.1.1.1	BA.1.1	BA.1.1_3	IDF-112201019140|EPI_ISL_9368893|France|BA.1.1.1|2022-01-05	83,99
EPI_ISL_9369271	BA.1.17.2	BA.1.17.2	BA.1.17.2	QEUH-3442050|EPI_ISL_9369271|United Kingdom|BA.1.17.2|2022-01-25	99,64
EPI_ISL_9374441	BA.2	A	A_14	MILK-344ACFA|EPI_ISL_9374441|United Kingdom|BA.2|2022-01-26	99,81
EPI_ISL_9374644	BA.1.1	BA.1.1	BA.1.1_3	ALDP-343D726|EPI_ISL_9374644|United Kingdom|BA.1.1|2022-01-25	99,64
EPI_ISL_9375094	BA.1	BA.1.15	BA.1.15	CA-CDC-STM-KT8HRPWN7|EPI_ISL_9375094|USA|BA.1|2022-01-16	99,64
EPI_ISL_9376925	BA.1.1	BA.1.1	BA.1.1_3	ALDP-3449280|EPI_ISL_9376925|United Kingdom|BA.1.1|2022-01-25	99,64
EPI_ISL_9377312	BA.1	BA.1	BA.1_4	ALDP-343F9E2|EPI_ISL_9377312|United Kingdom|BA.1|2022-01-21	99,64
EPI_ISL_9377850	BA.1.20	BA.1.15	BA.1.15	AZ-TG1187550|EPI_ISL_9377850|USA|BA.1.20|2022-01-14	83,46
EPI_ISL_9379206	BA.1.1	BA.1.1	BA.1.1_2	ICH-741116519|EPI_ISL_9379206|Israel|BA.1.1|2022-01-25	91,45
EPI_ISL_9379508	BA.1.17.2	BA.1.17.2	BA.1.17.2	HSLL-342EF11|EPI_ISL_9379508|United Kingdom|BA.1.17.2|2022-01-23	99,64
EPI_ISL_9380601	BA.1.15	BA.1	BA.1_4	GA-CDC-MMB13658218|EPI_ISL_9380601|USA|BA.1.15|2022-01-19	96,72
EPI_ISL_9381481	BA.1	BA.1.15	BA.1.15	LSPA-3420182|EPI_ISL_9381481|United Kingdom|BA.1|2022-01-22	99,64
EPI_ISL_9382396	BA.1.1	BA.1.1	BA.1.1_2	CA-CDC-FG-247276|EPI_ISL_9382396|USA|BA.1.1|2022-01-23	90,02
EPI_ISL_9382622	BA.2.9	A	A_17	BRBR-34182F3|EPI_ISL_9382622|United Kingdom|BA.2.9|2022-01-25	99,81
EPI_ISL_9382773	BA.1.1	BA.1.1	BA.1.1_2	BRBR-341F766|EPI_ISL_9382773|United Kingdom|BA.1.1|2022-01-25	99,62
EPI_ISL_9384670	BA.1.1	BA.1.1	BA.1.1_3	24177|EPI_ISL_9384670|Croatia|BA.1.1|2022-01-08	99,64
EPI_ISL_9387205	BA.1.1	BA.1.1	BA.1.1_3	QEUH-33EC912|EPI_ISL_9387205|United Kingdom|BA.1.1|2022-01-21	99,64
EPI_ISL_9387669	BA.1.1	BA.1.1	BA.1.1_3	QEUH-33DDE06|EPI_ISL_9387669|United Kingdom|BA.1.1|2022-01-22	99,64
EPI_ISL_9387872	BA.1.1.15	BA.1	BA.1_4	AZDelta-2203-33141|EPI_ISL_9387872|Belgium|BA.1.1.15|2022-01-26	92,65
EPI_ISL_9391299	BA.1.1.18	BA.1.1	BA.1.1_2	CO-CDPHE-2102701985|EPI_ISL_9391299|USA|BA.1.1.18|2022-01-12	90,98
EPI_ISL_9393003	BA.1	BA.1	BA.1_3	CA-CDPH-2000057698|EPI_ISL_9393003|USA|BA.1|2021-12-21	99,62
EPI_ISL_9394910	BA.1.1.18	BA.1.1	BA.1.1_2	NY-Wadsworth-22002365-01|EPI_ISL_9394910|USA|BA.1.1.18|2022-01-09	96,26
EPI_ISL_9395266	BA.1.1.18	BA.1.1	BA.1.1_2	CO-CDPHE-2102701870|EPI_ISL_9395266|USA|BA.1.1.18|2022-01-12	90,98
EPI_ISL_9395512	BA.1.1	BA.1.1	BA.1.1_2	CO-CDPHE-2102702821|EPI_ISL_9395512|USA|BA.1.1|2022-01-18	90,98

EPI_ISL_9395573	BA.1.1	BA.1.1	BA.1.1_2	CO-CDPHE-2102724232|EPI_ISL_9395573|USA|BA.1.1|2022-01-15	90,98
EPI_ISL_9397742	BA.1.17	BA.1.15	BA.1.15	WI-MHDL-2022010719|EPI_ISL_9397742|USA|BA.1.17|2022-01-10	84,05
EPI_ISL_9397929	BA.1	BA.1.15	BA.1.15	ALDP-3466742|EPI_ISL_9397929|United Kingdom|BA.1|2022-01-26	99,64
EPI_ISL_9398265	BA.1.1	BA.1.1	BA.1.1_3	ALDP-3467028|EPI_ISL_9398265|United Kingdom|BA.1.1|2022-01-26	99,64
EPI_ISL_9398646	BA.1.1	BA.1.1	BA.1.1_2	QEUH-3462A2F|EPI_ISL_9398646|United Kingdom|BA.1.1|2022-01-27	99,64
EPI_ISL_9399705	BA.1.1	BA.1.1	BA.1.1_2	HSLL-345DD79|EPI_ISL_9399705|United Kingdom|BA.1.1|2022-01-25	99,64
EPI_ISL_9400811	BA.1.1	BA.1.1	BA.1.1_2	CT-Yale-16005|EPI_ISL_9400811|USA|BA.1.1|2022-01-11	96,18
EPI_ISL_9401307	BA.1.15	BA.1	BA.1_1	MA-CDCBI-CRSP_6ETI5QNY5RIUER7B|EPI_ISL_9401307|USA|BA.1.15|2022-01-20	91,26
EPI_ISL_9402863	BA.1.1	BA.1.1	BA.1.1_3	PHEC-4S0ACZF0|EPI_ISL_9402863|United Kingdom|BA.1.1|2022	93,32
EPI_ISL_9403545	BA.1.1	BA.1.1	BA.1.1_3	SK-NML-317405|EPI_ISL_9403545|Canada|BA.1.1|2022-01-06	99,64
EPI_ISL_9404086	BA.1	BA.1	BA.1_4	HSGM-F19133|EPI_ISL_9404086|Turkey|BA.1|2022-01-18	99,64
EPI_ISL_9404199	BA.1	BA.1.15	BA.1.15	HSGM-F19085|EPI_ISL_9404199|Turkey|BA.1|2022-01-18	99,64
EPI_ISL_9404979	BA.1.1	BA.1.1	BA.1.1_3	PHEC-4T06EZ58|EPI_ISL_9404979|United Kingdom|BA.1.1|2022	99,64
EPI_ISL_9407969	BA.1.1	BA.1	BA.1_4	PHEC-4U038Z20|EPI_ISL_9407969|United Kingdom|BA.1.1|2022	96,68
EPI_ISL_9408669	BA.1.1	BA.1	BA.1_4	PHEC-4U07AZ9F|EPI_ISL_9408669|United Kingdom|BA.1.1|2022	84,96
EPI_ISL_9408695	BA.1.1	BA.1.1	BA.1.1_2	DCGC-342212|EPI_ISL_9408695|Denmark|BA.1.1|2022-01-25	99,56
EPI_ISL_9409367	BA.1	BA.2.12.1	BA.2.12.1	PHEC-4U085Z7F|EPI_ISL_9409367|United Kingdom|BA.1|2022	78,19
EPI_ISL_9409967	BA.2.9	A	A_17	DCGC-342531|EPI_ISL_9409967|Denmark|BA.2.9|2022-01-24	99,81
EPI_ISL_9410581	BA.2	A	A_17	DCGC-342823|EPI_ISL_9410581|Denmark|BA.2|2022-01-23	99,81
EPI_ISL_9410777	BA.1.15	BA.1.15	BA.1.15	MO-UMGC-33245|EPI_ISL_9410777|USA|BA.1.15|2022-01-12	84,53
EPI_ISL_9411488	BA.1	BA.1	BA.1_3	PHEC-YYNR4T3|EPI_ISL_9411488|United Kingdom|BA.1|2022-01-08	96,64
EPI_ISL_9411526	BA.1	BA.1	BA.1_4	PHEC-YYNR5AK|EPI_ISL_9411526|United Kingdom|BA.1|2022-01-11	96,72
EPI_ISL_9415878	BA.1.15	BA.1.15	BA.1.15	un-HMN-22012030600|EPI_ISL_9415878|France|BA.1.15|2021-12-25	95,12
EPI_ISL_9416310	BA.1.15	BA.1.15	BA.1.15	2995556863|EPI_ISL_9416310|Sweden|BA.1.15|2022-01-17	99,64
EPI_ISL_9417143	BA.1.1	BA.1.1	BA.1.1_2	NIC-INSPI-386884|EPI_ISL_9417143|Ecuador|BA.1.1|2022-01-14	99,64
EPI_ISL_9417188	BA.1.1	BA.1.15	BA.1.15	NIC-INSPI-108171|EPI_ISL_9417188|Ecuador|BA.1.1|2022-01-14	78,69
EPI_ISL_9418107	BA.1	BA.1.15	BA.1.15	GE-RUMC-000364|EPI_ISL_9418107|Netherlands|BA.1|2022-01-10	91,41
EPI_ISL_9420224	BA.1	BA.1.1	BA.1.1_1	IMBA_IMP-1440_G03|EPI_ISL_9420224|Austria|BA.1|2022-01-07	49,80
EPI_ISL_9420658	BA.1	BA.1.1	BA.1.1_1	IMBA_IMP-1447_H08|EPI_ISL_9420658|Austria|BA.1|2022-01-09	49,80
EPI_ISL_9420953	BA.1	BA.1.1	BA.1.1_1	IMBA_IMP-1446_D01|EPI_ISL_9420953|Austria|BA.1|2022-01-08	46,82
EPI_ISL_9422248	BA.1	BA.1.1	BA.1.1_1	IMBA_IMP-1475_H09|EPI_ISL_9422248|Austria|BA.1|2022-01-02	49,80
EPI_ISL_9424878	BA.1	AY.43	AY.43_1	IMBA_IMP-1418_C03|EPI_ISL_9424878|Austria|BA.1|2022-01-02	46,50
EPI_ISL_9428960	BA.1.1	BA.1.1	BA.1.1_2	RI-CDC-ASC210596000|EPI_ISL_9428960|USA|BA.1.1|2022-01-04	92,27
EPI_ISL_9431081	BA.1	BA.1.15	BA.1.15	HID_InDRE_FB995_E13313523408_S10796|EPI_ISL_9431081|Mexico|BA.1|2022-01-04	99,62
EPI_ISL_9431726	BA.1	BA.1	BA.1_4	PZH-UMB-10286|EPI_ISL_9431726|Poland|BA.1|2022-01-15	98,38
EPI_ISL_9432545	BA.1	BA.1	BA.1_4	PZH-UMB-9886|EPI_ISL_9432545|Poland|BA.1|2022-01-15	99,64
EPI_ISL_9435144	BA.1.1	BA.1.1	BA.1.1_2	VI-CDC-2-5451737|EPI_ISL_9435144|U.S. Virgin Islands|BA.1.1|2022-01-06	99,64
EPI_ISL_9435493	BA.1.1	BA.1.1	BA.1.1_2	WA-CDC-2-5464548|EPI_ISL_9435493|USA|BA.1.1|2022-01-10	99,64
EPI_ISL_9435856	BA.1.15	BA.1.15	BA.1.15	TX-CDC-QDX32887674|EPI_ISL_9435856|USA|BA.1.15|2022-01-14	99,64
EPI_ISL_9437872	BA.1.15	BA.1	BA.1_3	ND-NDDH-10719|EPI_ISL_9437872|USA|BA.1.15|2022-01-21	90,38
EPI_ISL_9440033	BA.1	BA.1.15	BA.1.15	LSPA-347783C|EPI_ISL_9440033|United Kingdom|BA.1|2022-01-25	99,64
EPI_ISL_9440067	BA.1.1	BA.1.1	BA.1.1_2	LSPA-34772F2|EPI_ISL_9440067|United Kingdom|BA.1.1|2022-01-26	99,64
EPI_ISL_9440088	BA.1.18	BA.1.15	BA.1.15	MILK-3479366|EPI_ISL_9440088|United Kingdom|BA.1.18|2022-01-28	99,64
EPI_ISL_9440248	BA.1.1	BA.1.1	BA.1.1_3	NEWC-3471823|EPI_ISL_9440248|United Kingdom|BA.1.1|2022-01-26	99,64
EPI_ISL_9443072	BA.1	BA.1.15	BA.1.15	PLYM-34691F6|EPI_ISL_9443072|United Kingdom|BA.1|2022-01-27	99,64
EPI_ISL_9448254	BA.1.16	BA.1	BA.1_4	PHEC-4V04CZ14|EPI_ISL_9448254|United Kingdom|BA.1.16|2022	98,49
EPI_ISL_9449120	BA.2	A	A_17	DCGC-344146|EPI_ISL_9449120|Denmark|BA.2|2022-01-25	99,96
EPI_ISL_9450042	BA.1	BA.1.15	BA.1.15	BW-RKI-I-479292|EPI_ISL_9450042|Germany|BA.1|2022-01-12	99,64
EPI_ISL_9450413	BA.1.1	BA.1	BA.1_3	BY-RKI-I-479421|EPI_ISL_9450413|Germany|BA.1.1|2022-01-14	98,97
EPI_ISL_9450420	BA.2.9	A	A_17	DCGC-344488|EPI_ISL_9450420|Denmark|BA.2.9|2022-01-24	99,81
EPI_ISL_9450693	BA.2	A	A_17	DCGC-344580|EPI_ISL_9450693|Denmark|BA.2|2022-01-25	99,81
EPI_ISL_9451053	BA.1.1	BA.1.1	BA.1.1_3	BY-RKI-I-479634|EPI_ISL_9451053|Germany|BA.1.1|2022-01-20	99,54
EPI_ISL_9452854	BA.1.1	BA.1.1	BA.1.1_2	NY-PRL-2022_0104_03B08|EPI_ISL_9452854|USA|BA.1.1|2021-12-26	94,64
EPI_ISL_9453098	BA.2	A	A_17	DCGC-345252|EPI_ISL_9453098|Denmark|BA.2|2022-01-25	99,79
EPI_ISL_9453264	BA.1.17	BA.2.12.1	BA.2.12.1	BW-RKI-I-480253|EPI_ISL_9453264|Germany|BA.1.17|2022-01-20	83,82
EPI_ISL_9453647	BA.1.1.1	BA.1.1	BA.1.1_3	BW-RKI-I-480387|EPI_ISL_9453647|Germany|BA.1.1.1|2022-01-20	92,63
EPI_ISL_9454624	BA.1.17.2	BA.1.17.2	BA.1.17.2	PHEP-YYDZFHO|EPI_ISL_9454624|United Kingdom|BA.1.17.2|2022-01-05	95,84
EPI_ISL_9456001	BA.1	BA.1	BA.1_3	PHWC-P8K4PZ|EPI_ISL_9456001|United Kingdom|BA.1|2022-01-04	94,56
EPI_ISL_9457996	BA.1.1	BA.1.1	BA.1.1_2	PHWC-P8MWZG|EPI_ISL_9457996|United Kingdom|BA.1.1|2022-01-06	96,03
EPI_ISL_9460060	BA.1	BA.1.1	BA.1.1_3	BY-RKI-I-484075|EPI_ISL_9460060|Germany|BA.1|2022-01-20	96,58
EPI_ISL_9463180	BA.1	BA.1.15	BA.1.15	NW-RKI-I-487452|EPI_ISL_9463180|Germany|BA.1|2022-01-25	99,64
EPI_ISL_9463201	BA.1.1	BA.1.1	BA.1.1_3	NW-RKI-I-487473|EPI_ISL_9463201|Germany|BA.1.1|2022-01-25	99,64
EPI_ISL_9465267	BA.1.1	BA.1.1	BA.1.1_3	KY-CDC-LC0497515|EPI_ISL_9465267|USA|BA.1.1|2022-01-12	99,64
EPI_ISL_9466572	BA.2.10.1	BA.2.10.1	BA.2.10.1	WB-INSACOG-1931502630478|EPI_ISL_9466572|India|BA.2.10.1|2021-12-29	99,81
EPI_ISL_9471393	BA.1.1	BA.1.1	BA.1.1_2	AZ-ASPHL-0441|EPI_ISL_9471393|USA|BA.1.1|2022-01-21	96,03
EPI_ISL_9474571	BA.1.1	BA.1.1	BA.1.1_2	NB-CHUDGLD-210122-MM01926R|EPI_ISL_9474571|Canada|BA.1.1|2022-01-21	99,64
EPI_ISL_9475052	BA.1.1	BA.1.1	BA.1.1_2	FL-CDC-LC0491699|EPI_ISL_9475052|USA|BA.1.1|2022-01-18	99,64
EPI_ISL_9475478	BA.1.1	BA.1.1	BA.1.1_3	VA-CDC-LC0495746|EPI_ISL_9475478|USA|BA.1.1|2022-01-18	99,64
EPI_ISL_9478809	BA.1.1	BA.1	BA.1_1	MA-CDCBI-CRSP_BRPIAQLIY7MKVI6P|EPI_ISL_9478809|USA|BA.1.1|2022-01-24	88,84
EPI_ISL_9480888	BA.1.1	BA.1.1	BA.1.1_2	NY-CDCBI-CRSP_W5IBLDSARQGRIWG2|EPI_ISL_9480888|USA|BA.1.1|2022-01-21	94,52
EPI_ISL_9481372	BA.1.1	BA.1.1	BA.1.1_2	MA-CDCBI-CRSP_Q7EPTPLD454IDLSD|EPI_ISL_9481372|USA|BA.1.1|2022-01-23	92,04
EPI_ISL_9482122	BA.1.1	BA.1.1	BA.1.1_2	NJ-CDC-LC0494405|EPI_ISL_9482122|USA|BA.1.1|2022-01-19	99,64
EPI_ISL_9482500	BA.1.17	BA.1	BA.1_2	SL-SU-20477069|EPI_ISL_9482500|Germany|BA.1.17|2022-01-28	99,62
EPI_ISL_9483067	BA.1.1	BA.1.1	BA.1.1_2	MN-MDH-22060|EPI_ISL_9483067|USA|BA.1.1|2022-01-17	96,03
EPI_ISL_9484392	BA.1.1	BA.1.15	BA.1.15	CO-CDC-MMB13681412|EPI_ISL_9484392|USA|BA.1.1|2022-01-19	95,42
EPI_ISL_9485909	BA.1.1	BA.1.1	BA.1.1_2	FL-CDC-ASC210582777|EPI_ISL_9485909|USA|BA.1.1|2022-01-11	96,85
EPI_ISL_9486363	BA.1.15	BA.1	BA.1_3	LA-CDC-ASC210593503|EPI_ISL_9486363|USA|BA.1.15|2022-01-12	96,87
EPI_ISL_9487190	BA.1.1	BA.1.1	BA.1.1_2	QC-L00426776001|EPI_ISL_9487190|Canada|BA.1.1|2021-12-29	99,64
EPI_ISL_9488797	BA.1	BA.2.12	BA.2.12	DL-IGIB1120220255536TV|EPI_ISL_9488797|India|BA.1|2022-01	88,84
EPI_ISL_9489937	BA.1	BA.1	BA.1_1	RI-CDCBI-RIDOH_SHICPJGDNW2NCXSB|EPI_ISL_9489937|USA|BA.1|2022-01-10	95,10
EPI_ISL_9490197	BA.1.1	BA.1	BA.1_1	MA-CDCBI-CRSP_DSHY2SYXZNQ5IX7G|EPI_ISL_9490197|USA|BA.1.1|2022-01-26	95,13
EPI_ISL_9499602	BA.1.17.2	BA.1.17.2	BA.1.17.2	PHWC-P8M91F|EPI_ISL_9499602|United Kingdom|BA.1.17.2|2022-01-15	96,03
EPI_ISL_9500040	BA.2	BA.2	BA.2_1	DCGC-347414|EPI_ISL_9500040|Denmark|BA.2|2022-01-27	99,79
EPI_ISL_9500363	BA.1.14.2	BA.1.15	BA.1.15	AM-FIOCRUZ-ILMD2202066|EPI_ISL_9500363|Brazil|BA.1.14.2|2022-01-19	99,64
EPI_ISL_9501857	BA.1.1	BA.1.1	BA.1.1_2	AB-ABPHL-50860|EPI_ISL_9501857|Canada|BA.1.1|2022-01-13	99,64
EPI_ISL_9502538	BA.1.1	BA.1	BA.1_4	CHP-INMEGEN-54-333|EPI_ISL_9502538|Mexico|BA.1.1|2022-01-18	90,08
EPI_ISL_9503924	BA.1.1	BA.1.1	BA.1.1_3	MH-ICMR-NIV-INSACOG-381999|EPI_ISL_9503924|India|BA.1.1|2021-12-15	99,81
EPI_ISL_9507126	BA.1	BA.1	BA.1_4	ALDP-3473311|EPI_ISL_9507126|United Kingdom|BA.1|2022-01-26	99,64
EPI_ISL_9508282	BA.1.1	BA.1.1	BA.1.1_2	PAC-IHU-60609-N1M|EPI_ISL_9508282|France|BA.1.1|2022-01	96,03
EPI_ISL_9509522	BA.1.1	BA.1.1	BA.1.1_2	PZH-UMB-10934|EPI_ISL_9509522|Poland|BA.1.1|2022-01-21	99,64
EPI_ISL_9511738	BA.1	BA.1.15	BA.1.15	6221163976|EPI_ISL_9511738|Sweden|BA.1|2022-01-20	99,64
EPI_ISL_9511821	BA.2	BA.2	BA.2_1	2946887464|EPI_ISL_9511821|Sweden|BA.2|2022-01-20	91,47
EPI_ISL_9513187	BA.1.1	BA.1	BA.1_4	587303|EPI_ISL_9513187|Slovenia|BA.1.1|2021-12-28	99,60
EPI_ISL_9515347	BA.1	BA.1	BA.1_4	MBLG-CTMAPF87658809|EPI_ISL_9515347|Belgium|BA.1|2022-01-24	99,64
EPI_ISL_9516078	BA.1.15	BA.1	BA.1_4	FL-BPHL-20094|EPI_ISL_9516078|USA|BA.1.15|2021-12-20	99,64
EPI_ISL_9516753	BA.1	BA.1.15	BA.1.15	CO-CDC-LC0505551|EPI_ISL_9516753|USA|BA.1|2022-01-17	99,64
EPI_ISL_9516834	BA.1	BA.1	BA.1_4	CT-SEMA4-2789|EPI_ISL_9516834|USA|BA.1|2022-01-12	99,64
EPI_ISL_9517246	BA.1.1	BA.1.1	BA.1.1_2	OK-CDC-LC0503972|EPI_ISL_9517246|USA|BA.1.1|2022-01-18	99,64
EPI_ISL_9518559	BA.1.1	BA.1.1	BA.1.1_3	FL-Curative-105407|EPI_ISL_9518559|USA|BA.1.1|2022-01-14	70,22
EPI_ISL_9518563	BA.1.1.18	BA.1.1	BA.1.1_3	FL-Curative-170829|EPI_ISL_9518563|USA|BA.1.1.18|2022-01-14	80,79
EPI_ISL_9519269	BA.1.1	BA.1.1	BA.1.1_2	UT-UPHL-220202623060|EPI_ISL_9519269|USA|BA.1.1|2022-01-22	99,64

EPI_ISL_9520573	BA.1.1	BA.1.1	BA.1.1_2	ID-IBL-787573|EPI_ISL_9520573|USA|BA.1.1|2022-01-20	99,60
EPI_ISL_9521125	BA.1.1	BA.1.1	BA.1.1_2	NRL_S341|EPI_ISL_9521125|Czech Republic|BA.1.1|2022-01-20	99,64
EPI_ISL_9524953	BA.1	BA.1	BA.1_4	CO-CDC-MMB13687582|EPI_ISL_9524953|USA|BA.1|2022-01-19	93,34
EPI_ISL_9527668	BA.1.1	BA.1.1	BA.1.1_2	FL-CDC-ASC210599944|EPI_ISL_9527668|USA|BA.1.1|2022-01-16	96,68
EPI_ISL_9528050	BA.1.18	BA.1	BA.1_3	IL-CDC-ASC210601117|EPI_ISL_9528050|USA|BA.1.18|2022-01-17	96,89
EPI_ISL_9528257	BA.1.1.1	BA.1.1	BA.1.1_3	ZH-EMC-4821|EPI_ISL_9528257|Netherlands|BA.1.1.1|2022-01-17	93,00
EPI_ISL_9528904	BA.1.15	BA.1.15	BA.1.15	KS-CDC-ASC210592867|EPI_ISL_9528904|USA|BA.1.15|2022-01-17	99,64
EPI_ISL_9533077	BA.1	BA.1	BA.1_1	NV-NSPHL-22-00004906|EPI_ISL_9533077|USA|BA.1|2022-01-03	95,42
EPI_ISL_9534108	BA.1.17.2	BA.1.17.2	BA.1.17.2	PZH-GUM-9070|EPI_ISL_9534108|Poland|BA.1.17.2|2022-01-20	99,64
EPI_ISL_9534218	BA.1	BA.1	BA.1_4	PZH-GUM-9228|EPI_ISL_9534218|Poland|BA.1|2022-01-22	80,75
EPI_ISL_9553755	BA.1.1.18	BA.1.1	BA.1.1_2	CO-CDPHE-2102722154|EPI_ISL_9553755|USA|BA.1.1.18|2022-01-14	90,98
EPI_ISL_9555396	BA.1.15.1	BA.1.15	BA.1.15	NORT-YB4B51|EPI_ISL_9555396|United Kingdom|BA.1.15.1|2022-01-12	95,86
EPI_ISL_9555846	BA.1.17.2	BA.1.17.2	BA.1.17.2	ALDP-34B728D|EPI_ISL_9555846|United Kingdom|BA.1.17.2|2022-01-28	99,64
EPI_ISL_9556297	BA.1.1	BA.1.1	BA.1.1_2	LSPA-34B3F02|EPI_ISL_9556297|United Kingdom|BA.1.1|2022-01-29	99,64
EPI_ISL_9559870	BA.1	BA.1.15	BA.1.15	LSPA-3494D67|EPI_ISL_9559870|United Kingdom|BA.1|2022-01-26	96,15
EPI_ISL_9559980	BA.1.1	BA.1.15	BA.1.15	LSPA-3495698|EPI_ISL_9559980|United Kingdom|BA.1.1|2022-01-26	99,64
EPI_ISL_9560300	BA.1	BA.1.15	BA.1.15	ALDP-348723B|EPI_ISL_9560300|United Kingdom|BA.1|2022-01-28	99,64
EPI_ISL_9561474	BA.1.1	BA.1.1	BA.1.1_3	QEUH-3490994|EPI_ISL_9561474|United Kingdom|BA.1.1|2022-01-29	99,64
EPI_ISL_9561752	BA.1	BA.1.15	BA.1.15	MILK-3498ECA|EPI_ISL_9561752|United Kingdom|BA.1|2022-01-29	99,64
EPI_ISL_9561846	BA.1.1	BA.1.1	BA.1.1_2	MILK-3498F5E|EPI_ISL_9561846|United Kingdom|BA.1.1|2022-01-29	99,64
EPI_ISL_9562114	BA.1.1.1	BA.1.1	BA.1.1_2	MILK-349FCBA|EPI_ISL_9562114|United Kingdom|BA.1.1.1|2022-01-29	99,64
EPI_ISL_9562589	BA.1.17.2	BA.1.15	BA.1.15	PHEC-YYN8DQT|EPI_ISL_9562589|United Kingdom|BA.1.17.2|2022-01-23	97,86
EPI_ISL_9563053	BA.1.17.2	BA.1.17.2	BA.1.17.2	ALDP-3489F14|EPI_ISL_9563053|United Kingdom|BA.1.17.2|2022-01-29	99,64
EPI_ISL_9563068	BA.1.1	BA.1.1	BA.1.1_3	PHEC-YYNG73N|EPI_ISL_9563068|United Kingdom|BA.1.1|2022-01-10	89,16
EPI_ISL_9565392	BA.1	BA.1.15	BA.1.15	QEUH-3495C2D|EPI_ISL_9565392|United Kingdom|BA.1|2022-01-28	93,23
EPI_ISL_9566200	BA.2	A	A_17	DCGC-349543|EPI_ISL_9566200|Denmark|BA.2|2022-01-26	99,98
EPI_ISL_9566412	BA.1	BA.1	BA.1_1	DCGC-349602|EPI_ISL_9566412|Denmark|BA.1|2022-01-27	99,50
EPI_ISL_9567836	BA.1	BA.1	BA.1_3	PHWC-P8QWK5|EPI_ISL_9567836|United Kingdom|BA.1|2022-01-16	96,03
EPI_ISL_9570502	BA.1.18	BA.1.15	BA.1.15	HDF-IPP04507|EPI_ISL_9570502|France|BA.1.18|2022-01-16	99,64
EPI_ISL_9571837	BA.1.15	BA.1.15	BA.1.15	LA-OD-6649251680|EPI_ISL_9571837|USA|BA.1.15|2022-01-24	96,05
EPI_ISL_9572169	BA.1.1	BA.1.1	BA.1.1_2	CO-CDPHE-2102722419|EPI_ISL_9572169|USA|BA.1.1|2022-01-16	96,03
EPI_ISL_9572213	BA.1	BA.1	BA.1_3	CO-CDPHE-2102740943|EPI_ISL_9572213|USA|BA.1|2022-01-18	96,03
EPI_ISL_9572568	BA.1.1	BA.1.1	BA.1.1_2	CO-CDPHE-2102721852|EPI_ISL_9572568|USA|BA.1.1|2022-01-16	87,50
EPI_ISL_9574167	BA.1.15	BA.1	BA.1_1	MA-CDCBI-CRSP_EGG26XCJQFPJMPHC|EPI_ISL_9574167|USA|BA.1.15|2022-01-24	95,04
EPI_ISL_9574550	BA.1.1	BA.1.1	BA.1.1_2	MA-CDCBI-CRSP_GM33TDOUUHXFWRFV|EPI_ISL_9574550|USA|BA.1.1|2022-01-27	95,12
EPI_ISL_9575028	BA.1	BA.1	BA.1_1	MA-CDCBI-CRSP_YIIZVI4DRP3CNXGK|EPI_ISL_9575028|USA|BA.1|2022-01-24	95,13
EPI_ISL_9575654	BA.1.20	BA.1	BA.1_1	VT-CDCBI-CRSP_GSVYBEE4PGIPIRCP|EPI_ISL_9575654|USA|BA.1.20|2022-01-25	95,12
EPI_ISL_9575810	BA.1.1	BA.1.1	BA.1.1_2	MA-CDCBI-CRSP_WBYGWUDY65RBDKNA|EPI_ISL_9575810|USA|BA.1.1|2022-01-27	94,52
EPI_ISL_9576507	BA.1.1	BA.1.1	BA.1.1_2	CA-CDC-STM-897KWHDJW|EPI_ISL_9576507|USA|BA.1.1|2022-01-18	99,62
EPI_ISL_9577972	BA.1	BA.1	BA.1_3	IA-CDC-FG-253233|EPI_ISL_9577972|USA|BA.1|2022-01-22	97,96
EPI_ISL_9578558	BA.1.1	BA.1.1	BA.1.1_2	NV-CDC-FG-252864|EPI_ISL_9578558|USA|BA.1.1|2022-01-25	90,99
EPI_ISL_9580818	BA.1.1	BA.1.1	BA.1.1_3	ALDP-34C69E0|EPI_ISL_9580818|United Kingdom|BA.1.1|2022-01-30	99,64
EPI_ISL_9581135	BA.1.17.2	BA.1.17.2	BA.1.17.2	NEWC-34C85CC|EPI_ISL_9581135|United Kingdom|BA.1.17.2|2022-01-30	99,64
EPI_ISL_9581228	BA.1.1	BA.1.1	BA.1.1_3	ALDP-34C9DC3|EPI_ISL_9581228|United Kingdom|BA.1.1|2022-01-30	99,64
EPI_ISL_9581278	BA.1	BA.1	BA.1_4	ALDP-34CA64F|EPI_ISL_9581278|United Kingdom|BA.1|2022-01-30	99,64
EPI_ISL_9581683	BA.1.1.4	BA.1.1	BA.1.1_2	ALDP-34CD354|EPI_ISL_9581683|United Kingdom|BA.1.1.4|2022-01-29	99,64
EPI_ISL_9582157	BA.1.15	BA.1.15	BA.1.15	MILK-34B149C|EPI_ISL_9582157|United Kingdom|BA.1.15|2022-01-30	99,62
EPI_ISL_9583959	BA.1.1	BA.1.1	BA.1.1_3	LSPA-34BEC97|EPI_ISL_9583959|United Kingdom|BA.1.1|2022-01-28	99,64
EPI_ISL_9584140	BA.1.16	BA.1	BA.1_3	LSPA-34B19E8|EPI_ISL_9584140|United Kingdom|BA.1.16|2022-01-27	99,64
EPI_ISL_9586481	BA.1.1	BA.1.1	BA.1.1_3	LSPA-34BCB41|EPI_ISL_9586481|United Kingdom|BA.1.1|2022-01-28	99,64
EPI_ISL_9586951	BA.1.1.13	BA.1.1	BA.1.1_3	LOND-YYBDQRD|EPI_ISL_9586951|United Kingdom|BA.1.1.13|2021-12-20	95,92
EPI_ISL_9586963	BA.1.15.1	BA.1.15	BA.1.15	LOND-YYBDXDR|EPI_ISL_9586963|United Kingdom|BA.1.15.1|2021-12-28	95,92
EPI_ISL_9587481	BA.1	BA.1	BA.1_4	PHEC-4W0A1ZC4|EPI_ISL_9587481|United Kingdom|BA.1|2022	99,64
EPI_ISL_9589276	BA.1	BA.1	BA.1_3	PHEC-YYN8JIH|EPI_ISL_9589276|United Kingdom|BA.1|2022-01-07	96,72
EPI_ISL_9589543	BA.1	BA.1	BA.1_4	2863|EPI_ISL_9589543|Poland|BA.1|2022-02-05	97,20
EPI_ISL_9590208	BA.1.1	BA.1.15	BA.1.15	SMC-7038701|EPI_ISL_9590208|Israel|BA.1.1|2022-01-18	73,84
EPI_ISL_9596360	BA.1.17.2	BA.1	BA.1_4	SIC_CQRC_3422006753|EPI_ISL_9596360|Italy|BA.1.17.2|2022-01-22	95,00
EPI_ISL_9596632	BA.1.18	BA.1.15	BA.1.15	CT-HUGTiPM075MP4B4|EPI_ISL_9596632|Spain|BA.1.18|2022-01-24	99,64
EPI_ISL_9599294	BA.1.1	BA.1.1	BA.1.1_2	LSPA-34E6EDC|EPI_ISL_9599294|United Kingdom|BA.1.1|2022-01-27	99,64
EPI_ISL_9599523	BA.1.17.2	BA.1.17.2	BA.1.17.2	LSPA-34E6C2D|EPI_ISL_9599523|United Kingdom|BA.1.17.2|2022-01-28	99,64
EPI_ISL_9600028	BA.2	A	A_14	HSLL-34ED233|EPI_ISL_9600028|United Kingdom|BA.2|2022-01-30	99,81
EPI_ISL_9602145	BA.1.1	BA.1.1	BA.1.1_2	ARCH-002643B8|EPI_ISL_9602145|United Kingdom|BA.1.1|2022	96,01
EPI_ISL_9602381	BA.1.1	BA.1.1	BA.1.1_2	NEWC-34E13C3|EPI_ISL_9602381|United Kingdom|BA.1.1|2022-01-31	99,64
EPI_ISL_9603367	BA.1.15.1	BA.1.15.1	BA.1.15.1	HSLL-34F6B87|EPI_ISL_9603367|United Kingdom|BA.1.15.1|2022-02-01	99,64
EPI_ISL_9603445	BA.1.1.1	BA.1.1	BA.1.1_3	NAQ-HCL722000113001|EPI_ISL_9603445|France|BA.1.1.1|2022-01-24	99,64
EPI_ISL_9604289	BA.2	A	A_14	GJ-INSACOG-GBRC4814|EPI_ISL_9604289|India|BA.2|2022-01-20	99,03
EPI_ISL_9604574	BA.1.1.1	BA.1.1	BA.1.1_3	NOR-HMN-22022020771|EPI_ISL_9604574|France|BA.1.1.1|2022-01-31	83,30
EPI_ISL_9604674	BA.1.1.1	BA.1.1	BA.1.1_3	IDF-HMN-22012260340|EPI_ISL_9604674|France|BA.1.1.1|2022-01-03	78,48
EPI_ISL_9605568	BA.1	BA.1	BA.1_3	NORT-YB57S6|EPI_ISL_9605568|United Kingdom|BA.1|2022	93,46
EPI_ISL_9605629	BA.1.17.2	BA.1.17.2	BA.1.17.2	NORT-YB5BOG|EPI_ISL_9605629|United Kingdom|BA.1.17.2|2022-01-18	94,05
EPI_ISL_9605691	BA.1.17.2	BA.1.17.2	BA.1.17.2	NORT-YB5ED4|EPI_ISL_9605691|United Kingdom|BA.1.17.2|2022-01-19	94,91
EPI_ISL_9605765	BA.1.1.14	BA.1.1	BA.1.1_2	NORT-YB5I8F|EPI_ISL_9605765|United Kingdom|BA.1.1.14|2022	91,51
EPI_ISL_9605771	BA.1.1	A	A_1	NCDC-NR1818|EPI_ISL_9605771|Nigeria|BA.1.1|2021-12-09	66,65
EPI_ISL_9609870	BA.1.1	BA.1.1	BA.1.1_2	CT-HUVH-E35927|EPI_ISL_9609870|Spain|BA.1.1|2022-01-24	96,39
EPI_ISL_9610739	BA.1	BA.1.15	BA.1.15	SMC-7037357|EPI_ISL_9610739|Israel|BA.1|2022-01-12	84,24
EPI_ISL_9610780	BA.1.1	BA.1.15	BA.1.15	SMC-7037428|EPI_ISL_9610780|Israel|BA.1.1|2022-01-13	74,11
EPI_ISL_9613860	BA.1.1	BA.1.1	BA.1.1_3	LSPA-34E55AB|EPI_ISL_9613860|United Kingdom|BA.1.1|2022-01-29	99,62
EPI_ISL_9614027	BA.1	BA.1	BA.1_3	D-NVRL-S22IRL00019248|EPI_ISL_9614027|Ireland|BA.1|2022-01-04	99,60
EPI_ISL_9614084	BA.1.16	BA.1	BA.1_4	LH-NVRL-S22IRL00021177|EPI_ISL_9614084|Ireland|BA.1.16|2022-01-04	99,64
EPI_ISL_9615310	BA.1	BA.2	BA.2_1	SMC-7033874|EPI_ISL_9615310|Israel|BA.1|2022-01-03	76,61
EPI_ISL_9615767	BA.1.1	BA.1.1	BA.1.1_2	OH-22AM-028CO00741|EPI_ISL_9615767|USA|BA.1.1|2022-01-28	99,64
EPI_ISL_9616370	BA.1.1.1	BA.1.1	BA.1.1_2	ZH-EMC-4919|EPI_ISL_9616370|Netherlands|BA.1.1.1|2022-01-29	95,94
EPI_ISL_9616703	BA.1.18	BA.1	BA.1_2	LNS6529706|EPI_ISL_9616703|Luxembourg|BA.1.18|2022-01-17	94,45
EPI_ISL_9617493	BA.1.17	BA.1.15	BA.1.15	LNS2711309|EPI_ISL_9617493|Luxembourg|BA.1.17|2022-01-21	79,39
EPI_ISL_9619987	BA.1.17	BA.1	BA.1_3	CL-COV23224|EPI_ISL_9619987|Spain|BA.1.17|2022-01-13	96,03
EPI_ISL_9621412	BA.1.17.2	BA.1.17.2	BA.1.17.2	QEUH-34D1B64|EPI_ISL_9621412|United Kingdom|BA.1.17.2|2022-01-31	99,64
EPI_ISL_9622715	BA.1.1	BA.1.1	BA.1.1_2	01_SE100_22CS501226|EPI_ISL_9622715|Sweden|BA.1.1|2022-01-26	96,43
EPI_ISL_9622795	BA.1	BA.1.15	BA.1.15	MN-CDC-IBX274185487011|EPI_ISL_9622795|USA|BA.1|2022-01-18	96,53
EPI_ISL_9623025	BA.1	BA.1	BA.1_4	01_SE100_22CS501315|EPI_ISL_9623025|Sweden|BA.1|2022-01-27	97,92
EPI_ISL_9623779	BA.1.1	BA.1.1	BA.1.1_3	FL-BPHL-20461|EPI_ISL_9623779|USA|BA.1.1|2021-12-27	97,84
EPI_ISL_9624930	BA.1.1	BA.1.1	BA.1.1_2	CA-CDC-QDX33015447|EPI_ISL_9624930|USA|BA.1.1|2022-01-18	99,64
EPI_ISL_9625077	BA.1.1	BA.1.1	BA.1.1_2	MS-MSPHL-0392|EPI_ISL_9625077|USA|BA.1.1|2022-01-20	94,73
EPI_ISL_9625334	BA.1.20	BA.1.15	BA.1.15	WI-UW-9058|EPI_ISL_9625334|USA|BA.1.20|2022-01-25	99,64
EPI_ISL_9625676	BA.1.1	BA.1.1	BA.1.1_3	LA-CDC-4479742-001|EPI_ISL_9625676|USA|BA.1.1|2022-01-03	99,64
EPI_ISL_9626501	BA.1.1	BA.1.1	BA.1.1_2	1354|EPI_ISL_9626501|Norway|BA.1.1|2022-01-04	97,56
EPI_ISL_9627708	BA.1.1.10	BA.1.1	BA.1.1_3	KS-KHEL-9615|EPI_ISL_9627708|USA|BA.1.1.10|2022-02-01	99,62
EPI_ISL_9628591	BA.1.1	BA.1.1	BA.1.1_2	TX-CDC-QDX32932407|EPI_ISL_9628591|USA|BA.1.1|2022-01-15	99,64
EPI_ISL_9628760	BA.1.1	BA.1.1	BA.1.1_3	IL-CDC-QDX33015775|EPI_ISL_9628760|USA|BA.1.1|2022-01-17	99,64
EPI_ISL_9631739	BA.1.1	BA.1	BA.1_4	CAM-TIGEM-IZSM-COLLI-25485|EPI_ISL_9631739|Italy|BA.1.1|2022-01-19	98,42
EPI_ISL_9633535	BA.1.15	BA.1.15	BA.1.15	IA-CDC-ASC210603640|EPI_ISL_9633535|USA|BA.1.15|2022-01-21	99,64
EPI_ISL_9634126	BA.1.1	BA.1.1	BA.1.1_2	TN-CDC-ASC210681940|EPI_ISL_9634126|USA|BA.1.1|2022-01-21	98,03

EPI_ISL_9635806	BA.1.15	BA.1.15	BA.1.15	TX-CDC-ASC210682459|EPI_ISL_9635806|USA|BA.1.15|2022-01-24	99,64
EPI_ISL_9635946	BA.1.1	BA.1.1	BA.1.1_3	MT-CDC-ASC210683565|EPI_ISL_9635946|USA|BA.1.1|2022-01-25	99,64
EPI_ISL_9636012	BA.1.1.18	BA.1.1	BA.1.1_2	NC-CDC-ASC210683624|EPI_ISL_9636012|USA|BA.1.1.18|2022-01-25	96,83
EPI_ISL_9636266	BA.1.17.2	BA.1.15	BA.1.15	NCDC-NR2622|EPI_ISL_9636266|Nigeria|BA.1.17.2|2021-12-22	86,45
EPI_ISL_9638807	BA.1.17	BA.1.15	BA.1.15	QLD32110|EPI_ISL_9638807|Australia|BA.1.17|2022-01-06	99,64
EPI_ISL_9639762	BA.1.17	BA.1.15	BA.1.15	QLD30146|EPI_ISL_9639762|Australia|BA.1.17|2022-01-04	99,64
EPI_ISL_9640982	BA.2	BA.2	BA.2_1	ALDP-351E7DF|EPI_ISL_9640982|United Kingdom|BA.2|2022-02-01	91,93
EPI_ISL_9642759	BA.2	BA.2	BA.2_1	LSPA-350A19C|EPI_ISL_9642759|United Kingdom|BA.2|2022-01-31	99,81
EPI_ISL_9643842	BA.1.17	BA.1	BA.1_4	ALDP-34FA97A|EPI_ISL_9643842|United Kingdom|BA.1.17|2022-01-31	99,64
EPI_ISL_9645465	BA.1	BA.1.15	BA.1.15	HSLL-3503D80|EPI_ISL_9645465|United Kingdom|BA.1|2022-01-31	99,64
EPI_ISL_9645531	BA.1.1	BA.1.1	BA.1.1_3	ALDP-35068B9|EPI_ISL_9645531|United Kingdom|BA.1.1|2022-01-31	99,64
EPI_ISL_9647648	BA.1	BA.1.15	BA.1.15	QEUH-34F1A25|EPI_ISL_9647648|United Kingdom|BA.1|2022-01-31	99,64
EPI_ISL_9647774	BA.1	BA.1.15	BA.1.15	QEUH-34F2CE2|EPI_ISL_9647774|United Kingdom|BA.1|2022-01-31	99,64
EPI_ISL_9649657	BA.1	BA.1.15	BA.1.15	PHEP-YYD5DN9|EPI_ISL_9649657|United Kingdom|BA.1|2022-01-23	84,49
EPI_ISL_9649688	BA.1.17.2	BA.1.15	BA.1.15	PHEP-YYD5FGI|EPI_ISL_9649688|United Kingdom|BA.1.17.2|2022-01-17	84,49
EPI_ISL_9651874	BA.2.9	A	A_17	DCGC-351777|EPI_ISL_9651874|Denmark|BA.2.9|2022-02-01	99,81
EPI_ISL_9652184	BA.2	BA.2	BA.2_1	DCGC-351981|EPI_ISL_9652184|Denmark|BA.2|2022-01-31	99,98
EPI_ISL_9652784	BA.1.17.2	BA.1	BA.1_2	DCGC-352641|EPI_ISL_9652784|Denmark|BA.1.17.2|2022-01-31	99,68
EPI_ISL_9653523	BA.2	A	A_17	DCGC-353392|EPI_ISL_9653523|Denmark|BA.2|2022-01-31	99,81
EPI_ISL_9654389	BA.1	BA.1	BA.1_1	DCGC-354174|EPI_ISL_9654389|Denmark|BA.1|2022-01-30	99,68
EPI_ISL_9655133	BA.2.9	A	A_17	DCGC-354722|EPI_ISL_9655133|Denmark|BA.2.9|2022-01-30	99,79
EPI_ISL_9657098	BA.1.1.1	BA.1.1	BA.1.1_2	SMC-7042188|EPI_ISL_9657098|Israel|BA.1.1.1|2022-02-01	96,26
EPI_ISL_9657176	BA.1.1	BA.1.1	BA.1.1_2	SMC-7040448|EPI_ISL_9657176|Israel|BA.1.1|2022-01-27	95,90
EPI_ISL_9658237	BA.1.18	BA.1	BA.1_4	HH-hpi-p12168|EPI_ISL_9658237|Germany|BA.1.18|2022-01-06	92,94
EPI_ISL_9658430	BA.1.9	BA.1.9	BA.1.9	NY-PRL-2022_0131_01D15|EPI_ISL_9658430|USA|BA.1.9|2022-01-27	99,64
EPI_ISL_9658570	BA.1.1	BA.1.1	BA.1.1_2	NY-Wadsworth-22005372-01|EPI_ISL_9658570|USA|BA.1.1|2022-01-25	95,82
EPI_ISL_9658637	BA.1	BA.1	BA.1_3	NY-Wadsworth-22003654-01|EPI_ISL_9658637|USA|BA.1|2022-01-12	96,03
EPI_ISL_9660375	BA.1.1	BA.1.15	BA.1.15	NY-PRL-2022_0126_00H24|EPI_ISL_9660375|USA|BA.1.1|2022-01-24	99,62
EPI_ISL_9661647	BA.1.1	BA.1.1	BA.1.1_3	NY-PRL-2022_0120_15J08|EPI_ISL_9661647|USA|BA.1.1|2022-01-18	99,52
EPI_ISL_9662938	BA.1	BA.1	BA.1_3	UKDD-ZUUL-D202201513|EPI_ISL_9662938|Czech Republic|BA.1|2022-01-18	96,47
EPI_ISL_9664781	BA.1.1.18	BA.1	BA.1_4	CO-CDC-FG-257016|EPI_ISL_9664781|USA|BA.1.1.18|2022-01-26	78,55
EPI_ISL_9665186	BA.2	A	A_17	BE-ChVir-LB-220204-8464|EPI_ISL_9665186|Germany|BA.2|2022-01-28	95,17
EPI_ISL_9665876	BA.1.1	BA.1.1	BA.1.1_2	CO-CDC-FG-255919|EPI_ISL_9665876|USA|BA.1.1|2022-01-28	92,90
EPI_ISL_9666507	BA.1	BA.1.15	BA.1.15	MN-CDC-IBX422360891228|EPI_ISL_9666507|USA|BA.1|2022-01-16	96,24
EPI_ISL_9666525	BA.1.1	BA.1.1	BA.1.1_2	MN-CDC-IBX813750822848|EPI_ISL_9666525|USA|BA.1.1|2022-01-16	96,41
EPI_ISL_9668305	BA.1.1	BA.1.1	BA.1.1_2	CA-SC-263952|EPI_ISL_9668305|USA|BA.1.1|2022-01-15	95,61
EPI_ISL_9668726	BA.1.1	BA.1.1	BA.1.1_2	OH-CDC-QDX33118676|EPI_ISL_9668726|USA|BA.1.1|2022-01-19	99,64
EPI_ISL_9672384	BA.1.1	BA.1	BA.1_4	RI-CDC-2-5488332|EPI_ISL_9672384|USA|BA.1.1|2022-01-05	99,62
EPI_ISL_9675967	BA.1.1.18	BA.1.1	BA.1.1_2	CO-CDPHE-2102715765|EPI_ISL_9675967|USA|BA.1.1.18|2022-01-17	90,98
EPI_ISL_9678352	BA.1.1	BA.1.1	BA.1.1_2	CT-HUGTiPM076TF1H10|EPI_ISL_9678352|Spain|BA.1.1|2022-01-31	99,64
EPI_ISL_9681212	BC.2	BA.1.1	BA.1.1_2	APU-INS-13168|EPI_ISL_9681212|Peru|BC.2|2021-12-30	99,56
EPI_ISL_9683048	BA.1.16	BA.1	BA.1_3	MILK-35450E3|EPI_ISL_9683048|United Kingdom|BA.1.16|2022-02-03	99,64
EPI_ISL_9684192	BA.1.1	BA.1.1	BA.1.1_2	ON-PHL-22-05475|EPI_ISL_9684192|Canada|BA.1.1|2022-01-15	91,55
EPI_ISL_9685048	BA.1.1	BA.1.1	BA.1.1_2	ON-PHL-22-06591|EPI_ISL_9685048|Canada|BA.1.1|2022-01-25	90,98
EPI_ISL_9687412	BA.1.1	BA.1.1	BA.1.1_2	LSPA-3519446|EPI_ISL_9687412|United Kingdom|BA.1.1|2022-01-30	99,62
EPI_ISL_9687536	BA.1.15	BA.1.15	BA.1.15	LSPA-35174B1|EPI_ISL_9687536|United Kingdom|BA.1.15|2022-01-30	99,64
EPI_ISL_9688096	BA.1	BA.1	BA.1_4	PZH-UMB-11396|EPI_ISL_9688096|Poland|BA.1|2022-01-24	83,21
EPI_ISL_9688845	BA.1.17.2	BA.1.17.2	BA.1.17.2	ALDP-34FE150|EPI_ISL_9688845|United Kingdom|BA.1.17.2|2022-01-31	99,64
EPI_ISL_9689614	BA.1.17.2	BA.1	BA.1_4	PHEC-5N053ZE1|EPI_ISL_9689614|United Kingdom|BA.1.17.2|2022	99,39
EPI_ISL_9690153	BA.2.9	A	A_17	DCGC-355522|EPI_ISL_9690153|Denmark|BA.2.9|2022-01-31	99,81
EPI_ISL_9690232	BA.2	A	A_17	DCGC-355570|EPI_ISL_9690232|Denmark|BA.2|2022-01-31	97,10
EPI_ISL_9692696	BA.2	BA.2.10	BA.2.10	IDF-CERBAHC-01950634|EPI_ISL_9692696|France|BA.2|2022-01-31	97,58
EPI_ISL_9692699	BA.1.1.1	BA.1.1	BA.1.1_2	IDF-CERBAHC-01951032|EPI_ISL_9692699|France|BA.1.1.1|2022-01-31	99,16
EPI_ISL_9696182	BA.2.10	BA.2	BA.2_1	TG-CDFD-DG-21-165652|EPI_ISL_9696182|India|BA.2.10|2021-12-31	71,17
EPI_ISL_9697247	BA.1.1	BA.1.1	BA.1.1_3	icddrb-GSB05-078|EPI_ISL_9697247|Bangladesh|BA.1.1|2022-01-12	99,64
EPI_ISL_9698460	BA.2	BA.2	BA.2_1	NI-IOV-1234792|EPI_ISL_9698460|Germany|BA.2|2022-01-18	93,99
EPI_ISL_9703780	BA.1.1	BA.1.1	BA.1.1_2	NY-UB-KHL-00830|EPI_ISL_9703780|USA|BA.1.1|2022-01-05	91,09
EPI_ISL_9703932	BA.1.1	BA.1	BA.1_4	ICH-741118077|EPI_ISL_9703932|Israel|BA.1.1|2022-01-29	66,40
EPI_ISL_9708611	BA.1.1	BA.1.1	BA.1.1_2	GA-IVY-HML1C141|EPI_ISL_9708611|USA|BA.1.1|2022-01-01	99,64
EPI_ISL_9709738	BA.1.1	BA.1.1	BA.1.1_2	LL-8401|EPI_ISL_9709738|Chile|BA.1.1|2022-01-11	94,94
EPI_ISL_9712352	BA.1	BA.1.15	BA.1.15	NY_MEDDAC_FD_313|EPI_ISL_9712352|USA|BA.1|2021-12-29	99,64
EPI_ISL_9713536	BA.1	BA.1.1	BA.1.1_3	PZH-UMB-12750|EPI_ISL_9713536|Poland|BA.1|2022-01-19	84,37
EPI_ISL_9713975	BA.1.1.11	BA.1.1	BA.1.1_2	VEN-IZSVe-22RS8041-6_VI|EPI_ISL_9713975|Italy|BA.1.1.11|2022-01-29	96,30
EPI_ISL_9714498	BA.1.1	BA.1.1	BA.1.1_2	08-016501-MB|EPI_ISL_9714498|Slovenia|BA.1.1|2022-01-13	96,20
EPI_ISL_9716241	BA.1.18	BA.1	BA.1_3	SN-RKI-I-500987|EPI_ISL_9716241|Germany|BA.1.18|2022-01-14	99,62
EPI_ISL_9716538	BA.1.1	BA.1.1	BA.1.1_3	NW-RKI-I-501299|EPI_ISL_9716538|Germany|BA.1.1|2022-01-19	99,64
EPI_ISL_9716750	BA.1	BA.1	BA.1_4	RP-RKI-I-501515|EPI_ISL_9716750|Germany|BA.1|2022-01-25	96,11
EPI_ISL_9716921	BA.1.1	BA.1	BA.1_1	NW-RKI-I-502033|EPI_ISL_9716921|Germany|BA.1.1|2022-01-24	91,99
EPI_ISL_9719646	BA.1.18	BA.1	BA.1_4	BW-RKI-I-503969|EPI_ISL_9719646|Germany|BA.1.18|2022-01-31	99,64
EPI_ISL_9721128	BA.2.10	A	A_14	WB-INSACOG-1931502662710|EPI_ISL_9721128|India|BA.2.10|2022-01-01	99,81
EPI_ISL_9722716	BA.1.1	BA.1.1	BA.1.1_3	NEWC-34DA318|EPI_ISL_9722716|United Kingdom|BA.1.1|2022-01-31	99,64
EPI_ISL_9723189	BA.1.1	BA.1.1	BA.1.1_3	LSPA-35612FA|EPI_ISL_9723189|United Kingdom|BA.1.1|2022-02-02	99,64
EPI_ISL_9723567	BA.1.17.2	BA.1.17.2	BA.1.17.2	MILK-35521E0|EPI_ISL_9723567|United Kingdom|BA.1.17.2|2022-02-03	99,64
EPI_ISL_9725321	BA.1.1	BA.1.1	BA.1.1_2	HH-RKI-I-507862|EPI_ISL_9725321|Germany|BA.1.1|2022-01-28	99,64
EPI_ISL_9728027	BA.1.1	BA.1.1	BA.1.1_3	QEUH-3543B4E|EPI_ISL_9728027|United Kingdom|BA.1.1|2022-02-02	99,64
EPI_ISL_9728421	BA.1.17.2	BA.1.15	BA.1.15	EDB40257|EPI_ISL_9728421|United Kingdom|BA.1.17.2|2022-01-22	90,98
EPI_ISL_9729549	BA.1.1	BA.1.1	BA.1.1_3	LSPA-353803E|EPI_ISL_9729549|United Kingdom|BA.1.1|2022-02-01	99,64
EPI_ISL_9730257	BA.1.1	BA.1.1	BA.1.1_3	KA-RFNB-4602|EPI_ISL_9730257|India|BA.1.1|2022-01-24	86,32
EPI_ISL_9732001	BA.1	BA.1.15	BA.1.15	ALDP-354E3A5|EPI_ISL_9732001|United Kingdom|BA.1|2022-02-01	99,64
EPI_ISL_9732117	BA.1	BA.1.15	BA.1.15	QEUH-3540051|EPI_ISL_9732117|United Kingdom|BA.1|2022-02-02	99,64
EPI_ISL_9732221	BA.1	BA.1	BA.1_4	QEUH-353FE2E|EPI_ISL_9732221|United Kingdom|BA.1|2022-02-02	99,64
EPI_ISL_9732291	BA.1	BA.1	BA.1_3	HH-RKI-I-512585|EPI_ISL_9732291|Germany|BA.1|2022-01-19	95,33
EPI_ISL_9734893	BA.1.1	BA.1.1	BA.1.1_2	NORT-YBHX9A|EPI_ISL_9734893|United Kingdom|BA.1.1|2022	90,98
EPI_ISL_9735323	BA.1.17.2	BA.1	BA.1_4	MH-NVRL-S22IRL00019505|EPI_ISL_9735323|Ireland|BA.1.17.2|2022-01-04	98,32
EPI_ISL_9736205	BA.1.17.2	BA.1.17.2	BA.1.17.2	QEUH-35321C8|EPI_ISL_9736205|United Kingdom|BA.1.17.2|2022-02-02	99,64
EPI_ISL_9736380	BA.2	A	A_17	HH-RKI-I-513588|EPI_ISL_9736380|Germany|BA.2|2022-01-28	97,10
EPI_ISL_9740225	BA.2	A	A_17	DCGC-357754|EPI_ISL_9740225|Denmark|BA.2|2022-02-02	99,79
EPI_ISL_9740248	BA.1.1	BA.2	BA.2_1	NW-RKI-I-514590|EPI_ISL_9740248|Germany|BA.1.1|2022-01-25	94,26
EPI_ISL_9741324	BA.1.1	BA.1.1	BA.1.1_3	PHEP-YYDHJNZ|EPI_ISL_9741324|United Kingdom|BA.1.1|2022-01-18	79,28
EPI_ISL_9741790	BA.2	A	A_17	DCGC-358205|EPI_ISL_9741790|Denmark|BA.2|2022-02-03	99,98
EPI_ISL_9744216	BA.2.9	A	A_17	DCGC-359024|EPI_ISL_9744216|Denmark|BA.2.9|2022-02-02	99,77
EPI_ISL_9745302	BA.1	BA.1	BA.1_3	SN-RKI-I-516169|EPI_ISL_9745302|Germany|BA.1|2022-01-27	99,62
EPI_ISL_9754012	BA.1.1	BA.1	BA.1_4	AZ-ASU41442|EPI_ISL_9754012|USA|BA.1.1|2022-01-28	66,88
EPI_ISL_9754488	BA.1.1	BA.1.1	BA.1.1_2	AZ-ASU41785|EPI_ISL_9754488|USA|BA.1.1|2022-02-02	90,98
EPI_ISL_9754823	BA.1.15	BA.1	BA.1_4	INC-2891-737867|EPI_ISL_9754823|Costa Rica|BA.1.15|2022-01-19	99,64
EPI_ISL_9756777	BA.1.17.2	BA.1.15	BA.1.15	CA-CDPH-6000006757|EPI_ISL_9756777|USA|BA.1.17.2|2021-12-18	85,69
EPI_ISL_9757577	BA.1.17.2	BA.1.17.2	BA.1.17.2	CA-HLX-STM-5Y6N87NYY|EPI_ISL_9757577|USA|BA.1.17.2|2022-01-07	99,64
EPI_ISL_9757868	BA.1.1	BA.1.1	BA.1.1_2	CA-HLX-STM-GRGR293A5|EPI_ISL_9757868|USA|BA.1.1|2022-01-15	98,55
EPI_ISL_9758609	BA.1.1.2	BA.1.1	BA.1.1_3	CA-HLX-STM-M26Z283KS|EPI_ISL_9758609|USA|BA.1.1.2|2022-01-08	99,64
EPI_ISL_9758920	BA.1.15	BA.1	BA.1_4	CA-HLX-STM-GMDAHN4Z2|EPI_ISL_9758920|USA|BA.1.15|2022-01-11	93,40
EPI_ISL_9758937	BA.1.1	BA.1.1	BA.1.1_2	CA-HLX-STM-DVXEJFFVH|EPI_ISL_9758937|USA|BA.1.1|2022-01-04	99,64

EPI_ISL_9760161	BA.1	BA.1.1	BA.1.1_2	BC-BCCDC-327407|EPI_ISL_9760161|Canada|BA.1|2022-01-01	99,64
EPI_ISL_9761957	BA.1	BA.1.15	BA.1.15	BC-BCCDC-328877|EPI_ISL_9761957|Canada|BA.1|2022-01-07	99,64
EPI_ISL_9764477	BA.1	BA.1.15	BA.1.15	NY-NYGC-6308-VTM1-OJNM229|EPI_ISL_9764477|USA|BA.1|2021-12-23	99,64
EPI_ISL_9765665	BA.1.15	BA.1.15	BA.1.15	BC-BCCDC-333432|EPI_ISL_9765665|Canada|BA.1.15|2022-01-03	99,64
EPI_ISL_9766897	BA.1.17.2	BA.1.17.2	BA.1.17.2	BC-BCCDC-336715|EPI_ISL_9766897|Canada|BA.1.17.2|2022-01-13	99,64
EPI_ISL_9767211	BA.1.17.2	BA.1.17.2	BA.1.17.2	AZ-TG1218201|EPI_ISL_9767211|USA|BA.1.17.2|2021-12-21	99,62
EPI_ISL_9771536	BA.1.1	BA.1.1	BA.1.1_2	AG-ETHZ-35947157|EPI_ISL_9771536|Switzerland|BA.1.1|2022-01-23	96,76
EPI_ISL_9773211	BA.1.1.10	BA.1.1	BA.1.1_2	FR-ETHZ-35958414|EPI_ISL_9773211|Switzerland|BA.1.1.10|2022-01-24	96,79
EPI_ISL_9773915	BA.1.17.2	BA.1.15	BA.1.15	PAC-IHU-58752_Nova2|EPI_ISL_9773915|France|BA.1.17.2|2021-12	89,75
EPI_ISL_9774301	BA.1.1	BA.1.1	BA.1.1_3	PAC-IHU-58429_Nova2|EPI_ISL_9774301|France|BA.1.1|2022-01	91,66
EPI_ISL_9775344	BA.1.1	BA.1.1	BA.1.1_3	PIE_IRCC_15879490|EPI_ISL_9775344|Italy|BA.1.1|2022-01-31	88,95
EPI_ISL_9776894	BA.1.1	BA.1.1	BA.1.1_2	MILK-357F55A|EPI_ISL_9776894|United Kingdom|BA.1.1|2022-02-04	99,64
EPI_ISL_9778475	BA.1.1	BA.1.1	BA.1.1_2	LSPA-35831AA|EPI_ISL_9778475|United Kingdom|BA.1.1|2022-02-02	99,64
EPI_ISL_9778784	BA.1	BA.1.15	BA.1.15	LSPA-3578C01|EPI_ISL_9778784|United Kingdom|BA.1|2022-02-03	99,64
EPI_ISL_9779657	BA.1.13.1	BA.1	BA.1_4	JK-GS-GSILab-889950|EPI_ISL_9779657|Indonesia|BA.1.13.1|2022-01-24	99,64
EPI_ISL_9780323	BA.1.1.13	BA.1.1	BA.1.1_3	MILK-357C5C6|EPI_ISL_9780323|United Kingdom|BA.1.1.13|2022-02-04	99,64
EPI_ISL_9780787	BA.1.1	BA.1.1	BA.1.1_2	MILK-357336B|EPI_ISL_9780787|United Kingdom|BA.1.1|2022-02-04	99,64
EPI_ISL_9780954	BA.1.1	BA.1.1	BA.1.1_3	LSPA-356D4E9|EPI_ISL_9780954|United Kingdom|BA.1.1|2022-02-02	99,64
EPI_ISL_9781668	BA.2.10	A	A_14	WB-IICB-S717-1|EPI_ISL_9781668|India|BA.2.10|2022-01-27	96,81
EPI_ISL_9781673	BA.2.10.1	BA.2.10.1	BA.2.10.1	WB-IICB-S722-1|EPI_ISL_9781673|India|BA.2.10.1|2022-01-22	99,68
EPI_ISL_9782548	BA.1.1	BA.1.1	BA.1.1_3	MILK-3559E97|EPI_ISL_9782548|United Kingdom|BA.1.1|2022-02-03	99,64
EPI_ISL_9783189	BA.2	A	A_14	QEUH-3557321|EPI_ISL_9783189|United Kingdom|BA.2|2022-02-03	99,81
EPI_ISL_9784754	BA.2.9	A	A_17	DCGC-360630|EPI_ISL_9784754|Denmark|BA.2.9|2022-02-03	98,34
EPI_ISL_9785475	BA.1	BA.1.15	BA.1.15	QEUH-355B4C0|EPI_ISL_9785475|United Kingdom|BA.1|2022-02-03	99,64
EPI_ISL_9786173	BA.2.10	A	A_14	MILK-355BA55|EPI_ISL_9786173|United Kingdom|BA.2.10|2022-02-02	99,81
EPI_ISL_9786750	BA.1.1	BA.1.1	BA.1.1_2	PHWC-P8ZE6M|EPI_ISL_9786750|United Kingdom|BA.1.1|2022-01-18	95,97
EPI_ISL_9787358	BA.1.1	BA.1.1	BA.1.1_3	LSPA-35711DC|EPI_ISL_9787358|United Kingdom|BA.1.1|2022-02-02	99,64
EPI_ISL_9787363	BA.1	BA.1	BA.1_4	PHWC-P8ZKCQ|EPI_ISL_9787363|United Kingdom|BA.1|2022-01-20	96,03
EPI_ISL_9787543	BA.2	A	A_17	ALDP-357B3BE|EPI_ISL_9787543|United Kingdom|BA.2|2022-02-03	99,81
EPI_ISL_9794495	BA.1.1	BA.1.1	BA.1.1_2	CE-FIOCRUZ-57026CE|EPI_ISL_9794495|Brazil|BA.1.1|2022-01-13	99,48
EPI_ISL_9794638	BA.1.1	BA.1.1	BA.1.1_2	VS-ETHZ-36222276|EPI_ISL_9794638|Switzerland|BA.1.1|2022-02-06	92,65
EPI_ISL_9794885	BA.1	BA.1	BA.1_4	uvzsr-C10-BA_22_00002856|EPI_ISL_9794885|Slovakia|BA.1|2022-01-11	96,64
EPI_ISL_9796431	BA.1.1	BA.1.1	BA.1.1_2	Kce-1630|EPI_ISL_9796431|Poland|BA.1.1|2022-02-07	87,62
EPI_ISL_9799456	BA.1.15	BA.1.15	BA.1.15	NJ-PHEL-V22007791|EPI_ISL_9799456|USA|BA.1.15|2022-01-27	89,20
EPI_ISL_9802733	BA.1.1	BA.1	BA.1_4	ZNA_25151570|EPI_ISL_9802733|Belgium|BA.1.1|2022-02-01	92,35
EPI_ISL_9804903	BA.1.14	BA.1.15	BA.1.15	SP-IB_150242|EPI_ISL_9804903|Brazil|BA.1.14|2022-02-05	99,64
EPI_ISL_9805765	BA.1.20	BA.1	BA.1_4	CA-SEARCH-65434|EPI_ISL_9805765|USA|BA.1.20|2022-01-03	99,48
EPI_ISL_9806243	BA.1	BA.1.15	BA.1.15	CA-SEARCH-65925|EPI_ISL_9806243|USA|BA.1|2022-01-06	99,48
EPI_ISL_9806414	BA.1.15	BA.1	BA.1_4	CA-SEARCH-66108|EPI_ISL_9806414|USA|BA.1.15|2021-12-22	99,48
EPI_ISL_9807031	BA.1.1	BA.1.1	BA.1.1_2	CA-SEARCH-66820|EPI_ISL_9807031|USA|BA.1.1|2022-01-05	99,48
EPI_ISL_9808527	BA.1	BA.1	BA.1_3	CA-SEARCH-68559|EPI_ISL_9808527|USA|BA.1|2022-01-09	97,90
EPI_ISL_9808732	BA.1	BA.1	BA.1_3	CA-SEARCH-68773|EPI_ISL_9808732|USA|BA.1|2021-12-23	97,90
EPI_ISL_9809027	BA.2.3	BA.2.12	BA.2.12	CT-CDCBI-CRSP_5TOSDRUAKXST7ZG6|EPI_ISL_9809027|USA|BA.2.3|2022-01-31	95,08
EPI_ISL_9809326	BA.1.1	BA.1	BA.1_1	MA-CDCBI-CRSP_55DLKIGXOHQRACS6|EPI_ISL_9809326|USA|BA.1.1|2022-02-01	84,70
EPI_ISL_9811091	BA.1.1	BA.1.1	BA.1.1_2	MA-CDCBI-CRSP_SVHAOO7ALK5Z4MIB|EPI_ISL_9811091|USA|BA.1.1|2022-02-01	89,37
EPI_ISL_9811649	BA.1.1	BA.1.1	BA.1.1_2	VT-CDCBI-CRSP_63RSRSITJY7HHGZQ|EPI_ISL_9811649|USA|BA.1.1|2022-01-29	89,93
EPI_ISL_9812072	BA.1.20	BA.1	BA.1_1	VT-CDCBI-CRSP_X57GQA2PKCFGBI4A|EPI_ISL_9812072|USA|BA.1.20|2022-01-31	95,12
EPI_ISL_9812401	BA.1.15.1	BA.1.15	BA.1.15	LOND-YYBD1TR|EPI_ISL_9812401|United Kingdom|BA.1.15.1|2021-12-21	95,92
EPI_ISL_9812426	BA.1	BA.1	BA.1_4	LOND-YYBDIQE|EPI_ISL_9812426|United Kingdom|BA.1|2021-12-17	93,30
EPI_ISL_9813021	BA.1	BA.1	BA.1_3	NORT-YB6QZF|EPI_ISL_9813021|United Kingdom|BA.1|2022	94,68
EPI_ISL_9813095	BA.1.17.2	BA.1.17.2	BA.1.17.2	NORT-YB6W5Z|EPI_ISL_9813095|United Kingdom|BA.1.17.2|2022	96,03
EPI_ISL_9813130	BA.1	BA.1.15	BA.1.15	NORT-YB6XG8|EPI_ISL_9813130|United Kingdom|BA.1|2022	94,01
EPI_ISL_9813835	BA.1.1	BA.1.1	BA.1.1_2	NORT-YB9ZG7|EPI_ISL_9813835|United Kingdom|BA.1.1|2022	90,98
EPI_ISL_9814187	BA.1.1	BA.1.1	BA.1.1_3	PHEC-5P07BZE7|EPI_ISL_9814187|United Kingdom|BA.1.1|2022	79,30
EPI_ISL_9814979	BA.1.1.14	BA.1.1	BA.1.1_3	PHEP-YYD7HCQ|EPI_ISL_9814979|United Kingdom|BA.1.1.14|2022-01-25	83,99
EPI_ISL_9815916	BA.1.1	BA.1.1	BA.1.1_2	PHWC-P87AFN|EPI_ISL_9815916|United Kingdom|BA.1.1|2022-01-26	95,33
EPI_ISL_9816122	BA.1.17.2	BA.1.17.2	BA.1.17.2	PHWC-P8A91K|EPI_ISL_9816122|United Kingdom|BA.1.17.2|2022-01-18	95,84
EPI_ISL_9818307	BA.1	BA.1.15	BA.1.15	SCOT-4269|EPI_ISL_9818307|United Kingdom|BA.1|2022-01-19	87,12
EPI_ISL_9819270	BA.1.1	BA.1.1	BA.1.1_2	NV-NSPHL-22-00035339|EPI_ISL_9819270|USA|BA.1.1|2022-02-07	99,64
EPI_ISL_9820062	BA.1.1	BA.1.1	BA.1.1_3	ALDP-35A9C70|EPI_ISL_9820062|United Kingdom|BA.1.1|2022-02-05	99,64
EPI_ISL_9820204	BA.3	BA.1.1	BA.1.1_3	PZH-UMB-13302|EPI_ISL_9820204|Poland|BA.3|2022-01-24	83,29
EPI_ISL_9824117	BA.1.17.2	BA.1.17.2	BA.1.17.2	LSPA-3585982|EPI_ISL_9824117|United Kingdom|BA.1.17.2|2022-02-02	99,64
EPI_ISL_9824229	BA.1.1	BA.1.1	BA.1.1_3	MILK-358F812|EPI_ISL_9824229|United Kingdom|BA.1.1|2022-02-05	99,64
EPI_ISL_9825887	BA.2.10	A	A_14	MILK-359BF3C|EPI_ISL_9825887|United Kingdom|BA.2.10|2022-02-04	99,81
EPI_ISL_9826528	BA.2.1	A	A_14	MILK-3597CEE|EPI_ISL_9826528|United Kingdom|BA.2.1|2022-02-06	99,81
EPI_ISL_9826758	BA.1.1	BA.1.1	BA.1.1_3	MILK-3597C75|EPI_ISL_9826758|United Kingdom|BA.1.1|2022-02-06	99,64
EPI_ISL_9827166	BA.1.1	BA.1.1	BA.1.1_3	MILK-358F33F|EPI_ISL_9827166|United Kingdom|BA.1.1|2022-02-05	99,64
EPI_ISL_9827975	BA.1	BA.1.15	BA.1.15	ALDP-359572B|EPI_ISL_9827975|United Kingdom|BA.1|2022-02-04	99,64
EPI_ISL_9828075	BA.1.14	BA.1.15	BA.1.15	MILK-3593ECE|EPI_ISL_9828075|United Kingdom|BA.1.14|2022-02-05	99,64
EPI_ISL_9828206	BA.1.1	BA.1.1	BA.1.1_3	MILK-359507B|EPI_ISL_9828206|United Kingdom|BA.1.1|2022-02-06	99,64
EPI_ISL_9828488	BA.1.1	BA.1.1	BA.1.1_3	MILK-359364E|EPI_ISL_9828488|United Kingdom|BA.1.1|2022-02-05	99,64
EPI_ISL_9828764	BA.1.1	BA.1.1	BA.1.1_2	MILK-35A6235|EPI_ISL_9828764|United Kingdom|BA.1.1|2022-02-05	99,64
EPI_ISL_9829180	BA.1.1	BA.1.1	BA.1.1_2	ALDP-357B9AD|EPI_ISL_9829180|United Kingdom|BA.1.1|2022-02-03	99,64
EPI_ISL_9829796	BA.1.19	BA.1.15	BA.1.15	run220209_RUVZ_TN-22-0316|EPI_ISL_9829796|Slovakia|BA.1.19|2022-02-09	98,99
EPI_ISL_9830085	BA.1.1	BA.1.1	BA.1.1_2	AZ-ASU42534|EPI_ISL_9830085|USA|BA.1.1|2022-02-07	91,53
EPI_ISL_9830106	BA.1	BA.1.15	BA.1.15	AZ-ASU42568|EPI_ISL_9830106|USA|BA.1|2022-02-07	92,18
EPI_ISL_9830360	BA.1	BA.1.17.2	BA.1.17.2	PZH-GUM-10712|EPI_ISL_9830360|Poland|BA.1|2022-01-17	99,64
EPI_ISL_9831505	BA.1.15	B.1.177	B.1.177_1	FL-SED-VTM-3741|EPI_ISL_9831505|USA|BA.1.15|2022-01	87,14
EPI_ISL_9831821	BA.1.1	BA.1.1	BA.1.1_2	FL-SED-VTM-4142|EPI_ISL_9831821|USA|BA.1.1|2022-01	99,64
EPI_ISL_9832393	BA.1.1.2	BA.1.1	BA.1.1_3	SZ-NIG-4-C1177|EPI_ISL_9832393|Japan|BA.1.1.2|2022-01-16	99,64
EPI_ISL_9833814	BA.1	BA.1	BA.1_4	NORW-315C0C7|EPI_ISL_9833814|United Kingdom|BA.1|2022-01-19	99,16
EPI_ISL_9833832	BA.1	BA.1	BA.1_3	NORW-315C329|EPI_ISL_9833832|United Kingdom|BA.1|2022-01-19	96,18
EPI_ISL_9833984	BA.1	BA.1	BA.1_3	NORW-315DBD8|EPI_ISL_9833984|United Kingdom|BA.1|2022-01-19	96,03
EPI_ISL_9834453	BA.1	BA.1	BA.1_4	NORW-3160610|EPI_ISL_9834453|United Kingdom|BA.1|2022-01-01	99,64
EPI_ISL_9834623	BA.1.15	BA.1	BA.1_3	NORW-3162076|EPI_ISL_9834623|United Kingdom|BA.1.15|2022-01-02	96,76
EPI_ISL_9834973	BA.1	BA.1	BA.1_2	NORW-31659CC|EPI_ISL_9834973|United Kingdom|BA.1|2022-01-11	99,50
EPI_ISL_9835065	BA.1.1.13	BA.1.1	BA.1.1_2	NORW-3167973|EPI_ISL_9835065|United Kingdom|BA.1.1.13|2022-01-21	99,62
EPI_ISL_9835538	BA.1.17.2	BA.1.17.2	BA.1.17.2	NORW-316E36C|EPI_ISL_9835538|United Kingdom|BA.1.17.2|2022-01-21	96,15
EPI_ISL_9837669	BA.2.3	BA.2.10	BA.2.10	PHEP-YYRYPM3|EPI_ISL_9837669|United Kingdom|BA.2.3|2022	99,79
EPI_ISL_9838574	BA.2	A	A_14	MILK-35BF600|EPI_ISL_9838574|United Kingdom|BA.2|2022-02-07	99,81
EPI_ISL_9839878	BA.1.1	BA.1.1	BA.1.1_3	MILK-35BF318|EPI_ISL_9839878|United Kingdom|BA.1.1|2022-02-06	99,64
EPI_ISL_9840174	BA.1	BA.1.15	BA.1.15	MILK-35BE15B|EPI_ISL_9840174|United Kingdom|BA.1|2022-02-06	99,64
EPI_ISL_9840523	BA.1.17.2	BA.1.17.2	BA.1.17.2	MILK-35B1C57|EPI_ISL_9840523|United Kingdom|BA.1.17.2|2022-02-05	99,64
EPI_ISL_9841398	BA.1.1	BA.1.1	BA.1.1_3	MILK-35BAFDD|EPI_ISL_9841398|United Kingdom|BA.1.1|2022-02-06	99,64
EPI_ISL_9841562	BA.1.1	BA.1.1	BA.1.1_3	MILK-35B579E|EPI_ISL_9841562|United Kingdom|BA.1.1|2022-02-07	99,64
EPI_ISL_9842142	BA.2	A	A_14	MILK-35BFEEA|EPI_ISL_9842142|United Kingdom|BA.2|2022-02-04	99,81
EPI_ISL_9844816	BA.2	A	A_1	DL-IGIB1120220300802TV|EPI_ISL_9844816|India|BA.2|2022-01	75,77
EPI_ISL_9844821	B.1.1.529	B.1.474	B.1.474	HR-IGIB1210606800555047TV|EPI_ISL_9844821|India|B.1.1.529|2022-02	77,03
EPI_ISL_9844857	BA.2	A	A_1	HR-IGIB1210607600583182TV|EPI_ISL_9844857|India|BA.2|2022-01	77,43
EPI_ISL_9847047	BA.1.1.2	BA.1.1	BA.1.1_3	TKYkbm1371|EPI_ISL_9847047|Japan|BA.1.1.2|2022-01-11	99,64
EPI_ISL_9848527	BA.1.17.2	BA.1.15	BA.1.15	NORT-YNBJO1|EPI_ISL_9848527|United Kingdom|BA.1.17.2|2022-01-13	90,98

EPI_ISL_9848830	BA.2.10	BA.2	BA.2_1	NPHL-S-1084|EPI_ISL_9848830|Nepal|BA.2.10|2022-01-17	94,54
EPI_ISL_9849434	BA.1.15	BA.1.15	BA.1.15	PHEP-YYRYO6X|EPI_ISL_9849434|United Kingdom|BA.1.15|2022-01-04	84,49
EPI_ISL_9849933	BA.1.1.13	BA.1.1	BA.1.1_3	KK-NVRL-S22IRL00049882|EPI_ISL_9849933|Ireland|BA.1.1.13|2022-01-11	99,64
EPI_ISL_9850013	BA.1.18	BA.1	BA.1_4	LS-NVRL-S22IRL00062451|EPI_ISL_9850013|Ireland|BA.1.18|2022-01-17	99,64
EPI_ISL_9850806	BA.1.20	BA.1	BA.1_3	LNS6239782|EPI_ISL_9850806|Luxembourg|BA.1.20|2022-01-24	85,04
EPI_ISL_9851152	BA.1.1.1	BA.1.15	BA.1.15	LNS3659761|EPI_ISL_9851152|Luxembourg|BA.1.1.1|2022-01-28	87,33
EPI_ISL_9851772	BA.1.18	BA.1	BA.1_2	LNS8615723|EPI_ISL_9851772|Luxembourg|BA.1.18|2022-01-25	89,12
EPI_ISL_9852185	BA.2	BA.2	BA.2_1	LB-R00059-S016|EPI_ISL_9852185|Austria|BA.2|2022-02-03	96,74
EPI_ISL_9852801	BA.1.13.1	BA.1.15	BA.1.15	JK-GS-GSILab-885569|EPI_ISL_9852801|Indonesia|BA.1.13.1|2022-01-20	99,64
EPI_ISL_9853122	BA.1.1	BA.1.1	BA.1.1_2	AM-FIOCRUZ-ILMD2203195|EPI_ISL_9853122|Brazil|BA.1.1|2022-01-26	99,64
EPI_ISL_9853660	BA.1.1	BA.1	BA.1_4	LNS8273928|EPI_ISL_9853660|Luxembourg|BA.1.1|2022-01-24	84,14
EPI_ISL_9854952	BA.2	A	A_17	DCGC-362148|EPI_ISL_9854952|Denmark|BA.2|2022-02-06	99,81
EPI_ISL_9855900	BA.2.14	BA.2.14	BA.2.14	DCGC-362584|EPI_ISL_9855900|Denmark|BA.2.14|2022-02-05	99,81
EPI_ISL_9856860	BA.2	BA.2	BA.2_1	IPG-1260|EPI_ISL_9856860|Belgium|BA.2|2022-02-04	84,20
EPI_ISL_9858999	BA.1.1	BA.1.1	BA.1.1_2	NY-PRL-2022_0207_01E05|EPI_ISL_9858999|USA|BA.1.1|2022-02-03	99,64
EPI_ISL_9860274	BA.2	BA.2	BA.2_1	LB-R00060-S070_S70|EPI_ISL_9860274|Austria|BA.2|2022-02-04	95,17
EPI_ISL_9861970	BA.1.1	BA.1.1	BA.1.1_2	KLU-RII-MH57517S|EPI_ISL_9861970|Russia|BA.1.1|2022-01-20	90,98
EPI_ISL_9863160	BA.1	BA.1	BA.1_1	MA-CDCBI-CRSP_TYWS6GGADMDN3DWK|EPI_ISL_9863160|USA|BA.1|2022-02-01	90,06
EPI_ISL_9866063	BA.2	A	A_14	LSPA-360B0BB|EPI_ISL_9866063|United Kingdom|BA.2|2022-02-06	99,81
EPI_ISL_9866278	BA.1.17.2	BA.1.17.2	BA.1.17.2	QEUH-3608018|EPI_ISL_9866278|United Kingdom|BA.1.17.2|2022-02-07	96,24
EPI_ISL_9867182	BA.1.1	BA.1.1	BA.1.1_2	MILK-35F17E1|EPI_ISL_9867182|United Kingdom|BA.1.1|2022-02-06	99,64
EPI_ISL_9868333	BA.1	BA.1.15	BA.1.15	MILK-35D8BA8|EPI_ISL_9868333|United Kingdom|BA.1|2022-02-06	99,64
EPI_ISL_9868531	BA.1.1	BA.1.1	BA.1.1_2	DL-LNHD156|EPI_ISL_9868531|India|BA.1.1|2022-01-22	99,98
EPI_ISL_9869387	BA.1.1.14	BA.1.1	BA.1.1_3	NEWC-35DE4A7|EPI_ISL_9869387|United Kingdom|BA.1.1.14|2022-02-07	99,64
EPI_ISL_9869796	BA.1.1.15	BA.1	BA.1_2	ALDP-35FC26D|EPI_ISL_9869796|United Kingdom|BA.1.1.15|2022-02-06	99,64
EPI_ISL_9871386	BA.2	BA.2	BA.2_1	NB-PAMM-B05-0809|EPI_ISL_9871386|Netherlands|BA.2|2022-02-05	97,10
EPI_ISL_9871984	BA.1	BA.1.15	BA.1.15	MILK-35E55D7|EPI_ISL_9871984|United Kingdom|BA.1|2022-02-08	99,64
EPI_ISL_9875161	BA.1.1	BA.1.15	BA.1.15	CMX-INMEGEN-57-116|EPI_ISL_9875161|Mexico|BA.1.1|2022-02-01	89,93
EPI_ISL_9881553	BA.1.1	BA.1.1	BA.1.1_2	WA-S17960|EPI_ISL_9881553|USA|BA.1.1|2022-01-16	99,64
EPI_ISL_9882157	BA.1.1	BA.1.1	BA.1.1_2	IA-SHL-2038452|EPI_ISL_9882157|USA|BA.1.1|2022-02-07	99,64
EPI_ISL_9882236	BA.1.1	BA.1.15	BA.1.15	TX-TAMGHRC-L-72180-21R|EPI_ISL_9882236|USA|BA.1.1|2021-12-21	99,43
EPI_ISL_9886779	BA.1.17.2	BA.1.17.2	BA.1.17.2	BC-BCCDC-336514|EPI_ISL_9886779|Canada|BA.1.17.2|2022-01-18	99,64
EPI_ISL_9886873	BA.1	BA.1.15	BA.1.15	BC-BCCDC-340866|EPI_ISL_9886873|Canada|BA.1|2022-01-20	99,64
EPI_ISL_9888226	BA.1	BA.1	BA.1_4	BE-IFIK-6539-6282|EPI_ISL_9888226|Switzerland|BA.1|2022-02-07	99,64
EPI_ISL_9888678	BA.1.17.2	BA.1.17.2	BA.1.17.2	QEUH-3623F29|EPI_ISL_9888678|United Kingdom|BA.1.17.2|2022-02-09	99,64
EPI_ISL_9889622	BA.2	A	A_14	QEUH-362C4B8|EPI_ISL_9889622|United Kingdom|BA.2|2022-02-08	96,20
EPI_ISL_9892004	BA.1.15	BA.1.15	BA.1.15	QEUH-3609FA1|EPI_ISL_9892004|United Kingdom|BA.1.15|2022-02-08	99,64
EPI_ISL_9896281	BA.1	BA.1.19	BA.1.19	LSPA-361442C|EPI_ISL_9896281|United Kingdom|BA.1|2022-02-05	99,64
EPI_ISL_9896729	BA.1	BA.1.15	BA.1.15	ALDP-3615EB4|EPI_ISL_9896729|United Kingdom|BA.1|2022-02-07	99,64
EPI_ISL_9898398	BA.1.1	BA.1.1	BA.1.1_2	QEUH-35F5817|EPI_ISL_9898398|United Kingdom|BA.1.1|2022-02-06	99,64
EPI_ISL_9900425	BA.2	A	A_14	QEUH-35C8B32|EPI_ISL_9900425|United Kingdom|BA.2|2022-02-06	99,81
EPI_ISL_9900481	BA.1	BA.1	BA.1_4	QEUH-35C4134|EPI_ISL_9900481|United Kingdom|BA.1|2022-02-06	99,64
EPI_ISL_9900700	BA.2	BA.2	BA.2_1	DCGC-365300|EPI_ISL_9900700|Denmark|BA.2|2022-02-07	89,85
EPI_ISL_9900931	BA.1.1	BA.1.1	BA.1.1_3	QEUH-35BA59F|EPI_ISL_9900931|United Kingdom|BA.1.1|2022-02-05	99,64
EPI_ISL_9901347	BA.2.23	A	A_14	QEUH-35B4164|EPI_ISL_9901347|United Kingdom|BA.2.23|2022-02-05	99,81
EPI_ISL_9901528	BA.2.9	BA.2.12	BA.2.12	DCGC-365603|EPI_ISL_9901528|Denmark|BA.2.9|2022-02-07	94,71
EPI_ISL_9902370	BA.1.1	BA.1.1	BA.1.1_3	ICH-741119318|EPI_ISL_9902370|Israel|BA.1.1|2022-02-03	91,41
EPI_ISL_9902463	BA.1.1	BA.1	BA.1_4	ICH-741119206|EPI_ISL_9902463|Israel|BA.1.1|2022-02-03	91,17
EPI_ISL_9903080	BA.2.9	A	A_17	DCGC-365879|EPI_ISL_9903080|Denmark|BA.2.9|2022-01-19	97,10
EPI_ISL_9903740	BA.1	BA.1	BA.1_3	IDF-CERBAHC-0253569|EPI_ISL_9903740|France|BA.1|2022-02-05	96,85
EPI_ISL_9905060	BA.1.1	BA.1.1	BA.1.1_2	CT-HUVH-EX44889|EPI_ISL_9905060|Spain|BA.1.1|2022-02-09	96,79
EPI_ISL_9905758	BA.1.15	BA.1.15	BA.1.15	RJ-SMS-ICMR-INSACOG-TS-1332|EPI_ISL_9905758|India|BA.1.15|2021-12-26	96,32
EPI_ISL_9905843	BA.2	BA.2.12	BA.2.12	RJ-SMS-ICMR-INSACOG-TS-1577|EPI_ISL_9905843|India|BA.2|2021-12-29	94,83
EPI_ISL_9907274	BA.2	A	A_14	097297802|EPI_ISL_9907274|Poland|BA.2|2022-02-14	99,03
EPI_ISL_9907712	BA.2	A	A_17	BE-ChVir-LB-220211-8492|EPI_ISL_9907712|Germany|BA.2|2022-02-07	97,08
EPI_ISL_9908778	BA.1	BA.1	BA.1_4	PZH-GUM-12192|EPI_ISL_9908778|Poland|BA.1|2022-01-28	99,64
EPI_ISL_9908982	BA.2	A	A_14	PZH-GUM-10956|EPI_ISL_9908982|Poland|BA.2|2022-01-27	99,81
EPI_ISL_9909062	BA.1.1.18	BA.1.1	BA.1.1_2	CO-CDC-LC0512139|EPI_ISL_9909062|USA|BA.1.1.18|2022-01-17	99,64
EPI_ISL_9909657	BA.1.1	BA.1.1	BA.1.1_2	CL-COV23592|EPI_ISL_9909657|Spain|BA.1.1|2021-12-03	96,03
EPI_ISL_9909696	BA.1.17	BA.1	BA.1_4	CL-COV24226|EPI_ISL_9909696|Spain|BA.1.17|2022-01-20	96,03
EPI_ISL_9910072	BA.1.20	BA.1.15	BA.1.15	MN-CDC-LC0513029|EPI_ISL_9910072|USA|BA.1.20|2022-01-19	99,64
EPI_ISL_9910438	BA.1.1	BA.1.1	BA.1.1_2	NY-CDC-LC0507443|EPI_ISL_9910438|USA|BA.1.1|2022-01-19	99,64
EPI_ISL_9911565	BA.1.18	A	A_1	DC-DFS-PHL-05384|EPI_ISL_9911565|USA|BA.1.18|2021-12-31	57,39
EPI_ISL_9911609	BA.1.1	BA.1.1	BA.1.1_2	OK-CDC-LC0516121|EPI_ISL_9911609|USA|BA.1.1|2022-01-21	99,12
EPI_ISL_9911824	BA.1	BA.1.1	BA.1.1_3	TG-GMCH-ICMR-INSACOG-C-343968|EPI_ISL_9911824|India|BA.1|2022-02-05	84,87
EPI_ISL_9913606	BA.1.1	BA.1.1	BA.1.1_2	VA-CDC-LC0509085|EPI_ISL_9913606|USA|BA.1.1|2022-01-25	99,64
EPI_ISL_9915900	BA.1.1	BA.1.1	BA.1.1_3	NJ-CDC-LC0508542|EPI_ISL_9915900|USA|BA.1.1|2022-01-26	99,64
EPI_ISL_9916195	BA.1.1	BA.1.1	BA.1.1_2	OCC-CHU-TLS-220161304301|EPI_ISL_9916195|France|BA.1.1|2022-01-16	82,48
EPI_ISL_9918573	BA.1.1.2	BA.1.1	BA.1.1_3	CT-CDC-LC0511256|EPI_ISL_9918573|USA|BA.1.1.2|2022-01-28	99,64
EPI_ISL_9919902	BA.1.17.2	BA.1.17.2	BA.1.17.2	IBT-LSC-VU_r40_38|EPI_ISL_9919902|Lithuania|BA.1.17.2|2022-01-31	99,56
EPI_ISL_9920179	BA.1	BA.1	BA.1_4	GU-CDC-2-5508077|EPI_ISL_9920179|Guam|BA.1|2022-01-07	99,64
EPI_ISL_9921490	BA.1.1	BA.1.1	BA.1.1_2	CA-CDPH-3000302387|EPI_ISL_9921490|USA|BA.1.1|2022-01-15	96,20
EPI_ISL_9921539	BA.1	BA.1	BA.1_4	CA-CDPH-3000302802|EPI_ISL_9921539|USA|BA.1|2022-01-20	81,07
EPI_ISL_9922418	BA.1.1	BA.1.1	BA.1.1_2	UT-UPHL-220213380929|EPI_ISL_9922418|USA|BA.1.1|2022-01-03	99,64
EPI_ISL_9925330	BA.1.1	BA.1.1	BA.1.1_2	WI-WSLH-2204136|EPI_ISL_9925330|USA|BA.1.1|2022-01-26	84,28
EPI_ISL_9925888	BA.1.1	BA.1.1	BA.1.1_2	MN-CDC-IBX153399164548|EPI_ISL_9925888|USA|BA.1.1|2022-01-17	93,99
EPI_ISL_9927351	BA.1.1	BA.1.1	BA.1.1_2	NM-CDC-ASC210683871|EPI_ISL_9927351|USA|BA.1.1|2022-01-25	99,64
EPI_ISL_9928247	BA.1.1	BA.1.1	BA.1.1_2	WV-CDC-ASC210546176|EPI_ISL_9928247|USA|BA.1.1|2022-01-28	99,64
EPI_ISL_9928558	BA.1.1	BA.1.1	BA.1.1_2	MT-CDC-ASC210545871|EPI_ISL_9928558|USA|BA.1.1|2022-01-28	99,64
EPI_ISL_9928603	BA.1.1	BA.1.1	BA.1.1_2	CA-SLOPH-C0960|EPI_ISL_9928603|USA|BA.1.1|2022-01-30	99,64
EPI_ISL_9930466	BA.1.1	BA.1.1	BA.1.1_2	CA-HLX-STM-DUVRCPQF9|EPI_ISL_9930466|USA|BA.1.1|2022-01-17	99,62
EPI_ISL_9931376	BA.1.1	BA.1.1	BA.1.1_2	MI-CDC-STM-WXQGXSCHT|EPI_ISL_9931376|USA|BA.1.1|2022-01-22	99,64
EPI_ISL_9931539	BA.1.1	BA.1.1	BA.1.1_2	FL-CDC-STM-727E5B7BG|EPI_ISL_9931539|USA|BA.1.1|2022-01-22	99,64
EPI_ISL_9933791	BA.1.15	BA.1.15	BA.1.15	MI-CDC-ASC210688266|EPI_ISL_9933791|USA|BA.1.15|2022-01-28	99,64
EPI_ISL_9934387	BA.1.1	BA.1.1	BA.1.1_2	OH-CDC-ASC210687481|EPI_ISL_9934387|USA|BA.1.1|2022-01-29	96,89
EPI_ISL_9934472	BA.1.15.2	BA.1.15.2	BA.1.15.2	MI-CDC-ASC210688035|EPI_ISL_9934472|USA|BA.1.15.2|2022-01-29	99,64
EPI_ISL_9934598	BA.1.1	BA.1	BA.1_4	WA-CDC-ASC210594580|EPI_ISL_9934598|USA|BA.1.1|2022-01-30	88,42
EPI_ISL_9934785	BA.1.1	BA.1.1	BA.1.1_2	MN-CDC-ASC210687519|EPI_ISL_9934785|USA|BA.1.1|2022-01-30	99,64
EPI_ISL_9935252	BA.1.15.2	BA.1.15	BA.1.15	WI-CDC-MMB13588323|EPI_ISL_9935252|USA|BA.1.15.2|2022-01-17	97,29
EPI_ISL_9940252	BA.1.1	BA.1	BA.1_4	OR-UW-22011904596|EPI_ISL_9940252|USA|BA.1.1|2022-01-19	87,62
EPI_ISL_9941583	BA.1.1	BA.1.1	BA.1.1_2	CA-CDC-FG-261885|EPI_ISL_9941583|USA|BA.1.1|2022-02-01	92,92
EPI_ISL_9941770	BA.1	BA.1.15	BA.1.15	NJ-CDC-ASC210689317|EPI_ISL_9941770|USA|BA.1|2022-01-31	99,64
EPI_ISL_9942287	BA.1.15	BA.1	BA.1_4	OR-CDC-QDX33311140|EPI_ISL_9942287|USA|BA.1.15|2022-01-26	99,64
EPI_ISL_9943105	BA.1.15	BA.1	BA.1_4	NC-CDC-MMB13943477|EPI_ISL_9943105|USA|BA.1.15|2022-01-27	94,68
EPI_ISL_9943443	BA.1.1.18	BA.1.1	BA.1.1_2	CO-CDC-MMB13955215|EPI_ISL_9943443|USA|BA.1.1.18|2022-01-27	97,20
EPI_ISL_9943864	BA.1.1	BA.1.1	BA.1.1_2	CA-CDC-FG-263829|EPI_ISL_9943864|USA|BA.1.1|2022-02-04	89,94
EPI_ISL_9946735	BA.1.1	BA.1.1	BA.1.1_2	FL-CDC-STM-RX77BQAXF|EPI_ISL_9946735|USA|BA.1.1|2022-01-29	99,64
EPI_ISL_9948025	BA.1.1.18	BA.1.1	BA.1.1_2	CO-CDC-QDX33353599|EPI_ISL_9948025|USA|BA.1.1.18|2022-01-27	99,64
EPI_ISL_9948434	BA.1.1.18	BA.1.1	BA.1.1_3	KS-CDC-QDX33398264|EPI_ISL_9948434|USA|BA.1.1.18|2022-01-30	99,64
EPI_ISL_9948462	BA.1.1	BA.1.1	BA.1.1_2	KS-CDC-QDX33398293|EPI_ISL_9948462|USA|BA.1.1|2022-01-30	99,64
EPI_ISL_9948618	BA.1.1.18	BA.1.1	BA.1.1_2	CA-CDC-FG-254267|EPI_ISL_9948618|USA|BA.1.1.18|2022-01-24	99,52

EPI_ISL_9949221	BA.1.18	BA.1	BA.1_4	CT-HUGTiPM078XX1H4|EPI_ISL_9949221|Spain|BA.1.18|2022-02-04	99,64
EPI_ISL_9949305	BA.1.1	BA.1.1	BA.1.1_2	CA-CDC-FG-262554|EPI_ISL_9949305|USA|BA.1.1|2022-01-31	98,09
EPI_ISL_9951232	BA.1.1	BA.1.1	BA.1.1_2	FL-CDC-ASC210687067|EPI_ISL_9951232|USA|BA.1.1|2022-01-28	96,60
EPI_ISL_9951991	BA.1.1	BA.1.1	BA.1.1_2	FL-CDC-ASC210688575|EPI_ISL_9951991|USA|BA.1.1|2022-01-30	99,64
EPI_ISL_9952973	BA.1.1	BA.1	BA.1_4	GA-CDC-MMB13996020|EPI_ISL_9952973|USA|BA.1.1|2022-01-28	93,34
EPI_ISL_9953624	BA.1.1.2	BA.1.15	BA.1.15	CA-CDC-FG-262824|EPI_ISL_9953624|USA|BA.1.1.2|2022-02-02	95,40
EPI_ISL_9954543	BA.1.1	BA.1.1	BA.1.1_2	CA-CDC-FG-264175|EPI_ISL_9954543|USA|BA.1.1|2022-02-04	90,99
EPI_ISL_9955964	BA.1.1	BA.1.1	BA.1.1_2	OK-CDC-ASC210647434|EPI_ISL_9955964|USA|BA.1.1|2022-02-01	99,64
EPI_ISL_9956193	BA.1.1	BA.1.1	BA.1.1_2	RI-CDC-ASC210648667|EPI_ISL_9956193|USA|BA.1.1|2022-02-02	99,64
EPI_ISL_9956526	BA.1.1	BA.1.1	BA.1.1_2	ME-CDC-QDX33398625|EPI_ISL_9956526|USA|BA.1.1|2022-01-25	99,64
EPI_ISL_9958740	BA.1.1	BA.1	BA.1_4	58099|EPI_ISL_9958740|Slovenia|BA.1.1|2022-01-14	99,66
EPI_ISL_9959264	BA.1.1	BA.1.1	BA.1.1_2	WV-CDC-LC0519395|EPI_ISL_9959264|USA|BA.1.1|2022-01-25	99,64
EPI_ISL_9960169	BA.1	BA.1	BA.1_4	KY-CDC-LC0524759|EPI_ISL_9960169|USA|BA.1|2022-01-26	95,69
EPI_ISL_9961422	BA.2.9	A	A_17	SUS0009252|EPI_ISL_9961422|Sweden|BA.2.9|2022-01-30	99,81
EPI_ISL_9961894	BA.2.9	BA.2	BA.2_1	SUS0009422|EPI_ISL_9961894|Sweden|BA.2.9|2022-02-01	99,81
EPI_ISL_9963304	BA.1.17.2	BA.1.17.2	BA.1.17.2	RC0358080|EPI_ISL_9963304|Estonia|BA.1.17.2|2022-01-21	99,64
EPI_ISL_9963534	BA.1.1	BA.1.1	BA.1.1_2	NORT-YNBSXWS|EPI_ISL_9963534|United Kingdom|BA.1.1|2022	99,64
EPI_ISL_9967700	BA.1.15	BA.1.15	BA.1.15	UT-CDC-LC0526588|EPI_ISL_9967700|USA|BA.1.15|2022-01-31	99,35
EPI_ISL_9969572	BA.1.1	BA.1.1	BA.1.1_2	CA-CDC-LC0521772|EPI_ISL_9969572|USA|BA.1.1|2022-02-02	87,60
EPI_ISL_9970990	BA.1.13	BA.1.15	BA.1.15	CVL-HMN-22012270799|EPI_ISL_9970990|France|BA.1.13|2022-01-03	89,62
EPI_ISL_9972937	BA.2.9	A	A_17	DCGC-368362|EPI_ISL_9972937|Denmark|BA.2.9|2022-02-08	99,79
EPI_ISL_9973246	BA.1.1	BA.1.1	BA.1.1_3	THL-202205655|EPI_ISL_9973246|Finland|BA.1.1|2022-01-19	99,64
EPI_ISL_9973653	BA.1.1	BA.1.1	BA.1.1_3	BFC-HMN-22012200578|EPI_ISL_9973653|France|BA.1.1|2022-01-10	77,64
EPI_ISL_9975594	BA.1.1	BA.1.1	BA.1.1_2	28123|EPI_ISL_9975594|Croatia|BA.1.1|2022-01-25	99,62
EPI_ISL_9976893	BA.1.1	BA.1.1	BA.1.1_2	MI-CDC-STM-KRHDB33X4|EPI_ISL_9976893|USA|BA.1.1|2022-02-01	99,64
EPI_ISL_9976933	BA.1.1	BA.1.1	BA.1.1_2	FL-CDC-STM-ZE4X6UN4R|EPI_ISL_9976933|USA|BA.1.1|2022-02-01	99,64
EPI_ISL_9978385	BA.1.15	BA.1	BA.1_4	CO-CDC-MMB13691887|EPI_ISL_9978385|USA|BA.1.15|2022-01-19	93,44
EPI_ISL_9978616	BA.1.1.18	BA.1.1	BA.1.1_2	CO-CDC-MMB13989845|EPI_ISL_9978616|USA|BA.1.1.18|2022-01-28	89,70
EPI_ISL_9979233	BA.1.1.1	BA.1.1	BA.1.1_3	GES-HMN-22022030173|EPI_ISL_9979233|France|BA.1.1.1|2022-01-24	85,46
EPI_ISL_9979341	BA.1	BA.1.14.1	BA.1.14.1	IDF-HMN-22022070700|EPI_ISL_9979341|France|BA.1|2022-02-07	89,33
EPI_ISL_9980499	BA.1.15	BA.1	BA.1_3	TX-CDC-FG-265838|EPI_ISL_9980499|USA|BA.1.15|2022-02-03	95,55
EPI_ISL_9985529	BA.2	A	A_17	PZH-UMB-14945|EPI_ISL_9985529|Poland|BA.2|2022-01-27	96,99
EPI_ISL_9986290	BA.2	BA.2	BA.2_1	VS-ICH-2220208576|EPI_ISL_9986290|Switzerland|BA.2|2022-02-03	99,81
EPI_ISL_9986888	BA.1.1.18	BA.1.1	BA.1.1_2	CO-CDC-MMB13754530|EPI_ISL_9986888|USA|BA.1.1.18|2022-01-21	97,29
EPI_ISL_9987763	BA.1.15	BA.1.15	BA.1.15	CA-SEARCH-115591|EPI_ISL_9987763|USA|BA.1.15|2022-01-02	99,64
EPI_ISL_9990032	BA.1	BA.1	BA.1_3	MILK-366DB2F|EPI_ISL_9990032|United Kingdom|BA.1|2022-02-10	99,64
EPI_ISL_9990364	BA.2.10	BA.2	BA.2_1	MILK-365A5CD|EPI_ISL_9990364|United Kingdom|BA.2.10|2022-02-11	89,60
EPI_ISL_9990715	BA.2	A	A_14	QEUH-36707E9|EPI_ISL_9990715|United Kingdom|BA.2|2022-02-10	99,79
EPI_ISL_9990975	BA.1.1	BA.1.1	BA.1.1_3	QEUH-3672FC1|EPI_ISL_9990975|United Kingdom|BA.1.1|2022-02-10	99,64
EPI_ISL_9991837	BA.2	A	A_14	MILK-367824A|EPI_ISL_9991837|United Kingdom|BA.2|2022-02-11	99,81
EPI_ISL_9992176	BA.2.1	A	A_14	LSPA-3663084|EPI_ISL_9992176|United Kingdom|BA.2.1|2022-02-10	99,81
EPI_ISL_9992620	BA.1	BA.1	BA.1_4	ALDP-3661F7A|EPI_ISL_9992620|United Kingdom|BA.1|2022-02-09	99,60
EPI_ISL_9994408	BA.1	BA.1.15	BA.1.15	MILK-3662832|EPI_ISL_9994408|United Kingdom|BA.1|2022-02-10	99,64
EPI_ISL_9995095	BA.2	A	A_14	MILK-3656FB4|EPI_ISL_9995095|United Kingdom|BA.2|2022-02-09	99,81
EPI_ISL_9995989	BA.1.1	BA.1.1	BA.1.1_2	QEUH-3649330|EPI_ISL_9995989|United Kingdom|BA.1.1|2022-02-10	99,64
EPI_ISL_9996342	BA.1.1	BA.1.1	BA.1.1_3	ALDP-36488BA|EPI_ISL_9996342|United Kingdom|BA.1.1|2022-02-08	99,64
EPI_ISL_9997821	BA.2.12	A	A_4	MILK-3655753|EPI_ISL_9997821|United Kingdom|BA.2.12|2022-02-10	99,81
EPI_ISL_9997929	BA.2	BA.2	BA.2_1	MILK-365D919|EPI_ISL_9997929|United Kingdom|BA.2|2022-02-10	99,81
EPI_ISL_9999185	BA.1.1.13	BA.1.1	BA.1.1_3	MILK-36539B9|EPI_ISL_9999185|United Kingdom|BA.1.1.13|2022-02-10	99,64
EPI_ISL_9999622	BA.1.1	BA.1.1	BA.1.1_2	MILK-364B4FF|EPI_ISL_9999622|United Kingdom|BA.1.1|2022-02-10	99,64

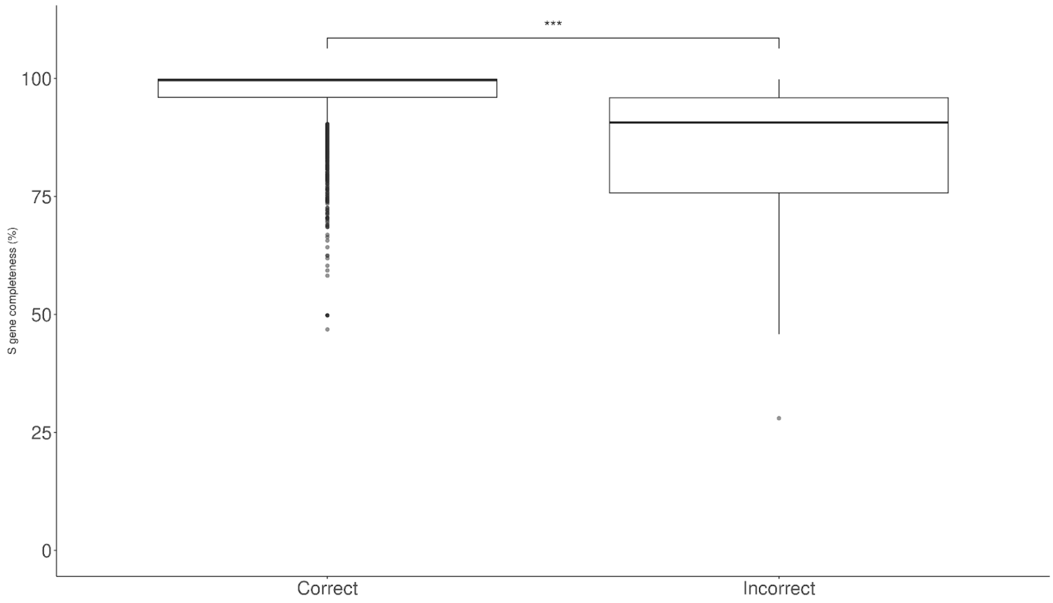

Figure S7: Differences between correct and incorrect PANGO lineage assignments of the Omicron dataset. Significant differences (p-value ≤ 0.001) are observed between correct and incorrect PANGO lineage assignments.

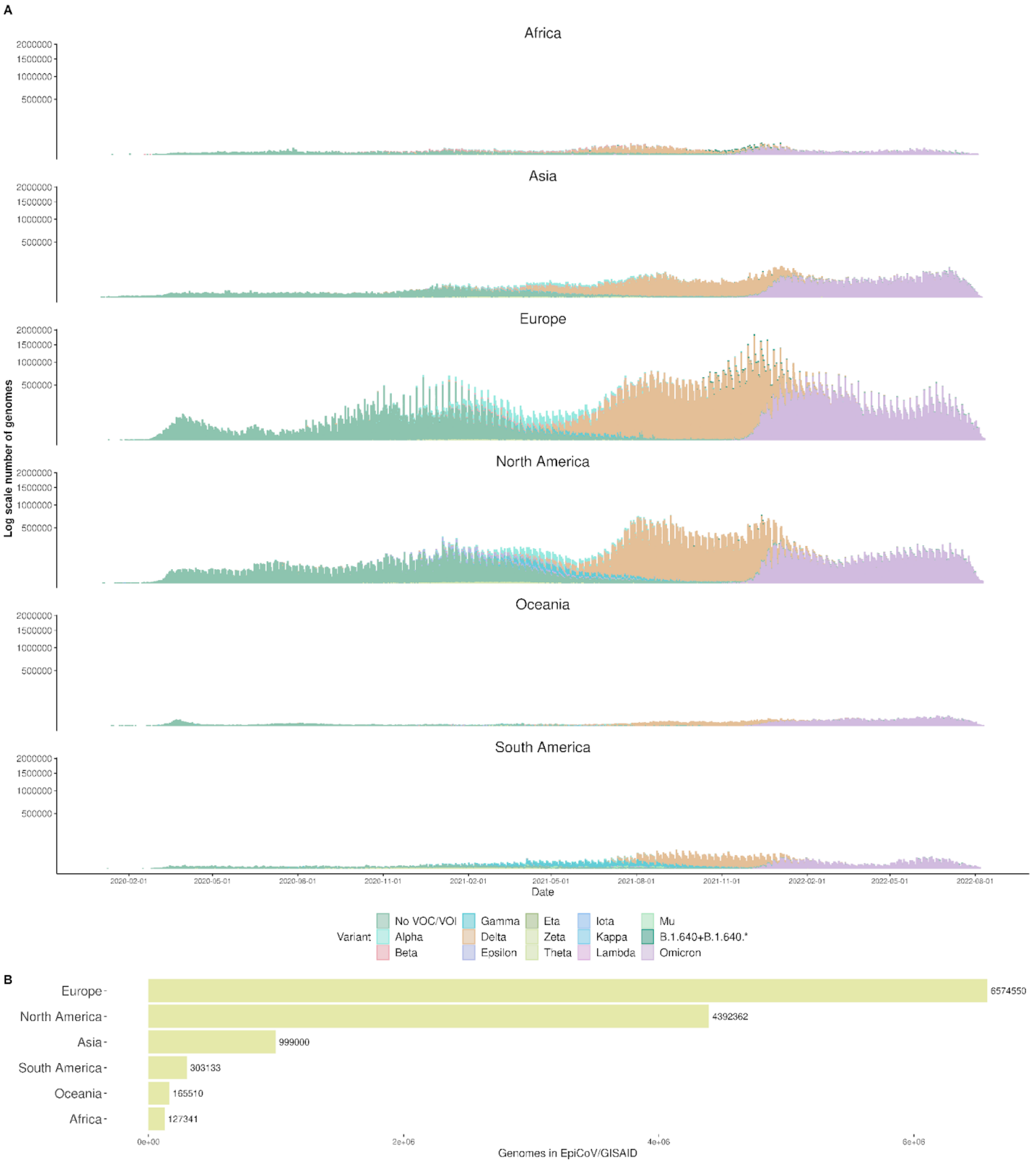

Figure S8: SARS-CoV-2 data representation in EpiCoV/GISAID database from January 2020 to August 2022 from each continent. **A**) EpiCoV/GISAID variant distribution per continent. **B)** Absolute number of genomes per continent

Table S6: Estimated time of workflow execution of the S gene based and WGS sequencing protocol

	Fast-S*	Standard-S*	WGS*
**Step**	**Total**	**Total**	**Total**
Reverse transcription	30 min	30 min	30 min
whole genome tiled amplification			240 min
S gene tiled amplification	80 min	80 min	
S gene amplicon dilution		20 min	
whole genome amplicon dilution			30 min
PCR barcoding of S gene amplicons	60 min	80 min	
Pooling and clean-up	30 min	30 min	30 min
End prep and clean-up	30 min	30 min	
AMX adapter ligation and final clean-up	60 min	60 min	
RAP adapter attachment			10 min
Flow cell priming and loading	15 min	15 min	15 min
Library prep	~5 hrs	~6 hrs	~5 hrs
Sequencing run	~12-18 hrs	~12-18 hrs	~24-46 hrs
Analysis and report	~1 hr	~1 hr	~2 hr

Variation in library prep and analysis time are expected to vary in different settings

Table S7: Cost estimation per run and per sample using the Standard-S protocol*

	reagent or unit cost (USD)	reaction or units	USD/reaction or units	reaction or units per run	USD total
PCR Barcoding Expansion Pack 1-96 (EXP-PBC096)	1700	2304	0.70	96.00	70.80
Ligation Sequencing Kit (SQK-LSK109)	599	6	99.80	1.00	99.80
NEB Blunt/TA Ligase Master Mix (M0367)	410	250	1.60	1.00	1.60
NEBNext FFPE Repair Mix (M6630)	592	96	6.20	1.00	6.20
NEBNext Ultra II End repair/dA-tailing Module (E7546)	795	96	8.30	1.00	8.30
NEBNext Quick Ligation Module (E6056)	1310	100	13.10	1.00	13.10
**Q5® High-Fidelity 2X Master Mix**	**757**	**2000**	**0.40**	**384.00**	**145.30**
Tiled primers	300	1000	0.30	96.00	28.80
AMpure XP beads	1600	60	26.70	0.50	13.30
1.5 ml Eppendorf DNA LoBind tubes	53	250	0.20	2.00	0.40
PCR tubes with dettachable cap	319	250	1.30	48.00	61.20
Nuclease-free water (e.g. ThermoFisher, cat # AM9937)	9	500	0.00	10.00	0.20
Freshly prepared 70% ethanol in nuclease-free water	61	2500	0.00	10.00	0.20
P1000 pipette and tips	103	10	10.30	0.50	5.20
P200 pipette and tips	94	10	9.40	0.50	4.70
P20 pipette and tips	91	10	9.10	4.00	36.40
P10 pipette and tips	91	10	9.10	2.00	18.20
Flow cell FLO-MIN106D** (2 runs)	475-900	1	475-900	0.5	238-450
Flow Cell Wash Kit XL	432	48	9	1	9
				**Total cost per run**	**761-973**
				**Total cost per sample**	**8-10**

Table S8: Cost estimation per run (96 samples) and per sample using the Fast-S protocol with the FLO-MIN106D ONT flow cell*

	reagent or unit cost (USD)	reaction or units	USD/reaction or units	reaction or units per run	USD total
PCR Barcoding Expansion Pack 1-96 (EXP-PBC096)	1700	2304	0.70	192.00	141.70
Ligation Sequencing Kit (SQK-LSK109)	599	6	99.80	1.00	99.80
NEB Blunt/TA Ligase Master Mix (M0367)	410	250	1.60	1.00	1.60
NEBNext FFPE Repair Mix (M6630)	592	96	6.20	1.00	6.20
NEBNext Ultra II End repair/dA-tailing Module (E7546)	795	96	8.30	1.00	8.30
NEBNext Quick Ligation Module (E6056)	1310	100	13.10	1.00	13.10
LongAmp® Taq 2X Master Mix	562	2000	0.30	96.00	27.00
Tiled primers	300	1000	0.30	192.00	57.60
AMpure XP beads	1600	60	26.70	0.50	13.30
1.5 ml Eppendorf DNA LoBind tubes	53	250	0.21	2.00	0.40
PCR tubes with dettachable cap	319	250	1.28	24.00	30.60
Nuclease-free water (e.g. ThermoFisher, cat # AM9937)	9	500	0.02	10.00	0.20
Freshly prepared 70% ethanol in nuclease-free water	60,53	2500	0.02	10.00	0.20
P1000 pipette and tips	103	10	10.30	0.50	5.20
P200 pipette and tips	94	10	9.40	0.50	4.70
P20 pipette and tips	91	10	9.10	2.00	18.20
P10 pipette and tips	91	10	9.10	2.00	18.20
Flow cell FLO-MIN106D** (2 runs)	475 - 900	1	475 - 900	0.5	237-450
Flow Cell Wash Kit XL	432	48	9	1	9
				**Total cost per run**	**693-905**
				**Total cost per sample**	**7.2-9.4**

Table S9: Cost estimation per run (96 samples) and per sample using the Fast-S protocol with the FLO-FLG001 ONT flow cell*

	reagent cost (USD)	reaction or units	USD/reaction or units	reaction or units per run	USD total
PCR Barcoding Expansion Pack 1-96 (EXP-PBC096)	1700	2304	0.70	192.00	141.70
Ligation Sequencing Kit (SQK-LSK109)	599	6	99.80	1.00	99.80
NEB Blunt/TA Ligase Master Mix (M0367)	410	250	1.60	1.00	1.60
NEBNext FFPE Repair Mix (M6630)	592	96	6.20	1.00	6.20
NEBNext Ultra II End repair/dA-tailing Module (E7546)	795	96	8.30	1.00	8.30
NEBNext Quick Ligation Module (E6056)	1310	100	13.10	1.00	13.10
LongAmp® Taq 2X Master Mix	562	2000	0.30	96.00	27.00
Tiled primers	300	1000	0.30	192.00	57.60
AMpure XP beads	1600	60	26.70	0.50	13.30
1.5 ml Eppendorf DNA LoBind tubes	53	250	0.21	2.00	0.40
PCR tubes with dettachable cap	319	250	1.28	24.00	30.60
Nuclease-free water (e.g. ThermoFisher, cat # AM9937)	9	500	0.02	10.00	0.20
Freshly prepared 70% ethanol in nuclease-free water	61	2500	0.02	10.00	0.20
P1000 pipette and tips	103	10	10.30	0.50	5.20
P200 pipette and tips	94	10	9.40	0.50	4.70
P20 pipette and tips	91	10	9.10	2.00	18.20
P10 pipette and tips	91	10	9.10	2.00	18.20
Flow cell FLO-FLG001 (Flongle)**	90	1	90	1	90
				**Total cost per run**	**536**
				**Total cost per sample**	**5.6**

Table S10: Cost estimation per run and per sample using SARS-CoV-2 WGS

Material	reagent or unit cost (USD)	reaction or units	USD/reaction or units	reaction or units per run	USD total
Midnight RT PCR Expansion (EXP-MRT001)	2880	576	5	96	480
Ethanol	60.5	2500	0	8	0.2
Nuclease-free water	9	500	0	10	0.2
Qubit dsDNA HS Assay Kit (ThermoFisher Q32851)	335	500	0.7	5	3.4
Qubit™ Assay Tubes (ThermoFisher Q32856)	95	500	0.2	5	1
1.5 ml Eppendorf DNA LoBind tubes	53	250	0.2	3	0.6
2 ml Eppendorf DNA LoBind tubes	53	250	0.2	1	0.2
5 ml Eppendorf DNA LoBind tubes	78	200	0.4	1	0.4
Eppendorf twin.tec® PCR plate 96 LoBind, semi-skirted (Cat # 0030129504) with PCR seals	161	25	6.4	3	19.3
P1000 pipette and tips	103	10	10.3	0.5	5.2
P200 pipette and tips	94	10	9.4	1	9.4
P20 pipette and tips	91	10	9.1	2	18.2
P10 pipette and tips	91	10	9.1	2	18.2
Flow cell FLO-MIN106D**	475-900			1	475-900
Flow Cell Wash Kit XL	432	48	9	1	9
				**Total cost per run**	**1040-1465**
				**Total cost per sample**	**11-15**

*Variation of costs are expected according to the location

**Flow cell cost depends of the number of flow cells purchased
