# Supplementary material for: Fast and cost-effective SARS-CoV-2 variant detection using Oxford Nanopore full-length spike gene sequencing
Source: Microb Genom. 2023 May 18;9(5):mgen001013. doi: 10.1099/mgen.0.001013 (PMC10272875; doi:10.1099/mgen.0.001013)
Supplement: Supplementary material 2 [file mgen-9-1013-s002.pdf]

SUPPLEMENTAL TABLE

Data Availability

GISAID Identifier: EPI_SET_220929wd
doi: 10.55876/gis8.220929wd

All genome sequences and associated metadata in this dataset are published in GISAID's EpiCoV database. To view the contributors of each individual sequence with details such as accession number, Virus name, Collection date, Originating Lab and Submitting Lab and the list of Authors, visit 10.55876/gis8.220929wd

Data Snapshot

- EPI_SET_220929wd is composed of 4,999 individual genome sequences.
- The collection dates range from 2021-11-16 to 2022-07-09;
- Data were collected in 90 countries and territories;
- All sequences in this dataset are compared relative to hCoV-19/Wuhan/WIV04/2019 (WIV04), the official reference sequence employed by GISAID (EPI_ISL_402124). Learn more at https://gisaid.org/WIV04.
